# Diastereodivergent *cis*- and *trans*-fused [4 + 2] annulations of cyclic 1,3-dienes and 1-azadienes *via* ligand-controlled palladium catalysis†

**DOI:** 10.1039/d2sc06813c

**Published:** 2023-01-17

**Authors:** Yuan Hu, Jin-Yu Huang, Ru-Jie Yan, Zhi-Chao Chen, Qin Ouyang, Wei Du, Ying-Chun Chen

**Affiliations:** a Key Laboratory of Drug-Targeting and Drug Delivery System of the Education Ministry and Sichuan Province, Sichuan Research Center for Drug Precision Industrial Technology, West China School of Pharmacy, Sichuan University Chengdu 610041 China chenzhichao@scu.edu.cn ycchen@scu.edu.cn +86 28 85502609; b College of Pharmacy, Third Military Medical University Shapingba Chongqing 400038 China

## Abstract

Despite the blossoming of reports of diastereodivergent synthesis over the past years, switchable control of the stereochemistry of the bridgehead atoms of the fused frameworks has been significantly underdeveloped. Here we disclose the ability of Pd^0^-π-Lewis base catalysis to finely reverse the concerted inverse-electron-demand aza-Diels–Alder cycloaddition reaction between cyclic 1,3-dienes and aurone-derived 1-azadienes. In contrast, the *in situ*-formed HOMO-energy-increased Pd^0^-η^2^-complexes of cyclic 1,3-dienes underwent a cascade vinylogous Michael addition/allylic amination process with 1-azadienes. Moreover, judicious selection of chiral ligands allowed for switchable diastereodivergent [4 + 2] annulations to be accomplished, resulting in the construction of both *cis*- and *trans*-fused tetrahydropyridine architectures in high yields with moderate to excellent stereoselectivity levels. A variety of acyclic 1,3-dienes and 1-heterodienes were also applied, and furnished a structural diversity of enantioenriched frameworks.

## Abstract 2

Diastereodivergent and asymmetric [4 + 2] annulations between cyclic 1,3-dienes and 1-azadienes *via* ligand-controlled Pd catalysis have been realized, furnishing both *cis*- and *trans*-fused tetrahydropyridines in good yields and stereoselectivity levels.

## Graphical abstract

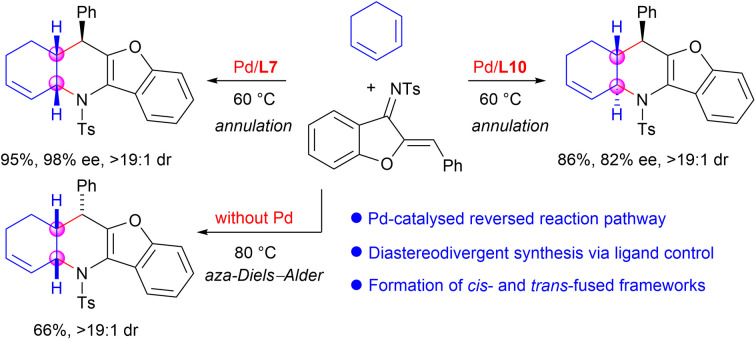

## Introduction

**Scheme 1:**
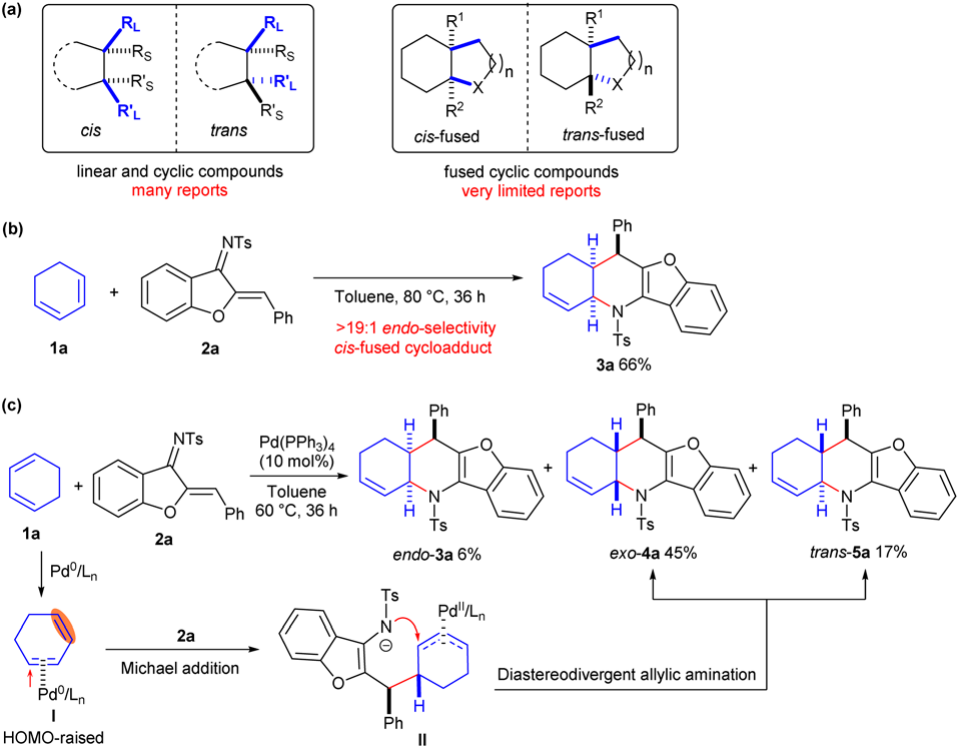
Diastereodivergent construction of *cis*- and *trans*-fused cyclic frameworks. (a) Diastereodivergent synthesis of various frameworks. (b) Uncatalysed inverse-electron-demand aza-Diels–Alder reaction. (c) Diastereodivergent [4 + 2] annulations enabled by Pd^0^-π-Lewis base catalysis.

As different stereoisomers usually display distinct biological activities, precise construction of multiple optically active isomers has always been an attractive but challenging task in organic chemistry and the drug discovery field.^1^ Diastereodivergent synthesis, which enables producing diverse diastereomers from the same set of starting materials just by varying reaction conditions, has emerged as a flourishing area owing to its high efficiency and great versatility.^2^ Several strategies, including tuning the catalysts,^3^ ligands,^4^ solvents^5^ and additives,^6^*etc*,^7^ have demonstrated to be useful for furnishing divergently many diastereomers with linear and cyclic structures. However, there have been few well-developed studies showing an ability to switch the stereochemistry of the bridgehead atoms in fused structures, and only a few examples have been uncovered for constructing both *cis*- and *trans*-fused bicyclic or polycyclic frameworks (Scheme 1a).^8^

The application of cyclic alkenes as dienophile partners in a Diels–Alder cycloaddition reaction provides a very straight-forward strategy to access fused skeletons; however, *cis*-fused products are usually obtained due to the inherent concerted reaction mechanism.^9^ As illustrated in Scheme 1b, exclusive *endo*-selectivity was observed in the uncatalysed inverse-electron-demand Diels–Alder reaction between 1,3-cyclohexadiene 1a and aurone-derived 1-azadiene 2a,^10^ resulting in the formation of *cis*-fused polycyclic product 3a in a moderate yield after heating the reaction mixture at 80 °C for 36 h. Recently, our group demonstrated that Pd^0^ could act as a π-Lewis base catalyst to increase the highest occupied molecular orbital (HOMO) energy of linear 1,3-dienes through η^2^-coordination, thus promoting asymmetric Friedel–Crafts-type vinylogous addition to *N*-sulfonylimines enantioselectively.^11^ We envisaged that the Pd^0^-π-Lewis base catalysis would facilitate the asymmetric assembly of 1,3-cyclohexadiene 1a and 1-azadiene 2a by generating the high-HOMO-energy complex I. The reaction of 1a with 1-azadiene 2a indeed was promoted at a lower temperature (60 °C) in the presence of catalytic amounts of Pd(PPh_3_)_4_; intriguingly, *endo-cis*-3a was produced in a low yield, whereas diastereomeric *exo-cis* cycloadduct 4a and unexpected *trans*-5a were predominantly produced (from ^1^H NMR analysis).^12^ These experimental results suggested that Pd^0^ might render 1,3-cyclohexadiene 1a more nucleophilic upon π-Lewis base activation, which would reverse the synergistic cycloaddition to a cascade vinylogous Michael addition and diastereodivergent allylic amination process with 1-azadiene 2a, as proposed in Scheme 1c. In the current work, we carried out a detailed study of the diastereodivergent and asymmetric [4 + 2] annulations between cyclic 1,3-dienes and 1-azadienes *via* ligand-controlled Pd catalysis. These annulations furnished both *cis*- and *trans*-fused tetrahydro-pyridine frameworks, structures having potential biological relevance, in a switchable and enantioenriched manner.^13^

## Results and discussion

### Optimisation of conditions for the diastereodivergent [4 + 2] annulation reaction

As discussed above, using Pd(PPh_3_)_4_ successfully changed the reaction pathway of the reaction of 1,3-cyclohexadiene 1a with 1-azadiene 2a from a concerted Diels–Alder process into a stepwise tandem vinylogous Michael addition/allylic amination sequence, making the potential diastereodivergent construction of both *cis*- and *trans*-fused cycloadducts possible when properly tuning the catalytic conditions. A series of chiral ligands in combination with Pd_2_(dba)_3_ were investigated in order to realise the diastereo-divergent [4 + 2] annulations asymmetrically. While commonly used *S*-BINAP L1 and Trost's ligand L2 failed to promote the conversions at 60 °C (Table 1, entries 1 and 2), using TADDOL-derived phosphoramidite ligand L3 pleasingly provided chiral *exo*-4a in moderate yield and enantioselectivity with complete diastereocontrol (entry 3). Other types of phosphoramidite ligands, namely L4–L7, derived from different chiral backbones were also screened (entries 4–7), and 4a was finally obtained as a single diastereomer in outstanding yield and enantioselectivity by using a SPINOL-based ligand L7 (entry 7).^14^ High catalytic efficiency was still observed with lower ligand loadings (entry 8), but the yield was decreased significantly with 5 mol% palladium (entry 9). In order to switch the diastereoselectivity, more chiral ligands were investigated. While Tang's chiral P-based ligand L8 and 1,2-aminoalcohol-derived ligand L9 delivered the products as diastereomeric mixtures (entries 10 and 11),^15,16^ employing commercially available (*R*,*R*)-Me-DuPhos monoxide L10 led to exclusive formation of *trans*-5a in an excellent yield with good enantiocontrol (entry 12). In order to further improve the enantioselectivity for 5a, more reaction parameters involving ligands, solvents, additives and temperature were evaluated, but inferior results were generally observed (entries 13–21).^17,18^

**Table 1:** Optimisation of catalytic conditions for diastereodivergent [4 + 2] annulations^aa^

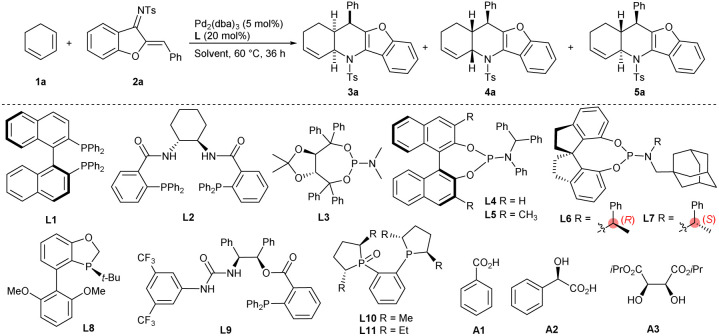						
Entry	L	Solvent	Additive	Yield^b^ (%)	dr^c^	ee^d^ (%)
1	L1	Toluene	—	Trace	—	—
2	L2	Toluene	—	Trace	—	—
3	L3	Toluene	—	4a, 62	>19 : 1	75
4	L4	Toluene	—	4a, 82	>19 : 1	93
5	L5	Toluene	—	4a, 30	4 : 1	71
6	L6	Toluene	—	4a, 73	10 : 1	93
7	L7	Toluene	—	4a, 95	>19 : 1	98
8^e^	L7	Toluene	—	4a, 95	>19 : 1	98
9^f^	L7	Toluene	—	4a, 50	>19 : 1	98
10	L8	Toluene	—	Messy	—	—
11	L9	Toluene	—	Messy	—	—
12	L10	Toluene	—	5a, 92	<1 : 19	82
13	L11	Toluene	—	Trace	—	—
14	L10	THF	—	5a, 51	1 : 10	80
15	L10	Dioxane	—	5a, 35	1 : 11	82
16	L10	CHCl_3_	—	5a, 20	1 : 2	89
17	L10	Toluene	A1	5a, 33	<1 : 19	83
18	L10	Toluene	A2	5a, 76	<1 : 19	80
19	L10	Toluene	A3	5a, 75	<1 : 19	80
20^g^	L10	Toluene	—	5a, 25	<1 : 19	82
21^h^	L10	Toluene	—	5a, 56	<1 : 19	83

a Unless noted otherwise, reactions were carried out with 1a (0.1 mmol), 2a (0.05 mmol), Pd_2_(dba)_3_ (5 mol%), L (20 mol%) in toluene (0.5 mL) at 60 °C for 36 h under Ar.

b Yield of the isolated product.

c The ratio of 4a/5a, determined from ^1^H NMR analysis of crude products.

d Determined from HPLC analysis on a chiral stationary phase.

e L7 (10 mol%).

f With Pd_2_(dba)_3_ (2.5 mol%).

g With L10 (10 mol%).

h At 50 °C, for 72 h.

### Substrate scope and limitations

**Scheme 2:**
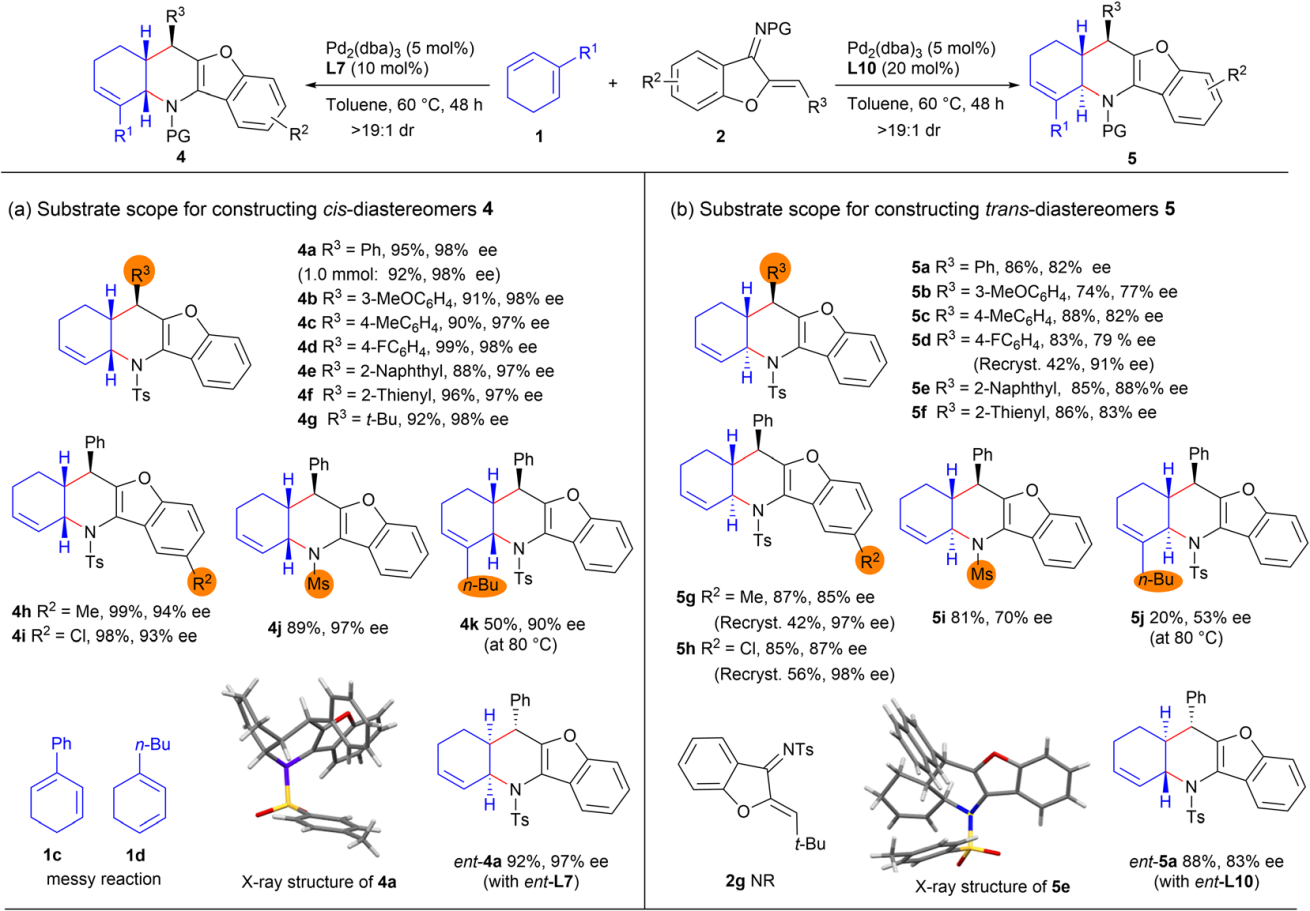
Substrate scope and limitations of diastereodivergent [4 + 2] annulations. Unless noted otherwise, reactions were carried out with 1 (0.2 mmol, 2.0 equiv.), 1-azadiene 2 (0.1 mmol, 1.0 equiv.), Pd_2_(dba)_3_ (5 mol%), and L7 (10 mol%) or L10 (20 mol%) in toluene (1.0 mL) at 60 °C for 48 h under Ar.

With the optimised catalytic conditions in hand, we first investigated the substrate scope and limitations for asymmetric synthesis of *cis*-fused diastereomers 4 under the catalysis of Pd_2_(dba)_3_ and ligand L7. As summarised in Scheme 2a, an array of *N*-tosyl 1-azadienes 2 bearing diverse aryl, heteroaryl, and even *tert*-butyl groups underwent the [4 + 2] annulations with 1,3-cyclohexadiene 1a smoothly, affording the expected products 4b–4g with excellent yields and stereoselectivity, even on a 1.0 mmol scale (for product 4a). In addition, varying the substituents on the benzofuran ring and *N*-protecting group had minimal effect on the reactivity and stereoselectivity, as comparably good results were attained for products 4h–4j. Moreover, diene partner 1b bearing a 2-*n*-butyl group was also applicable, having reacted to form enantioenriched product 4k in a moderate yield, whereas complex reaction profiles were observed with 2-phenyl-1,3-cyclohexadiene 1c and 1-*n*-butyl-1,3-cyclohexadiene 1d.

The substrate scope for the synthesis of *tran*s-fused diastereomers 5 was investigated next. As outlined in Scheme 2b, this synthesis was observed to be relatively inefficient when Pd_2_(dba)_3_ in combination with L10 was used as the catalyst. The *trans*-fused products 5b–5i were generally obtained in good yields and enantioselectivity but with exclusive diastereoselectivity, whereas a significantly decreased yield and enantiocontrol were observed for alkyl-substituted product 5j. And the alkyl-substituted 1-azadiene 2g was not reactive. It should be noted that the optical purity levels of some products, such as 5d, 5g and 5h, could be readily improved by subjecting them to simple recrystallisation (Scheme 2b, data in parentheses). Moreover, *ent*-4a and *ent*-5a could be effectively furnished by using the combination of Pd_2_(dba)_3_ with complementary ligands *ent*-L7 and *ent*-L10, respectively; thus four diastereomers could be smoothly produced just by tuning the ligands, demonstrating the versatility of the current method.

**Scheme 3:**
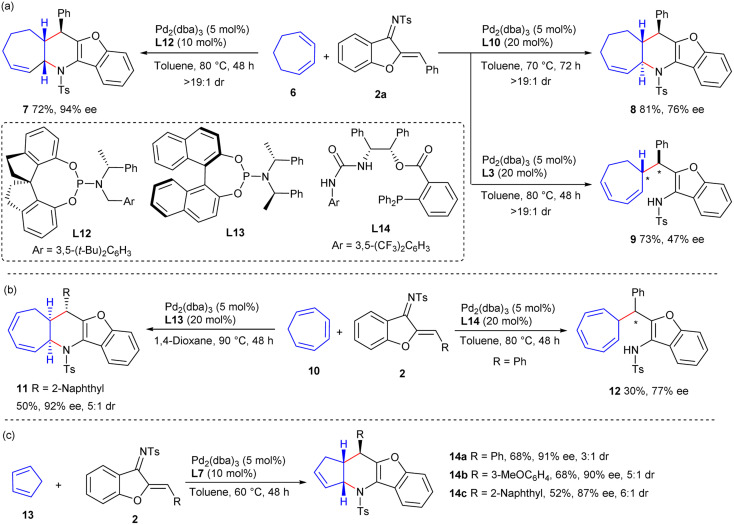
Investigation of more cyclic dienes or polyenes. (a) Reactions of cycloheptadiene 6. (b) Reactions of cycloheptatriene 10. (c) Reactions of cyclopentadiene 13.

Additional types of cyclic dienes and even polyenes were investigated. As illustrated in Scheme 3a, 1,3-cycloheptadiene 6 could be successfully applied to diastereodivergent [4 + 2] annulations with 1-azadiene 2a to produce *cis*-product 7 and *trans*-product 8 in moderate to good yields and stereoselectivity, by employing ligands L12 and L10, respectively. Interestingly, a β-H elimination, rather than *N*-allylic alkylation, occurred to give adduct 9 in a moderate yield with fair enantioselectivity, when using TADDOL-derived phosphoramidite ligand L3; this result provided further support for the involvement of a stepwise process in the observed [4 + 2] reaction. Additionally, *cis*-fused cycloadduct 11 was obtained from cycloheptatriene 10 and 1-azadiene 2 with excellent enantioselectivity and moderate diastereoselectivity when using Pd/L13 as the catalyst, whereas β-H elimination product 12 was delivered with moderate results when using Pd/L14 (Scheme 3b). Furthermore, the assemblies of cyclopentadiene 13 and 1-azadienes 2 occurred successfully when Pd/L7 was used as the catalyst, and *exo*-selective cycloadducts 14a–14c were furnished with high enantioselectivity and moderate diastereoselectivity (Scheme 3c).

**Scheme 4:**
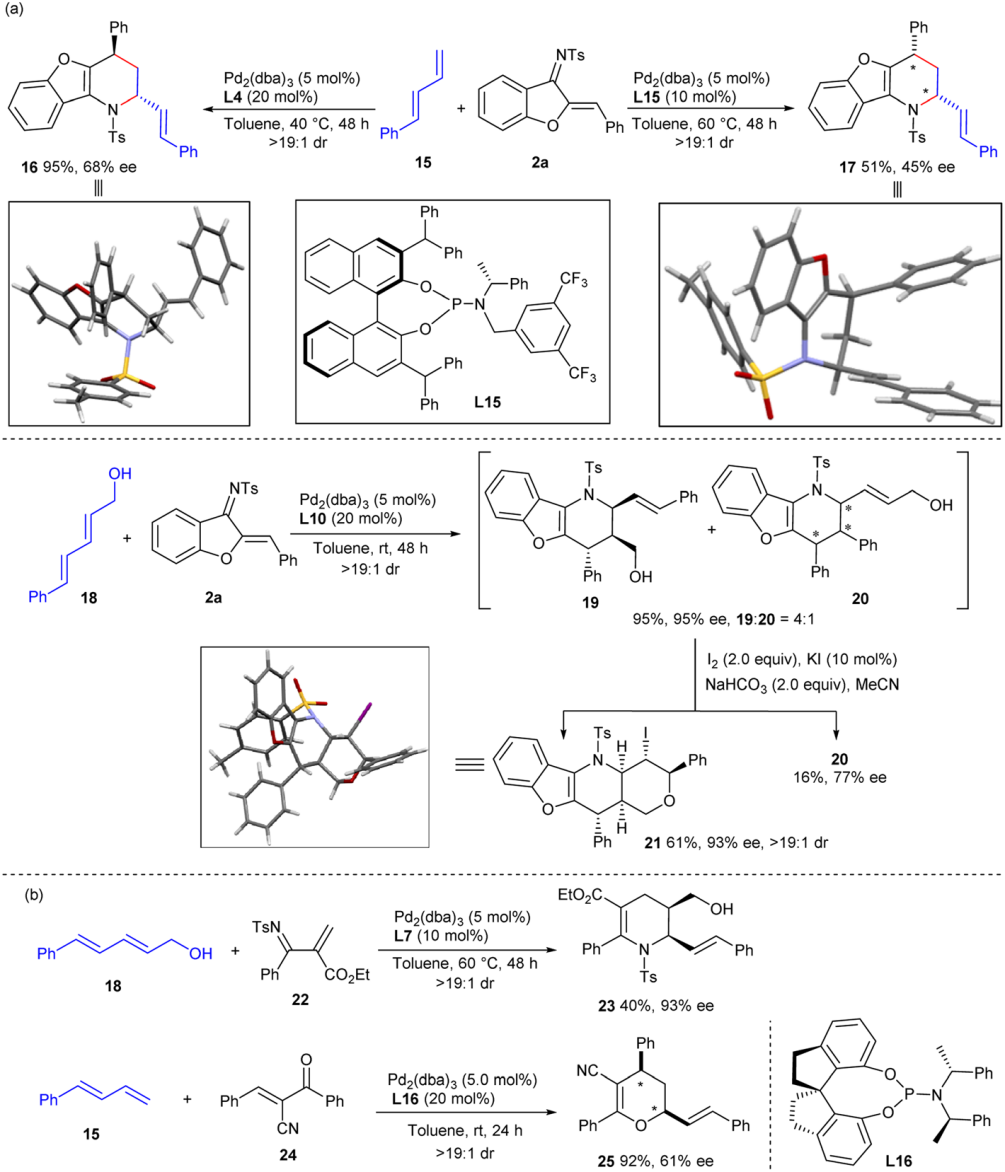
Investigation of linear 1,3-dienes and 1-heterodienes. (a) Assembly of linear 1,3-diene and 1-azadiene 2a. (b) Investigation of linear electron-deficient heterodienes.

The current catalytic strategy could be further expanded to acyclic dienes, further enriching the structural diversity of the frameworks constructed. As depicted in Scheme 4a, diastereodivergent [4 + 2] annulations between linear terminal 1,3-diene 15 and 1-azadiene 2a could be similarly realised *via* ligand-controlled palladium catalysis. Both *exo*- and *endo*-selective cycloadducts 16 and 17 were efficiently constructed when using ligands L4 and L15, respectively, albeit with moderate enantioselectivity. Internal diene 18 exhibited higher reactivity with 1-azadiene 2a when using Pd/L10 as the catalyst, providing the *exo*-cycloadduct 19 in excellent yield and stereoselectivity, along with an inseparable regioisomer 20. Nevertheless, a chemoselective intramolecular iodoetherification of 19 was further carried out to generate tetracyclic product 21 with exclusive diastereocontrol. Apart from cyclic 1-azadienes 2, linear 2-*N*-tosyliminoacrylate 22 and 1-oxadiene 24 were also reliable partners in the assemblies with linear 1,3-dienes,^11*e*^ furnishing tetrahydropyridine product 23 and dihydropyran 25, respectively, with moderate results. These experiments well verified the general compatibility of the π-Lewis base catalysis of Pd^0^ complexes for activating diene substrates, though some improvements remain to be made.

## Conclusions

Taking advantage of the unique vinylogous activation feature of Pd^0^-π-Lewis base catalysis, we successfully reversed the process of the concerted and *endo*-selective hetero-Diels–Alder cycloaddition reaction between cyclic 1,3-diene and electron-deficient aurone-derived cyclic 1-azadienes into a stepwise cascade vinylogous Michael addition/allylic amination sequence. As a result, the formal *exo*-selective and *cis*-fused [4 + 2] annulation products with polycyclic architectures were efficiently constructed with moderate to excellent enantioselectivity and diastereoselectivity levels by using a chiral SPINOL-derived phosphoramidite ligand. Moreover, the switchable diastereodivergent synthesis of challenging *trans*-fused [4 + 2] frameworks was also successfully accomplished by employing commercially available (*R*,*R*)-Me-DuPhos monoxide ligand. In addition, the current Pd^0^-π-Lewis base catalysis was applicable to the assemblies for a variety of acyclic 1,3-dienes and 1-heterodienes, further enriching the structural diversity and versatility of relevant cycloadducts.

## Data availability

The data that support the findings of this study are available in the ESI† or on request from the corresponding author.

## Author contributions

All authors contributed to the writing of the manuscript and have given approval to the final version of the manuscript.

## Conflicts of interest

There are no conflicts to declare.

## Supplementary Material

SC-014-D2SC06813C-s001

SC-014-D2SC06813C-s002

## References

[cit1] Brooks W. H., Guida W. C., Daniel K. G. (2011). Curr. Top. Med. Chem..

[cit2] Lin L., Feng X. (2017). Chem.–Eur. J..

[cit3] Feng X., Zhou Z., Zhou R., Zhou Q.-Q., Dong L., Chen Y.-C. (2012). J. Am. Chem. Soc..

[cit4] Trost B. M., Cramer N., Silverman S. M. (2007). J. Am. Chem. Soc..

[cit5] Jensen K. L., Weise C. F., Dickmeiss G., Morana F., Davis R. L., Jørgensen K. A. (2012). Chem. - Eur. J..

[cit6] Tian X., Cassani C., Liu Y., Moran A., Urakawa A., Galzerano P., Arce E., Melchiorre P. (2011). J. Am. Chem. Soc..

[cit7] Shi S.-L., Wong Z. L., Buchwald S. L. (2016). Nature.

[cit8] Gilmour R., Prior T. J., Burton J. W., Holmes A. B. (2007). Chem. Commun..

[cit9] Zhu Y., Chen X., Xie M., Dong S., Qiao Z., Lin L., Liu X., Feng X. (2010). Chem. - Eur. J..

[cit10] Deng Q., Meng X. (2020). Chem.–Asian J..

[cit11] Xiao B.-X., Jiang B., Yan R.-J., Zhu J.-X., Xie K., Gao X.-Y., Ouyang Q., Du W., Chen Y.-C. (2021). J. Am. Chem. Soc..

[cit12] Hatano M., Mizuno T., Izumiseki A., Usami R., Asai T., Akakura M., Ishihara K. (2011). Angew. Chem., Int. Ed..

[cit13] Scott J. D., Williams R. M. (2002). Chem. Rev..

[cit14] Xie J.-H., Zhou Q.-L. (2008). Acc. Chem. Res..

[cit15] Xu G., Senanayake C. H., Tang W. (2019). Acc. Chem. Res..

[cit16] Chen C., Yang X.-X., Zhao Z., Han B., Du W., Chen Y.-C. (2022). Chem. Commun..

[cit17] For more condition screenings, see the ESI†

[cit18] For more control experiments and proposed ligand-based stereocontrol models, see the ESI†

