# Supplementary material for: Diastereodivergent cis- and trans-fused [4 + 2] annulations of cyclic 1,3-dienes and 1-azadienes via ligand-controlled palladium catalysis
Source: Chem Sci. 2023 Jan 17;14(7):1896–901. doi: 10.1039/d2sc06813c (PMC9931049; doi:10.1039/d2sc06813c)
Supplement: SC-014-D2SC06813C-s001 [file SC-014-D2SC06813C-s001.pdf]

Electronic Supplementary Material (ESI) for Chemical Science.
This journal is © The Royal Society of Chemistry 2023

Diastereodivergent *cis*- and *trans*-fused [4+2] annulations of cyclic 1,3-dienes and 1-azadienes via ligand-controlled palladium catalysis

Yuan Hu,[a] Jin-Yu Huang,[a] Ru-Jie Yan,[a] Zhi-Chao Chen,*[a] Qin Ouyang,[b] Wei Du,[a] and Ying-Chun Chen*[ab]

[a] Key Laboratory of Drug-Targeting and Drug Delivery System of the Education Ministry and Sichuan Province, and Sichuan Research Center for Drug Precision Industrial Technology, West China School of Pharmacy, Sichuan University, Chengdu 610041, China.

[b] College of Pharmacy, Third Military Medical University, Shapingba, Chongqing 400038, China.

*Corresponding Authors: chenzhichao@scu.edu.cn; ycchen@scu.edu.cn

Supplementary Information

1. General methods and materials **S1**

2. Preparation and characterization of ligand L15 .... **S2**

3. Detailed screening conditions **S3**

3.1 Preliminary ligand screenings for diastereodivergent [4+2] annulation S3

3.2 Detailed screening conditions for asymmetric synthesis of **4a** S4

3.3 Detailed screening conditions for asymmetric synthesis of **5a** S4

3.4 Detailed screening conditions for asymmetric synthesis of **3a** S6

4.General procedure for ligand-controlled diastereodivergent synthesis **S6**

4.1 Procedure for synthesis of **3a** S6

4.2 General procedure for asymmetric synthesis of *exo*-**4** S7

4.3 General procedure for asymmetric synthesis of diastereomers **5** S15

5. More substrate exploration **S22**

5.1 Exploration of more cyclic dienes and polyenes S22

5.2 Exploration of linear 1,3-dienes S28

5.3 Exploration of linear electron-deficient heterodienes S31

5.4 Unsuccessful substrates attempts S33

6. Mechanism investigation **S34**

6.1 Control experiments S34

6.2 Proposed origin of the divergent diasteroselectivity S35

6.3 DFT calculations for the reaction of 1-azadiene **2a** and 1,3-butadiene **15** S36

7. Crystal data and structural refinement **S37**

8. NMR, HRMS spectra and HPLC chromatograms **S49**

9. Computational method **S161**

1. General methods and materials

Unless otherwise noted, the reactions were carried out under ambient atmosphere; when the reactions required heating, the heat source was oil bath. ^{1}H NMR (400 or 600 MHz), ^{13}C NMR (100 or 150 MHz), ^{31}P NMR (162 MHz) and ^{19}F NMR (375 MHz) spectra were recorded on Varian INOVA-400/54, Agilent DD2-600/54 or Bruker Ascend^{TM} 400 instruments (Chemical shifts were reported in ppm from tetramethylsilane with the solvent resonance as the internal standard in $CDCl_3$ solution, unless otherwise noted). The following abbreviations were used to explain the multiplicities: s = singlet, d = doublet, t = triplet, q = quartet, dd = double doublet, ddd = doublet of doublet of doublets, dt = doublet of triplets, m = multiplet, and coupling constants (*J*) are reported in Hertz (Hz). High resolution mass spectra (HRMS) were recorded on a Waters SYNAPT G2, Agilent G1969-85000 or Shimadzu LCMS-IT-TOF using a time-of-flight mass spectrometer equipped with electrospray ionization (ESI) source. X-ray diffraction experiments were carried out on an Agilent Xcalibur or Bruker APEX-II CCD diffractometer, and the data obtained were deposited at the Cambridge Crystallographic Data Centre (CCDC 2050823, 2050825–2050826 and 2219802–2219806). In each case, enantiomeric excess was determined by HPLC (Agilent Technologies: 1220 Infinity II, 1200 Series, 1260 Infinity) analysis on a chiral column in comparison with authentic racemate, using a Daicel Chiralpak AD-H Column (250 × 4.6 mm), Chiralpak IB Column (250 × 4.6 mm), Chiralpak IC Column (250 × 4.6 mm), Chiralpak ID Column (250 × 4.6 mm), Chiralpak IE Column (250 × 4.6 mm), Chiralpak IF Column (250 × 4.6 mm), Chiralpak IG Column (250 × 4.6 mm), Chiralpak IH Column (250 × 4.6 mm). UV detection was monitored at 254 nm. The specific optical rotation was obtained from Rudolph Research Analytical Autopol I automatic polarimeter in $CHCl_3$ solution at 25 °C. The melting point was obtained from WRX-4 Mel-Temp apparatus. Column chromatography was performed on silica gel (300–400 mesh) eluting with ethyl acetate (EtOAc)/petroleum ether or dichloromethane (DCM)/petroleum ether. TLC was performed on glass-backed silica plates. UV light, I_2, and solution of potassium permanganate were used to visualize products or starting materials. Experiments involving moisture and/or air sensitive components were performed under a positive pressure of argon in oven-dried glassware equipped with a rubber septum inlet. Toluene was freshly distilled from CaH_2 under an atmosphere of dry argon. Dried solvents and liquid reagents were transferred via oven-dried syringe. Petroleum ether and EtOAc were distilled. 1,3-Cyclohexadiene **1a**, cycloheptadiene **6** and cycloheptatriene **10** were used without purification as commercially available. Dicyclopentadiene was cracked at 170 °C and re-

distilled to give 1,3-cyclopentadiene **13**. The ligand **L7**,[1] **L12**,[1] **1b**,[2] 1-azadiene **2**,[3] 1,3-diene **15**,[4] internal diene **18**,[5] 2-*N*-tosyliminoacrylate **22**[6] and 1-oxadiene **24**[7] were prepared according to the literature procedures.

(1) X. Song, J. Zhang, Y. X. Wu, Q. Ouyang, W. Du and Y.-C. Chen, *J. Am. Chem. Soc.*, 2022, **144**, 9564−9569.

(2) H. E. Burks, L. T. Kliman and J. P. Morken, *J. Am. Chem. Soc.*, 2009, **131**, 9134–9135.

(3) Z.-Q. Rong, M. Wang, C. H. E. Chow and Y. Zhao, *Chem. Eur. J.*, 2016, **22**, 9483−9487.

(4) A. Lishchynskyi and K. Muñiz, *Chem. Eur. J.*, 2022, **18**, 2212–2216.

(5) F. Carlet, G. Bertarini, G. Broggini and G. Poli, *Eur. J. Org. Chem.*, 2021, 2162–2168.

(6) H. Liu, Q. Zhang, L. Wang and X. Tong, *Chem. Eur. J.*, 2010, **16**, 1968–1972.

(7) A. P. Dieskau, M. S. Holzwarth and B. Plietker, *J. Am. Chem. Soc.*, 2012, **134**, 5048–5051.

2. Preparation and characterization of ligand L15

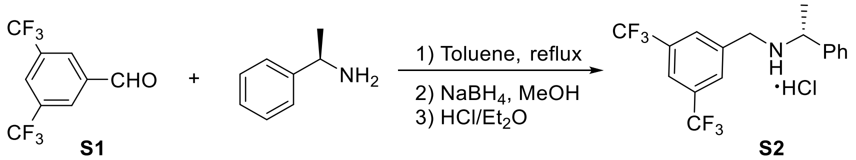

The mixture of **S1** (2.42 g, 10.0 mmol) and (*R*)-1-phenylethylamine (1.21 g, 10.0 mmol) in toluene (12 mL) was stirred at 110 °C for 2 h. After cooled to room temperature, anhydrous $MgSO_4$ (30.0 g) was added to the mixture and stirred for 30 min, followed by filtration and concentration. The residue was dissolved in methanol (20.0 mL), and $NaBH_4$ (378.0 mg, 10.00 mmol) was added in three portions at 0 °C. After 30 min, the mixture was quenched with water (20.0 mL), and extracted with EtOAc (20 mL × 3). The combined organic layers were washed with brine, dried over anhydrous Na_2SO_4, filtered and concentrated in vacuo. Then concentrated HCl (aq) (2.0 mL) was added dropwise to the solution of the crude secondary amine in diethyl ether (10.0 mL). The white precipitates were filtered and washed with EtOAc to afford **S2** (3.0 g, 86% yield).

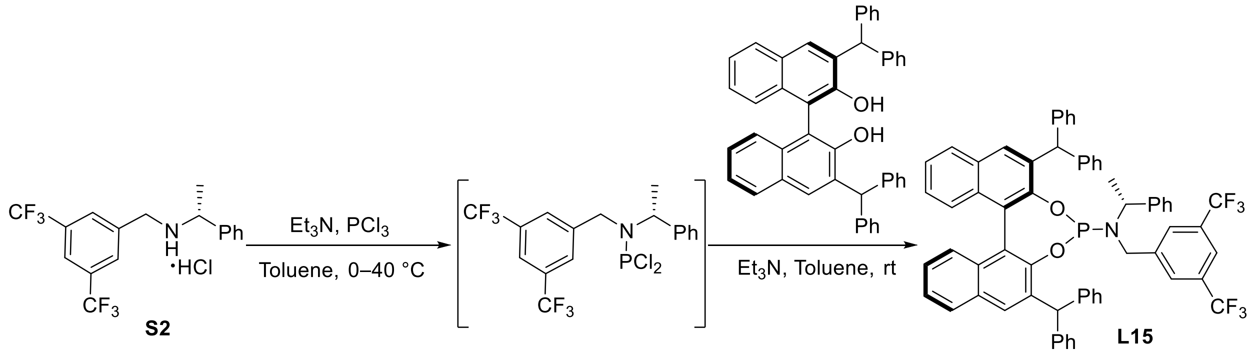

Triethylamine (0.1 mL, 1.2 mmol, 6.0 equiv) was added dropwise to a stirred ice-cooled solution

of PCl_3 (0.11 mL, 1.2 mmol, 6.0 equiv) in dry toluene (1.0 mL) under argon atmosphere. The resultant mixture was gradually warmed to 40 °C before **S2** (154.0 mg, 0.4000 mmol, 2.0 equiv) was added. After stirred for additional 4 h, the mixture was concentrated in vacuo. The residue was dissolved in dry toluene, and (*R*)-3,3'-dibenzhydryl-[1,1'-binaphthalene]-2,2'-diol (124.0 mg, 0.2000 mmol) and triethylamine (0.10 mL, 1.2 mmol, 6.0 equiv) were added sequentially under argon atmosphere. Then the mixture was stirred at room temperature for 12 h. The mixture was concentrated, and the residue was purified by flash chromatography on silica gel (EtOAc/petroleum ether = 1/100) to give ligand **L15**: 94 mg (0.095 mmol), as a white solid, 47% yield; mp = 141–143 °C; $[\alpha]_D^{25}$ = –139.0 (*c* = 0.41 in $CHCl_3$); ^1H NMR (400 MHz, $CDCl_3$) δ (ppm) 7.71 (d, *J* = 8.2 Hz, 1H), 7.66 (d, *J* = 8.2 Hz, 1H), 7.60 (s, 1H), 7.54 (s, 2H), 7.45 (d, *J* = 5.4 Hz, 2H), 7.41–6.96 (m, 29H), 6.90–6.77 (m, 2H), 6.29 (s, 1H), 5.80 (s, 1H), 4.18 (d, *J* = 15.7 Hz, 1H), 4.09–3.88 (m, 1H), 3.47 (d, *J* = 15.6 Hz, 1H), 1.35 (dd, *J* = 7.3, 2.5 Hz, 3H); ^{13}C NMR (150 MHz, $CDCl_3$) δ (ppm) 148.71, 148.67, 147.7, 143.9, 143.7, 142.9, 142.6, 142.1, 142.0, 141.6, 135.8, 135.1, 132.4, 132.0, 131.8, 131.4, 131.2, 130.9 (d, $^2J_{FC}$ = 33.0 Hz), 130.8, 130.5, 130.1, 129.89, 129.86, 128.8, 128.7, 128.6, 128.4, 128.31, 128.28, 128.12, 128.07, 128.0, 127.5, 127.4, 127.0, 126.9, 126.7, 126.49, 126.45, 126.3, 125.8, 125.7, 124.9, 124.6, 124.3, 123.4 (d, $^1J_{FC}$ = 272.2 Hz), 122.13, 122.10, 120.6 (m), 57.5 (d, *J* = 34.7 Hz), 51.0, 50.3, 47.3 (d, *J* = 4.6 Hz), 21.7 (d, *J* = 24.3 Hz); ^{19}F NMR (375 MHz, $CDCl_3$) δ (ppm) –62.6; ^{31}P NMR (162 MHz, $CDCl_3$): δ (ppm) 139.4 (d, *J* = 12.0 Hz); HRMS (ESI-TOF) m/z: $[M + H]^+$ Calcd for $C_{63}H_{47}F_6NO_2P^+$ 994.3243; Found 994.3246.

3. Detailed screening conditions

3.1 Preliminary ligand screenings for diastereodivergent [4+2] annulation

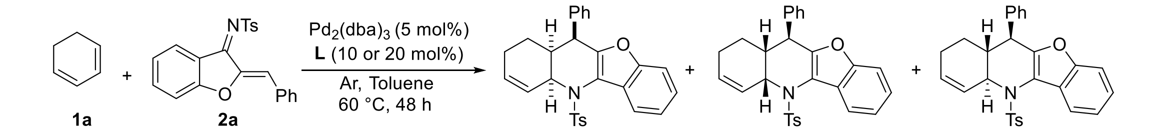

1a + **2a** → **3a** + **4a** + **5a**

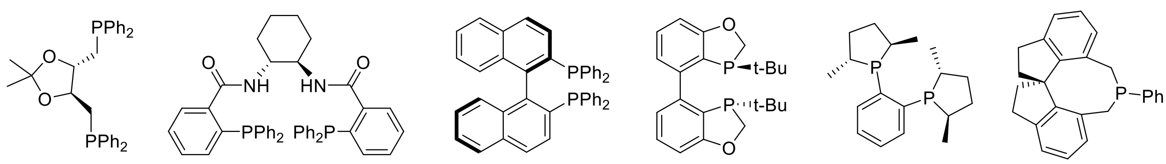

30 % Conv. | trace | bad conv. | Trace | Trace | 30% Conv.

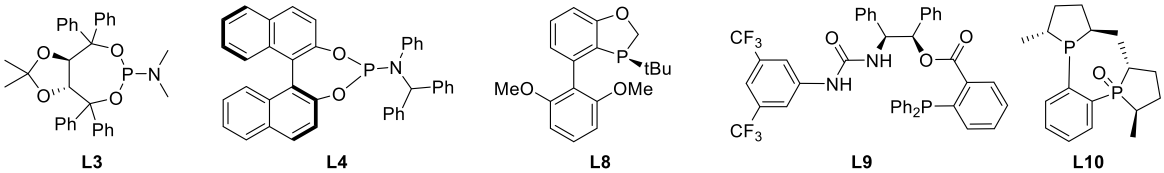

L3
4a: 62%, 75% ee
>19:1 dr

L4
4a: 82%, 92% ee
>19:1 dr

L8
35%, 90% ee
4a:(**3a**+**5a**) = 1:1

L9
53%, 87% ee
3a:(**4a**+**5a**) = 1:1

L10
5a: 92%, 82% ee
>19:1 dr

A series of chiral ligands derived from different backbones were investigated. As outlined above, phosphoramidite ligands **L3** and **L4** led to the exclusive formation of **4a**, while 1,2-aminoalcohol-drived **L9** and (*R,R*)-Me-DuPhos monoxide **L10** were favourable to produce **3a** and **5a**, respectively. More relevant ligands and other parameters were screened subsequently in order to realise the diastereodivergent synthesis.

3.2 Detailed screening conditions for asymmetric synthesis of 4a

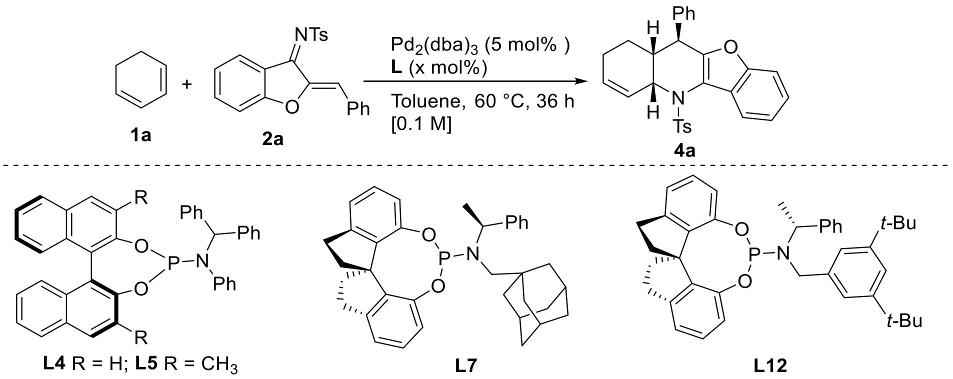

Entry	L	x	Yield (%)[b]	**4a**:(**3a**+**5a**)[c]	ee (%)[d]
1	**L4**	20	82	>19:1	93
2	**L5**	20	30	4:1	71
3	**L7**	20	95	>19:1	98
4	**L7**	10	95	>19:1	98
5[e]	**L7**	10	50	>19:1	98
6	**L12**	10	60	>19:1	91

[a] Unless noted otherwise, reactions were performed with **1a** (0.1 mmol), **2a** (0.05 mmol), $Pd_2(dba)_3$ (5 mol%), **L** (x mol%) in dry toluene (0.5 mL) at 60 °C for 36 h under Ar. [b] Yield of isolated product. [c] Determined by ^1H NMR analysis. [d] The data referred to ee of **4a**, determined by HPLC analysis on a chiral stationary phase. [e] $Pd_2(dba)_3$ (2.5 mol%).

3.3 Detailed screening conditions for asymmetric synthesis of 5a

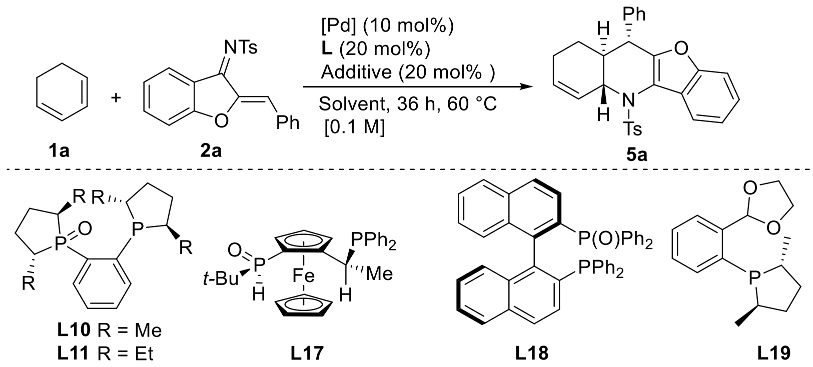

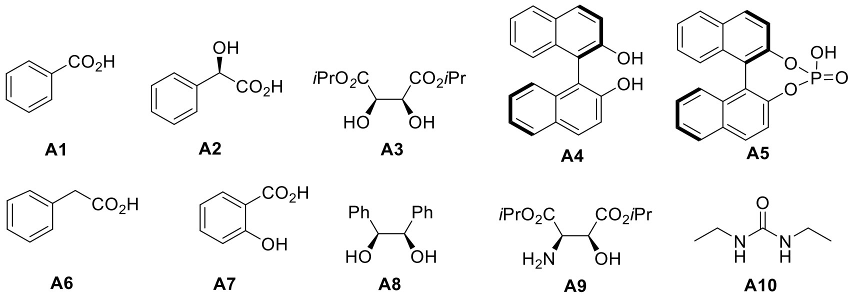

Entry	[Pd]	**L**	Solvent	Additive	Yield (%)[b]	**5a**:(**3a**+**4a**)[c]	ee (%)[d]
1	$Pd_2(dba)_3$	**L10**	Toluene	/	92	>19:1	82
2	$Pd_2(dba)_3$	**L11**	Toluene	/	Trace	/	/
3	$Pd_2(dba)_3$	**L17**	Toluene	/	Trace	/	/
4	$Pd_2(dba)_3$	**L18**	Toluene	/	Trace	/	/
5	$Pd_2(dba)_3$	**L19**	Toluene	/	Trace	/	/
6	$Pd_2(dba)_3$	**L10**	THF	/	51	10:1	80
7	$Pd_2(dba)_3$	**L10**	2-Me-THF	/	61	>19:1	80
8	$Pd_2(dba)_3$	**L10**	$PhCF_3$	/	10	7:1	77
9	$Pd_2(dba)_3$	**L10**	Dioxane	/	35	11:1	82
10	$Pd_2(dba)_3$	**L10**	CH_2Cl_2	/	26	7:3	83
11	$Pd_2(dba)_3$	**L10**	$CHCl_3$	/	20	1:2	89
12	$Pd_2(dba)_3$	**L10**	Xylene	/	Trace	/	/
13	$Pd_2(dba)_3$	**L10**	EtOAc	/	Trace	/	/
14	$Pd_2(dba)_3$	**L10**	Anisole	/	Trace	/	/
15	$Pd_2(dba)_3$	**L10**	Toluene	**A1**	33	>19:1	83
16	$Pd_2(dba)_3$	**L10**	Toluene	**A2**	76	>19:1	80
17	$Pd_2(dba)_3$	**L10**	Toluene	**A3**	75	>19:1	80
18	$Pd_2(dba)_3$	**L10**	Toluene	**A4**	58	>19:1	80
19	$Pd_2(dba)_3$	**L10**	Toluene	**A5**	Trace	/	/
20	$Pd_2(dba)_3$	**L10**	Toluene	**A6**	Trace	/	/
21	$Pd_2(dba)_3$	**L10**	Toluene	**A7**	Trace	/	/
22	$Pd_2(dba)_3$	**L10**	Toluene	**A8**	73	>19:1	80
23	$Pd_2(dba)_3$	**L10**	Toluene	**A9**	70	>19:1	80
24	$Pd_2(dba)_3$	**L10**	Toluene	**A10**	76	>19:1	80
25	$[Pd(allyl)Cl]_2$	**L10**	Toluene	/	90	>19:1	80
26	$Pd(OAc)_2$	**L10**	Toluene	/	39	>19:1	80
27	$Pd(CH_3CN)_2Cl_2$	**L10**	Toluene	/	NR	/	/
28[e]	$Pd_2(dba)_3$	**L10**	Toluene	/	56	>19:1	82
29[f]	$Pd_2(dba)_3$	**L10**	Toluene	/	25	>19:1	82

^{a} Unless noted otherwise, reactions were performed with **1a** (0.1 mmol), **2a** (0.05 mmol), [Pd] source (10 mol%), **L** (20 mol%), acid additive (20 mol%) in dry toluene (0.5 mL) at 60 °C for 36 h under Ar. ^{b} Yield of isolated product. ^{c} Determined by HPLC analysis. ^{d} The data referred to ee of **5a**, determined by HPLC analysis on a chiral stationary phase. ^{e} At 50 ℃. ^{f} With **L10** (10 mol%).

3.4 Detailed screening conditions for asymmetric synthesis of 3a

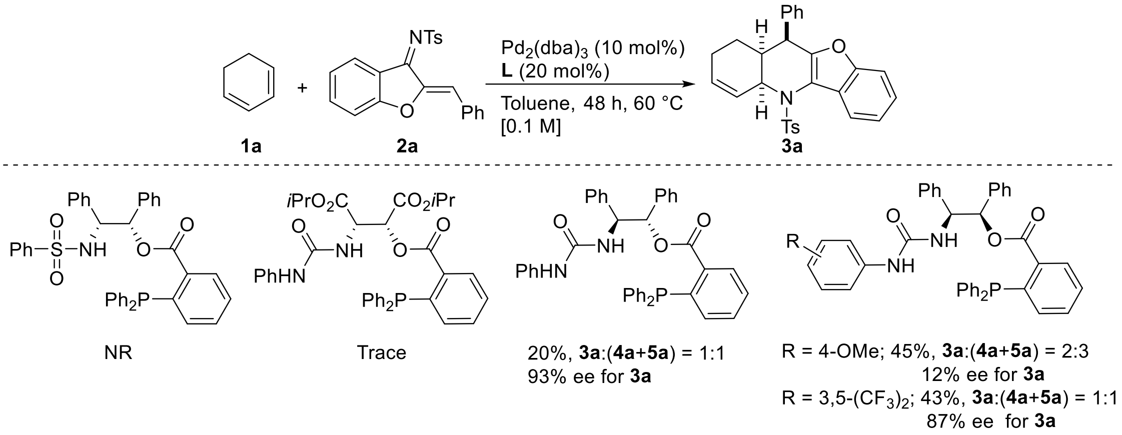

As the reaction of **1a** with **2a** could not proceeded without palladium at 60 ℃, the potential asymmetric synthesis of **3a** also might be achieved. Although a series of 1,2-aminoalcohol-drived bifunctional ligands were screened, bad diastereoselectivity was generally observed. As a result, currently successful construction of chiral *endo*-**3a** has not been realised yet.

4.General procedure for ligand-controlled diastereodivergent synthesis

4.1 Procedure for synthesis of 3a

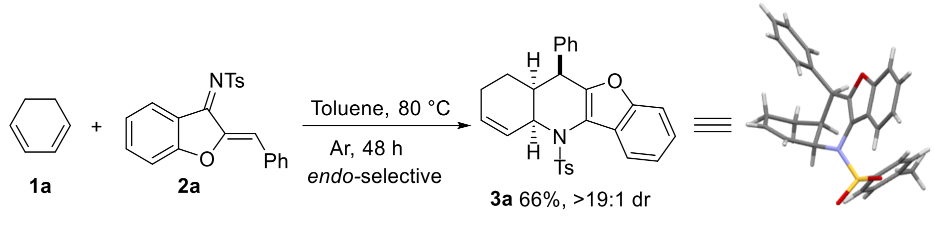

To an oven dried 10 mL Schlenk tube equipped with a stirring bar was added *N*-((*E*)-2-((*Z*)-benzylidene)benzofuran-3(2*H*)-ylidene)-4-methylbenzenesulfonamide **2a** (37.5 mg, 0.100 mmol, 1.0 equiv). The tube was capped, evacuated and back-filled with argon for five times. Then degassed dry toluene (0.5 mL) and 1,3-cyclohexadiene **1a** (19.0 μL, 0.200 mmol, 2.0 equiv) were added via syringe sequentially under argon atmosphere. The mixture was stirred at 80 °C for 48 h. After complete consumption of **2a**, the mixture was directly purified by flash chromatography on silica gel

(EtOAc/petroleum ether = 1/50) to give product **3a**: 30.0 mg (0.0658 mmol), as a white solid, 66% yield; >19:1 dr; mp: 192–194 °C; ^{1}H NMR (400 MHz, $CDCl_3$) δ (ppm) 8.17 (d, *J* = 7.2 Hz, 1H), 7.49 (d, *J* = 8.0 Hz, 2H), 7.39–7.28 (m, 3H), 7.28–7.11 (m,5H), 7.05–6.97 (m, 2H), 5.72 (dd, *J* = 10.2, 2.6 Hz, 1H), 5.39–5.33 (m, 1H), 4.72–4.64 (m, 1H), 3.70 (d, *J* = 7.6 Hz, 1H), 2.42 (s, 3H), 2.08–1.98 (m, 1H), 1.74–1.62 (m, 1H), 1.61–1.43 (m, 2H), 0.62–0.42 (m, 1H); ^{13}C NMR (100 MHz, $CDCl_3$) δ (ppm) 153.9, 148.6, 144.0, 138.0, 134.3, 132.0, 130.3, 129.7, 127.6, 127.5, 127.1, 126.9, 124.7, 124.3, 123.0, 122.4, 117.9, 111.2, 55.9, 41.9, 34.8, 22.0, 21.6, 21.5; HRMS (ESI-TOF) m/z: $[M + Na]^+$ Calcd for $C_{28}H_{25}NO_3SNa^+$ 478.1447; Found 478.1456. *Its relative configuration has been determined by X-ray diffraction analysis.*

4.2 General procedure for asymmetric synthesis of *exo*-4

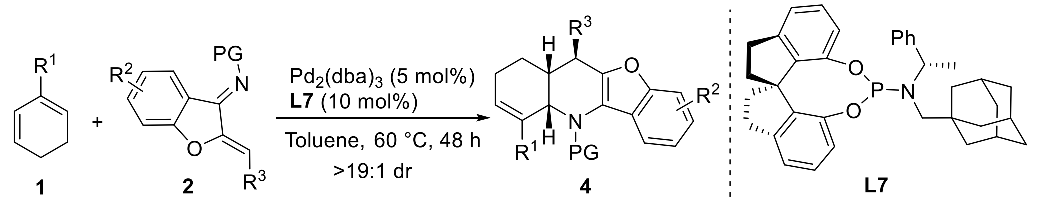

General procdure: To an oven dried 10 mL Schlenk tube equipped with a stirring bar were added 1-azadiene **2** (0.1 mmol, 1.0 equiv), $Pd_2(dba)_3$ (5 mol%) and **L7** (10 mol%). The tube was capped, evacuated and back-filled with argon for five times. Then degassed dry toluene (1.0 mL) and 1,3-cyclohexadiene **1** (0.20 mmol, 2.0 equiv) were added via syringe sequentially under argon atmosphere. The mixture was stirred at 60 °C for 48 h. After complete consumption of **2**, the mixture was directly purified by flash chromatography on silica gel (petroleum ether/EtOAc) to afford product **4**.

*Note: Racemic **4** was obtained by using $Pd_2(dba)_3$ in combination with racemic **L7**. The drs indicated the diastereomeric purity of the isolated products.*

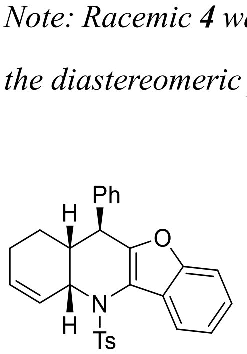

Synthesis of 4a: To an oven dried 10 mL Schlenk tube equipped with a stirring bar were added *N*-((*E*)-2-((*Z*)-benzylidene)benzofuran-3(2*H*)-ylidene)-4-methyl benzenesulfonamide **2a** (37.5 mg, 0.100 mmol, 1.0 equiv), $Pd_2(dba)_3$ (4.6 mg, 0.0050 mmol, 5 mol%) and **L7** (5.5 mg, 0.010 mmol, 10 mol%). The tube was capped, evacuated and back-filled with argon for five times. Then degassed dry toluene (1.0 mL) and 1,3-cyclohexadiene **1a** (19.0 μL, 0.200 mmol, 2.0 equiv) were added via syringe sequentially under argon atmosphere. The mixture was stirred at 60 °C for 48 h. After complete consumption of **2a**, the mixture was directly purified by flash chromatography on silica gel (EtOAc/petroleum ether = 1/50)

to afford product **4a**: 43.1 mg (0.0948 mmol), as a white solid, 95% yield; mp 186–188 ℃; $[\alpha]_D^{25}$ = +248.2 (*c* = 0.34 in $CHCl_3$); >19:1 dr; 98% ee, determined by HPLC analysis (Daicel Chiralpak IG, *n*-Hexane/*i*-PrOH = 90/10, flow rate =1.0 mL/min, λ = 254 nm) t_R = 15.12 min (major), t_R = 19.19 min (minor); ^{1}H NMR (600 MHz, $CDCl_3$) δ (ppm) 8.20 (d, *J* = 7.8 Hz, 1H), 7.58 (d, *J* = 7.5 Hz, 2H), 7.35–7.22 (m, 5H), 7.21–7.19 (m, 1H), 7.17–7.04 (m, 2H), 6.50 (d, *J* = 7.2 Hz, 2H), 5.79–5.67 (m, 1H), 5.57 (d, *J* = 10.2 Hz, 1H), 5.10–4.96 (m, 1H), 3.82 (d, *J* = 9.9 Hz, 1H), 2.43 (s, 3H), 2.27–2.18 (m, 1H), 2.13–2.04 (m, 1H), 1.81–1.74 (m, 1H), 1.71–1.59 (m, 2H); ^{13}C NMR (150 MHz, $CDCl_3$) δ (ppm) 154.2, 150.0, 144.0, 139.7, 134.7, 129.94, 129.87, 128.33, 128.31, 128.1, 128.0, 127.2, 124.7, 124.4, 123.0, 122.4, 116.9, 111.1, 58.3, 39.3, 36.6, 22.1, 21.6, 20.5; HRMS (ESI-TOF) m/z: $[M + H]^+$ Calcd for $C_{28}H_{26}NO_3S^+$ 456.1628; Found 456.1629.

Asymmetric synthesis of 4a on a 1.0 mmol scale: To an oven-dried 100 mL Schlenk tube equipped with a magnetic stirring bar were added *N*-((*E*)-2-((*Z*)-benzylidene)benzofuran-3(2*H*)-ylidene)-4-methylbenzenesulfonamide **2a** (375 mg, 1.00 mmol, 1.0 equiv), $Pd_2(dba)_3$ (46.0 mg, 0.0500 mmol, 5 mol%) and ligand **L7** (55.0 mg, 0.100 mmol, 10 mol%). The tube was capped, evacuated and back-filled with argon for five times. Then degassed dry toluene (10 mL) and 1,3-cyclohexadiene **1a** (191 μL, 0.200 mmol, 2.0 equiv) were added via syringe sequentially under argon atmosphere. The mixture was stirred at 60 °C for 48 h. After complete consumption of **2a**, the mixture was concentrated and the residue was purified by flash chromatography on silica gel (EtOAc/petroleum ether = 1/50) to afford product **4a**: 421.2 mg (0.9245 mmol), as a white solid, 92% yield; >19:1 dr; 98% ee.

Synthesis of *ent*-4a: To an oven dried 10 mL Schlenk tube equipped with a stirring bar were added *N*-((*E*)-2-((*Z*)-benzylidene)benzofuran-3(2*H*)-ylidene)-4-methylbenzenesulfonamide **2a** (37.5 mg, 0.100 mmol, 1.0 equiv), $Pd_2(dba)_3$ (4.6 mg, 0.0050 mmol, 5 mol%) and *ent*-**L7** (5.5 mg, 0.010 mmol, 10 mol%). The tube was capped, evacuated and back-filled with argon for five times. Then degassed dry toluene (1.0 mL) and 1,3-cyclohexadiene **1a** (19.0 μL, 0.200 mmol, 2.0 equiv) were added via syringe sequentially under argon atmosphere. The mixture was stirred at 60 °C for 48 h. After complete consumption of **2a**, the mixture was directly purified by flash chromatography on silica gel (EtOAc/petroleum ether = 1/50) to afford product *ent*-**4a**: 41.8 mg (0.0917 mmol), as a white solid, 92% yield; $[\alpha]_D^{25}$ = –260.0 (*c* = 0.33 in $CHCl_3$); >19:1 dr; 97% ee, determined by HPLC analysis (Daicel Chiralpak IG, *n*-Hexane/*i*-PrOH = 90/10, flow rate =1.0 mL/min, λ = 254 nm) t_R = 14.92 min (minor), t_R = 18.63 min (major).

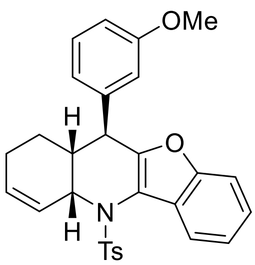

Synthesis of 4b: To an oven dried 10 mL Schlenk tube equipped with a stirring bar were added *N*-((*E*)-2-((*Z*)-3-methoxybenzylidene)benzofuran-3(2*H*)-ylidene)-4-methylbenzenesulfonamide **2b** (40.5 mg, 0.100 mmol, 0.100 mmol, 1.0 equiv), $Pd_2(dba)_3$ (4.6 mg, 0.0050 mmol, 5 mol%) and **L7** (5.5 mg, 0.010 mmol, 10 mol%). The tube was capped, evacuated and back-filled with argon for five times. Then degassed dry toluene (1.0 mL) and 1,3-cyclohexadiene **1a** (19.0 μL, 0.200 mmol, 2.0 equiv) were added via syringe sequentially under argon atmosphere. The mixture was stirred at 60 ℃ for 48 h. After complete consumption of **2b**, the mixture was directly purified by flash chromatography on silica gel (EtOAc/petroleum ether = 1/50) to afford product **4b**: 44.3 mg (0.0912 mmol), as a white solid, 91% yield; mp 199–201 °C; $[\alpha]_D^{25}$ = +272.6 (*c* = 0.27 in $CHCl_3$); >19:1 dr; 98% ee, determined by HPLC analysis (Daicel Chiralpak IG, *n*-Hexane/*i*-PrOH = 90/10, flow rate =1.0 mL/min, λ = 254 nm) t_R = 19.89 min (major), t_R = 22.13 min (minor); ^1H NMR (400 MHz, $CDCl_3$) δ (ppm) 8.20 (d, *J* = 7.6 Hz, 1H), 7.57 (d, *J* = 7.9 Hz, 2H), 7.35–7.25 (m, 5H), 7.11–7.02 (m, 1H), 6.74 (dd, *J* = 8.2, 2.5 Hz, 1H), 6.16 (s, 1H), 6.11 (d, *J* = 7.5 Hz, 1H), 5.77–5.67 (m, 1H), 5.57 (d, *J* = 10.3 Hz, 1H), 5.09–4.96 (m, 1H), 3.80 (d, *J* = 10.0 Hz, 1H), 3.75 (s, 3H), 2.41 (s, 3H), 2.29–2.15 (m, 1H), 2.14–2.00 (m, 1H), 1.84–1.71 (m, 1H), 1.71–1.60 (m, 2H); ^{13}C NMR (150 MHz, $CDCl_3$) δ (ppm) 159.7, 154.2, 149.8, 144.3, 141.2, 134.6, 130.0, 129.9, 129.3, 128.3, 127.9, 124.7, 124.4, 122.9, 122.5, 120.6, 117.0, 115.2, 111.2, 111.1, 58.3, 55.1, 39.2, 36.3, 22.2, 21.5, 20.6; HRMS (ESI-TOF) m/z: $[M + H]^+$ Calcd for $C_{29}H_{28}NO_4S^+$ 486.1734; Found 486.1741.

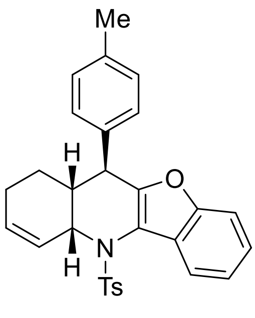

Synthesis of 4c: To an oven dried 10 mL Schlenk tube equipped with a stirring bar were added 4-methyl-*N*-((*E*)-2-((*Z*)-4-methylbenzylidene)benzofuran-3(2*H*)-ylidene)benzenesulfonamide **2c** (38.9 mg, 0.100 mmol, 1.0 equiv), $Pd_2(dba)_3$ (4.6 mg, 0.0050 mmol, 5 mol%) and **L7** (5.5 mg, 0.010 mmol, 10 mol%). The tube was capped, evacuated and back-filled with argon for five times. Then degassed dry toluene (1.0 mL) and 1,3-cyclohexadiene **1a** (19.0 μL, 0.200 mmol, 2.0 equiv) were added via syringe sequentially under argon atmosphere. The mixture was stirred at 60 ℃ for 48 h. After complete consumption of **2c**, the mixture was directly purified by flash chromatography on silica gel (EtOAc/petroleum ether = 1/50) to afford product **4c**: 42.3 mg (0.0901 mmol), as a white solid, 90% yield; mp 174–176 °C; $[\alpha]_D^{25}$ = +231.1 (*c* = 0.33 in

$CHCl_3$); >19:1 dr; 97% ee, determined by HPLC analysis (Daicel Chiralpak IG, *n*-Hexane/*i*-PrOH = 90/10, flow rate =1.0 mL/min, λ = 254 nm) t_R = 14.98 min (major), t_R = 19.85 min (minor); ^{1}H NMR (400 MHz, $CDCl_3$) δ (ppm) 8.20 (d, *J* = 7.7, 1H), 7.71–7.49 (m, 2H), 7.34–7.21 (m, 5H), 6.94 (d, *J* = 7.8 Hz, 2H), 6.49–6.29 (m, 2H), 5.81–5.65 (m, 1H), 5.59–5.55 (m, 1H), 5.04–5.02 (m, 1H), 3.79 (d, *J* = 9.9 Hz, 1H), 2.44 (s, 3H), 2.29 (s, 3H), 2.22–2.19 (m, 1H), 2.12–2.04 (m, 1H), 1.78–1.73 (m, 1H), 1.71–1.58 (m, 2H); ^{13}C NMR (100 MHz, $CDCl_3$) δ (ppm) 154.2, 150.3, 144.1, 137.0, 136.6, 134.8, 129.92, 129.85, 129.1, 128.4, 128.1, 124.8, 124.4, 123.0, 122.4, 116.9, 111.2, 58.3, 38.9, 36.6, 22.2, 21.6, 21.1, 20.6; HRMS (ESI-TOF) m/z: $[M + H]^+$ Calcd for $C_{29}H_{28}NO_3S^+$ 470.1784; Found 470.1793.

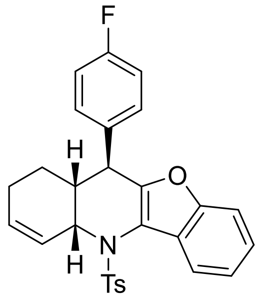

Synthesis of 4d: To an oven dried 10 mL Schlenk tube equipped with a stirring bar were added *N*-((*E*)-2-((*Z*)-4-fluorobenzylidene)benzofuran-3(2*H*) -ylidene)-4-methylbenzenesulfonamide **2d** (39.3 mg, 0.100 mmol, 1.0 equiv), $Pd_2(dba)_3$ (4.6 mg, 0.0050 mmol, 5 mol%) and **L7** (5.5 mg, 0.010 mmol, 10 mol%). The tube was capped, evacuated and back-filled with argon for five times. Then degassed dry toluene (1.0 mL) and 1,3-cyclohexadiene **1a** (19.0 μL, 0.200 mmol, 2.0 equiv) were added via syringe sequentially under argon atmosphere. The mixture was stirred at 60 ℃ for 48 h. After complete consumption of **2d**, the mixture was directly purified by flash chromatography on silica gel (EtOAc/petroleum ether = 1/50) to afford product **4d**: 47.0 mg (0.0992 mmol), as a white solid, 99% yield; mp 187–189 °C; $[\alpha]_D^{25}$ = +213.8 (*c* = 0.58 in $CHCl_3$); >19:1 dr; 98% ee, determined by HPLC analysis (Daicel Chiralpak IG, *n*-Hexane/*i*-PrOH = 90/10, flow rate =1.0 mL/min, λ = 254 nm) t_R = 14.60 min (major), t_R = 19.85 min (minor); ^{1}H NMR (400 MHz, $CDCl_3$) δ (ppm) 8.20 (d, *J* = 7.6 Hz, 1H), 7.70–7.46 (m, 2H), 7.50–7.14 (m, 5H), 6.94–6.68 (m, 2H), 6.64–6.29 (m, 2H), 5.74–5.70 (m, 1H), 5.57 (dt, *J* = 10.2, 2.0 Hz, 1H), 5.07–5.00 (m, 1H), 3.81 (d, *J* = 9.9 Hz, 1H), 2.45 (s, 3H), 2.33–1.93 (m, 2H), 1.73–1.64 (m, 2H), 1.61–1.59 (m, 1H); ^{13}C NMR (100 MHz, $CDCl_3$) δ (ppm) 161.9 (d, $^1J_{FC}$ = 246.2 Hz), 154.2, 149.7, 144.1, 135.39, 135.36, 134.8, 129.91, 129.87, 129.6 (d, $^3J_{FC}$ = 7.9 Hz), 128.3, 128.1, 124.64, 124.56, 123.1, 122.5, 117.0, 115.3 (d, $^2J_{FC}$ = 21.3 Hz), 111.1, 58.3, 38.5, 36.7, 22.0, 21.6, 20.5; ^{19}F NMR (375 MHz, $CDCl_3$) δ (ppm) –115.1; HRMS (ESI-TOF) m/z: $[M + Na]^+$ Calcd for $C_{28}H_{24}FNO_3SNa^+$ 496.1353; Found 496.1359.

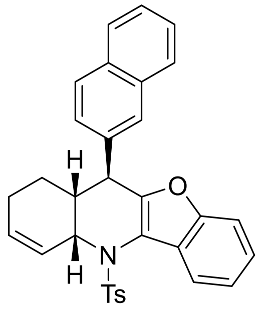

Synthesis of 4e: To an oven dried 10 mL Schlenk tube equipped with a stirring bar were added 4-methyl-*N*-((2*Z*,3*E*)-2-(naphthalen-2-ylmethylene)-benzofuran-3(2*H*)-ylidene)benzenesulfonamide **2e** (42.6 mg, 0.100 mmol, 1.0 equiv), $Pd_2(dba)_3$ (4.6 mg, 0.0050 mmol, 5 mol%) and **L7** (5.5 mg, 0.010 mmol, 10 mol%). The tube was capped, evacuated and back-filled with argon for five times. Then degassed dry toluene (1.0 mL) and 1,3-cyclohexadiene **1a** (19.0 μL, 0.200 mmol, 2.0 equiv) were added via syringe sequentially under argon atmosphere. The mixture was stirred at 60 ℃ for 48 h. After complete consumption of **2e**, the mixture was directly purified by flash chromatography on silica gel (EtOAc/petroleum ether = 1/50) to afford product **4e**: 44.6 mg (0.0882 mmol), as a white solid, 88% yield; mp 240–242 °C; $[\alpha]_D^{25}$ = +343.4 (*c* = 0.39 in $CHCl_3$); >19:1 dr; 97% ee, determined by HPLC analysis (Daicel Chiralpak IG, *n*-Hexane/*i*-PrOH = 90/10, flow rate =1.0 mL/min, λ = 254 nm) t_R = 19.67 min (major), t_R = 25.58 min (minor); ^{1}H NMR (400 MHz, $CDCl_3$) δ (ppm) 8.24 (d, *J* = 7.9 Hz, 1H), 7.81–7.73 (m, 1H), 7.71–7.58 (m, 3H), 7.54 (d, *J* = 8.5 Hz, 1H), 7.50–7.38 (m, 2H), 7.37–7.18 (m, 7H), 6.38 (dd, *J* = 8.4, 1.8 Hz, 1H), 5.81–5.71 (m, 1H), 5.66–5.56 (m, 1H), 5.14–5.02 (m, 1H), 4.01 (d, *J* = 9.9 Hz, 1H), 2.45 (s, 3H), 2.37–2.24 (m, 1H), 2.22–2.01 (m, 1H), 1.89–1.84 (m, 1H), 1.70–1.63 (m, 2H); ^{13}C NMR (100 MHz, $CDCl_3$) δ (ppm) 154.2, 149.9, 144.2, 137.0, 134.8, 133.2, 132.6, 130.01, 129.99, 128.3, 128.1, 127.6, 127.5, 127.4, 126.2, 125.9, 125.6, 124.7, 124.5, 123.0, 122.5, 117.2, 111.1, 58.3, 39.4, 36.4, 22.1, 21.7, 20.7; HRMS (ESI-TOF) m/z: $[M + Na]^+$ Calcd for $C_{32}H_{27}NO_3SNa^+$ 528.1604; Found 528.1609.

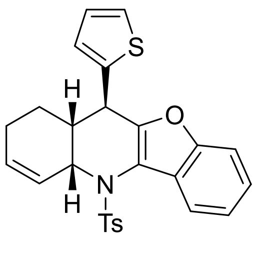

Synthesis of 4f: To an oven dried 10 mL Schlenk tube equipped with a stirring bar were added 4-methyl-*N*-((*2Z,3E*)-2-(thiophen-2-ylmethylene)benzofuran-3(*2H*)-ylidene)benzenesulfonamide **2f** (38.1 mg, 0.100 mmol, 1.0 equiv), $Pd_2(dba)_3$ (4.6 mg, 0.0050 mmol, 5 mol%) and **L7** (5.5 mg, 0.010 mmol, 10 mol%). The tube was capped, evacuated and back-filled with argon for five times. Then degassed dry toluene (1.0 mL) and 1,3-cyclohexadiene **1a** (19.0 μL, 0.200 mmol, 2.0 equiv) were added via syringe sequentially under argon atmosphere. The mixture was stirred at 60 ℃ for 48 h. After complete consumption of **2f**, the mixture was directly purified by flash chromatography on silica gel (EtOAc/petroleum ether = 1/50) to afford product **4f**: 44.4 mg (0.0962 mmol), as a white solid, 96% yield; mp 169–171 °C; $[\alpha]_D^{25}$ = +216.6 (*c* = 0.27 in $CHCl_3$); >19:1 dr; 97% ee, determined by HPLC analysis (Daicel Chiralpak IG, *n*-Hexane/*i*-PrOH = 90/10, flow rate

=1.0 mL/min, λ = 254 nm) t_R = 16.48 min (major), t_R = 20.26 min (minor); ^{1}H NMR (400 MHz, $CDCl_3$) δ (ppm) 8.24–8.13 (m, 1H), 7.55 (d, *J* = 8.3 Hz, 2H), 7.36–7.30 (m, 2H), 7.29 (d, *J* = 2.0 Hz, 1H), 7.28–7.24 (m, 2H), 7.12 (dd, *J* = 5.1, 1.2 Hz, 1H), 6.87 (dd, *J* = 5.2, 3.5 Hz, 1H), 6.51 (dd, *J* = 3.6, 1.2 Hz, 1H), 5.72–5.68 (m, 1H), 5.56 (dt, *J* = 10.2, 2.0 Hz, 1H) , 5.03–4.90 (m, 1H), 4.17 (d, *J* = 9.9 Hz, 1H), 2.41 (s, 3H), 2.25–1.98 (m, 2H), 1.83–1.63 (m, 3H); ^{13}C NMR (100 MHz, $CDCl_3$) δ (ppm) 154.2, 148.7, 144.1, 142.0, 134.5, 130.03, 129.97, 128.2, 127.8, 126.6, 126.0, 124.7, 124.6, 124.4, 123.0, 122.7, 116.5, 111.2, 58.1, 36.6, 34.4, 22.6, 21.6, 20.4; HRMS (ESI-TOF) m/z: [M + Na]$^+$ Calcd for $C_{26}H_{23}NO_3S_2Na^+$ 484.1012; Found 484.1021.

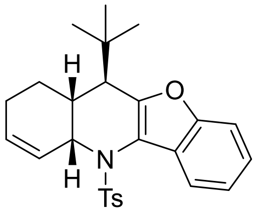

Synthesis of 4g: To an oven dried 10 mL Schlenk tube equipped with a stirring bar were added *N*-((2*Z*,3*E*)-2-(2,2-dimethylpropylidene)benzofuran-3(2*H*)-ylidene)-4-methylbenzenesulfonamide **2g** (35.5 mg, 0.100 mmol, 1.0 equiv), $Pd_2(dba)_3$ (4.6 mg, 0.0050 mmol, 5 mol%) and **L7** (5.5 mg, 0.010 mmol, 10 mol%). The tube was capped, evacuated and back-filled with argon for five times. Then degassed dry toluene (1.0 mL) and 1,3-cyclohexadiene **1a** (19.0 μL, 0.200 mmol, 2.0 equiv) were added via syringe sequentially under argon atmosphere. The mixture was stirred at 60 ℃ for 48 h. After complete consumption of **2g**, the mixture was directly purified by flash chromatography on silica gel (EtOAc/petroleum ether = 1/50) to afford product **4g**: 40.0 mg (0.0918 mmol), as a white solid, 92% yield; mp 173–175 ℃; $[\alpha]_D^{25}$ = +60 (*c* = 0.37 in $CHCl_3$); >19:1 dr; 98% ee, determined by HPLC analysis (Daicel Chiralpak IC, *n*-Hexane/*i*-PrOH = 90/10, flow rate =1.0 mL/min, λ = 254 nm) t_R = 8.38 min (major), t_R = 17.07 min (minor); ^{1}H NMR (400 MHz, $CDCl_3$) δ (ppm) 8.10–7.96 (m, 1H), 7.79–7.67 (m, 2H), 7.45–7.33 (m, 1H), 7.29–7.21 (m, 4H), 5.66–5.61 (m, 1H), 5.30–5.29 (m, 1H), 4.90–4.70 (m, 1H), 2.62 (d, *J* = 7.8 Hz, 1H), 2.39 (s, 3H), 2.37–2.28 (m, 1H), 2.19–2.08 (m, 1H), 2.02–1.79 (m, 3H), 0.74 (s, 9H); ^{13}C NMR (100 MHz, $CDCl_3$) δ (ppm) 153.72, 153.66, 143.8, 136.6, 130.8, 129.8, 128.9, 128.0, 124.9, 123.9, 122.8, 121.4, 115.7, 110.9, 58.6, 42.3, 35.0, 33.5, 28.5, 26.4, 21.5, 20.3; HRMS (ESI-TOF) m/z: [M + Na]$^+$ Calcd for $C_{26}H_{29}NO_3SNa^+$ 458.1760; Found 458.1767.

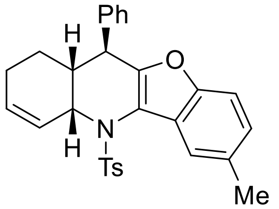

Synthesis of 4h: To an oven dried 10 mL Schlenk tube equipped with a stirring bar were added *N*-((*E*)-2-((*Z*)-benzylidene)-5-methylbenzofuran-3(2*H*)-ylidene)-4-methylbenzenesulfonamide **2h** (38.9 mg, 0.100 mmol, 1.0 equiv), $Pd_2(dba)_3$ (4.6 mg, 0.0050 mmol, 5 mol%) and **L7** (5.5 mg, 0.010 mmol, 10 mol%). The tube was capped, evacuated and back-filled with argon for five times. Then

degassed dry toluene (1.0 mL) and 1,3-cyclohexadiene **1a** (19.0 μL, 0.200 mmol, 2.0 equiv) were added via syringe sequentially under argon atmosphere. The mixture was stirred at 60 ℃ for 48 h. After complete consumption of **2h**, the mixture was directly purified by flash chromatography on silica gel (EtOAc/petroleum ether = 1/50) to afford product **4h**: 46.9 mg (0.0999 mmol), as a white solid, 99% yield; mp 193–195 °C; $[\alpha]_D^{25}$ = +259.4 (*c* = 0.34 in $CHCl_3$); >19:1 dr; 94% ee, determined by HPLC analysis (Daicel Chiralpak IG, *n*-Hexane/*i*-PrOH = 90/10, flow rate =1.0 mL/min, λ = 254 nm) t_R = 12.04 min (major), t_R = 14.18 min (minor); ^{1}H NMR (400 MHz, $CDCl_3$) δ (ppm) 7.97 (s, 1H), 7.59 (d, *J* = 8.3 Hz, 2H), 7.27 (d, *J* = 8.0 Hz, 2H), 7.22–7.04 (m, 5H), 6.60–6.43 (m, 2H), 5.78–5.66 (m, 1H), 5.62–5.50 (m, 1H), 5.08–4.98 (m, 1H), 3.80 (d, *J* = 9.9 Hz, 1H), 2.49 (s, 3H), 2.44 (s, 3H), 2.30–2.16 (m, 1H), 2.15–1.98 (m, 1H), 1.80–1.74 (m, 1H), 1.71–1.58 (m, 2H); ^{13}C NMR (100 MHz, $CDCl_3$) δ (ppm) 152.7, 150.2, 144.0, 139.8, 134.8, 132.4, 129.89, 129.86, 128.34, 128.32, 128.2, 128.1, 127.2, 125.6, 124.7, 122.1, 116.7, 110.6, 58.3, 39.3, 36.6, 22.1, 21.6, 21.5, 20.6; HRMS (ESI-TOF) m/z: $[M + Na]^+$ Calcd for $C_{29}H_{27}NO_3SNa^+$ 492.1604; Found 492.1604.

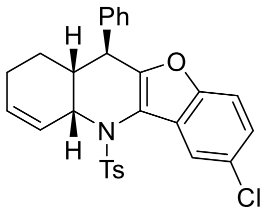

Synthesis of 4i: To an oven dried 10 mL Schlenk tube equipped with a stirring bar were added *N*-((*E*)-2-((*Z*)-benzylidene)-5-chlorobenzofuran-3(2*H*)-ylidene) -4-methylbenzenesulfonamide **2i** (41.0 mg, 0.100 mmol, 1.0 equiv), $Pd_2(dba)_3$ (4.6 mg, 0.0050 mmol, 5 mol%) and **L7** (5.5 mg, 0.010 mmol, 10 mol%). The tube was capped, evacuated and back-filled with argon for five times. Then degassed dry toluene (1.0 mL) and 1,3-cyclohexadiene **1a** (19.0 μL, 0.200 mmol, 2.0 equiv) were added via syringe sequentially under argon atmosphere. The mixture was stirred at 60 ℃ for 48 h. After complete consumption of **2i**, the mixture was directly purified by flash chromatography on silica gel (EtOAc/petroleum ether = 1/50) to afford product **4i**: 48.2 mg (0.0984 mmol), as a white solid, 98% yield; mp 188–190 ℃; $[\alpha]_D^{25}$ = +258.0 (*c* = 0.35 in $CHCl_3$); >19:1 dr; 93% ee, determined by HPLC analysis (Daicel Chiralpak IG, *n*-Hexane/*i*-PrOH = 90/10, flow rate =1.0 mL/min, λ = 254 nm) t_R = 14.15 min (minor), t_R = 17.53 min (major); ^{1}H NMR (400 MHz, $CDCl_3$) δ (ppm) 8.18 (d, *J* = 2.0 Hz, 1H), 7.59 (d, *J* = 8.0 Hz, 2H), 7.29 (d, *J* = 8.0 Hz, 2H), 7.25–7.18 (m, 3H), 7.17–7.10 (m, 2H), 6.55–6.45 (m, 2H), 5.80–5.70 (m, 1H), 5.60–5.48 (m, 1H), 5.08–4.97 (m, 1H), 3.80 (d, *J* = 9.9 Hz, 1H), 2.45 (s, 3H), 2.30–2.17 (m, 1H), 2.15–2.04 (m, 1H), 1.82–1.74 (m, 1H), 1.73–1.59 (m, 2H); ^{13}C NMR (100 MHz, $CDCl_3$) δ (ppm) 152.6, 151.6, 144.3, 139.3, 134.6, 130.2, 130.0, 128.8, 128.4, 128.12, 128.07, 127.4, 126.0, 124.7, 122.1, 116.6, 112.1, 58.3, 39.3, 36.5, 22.0, 21.6, 20.5; HRMS (ESI-TOF) m/z: [M +

Na]$^+$ Calcd for $C_{28}H_{24}{}^{35}ClNO_3SNa^+$ 512.1058; Found 512.1060; Calcd for $C_{28}H_{24}{}^{37}ClNO_3SNa^+$ 514.1029; Found 514.1038.

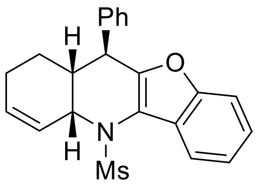

Synthesis of 4j: To an oven dried 10 mL Schlenk tube equipped with a stirring bar were added *N*-((*E*)-2-((*Z*)-benzylidene)benzofuran-3(2*H*)-ylidene)-4-methylbenzenesulfonamide **2j** (30.0 mg, 0.100 mmol, 1.0 equiv), $Pd_2(dba)_3$ (4.6 mg, 0.0050 mmol, 5 mol%) and **L7** (5.5 mg, 0.010 mmol, 10 mol%). The tube was capped, evacuated and back-filled with argon for five times. Then degassed dry toluene (1.0 mL) and 1,3-cyclohexadiene **1a** (19.0 μL, 0.200 mmol, 2.0 equiv) were added via syringe sequentially under argon atmosphere. The mixture was stirred at 60 °C for 48 h. After complete consumption of **2j**, the mixture was directly purified by flash chromatography on silica gel (EtOAc/petroleum ether = 1/50) to afford product **4j**: 33.6 mg (0.0885 mmol), as a white solid, 89% yield; mp 89–91 ℃; $[\alpha]_D^{25}$ = +10.9 (*c* = 0.55 in $CHCl_3$); >19:1 dr; 97% ee, determined by HPLC analysis (Daicel Chiralpak IG, *n*-Hexane/*i*-PrOH = 90/10, flow rate =1.0 mL/min, λ = 254 nm) t_R = 18.26 min (major), t_R = 42.92 min (minor); ^{1}H NMR (400 MHz, $CDCl_3$) δ (ppm) 8.08–7.88 (m, 1H), 7.41–7.28 (m, 4H), 7.26–7.19 (m, 4H), 5.87–5.74 (m, 1H), 5.61–5.51 (m, 1H), 5.05–4.94 (m, 1H), 4.09 (d, *J* = 9.8 Hz, 1H), 3.10 (s, 3H), 2.58–2.49 (m, 1H), 2.43–2.29 (m, 1H), 2.25–2.13 (m, 1H), 1.97–1.80 (m, 2H); ^{13}C NMR (100 MHz, $CDCl_3$) δ (ppm) 154.2, 149.1, 139.8, 130.3, 129.0, 128.2, 128.1, 127.6, 124.6, 124.2, 123.0, 122.0, 117.1, 111.2, 58.0, 39.6, 38.6, 38.4, 22.3, 20.6; HRMS (ESI-TOF) m/z: [M + Na]$^+$ Calcd for $C_{22}H_{21}NO_3SNa^+$ 402.1134; Found 402.1142.

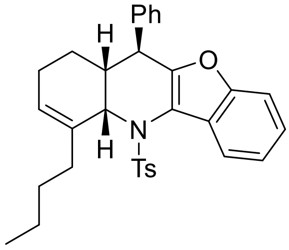

Synthesis of 4k: To an oven dried 10 mL Schlenk tube equipped with a stirring bar were added *N*-((*E*)-2-((*Z*)-benzylidene)benzofuran-3(2*H*)-ylid ene)-4-methylbenzenesulfonamide **2a** (37.5 mg, 0.100 mmol, 1.0 equiv), 2-butylcyclohexa-1,3-diene **1b** (27.2 mg, 0.200 mmol, 2.0 equiv), $Pd_2(dba)_3$ (4.6 mg, 0.0050 mmol, 5 mol%) and **L7** (5.5 mg, 0.010 mmol, 10 mol%). Then distilled toluene (1.0 mL) was added via syringe. The tube was evacuated followed by back-filled with argon for five times. The mixture was stirred at 80 ℃ for 48 h. After complete consumption of **2a**, the mixture was directly purified by flash chromatography on silica gel (EtOAc/petroleum ether = 1/100) to afford product **4k**: 25.6 mg (0.0500 mmol), as a white solid, 50% yield; mp 237–239 ℃; $[\alpha]_D^{25}$ = +149.2 (*c* = 0.25 in $CHCl_3$); >19:1 dr; 90% ee, determined by HPLC analysis (Daicel Chiralpak IG, *n*-Hexane/*i*-PrOH = 90/10, flow rate =1.0 mL/min, λ = 254 nm) t_R = 7.78 min

(major), t_R = 9.24 min (minor); ^{1}H NMR δ (ppm) 8.20 (d, J = 7.7 Hz, 1H), 7.58 (d, J = 7.9 Hz, 2H), 7.33–7.26 (m, 5H), 7.21–7.16 (m, 1H), 7.14–7.08 (m, 2H), 6.51–6.43 (m, 2H), 5.44 (s, 1H), 5.08 (s, 1H), 3.85 (d, J = 10.1 Hz, 1H), 2.44 (s, 3H), 2.32–2.19 (m, 1H), 2.16–2.02 (m, 2H), 1.88–1.74 (m, 2H), 1.69–1.51 (m, 3H), 1.36–1.22 (m, 3H), 0.87 (t, J = 7.1 Hz, 3H); ^{13}C NMR (100 MHz, $CDCl_3$) δ (ppm) 154.2, 149.1, 139.8, 130.3, 129.0, 128.2, 128.1, 127.6, 124.6, 124.2, 123.0, 122.0, 117.0, 111.2, 58.0, 39.6, 38.6, 38.4, 22.3, 20.6; HRMS (ESI-TOF) m/z: $[M + Na]^+$ Calcd for $C_{32}H_{33}NO_3SNa^+$ 534.2073; Found 534.2075.

4.3 General procedure for asymmetric synthesis of diastereomers 5

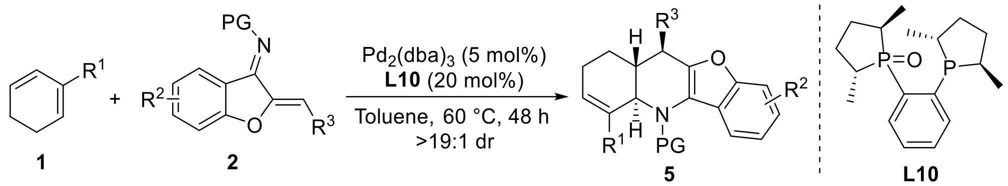

General procdure: To an oven dried 10 mL Schlenk tube equipped with a stirring bar were added 1-azadiene **2** (0.100 mmol, 1.0 equiv), $Pd_2(dba)_3$ (5 mol%) and **L10** (20 mol%). The tube was capped, evacuated and back-filled with argon for five times. Then degassed dry toluene (1.0 mL) and 1,3-cyclohexadiene **1** (0.200 mmol, 2.0 equiv) were added via syringe sequentially under argon atmosphere. The mixture was stirred at 60 °C for 48 h. After complete consumption of **2**, the mixture was directly purified by flash chromatography on silica gel (EtOAc/petroleum ether = 1/50) to afford product **5**.

*Note: Racemic **5** was obtained by using $Pd_2(dba)_3$ in combination with racemic **L10**. The dr indicated the diastereomeric purity of the isolated products.*

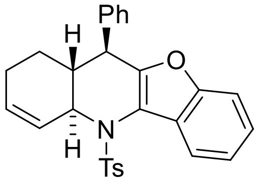

Synthesis of 5a: To an oven dried 10 mL Schlenk tube equipped with a stirring bar were added *N*-((*E*)-2-((Z)-benzylidene)benzofuran-3(2*H*)-ylidene)-4-methylbenzenesulfonamide **2a** (37.5 mg, 0.100 mmol, 1.0 equiv), $Pd_2(dba)_3$ (4.6 mg, 0.0050 mmol, 5 mol%) and **L10** (6.4 mg, 0.020 mmol, 20 mol%). The tube was capped, evacuated and back-filled with argon for five times. Then degassed dry toluene (1.0 mL) and 1,3-cyclohexadiene **1a** (19.0 μL, 0.200 mmol, 2.0 equiv) were added via syringe sequentially under argon atmosphere. The mixture was stirred at 60 ℃ for 48 h. After complete consumption of **2a**, the mixture was directly purified by flash chromatography on silica gel (EtOAc/petroleum ether

= /50) to afford product **5a**: 39.2 mg (0.0860 mmol), as a white solid, 86% yield; mp 174–176 ℃; $[\alpha]_D^{25}$ = +207.3 (*c* = 0.28 in $CHCl_3$); >19:1 dr; 82% ee, determined by HPLC analysis (Daicel Chiralpak AD, *n*-Hexane/*i*-PrOH = 90/10, flow rate =1.0 mL/min, λ = 254 nm) t_R = 11.75 min (major), t_R = 19.31 min (minor); ^1H NMR (400 MHz, $CDCl_3$) δ (ppm) 8.12 (d, *J* = 7.5, 1H), 7.65 (d, *J* = 8.0 Hz, 2H), 7.31–7.18 (m, 8H), 6.78–6.67 (m, 2H), 6.42 (dt, *J* = 10.6, 2.8 Hz, 1H), 5.99–5.95 (m, 1H), 4.28–4.11 (m, 1H), 3.53 (d, *J* = 10.2 Hz, 1H), 2.42 (s, 3H), 2.03–1.94 (m, 1H), 1.90–1.72 (m, 1H), 1.68–1.53 (m, 2H), 1.30–1.21 (m, 1H); ^{13}C NMR (100 MHz, $CDCl_3$) δ (ppm) 153.9, 149.9, 144.0, 138.8, 135.8, 129.6, 129.1, 129.0, 128.5, 128.3, 127.4, 126.0, 125.2, 124.4, 123.0, 122.5, 122.0, 111.1, 64.5, 48.3, 38.9, 26.3, 24.3, 21.6; HRMS (ESI-TOF) m/z: $[M + Na]^+$ Calcd for $C_{28}H_{25}NO_3SNa^+$ 478.1447; Found 478.1447.

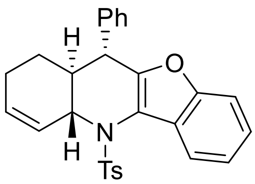

Synthesis of *ent*-5a: To an oven dried 10 mL Schlenk tube equipped with a stirring bar were added *N*-((*E*)-2-((*Z*)-benzylidene)benzofuran-3(2*H*)-ylidene)-4-methylbenzenesulfonamide **2a** (37.5 mg, 0.100 mmol, 1.0 equiv), $Pd_2(dba)_3$ (4.6 mg, 0.0050 mmol, 5 mol%) and *ent*-**L10** (6.4 mg, 0.020 mmol, 20 mol%). The tube was capped, evacuated and back-filled with argon for five times. Then degassed dry toluene (1.0 mL) and 1,3-cyclohexadiene **1a** (19.0 μL, 0.200 mmol, 2.0 equiv) were added via syringe sequentially under argon atmosphere. The mixture was stirred at 60 °C for 48 h. After complete consumption of **2a**, the mixture was directly purified by flash chromatography on silica gel (EtOAc/petroleum ether =1/50) to afford product *ent*-**5a**: 41.0 mg (0.0878 mmol), as a white solid, 88% yield; $[\alpha]_D^{25}$ = –219.7 (*c* = 0.31 in $CHCl_3$); >19:1 dr; 83% ee, determined by HPLC analysis (Daicel Chiralpak AD, n-Hexane/i-PrOH = 90/10, flow rate =1.0 mL/min, λ = 254 nm) t_R = 11.78 min (minor), t_R = 19.16 min (major).

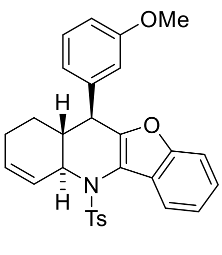

Synthesis of 5b: To an oven dried 10 mL Schlenk tube equipped with a stirring bar were add *N*-((*E*)-2-((*Z*)-3-methoxybenzylidene)benzofuran-3(2*H*)-ylidene)-4-methylbenzenesulfonamide **2b** (40.5 mg, 0.100 mmol, 1.0 equiv), $Pd_2(dba)_3$ (4.6 mg, 0.0050 mmol, 5 mol%) and **L10** (6.4 mg, 0.020 mmol, 20 mol%). The tube was capped, evacuated and back-filled with argon for five times. Then degassed dry toluene (1.0 mL) and 1,3-cyclohexadiene **1a** (19.0 μL, 0.200 mmol, 2.0 equiv) were added via syringe sequentially under argon atmosphere. The mixture was stirred at 60 ℃ for 48 h. After complete consumption of **2b**, the mixture was directly purified by flash chromatography on silica gel

(EtOAc/petroleum ether = 1/50) to afford product **5b**: 35.8 mg (0.0737 mmol), as a white solid, 74% yield; mp 198–200 °C; $[\alpha]_D^{25}$ = +173.3 (*c* = 0.27 in $CHCl_3$); >19:1 dr; 77% ee, determined by HPLC analysis (Daicel Chiralpak IG, *n*-Hexane/*i*-PrOH = 80/20, flow rate =1.0 mL/min, λ = 254 nm) t_R = 19.62 min (minor), t_R = 21.05 min (major); ^1H NMR (400 MHz, $CDCl_3$) δ (ppm) 8.11 (d, *J* = 7.8 Hz, 1H), 7.64 (d, *J* = 8.1 Hz, 2H), 7.34–7.26 (m, 4H), 7.18–7.07 (m, 1H), 6.80–6.74 (m, 1H), 6.46–6.38 (m, 1H), 6.36–6.28 (m, 2H), 6.02–5.93 (m, 1H), 4.24–4.12 (m 1H), 3.78 (s, 3H), 3.50 (d, *J* = 10.3 Hz, 1H), 2.40 (s, 3H), 2.05–1.92 (m, 1H), 1.85–1.74 (m, 1H), 1.69–1.62 (m, 1H), 1.60–1.51 (m, 2H), 1.26–1.23 (m, 1H); ^{13}C NMR (100 MHz, $CDCl_3$) δ (ppm) 159.6, 153.9, 149.8, 144.1, 140.3, 135.6, 129.8, 129.2, 129.0, 128.9, 126.0, 125.2, 124.4, 122.9, 122.6, 122.0, 120.9, 115.3, 111.5, 111.1, 64.4, 55.1, 48.3, 38.7, 26.4, 24.2, 21.6; HRMS (ESI-TOF) m/z: $[M + Na]^+$ Calcd for $C_{29}H_{27}NO_4SNa^+$ 508.1553; Found 508.1555.

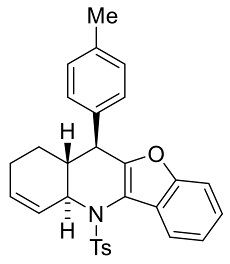

Synthesis of 5c: To an oven dried 10 mL Schlenk tube equipped with a stirring bar were added 4-methyl-*N*-((*E*)-2-((*Z*)-4-methylbenzylidene)benzofuran-3(2*H*)-ylidene)benzenesulfonamide **2c** (38.9 mg, 0.100 mmol, 1.0 equiv), $Pd_2(dba)_3$ (4.6 mg, 0.0050 mmol, 5 mol%) and **L10** (6.4 mg, 0.020 mmol, 20 mol%). The tube was capped, evacuated and back-filled with argon for five times. Then degassed dry toluene (1.0 mL) and 1,3-cyclohexadiene **1a** (19.0 μL, 0.200 mmol, 2.0 equiv) were added via syringe sequentially under argon atmosphere. The mixture was stirred at 60 °C for 48 h. After complete consumption of **2c**, the mixture was directly purified by flash chromatography on silica gel (EtOAc/petroleum ether = 1/50) to afford product **5c**: 41.1 mg (0.0875 mmol), as a white solid, 88% yield; mp 187–189 °C; $[\alpha]_D^{25}$ = +193.1 (*c* = 0.44 in $CHCl_3$); >19:1 dr; 82% ee, determined by HPLC analysis (Daicel Chiralpak IG, *n*-Hexane/*i*-PrOH = 90/10, flow rate =1.0 mL/min, λ = 254 nm) t_R = 13.20 min (major), t_R = 21.41 min (minor); ^1H NMR (400 MHz, $CDCl_3$) δ (ppm) 8.16–8.02 (m, 1H), 7.64 (d, *J* = 8.0 Hz, 2H), 7.33–7.27 (m, 3H), 7.26–7.21 (m, 2H), 7.00 (d, *J* = 7.7 Hz, 2H), 6.61 (d, *J* = 7.9 Hz, 2H), 6.47–6.34 (m, 1H), 6.03–5.90 (m, 1H), 4.27–4.12 (m, 1H), 3.50 (d, *J* = 10.3 Hz, 1H), 2.43 (s, 3H), 2.32 (s, 3H), 2.08–1.90 (m, 1H), 1.85–1.70 (m, 1H), 1.68–1.60 (m, 2H), 1.28–1.15 (m, 1H); ^{13}C NMR (100 MHz, $CDCl_3$) δ (ppm) 153.9, 150.1, 144.0, 137.1, 135.8, 135.7, 129.6, 129.11, 129.06, 129.0, 128.3, 126.1, 125.2, 124.3, 122.9, 122.5, 121.9, 111.2, 64.5, 48.0, 38.9, 26.3, 24.3, 21.6, 21.1; HRMS (ESI-TOF) m/z: $[M + Na]^+$ Calcd for $C_{29}H_{27}NO_3SNa^+$ 492.1604; Found 492.1606.

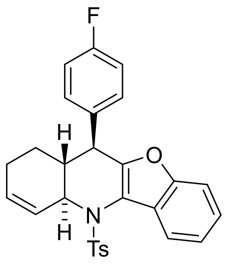

Synthesis of 5d: To an oven dried 10 mL Schlenk tube equipped with a stirring bar were added *N*-((*E*)-2-((*Z*)-4-fluorobenzylidene)benzofuran-3(2*H*) -ylidene)-4-methylbenzenesulfonamide **2d** (39.3 mg, 0.100 mmol, 1.0 equiv), $Pd_2(dba)_3$ (4.6 mg, 0.0050 mmol, 5 mol%) and **L10** (6.4 mg, 0.020 mmol, 20 mol%). The tube was capped, evacuated and back-filled with argon for five times. Then degassed dry toluene (1.0 mL) and 1,3-cyclohexadiene **1a** (19.0 μL, 0.200 mmol, 2.0 equiv) were added via syringe sequentially under argon atmosphere. The mixture was stirred at 60 ℃ for 48 h. After complete consumption of **2d**, the mixture was directly purified by flash chromatography on silica gel (EtOAc/DCM/petroleum ether = 1/1/50) to afford product **5d**: 39.3 mg (0.0830 mmol), as a white solid, 83% yield; mp 121–123 ℃; $[\alpha]_D^{25}$ = +165.2 (*c* = 0.62 in $CHCl_3$); >19:1 dr; 79% ee, determined by HPLC analysis (Daicel Chiralpak AD, *n*-Hexane/*i*-PrOH = 90/10, flow rate =1.0 mL/min, λ = 254 nm) t_R = 13.47 min (major), t_R = 19.21 min (minor); ^1H NMR (400 MHz, $CDCl_3$) δ (ppm) 8.11 (d, *J* = 7.8 Hz, 1H), 7.65 (d, *J* = 8.0 Hz, 2H), 7.35–7.27 (m, 4H), 6.96–6.78 (m, 2H), 6.75–6.54 (m, 2H), 6.40 (d, *J* = 10.5 Hz, 1H), 6.06–5.88 (m, 1H), 4.24–4.13 (m, 1H), 3.53 (d, *J* = 10.3 Hz, 1H), 2.43 (s, 3H), 2.07–1.92 (m, 1H), 1.87–1.72 (m, 1H), 1.67–1.59 (m, 2H), 1.27–1.20 (m, 1H); ^{13}C NMR (100 MHz, $CDCl_3$) δ (ppm) 162.1 (d, $^1J_{FC}$ = 247.0 Hz), 153.9, 149.6, 144.1, 135.8, 134.5, 129.9, 129.8, 129.9 (d, $^3J_{FC}$ = 8.0 Hz), 129.1, 129.0, 126.0, 125.1, 124.6, 123.1, 122.6, 122.1, 115.3 (d, $^2J_{FC}$ = 21.3 Hz), 111.1, 64.5, 47.5, 39.1, 26.3, 24.3, 21.6; ^{19}F NMR (375 MHz, $CDCl_3$) δ (ppm) –115.0; HRMS (ESI-TOF) m/z: $[M + Na]^+$ Calcd for $C_{28}H_{24}FNO_3SNa^+$ 496.1353; Found 496.1354. Recrystallisation of the above product from petroleum ether (7.0 mL) and EtOAc (1.0 mL) gave a white solid, 20.0 mg (0.0422 mmol), 42% yield, 91% ee.

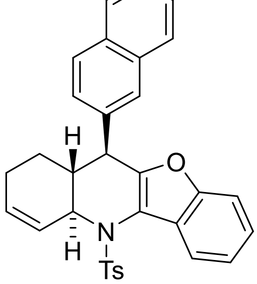

Synthesis of 5e: To an oven dried 10 mL Schlenk tube equipped with a stirring bar were added 4-methyl-*N*-((2*Z*,3*E*)-2-(naphthalen-2-ylmethylene)-benzo furan-3(2*H*)-ylidene)benzenesulfonamide **2e** (42.6 mg, 0.100 mmol, 1.0 equiv), $Pd_2(dba)_3$ (4.6 mg, 0.0050 mmol, 5 mol%) and **L10** (6.4 mg, 0.020 mmol, 20 mol%). The tube was capped, evacuated and back-filled with argon for five times. Then degassed dry toluene (1.0 mL) and 1,3-cyclohexadiene **1a** (19.0 μL, 0.200 mmol, 2.0 equiv) were added via syringe sequentially under argon atmosphere. The mixture was stirred at 60 ℃ for 48 h. After complete consumption of **2e**, the mixture was directly purified by flash chromatography on silica gel (EtOAc/DCM/petroleum ether = 1/25/50) to afford product **5e**:

42.9 mg (0.0848 mmol), as a white solid, 85% yield; mp 198–200 ℃; $[\alpha]_D^{25}$ = +371.1 (*c* = 0.24 in $CHCl_3$); >19:1 dr; 88% ee, determined by HPLC analysis (Daicel Chiralpak IG, *n*-Hexane/*i*-PrOH = 80/20, flow rate =1.0 mL/min, λ = 254 nm) t_R = 18.82 min (major), t_R = 20.63 min (minor); ^{1}H NMR (400 MHz, $CDCl_3$) δ (ppm) 8.15 (d, *J* = 7.8 Hz, 1H), 7.82–7.77 (m, 1H), 7.75–7.67 (m, 3H), 7.63 (d, *J* = 8.5 Hz, 1H), 7.50–7.43 (m, 2H), 7.38 (s, 1H), 7.33–7.25 (m, 5H), 6.70 (dd, *J* = 8.4, 1.8 Hz, 1H), 6.45 (dt, *J* = 10.4, 2.7 Hz,1H), 6.05–5.91 (m, 1H), 4.31–4.20 (m, 1H), 3.70 (d, *J* = 10.2 Hz, 1H), 2.43 (s, 3H), 2.03–1.92 (m, 1H), 1.80–1.63 (m, 3H), 1.37–1.23 (m, 1H); ^{13}C NMR (100 MHz, $CDCl_3$) δ (ppm) 154.0, 149.8, 144.1, 136.2, 135.8, 133.2, 132.7, 129.7, 129.1, 128.0, 127.7, 127.6, 127.5, 126.2, 126.0, 125.9, 125.2, 124.5, 123.0, 122.6, 122.2, 111.1, 64.4, 48.5, 38.8, 26.3, 24.2, 21.7; HRMS (ESI-TOF) m/z: $[M + Na]^+$ Calcd for $C_{32}H_{27}NO_3SNa^+$ 528.1604; Found 528.1605.

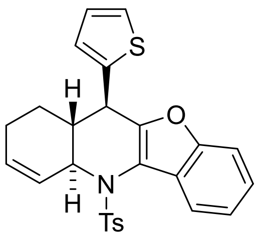

Synthesis of 5f: To an oven dried 10 mL Schlenk tube equipped with a stirring bar were added 4-methyl-*N*-((*2Z,3E*)-2-(thiophen-2-ylmethylene)benzofuran-3(*2H*)-ylidene)benzenesulfonamide **2f** (38.1 mg, 0.100 mmol, 1.0 equiv), $Pd_2(dba)_3$ (4.6 mg, 0.0050 mmol, 5 mol%) and **L10** (6.6 mg, 0.01 mmol, 10 mol%). The tube was capped, evacuated and back-filled with argon for five times. Then degassed dry toluene (1.0 mL) and 1,3-cyclohexadiene **1a** (19.0 μL, 0.200 mmol, 2.0 equiv) were added via syringe sequentially under argon atmosphere. The mixture was stirred at 60 ℃ for 48 h. After complete consumption of **2f**, the mixture was directly purified by flash chromatography on silica gel (EtOAc/petroleum ether = 1/50) to afford product **5f**: 39.8 mg (0.0862 mmol), as a white solid, 86% yield; mp 106–108 ℃; $[\alpha]_D^{25}$ = +161.3 (*c* = 0.48 in $CHCl_3$); >19:1 dr; 83% ee, determined by HPLC analysis (Daicel Chiralpak AD, *n*-Hexane/*i*-PrOH = 80/20, flow rate =1.0 mL/min, λ = 254 nm) t_R = 8.56 min (major), t_R = 10.07 min (minor); ^{1}H NMR (400 MHz, $CDCl_3$) δ (ppm) 8.11 (d, *J* = 7.5 Hz, 1H), 7.61 (d, *J* = 8.0 Hz, 2H), 7.39–7.13 (m, 6H), 6.96–6.83 (m, 1H), 6.71–6.58 (m, 1H), 6.43 (d, *J* = 10.6 Hz, 1H), 6.09–5.85 (m, 1H), 4.23–4.03 (m, 1H), 3.88 (d, *J* = 10.5 Hz, 1H), 2.40 (s, 3H), 2.07–1.93 (m, 1H), 1.84–1.68 (m, 2H), 1.55–1.42 (m, 1H), 1.30–1.15 (m, 1H); ^{13}C NMR (100 MHz, $CDCl_3$) δ (ppm) 153.9, 148.7, 144.0, 141.3, 135.6, 129.8, 129.3, 128.9, 126.6, 126.4, 125.7, 125.2, 124.6, 124.5, 123.0, 122.7, 121.6, 111.2, 64.2, 43.1, 39.1, 26.7, 24.3, 21.6; HRMS (ESI-TOF) m/z: $[M + Na]^+$ Calcd for $C_{26}H_{23}NO_3S_2Na^+$ 484.1012; Found 484.1012.

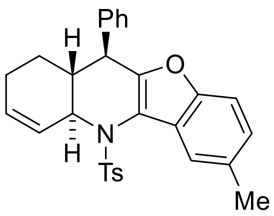

Synthesis of 5g: To an oven dried 10 mL Schlenk tube equipped with a stirring bar were added *N*-((*E*)-2-((*Z*)-benzylidene)-5-methylbenzofuran-3(2*H*)-ylidene)-4-methylbenzenesulfonamide **2h** (38.9 mg, 0.100 mmol, 1.0 equiv), $Pd_2(dba)_3$ (4.6 mg, 0.0050 mmol, 5 mol%) and **L10** (6.4 mg, 0.020 mmol, 20 mol%). The tube was capped, evacuated and back-filled with argon for five times. Then degassed dry toluene (1.0 mL) and 1,3-cyclohexadiene **1a** (19.0 μL, 0.200 mmol, 2.0 equiv) were added via syringe sequentially under argon atmosphere. The mixture was stirred at 60 ℃ for 48 h. After complete consumption of **2h**, the mixture was directly purified by flash chromatography on silica gel (EtOAc/petroleum ether = 1/50) to afford product **5g**: 40.9 mg (0.0871 mmol), as a white solid, 87% yield; mp 112–114 ℃; $[\alpha]_D^{25}$ = +242.6 (*c* = 0.39 in $CHCl_3$); >19:1 dr; 85% ee, determined by HPLC analysis (Daicel Chiralpak IG, *n*-Hexane/*i*-PrOH = 80/20, flow rate =1.0 mL/min, λ = 254 nm) t_R = 12.51 min (minor), t_R = 17.10 min (major); ^{1}H NMR (400 MHz, $CDCl_3$) δ (ppm) 7.90 (s, 1H), 7.69–7.63 (m, 2H), 7.29–7.26 (m, 1H), 7.25–7.24 (m, 1H), 7.24–7.13 (m, 4H), 7.06 (dd, *J* = 8.5, 1.8 Hz, 1H), 6.75–6.68 (m, 2H), 6.42 (dt, *J* = 10.6, 2.6 Hz, 1H), 6.01–5.92 (m, 1H), 4.22–4.13 (m, 1H), 3.51 (d, *J* = 10.2 Hz, 1H), 2.48 (s, 3H), 2.42 (s, 3H), 2.04–1.92 (m, 1H), 1.85–1.72 (m, 1H), 1.67–1.56 (m, 2H), 1.27–1.22 (m, 1H); ^{13}C NMR (100 MHz, $CDCl_3$) δ (ppm) 152.4, 150.1, 144.0, 138.9, 135.8, 132.5, 129.6, 129.1, 128.9, 128.5, 128.3, 127.4, 126.1, 125.7, 125.2, 122.2, 121.8, 110.7, 64.5, 48.4, 38.9, 26.3, 24.3, 21.6, 21.5; HRMS (ESI-TOF) m/z: $[M + Na]^+$ Calcd for $C_{29}H_{27}NO_3SNa^+$ 492.1604; Found 492.1601. Recrystallisation of the above product from petroleum ether (7.0 mL) and EtOAc (1.0 mL) gave a white solid, 20.2 mg (0.0422 mmol), 42% yield, 97% ee.

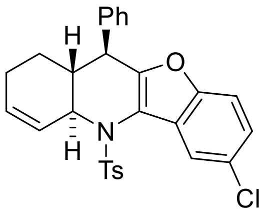

Synthesis of 5h: To an oven dried 10 mL Schlenk tube equipped with a stirring bar were added *N*-(*E*)-2-((*Z*)-benzylidene)-5-chlorobenzofuran-3(2*H*)-ylidene)-4-methylbenzenesulfonamide **2i** (41.0 mg, 0.100 mmol, 1.0 equiv), $Pd_2(dba)_3$ (4.6 mg, 0.0050 mmol, 5 mol%) and **L10** (6.4 mg, 0.020 mmol, 20 mol%). The tube was capped, evacuated and back-filled with argon for five times. Then degassed dry toluene (1.0 mL) and 1,3-cyclohexadiene **1a** (19.0 μL, 0.200 mmol, 2.0 equiv) were added via syringe sequentially under argon atmosphere. The mixture was stirred at 60 ℃ for 48 h. After complete consumption of **2i**, the mixture was directly purified by flash chromatography on silica gel (EtOAc/petroleum ether = 1/50) to afford product **5h**: 41.7 mg (0.0851 mmol), as a white

solid, 85% yield; mp 108–110 °C; $[\alpha]_D^{25}$ = +226.9 (*c* = 0.48 in $CHCl_3$); >19:1 dr; 87% ee, determined by HPLC analysis (Daicel Chiralpak IA, *n*-Hexane/*i*-PrOH = 60/40, flow rate =1.0 mL/min, λ = 254 nm) t_R = 5.49 min (minor), t_R = 7.18 min (major); ^{1}H NMR (400 MHz, $CDCl_3$) δ (ppm) 8.12–8.07 (m, 1H), 7.68 – 7.62 (m, 2H), 7.31–7.26 (m, 2H), 7.25–7.16 (m, 5H), 6.75–6.67 (m, 2H), 6.40 (dt, *J* = 10.7, 2.7 Hz, 1H), 6.02–5.90 (m, 1H), 4.23–4.14 (m, 1H), 3.52 (d, *J* = 9.9 Hz, 1H), 2.45 (s, 3H), 2.09–1.93 (m, 1H), 1.89–1.74 (m, 1H), 1.70–1.57 (m, 2H), 1.28–1.22 (m, 1H); ^{13}C NMR (100 MHz, $CDCl_3$) δ (ppm) 152.3, 151.4, 144.2, 138.4, 135.6, 129.7, 129.2, 129.1, 128.8, 128.42, 128.39, 127.5, 126.5, 125.8, 124.7, 122.3, 121.6, 112.1, 64.5, 48.3, 38.9, 26.3, 24.2, 21.6; HRMS (ESI-TOF) m/z: $[M + Na]^+$ Calcd for $C_{28}H_{24}{}^{35}ClNO_3SNa^+$ 512.1058; Found 512.1061; Calcd for $C_{28}H_{24}{}^{37}ClNO_3SNa^+$ 514.1028; Found 514.1041. Recrystallisation of the above product from petroleum ether (7.0 mL) and EtOAc (1.0 mL) gave a white solid, 27.2 mg (0.0555 mmol), 56% yield, 98% ee.

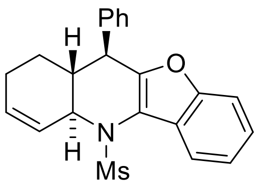

Synthesis of 5i: To an oven dried 10 mL Schlenk tube equipped with a stirring bar were add *N*-((*E*)-2-((*Z*)-benzylidene)benzofuran-3(2*H*)-ylidene)-4-methyl benzenesulfonamide **2j** (30.0 mg, 0.100 mmol, 1.0 equiv), $Pd_2(dba)_3$ (4.6 mg, 0.0050 mmol, 5 mol%) and **L10** (6.4 mg, 0.020 mmol, 20 mol%). The tube was capped, evacuated and back-filled with argon for five times. Then degassed dry toluene (1.0 mL) and 1,3-cyclohexadiene **1a** (19.0 μL, 0.200 mmol, 2.0 equiv) were added via syringe sequentially under argon atmosphere. The mixture was stirred at 60 °C for 48 h. After complete consumption of **2j**, the mixture was directly purified by flash chromatography on silica gel (EtOAc/petroleum ether = 1/50) to afford product **5i**: 30.6 mg (0.0806 mmol), as a white solid, 81% yield; mp 166–168 °C; $[\alpha]_D^{25}$ = +24.5 (*c* = 0.22 in $CHCl_3$); >19:1 dr; 70% ee, determined by HPLC analysis (Daicel Chiralpak IG, *n*-Hexane/*i*-PrOH = 90/10, flow rate =1.0 mL/min, λ = 254 nm) t_R = 20.91 min (major), t_R = 42.24 min (minor); ^{1}H NMR (400 MHz, $CDCl_3$) δ (ppm) 7.75 (dd, *J* = 6.8, 2.2 Hz, 1H), 7.34–7.25 (m, 3H), 7.21–7.13 (m, 5H), 6.21 (dd, *J* = 10.0, 2.2 Hz, 1H), 5.95 (dt, *J* = 11.0, 3.0 Hz, 1H), 4.23–4.13 (m, 1H), 3.71 (d, *J* = 10.1 Hz, 1H), 3.09 (s, 3H), 2.44–2.31 (m, 1H), 2.10–1.97 (m, 2H), 1.96–1.89 (m, 1H), 1.46–1.35 (m, 1H); ^{13}C NMR (100 MHz, $CDCl_3$) δ (ppm) 153.8, 149.1, 139.2, 130.6, 128.9, 128.5, 127.7, 124.9, 124.8, 124.5, 122.9, 122.1, 121.5, 111.3, 63.7, 48.2, 42.4, 41.6, 26.3, 24.6; HRMS (ESI-TOF) m/z: $[M + Na]^+$ Calcd for $C_{22}H_{21}NO_3SNa^+$ 402.1134; Found 402.1136.

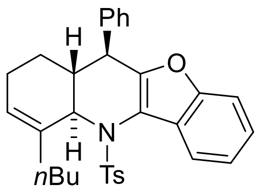

Synthesis of 5j: To an oven dried 10 mL Schlenk tube equipped with a stirring bar were added *N*-((*E*)-2-((*Z*)-benzylidene)benzofuran-3(2*H*)-ylidene)-4-methylbenzenesulfonamide **2a** (37.5 mg, 0.100 mmol, 1.0 equiv), $Pd_2(dba)_3$ (4.6 mg, 0.0050 mmol, 5 mol%) and **L10** (6.4 mg, 0.020 mmol, 20 mol%). Then degassed dry toluene (1.0 mL) and 2-butylcyclohexa-1,3-diene **1b** (27.2 mg, 0.200 mmol, 2.0 equiv) were added via syringe sequentially under argon atmosphere. The mixture was stirred at 80 ℃ for 48 h. After complete consumption of **2a**, the mixture was directly purified by flash chromatography on silica gel (EtOAc/petroleum ether = 1/100) to afford product **5j**: 10.3 mg (0.0201 mmol), as a white solid, 20% yield, as a white solid; mp 181–183 ℃; $[\alpha]_D^{25}$ = +62.9 (*c* = 0.39 in $CHCl_3$); >19:1 dr; 53% ee, determined by HPLC analysis (Daicel Chiralpak IG, *n*-Hexane/*i*-PrOH = 90/10, flow rate =1.0 mL/min, λ = 256 nm) t_R = 9.90 min (major), t_R = 11.98 min (minor); ^1H NMR (400 MHz, $CDCl_3$) δ (ppm) 8.12 (d, *J* = 7.5 Hz, 1H), 7.51 (d, *J* = 8.2 Hz, 2H), 7.37–7.27 (m, 3H), 7.25–7.17 (m, 3H), 7.15–7.07 (m, 2H), 6.42 (dd, *J* = 7.1, 1.7 Hz, 2H), 5.86–5.78 (m, 1H), 4.20–4.09 (m, 1H), 3.60 (d, *J* = 10.6 Hz, 1H), 2.71–2.59 (m, 1H), 2.51–2.44 (m, 1H), 2.44 (s, 3H), 2.12–1.91 (m, 3H), 1.57–1.46 (m, 2H), 1.43–1.33 (m, 3H), 1.20–1.08 (m, 1H), 0.92 (t, *J* = 7.2 Hz, 3H); ^{13}C NMR (100 MHz, $CDCl_3$) δ (ppm) 153.9, 150.8, 143.9, 139.0, 137.7, 136.2, 129.6, 129.3, 128.3, 128.2, 127.3, 125.6, 124.8, 124.4, 123.1, 123.0, 122.1, 111.3, 68.8, 49.6, 39.8, 34.1, 31.3, 26.9, 24.7, 22.6, 21.6, 14.1; HRMS (ESI-TOF) m/z: $[M + Na]^+$ Calcd for $C_{32}H_{33}NO_3SNa^+$ 534.2073; Found 534.2074.

5. More substrate exploration

5.1 Exploration of more cyclic dienes and polyenes

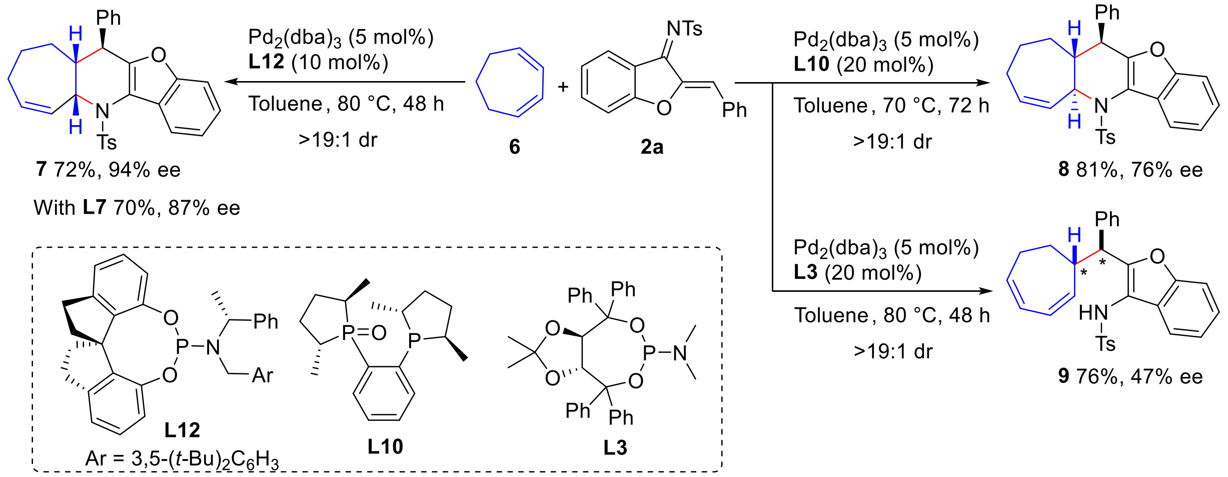

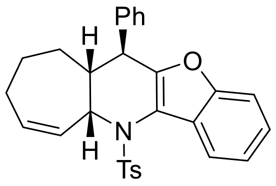

Synthesis of 7: To an oven dried 10 mL Schlenk tube equipped with a stirring bar were added *N*-((*E*)-2-((*Z*)-benzylidene)benzofuran-3(2*H*)-ylidene)-4-methylbenzenesulfonamide **2a** (37.5 mg, 0.100 mmol, 1.0 equiv), $Pd_2(dba)_3$ (4.6 mg, 0.0050 mmol, 5 mol%) and **L12** (6.0 mg, 0.010 mmol, 10 mol%). The tube was capped, evacuated and back-filled with argon for five times. Then degassed dry toluene (1.0 mL) and 1,3-cycloheptadiene **6** (22.0 μL, 0.200 mmol, 2.0 equiv) were added via syringe sequentially under argon atmosphere. The mixture was stirred at 60 ℃ for 48 h. After complete consumption of **2a**, the mixture was directly purified by flash chromatography on silica gel (EtOAc/petroleum ether = 1/50) to afford product **7**: 34.0 mg (0.0724 mmol), as a white solid, 72% yield; mp 181–183 ℃; $[\alpha]_D^{25}$ = +20.0 (*c* = 0.41 in $CHCl_3$); >19:1 dr; 94% ee, determined by HPLC analysis (Daicel Chiralpak IG, *n*-Hexane/*i*-PrOH = 90/10, flow rate =1.0 mL/min, λ = 254 nm) t_R = 7.38 min (major), t_R = 10.04 min (minor); ^1H NMR (400 MHz, $CDCl_3$) δ (ppm) 8.25–8.19 (m, 1H), 7.68–7.56 (m, 2H), 7.30–7.26 (m, 4H), 7.20–7.16 (m, 1H), 7.15–7.09 (m, 2H), 6.56–6.50 (m, 2H), 5.90–5.77(m, 1H), 5.42–5.38 (m, 1H), 5.18–5.16 (m, 1H), 3.95 (d, *J* = 11.4 Hz, 1H), 2.43 (s, 3H), 2.31–2.18 (m, 1H), 2.09 (d, *J* = 18.6 Hz, 1H), 1.80–1.64 (m, 1H), 1.53–1.42 (m, 4H); ^{13}C NMR (100 MHz, $CDCl_3$) δ (ppm) 154.3, 150.0, 144.1, 143.3, 138.9, 135.1, 132.6, 132.3, 130.5, 129.8, 129.6, 129.0, 128.4, 128.3, 128.3, 128.2, 127.9, 127.2, 125.4, 124.6, 124.5, 123.0, 122.4, 117. 9, 111.2, 62.7, 40.6, 39.7, 29.4, 28.8, 21.5, 21.0; HRMS (ESI-TOF) m/z: $[M + Na]^+$ Calcd for $C_{29}H_{27}NO_3SNa^+$ 492.1604; Found 492.1613.

*Note: 7 was obtained in 70 yield and 87% ee by using $Pd_2(dba)_3$ in combination with **L7**, and the configuration of 7 was assigned by analogy with products **4**.*

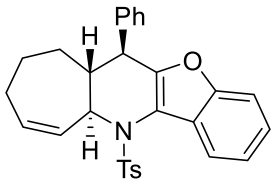

Synthesis of 8: To an oven dried 10 mL Schlenk tube equipped with a stirring bar were added *N*-((*E*)-2-((*Z*)-benzylidene)benzofuran-3(2*H*)-ylidene)-4-methylbenzenesulfonamide **2a** (37.5 mg, 0.100 mmol, 1.0 equiv), $Pd_2(dba)_3$ (4.6 mg, 0.0050 mmol, 5 mol%) and **L10** (6.4 mg, 0.020 mmol, 20 mol%). The tube was capped, evacuated and back-filled with argon for five times. Then degassed dry toluene (1.0 mL) and 1,3-cycloheptadiene **6** (22.0 μL, 0.200 mmol, 2.0 equiv) were added via syringe sequentially under argon atmosphere. The mixture was stirred at 70 ℃ for 72 h. After complete consumption of **2a**, the mixture was directly purified by flash chromatography on silica gel (EtOAc/petroleum ether = 1/50) to afford product **8**: 37.8 mg (0.0805 mmol), as a white solid, 81%

yield; mp 199–201 ℃; $[\alpha]_D^{25}$ = –16.5 (*c* = 0.55 in $CHCl_3$); >19:1 dr; 76% ee, determined by HPLC analysis (Daicel Chiralpak IG, *n*-Hexane/*i*-PrOH = 90/10, flow rate =1.0 mL/min, λ = 254 nm) t_R = 11.72 min (major), t_R = 14.73 min (minor); ^{1}H NMR (400 MHz, $CDCl_3$) δ (ppm) 8.03–7.95 (m, 1H), 7.54–7.48 (m, 2H), 7.37–7.27 (m, 7H), 7.26–7.21 (m, 1H), 6.94–6.83 (m, 2H), 6.09 (ddd, *J* = 11.3, 4.0, 1.5 Hz, 1H), 5.86–5.76 (m, 1H), 4.58–4.47 (m, 1H), 2.48 (s, 3H), 2.39 (d, *J* = 11.0 Hz, 1H), 2.23–2.10 (m, 2H), 2.09–1.99 (m, 1H), 1.71–1.59 (m, 1H), 1.54–1.46 (m, 1H), 1.27–1.20 (m, 1H), 1.16–1.07 (m, 1H); ^{13}C NMR (100 MHz, $CDCl_3$) δ (ppm) 153.8, 152.1, 144.1, 136.4, 133.7, 130.1, 129.5, 129.1, 128.6, 127.9, 127.6, 125.4, 123.9, 123.2, 121.2, 117.6, 111.4, 63.1, 50.7, 45.9, 33.3, 27.4, 25.0, 21.6; HRMS (ESI-TOF) m/z: $[M + Na]^+$ Calcd for $C_{29}H_{27}NO_3SNa^+$ 492.1604; Found 492.1604. *The configuration of **8** was assigned by analogy with products **5**.*

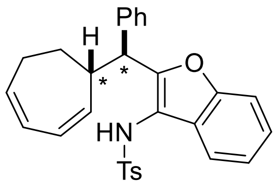

Synthesis of 9: To an oven dried 10 mL Schlenk tube equipped with a stirring bar were added *N*-((*E*)-2-((*Z*)-benzylidene)benzofuran-3(2*H*)-ylidene)-4-methylbenzenesulfonamide **2a** (37.5 mg, 0.100 mmol, 1.0 equiv), $Pd_2(dba)_3$ (4.6 mg, 0.0050 mmol, 5 mol%) and **L3** (10.8 mg, 0.0200 mmol, 20 mol%). The tube was capped, evacuated and back-filled with argon for five times. Then degassed dry toluene (1.0 mL) and 1,3-cycloheptadiene **6** (22.0 μL, 0.200 mmol, 2.0 equiv) were added via syringe sequentially under argon atmosphere. The mixture was stirred at 80 ℃ for 36 h. After complete consumption of **2a**, the mixture was directly purified by flash chromatography on silica gel (EtOAc/petroleum ether = 1/50) to afford product **9**: 34.0 mg (0.0725 mmol), as a colorless oil, 73% yield; $[\alpha]_D^{25}$ = –74.0 (*c* = 0.34 in $CHCl_3$); >19:1 dr; 47% ee, determined by HPLC analysis (Daicel Chiralpak IE, *n*-Hexane/*i*-PrOH = 95/5, flow rate =1.0 mL/min, λ = 254 nm) t_R = 8.87 min (minor), t_R = 10.0 min (major); ^{1}H NMR (400 MHz, $CDCl_3$) δ (ppm) 7.65 (d, *J* = 8.1 Hz, 2H), 7.38 (d, *J* = 8.3 Hz, 1H), 7.25–7.17 (m, 7H), 7.16–7.08 (m, 3H), 6.11–6.01 (m, 1H), 5.93–5.82 (m, 2H), 5.69 (dd, *J* = 11.6, 6.5 Hz, 1H), 5.45 (dd, *J* = 11.6, 5.3 Hz, 1H), 3.88 (d, *J* = 11.4 Hz, 1H), 3.33–3.27 (m, 1H), 2.41 (s, 3H), 2.23–2.14 (m, 2H), 1.68–1.62 (m, 1H), 1.55–1.45 (m, 1H); ^{13}C NMR (100 MHz, $CDCl_3$) δ (ppm) 156.0, 153.3, 143.9, 138.7, 136.8, 135.1, 134.9, 129.7, 128.6, 128.5, 127.6, 127.0, 126.0, 125.1, 124.5, 124.2, 123.0, 119.4, 113.1, 111.4, 45.9, 43.7, 29.0, 28.2, 21.6; HRMS (ESI-TOF) m/z: $[M + Na]^+$ Calcd for $C_{29}H_{27}NO_3SNa^+$ 492.1604; Found 492.1612.

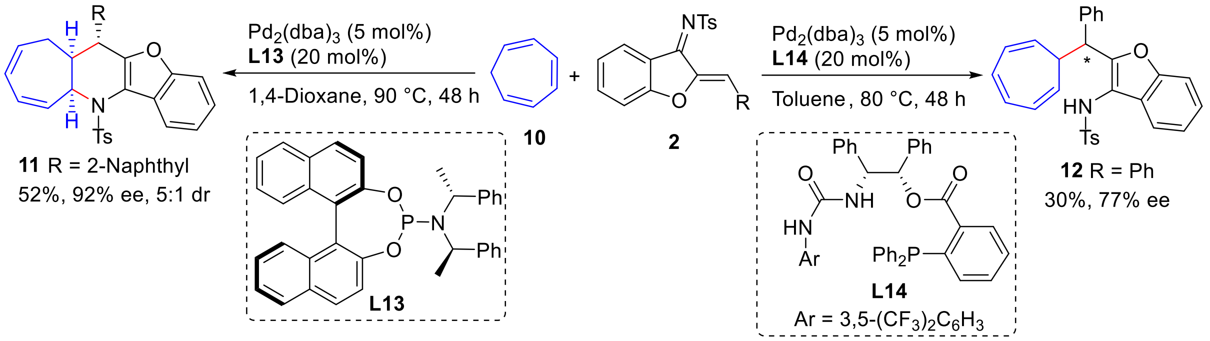

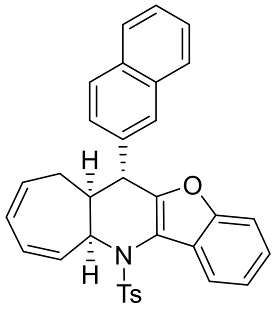

Synthesis of 11: To an oven dried 10 mL Schlenk tube equipped with a stirring bar were added 4-methyl-*N*-((2*Z*,3*E*)-2-(naphthalen-2-ylmethylene)-benzofuran-3(2*H*)-ylidene)benzenesulfonamide **2e** (85.0 mg, 0.200 mmol, 1.0 equiv), $Pd_2(dba)_3$ (9.2 mg, 0.010 mmol, 5 mol%) and **L13** (21.6 mg, 0.0400 mmol, 20 mol%). The tube was capped, evacuated and back-filled with argon for five times. Then degassed dry toluene (1.0 mL) and cycloheptatriene **10** (32.0 μL, 0.200 mmol, 2.0 equiv) were added via syringe sequentially under argon atmosphere. The mixture was stirred at 90 ℃ for 48 h. After complete consumption of **2e**, the mixture was directly purified by flash chromatography on silica gel (EtOAc/petroleum ether = 1/50) to afford product **11**: 52.2 mg (0.0504 mmol), as a white solid, 50% yield; mp 241–242 ℃; $[\alpha]_D^{25}$ = –179.1 (*c* = 0.33 in $CHCl_3$); 5:1 dr; 92% ee, determined by HPLC analysis (Daicel Chiralpak IC, *n*-Hexane/*i*-PrOH = 90/10, flow rate =1.0 mL/min, λ = 254 nm) t_R = 10.77 min (minor), t_R = 19.23 min (major); Pure **11** for NMR analysis was obtained after recrystallisation. ^1H NMR (400 MHz, $CDCl_3$) δ (ppm) 8.27–8.21 (m, 1H), 7.80–7.75 (m, 1H), 7.69–7.61 (m, 3H), 7.57 (d, *J* = 8.4 Hz, 1H), 7.48–7.42 (m, 2H), 7.35–7.30 (m, 3H), 7.28–7.26 (m, 1H), 7.23–7.21 (m, 1H), 6.47 (dd, *J* = 8.5, 1.8 Hz, 1H), 5.96–5.86 (m, 1H), 5.79 (ddd, *J* = 12.1, 7.1, 2.9 Hz, 1H), 5.71–5.64 (m, 1H), 5.58 (d, *J* = 12.3 Hz, 1H), 5.34–5.26 (m, 1H), 4.20 (d, *J* = 11.4 Hz, 1H), 2.45 (s, 3H), 2.34–2.25 (m, 1H), 2.13–2.05 (m, 1H), 1.99–1.86 (m, 1H); ^{13}C NMR (100 MHz, $CDCl_3$) δ (ppm) 154.4, 150.4, 144.3, 136.3, 134.9, 133.3, 132.8, 132.7, 130.7, 130.0, 128.1, 128.0, 127.6, 127.51, 127.48, 126.2, 125.9, 125.7, 125.6, 124.6, 124.5, 123.1, 122.4, 117.4, 111.3, 62.7, 41.2, 38.5, 29.0, 21.7; HRMS (ESI-TOF) m/z: [M + Na]+ Calcd for $C_{33}H_{27}NO_3SNa^+$ 540.1604; Found 540.1602.

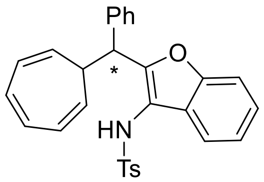

Synthesis of 12: To an oven dried 10 mL Schlenk tube equipped with a stirring bar were added *N*-((*E*)-2-((*Z*)-benzylidene)benzofuran-3(2*H*)-ylidene)-4-methylbenzenesulfonamide **2a** (37.5 mg, 0.100 mmol, 1.0 equiv), $Pd_2(dba)_3$

(4.6 mg, 0.0050 mmol, 5 mol%) and **L14** (15.2 mg, 0.0200 mmol, 20 mol%). The tube was capped, evacuated and back-filled with argon for five times. Then degassed dry toluene (1.0 mL) and cycloheptatriene **10** (16.0 μL, 0.200 mmol, 2.0 equiv) were added via syringe sequentially under argon atmosphere. The mixture was stirred at 80 ℃ for 48 h. After complete consumption of **2a**, the mixture was directly purified by flash chromatography on silica gel (EtOAc/petroleum ether = 1/50) to afford **12**: 14.0 mg (0.0299 mmol), as a yellow oil, 30% yield; $[\alpha]_D^{25}$ = –32.2 (*c* = 0.34 in $CHCl_3$); 77% ee, determined by HPLC analysis (Daicel Chiralpak IH, *n*-Hexane/*i*-PrOH = 90/10, flow rate =1.0 mL/min, λ = 254 nm) t_R = 8.39 min (minor), t_R = 9.77 min (major); ^{1}H NMR (400 MHz, $CDCl_3$) δ (ppm) 7.68–7.62 (m, 2H), 7.37–7.33 (m, 1H), 7.25–7.21 (m, 4H), 7.19–7.15 (m, 3H), 7.05–7.00 (m, 1H), 6.97–6.91 (m, 1H), 6.77–6.63 (m, 2H), 6.16–6.08 (m, 2H), 5.95 (s, 1H), 5.21 (dd, *J* = 9.8, 6.0 Hz, 1H), 5.07 (dd, *J* = 9.7, 6.1 Hz, 1H), 4.39 (d, *J* = 11.8 Hz, 1H), 2.79–2.68 (m, 1H), 2.37 (s, 3H); ^{13}C NMR (100 MHz, $CDCl_3$) δ (ppm) 156.3, 153.2, 144.1, 139.2, 136.6, 131.1, 130.6, 129.8, 128.7, 128.5, 127.6, 127.1, 125.7, 125.2, 125.1, 124.5, 124.2, 122.9, 118.8, 113.5, 111.5, 43.6, 42.3, 21.6; HRMS (ESI-ToF) m/z: $[M + Na]^+$ Calcd for $C_{29}H_{25}NO_3SNa^+$ 490.1447; Found 490.1443.

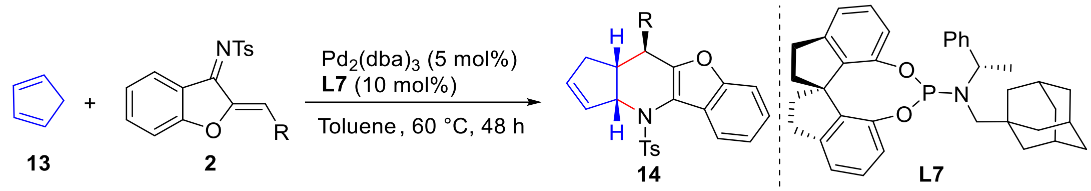

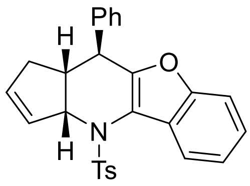

Synthesis of 14a: To an oven dried 10 mL Schlenk tube equipped with a stirring bar were added *N*-((*E*)-2-((*Z*)-benzylidene)benzofuran-3(2*H*)-ylidene)-4-methylbenzenesulfonamide **2a** (37.5 mg, 0.100 mmol, 1.0 equiv), $Pd_2(dba)_3$ (4.6 mg, 0.0050 mmol, 5 mol%) and **L7** (5.5 mg, 0.010 mmol, 10 mol%). The tube was capped, evacuated and back-filled with argon for five times. Then degassed dry toluene (1.0 mL) and cyclopentadiene **13** (16.0 μL, 0.200 mmol, 2.0 equiv) were added via syringe sequentially under argon atmosphere. The mixture was stirred at 60 ℃ for 48 h. After complete consumption of **2a**, the mixture was directly purified by flash chromatography on silica gel (petroleum ether/dichloromethane = 3/1) to afford product **14a**: 29.8 mg (0.0675 mmol), as a white solid, 68% yield; mp 160–162 ℃; $[\alpha]_D^{25}$ = +278.3 (*c* = 0.46 in $CHCl_3$); 3:1 dr; 91% ee, determined by HPLC analysis (Daicel Chiralpak ID, *n*-Hexane/*i*-PrOH = 80/20, flow rate =1.0 mL/min, λ = 254 nm) t_R = 14.88 min (major), t_R = 18.36 min (minor); ^{1}H NMR (400 MHz, $CDCl_3$) δ (ppm) 8.22 (d, *J* = 7.7 Hz, 1H), 7.56 (d, *J* = 8.0 Hz, 2H), 7.36–7.22 (m, 7H), 7.21–7.16 (m, 1H), 7.13–7.06 (m, 2H), 6.50–6.38

(m, 2H), 5.93–5.80 (m, 2H), 5.35–5.24 (m, 1H), 3.55 (d, *J* = 7.1 Hz, 1H), 2.45 (s, 3H), 2.43–2.37 (m, 1H), 2.30 (dd, *J* = 15.7, 2.8 Hz, 1H), 2.22–2.12 (m, 1H); ^{13}C NMR (100 MHz, $CDCl_3$) δ (ppm) 153.6, 150.4, 144.1, 140.6, 134.0, 133.7, 130.3, 129.9, 128.4, 128.3, 128.2, 127.9, 127.1, 124.7, 124.5, 123.0, 122.6, 111.1, 66.2, 43.6, 43.5, 38.4, 21.6; HRMS (ESI-TOF) m/z: $[M + Na]^+$ Calcd for $C_{27}H_{23}NO_3SNa^+$ 464.1291; Found 464.1297.

Note: Racemic ***14a–14c*** *were obtained by using $Pd_2(dba)_3$ in combination with racemic* ***L7****.*

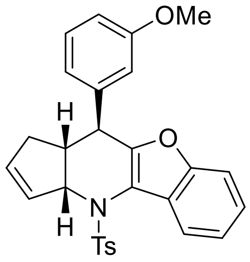

Synthesis of 14b: To an oven dried 10 mL Schlenk tube equipped with a stirring bar were added *N*-((*E*)-2-((*Z*)-3-methoxybenzylidene)benzofuran-3(2*H*)-ylidene)-4-methylbenzenesulfonamide **2b** (40.5 mg, 0.100 mmol, 1.0 equiv), $Pd_2(dba)_3$ (4.6 mg, 0.0050 mmol, 5 mol%) and **L7** (5.5 mg, 0.010 mmol, 10 mol%). The tube was capped, evacuated and back-filled with argon for five times. Then degassed dry toluene (1.0 mL) and cyclopentadiene **13** (16.0 μL, 0.200 mmol, 2.0 equiv) were added via syringe sequentially under argon atmosphere. The mixture was stirred at 60 ℃ for 48 h. After complete consumption of **2b**, the mixture was directly purified by flash chromatography on silica gel (petroleum ether/ dichloromethane = 3/1) to afford product **14b**: 31.9 mg, as a white solid (0.0676 mmol), 68% yield; mp 151−153 ℃; $[\alpha]_D^{25}$ = +257.0 (*c* = 0.46 in $CHCl_3$); 5:1 dr; 90% ee, determined by HPLC analysis (Daicel Chiralpak ID, *n*-Hexane/*i*-PrOH = 80/20, flow rate =1.0 mL/min, λ = 254 nm) t_R = 13.72 min (major), t_R = 15.62 min (minor); ^{1}H NMR (400 MHz, $CDCl_3$) δ (ppm) 8.31–8.16 (m, 1H), 7.60–7.48 (m, 2H), 7.34–7.21 (m, 6H), 7.05–6.99 (m, 1H), 6.72 (ddd, *J* = 8.3, 2.6, 0.9 Hz, 1H), 6.21–6.12 (m, 1H), 6.01 (dt, *J* = 7.8, 1.2 Hz, 1H), 5.91–5.79 (m, 2H), 5.37–5.13 (m, 1H), 3.75 (s, 3H), 3.52 (d, *J* = 7.3 Hz, 1H), 2.42 (s, 3H), 2.46–2.36 (m, 1H), 2.29 (dd, *J* = 15.6, 3.0 Hz, 1H), 2.18–2.10 (m, 1H); ^{13}C NMR (100 MHz, $CDCl_3$) δ (ppm) 153.6, 150.2, 144.3, 142.0, 134.0, 133.5, 130.3, 129.9, 129.4, 128.1, 124.7, 124.5, 122.9, 122.6, 120.3, 117.5, 115.0, 111.2, 111.1, 66.0, 55.1, 43.4, 43.2, 38.3, 21.6; HRMS (ESI-TOF) m/z: $[M + Na]^+$ Calcd for $C_{28}H_{25}NO_4SNa^+$ 494.1397; Found 494.1395.

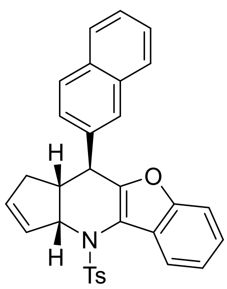

Synthesis of 14c: To an oven dried 10 mL Schlenk tube equipped with a stirring bar were added 4-methyl-*N*-((2*Z*,3*E*)-2-(naphthalen-2-ylmethylene)-benzo furan-3(2*H*)-ylidene)benzenesulfonamide **2e** (42.6 mg, 0.100 mmol, 1.0 equiv), $Pd_2(dba)_3$ (4.6 mg, 0.0050 mmol, 5 mol%) and **L7** (5.5 mg, 0.010 mmol, 10 mol%). The tube was capped, evacuated and back-filled with argon for five times.

Then degassed dry toluene (1.0 mL) and cyclopentadiene **13** (16.0 μL, 0.200 mmol, 2.0 equiv) were added via syringe sequentially under argon atmosphere. The mixture was stirred at 60 ℃ for 48 h. After complete consumption of **2e**, the mixture was directly purified by flash chromatography on silica gel (petroleum ether/dichloromethane = 3/1) to afford product **14c**: 25.4 mg (0.0517 mmol), as a white solid, 52% yield; mp 175–177 ℃; $[\alpha]_D^{25}$ = +345.7 (*c* = 0.21 in $CHCl_3$); 6:1 dr; 87% ee, determined by HPLC analysis (Daicel Chiralpak ID, *n*-Hexane/*i*-PrOH = 80/20, flow rate =1.0 mL/min, λ = 254 nm) t_R = 14.88 min (major), t_R = 18.83 min (minor); ^{1}H NMR (400 MHz, CDCl3) δ (ppm) 8.24 (dd, *J* = 7.8, 1.2 Hz, 1H), 7.79–7.73 (m, 1H), 7.70–7.66 (m, 1H), 7.62–7.57 (m, 2H), 7.53–7.49 (m, 1H), 7.48–7.43 (m, 2H), 7.35–7.29 (m, 2H), 7.27–7.19 (m, 4H), 6.27 (dd, *J* = 8.5, 1.8 Hz, 1H), 5.94–5.84 (m, 2H), 5.38–5.29 (m, 1H), 3.74 (d, *J* = 7.1 Hz, 1H), 2.50–2.41 (m, 1H), 2.39 (s, 4H), 2.29–2.24 (m, 1H); ^{13}C NMR (100 MHz, $CDCl_3$) δ (ppm) 153.7, 150.3, 144.2, 137.8, 134.0, 133.7, 133.2, 132.5, 130.4, 129.9, 128.21, 128.17, 127.58, 127.55, 127.0, 126.3, 125.9, 125.3, 124.7, 124.6, 123.0, 122.7, 117.7, 111.1, 66.1, 43.6, 43.2, 38.4, 21.6; HRMS (ESI-TOF) m/z: $[M + Na]^+$ Calcd for $C_{31}H_{25}NO_3SNa^+$ 514.1447; Found 514.1443.

5.2 Exploration of linear 1,3-dienes

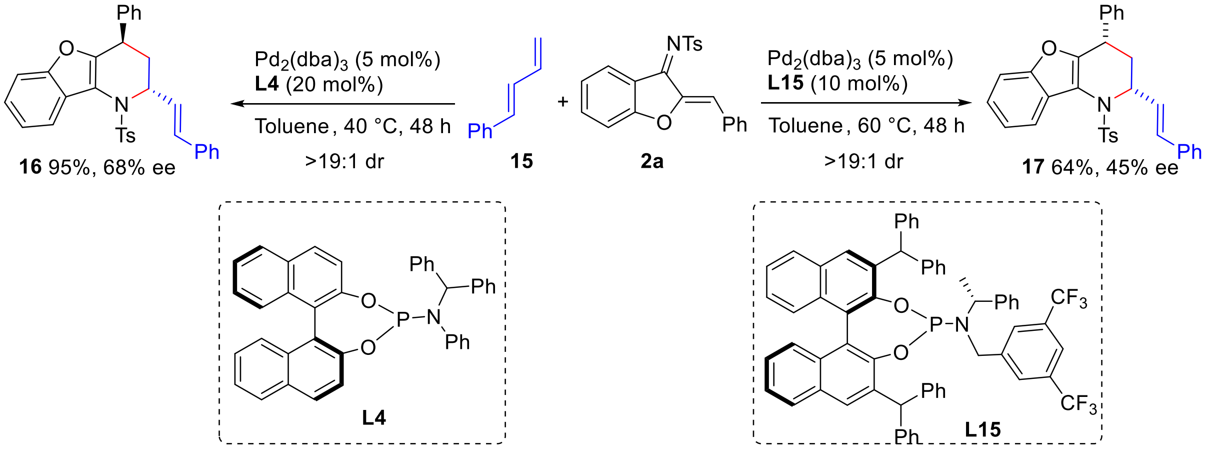

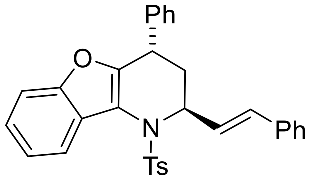

Synthesis of 16: To an oven-dried 10 mL tube equipped with a septum and a stirring bar were charged with $Pd_2(dba)_3$ (4.6 mg, 0.0050 mmol, 5 mol%) and **L4** (11.4 mg, 0.0200 mmol, 10 mol%), the tube was evacuated and backfilled with argon for three times, then degassed dry toluene (1.0 mL) was added via syringe. The mixture was stirred for 30 min at room temperature before transferred to another Schlenk tube containing (*E*)-buta-1,3-dien-1-ylbenzene **15** (26.0 mg, 0.200 mmol, 2.0 equiv) and *N*-((*E*)-2-((*Z*)-benzylidene)benzofuran-3(2*H*)-ylidene)-4-methylbenzenesulfonamide **2a** (37.5

mg, 0.100 mmol, 1.0 equiv) under argon atmosphere. The resultant mixture was stirred at 40 ℃ for 48 h. After completion, the mixture was purified by flash chromatography on silica gel (petroleum ether/DCM = 3/1) gave the product **16**: 48.2 mg (0.0953 mmol), as a white solid, 95% yield; mp = 255–257 ℃; >19:1 dr; $[\alpha]_D^{25}$ = –95.1 (*c* = 0.23 in $CHCl_3$); 68% ee, determined by HPLC analysis [Chiralpak column IF *n*-Hexane/*i*-PrOH = 80/20, flow rate: 1.0 mL/min, 254 nm, t_R (major) = 9.81 min, t_R (minor) = 13.84 min]; ^{1}H NMR (400 MHz, $CDCl_3$): δ (ppm) 8.29 (d, *J* = 7.8 Hz, 1H), 7.66–7.57 (m, 2H), 7.38–7.26 (m, 7H), 7.25–7.14 (m, 6H), 6.74–6.57 (m, 3H), 6.10 (dd, *J* = 15.9, 4.8 Hz, 1H), 5.20–5.11 (m, 1H), 3.96 (dd, *J* = 11.6, 7.1 Hz, 1H), 2.41 (s, 3H), 2.04 (ddd, *J* = 14.1, 7.1, 2.5 Hz, 1H), 1.54–1.47 (m, 1H); ^{13}C NMR (100 MHz, $CDCl_3$): δ (ppm) 154.0, 148.5, 144.2, 140.1, 136.1, 134.4, 132.1, 129.9, 128.51, 128.49, 128.0, 127.8, 127.6, 127.3, 126.5, 125.9, 124.7, 124.5, 123.1, 122.6, 118.1, 111.2, 58.4, 37.2, 34.5, 21.6; HRMS (ESI-TOF) m/z: $[M + Na]^+$ Calcd for $C_{32}H_{27}NO_3SNa^+$ 528.1609; Found 528.1607.

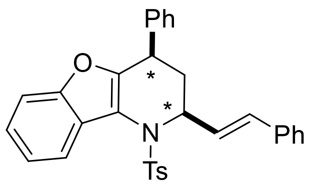

Synthesis of 17*:* To an oven dried 10 mL Schlenk tube equipped with a stirring bar were added *N*-((*E*)-2-((*Z*)-benzylidene)benzofuran-3(2*H*)-ylidene)-4-methylbenzenesulfonamide **2a** (37.5 mg, 0.100 mmol, 1.0 equiv), $Pd_2(dba)_3$ (4.6 mg, 0.0050 mmol, 5 mol%) and **L15** (18.8 mg, 0.0200 mmol, 10 mol%). The tube was capped, evacuated and back-filled with argon for five times. Then degassed dry toluene (1.0 mL) and (*E*)-buta-1,3-dien-1-ylbenzene **15** (26.0 mg, 0.200 mmol, 2.0 equiv) were added via syringe sequentially under argon atmosphere. The mixture was stirred at 60 ℃ for 48 h. After complete consumption of **2a**, the mixture was directly purified by flash chromatography on silica gel (DCM/petroleum ether = 1/3) to afford product **17**: 25.6 mg (0.0506 mmol), as a white solid, 51% yield; mp = 166–169 ℃; $[\alpha]_D^{25}$ = –30.0 (*c* = 0.40 in $CHCl_3$); >19:1 dr; 45% ee, determined by HPLC analysis [Chiralpak column IF *n*-Hexane/*i*-PrOH = 80/20, flow rate: 1.0 mL/min, 254 nm, t_R (major) = 10.68 min, t_R (minor) = 20.86 min]; ^{1}H NMR (400 MHz, $CDCl_3$): δ (ppm) (ppm) 8.33 (dd, *J* = 7.3, 1.6 Hz, 1H), 7.60–7.55 (m, 2H), 7.47–7.38 (m, 2H), 7.37–7.27 (m, 4H), 7.16–7.08 (m, 6H), 6.93–6.86 (m, 2H), 6.83–6.78 (m, 2H), 6.60 (dd, *J* = 15.9, 2.2 Hz, 1H), 5.51 (dd, *J* = 15.8, 3.9 Hz, 1H), 5.09–5.00 (m, 1H), 3.99 (d, *J* = 7.5 Hz, 1H), 2.44 (s, 3H), 2.06–1.97 (m, 1H), 1.91–1.78 (m, 1H); ^{13}C NMR (100 MHz, $CDCl_3$): δ (ppm) 153.8, 147.1, 144.2, 141.4, 136.3, 134.8, 130.1, 129.8, 128.41, 128.39, 128.2, 127.7, 127.6, 127.3, 126.53, 126.48, 124.7, 124.5, 123.1, 122.8, 118.8, 111.2, 56.7, 36.1, 33.2, 21.7; HRMS (ESI-TOF) m/z: $[M + Na]^+$ Calcd for

$C_{32}H_{27}NO_3SNa^+$ 528.1609; Found 528.1609. *Its relative configuration has been determined by X-ray diffraction analysis.*

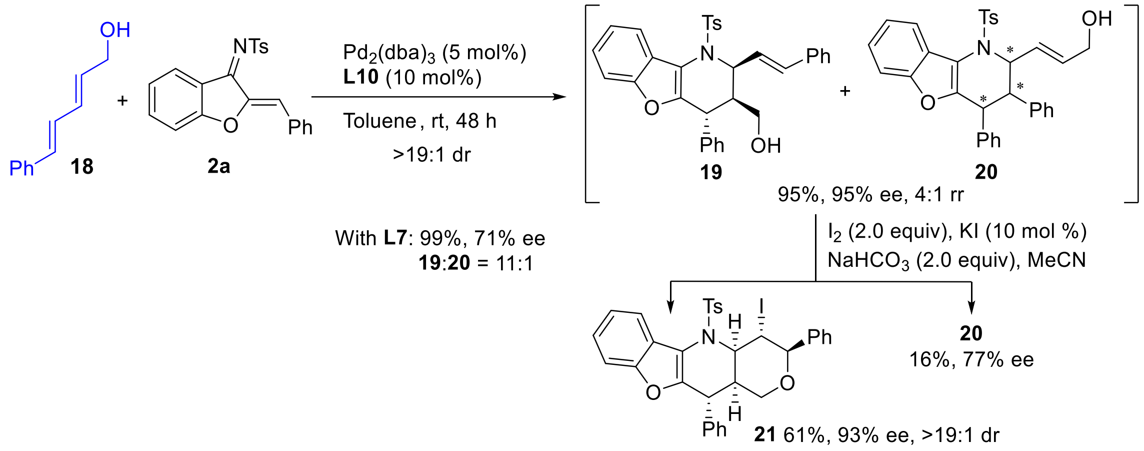

Synthesis of 19: To an oven dried 10 mL Schlenk tube equipped with a stirring bar were added *N*-((*E*)-2-((*Z*)-benzylidene)benzofuran-3(2*H*)-ylidene)-4-methylbenzenesulfonamide **2a** (37.5 mg, 0.100 mmol, 1.0 equiv), (2*E*,4*E*)-5-phenylpenta-2,4-dien-1-ol **18** (32.0 mg, 0.20 mmol, 2.0 equiv), $Pd_2(dba)_3$ (4.6 mg, 0.0050 mmol, 5 mol%) and **L10** (6.4 mg, 0.020 mmol, 20 mol%). The tube was capped, evacuated and back-filled with argon for five times. Then dry distilled toluene (1.0 mL) was added via syringe under argon atmosphere. The mixture was stirred at room temperature for 48 h. After complete consumption of **2a**, the mixture was directly purified by flash chromatography on silica gel (petroleum ether/EA = 50/1) to afford product **19** and **20** as inseparable regioisomers: 50.9 mg (0.0950 mmol), 95% yield, as a white solid, mp = 200–202 ℃; $[\alpha]_D^{25}$ = –206.3 (*c* = 0.42 in $CHCl_3$); 4:1 rr; 95% ee, determined by HPLC analysis [Chiralpak column ID *n*-Hexane/*i*-PrOH = 60/40, flow rate: 1.0 mL/min, 254 nm, t_R (major) = 5.59 min, t_R (minor) = 7.61 min].

19: ^1H NMR (400 MHz, $CDCl_3$) δ (ppm) 8.26 (dd, *J* = 7.3, 1.2 Hz, 1H), 7.74–7.62 (m, 2H), 7.37–7.27 (m, 8H), 7.25–7.18 (m, 3H), 7.18–7.11 (m, 2H), 6.82 (d, *J* = 15.7 Hz, 1H), 6.62–6.51 (m, 2H), 6.06 (dd, *J* = 15.8, 7.7 Hz, 1H), 5.41–5.33 (m, 1H), 3.61 (d, *J* = 11.4 Hz, 1H), 3.52–3.34 (m, 2H), 2.45 (s, 3H), 1.94–1.76 (m, 1H); ^{13}C NMR (100 MHz, $CDCl_3$) δ (ppm) 154.3, 148.3, 144.2, 138.6, 136.1, 135.0, 134.9, 129.9, 128.6, 128.5, 128.18, 128.16, 128.1, 127.6, 126.8, 124.8, 124.3, 123.1, 122.8, 121.5, 117.8, 111.2, 60.9, 60.8, 44.7, 39.9, 21.6; HRMS (ESI-TOF) m/z: $[M + Na]^+$ Calcd for $C_{33}H_{29}NO_3SNa^+$ 558.1710; Found 558.1720.

Synthesis of 21 and recovery of 20: The above inseparable regioisomers **19** and **20** (4:1, 53.6 mg, 0.100 mmol, 1.0 equiv), I_2 (50.6 mg, 0.200 mmol, 2.0 equiv), KI (1.7 mg, 0.010 mmol, 10 mol%) and

$NaHCO_3$ (16.8 mg, 0. 200 mmol, 2.0 equiv) were added into CH_3CN (0.5 mL). The mixture was stirred at 40 ℃ for 2 h under argon atmosphere. After complete consumption of **19**, the mixture was directly purified by flash chromatography on silica gel (petroleum ether/EtOAc = 20/1) to afford products **21** and **20**.

21: 40.3 mg (0.0609 mmol), as a white solid, 61% yield; mp = 169–171 ℃; $[\alpha]_D^{25}$ = –360.0 (*c* = 0.40 in $CHCl_3$); 93% ee, determined by HPLC analysis [Chiralpak column AD *n*-Hexane/*i*-PrOH = 80/20,, flow rate: 1.0 mL/min, 254 nm, t_R (major) = 9.69 min, t_R (minor) = 12.46 min]; ^{1}H NMR (400 MHz, $CDCl_3$) δ (ppm) 8.22–8.18 (m, 1H), 7.65–7.58 (m, 2H), 7.37–7.28 (m, 10H), 7.24–7.19 (m, 1H), 7.19–7.09 (m, 2H), 6.54–6.48 (m, 2H), 4.85 (dd, *J* = 11.3, 4.4 Hz, 1H), 4.69 (d, *J* = 10.6 Hz, 1H), 4.31 (d, *J* = 10.5 Hz, 1H), 4.07 (t, *J* = 10.9 Hz, 1H), 3.85–3.81 (dd, *J* = 12.3, 1.6 Hz, 1H), 3.72 (dd, *J* = 12.3, 2.1 Hz, 1H), 2.46 (s, 3H), 1.96–1.84 (m, 1H); ^{13}C NMR (100 MHz, $CDCl_3$) δ (ppm) 154.5, 148.0, 144.5, 139.2, 138.3, 134.7, 130.1, 129.0, 128.6, 128.5, 128.4, 128.1, 127.7, 127.6, 125.0, 124.3, 123.4, 122.4, 116.7, 111.3, 86.8, 77.3, 77.0, 76.7, 67.6, 64.7, 42.4, 39.1, 32.4, 21.6; HRMS (ESI-TOF) m/z: $[M + Na]^+$ Calcd for $C_{33}H_{28}INO_3SNa^+$ 684.0676; Found 684.0672.

20: 8.7 mg, as a white solid, 16% yield (0.0162 mmol); mp = 130–132 ℃; $[\alpha]_D^{25}$ = +170.7 (*c* = 0.23 in $CHCl_3$); 77% ee, determined by HPLC analysis [Chiralpak column ID *n*-Hexane/*i*-PrOH = 60/40, flow rate: 1.0 mL/min, 254 nm, t_R (major) = 6.31 min, t_R (minor) = 6.83 min]; ^{1}H NMR (400 MHz, $CDCl_3$) δ (ppm) 8.30–8.25 (m, 1H), 7.79–7.73 (m, 2H), 7.39 (d, *J* = 8.1 Hz, 2H), 7.37–7.34 (m, 1H), 7.33–7.31 (m, 2H), 7.24–7.22 (m, 1H), 7.22–7.19 (m, 1H), 7.19–7.13 (m, 1H), 7.09–7.04 (m, 1H), 7.03–6.98 (m, 4H), 6.54–6.48 (m, 2H), 5.65–5.59 (m, 2H), 5.03–4.93 (m, 1H), 4.28 (d, *J* = 11.8 Hz, 1H), 4.06–3.97 (m, 2H), 2.93 (dd, *J* = 11.9, 3.8 Hz, 1H), 2.47 (s, 3H); ^{13}C NMR (100 MHz, $CDCl_3$) δ (ppm) 154.4, 149.0, 144.5, 138.1, 136.8, 134. 9, 134.2, 130.0, 129.0, 128.3, 128.2, 127.3, 127.1, 124.8, 124.3, 123.2, 122.6, 122.0, 111.4, 64.2, 62.6, 48.5, 41.2, 21.6; HRMS (ESI-TOF) m/z: $[M + Na]^+$ Calcd for $C_{33}H_{29}NO_3SNa^+$ 558.1710; Found 558.1716.

5.3 Exploration of linear electron-deficient heterodienes

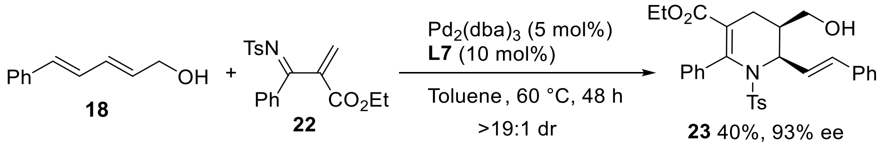

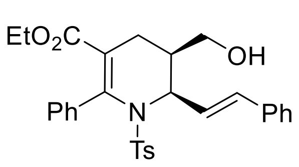

Synthesis of 23: To an oven dried 10 mL Schlenk tube equipped with a stirring bar were added ethyl (*Z*)-2-(phenyl(tosylimino)methyl) acrylate **22** (71.4 mg, 0.200 mmol, 1.0 equiv), 1,3-diene **18** (48.0 mg, 0.300 mmol, 1.5 equiv), $Pd_2(dba)_3$ (9.2 mg, 0.010 mmol, 5 mol%) and **L7** (5.5 mg, 0.020 mmol, 10 mol%). The tube was evacuated and back-filled with argon for five times. Then degassed dry toluene (1.0 mL) was added via syringe under argon atmosphere. The mixture was stirred at 60 ℃ for 48 h. After complete consumption of **22**, the mixture was purified by flash chromatography on silica gel twice (petroleum ether/EtOAc = 10/1, dichloromethane/EtOAc = 50:1) to afford product **23**: 41.0 mg (0.0396 mmol), as a colorless oil, 40% yield; $[\alpha]_D^{25}$ = –5.3 (*c* = 0.57 in $CHCl_3$); 93% ee, determined by HPLC analysis [Chiralpak column ID *n*-Hexane/*i*-PrOH = 80/20, flow rate: 1.0 mL/min, 254 nm, t_R (minor) = 20.05 min, t_R (major) = 22.35 min]; ^{1}H NMR (400 MHz, $CDCl_3$) δ (ppm) 7.43–7.38 (m, 2H), 7.37–7.32 (m, 2H), 7.32–7.16 (m, 5H), 7.13–7.08 (m, 4H), , 6.86 (d, *J* = 15.8 Hz, 1H), 6.14 (dd, *J* = 15.8, 8.6 Hz, 1H), 5.44 (dd, *J* = 8.6, 4.0 Hz, 1H), 3.84 (q, *J* = 7.2 Hz, 2H), 3.70 (dd, *J* = 11.0, 5.3 Hz, 1H), 3.49 (dd, *J* = 11.0, 8.9 Hz, 1H), 2.72 (dd, *J* = 18.5, 6.4 Hz, 1H), 2.36 (s, 3H), 2.31–2.15 (m, 1H), 2.01–1.83 (m, 1H) , 0.83 (t, *J* = 7.2 Hz, 3H); ^{13}C NMR (100 MHz, $CDCl_3$) δ (ppm) 168.4, 143.4, 142.9, 137.8, 136.3, 136.2, 135.3, 129.7, 129.3, 128.7, 128.23, 128.21, 127.1, 127.0, 126.7, 121.6, 117.9, 63.9, 60.4, 57.8, 39.3, 24.9, 21.5, 13.5; HRMS (ESI-TOF) m/z: $[M + Na]^+$ Calcd for $C_{30}H_{31}NO_5SNa^+$ 540.1815; Found 540.1811.

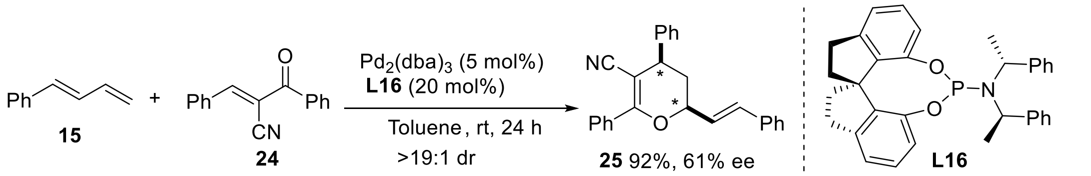

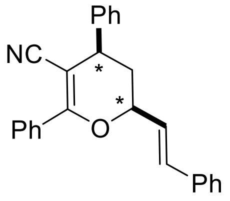

Synthesis of 25: To an oven dried 10 mL Schlenk tube equipped with with a stirring bar were added (*E*)-2-benzoyl-3-phenylacrylonitrile **24** (23.3 mg, 0.100 mmol, 1.0 equiv), (*E*)-buta-1,3-dien-1-ylbenzene **15** (26.0 mg, 0.200 mmol, 2.0 equiv), $Pd_2(dba)_3$ (4.6 mg, 0.0050 mmol, 5 mol%) and **L16** (10.2 mg, 0.0200 mmol, 20 mol%). The tube was evacuated and back-filled with argon for five times. Then degassed dry toluene (1.0 mL) was added via syringe under argon atmosphere. The mixture was stirred at rt for 24 h. After complete consumption of **24**, the mixture was directly purified by flash chromatography on silica gel (DCM/petroleum ether = 1/3) to afford product **25**: 33.4 mg (0.092 mmol), as a white

semi-solid, 92% yield; mp = 82–84 ℃; >19:1 dr; $[\alpha]_D^{25}$ = +15.2 (*c* = 0.32 in $CHCl_3$); 61% ee, determined by HPLC analysis [Chiralpak column IB *n*-Hexane/*i*-PrOH = 80/20, flow rate: 1.0 mL/min, 254 nm, t_R (minor) = 16.68 min, t_R (major) = 17.68 min]; ^{1}H NMR (400 MHz, $CDCl_3$) δ (ppm) 7.76 (dd, *J* = 7.7, 1.9 Hz, 2H), 7.47–7.19 (m, 13H), 6.69 (d, *J* = 16.0 Hz, 1H), 6.23 (dd, *J* = 16.0, 6.5 Hz, 1H), 4.83 (dd, *J* = 11.3, 6.6 Hz, 1H), 3.84 (dd, *J* = 11.5, 6.5 Hz, 1H), 2.37 (ddd, *J* = 14.1, 6.5, 1.9 Hz, 1H), 2.01–1.87 (m, 1H); ^{13}C NMR (100 MHz, $CDCl_3$) δ (ppm) 166.2, 141.2, 135.8, 133.1, 133.0, 130.9, 129.0, 128.7, 128.38, 128.36, 128.3, 127.63, 127.59, 126.6, 126.3, 119.6, 88.4, 78.5, 41.5, 37.8; HRMS (ESI-TOF) m/z: $[M + Na]^+$ Calcd for $C_{26}H_{21}NO_3SNa^+$ 386.1515; Found 386.1515. *Its relative configuration has been determined by X-ray diffraction analysis.*

5.4 Unsuccessful substrates attempts

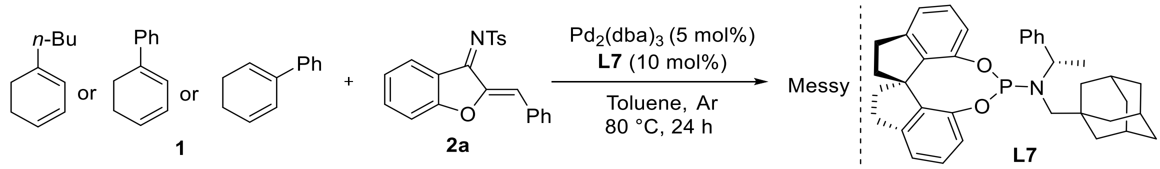

To further expand the substrate scope, some differently substituted 1,3-cyclohexadiene were tested under the optimal conditions. Unfortunately, complex reaction profiles were generally observed as outlined above.

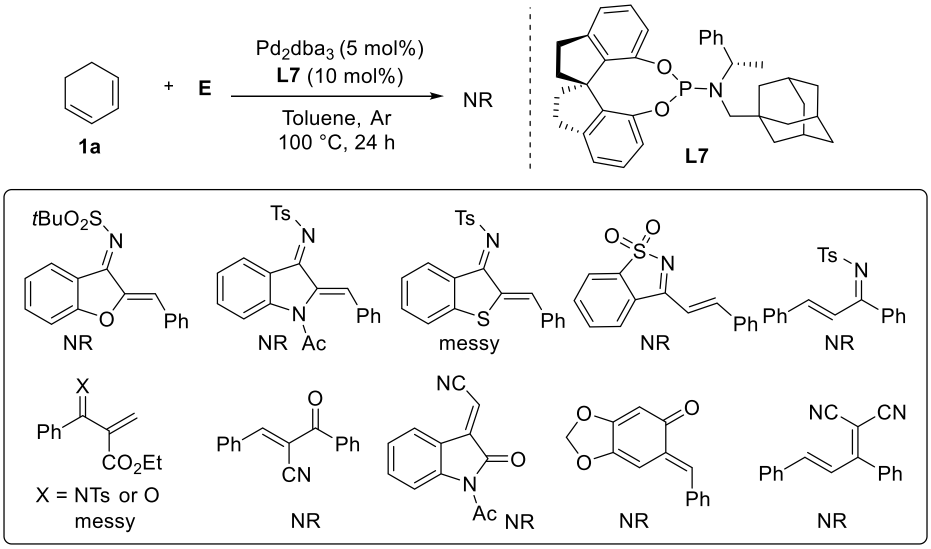

Meanwhile, the above outlined electron-deficient dienes were inert to the reaction under the optimized conditions.

6. Mechanism investigation

6.1 Control experiments

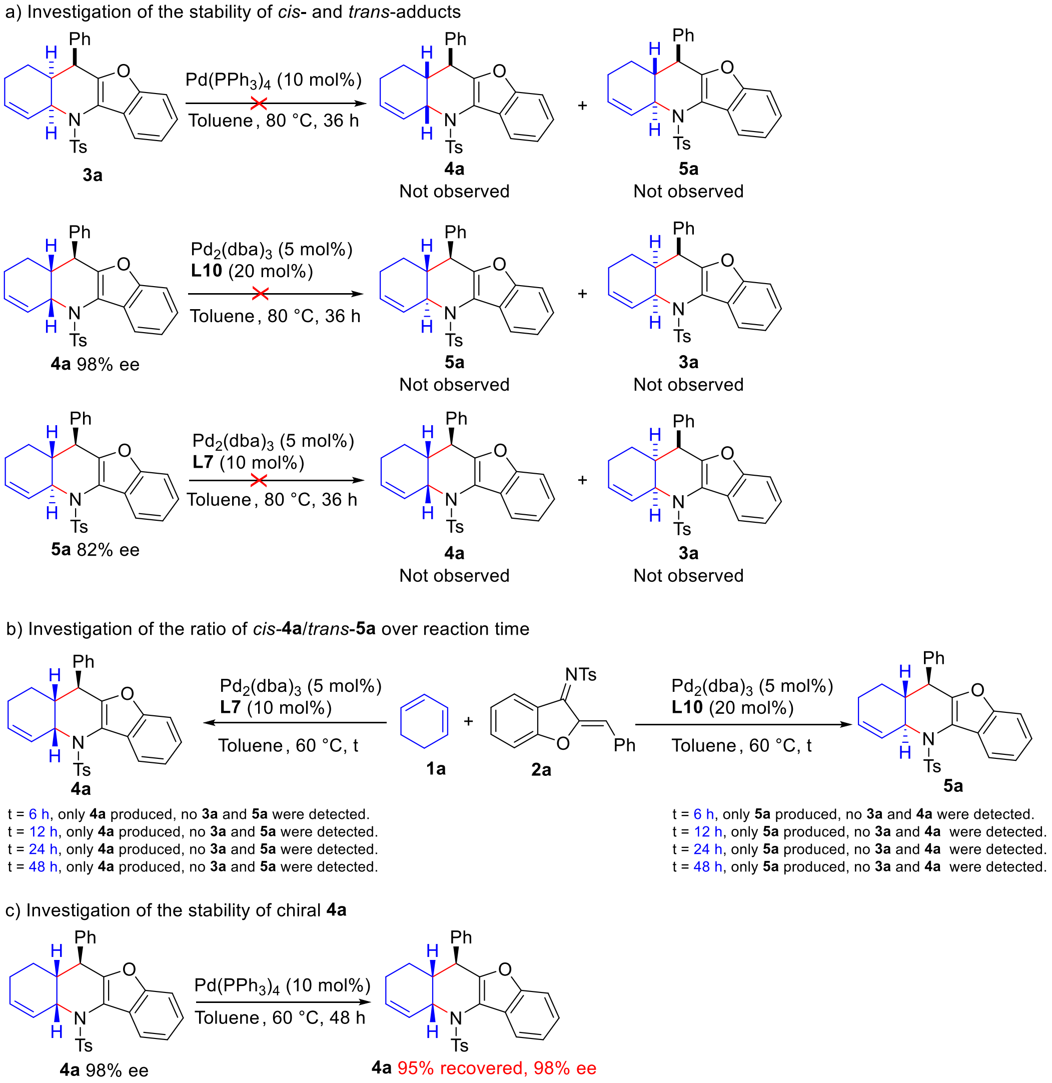

Figure S1 Control experiments to elucidate the stability of products

As demonstrated above, products *endo-cis*-**3a**, *exo-cis*-**4a** and *trans*-**5a** were not interconvertible under diverse catalytic conditions, even at high temperature. The ratio of *cis*-**4a**/*trans*-**5a** was not changed along the reaction process, according to the ^{1}H NMR analysis of the reaction solution in 6 h, 12 h, 24 h, and 48 h, respectively. In addition, the enantioselectivity of chiral **4a** kept unchanged when exposed to $Pd(PPh_3)_4$. *These results well demonstrated that the annulations of cyclic 1,3-dienes and 1-azadienes was irreversible, and the diastereoselectivity was controlled by ligand rather than thermodynamics.*

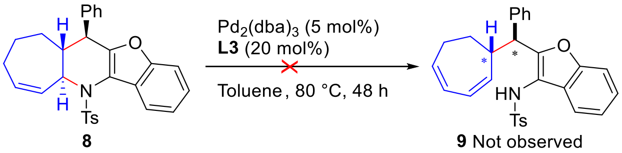

Treatment of **8** with $Pd_2(dba)_3$/**L3** in toluene at 80 ℃ for 48 h did not lead to the formation of **9**, which indicated that **8**, although bearing an allylic amine moiety, was stable and would not undergo ring opening under the catalysis of palladium.

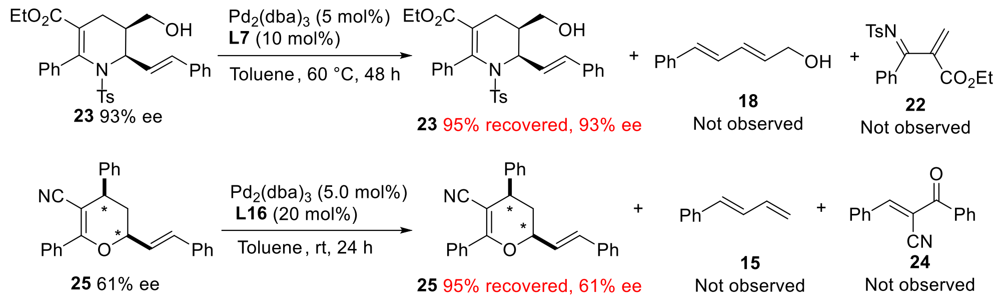

No obvious transformations were observed by treatment of enantioenriched **23** or **25** with $Pd_2(dba)_3$/**L7**. Chiral **23** and **25** with unchanged enantioselectivity were recovered quantitatively. *The results further indicated that the reaction involving linear 1,3-diene was also irreversible.*

6.2 Proposed origin of the divergent diasteroselectivity

The above control experiments confirmed that the formation of both *cis*-**4a** and *trans*-**5a** was a stepwise cascade vinylogous Michael addition/allylic amination process. According to our previous works (*JACS*, **2021**, *143*, 4809; *ACIE*, **2021**, *60*, 26762), vinylogous Michael addition of HOMO-raised η^2-complex **I-A** of Pd^0 with 1,3-cyclohexadiene **1a** to 1-azadiene **2a** from *Si*-face in an outer sphere pattern led to the generation of intermediate **II-A**, as illustrated in Figure S2. Because of the large steric hinderance of **L7**, subsequent intramolecular nucleophilic substitution of allylic Pd^{II} complex with N-Ts moiety would proceed through an outer-sphere manner to furnish *cis*-**4a**. This proposal was further confirmed by utilizing small dimethylamine-substituted ligand **L20**, which delivered *trans*-**5a** predominantly.

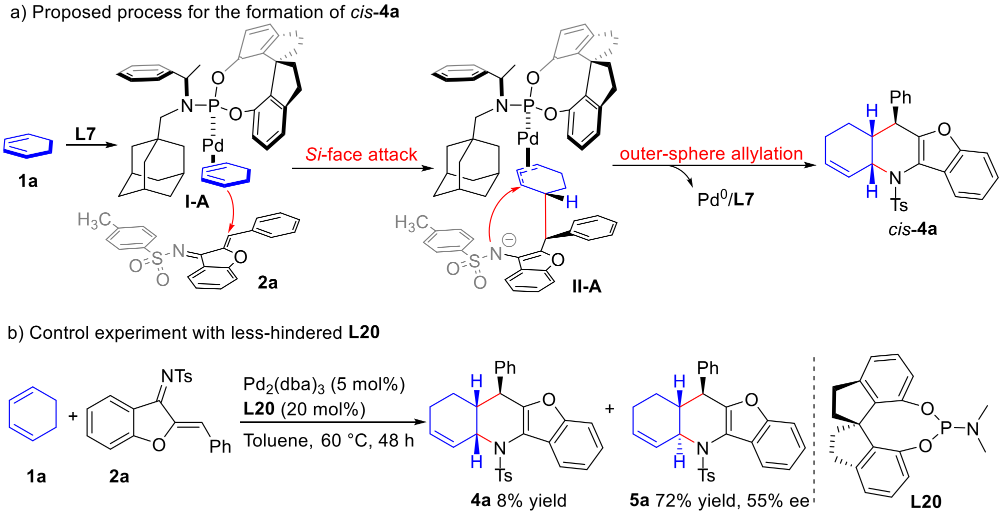

Figure S2 Proposed mechanism for the formation of *cis*-**4a** and control experiment

On the other hand, as illustrated in Figure S3, 1,3-cyclohexadiene **1a** and $Pd_2(dba)_3$ complexed with two moleculear of **L10** to form highly reactive complex **I-B**, which attacked 1-azadiene **2a** from *Si*-face to form species **II-B**. In this case, the carbon-carbon bond rotation and ligand exchange of **II-B** were liable to form more stable electron-neutral **IV-B**, since the less bulky of **L10**. Subsequent reductive elimination deliverd *trans*-**5a**. In conclusion, the inner sphere allylic amination step caused by small steric hinderance of **L10** might be responsible for the observed *trans*-diastereoselectivity. The necessity of two equivalent of **L10** (with regard to palladium) and the effect of less steric hindered ligand were validated by control experiments S3b and S2b, respectively.

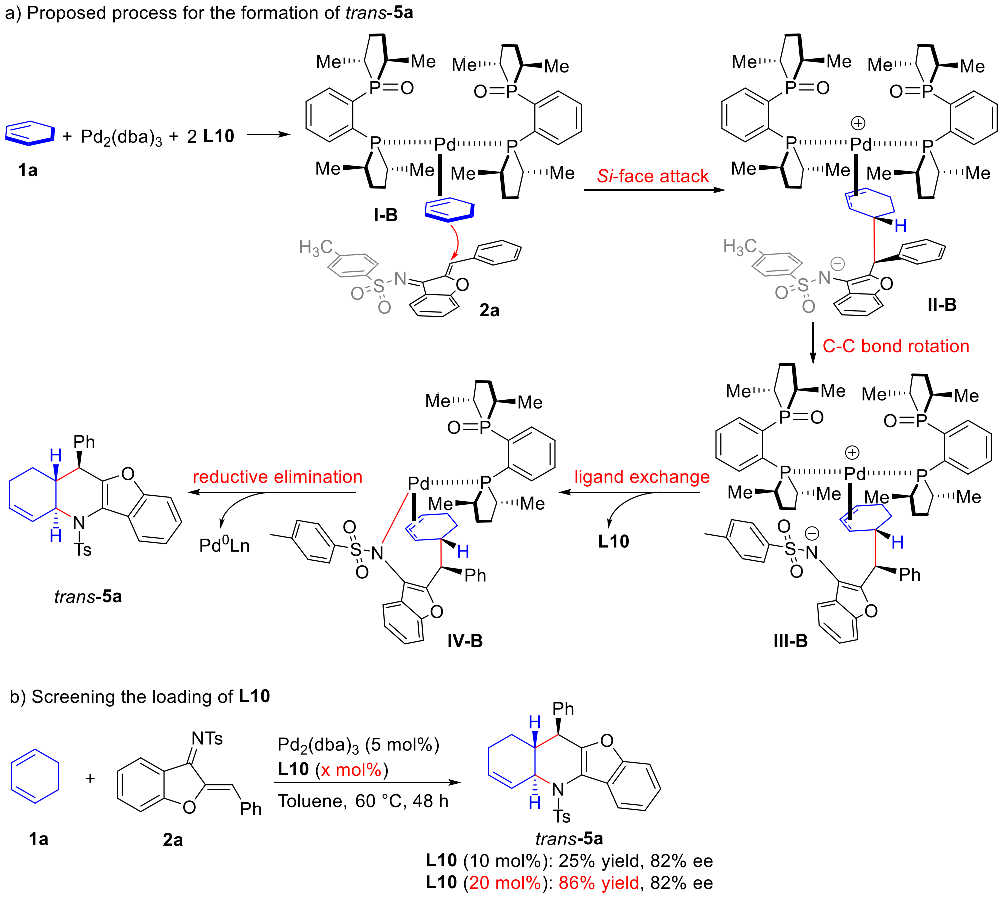

Figure S3 Proposed mechanism for the formation of *trans*-**5a** and control experiment

6.3 DFT calculations for the reaction of 1-azadiene 2a and 1,3-butadiene 15

The mechanism for the reaction of **2a** and **15** with/without $Pd(PPh_3)_4$ was calculated for further elucidating the catalytic process. The assembly of **2a** and **15** without palladium is a concerted Diels–Alder process. We calculated the **diene-*endo*-TS** and **diene-*exo*-TS** for the formation of *endo*-product **17** and *exo*-product **16**, and found the energy of **diene-*endo*-TS** with a value of 27.1 kcal/mol related to the energy summary of **15** and **2a**, was 4.6 kcal/mol lower than **diene-*exo*-TS**. These results were consistent with the results that **17** was produced with excellent *endo*-diastereoselectivity upon heating. On the other hand, the reaction mechanism of **2a** and **15** in the presence of $Pd(PPh_3)_4$ is a

stepwise process. Different configurations for the first addition step of the C-C bond formation have been calculated and the energy barriers were similar from **2a-TS1** and **2a-TS1′**, and the lower one with a value of 25.8 kcal/mol (**2a-TS1′**). Because **2a-INT1** and **2a-INT1′** could isomerize to each other via C-C bond rotation, the diastereoselectivity was determined by the second step. The energy of **diene-Pd-*exo*-TS** was 3.8 kcal/mol lower than that of **diene-Pd-*endo*-TS**, suggesting the *exo*-product **16** could be produced preferably, which is consistent with the experimental results.

Comparing the two reaction [with/without $Pd(PPh_3)_4$], the reaction of **2a** and **15** in the absence of $Pd(PPh_3)_4$ needed higher temperature as the energy barriers was 27.1 kcal/mol, which was 1.3 kcal/mol higher than the highest energy barrier of the reaction with $Pd(PPh_3)_4$ (25.8 kcal/mol). Thanks to the Pd^0-π-Lewis base activation, **INT1** showed significantly raised reactivity than parent **15**. These DFT calculations were consistent with the experimental results.

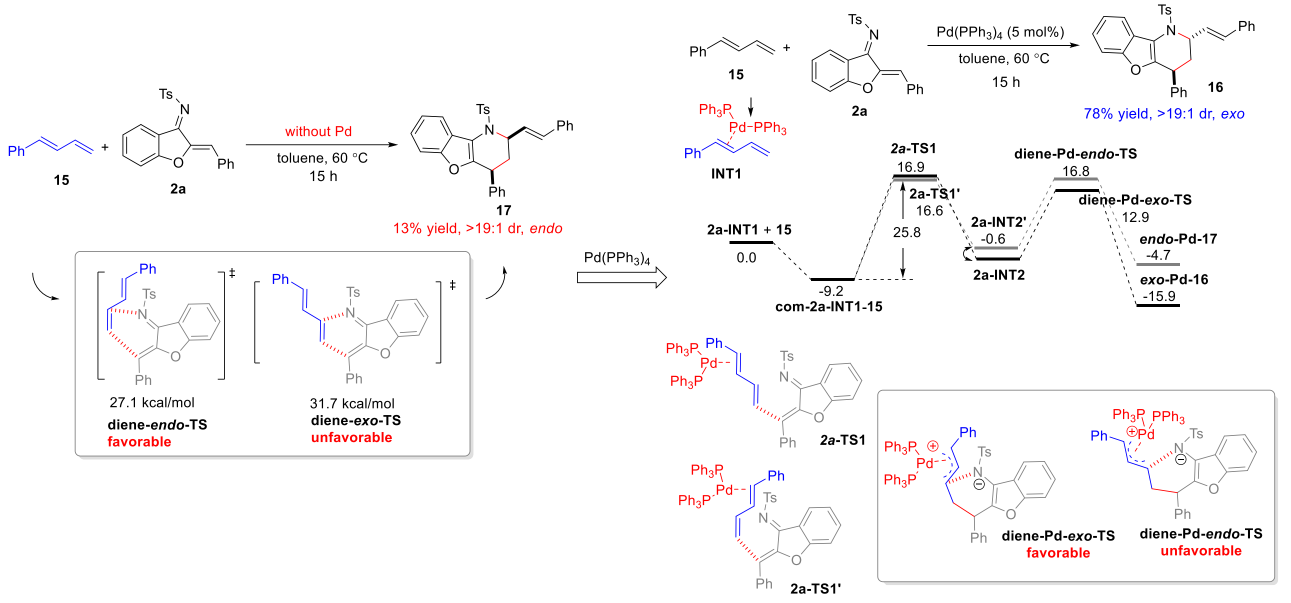

Figure S4 Computed potential energy surface of the reaction of **2a** and **15** with/without $Pd(PPh_3)_4$ at the B3LYP-D3/6-31(d)//B3LYP-D3/6-311++G(d,p) and SDD for Pd (toluene) level and are given in kcal/mol.

7. Crystal data and structural refinement

Procedure for the recrystallization of racemic 3a: To a 10 mL tube containing **3a** (16 mg) were added *n*-hexane (2 mL) and THF (2.5 mL). The mixture was heated until a clear solution was formed, which was kept aside at room temperature to obtain crystals. The crystals were subjected for single crystal XRD to determine the relative configuration of **3a**. The data were collected by Bruker APEX-II CCD equipped with a Mo radiation source (Kα = 0.71073 Å) at 290.0 K. CCDC 2050825 (**3a**) contains the supplementary crystallographic data for this paper. These data can be obtained free of

charge via www.ccdc.cam.ac.uk/data_request/cif.

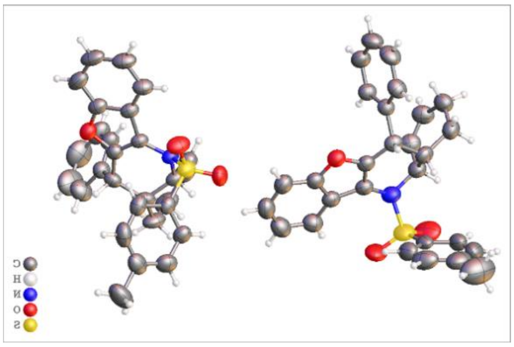

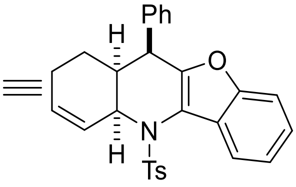

3a

(ellipsoid contour probability 50%)

Identification code	**3a**
Empirical formula	$C_{28}H_{25}NO_3S$
Formula weight	455.55
Temperature/K	290.0
Crystal system	triclinic
Space group	P-1
a/Å	11.6139(7)
b/Å	13.4764(10)
c/Å	14.9925(10)
α/°	92.051(3)
β/°	93.362(3)
γ/°	94.406(3)
Volume/Å^3	2333.7(3)
Z	4
ρ_{calc}g/cm^3	1.297
μ/mm^{-1}	0.169
F(000)	960.0
Crystal size/mm^3	0.3 × 0.3 × 0.2
Radiation	MoKα (λ = 0.71073)
2Θ range for data collection/°	3.994 to 55.098
Index ranges	$-15 \leq h \leq 14$, $-17 \leq k \leq 17$, $-19 \leq l \leq 19$
Reflections collected	85300
Independent reflections	10757 [R_{int} = 0.1297, R_{sigma} = 0.1036]
Data/restraints/parameters	10757/0/597

Goodness-of-fit on F^2	1.038
Final R indexes [I>=2σ (I)]	R_1 = 0.0603, wR_2 = 0.1328
Final R indexes [all data]	R_1 = 0.1390, wR_2 = 0.1605
Largest diff. peak/hole / e Å^{-3}	0.17/-0.33

Procedure for the recrystallization of chiral 4a: To a 10 mL tube containing **4a** (20 mg) were added *n*-hexane (2 mL) and *i*-PrOH (2.5 mL). The mixture was heated until a clear solution was formed, which was kept aside at room temperature to obtain crystals. The crystals were subjected for single crystal XRD to determine the absolute configuration of **4a**. The data were collected by an Agilent Gemini equipped a Cu radiation source (Kα = 1.54184 Å) at 292.90 K. CCDC 2050826 (**4a**) contains the supplementary crystallographic data for this paper. These data can be obtained free of charge via www.ccdc.cam.ac.uk/data_request/cif.

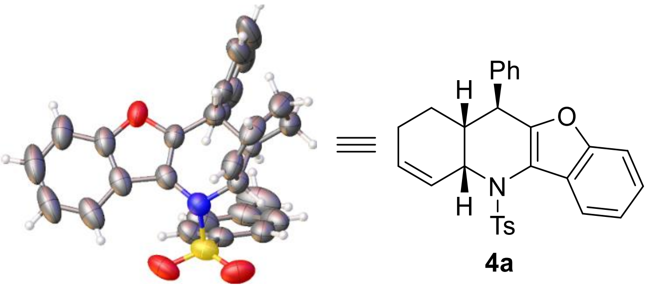

4a

(ellipsoid contour probability 50%)

Identification code	**4a**
Empirical formula	$C_{28}H_{25}NO_3S$
Formula weight	455.55
Temperature/K	292.90(10)
Crystal system	orthorhombic
Space group	$P2_12_12_1$
a/Å	11.0546(3)
b/Å	11.54205(18)
c/Å	18.2492(4)
α/°	90
β/°	90
γ/°	90
Volume/Å^3	2328.47(8)
Z	4

ρ_{calc}g/cm^3	1.299
μ/mm^{-1}	1.475
F(000)	960.0
Crystal size/mm^3	0.4 × 0.2 × 0.1
Radiation	CuKα (λ = 1.54184)
2Θ range for data collection/°	9.066 to 142.848
Index ranges	$-13 \leq h \leq 13$, $-14 \leq k \leq 8$, $-21 \leq l \leq 22$
Reflections collected	12665
Independent reflections	4476 [R_{int} = 0.0352, R_{sigma} = 0.0307]
Data/restraints/parameters	4476/0/299
Goodness-of-fit on F^2	1.049
Final R indexes [I>=2σ (I)]	R_1 = 0.0564, wR_2 = 0.1522
Final R indexes [all data]	R_1 = 0.0596, wR_2 = 0.1590
Largest diff. peak/hole / e Å^{-3}	0.27/-0.27
Flack parameter	0.011(12)

Procedure for the recrystallization of chiral 5e: To a 10 mL tube containing **5e** (15 mg) were added *n*-hexane (1.5 mL) and *i*-PrOH (2.5 mL). The mixture was heated until a clear solution was formed, which was kept aside at room temperature to obtain crystals. The crystals were subjected for single crystal XRD to determine the absolute configuration of **5e**. The data were collected by a Bruker APEX-II CCD equipped with a Mo radiation source (Kα = 0.71073 Å) at 301.0 K. CCDC 2219802 (**5e**) contains the supplementary crystallographic data for this paper. These data can be obtained free of charge via www.ccdc.cam.ac.uk/data_request/cif.

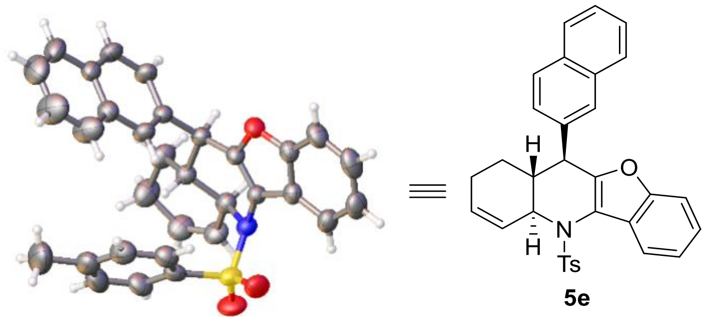

5e

(ellipsoid contour probability 50%)

Identification code	**5e**
Empirical formula	$C_{32}H_{27}NO_3S$

Formula weight	505.60
Temperature/K	301.0
Crystal system	orthorhombic
Space group	$P2_12_12_1$
a/Å	8.7448(3)
b/Å	12.1996(5)
c/Å	23.9382(10)
α/°	90
β/°	90
γ/°	90
Volume/Å^3	2553.80(17)
Z	4
ρ_{calc}g/cm^3	1.315
μ/mm^{-1}	0.162
F(000)	1064.0
Crystal size/mm^3	0.36 × 0.15 × 0.13
Radiation	MoKα (λ = 0.71073)
2Θ range for data collection/ °	4.768 to 55.018
Index ranges	-11 ≤ h ≤ 11, -15 ≤ k ≤ 15, -31 ≤ l ≤ 31
Reflections collected	38309
Independent reflections	5866 [R_{int} = 0.0960, R_{sigma} = 0.0518]
Data/restraints/parameters	5866/0/335
Goodness-of-fit on F^2	1.008
Final R indexes [I>=2σ (I)]	R_1 = 0.0389, wR_2 = 0.0801
Final R indexes [all data]	R_1 = 0.0727, wR_2 = 0.0915
Largest diff. peak/hole / e Å^{-3}	0.13/-0.25
Flack parameter	-0.01(4)

Procedure for the recrystallization of chiral 11: To a 10 mL tube containing **11** (15 mg) were added *n*-hexane (1.5 mL) and EtOAc (2.5 mL). The mixture was heated until a clear solution was formed, which was kept aside at room temperature to obtain crystals. The crystals were subjected for single crystal XRD to determine the absolute configuration of **11**. The data were collected by a Bruker APEX-II CCD equipped with a Mo radiation source (Kα = 0.71073 Å) at 273.15 K. CCDC 2219803

(**11**) contains the supplementary crystallographic data for this paper. These data can be obtained free of charge via www.ccdc.cam.ac.uk/data_request/cif.

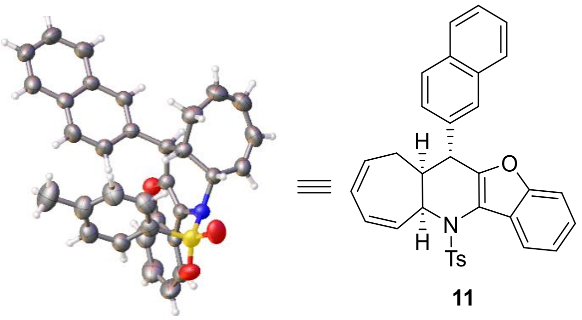

(ellipsoid contour probability 50%)

Identification code	**11**
Empirical formula	$C_{33}H_{27}NO_3S$
Formula weight	517.61
Temperature/K	273.15
Crystal system	monoclinic
Space group	$P2_1$
a/Å	9.7335(10)
b/Å	12.7669(13)
c/Å	10.7629(11)
α/°	90
β/°	90.849(4)
γ/°	90
Volume/Å^3	1337.3(2)
Z	2
ρ_{calc}g/cm^3	1.285
μ/mm^{-1}	0.156
F(000)	544.0
Crystal size/mm^3	0.31 × 0.23 × 0.11
Radiation	MoKα (λ = 0.71073)
2Θ range for data collection/°	4.186 to 55.136
Index ranges	$-12 \leq h \leq 12$, $-16 \leq k \leq 16$, $-13 \leq l \leq 14$
Reflections collected	28070
Independent reflections	6155 [R_{int} = 0.0588, R_{sigma} = 0.0466]

Data/restraints/parameters	6155/1/344
Goodness-of-fit on F^2	1.027
Final R indexes [I>=2σ (I)]	R_1 = 0.0540, wR_2 = 0.1333
Final R indexes [all data]	R_1 = 0.0738, wR_2 = 0.1450
Largest diff. peak/hole / e $Å^{-3}$	0.56/-0.22
Flack parameter	0.08(3)

Procedure for the recrystallization of chiral 16: To a 10 mL tube containing **16** (15 mg) were added *n*-hexane (1.5 mL) and THF (2.5 mL). The mixture was heated until a clear solution was formed, which was kept aside at room temperature to obtain crystals. The crystals were subjected for single crystal XRD to determine the absolute configuration of **16**. The data were collected by a Bruker APEX-II CCD equipped with a Mo radiation source (Kα = 0.71073 Å) at 300.0 K. CCDC 2219804 (**16**) contains the supplementary crystallographic data for this paper. These data can be obtained free of charge via www.ccdc.cam.ac.uk/data_request/cif.

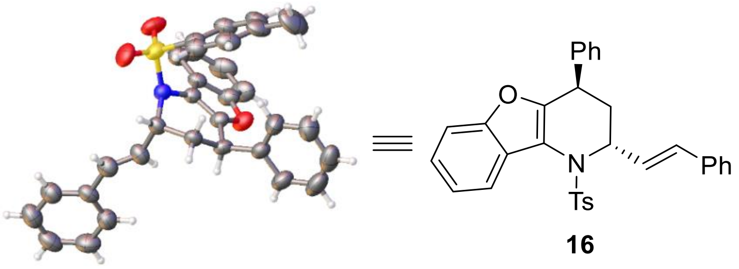

16

(ellipsoid contour probability 50%)

Identification code	**16**
Empirical formula	$C_{32}H_{27}NO_3S$
Formula weight	505.60
Temperature/K	300.0
Crystal system	orthorhombic
Space group	$P2_12_12_1$
a/Å	7.9263(6)
b/Å	10.6732(11)
c/Å	31.221(3)
α/°	90
β/°	90
γ/°	90

Volume/Å^3	2641.2(4)
Z	4
ρ_{calc}g/cm^3	1.271
μ/mm^{-1}	0.157
F(000)	1064.0
Crystal size/mm^3	0.41 × 0.12 × 0.11
Radiation	MoKα (λ = 0.71073)
2Θ range for data collection/°	4.032 to 54.916
Index ranges	$-10 \le h \le 10$, $-13 \le k \le 13$, $-40 \le l \le 34$
Reflections collected	23184
Independent reflections	6027 [R_{int} = 0.0697, R_{sigma} = 0.0658]
Data/restraints/parameters	6027/0/335
Goodness-of-fit on F^2	1.023
Final R indexes [I>=2σ (I)]	R_1 = 0.0493, wR_2 = 0.0937
Final R indexes [all data]	R_1 = 0.1072, wR_2 = 0.1168
Largest diff. peak/hole / e Å^{-3}	0.32/-0.23
Flack parameter	-0.05(6)

Procedure for the recrystallization of racemic 17: To a 10 mL tube containing **17** (15 mg) were added *n*-hexane (1.5 mL) and *i*-PrOH (2.5 mL). The mixture was heated until a clear solution was formed, which was kept aside at room temperature to obtain crystals. The crystals were subjected for single crystal XRD to determine the relative configuration of **17**. The data were collected by an Agilent Gemini equipped with a Cu radiation source (Kα = 1.54184 Å) at 294.93 K. CCDC 2050823 (**17**) contains the supplementary crystallographic data for this paper. These data can be obtained free of charge via www.ccdc.cam.ac.uk/data_request/cif.

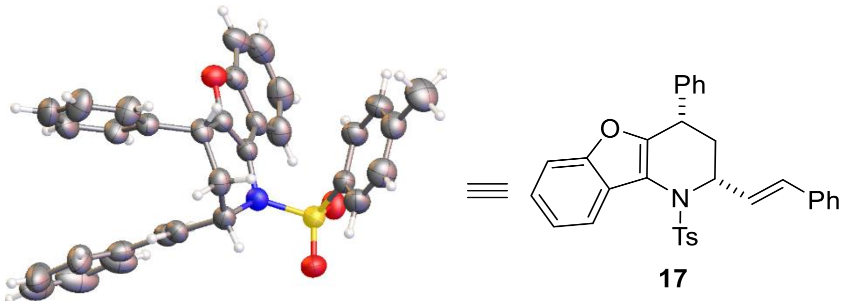

17

(ellipsoid contour probability 50%)

Identification code	**17**

Empirical formula	$C_{32}H_{27}NO_3S$
Formula weight	505.60
Temperature/K	294.93(10)
Crystal system	monoclinic
Space group	$P2_1/n$
a/Å	13.2549(3)
b/Å	11.5853(4)
c/Å	17.1735(4)
α/°	90
β/°	97.293(2)
γ/°	90
Volume/Å^3	2615.86(12)
Z	4
ρ_{calc}g/cm^3	1.284
μ/mm^{-1}	1.368
F(000)	1064.0
Crystal size/mm^3	0.5 × 0.4 × 0.3
Radiation	CuKα (λ = 1.54184)
2Θ range for data collection/°	7.956 to 143.642
Index ranges	-16 ≤ h ≤ 16, -12 ≤ k ≤ 14, -15 ≤ l ≤ 21
Reflections collected	14291
Independent reflections	5019 [R_{int} = 0.0407, R_{sigma} = 0.0332]
Data/restraints/parameters	5019/0/335
Goodness-of-fit on F^2	1.033
Final R indexes [I>=2σ (I)]	R_1 = 0.0607, wR_2 = 0.1639
Final R indexes [all data]	R_1 = 0.0687, wR_2 = 0.1763
Largest diff. peak/hole / e Å^{-3}	0.26/-0.45

Procedure for the recrystallization of chiral 21: To a 10 mL tube containing **21** (15 mg) were added *n*-hexane (1.5 mL) and THF (2.5 mL). The mixture was heated until a clear solution was formed, which was kept aside at room temperature to obtain crystals. The crystals were subjected for single crystal XRD to determine the absolute configuration of **21**. The data were collected by a Bruker APEX-II CCD equipped with a Mo radiation source (Kα = 0.71073 Å) at 273.15 K. CCDC 2219805

(**21**) contains the supplementary crystallographic data for this paper. These data can be obtained free of charge via www.ccdc.cam.ac.uk/data_request/cif.

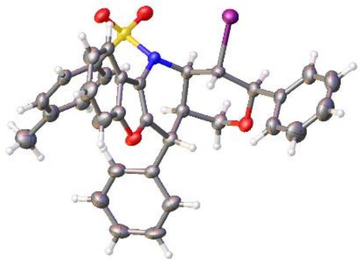

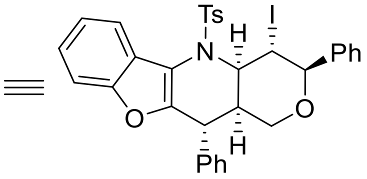

21

(ellipsoid contour probability 50%)

Identification code	**21**
Empirical formula	$C_{33}H_{28}INO_4S$
Formula weight	661.52
Temperature/K	161.0
Crystal system	orthorhombic
Space group	$P2_12_12_1$
a/Å	11.0840(12)
b/Å	11.2741(10)
c/Å	27.234(3)
α/°	90
β/°	90
γ/°	90
Volume/$Å^3$	3403.2(6)
Z	4
$\rho_{calc}g/cm^3$	1.291
μ/mm^{-1}	1.035
F(000)	1336.0
Crystal size/mm^3	0.35 × 0.22 × 0.09
Radiation	MoKα (λ = 0.71073)
2Θ range for data collection/°	4.738 to 55.044
Index ranges	$-14 \leq h \leq 14, -13 \leq k \leq 14, -35 \leq l \leq 35$
Reflections collected	51913
Independent reflections	7778 [R_{int} = 0.0814, R_{sigma} = 0.0497]
Data/restraints/parameters	7778/0/362

Goodness-of-fit on F^2	1.020
Final R indexes [I>=2σ (I)]	R_1 = 0.0286, wR_2 = 0.0596
Final R indexes [all data]	R_1 = 0.0420, wR_2 = 0.0633
Largest diff. peak/hole / e Å^{-3}	0.31/-0.45
Flack parameter	0.001(10)

Procedure for the recrystallization of racemic 25: To a 10 mL tube containing **25** (15 mg) were added *n*-hexane (1.5 mL) and *i*-PrOH (2.5 mL). The mixture was heated until a clear solution was formed, which was kept aside at room temperature to obtain crystals. The crystals were subjected for single crystal XRD to determine the relative configuration of **25**. The data were collected by a Bruker APEX-II CCD equipped with a Mo radiation source (Kα = 0.71073 Å) at 273.15 K. CCDC 2219806 (**25**) contains the supplementary crystallographic data for this paper. These data can be obtained free of charge via www.ccdc.cam.ac.uk/data_request/cif.

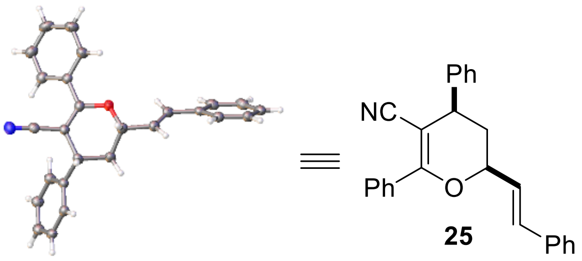

(ellipsoid contour probability 50%)

Identification code	**25**
Empirical formula	$C_{26}H_{21}NO$
Formula weight	363.44
Temperature/K	149.0
Crystal system	monoclinic
Space group	$P2_1/n$
a/Å	10.7659(11)
b/Å	13.1580(15)
c/Å	27.887(3)
α/°	90
β/°	100.454(4)
γ/°	90
Volume/Å^3	3884.8(7)

Z	8
ρ_{calc}g/cm^3	1.243
μ/mm^{-1}	0.075
F(000)	1536.0
Crystal size/mm^3	0.45 × 0.13 × 0.04
Radiation	MoKα (λ = 0.71073)
2Θ range for data collection/ °	4.29 to 55.022
Index ranges	$-13 \le h \le 13$, $-17 \le k \le 17$, $-36 \le l \le 33$
Reflections collected	36992
Independent reflections	8881 [R_{int} = 0.1161, R_{sigma} = 0.1068]
Data/restraints/parameters	8881/0/505
Goodness-of-fit on F^2	1.021
Final R indexes [I>=2σ (I)]	R_1 = 0.0681, wR_2 = 0.1649
Final R indexes [all data]	R_1 = 0.1112, wR_2 = 0.1963
Largest diff. peak/hole / e Å^{-3}	0.37/-0.32

8. NMR, HRMS spectra and HPLC chromatograms

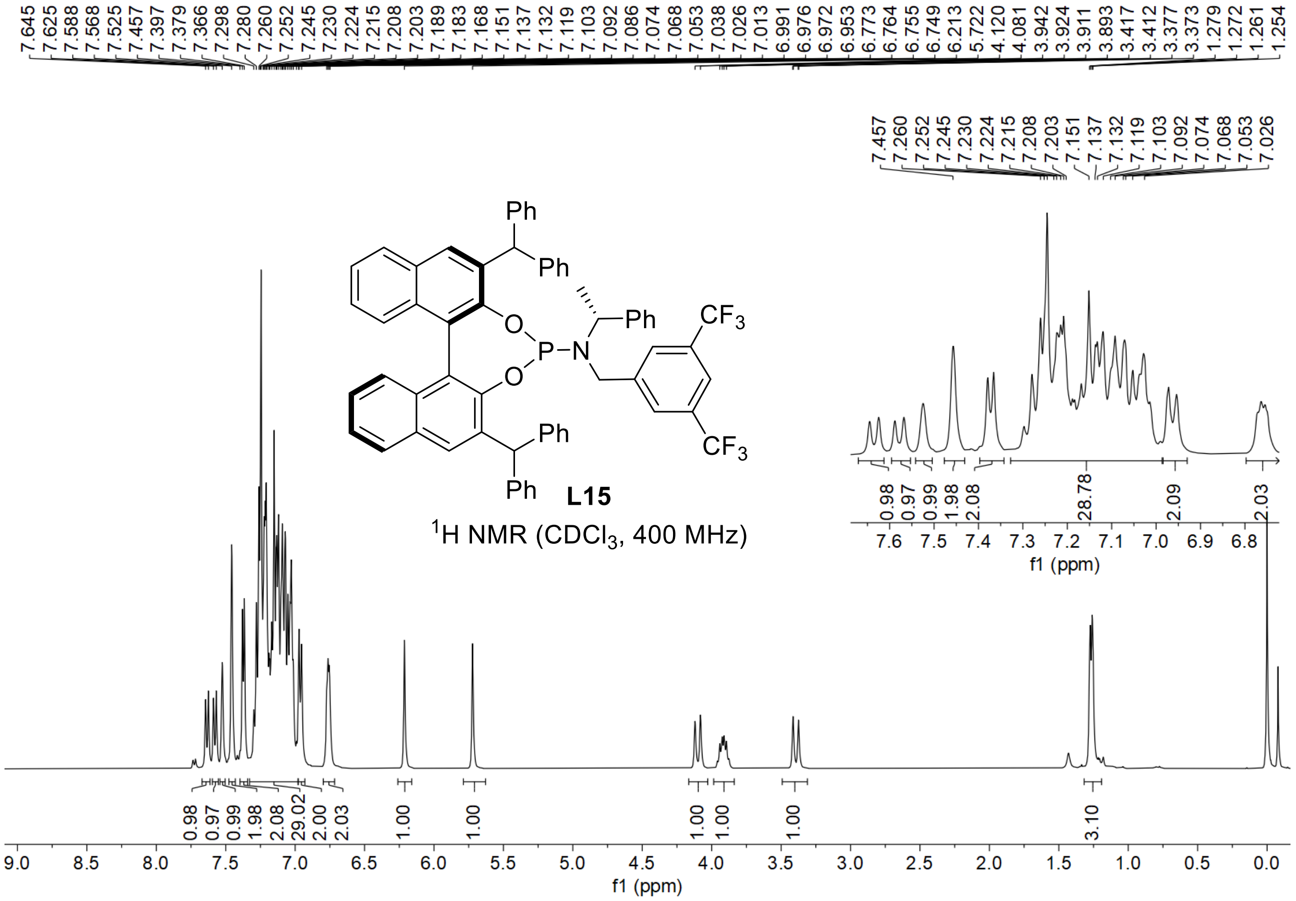

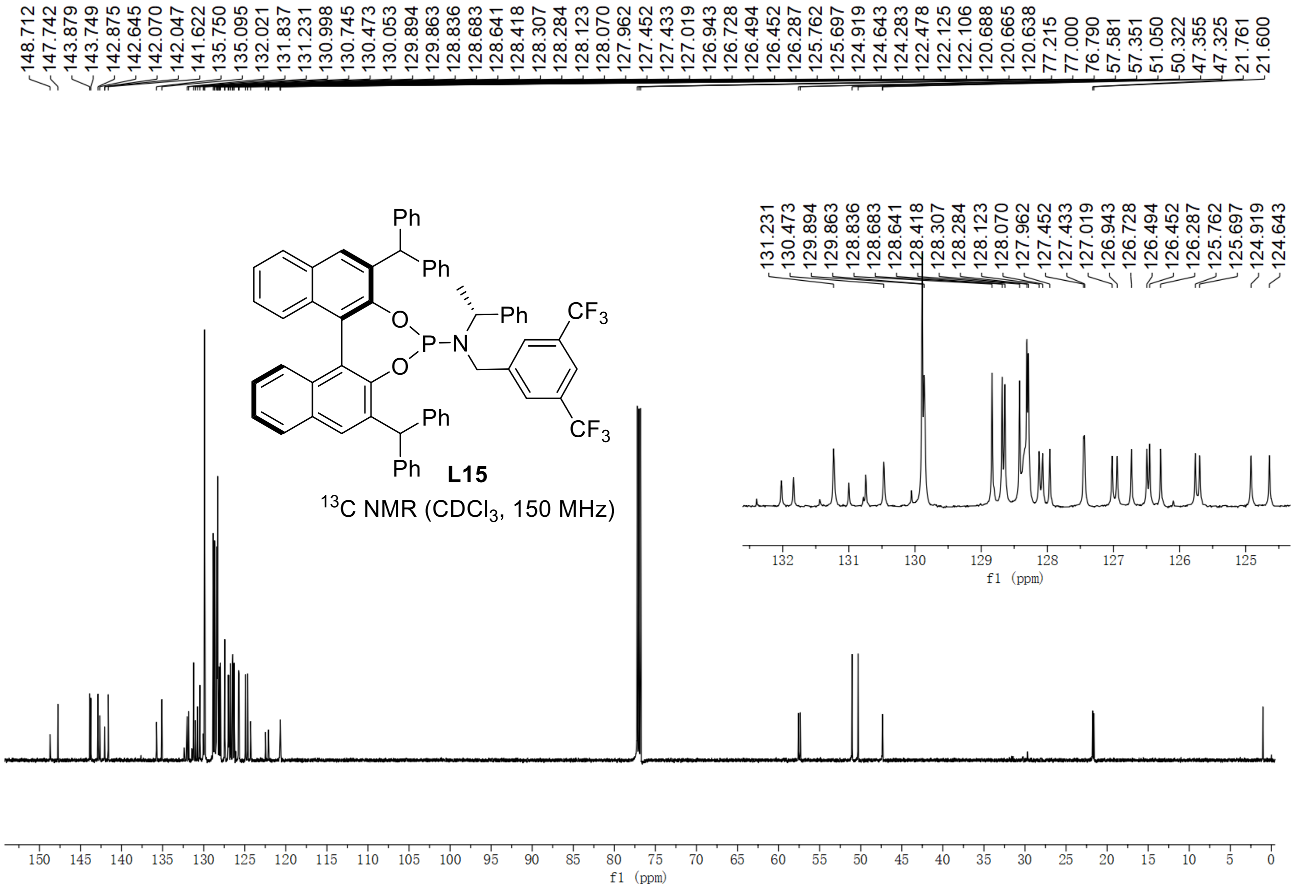

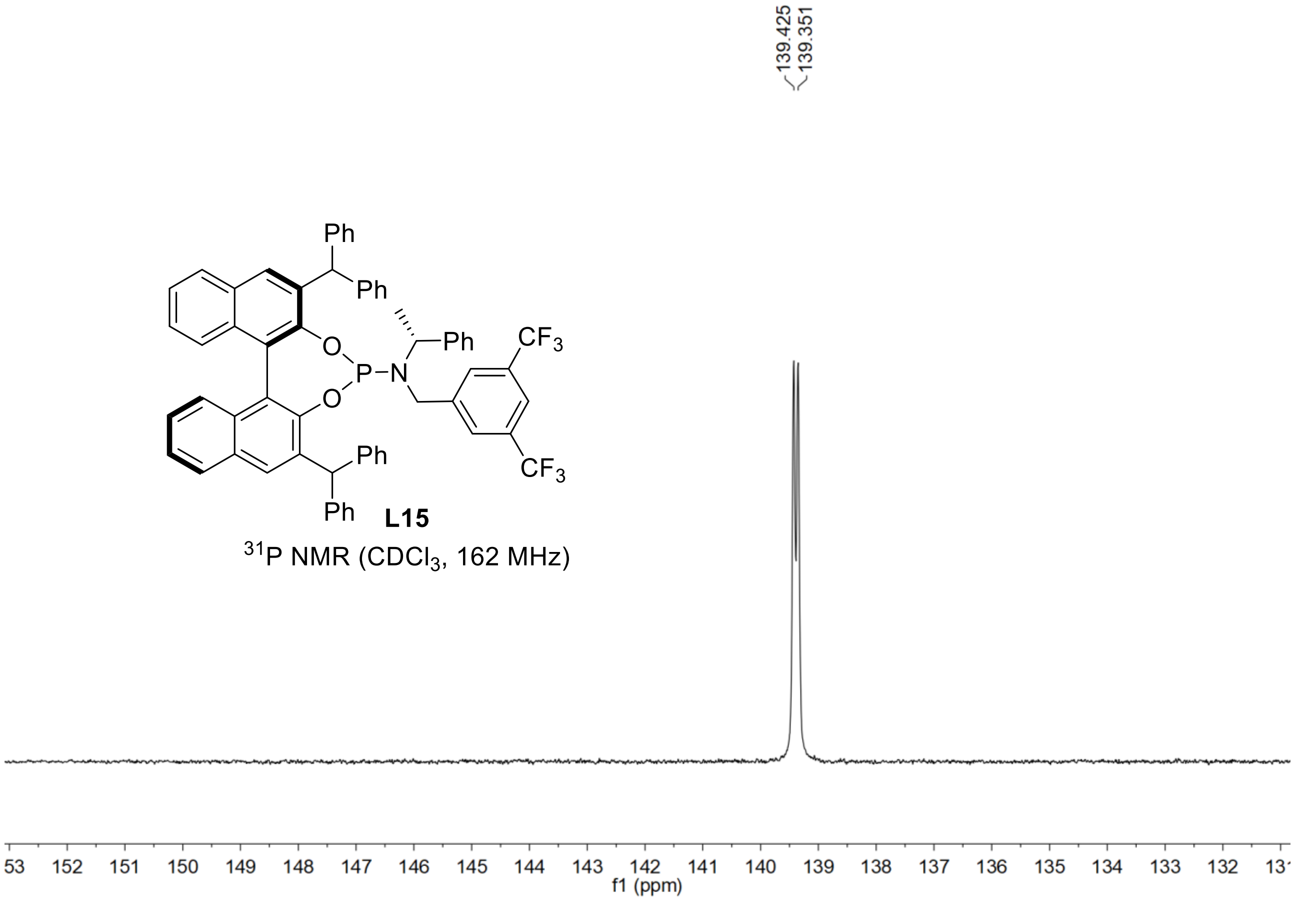
139.425
139.351
Ph
Ph
Ph
CF3
O
P–N
O
Ph
CF3
Ph
L15
31P NMR (CDCl3, 162 MHz)
53 152 151 150 149 148 147 146 145 144 143 142 141 140 139 138 137 136 135 134 133 132
f1 (ppm)

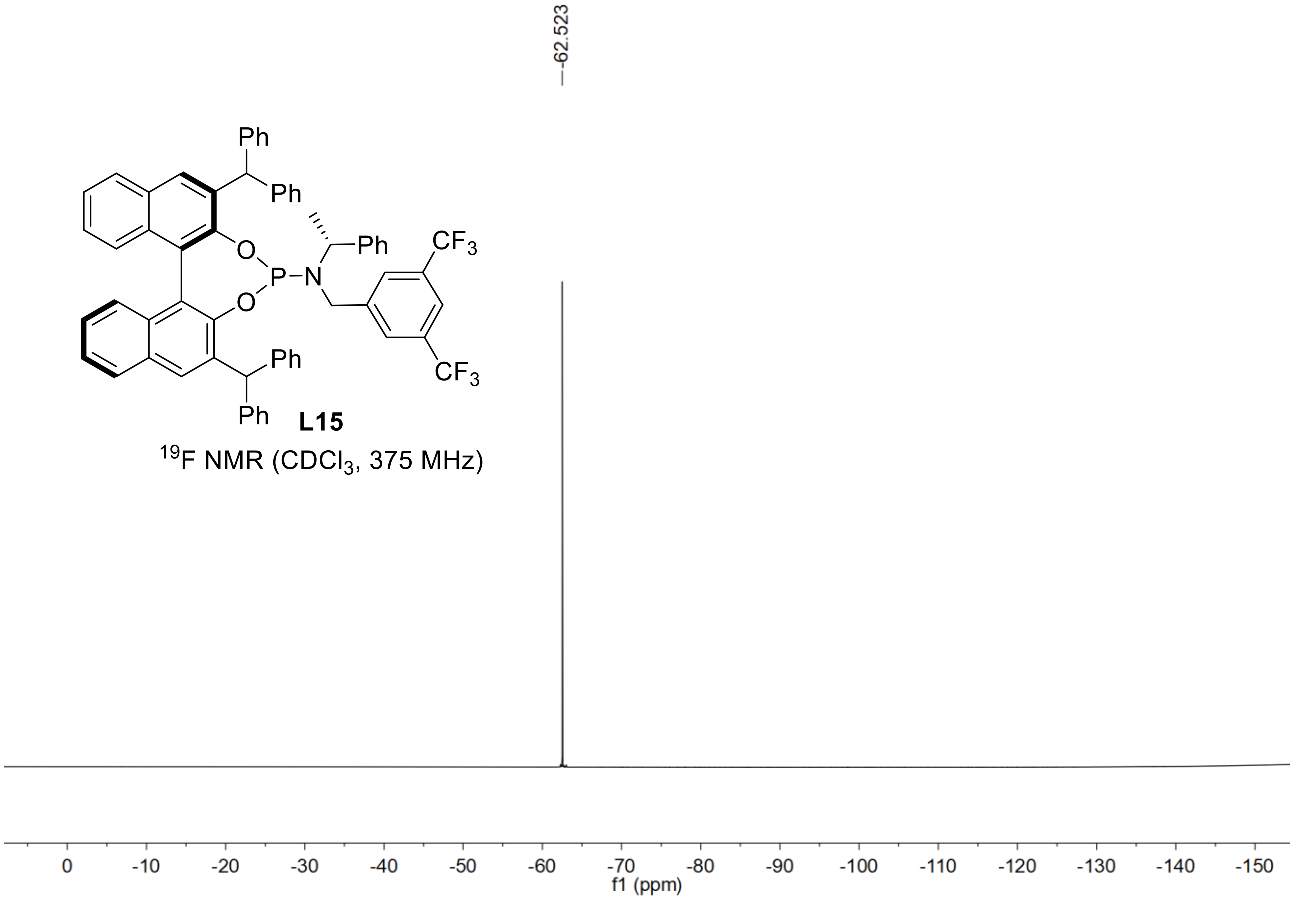
-62.523
Ph
Ph
Ph
CF3
O
P–N
O
Ph
CF3
Ph
L15
19F NMR (CDCl3, 375 MHz)
0 -10 -20 -30 -40 -50 -60 -70 -80 -90 -100 -110 -120 -130 -140 -150
f1 (ppm)

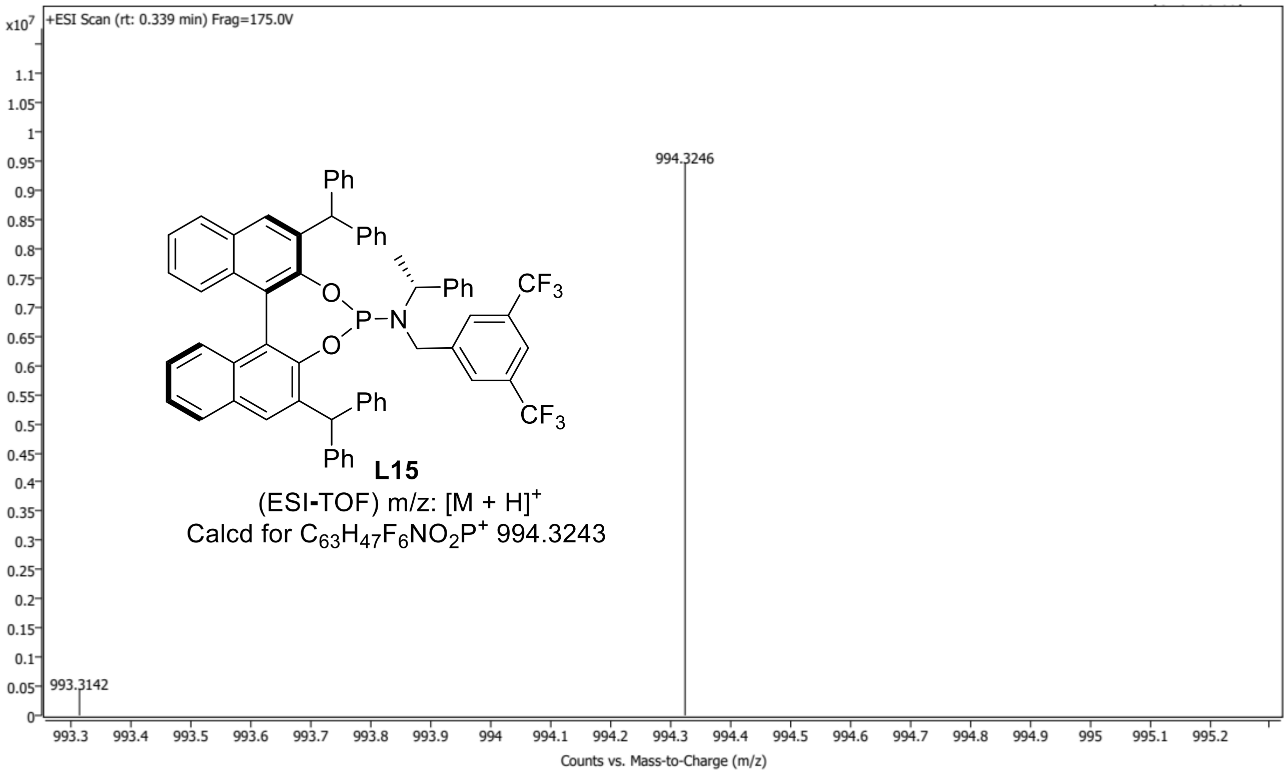

x10^7
+ESI Scan (rt: 0.339 min) Frag=175.0V
994.3246
993.3142
L15
(ESI-TOF) m/z: [M + H]+
Calcd for C63H47F6NO2P+ 994.3243
Counts vs. Mass-to-Charge (m/z)

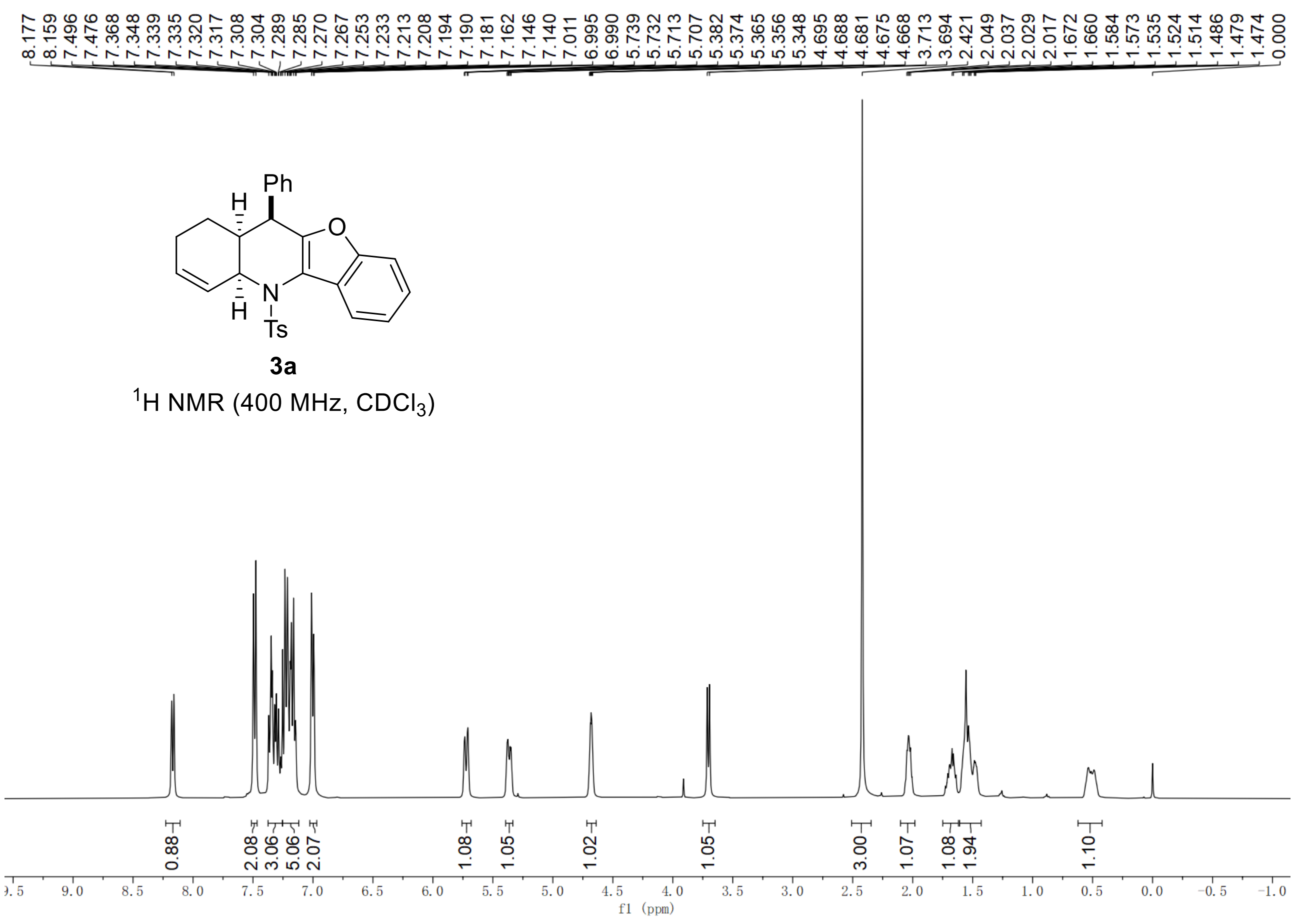

3a

^{1}H NMR (400 MHz, $CDCl_3$)

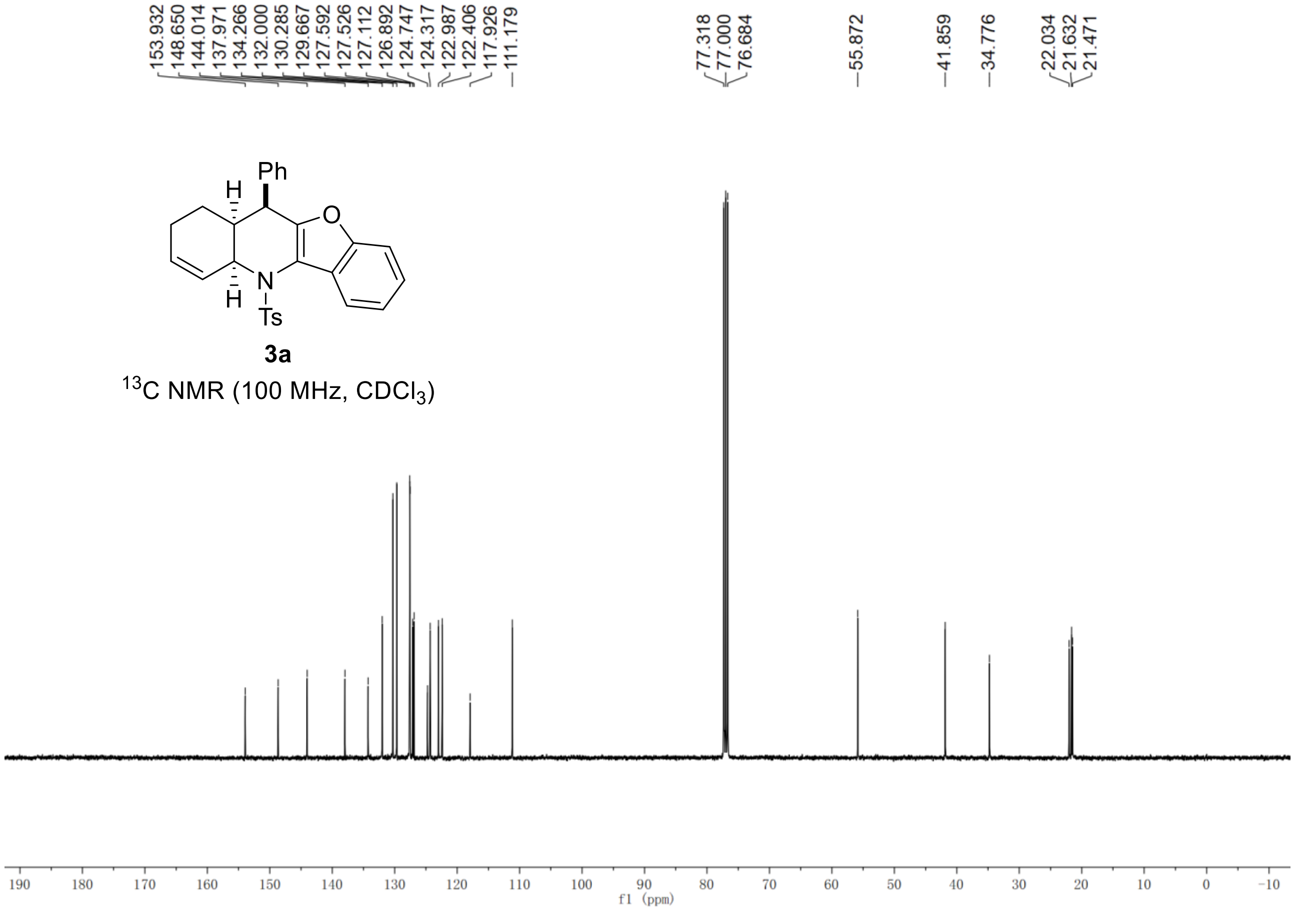

3a

^{13}C NMR (100 MHz, $CDCl_3$)

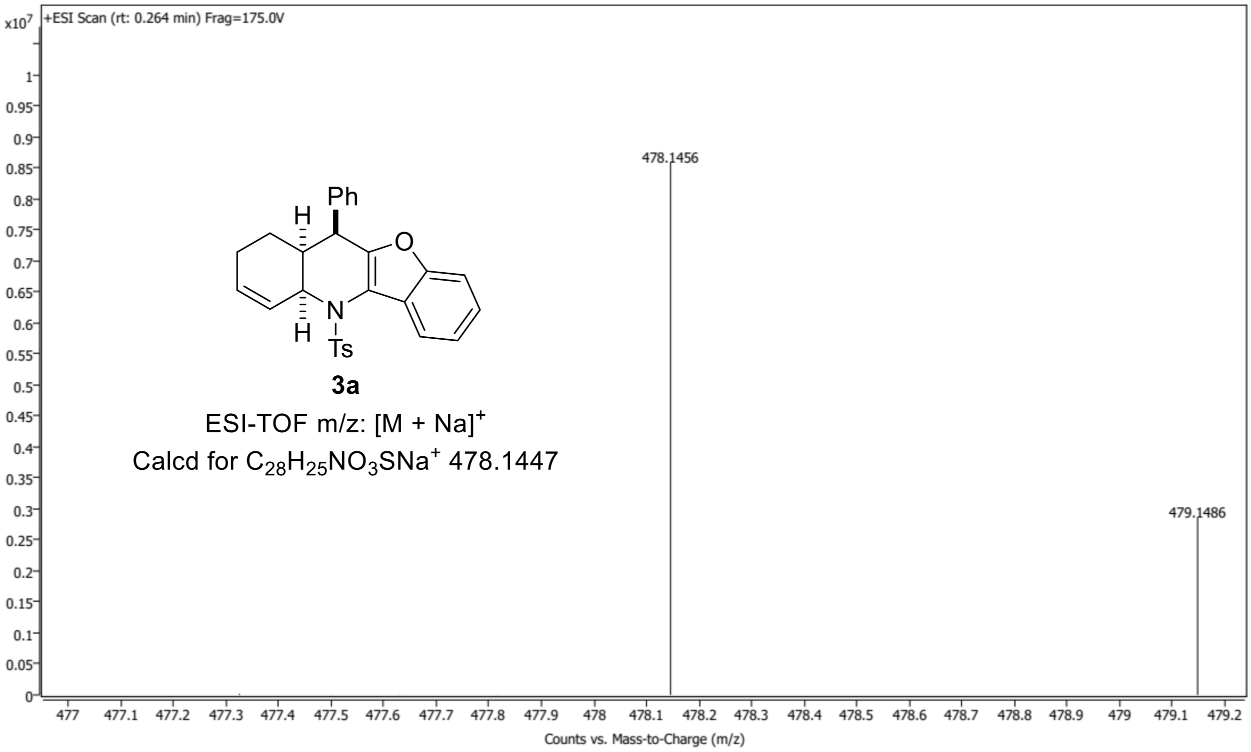
+ESI Scan (rt: 0.264 min) Frag=175.0V
x10^7
478.1456
479.1486
Ph
H
O
N
H
Ts
3a
ESI-TOF m/z: [M + Na]+
Calcd for $C_{28}H_{25}NO_3SNa^+$ 478.1447
Counts vs. Mass-to-Charge (m/z)

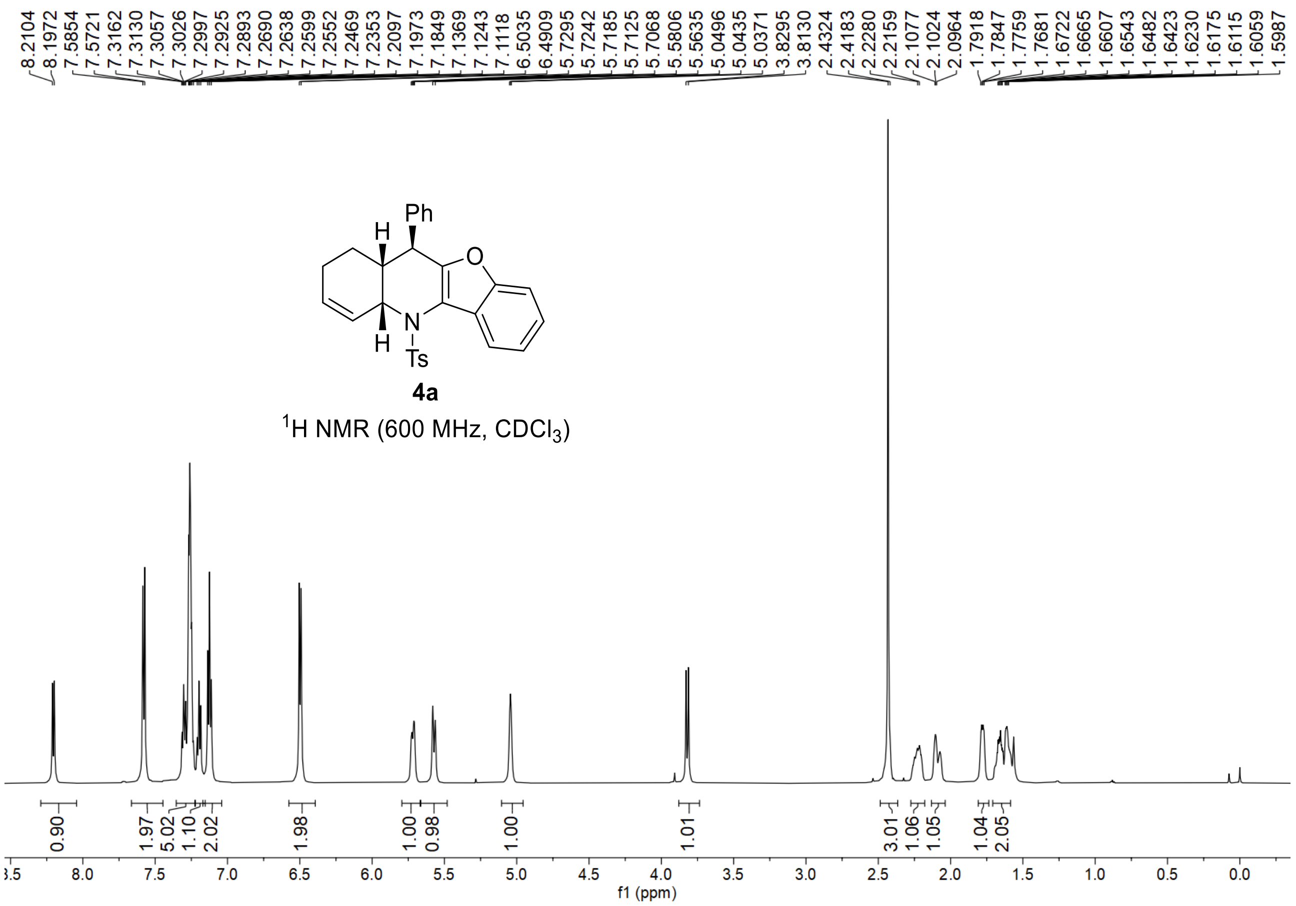
8.2104
8.1972
7.5854
7.5721
7.3162
7.3130
7.3057
7.3026
7.2997
7.2925
7.2893
7.2690
7.2638
7.2599
7.2552
7.2469
7.2353
7.2097
7.1973
7.1849
7.1369
7.1243
7.1118
6.5035
6.4909
5.7295
5.7242
5.7185
5.7125
5.7068
5.5806
5.5635
5.0496
5.0435
5.0371
3.8295
3.8130
2.4324
2.4183
2.2280
2.2159
2.1077
2.1024
2.0964
1.7918
1.7847
1.7759
1.7681
1.6722
1.6665
1.6607
1.6543
1.6482
1.6423
1.6230
1.6175
1.6115
1.6059
1.5987
Ph
H
O
N
H
Ts
4a
^{1}H NMR (600 MHz, CDCl$_3$)
0.90
1.97
5.02
1.10
2.02
1.98
1.00
0.98
1.00
1.01
3.01
1.06
1.05
1.04
2.05
f1 (ppm)

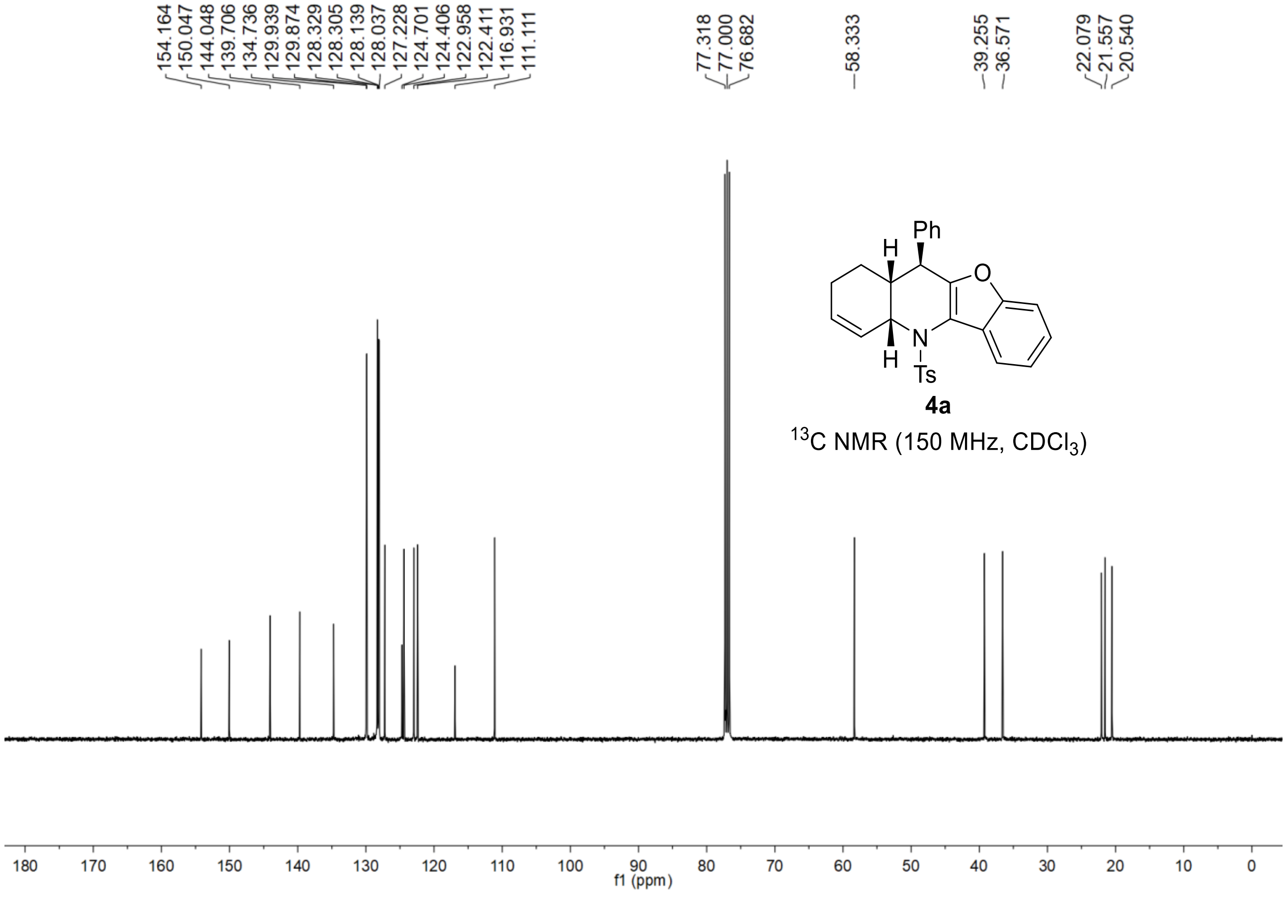
154.164
150.047
144.048
139.706
134.736
129.939
129.874
128.329
128.305
128.139
128.037
127.228
124.701
124.406
122.958
122.411
116.931
111.111
77.318
77.000
76.682
58.333
39.255
36.571
22.079
21.557
20.540
Ph
H
O
N
H
Ts
4a
^{13}C NMR (150 MHz, CDCl$_3$)
f1 (ppm)

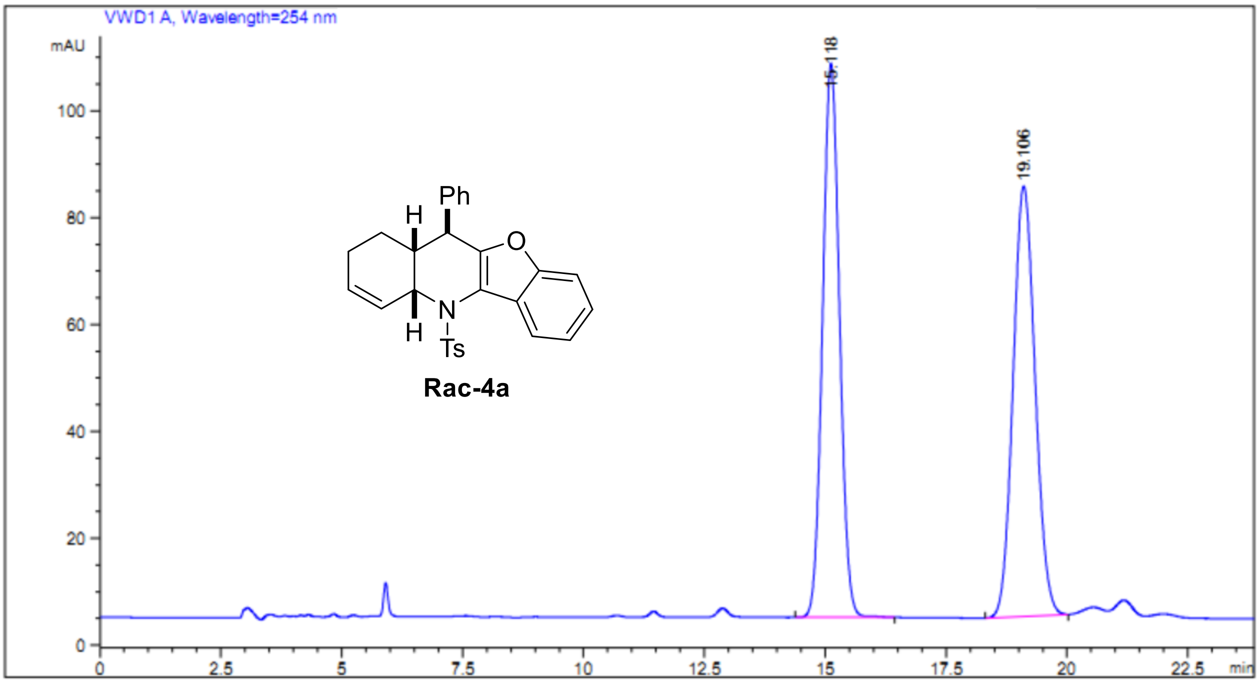

#	[min]		[min]	[mAU*s]	[mAU]	%
1	15.118	BB	0.3786	2521.94507	103.63020	49.7283
2	19.106	BB	0.4916	2549.49829	80.53268	50.2717

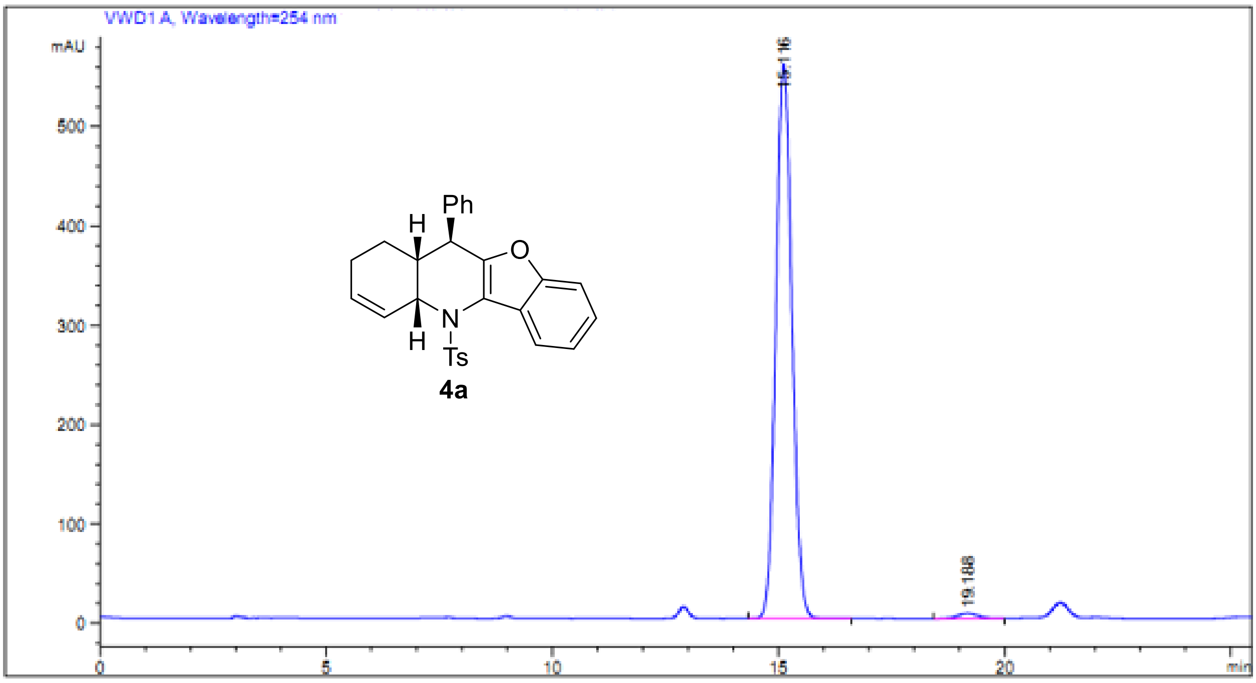

#	[min]		[min]	[mAU*s]	[mAU]	%
1	15.116	BB	0.3858	1.37939e4	558.28955	98.7704
2	19.188	BB	0.5049	171.71707	5.35892	1.2296

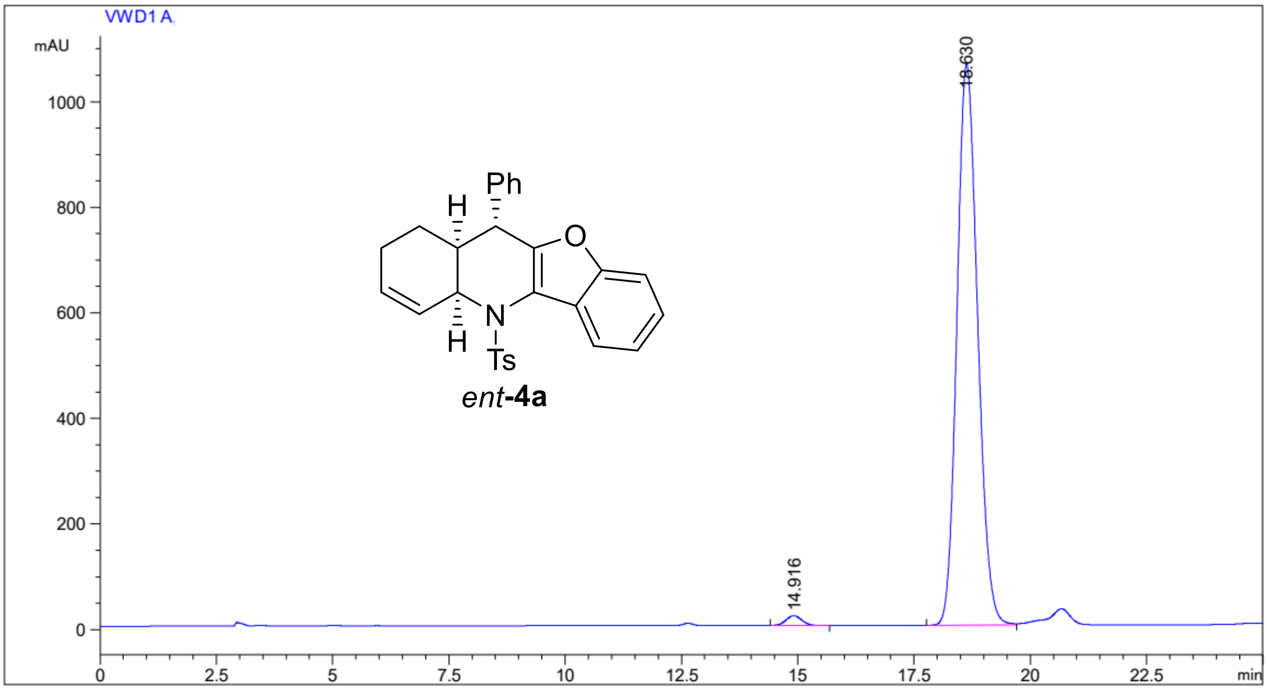

#	[min]		[min]	mAU	*s	[mAU]	%
1	14.916	VB	0.3736	448.99615		18.68349	1.3493
2	18.630	BV	0.4810	3.28265e4		1063.50916	98.6507

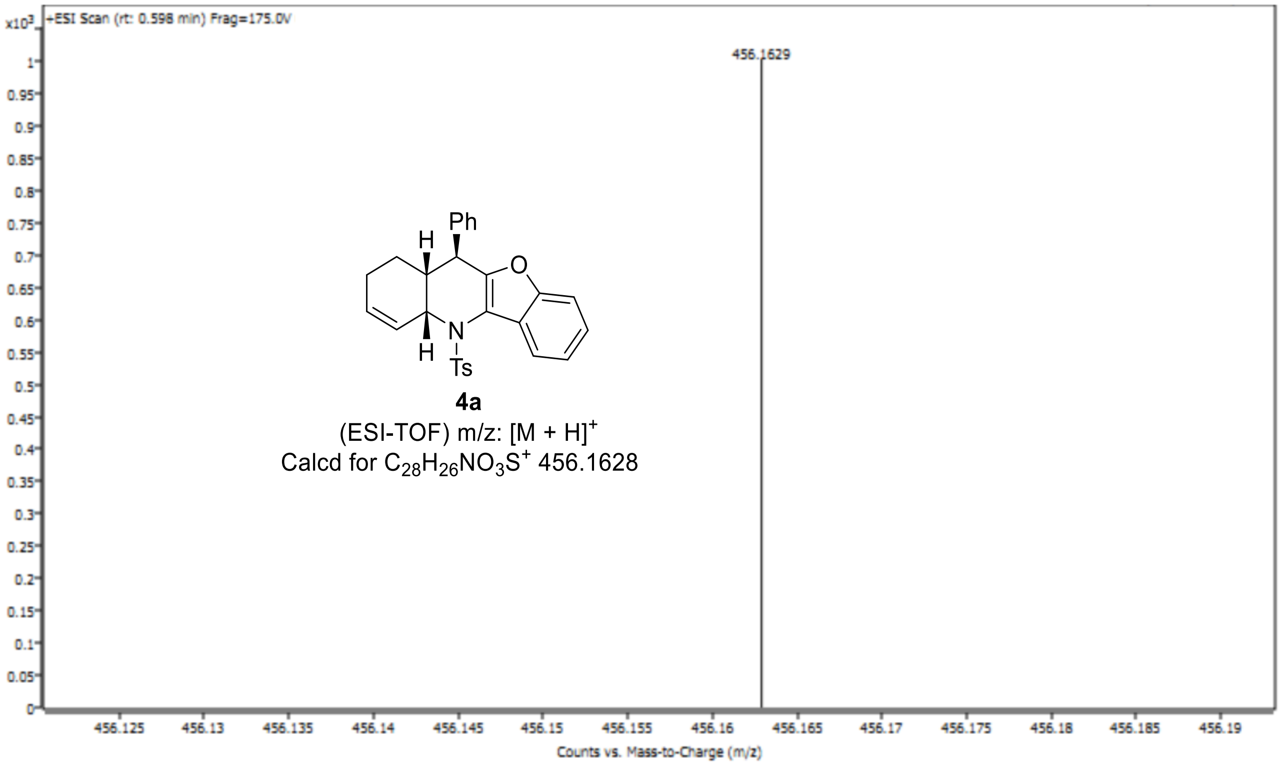

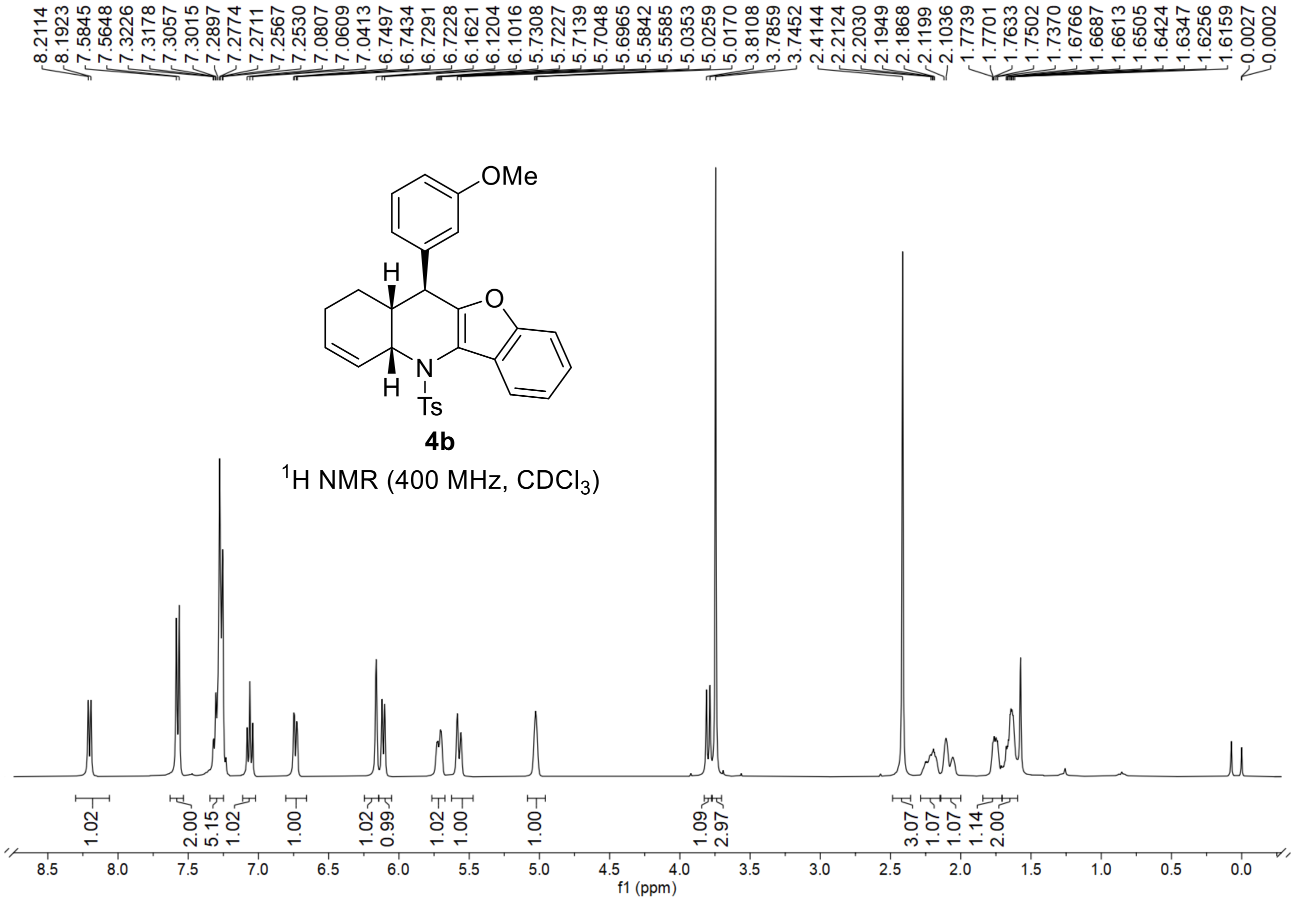
8.2114
8.1923
7.5845
7.5648
7.3226
7.3178
7.3057
7.3015
7.2897
7.2774
7.2711
7.2567
7.2530
7.0807
7.0609
7.0413
6.7497
6.7434
6.7291
6.7228
6.1621
6.1204
6.1016
5.7308
5.7227
5.7139
5.7048
5.6965
5.5842
5.5585
5.0353
5.0259
5.0170
3.8108
3.7859
3.7452
2.4144
2.2124
2.2030
2.1949
2.1868
2.1199
2.1036
1.7739
1.7701
1.7633
1.7502
1.7370
1.6766
1.6687
1.6613
1.6505
1.6424
1.6347
1.6256
1.6159
0.0027
0.0002
OMe
H
O
N
H
Ts
4b
^{1}H NMR (400 MHz, CDCl$_3$)
1.02
2.00
5.15
1.02
1.00
1.02
0.99
1.02
1.00
1.00
1.09
2.97
3.07
1.07
1.07
1.14
2.00
f1 (ppm)

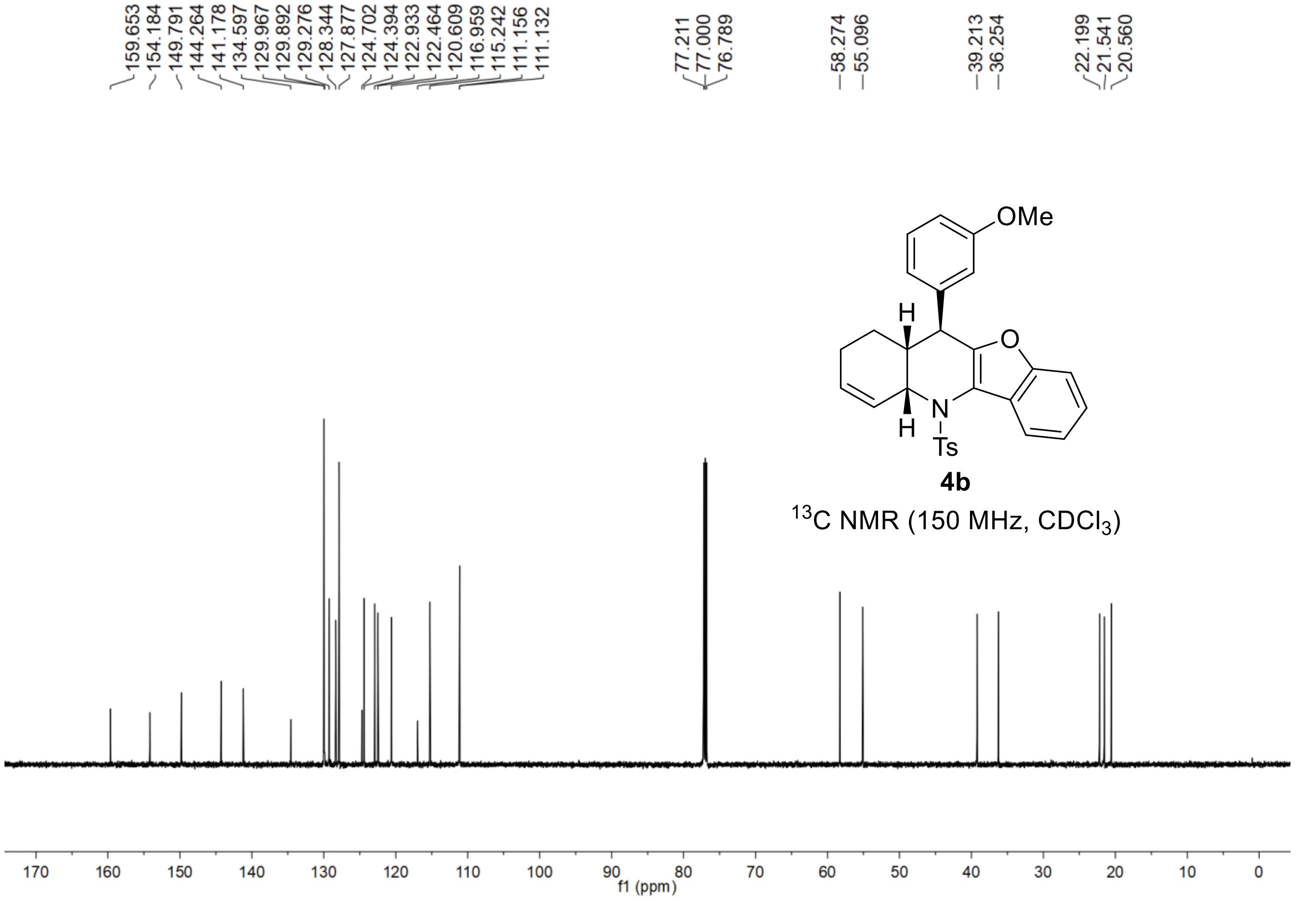
159.653
154.184
149.791
144.264
141.178
134.597
129.967
129.892
129.276
128.344
127.877
124.702
124.394
122.933
122.464
120.609
116.959
115.242
111.156
111.132
77.211
77.000
76.789
58.274
55.096
39.213
36.254
22.199
21.541
20.560
OMe
H
O
N
H
Ts
4b
^{13}C NMR (150 MHz, CDCl$_3$)
f1 (ppm)

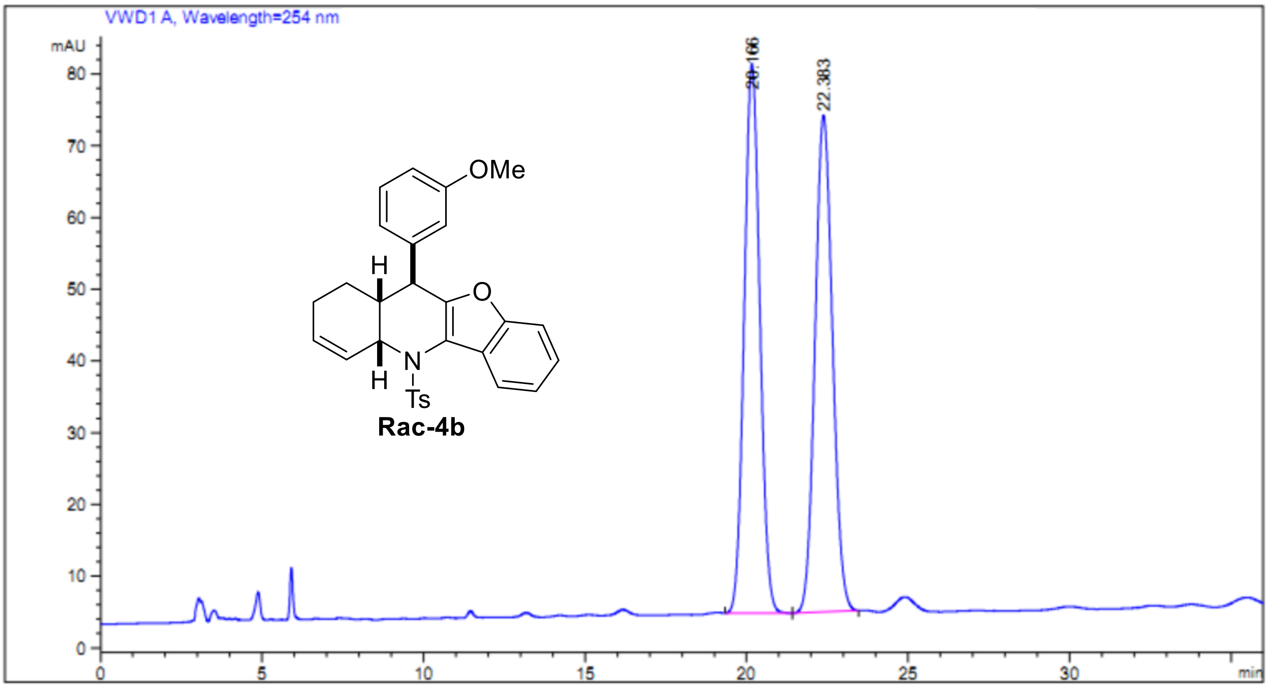

#	[min]		[min]	[mAU*s]	[mAU]	%
1	20.166	BB	0.5234	2574.43481	76.55724	49.6797
2	22.383	BB	0.5908	2607.63110	69.23320	50.3203

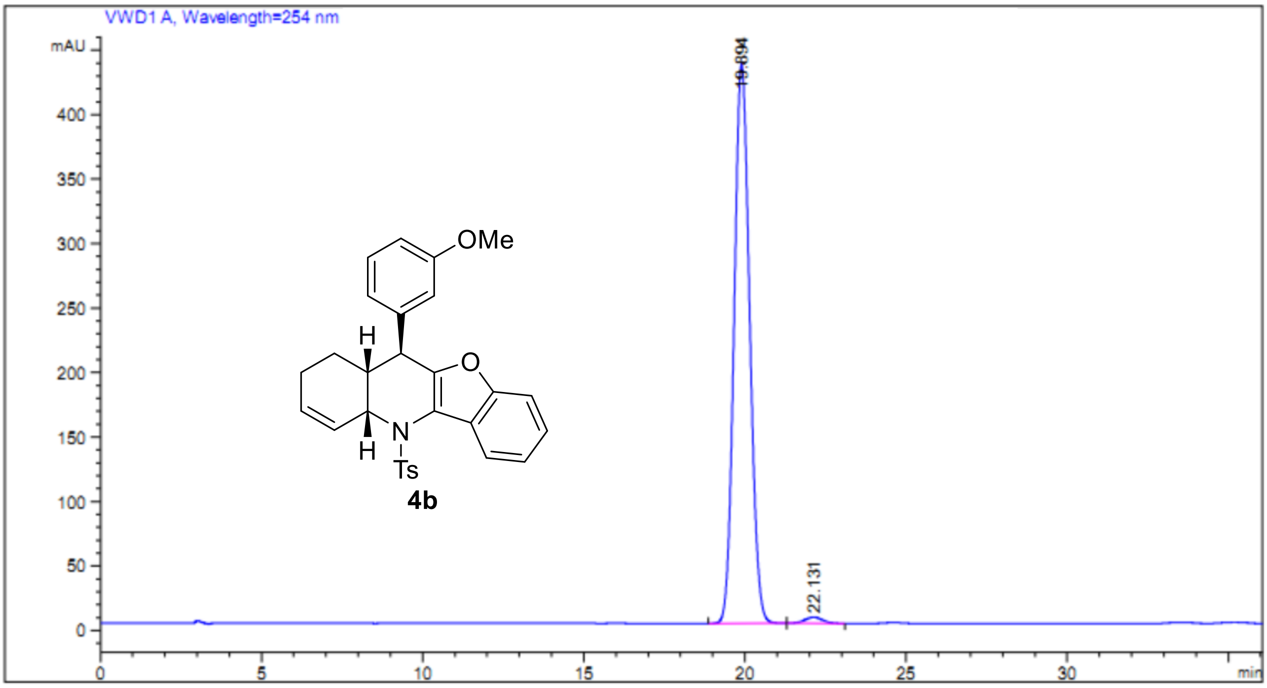

#	[min]		[min]	[mAU*s]	[mAU]	%
1	19.894	BB	0.5217	1.45212e4	433.71042	98.7962
2	22.131	BB	0.5673	176.94371	4.73173	1.2038

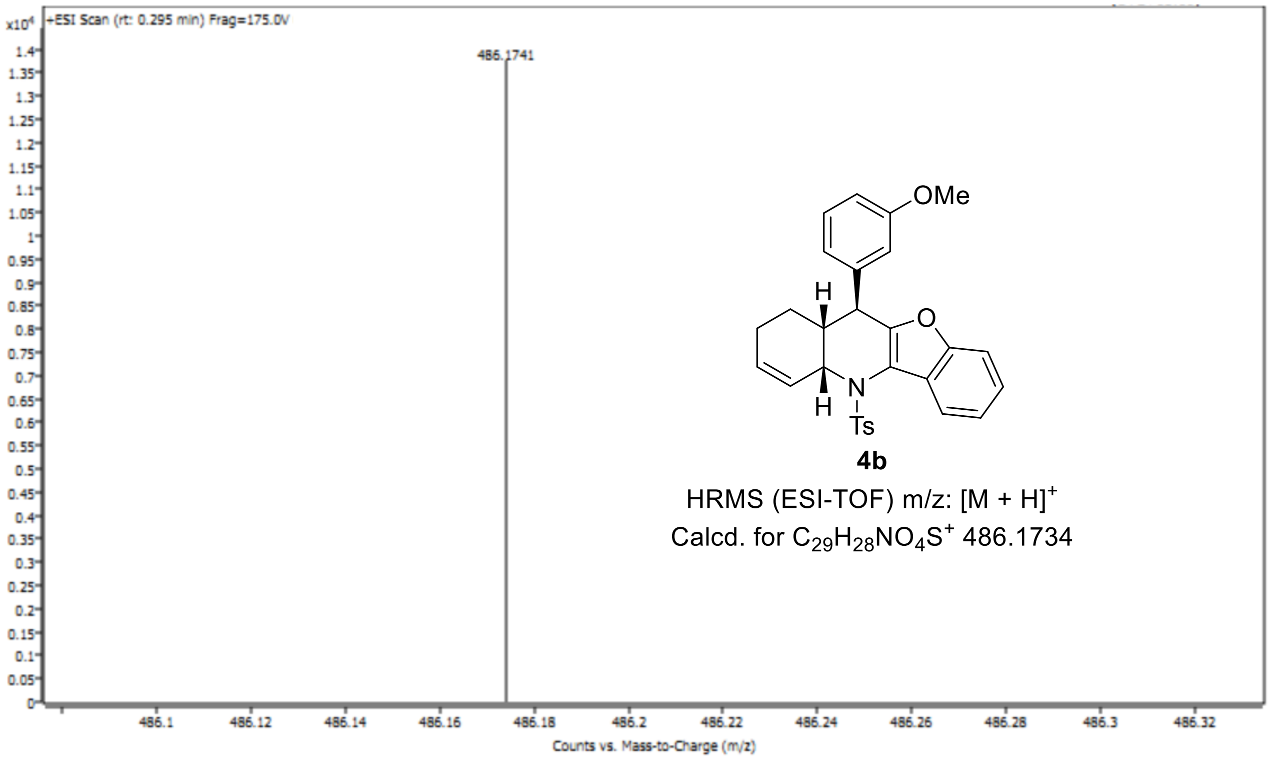
x10^4
+ESI Scan (rt: 0.295 min) Frag=175.0V
486.1741
OMe
H
O
N
H
Ts
4b
HRMS (ESI-TOF) m/z: [M + H]+
Calcd. for C29H28NO4S+ 486.1734
Counts vs. Mass-to-Charge (m/z)

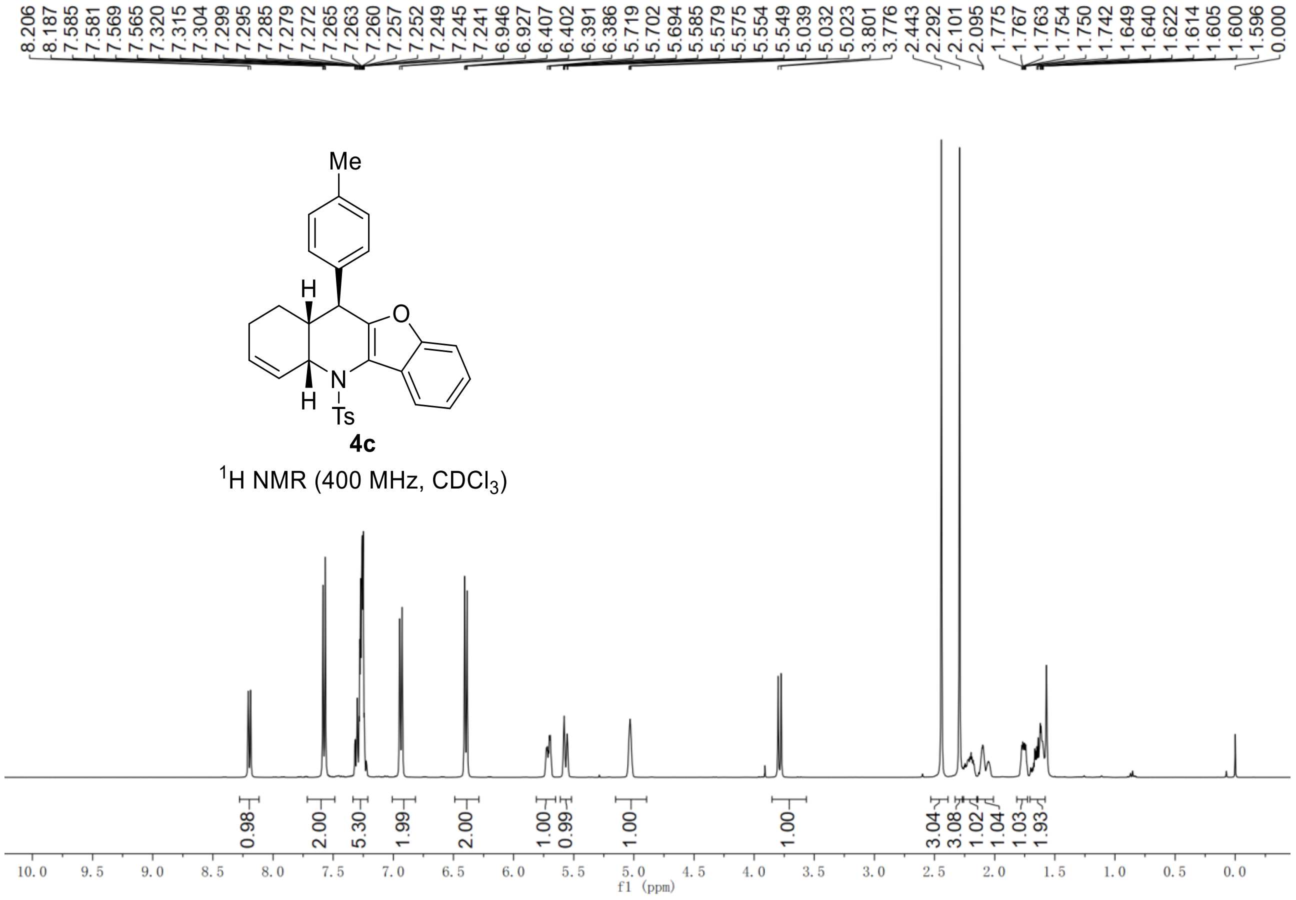
4c
^{1}H NMR (400 MHz, CDCl$_3$)
f1 (ppm)

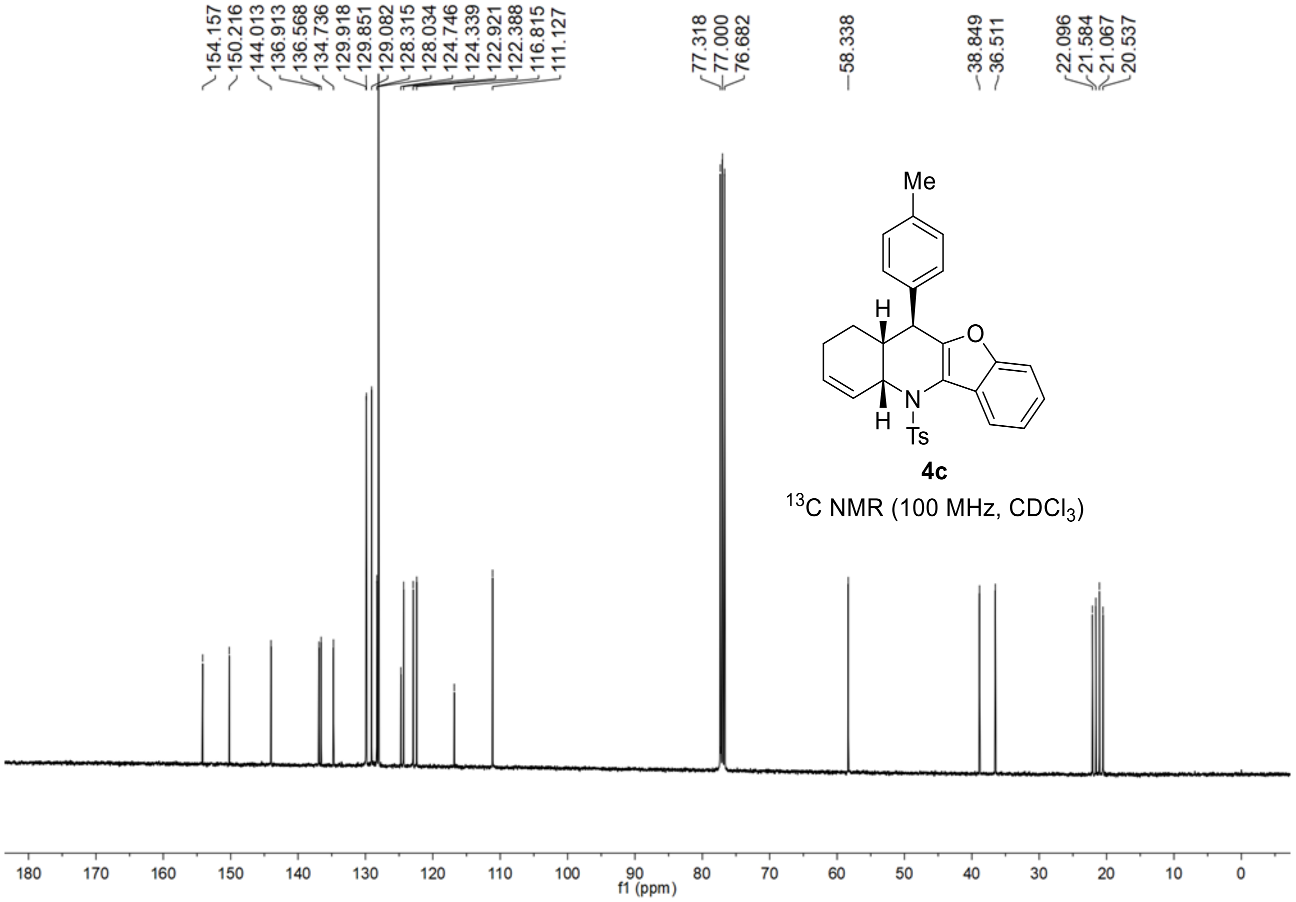
4c
^{13}C NMR (100 MHz, CDCl$_3$)
f1 (ppm)

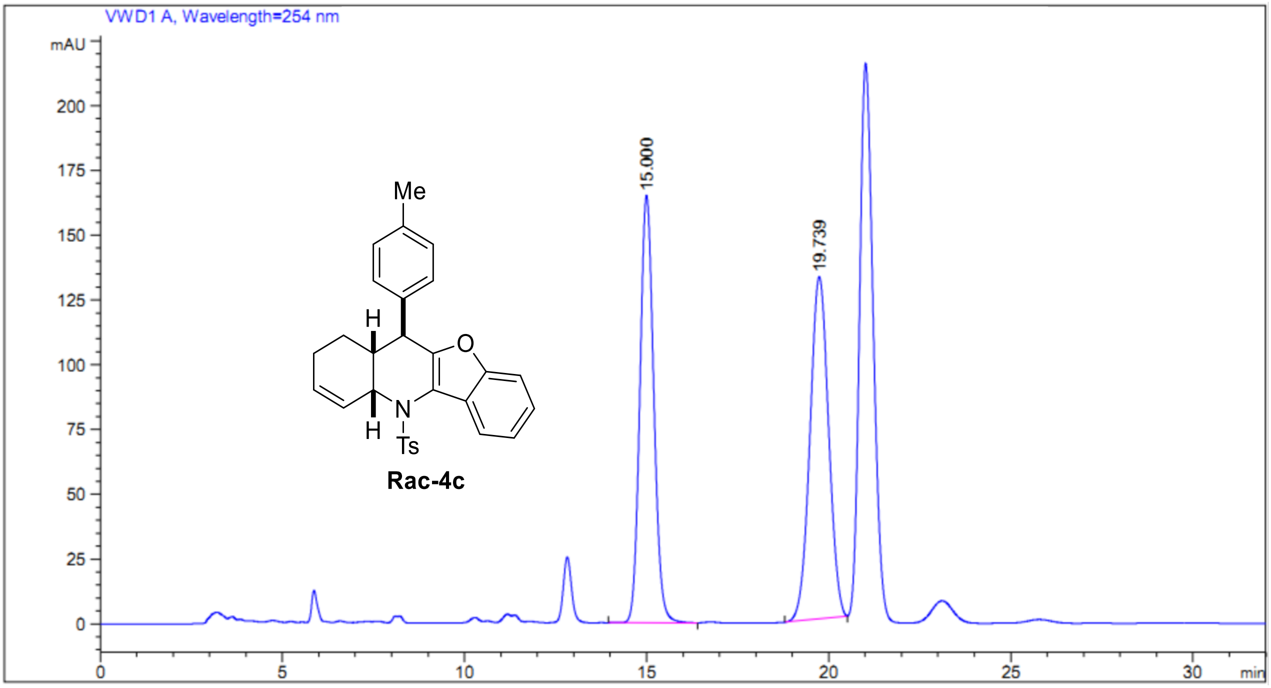

#	[min]		[min]	[mAU*s]	[mAU]	%
1	15.000	BV	0.4098	4355.52246	165.03610	48.0408
2	19.739	BB	0.5558	4710.77832	132.09181	51.9592

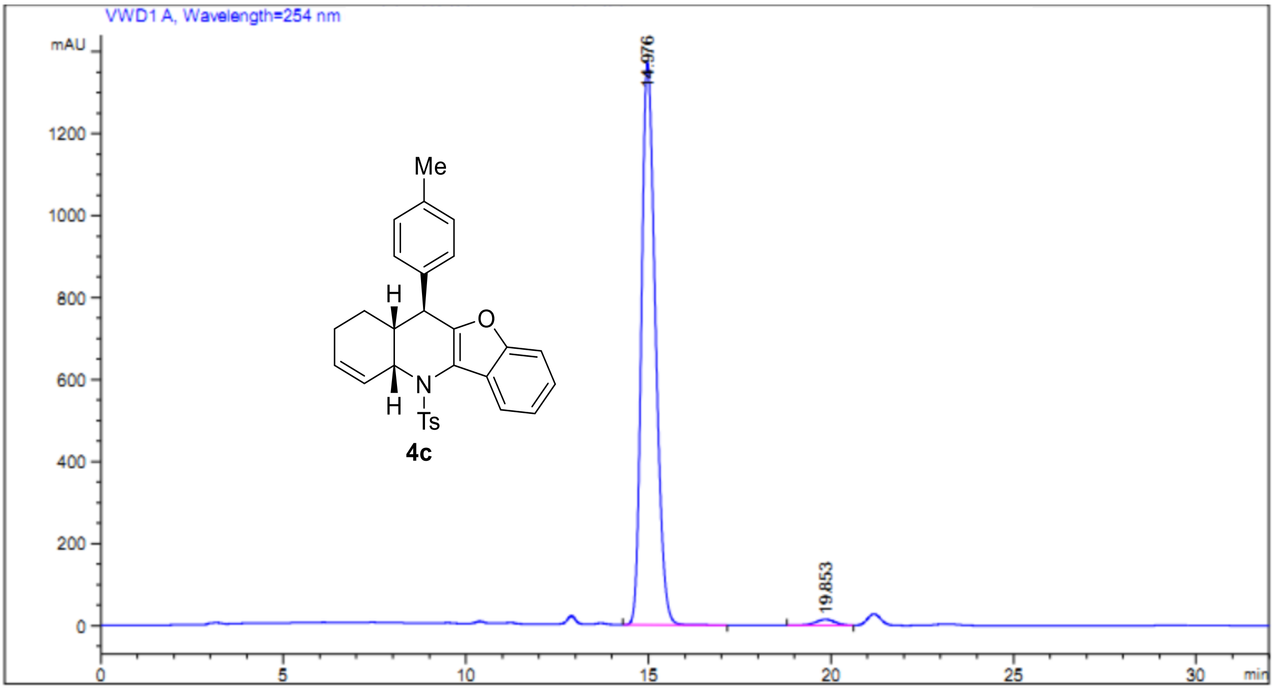

#	[min]		[min]	[mAU*s]	[mAU]	%
1	14.976	BBA	0.4141	3.63380e4	1370.75256	98.5370
2	19.853	BB	0.5811	539.50513	14.45436	1.4630

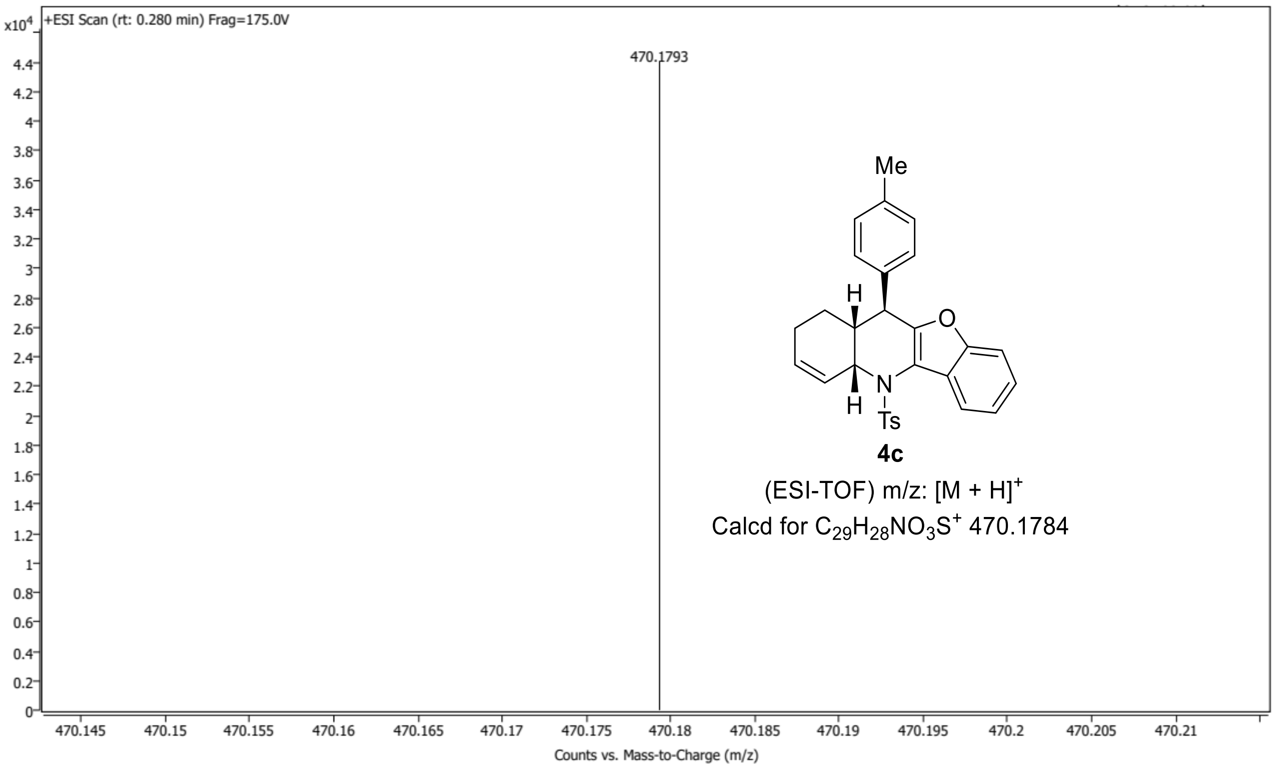

4c

(ESI-TOF) m/z: $[M + H]^+$

Calcd for $C_{29}H_{28}NO_3S^+$ 470.1784

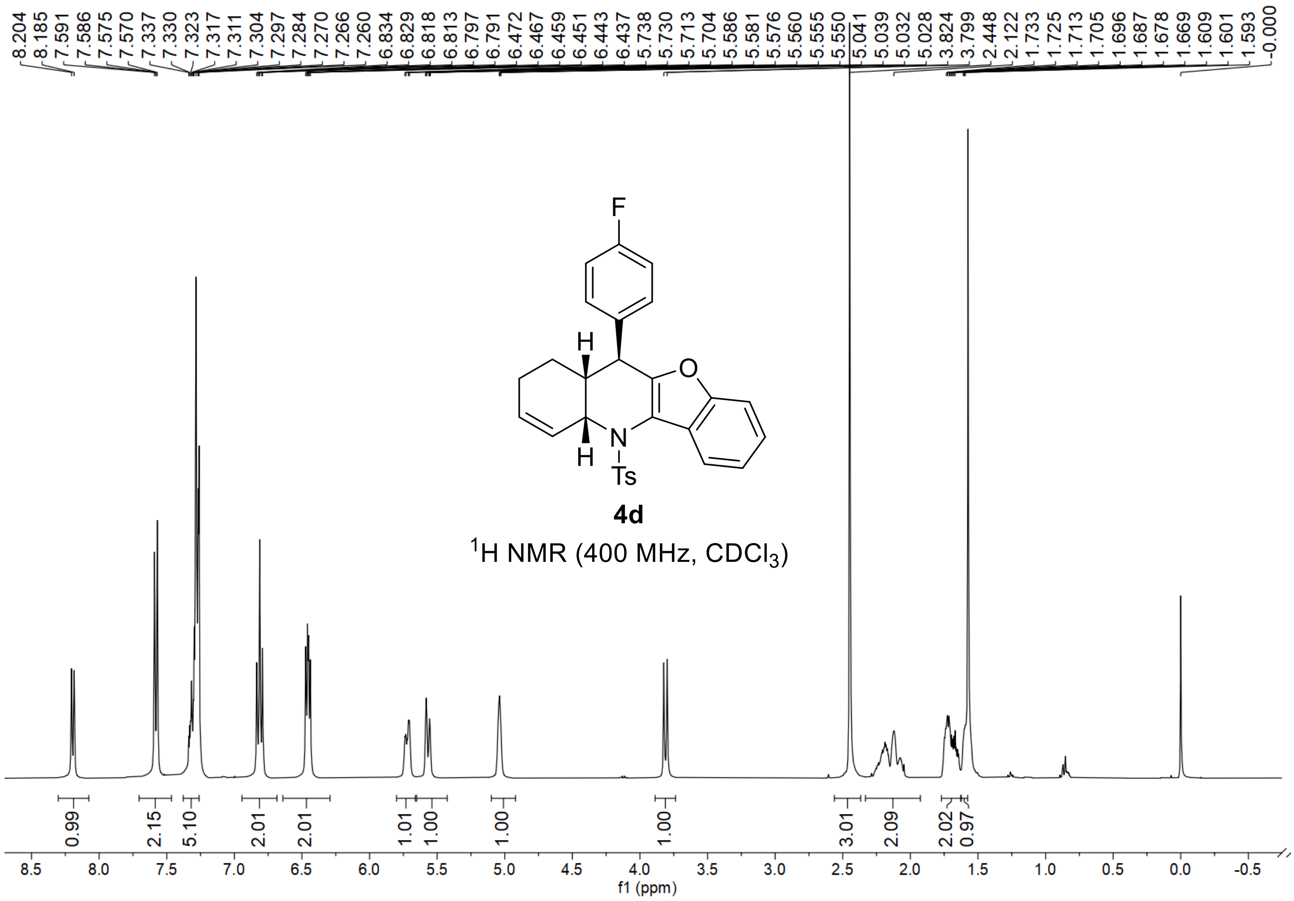
8.204
8.185
7.591
7.586
7.575
7.570
7.337
7.330
7.323
7.317
7.311
7.304
7.297
7.284
7.270
7.266
7.260
6.834
6.829
6.818
6.813
6.797
6.791
6.472
6.467
6.459
6.451
6.443
6.437
5.738
5.730
5.713
5.704
5.586
5.581
5.576
5.560
5.555
5.550
5.041
5.039
5.032
5.028
3.824
3.799
2.448
2.122
1.733
1.725
1.713
1.705
1.696
1.687
1.678
1.669
1.609
1.601
1.593
-0.000
F
H
O
H
N
Ts
4d
1H NMR (400 MHz, CDCl3)
0.99
2.15
5.10
2.01
2.01
1.01
1.00
1.00
1.00
3.01
2.09
2.02
0.97
f1 (ppm)

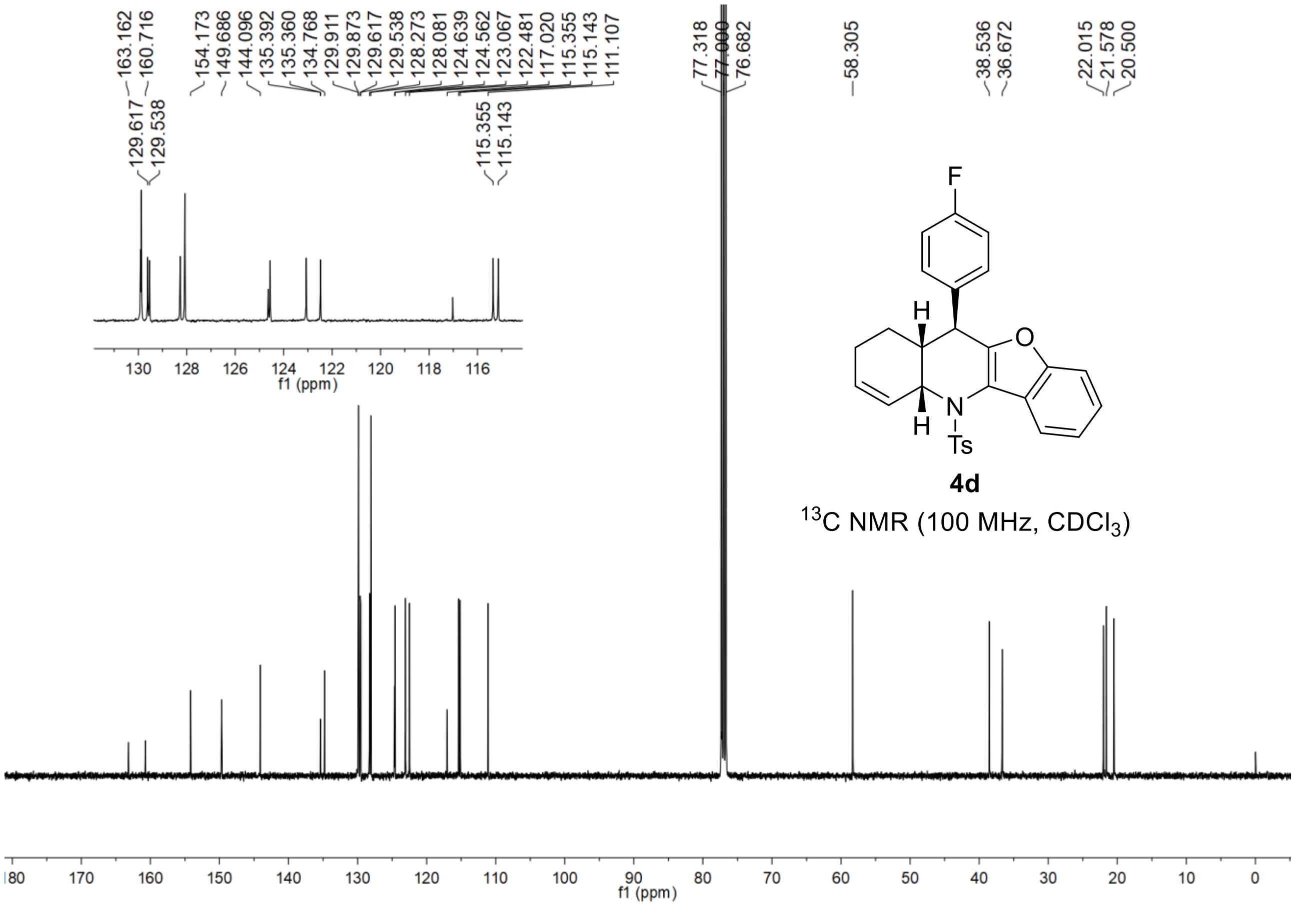
163.162
160.716
154.173
149.686
144.096
135.392
135.360
134.768
129.911
129.873
129.617
129.538
128.273
128.081
124.639
124.562
123.067
122.481
117.020
115.355
115.143
111.107
77.318
77.000
76.682
58.305
38.536
36.672
22.015
21.578
20.500
129.617
129.538
115.355
115.143
f1 (ppm)
F
H
O
H
N
Ts
4d
13C NMR (100 MHz, CDCl3)
f1 (ppm)

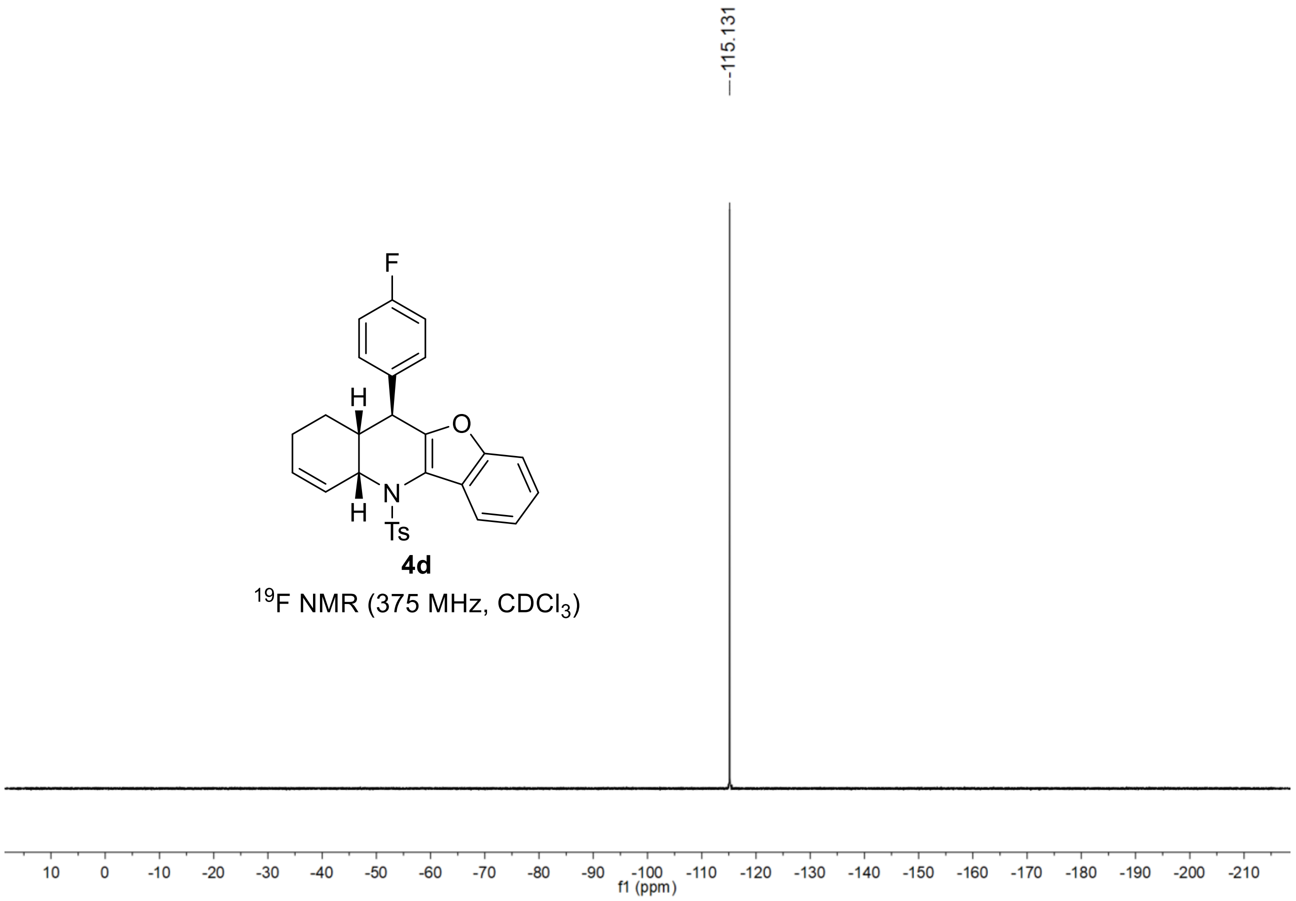
-115.131
F
H
O
N
H
Ts
4d
19F NMR (375 MHz, CDCl3)
10 0 -10 -20 -30 -40 -50 -60 -70 -80 -90 -100 -110 -120 -130 -140 -150 -160 -170 -180 -190 -200 -210
f1 (ppm)

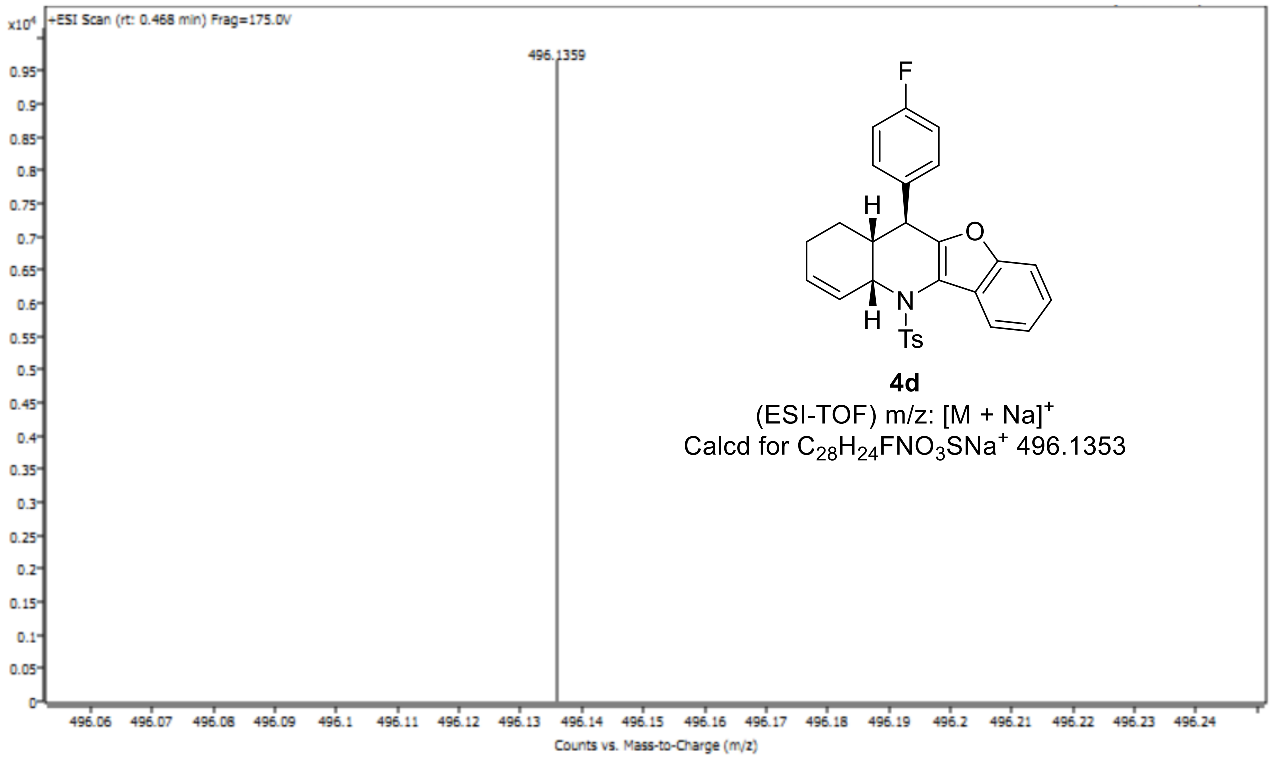
x10^4
+ESI Scan (rt: 0.468 min) Frag=175.0V
496.1359
F
H
O
N
H
Ts
4d
(ESI-TOF) m/z: [M + Na]+
Calcd for C28H24FNO3SNa+ 496.1353
Counts vs. Mass-to-Charge (m/z)

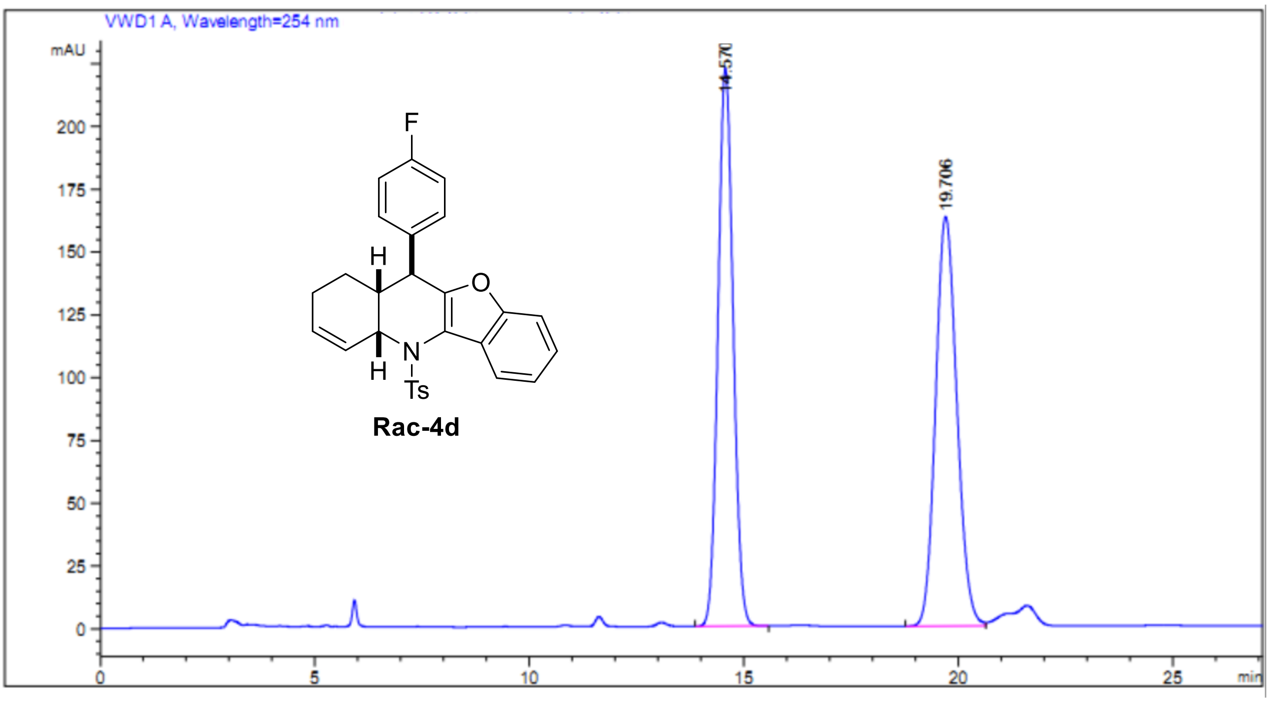

#	[min]		[min]	[mAU*s]	[mAU]	%
1	14.570	BB	0.3831	5466.55225	222.21568	49.2973
2	19.706	BV	0.5362	5622.40283	163.07292	50.7027

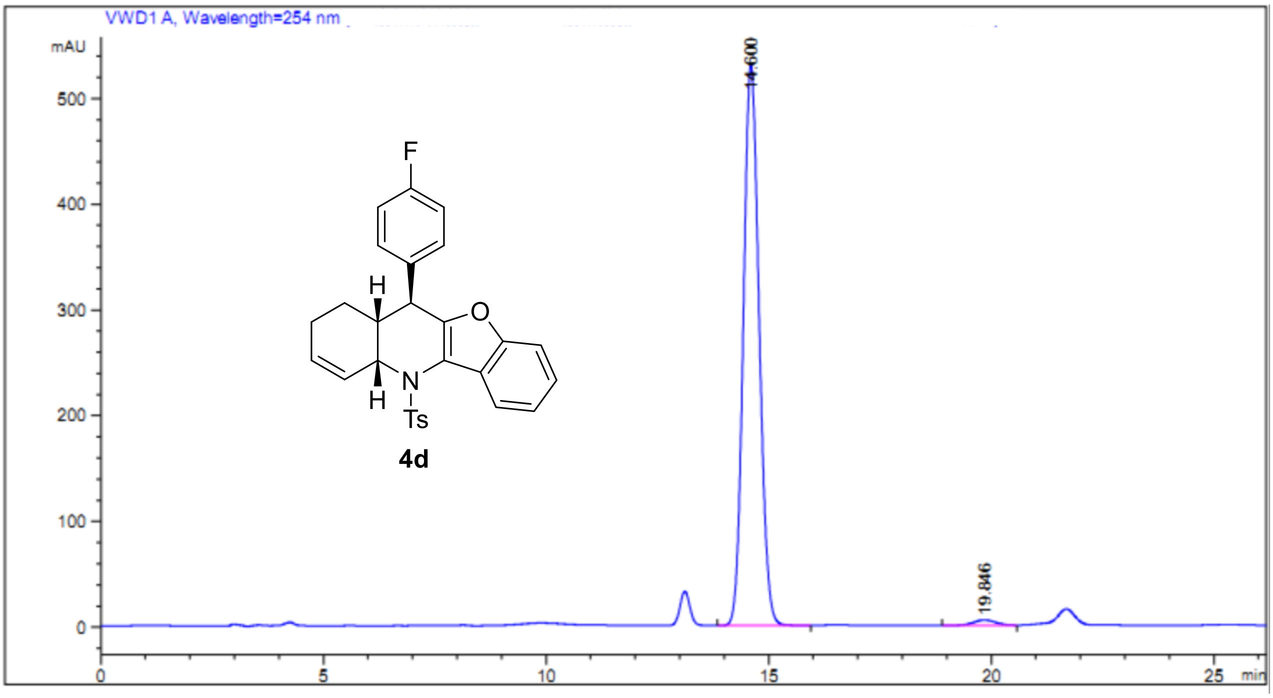

#	[min]		[min]	[mAU*s]	[mAU]	%
1	14.600	BB	0.3825	1.30177e4	530.24335	98.7264
2	19.846	BB	0.5215	167.93855	4.98060	1.2736

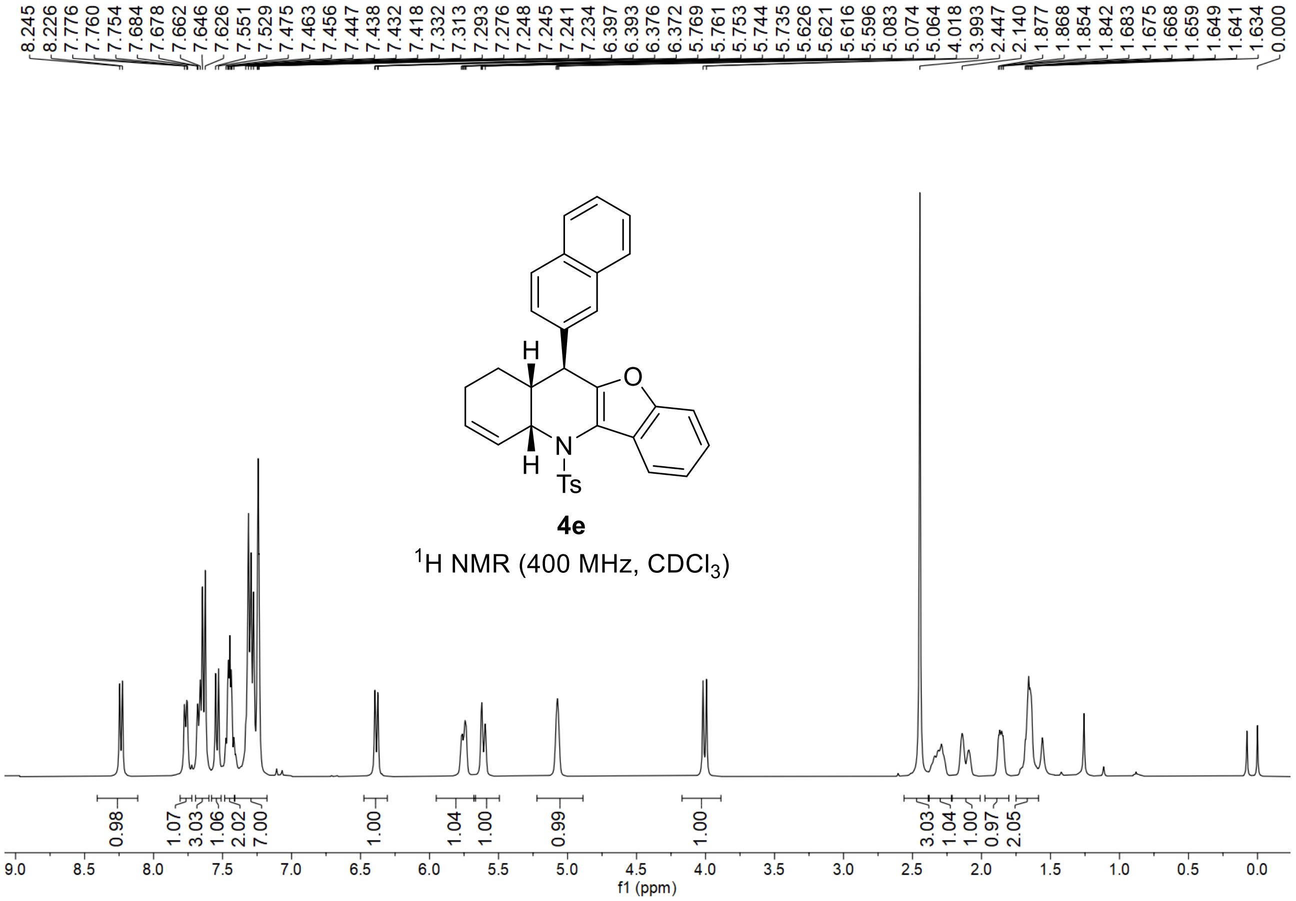
8.245 8.226 7.776 7.760 7.754 7.684 7.678 7.662 7.646 7.626 7.551 7.529 7.475 7.463 7.456 7.447 7.438 7.432 7.418 7.332 7.313 7.293 7.276 7.248 7.245 7.241 7.234 6.397 6.393 6.376 6.372 5.769 5.761 5.753 5.744 5.735 5.626 5.621 5.616 5.596 5.083 5.074 5.064 4.018 3.993 2.447 2.140 1.877 1.868 1.854 1.842 1.683 1.675 1.668 1.659 1.649 1.641 1.634 0.000
H
O
H
N
Ts
4e
1H NMR (400 MHz, CDCl3)
0.98 1.07 3.03 1.06 2.02 7.00 1.00 1.04 1.00 0.99 1.00 3.03 1.04 1.00 0.97 2.05
9.0 8.5 8.0 7.5 7.0 6.5 6.0 5.5 5.0 4.5 4.0 3.5 3.0 2.5 2.0 1.5 1.0 0.5 0.0
f1 (ppm)

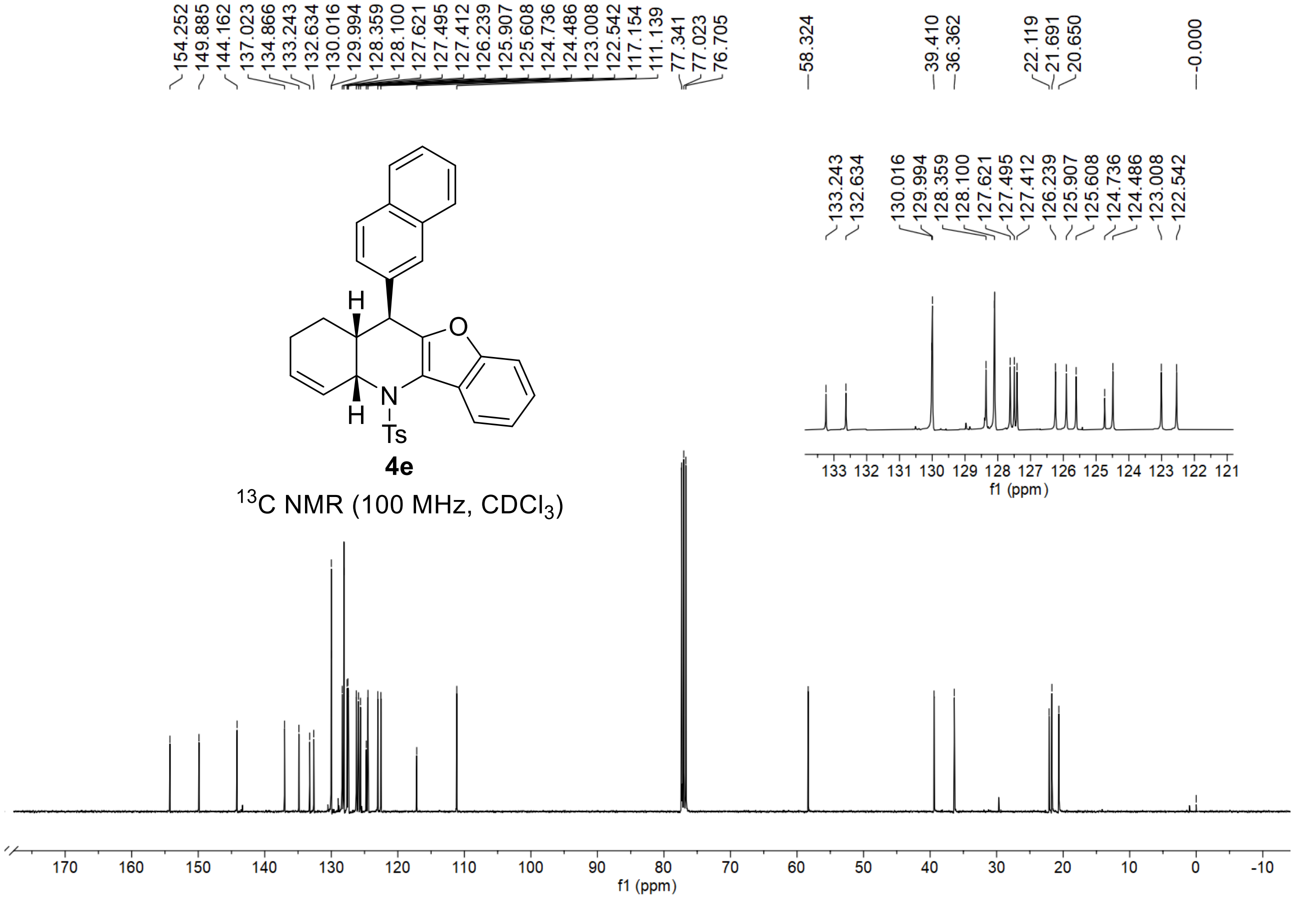
154.252 149.885 144.162 137.023 134.866 133.243 132.634 130.016 129.994 128.359 128.100 127.621 127.495 127.412 126.239 125.907 125.608 124.736 124.486 123.008 122.542 117.154 111.139 77.341 77.023 76.705 58.324 39.410 36.362 22.119 21.691 20.650 -0.000
133.243 132.634 130.016 129.994 128.359 128.100 127.621 127.495 127.412 126.239 125.907 125.608 124.736 124.486 123.008 122.542
133 132 131 130 129 128 127 126 125 124 123 122 121
f1 (ppm)
H
O
H
N
Ts
4e
13C NMR (100 MHz, CDCl3)
170 160 150 140 130 120 110 100 90 80 70 60 50 40 30 20 10 0 -10
f1 (ppm)

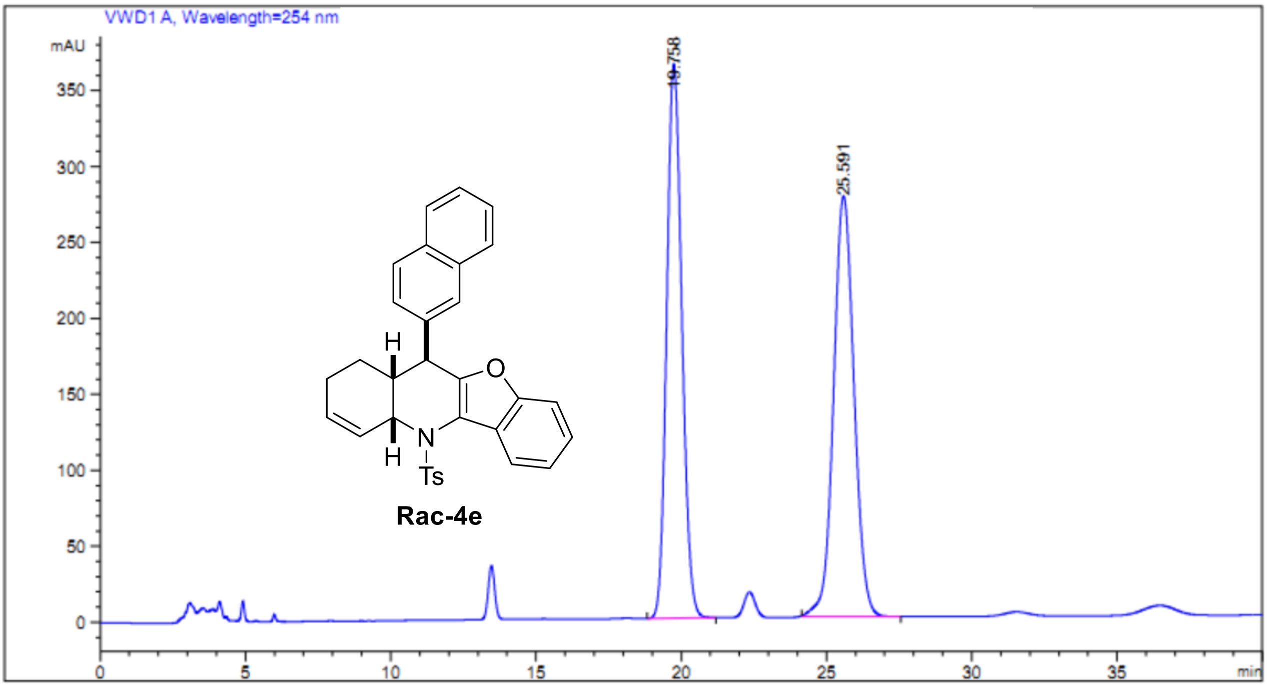

#	[min]		[min]	[mAU*s]	[mAU]	%
1	19.758	BB	0.6224	1.31983e4	353.13330	49.3258
2	25.591	BB	0.7636	1.35591e4	274.05035	50.6742

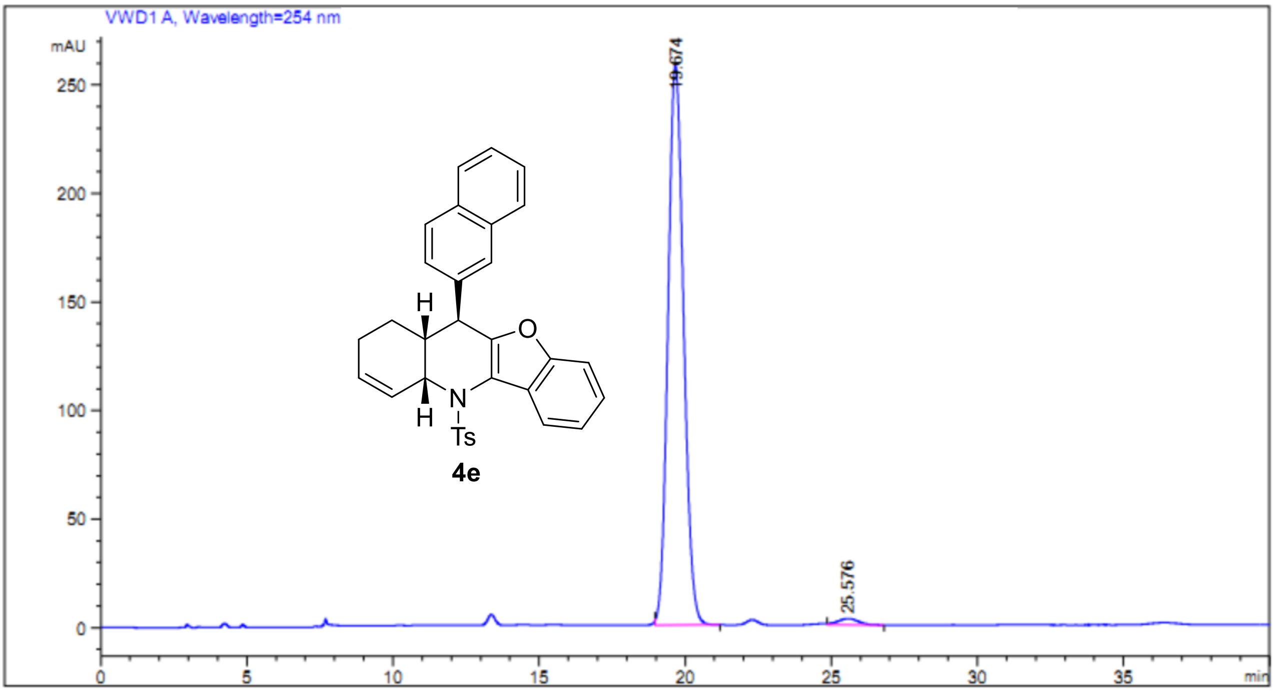

#	[min]		[min]	[mAU*s]	[mAU]	%
1	19.674	BB	0.6251	9551.05762	254.00978	98.5868
2	25.576	BB	0.7551	136.90572	2.80865	1.4132

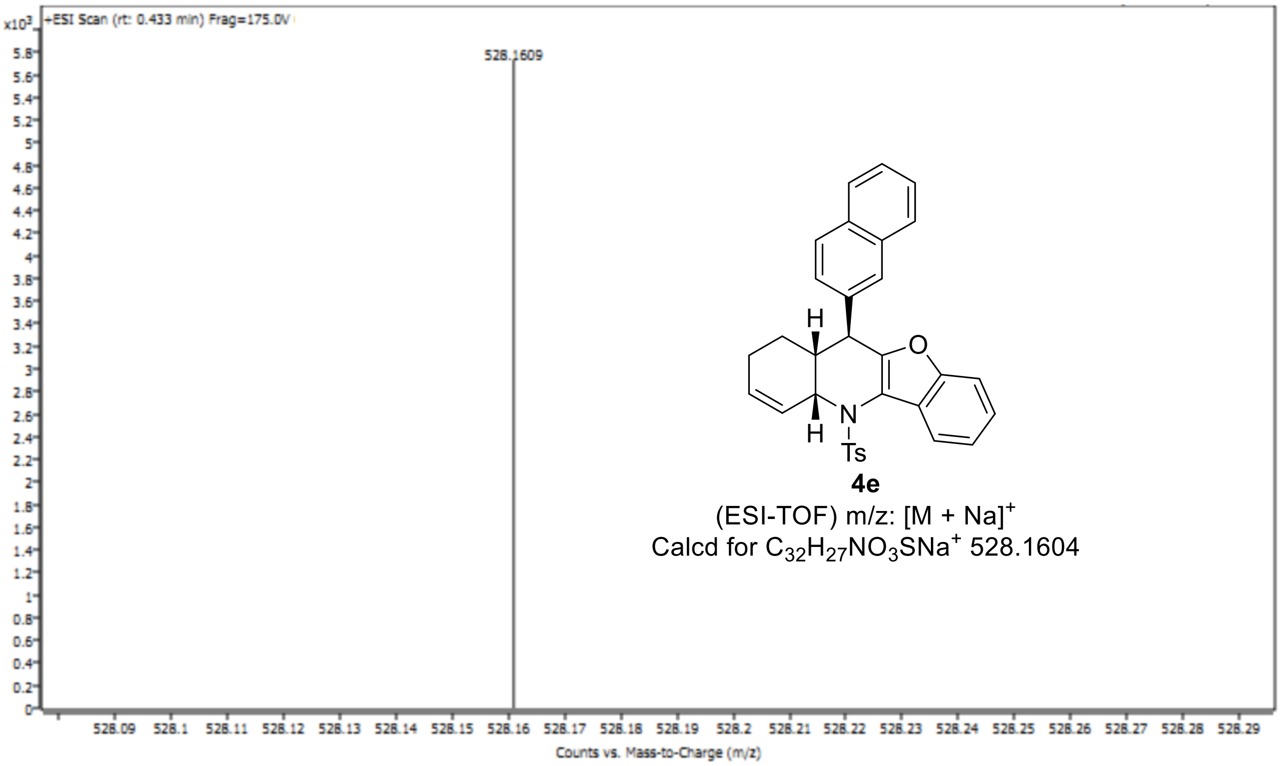
x10^3
+ESI Scan (rt: 0.433 min) Frag=175.0V
528.1609
H
H
O
N
Ts
4e
(ESI-TOF) m/z: [M + Na]+
Calcd for C32H27NO3SNa+ 528.1604
Counts vs. Mass-to-Charge (m/z)

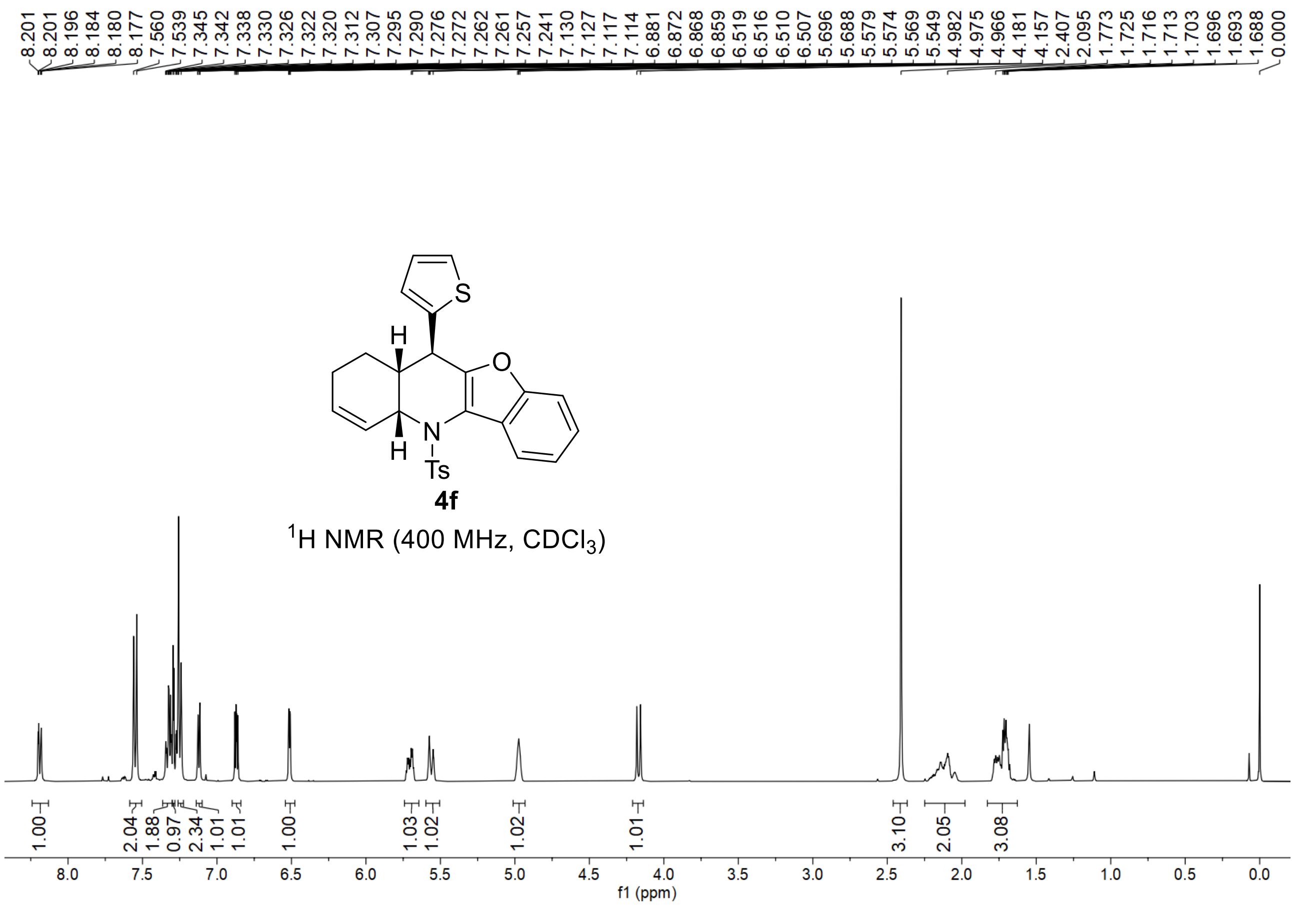
8.201 8.201 8.196 8.184 8.180 8.177 7.560 7.539 7.345 7.342 7.338 7.330 7.326 7.322 7.320 7.312 7.307 7.295 7.290 7.276 7.272 7.262 7.261 7.257 7.241 7.130 7.127 7.117 7.114 6.881 6.872 6.868 6.859 6.519 6.516 6.510 6.507 5.696 5.688 5.579 5.574 5.569 5.549 4.982 4.975 4.966 4.181 4.157 2.407 2.095 1.773 1.725 1.716 1.713 1.703 1.696 1.693 1.688 0.000
S
H
O
N
H
Ts
4f
1H NMR (400 MHz, CDCl3)
1.00 2.04 1.88 0.97 2.34 1.01 1.01 1.00 1.03 1.02 1.02 1.01 3.10 2.05 3.08
f1 (ppm)

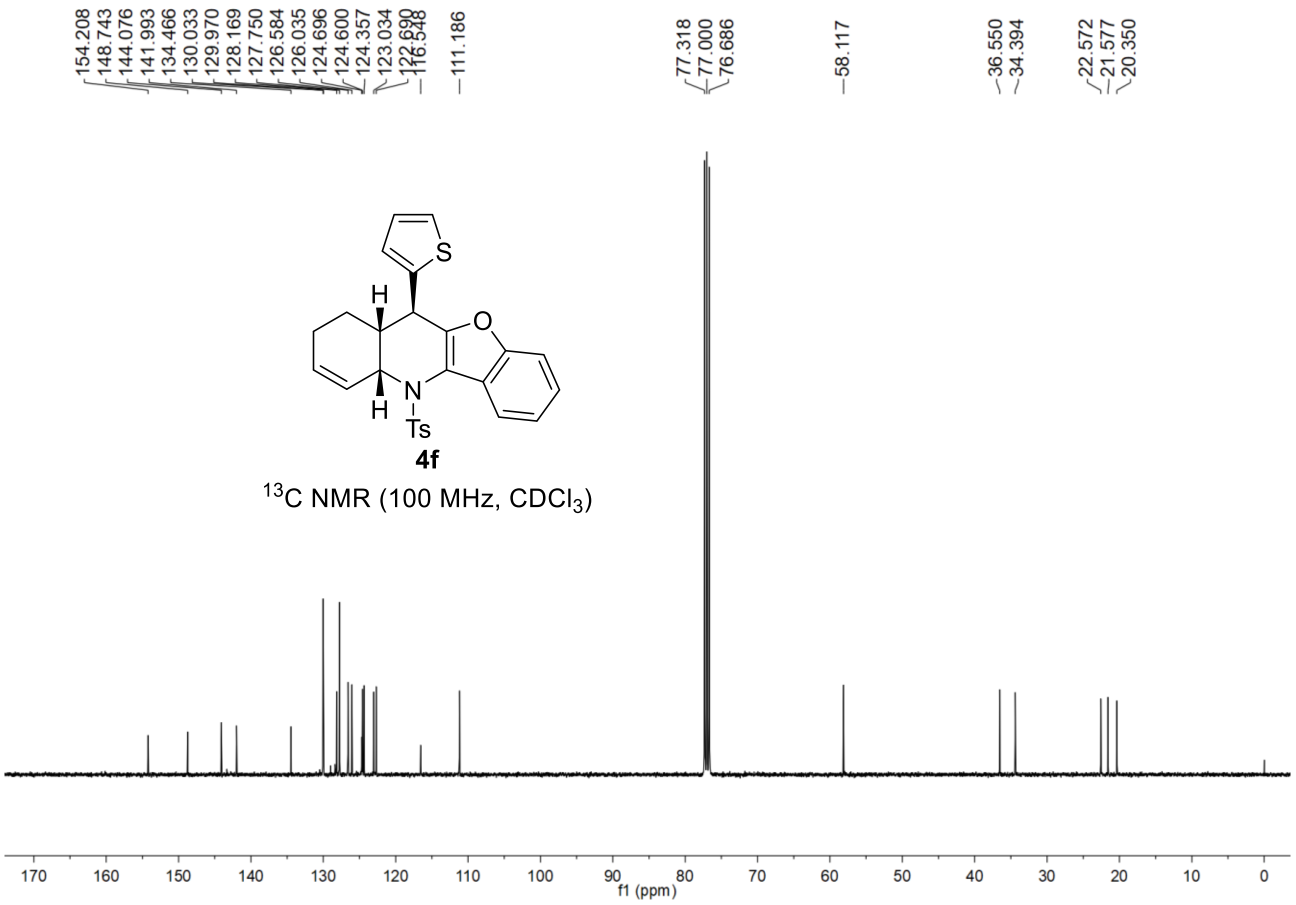
154.208 148.743 144.076 141.993 134.466 130.033 129.970 128.169 127.750 126.584 126.035 124.696 124.600 124.357 123.034 116.548 111.186
77.318 77.000 76.686
58.117
36.550 34.394
22.572 21.577 20.350
S
H
O
N
H
Ts
4f
13C NMR (100 MHz, CDCl3)
f1 (ppm)

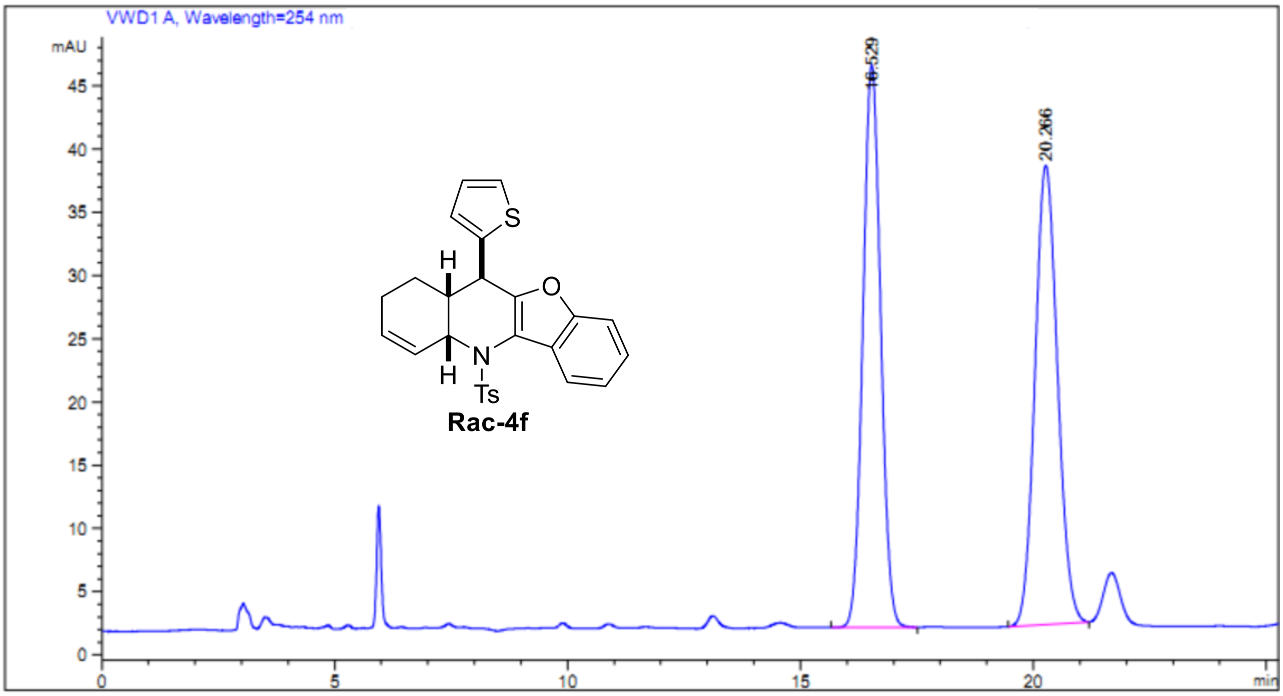

#	[min]		[min]	[mAU*s]	[mAU]	%
1	16.529	BB	0.4140	1178.56799	44.48122	49.1112
2	20.266	BB	0.5221	1221.22815	36.29995	50.8888

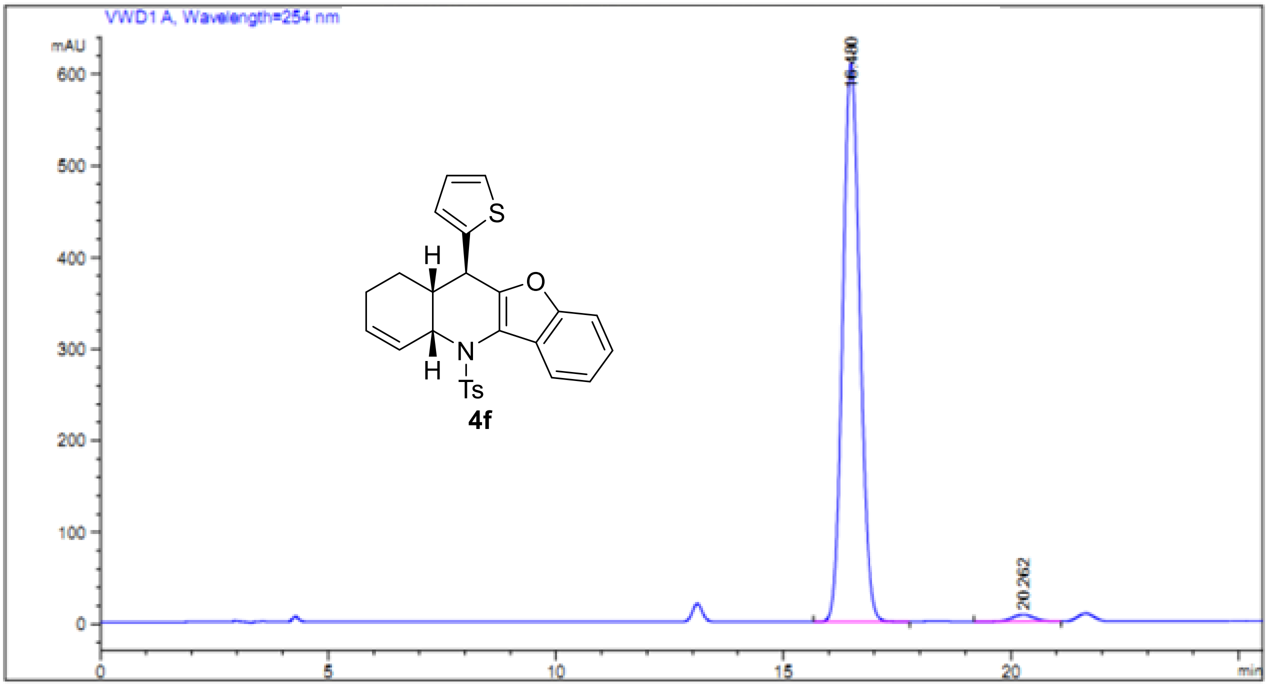

#	[min]		[min]	[mAU*s]	[mAU]	%
1	16.480	BB	0.4099	1.59952e4	608.75262	98.4334
2	20.262	BB	0.5349	254.57562	7.40803	1.5666

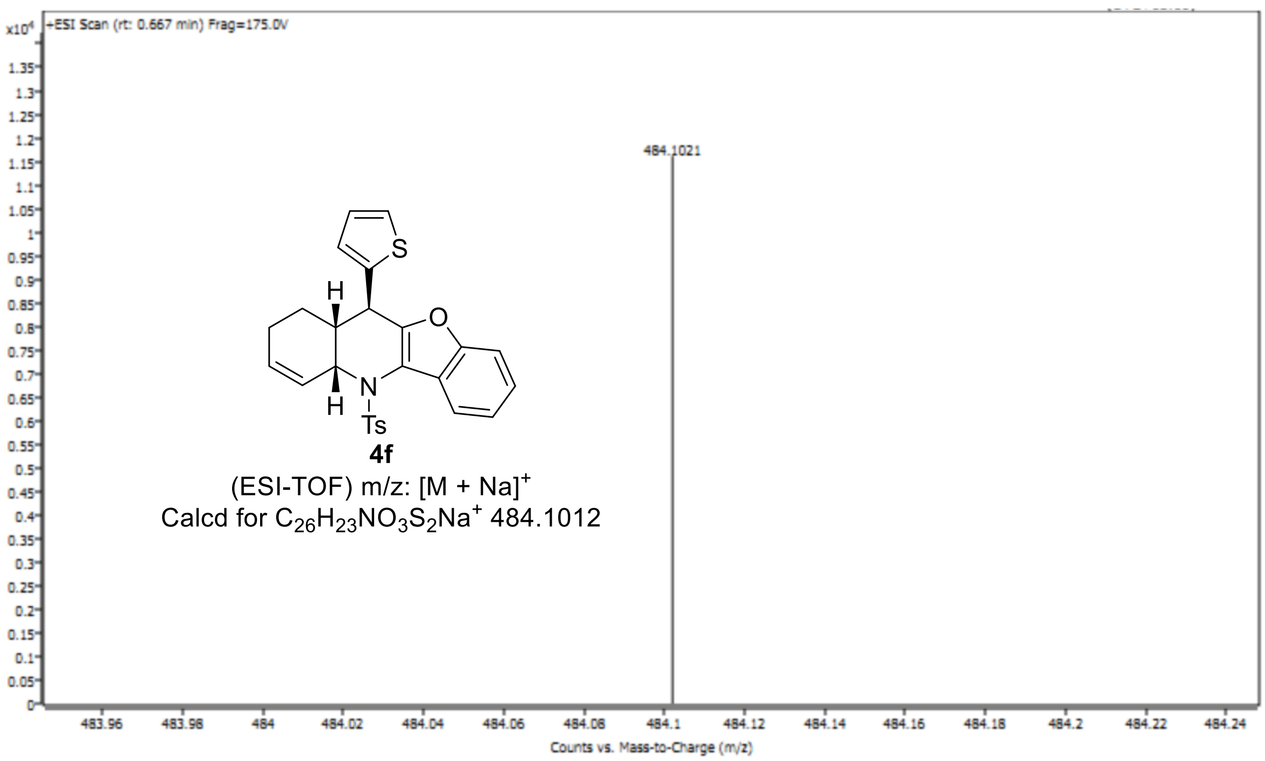
+ESI Scan (rt: 0.667 min) Frag=175.0V
x10^4
484.1021
S
H
O
N
H
Ts
4f
(ESI-TOF) m/z: [M + Na]+
Calcd for C26H23NO3S2Na+ 484.1012
Counts vs. Mass-to-Charge (m/z)

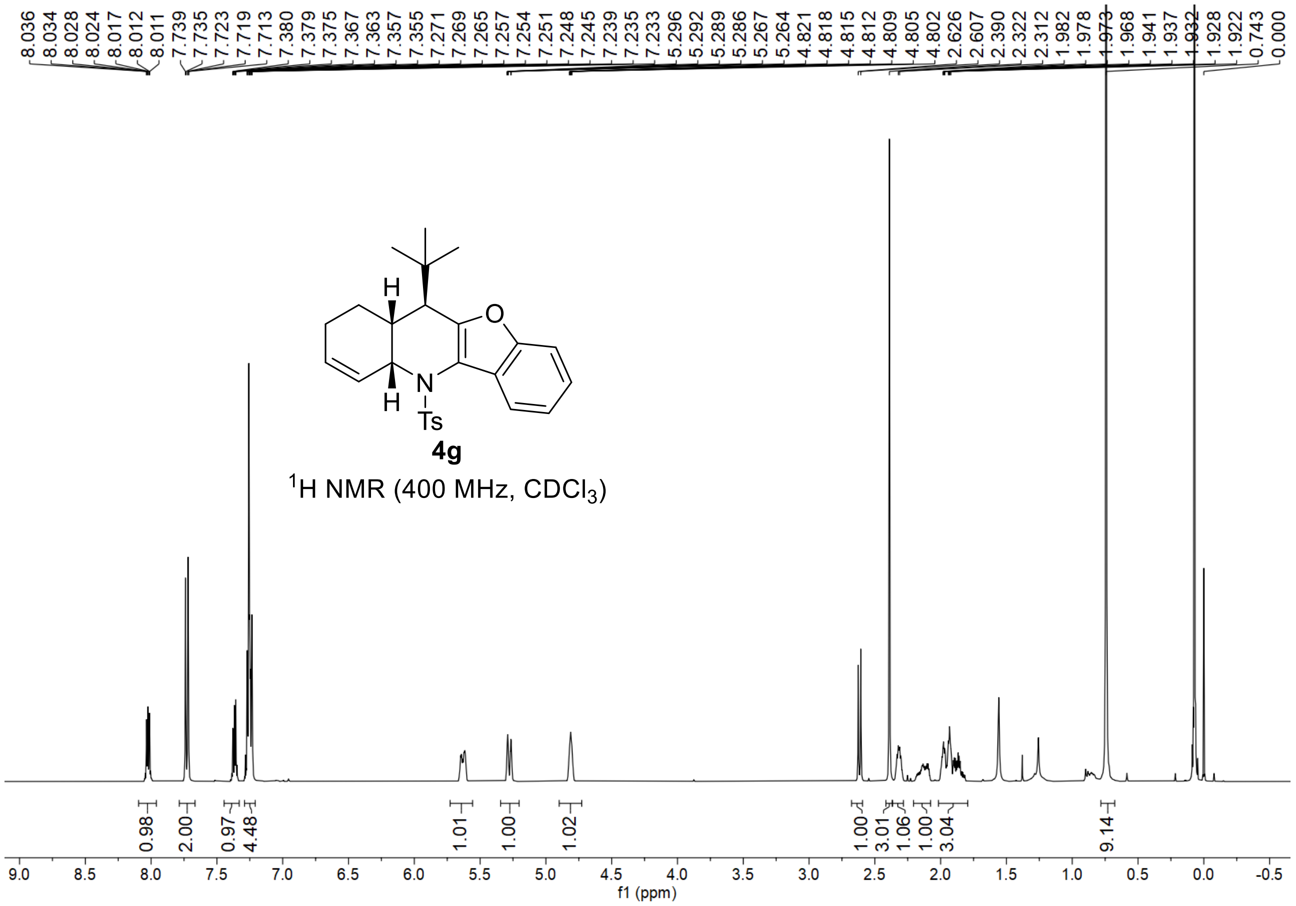

8.036
8.034
8.028
8.024
8.017
8.012
8.011
7.739
7.735
7.723
7.719
7.713
7.380
7.379
7.375
7.367
7.363
7.357
7.355
7.271
7.269
7.265
7.257
7.254
7.251
7.248
7.245
7.239
7.235
7.233
5.296
5.292
5.289
5.286
5.267
5.264
4.821
4.818
4.815
4.812
4.809
4.805
4.802
2.626
2.607
2.390
2.322
2.312
1.982
1.978
1.973
1.968
1.941
1.937
1.932
1.928
1.922
0.743
0.000
H
H
O
N
Ts
4g
1H NMR (400 MHz, CDCl3)
0.98
2.00
0.97
4.48
1.01
1.00
1.02
1.00
3.01
1.06
1.00
3.04
9.14
f1 (ppm)

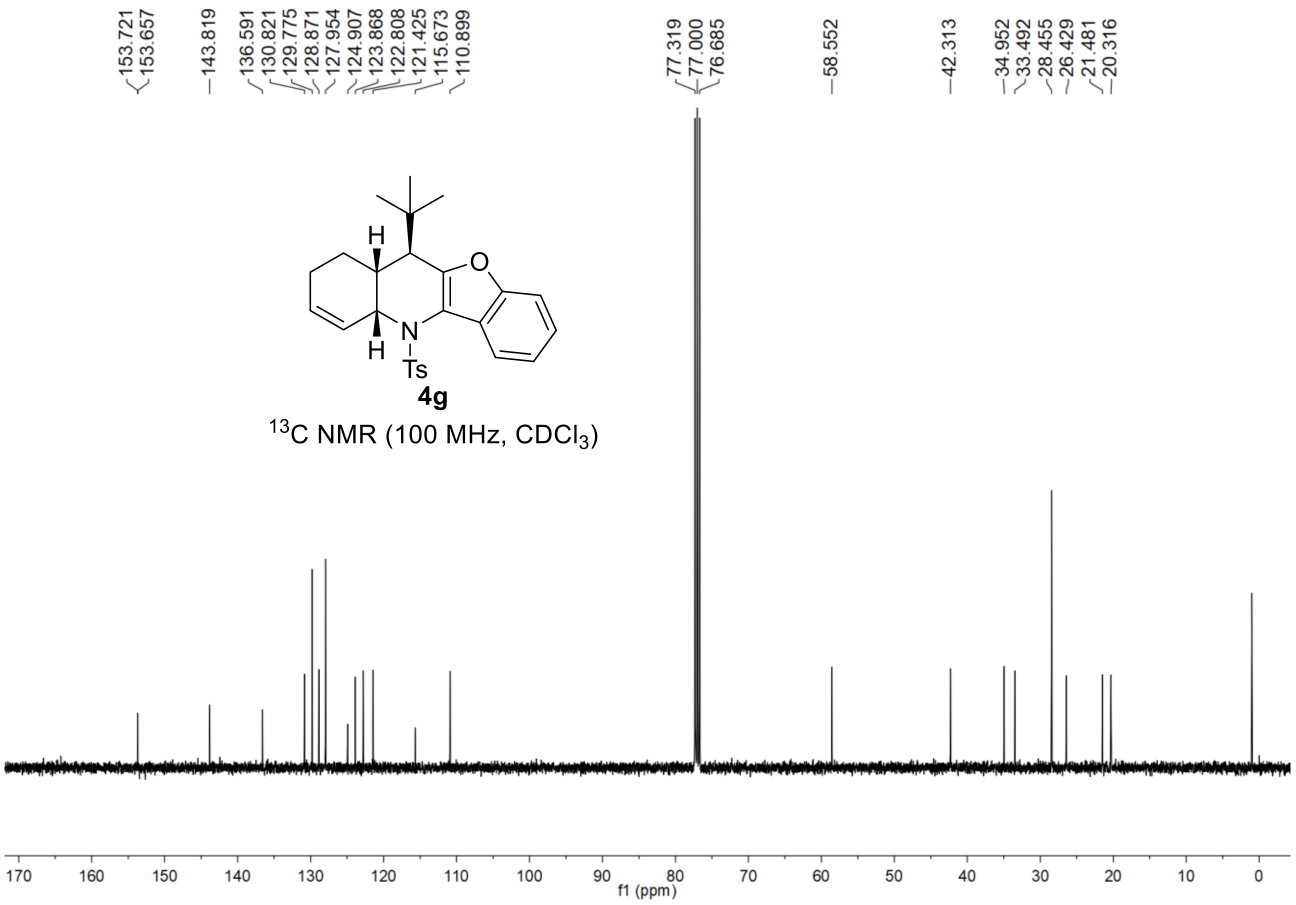

153.721
153.657
143.819
136.591
130.821
129.775
128.871
127.954
124.907
123.868
122.808
121.425
115.673
110.899
77.319
77.000
76.685
58.552
42.313
34.952
33.492
28.455
26.429
21.481
20.316
H
H
O
N
Ts
4g
13C NMR (100 MHz, CDCl3)
f1 (ppm)

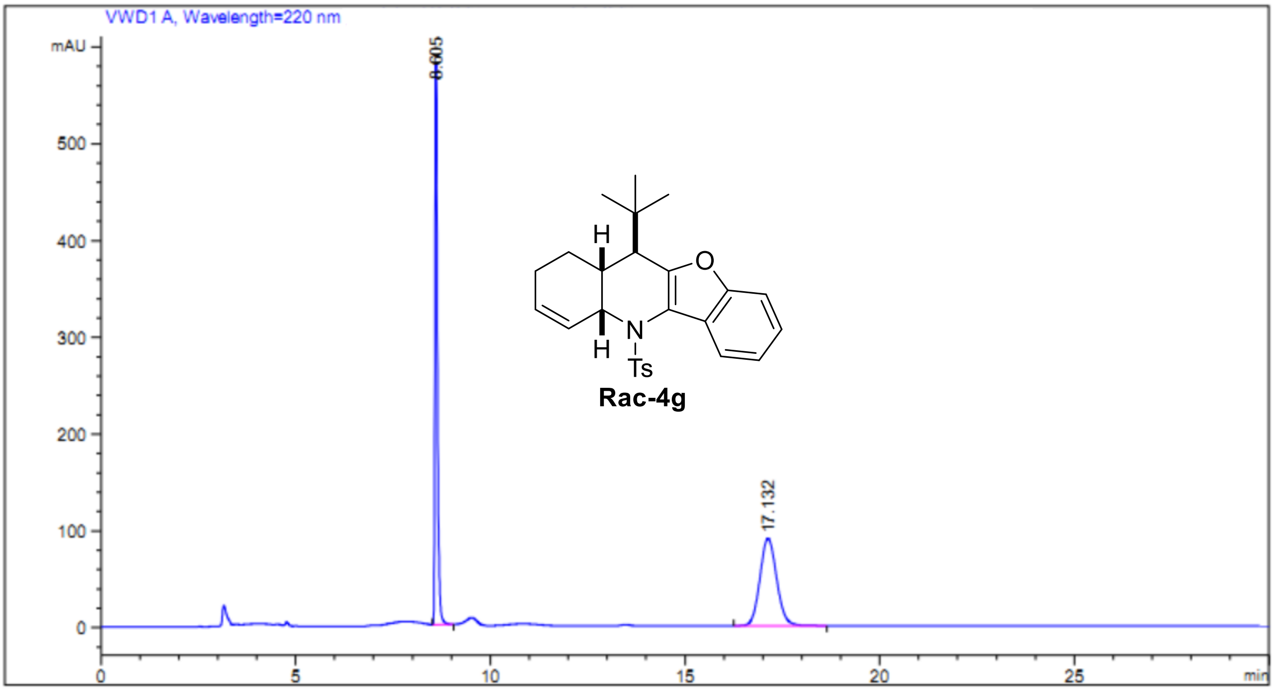

#	[min]		[min]	[mAU*s]	[mAU]	%
1	8.605	BB	0.0653	2517.97241	579.26654	48.3107
2	17.132	BB	0.4630	2694.06689	90.33281	51.6893

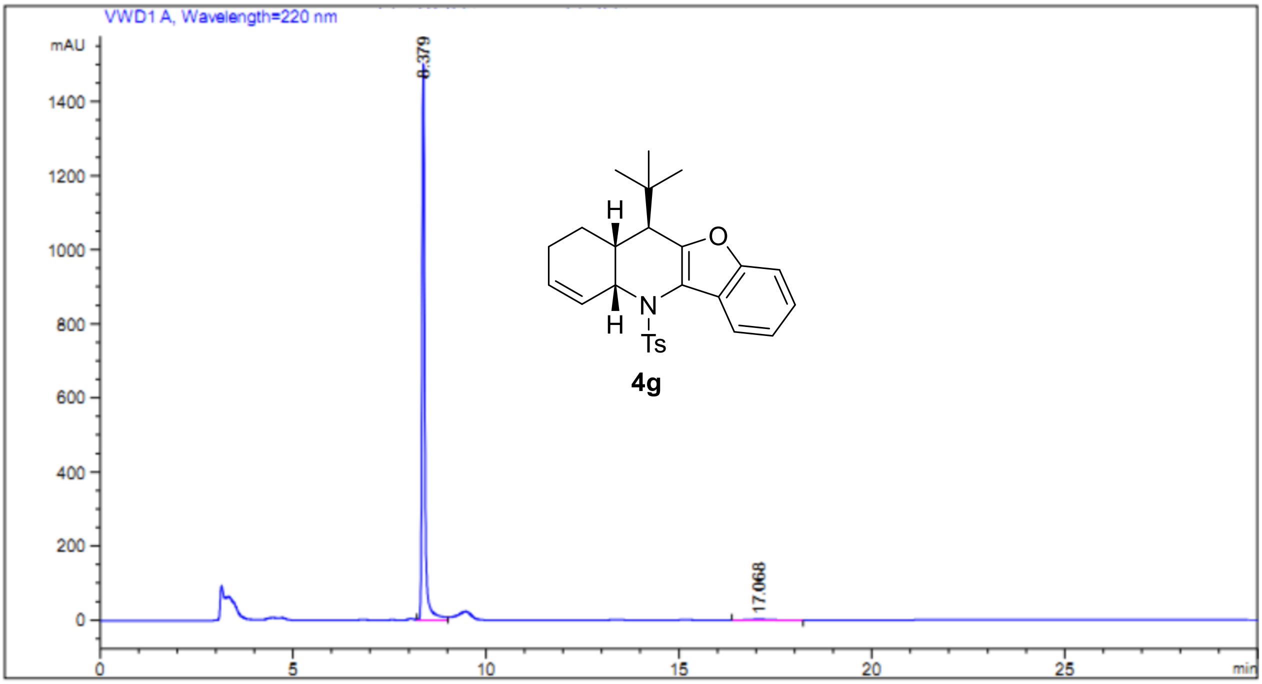

#	[min]		[min]	[mAU*s]	[mAU]	%
1	8.379	VV	0.0730	7358.41504	1505.52246	99.1065
2	17.068	BB	0.4730	66.34195	2.06153	0.8935

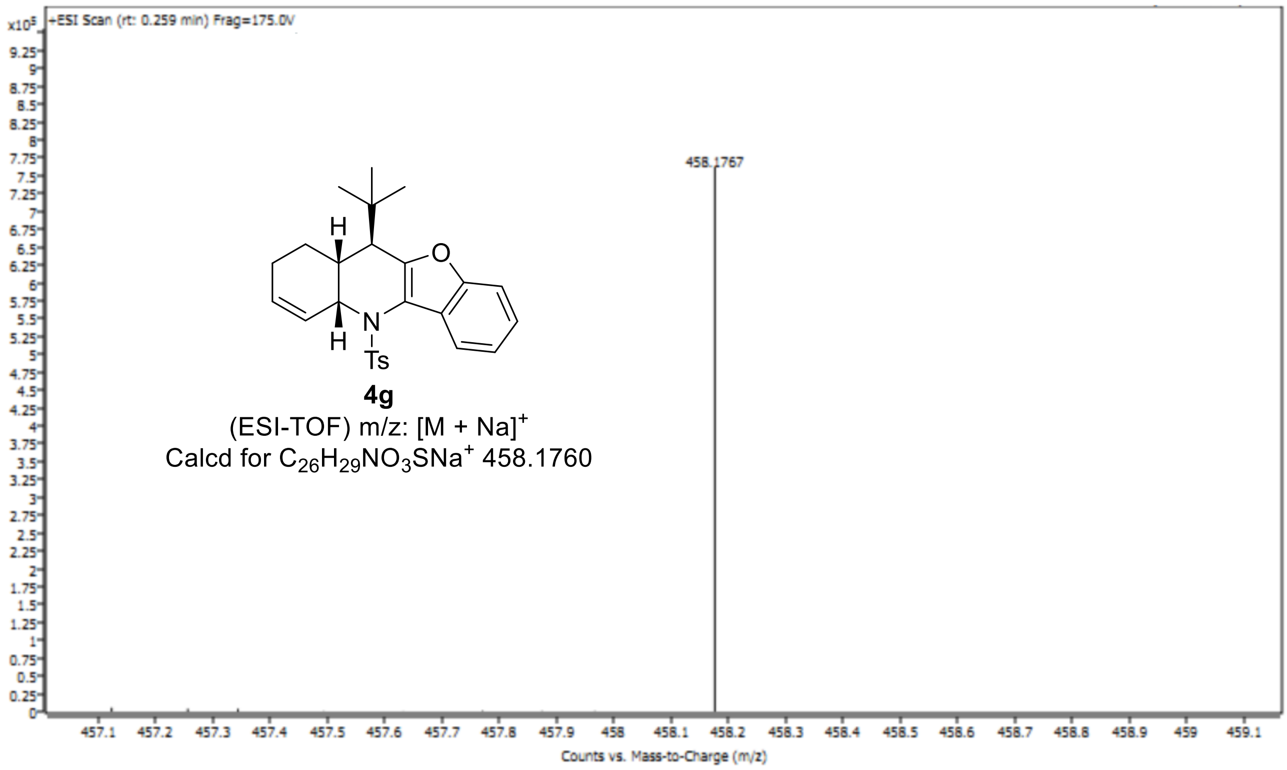
+ESI Scan (rt: 0.259 min) Frag=175.0V
x10^5
458.1767
4g
(ESI-TOF) m/z: [M + Na]+
Calcd for C26H29NO3SNa+ 458.1760
Counts vs. Mass-to-Charge (m/z)

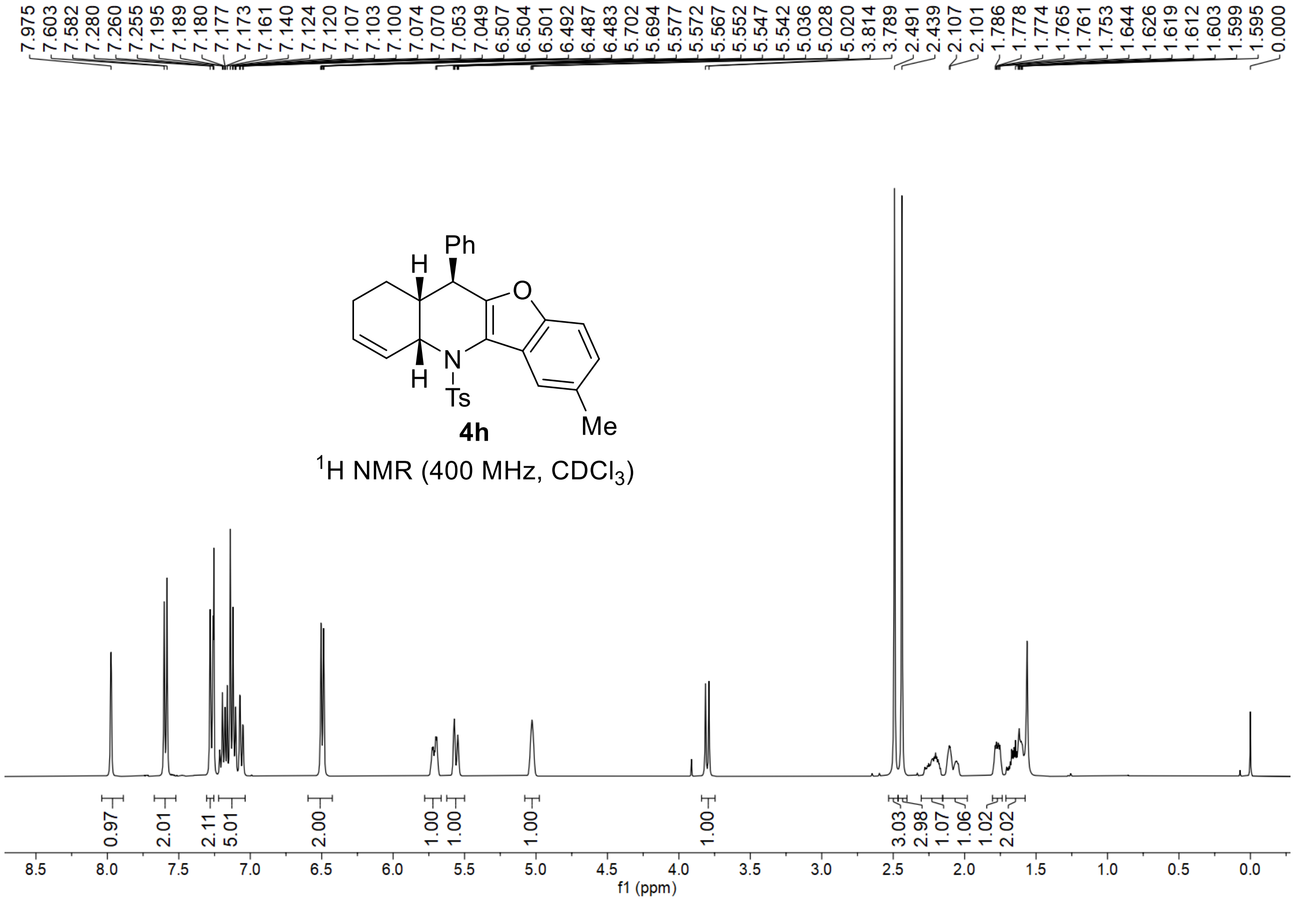
Ph
H
O
N
H
Ts
Me
4h
1H NMR (400 MHz, CDCl3)
f1 (ppm)

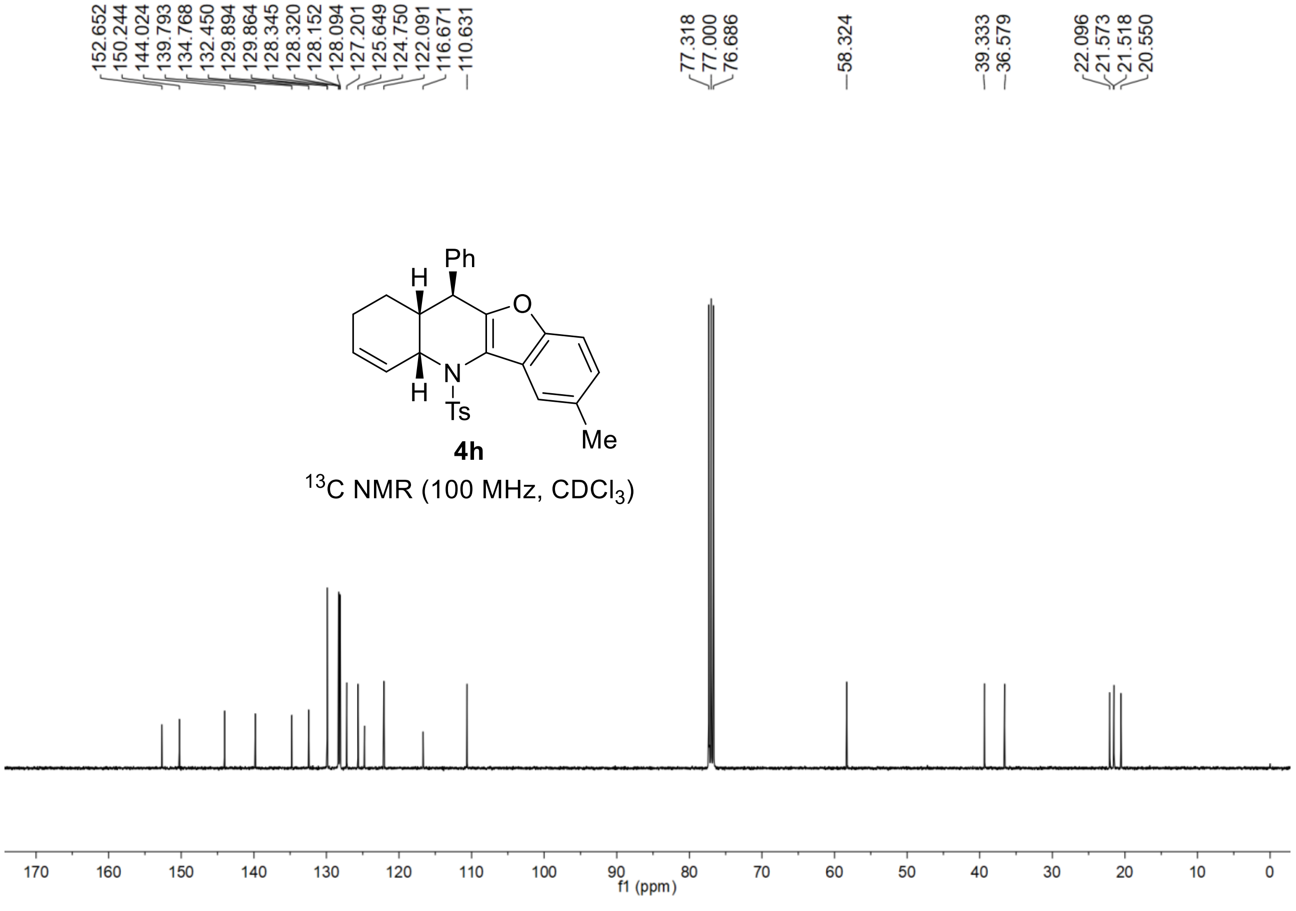
Ph
H
O
N
H
Ts
Me
4h
13C NMR (100 MHz, CDCl3)
f1 (ppm)

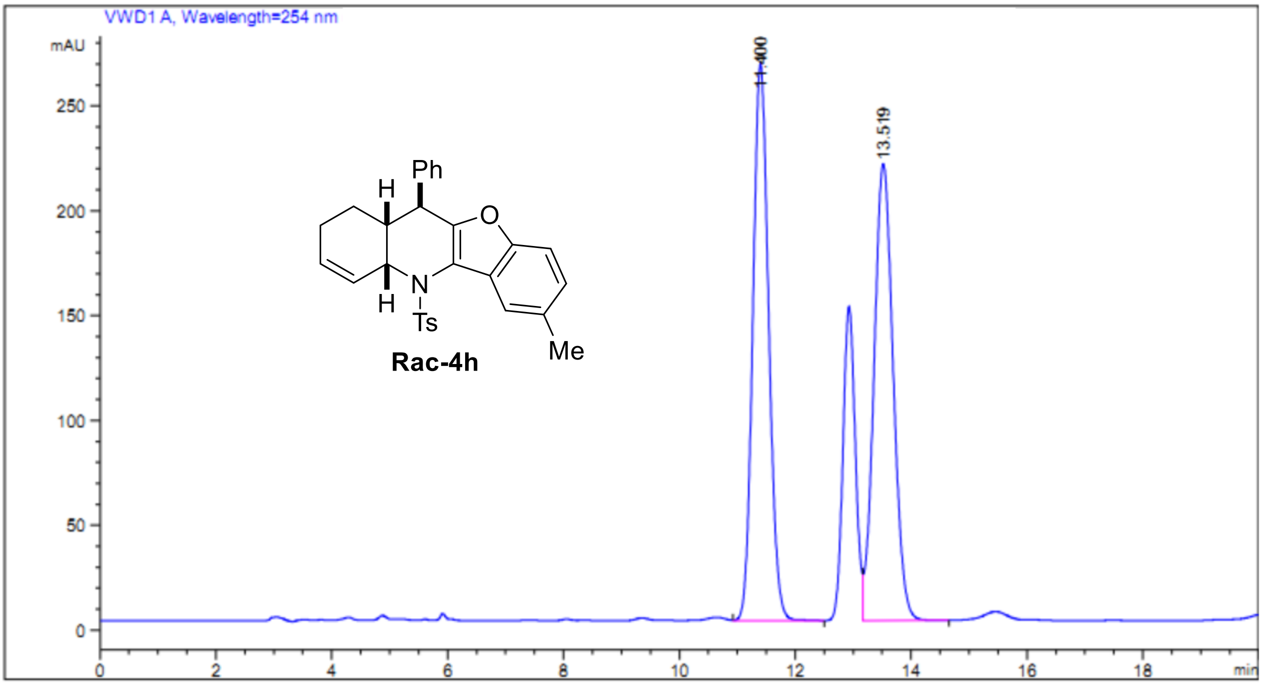

#	[min]		[min]	[mAU*s]	[mAU]	%
1	11.400	VB	0.2955	5037.05176	265.62692	50.1354
2	13.519	VB	0.3571	5009.85352	217.86008	49.8646

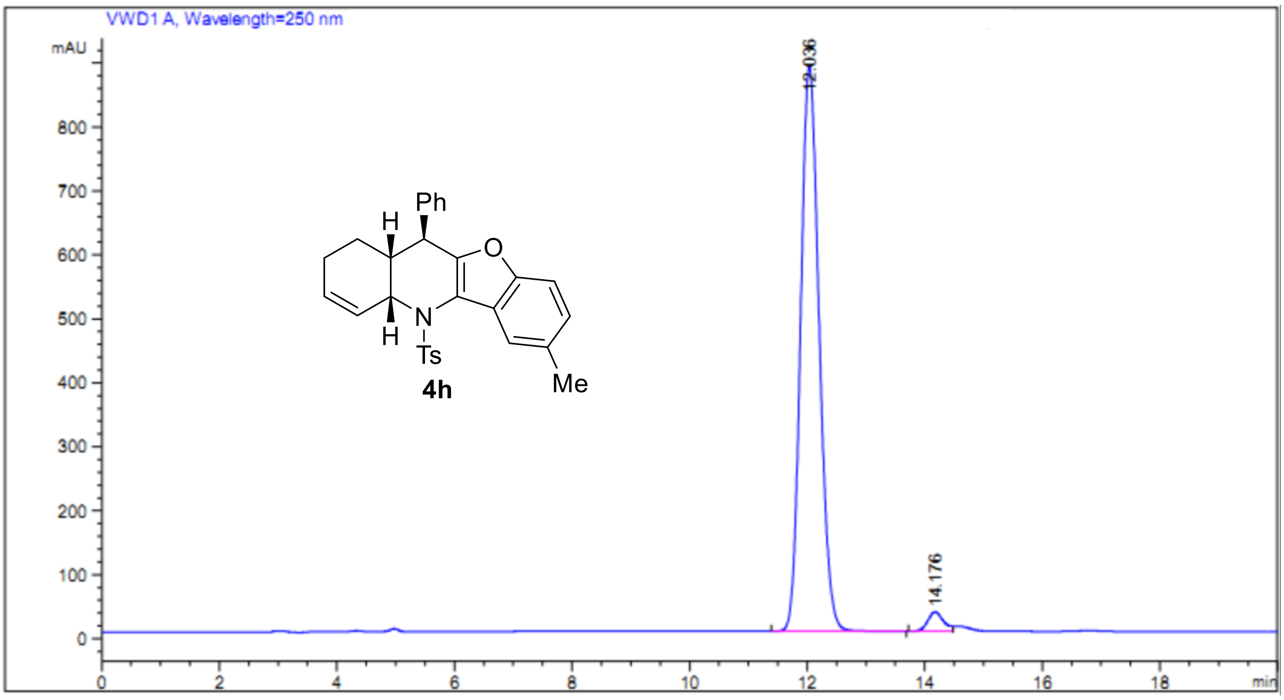

#	[min]		[min]	[mAU*s]	[mAU]	%
1	12.036	BB	0.3401	1.92412e4	883.25452	97.1641
2	14.176	BV	0.2834	561.59491	30.07371	2.8359

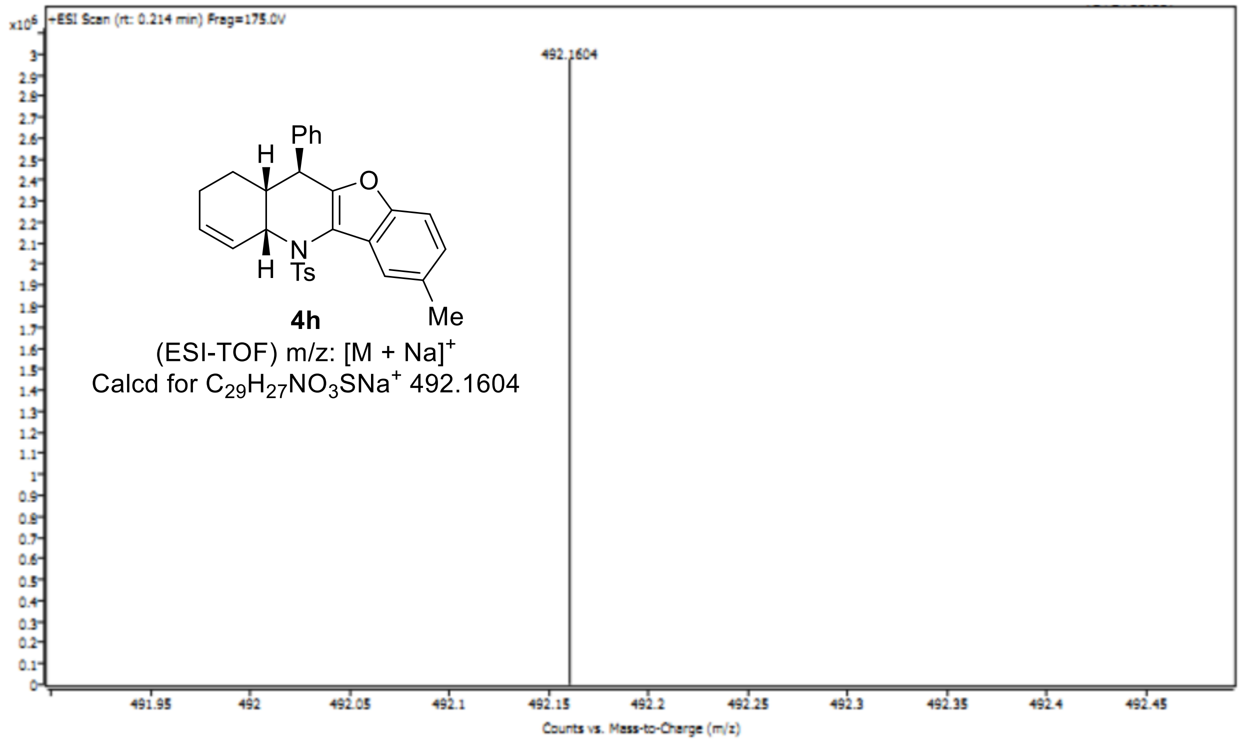
x10^6 +ESI Scan (rt: 0.214 min) Frag=175.0V
492.1604
Ph
H
O
N
H Ts
Me
4h
(ESI-TOF) m/z: [M + Na]+
Calcd for C29H27NO3SNa+ 492.1604
491.95
492
492.05
492.1
492.15
492.2
492.25
492.3
492.35
492.4
492.45
Counts vs. Mass-to-Charge (m/z)

4i

^{1}H NMR (400 MHz, $CDCl_3$)

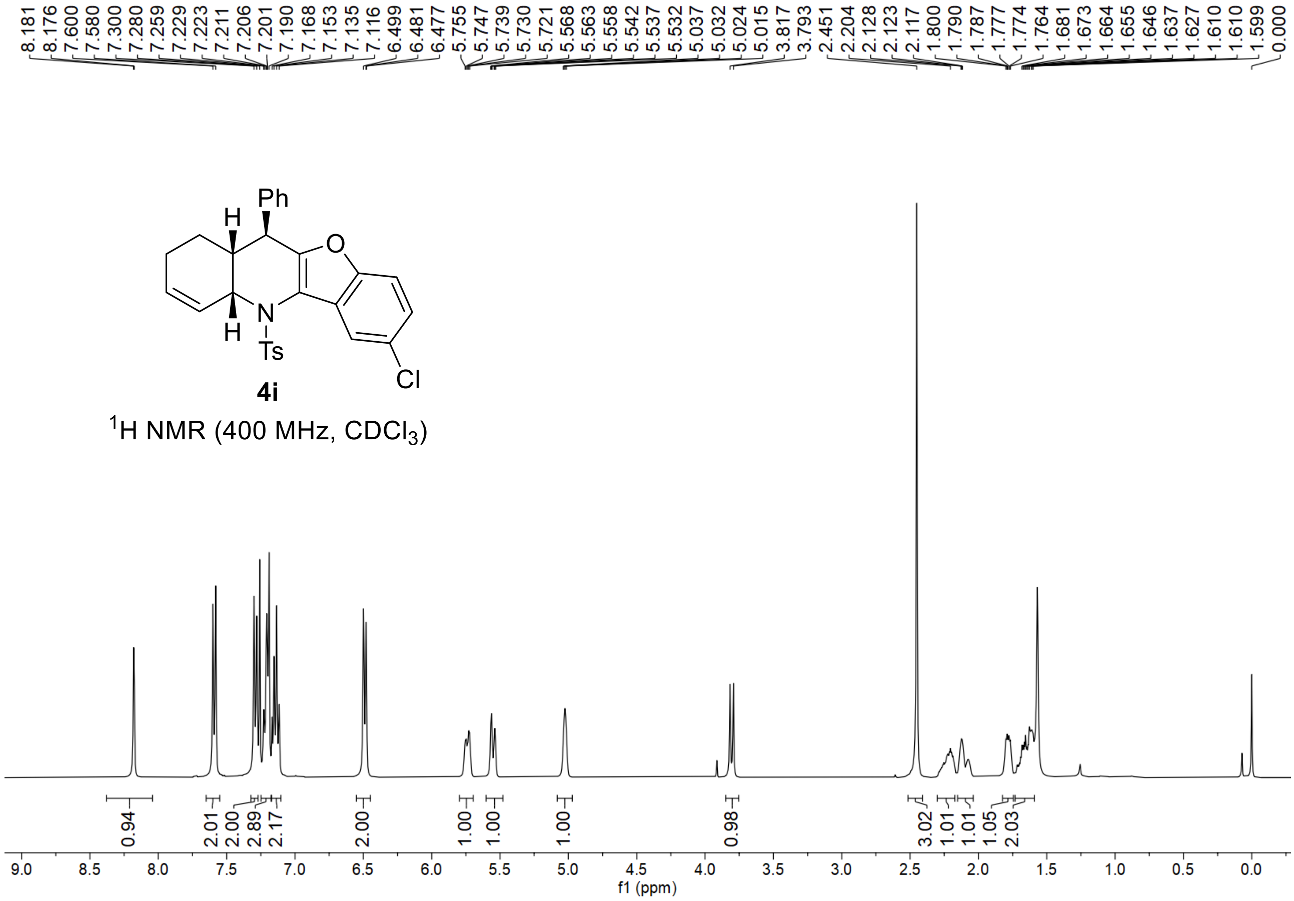

4i

^{13}C NMR (100 MHz, $CDCl_3$)

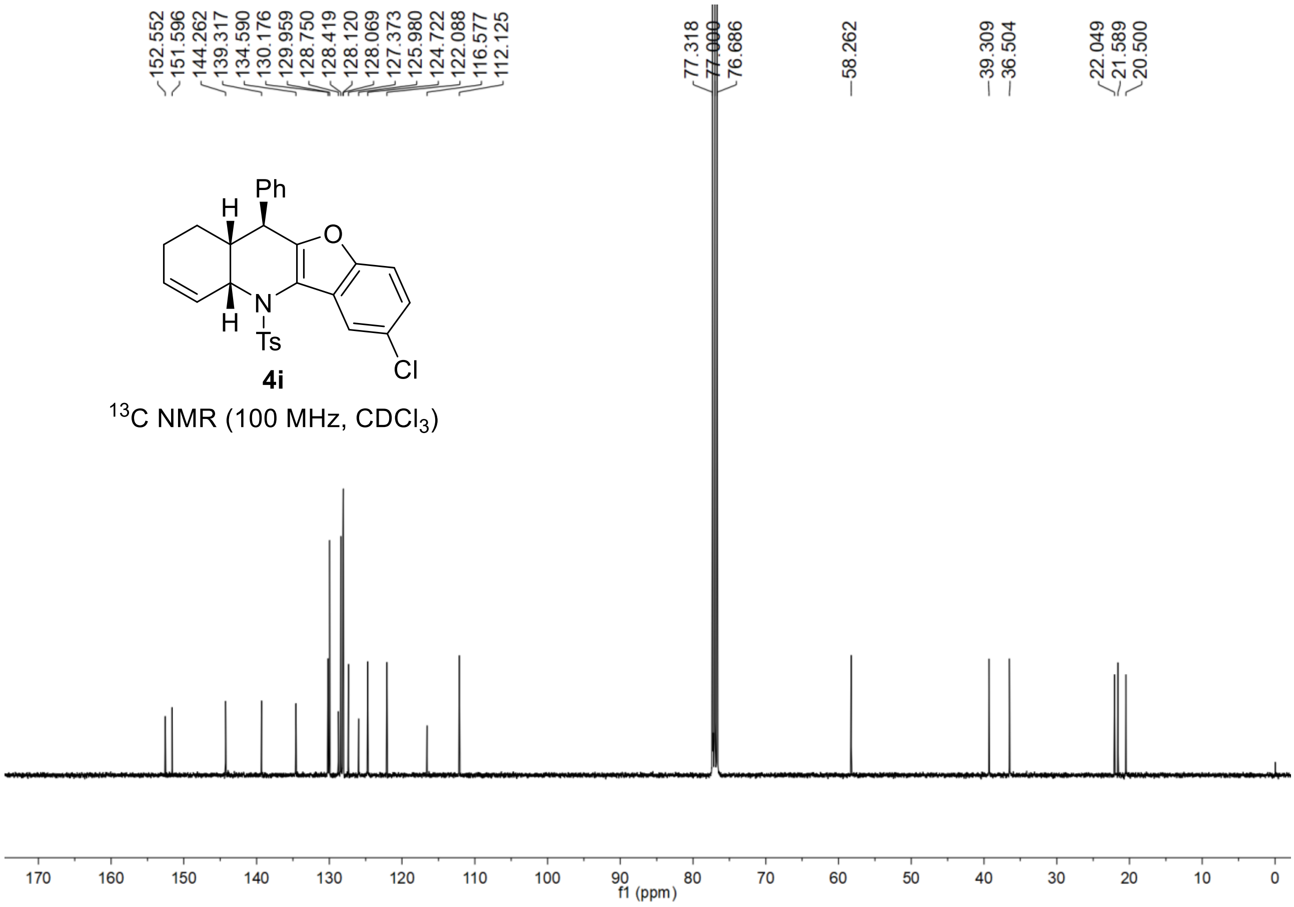

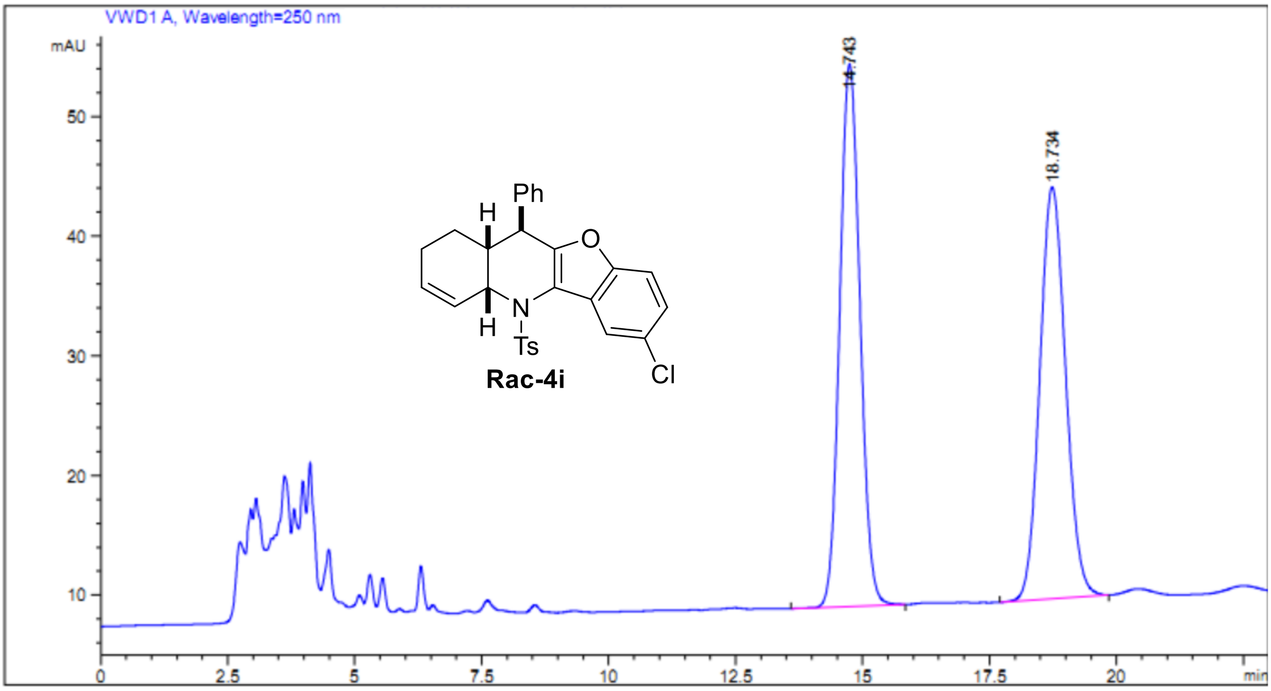

#	[min]		[min]	[mAU*s]	[mAU]	%
1	14.743	BB	0.4342	1257.26282	45.33516	50.8213
2	18.734	BB	0.5536	1216.62671	34.42382	49.1787

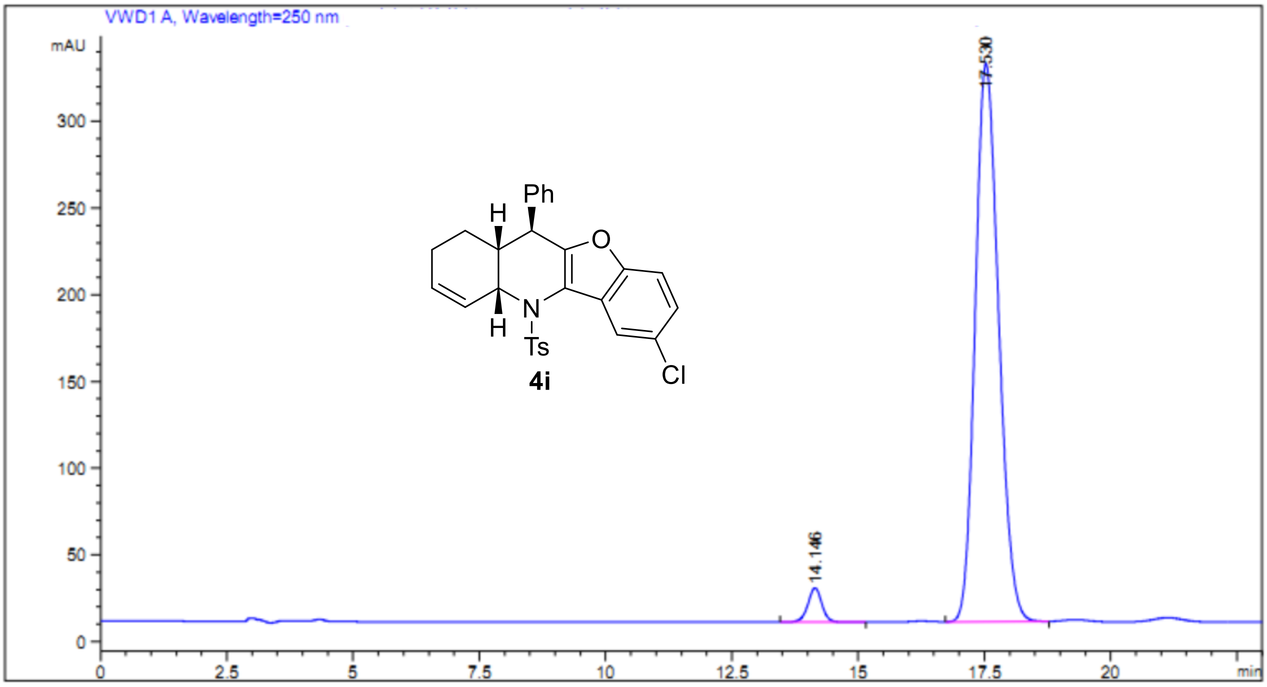

#	[min]		[min]	[mAU*s]	[mAU]	%
1	14.146	BB	0.2951	381.65308	19.77401	3.5419
2	17.530	BB	0.5035	1.03937e4	321.75018	96.4581

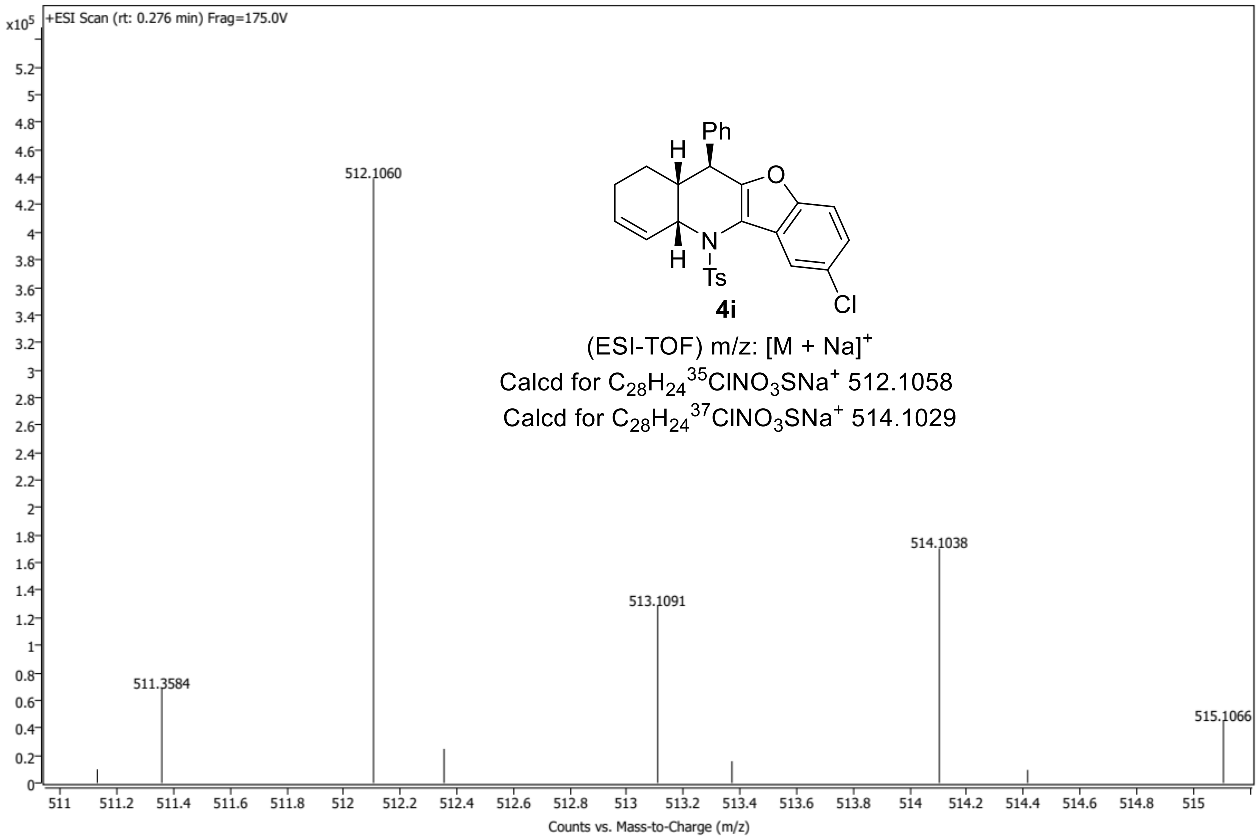

+ESI Scan (rt: 0.276 min) Frag=175.0V
x10^5
512.1060
511.3584
513.1091
514.1038
515.1066
Ph
H
O
N
H
Ts
Cl
4i
(ESI-TOF) m/z: [M + Na]+
Calcd for C28H24 35ClNO3SNa+ 512.1058
Calcd for C28H24 37ClNO3SNa+ 514.1029
Counts vs. Mass-to-Charge (m/z)

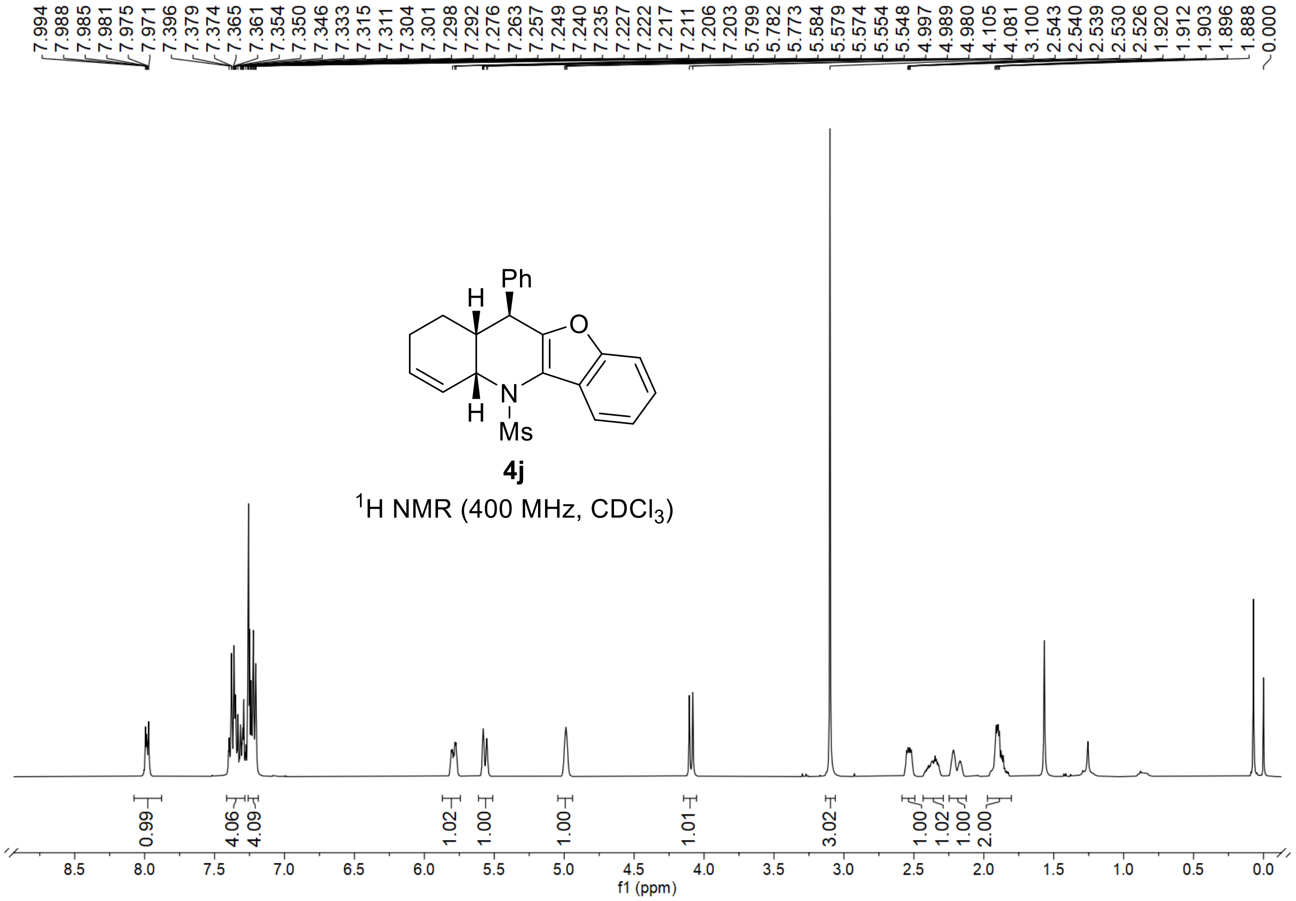
7.994
7.988
7.985
7.981
7.975
7.971
7.396
7.379
7.374
7.365
7.361
7.354
7.350
7.346
7.333
7.315
7.311
7.304
7.301
7.298
7.292
7.276
7.263
7.257
7.249
7.240
7.235
7.227
7.222
7.217
7.211
7.206
7.203
5.799
5.782
5.773
5.584
5.579
5.574
5.554
5.548
4.997
4.989
4.980
4.105
4.081
3.100
2.543
2.540
2.539
2.530
2.526
1.920
1.912
1.903
1.896
1.888
0.000
Ph
H
O
N
H
Ms
4j
¹H NMR (400 MHz, CDCl₃)
0.99
4.06
4.09
1.02
1.00
1.00
1.01
3.02
1.00
1.02
1.00
2.00
8.5
8.0
7.5
7.0
6.5
6.0
5.5
5.0
4.5
4.0
3.5
3.0
2.5
2.0
1.5
1.0
0.5
0.0
f1 (ppm)

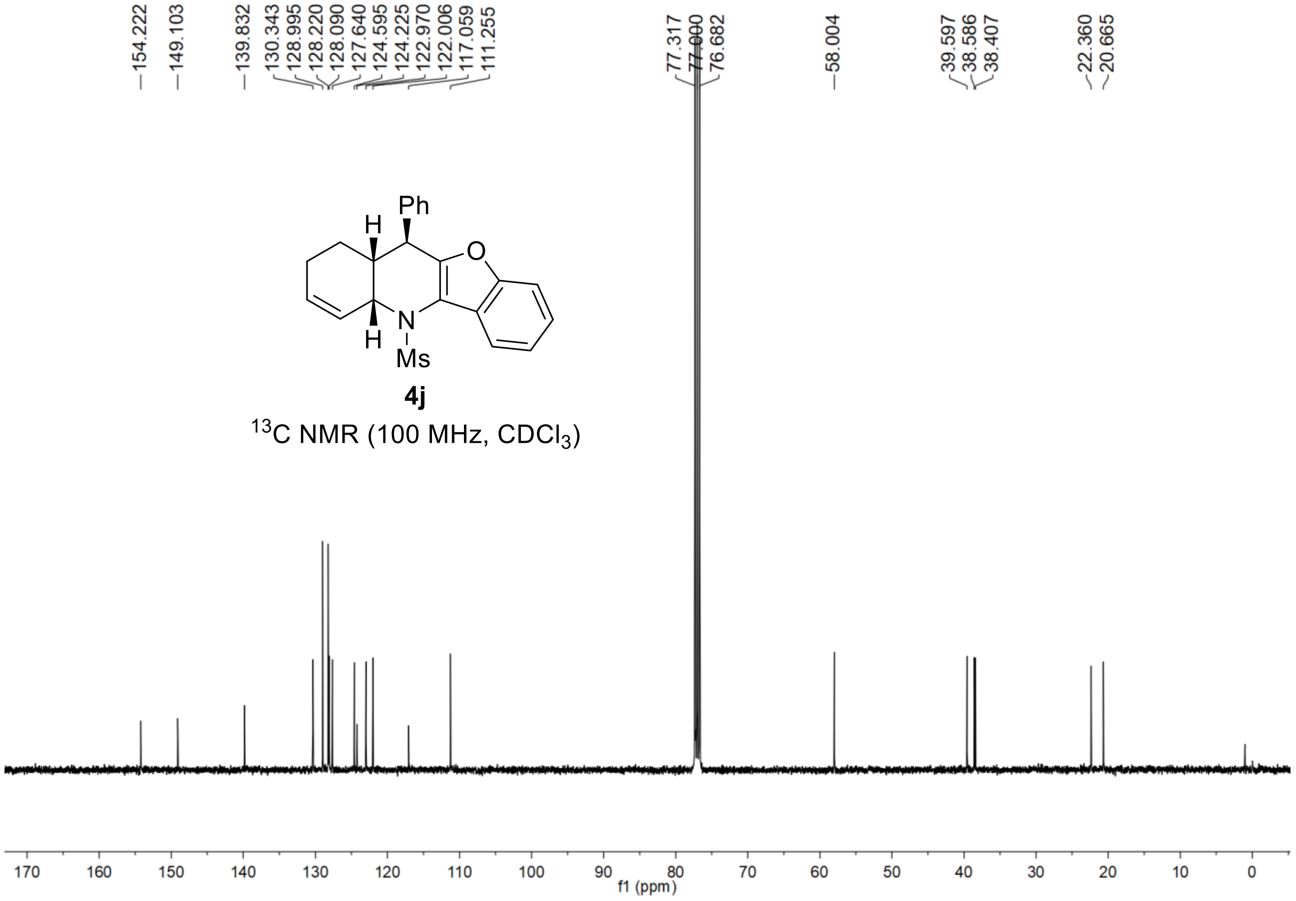
154.222
149.103
139.832
130.343
128.995
128.220
128.090
127.640
124.595
124.225
122.970
122.006
117.059
111.255
77.317
77.000
76.682
58.004
39.597
38.586
38.407
22.360
20.665
Ph
H
O
N
H
Ms
4j
¹³C NMR (100 MHz, CDCl₃)
170
160
150
140
130
120
110
100
90
80
70
60
50
40
30
20
10
0
f1 (ppm)

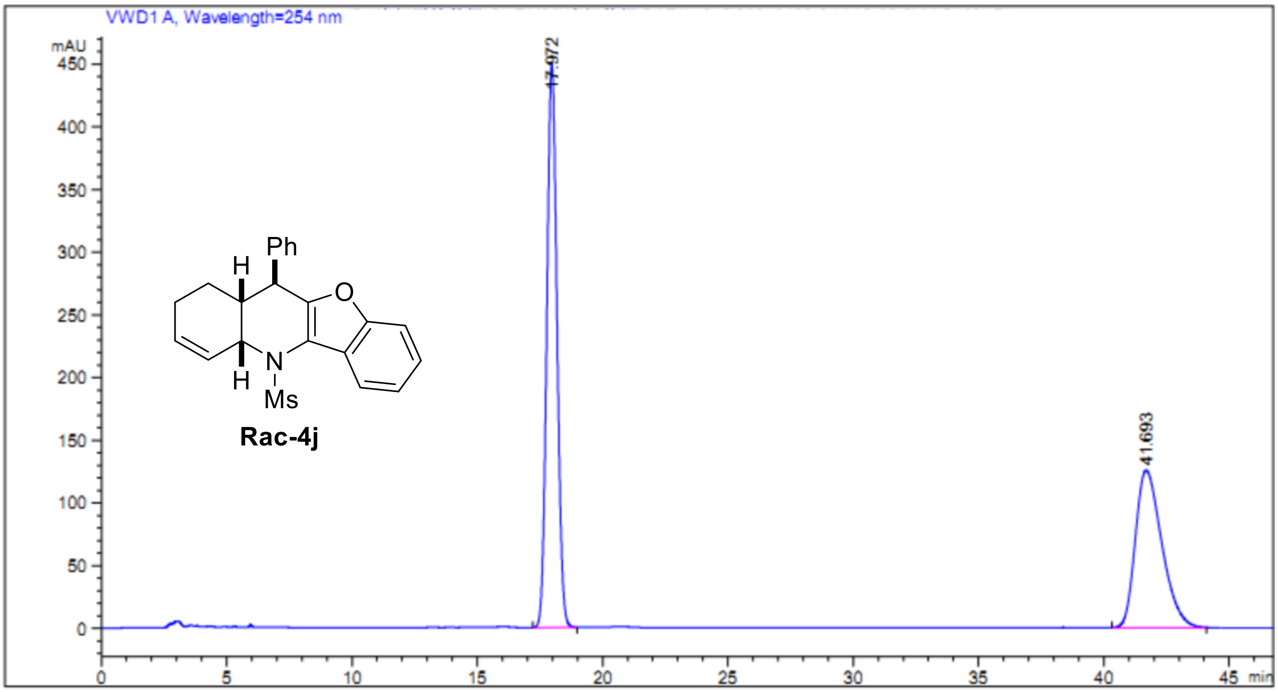

#	[min]		[min]	[mAU*s]	[mAU]	%
1	17.972	BB	0.4399	1.26156e4	448.96817	57.7784
2	41.693	BB	1.1233	9218.83301	125.52738	42.2216

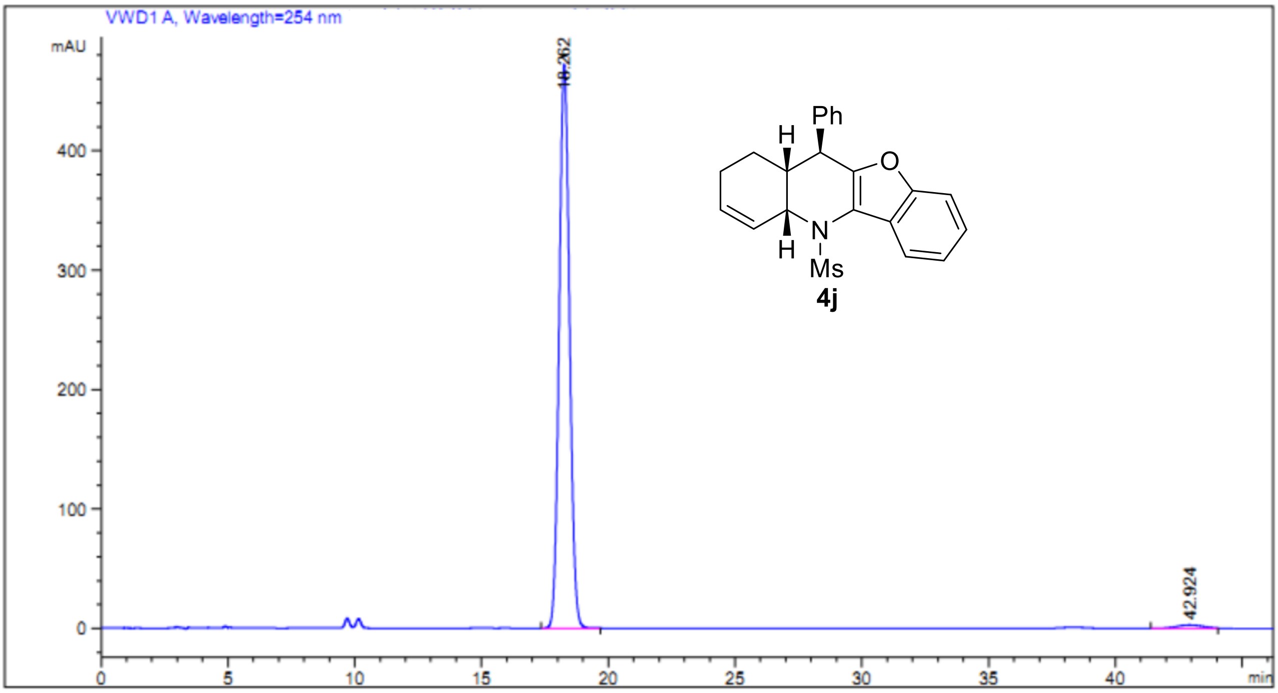

#	[min]		[min]	[mAU*s]	[mAU]	%
1	18.262	BBA	0.4451	1.34678e4	471.85791	98.4604
2	42.924	BBA	0.8633	210.59492	2.95057	1.5396

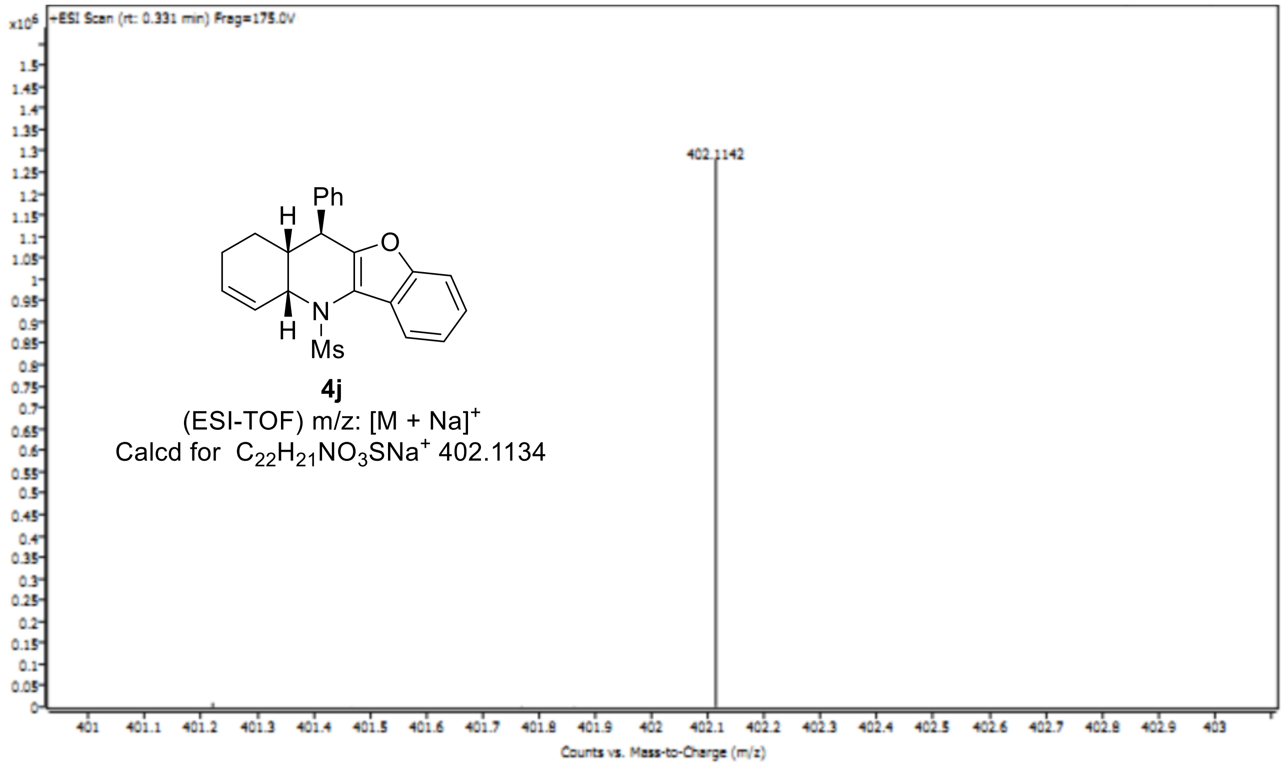
+ESI Scan (rt: 0.331 min) Frag=175.0V
x10^6
402.1142
Ph
H
O
N
H
Ms
4j
(ESI-TOF) m/z: [M + Na]+
Calcd for C22H21NO3SNa+ 402.1134
Counts vs. Mass-to-Charge (m/z)

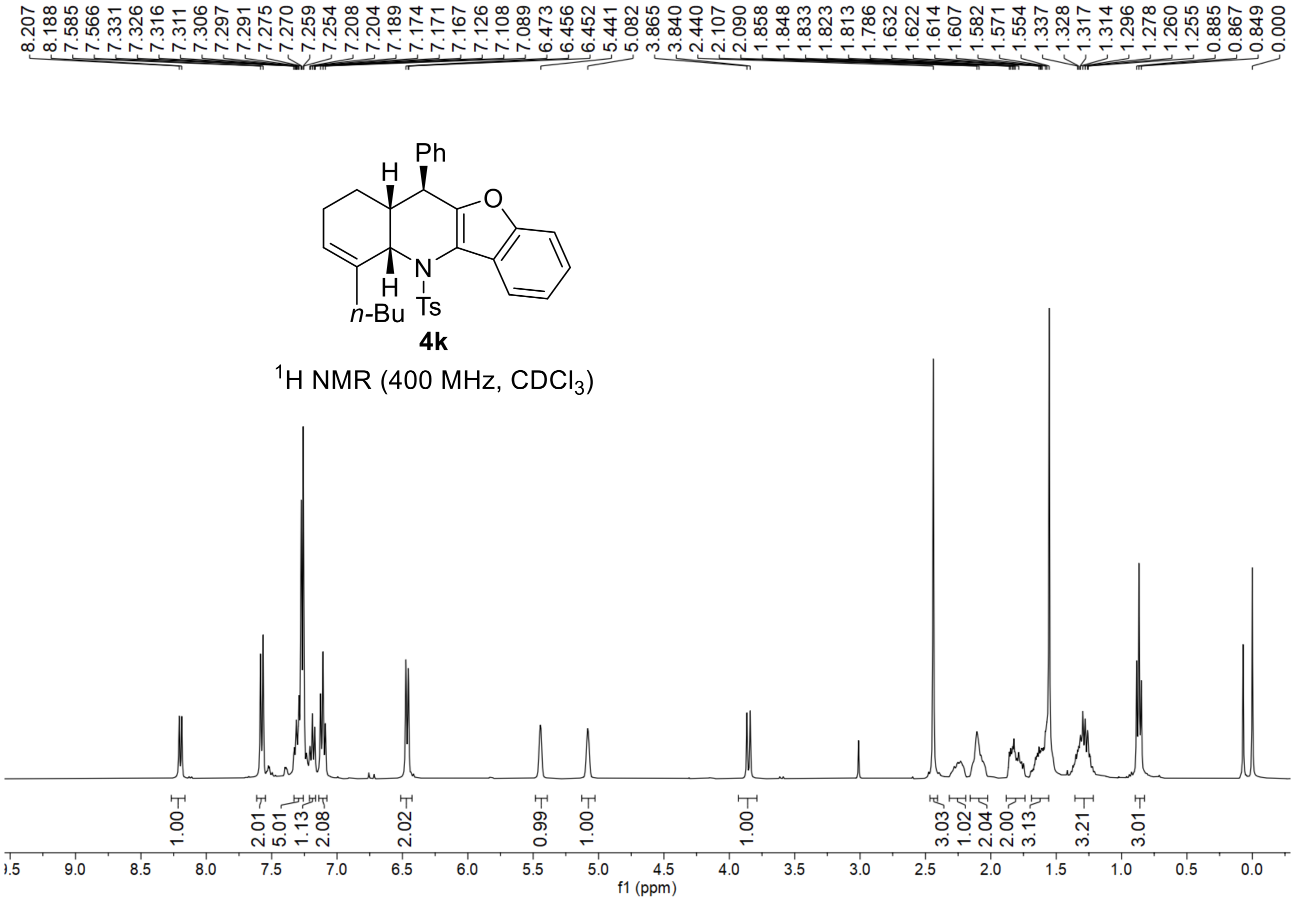
8.207 8.188 7.585 7.566 7.331 7.326 7.316 7.311 7.306 7.297 7.291 7.275 7.270 7.259 7.254 7.208 7.204 7.189 7.174 7.171 7.167 7.126 7.108 7.089 6.473 6.456 6.452 5.441 5.082 3.865 3.840 2.440 2.107 2.090 1.858 1.848 1.833 1.823 1.813 1.786 1.632 1.622 1.614 1.607 1.582 1.571 1.554 1.337 1.328 1.317 1.314 1.296 1.278 1.260 1.255 0.885 0.867 0.849 0.000
Ph
H
O
N
H
Ts
n-Bu
4k
1H NMR (400 MHz, CDCl3)
1.00 2.01 5.01 1.13 2.08 2.02 0.99 1.00 1.00 3.03 1.02 2.04 2.00 3.13 3.21 3.01
9.0 8.5 8.0 7.5 7.0 6.5 6.0 5.5 5.0 4.5 4.0 3.5 3.0 2.5 2.0 1.5 1.0 0.5 0.0
f1 (ppm)

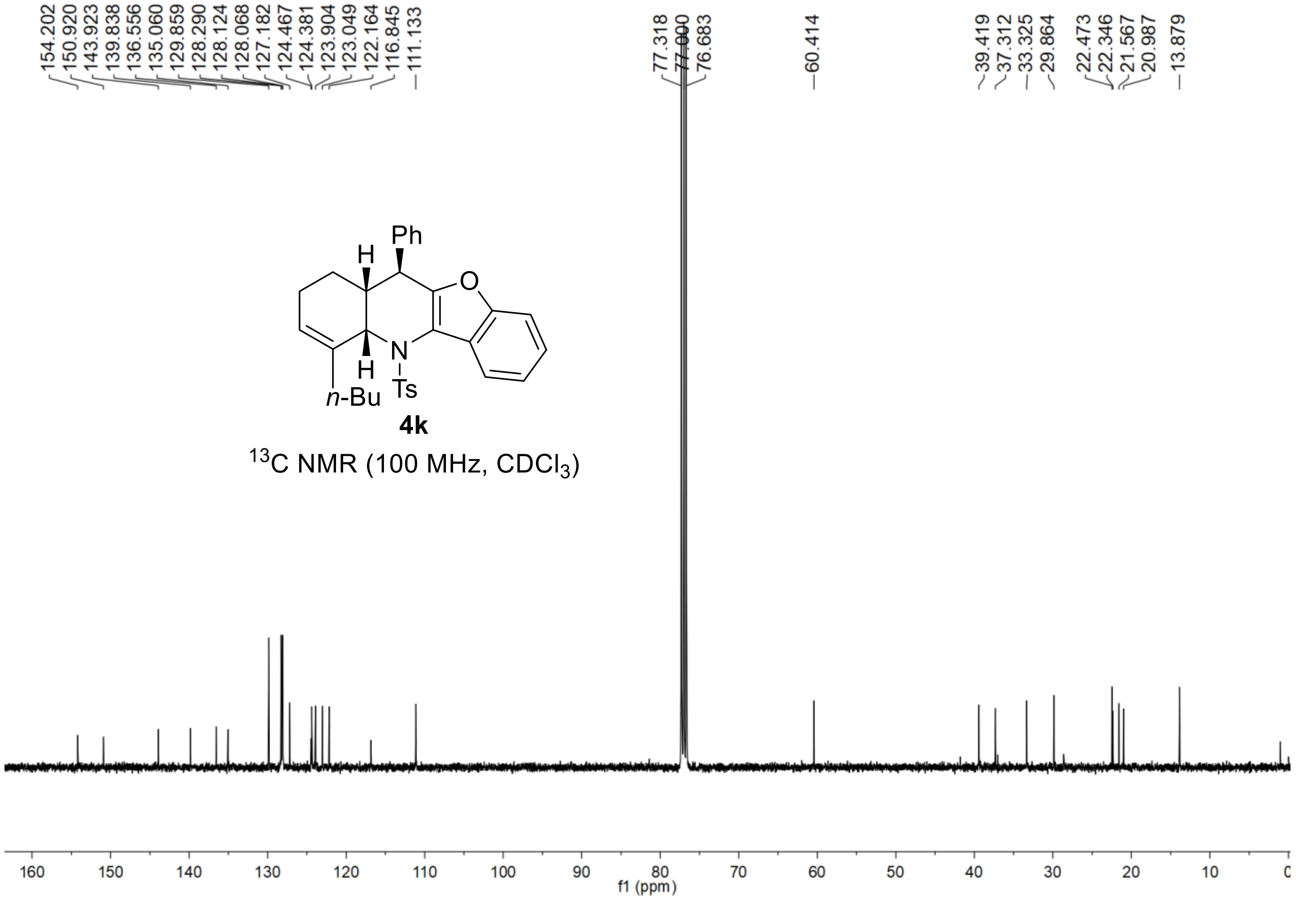
154.202 150.920 143.923 139.838 136.556 135.060 129.859 128.290 128.124 128.068 127.182 124.467 124.381 123.904 123.049 122.164 116.845 111.133
77.318 77.000 76.683
60.414
39.419 37.312 33.325 29.864
22.473 22.346 21.567 20.987
13.879
Ph
H
O
N
H
Ts
n-Bu
4k
13C NMR (100 MHz, CDCl3)
160 150 140 130 120 110 100 90 80 70 60 50 40 30 20 10 0
f1 (ppm)

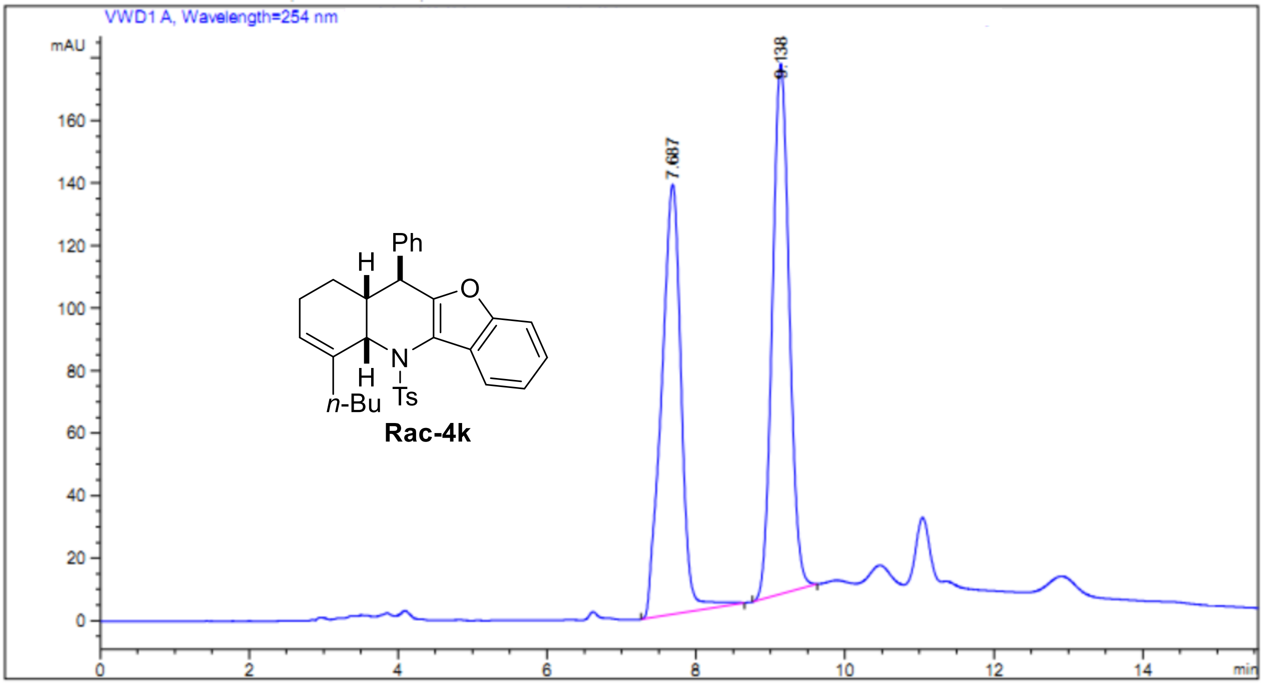

#	[min]		[min]	[mAU*s]	[mAU]	%
1	7.687	BB	0.2749	2518.17505	137.45985	48.4014
2	9.138	BB	0.2458	2684.51465	169.65187	51.5986

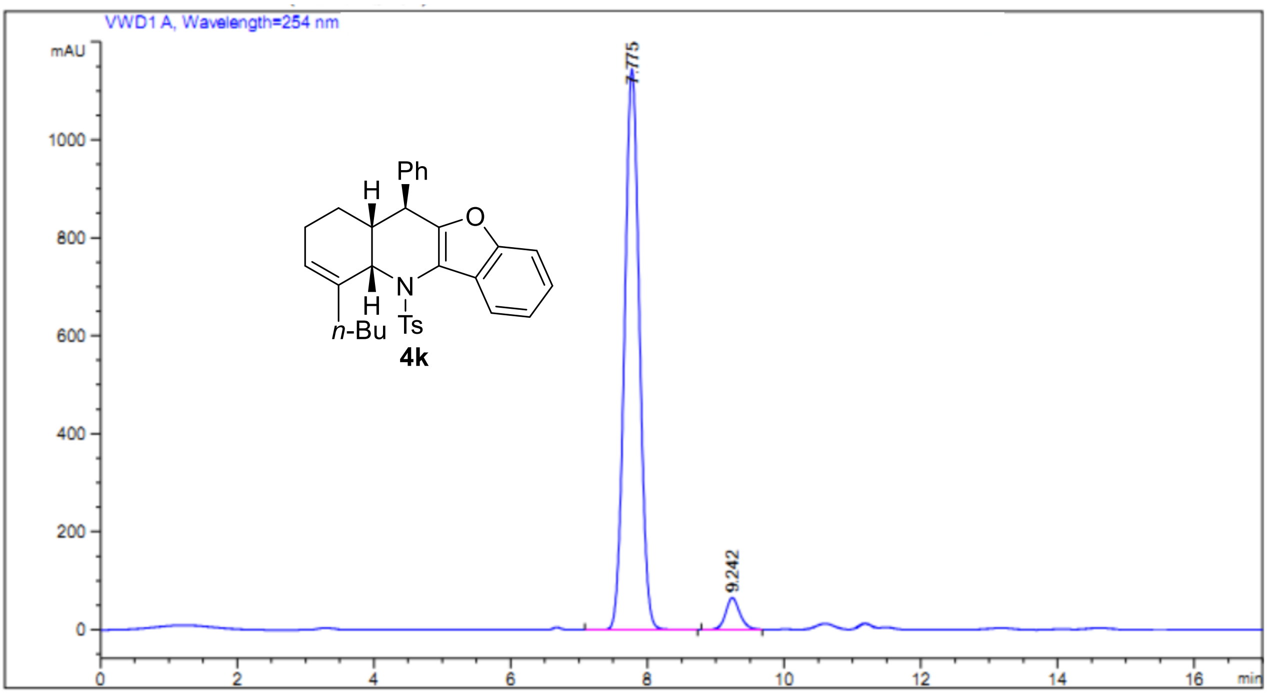

#	[min]		[min]	[mAU*s]	[mAU]	%
1	7.775	BB	0.2340	1.72324e4	1143.59082	94.8896
2	9.242	BBA	0.2157	928.07147	65.15652	5.1104

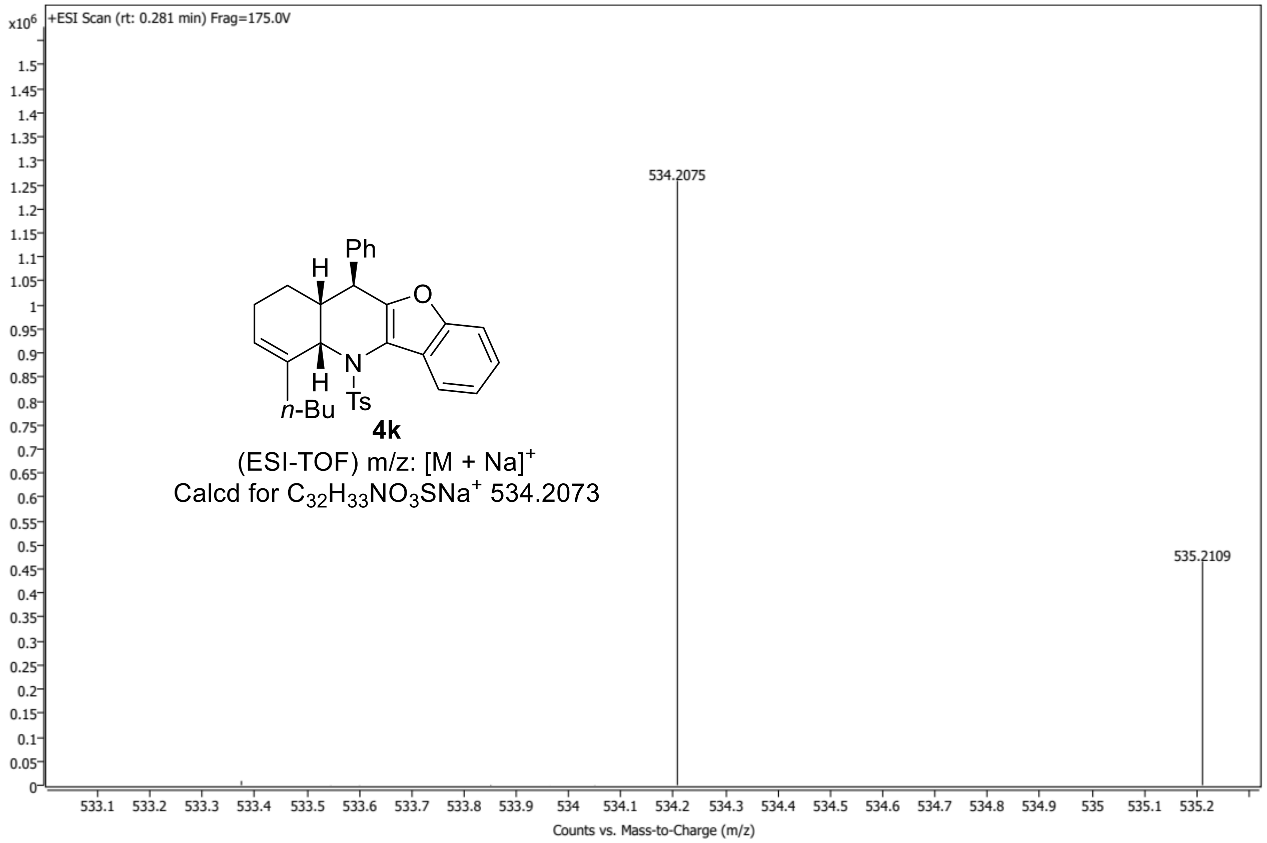
+ESI Scan (rt: 0.281 min) Frag=175.0V
x10^6
534.2075
535.2109
Ph
H
O
N
H
Ts
n-Bu
4k
(ESI-TOF) m/z: [M + Na]+
Calcd for C32H33NO3SNa+ 534.2073
Counts vs. Mass-to-Charge (m/z)

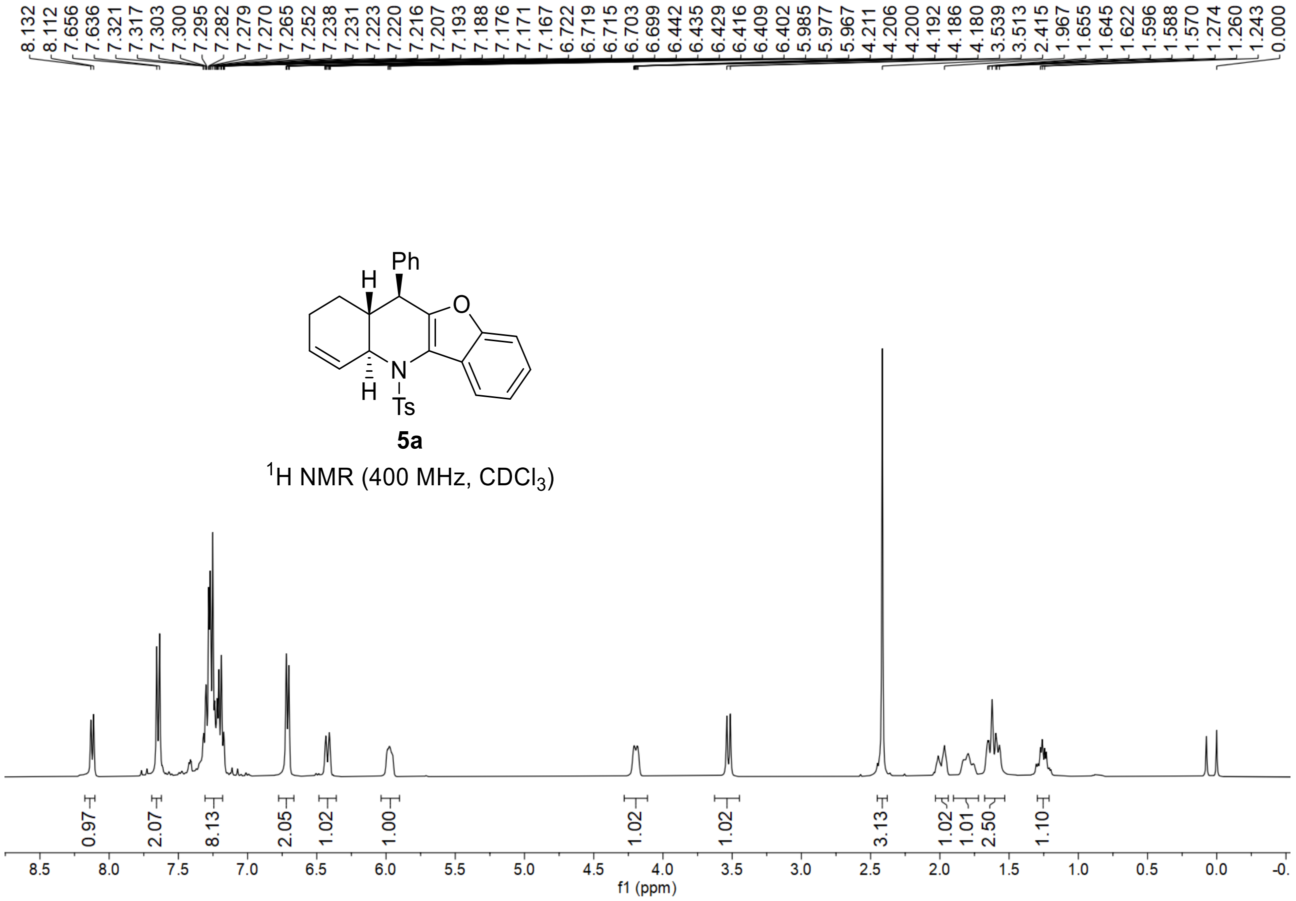
8.132 8.112 7.656 7.636 7.321 7.317 7.303 7.300 7.295 7.282 7.279 7.270 7.265 7.252 7.238 7.231 7.223 7.220 7.216 7.207 7.193 7.188 7.176 7.171 7.167 6.722 6.719 6.715 6.703 6.699 6.442 6.435 6.429 6.416 6.409 6.402 5.985 5.977 5.967 4.211 4.206 4.200 4.192 4.186 4.180 3.539 3.513 2.415 1.967 1.655 1.645 1.622 1.596 1.588 1.570 1.274 1.260 1.243 0.000
Ph
H
O
N
H
Ts
5a
1H NMR (400 MHz, CDCl3)
0.97 2.07 8.13 2.05 1.02 1.00 1.02 1.02 3.13 1.02 1.01 2.50 1.10
f1 (ppm)

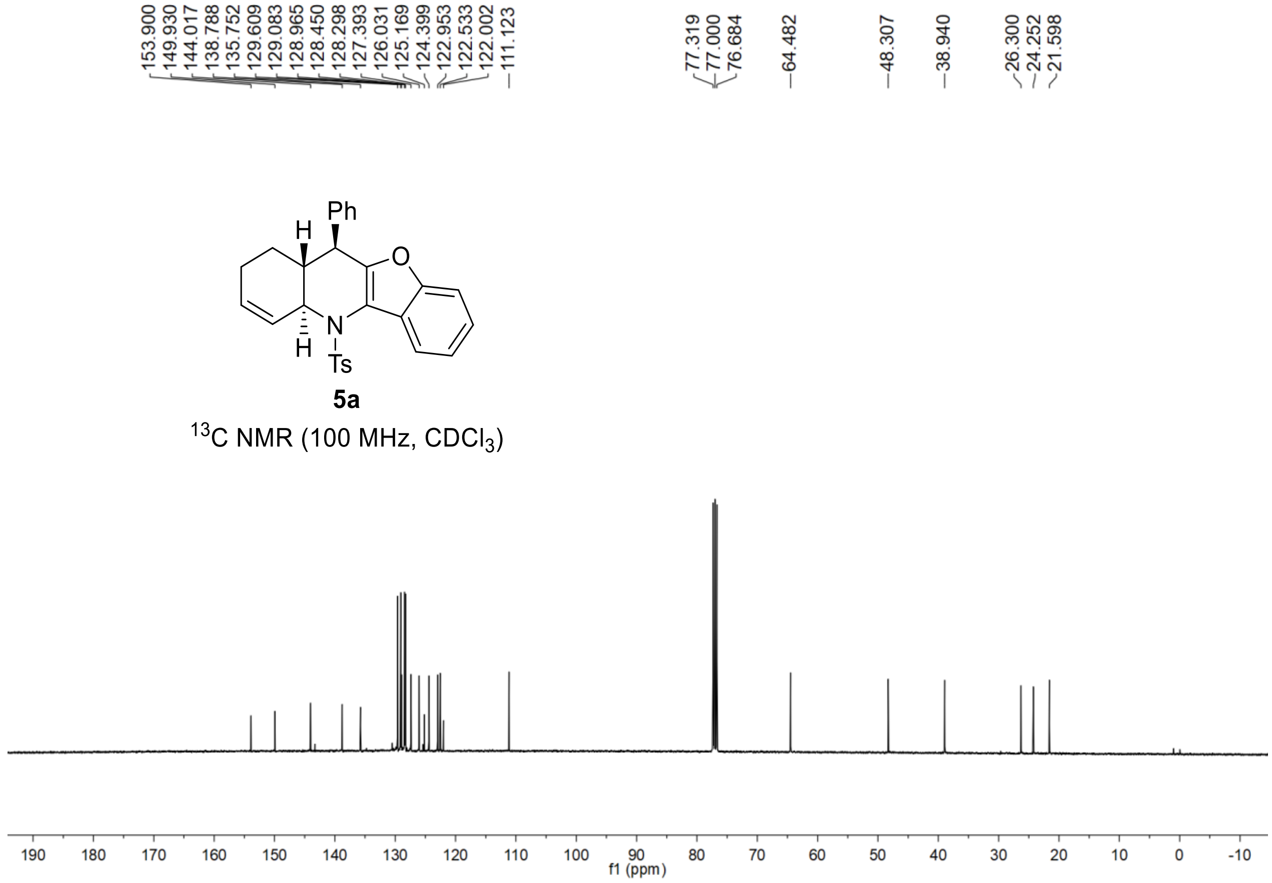
153.900 149.930 144.017 138.788 135.752 129.609 129.083 128.965 128.450 128.298 127.393 126.031 125.169 124.399 122.953 122.533 122.002 111.123
77.319 77.000 76.684
64.482
48.307
38.940
26.300 24.252 21.598
Ph
H
O
N
H
Ts
5a
13C NMR (100 MHz, CDCl3)
f1 (ppm)

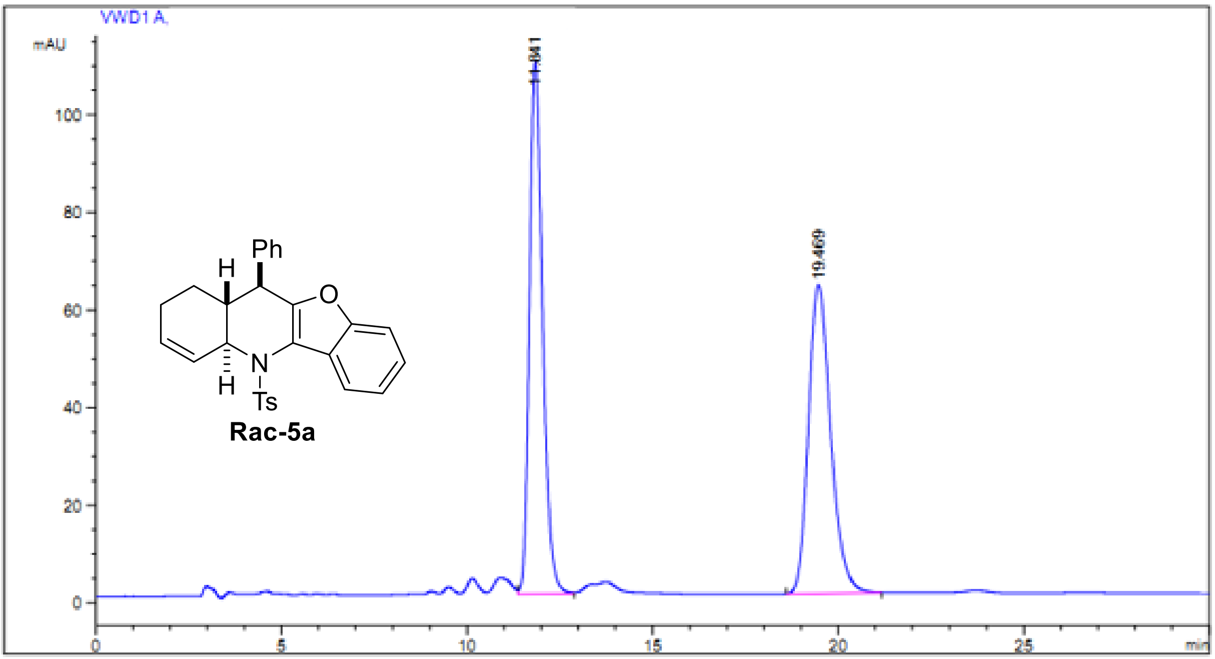

#	[min]		[min]	mAU *s	[mAU]	%
1	11.841	VV	0.3804	2683.02319	108.97501	50.4386
2	19.469	BB	0.6404	2636.35767	63.27567	49.5614

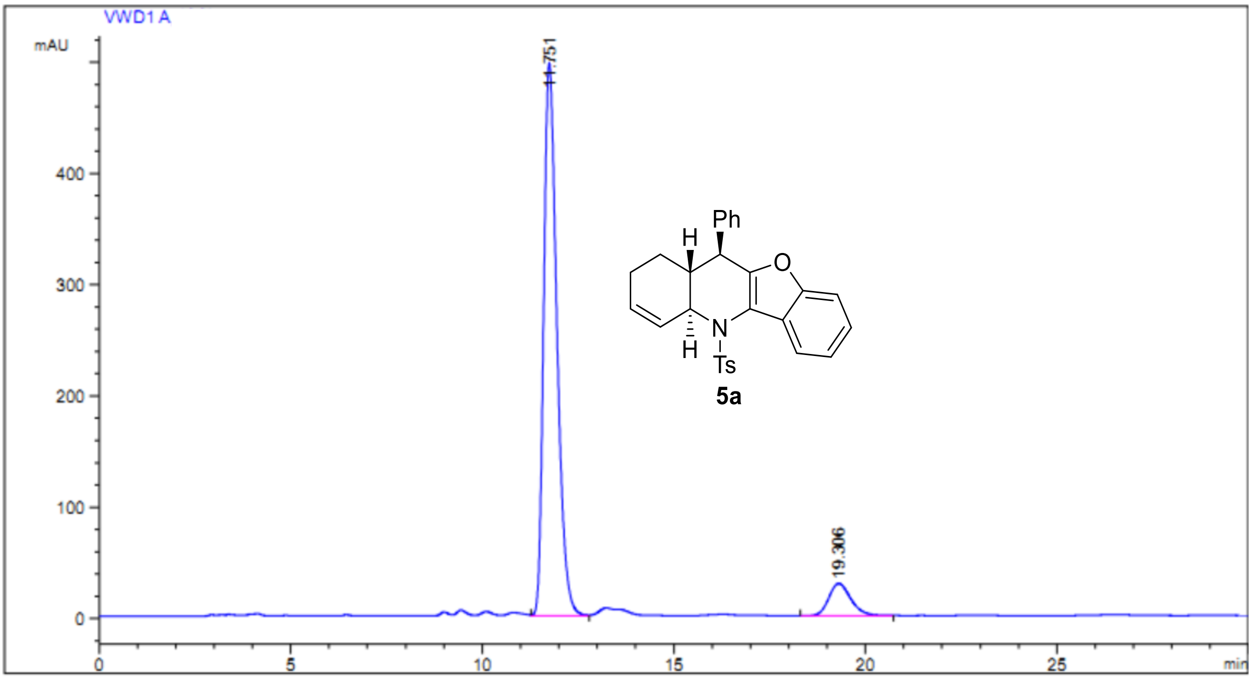

#	[min]		[min]	mAU *s	[mAU]	%
1	11.751	VV	0.3670	1.19035e4	496.57968	91.1792
2	19.306	VB	0.6106	1151.56470	28.89340	8.8208

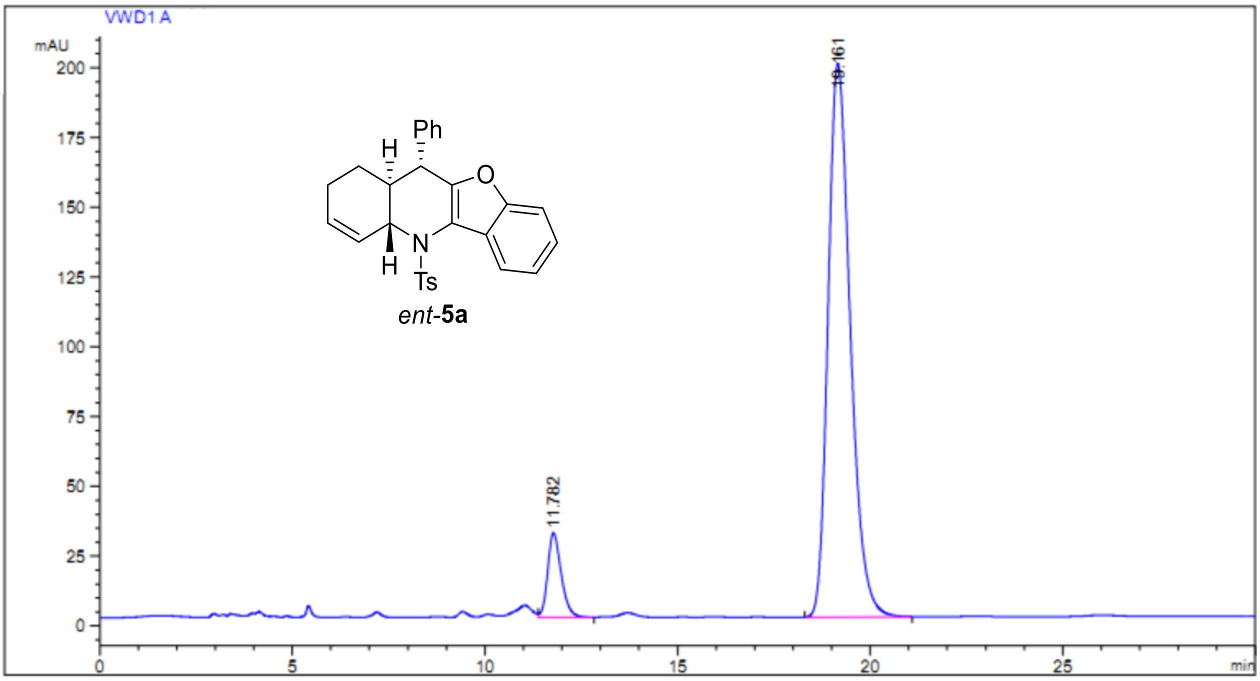

#	[min]		[min]	mAU *s	[mAU]	%
1	11.782	VB	0.3732	740.02606	30.20142	8.5200
2	19.161	BB	0.6161	7945.75879	198.24857	91.4800

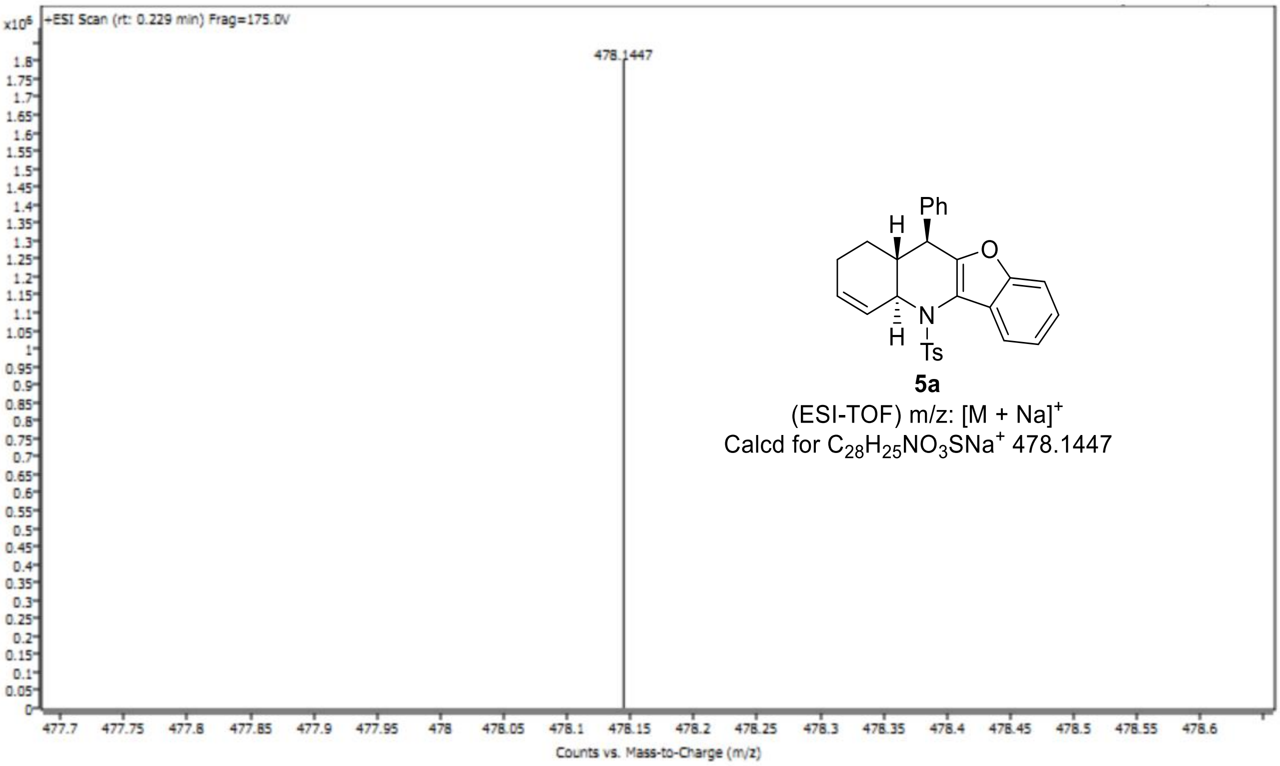

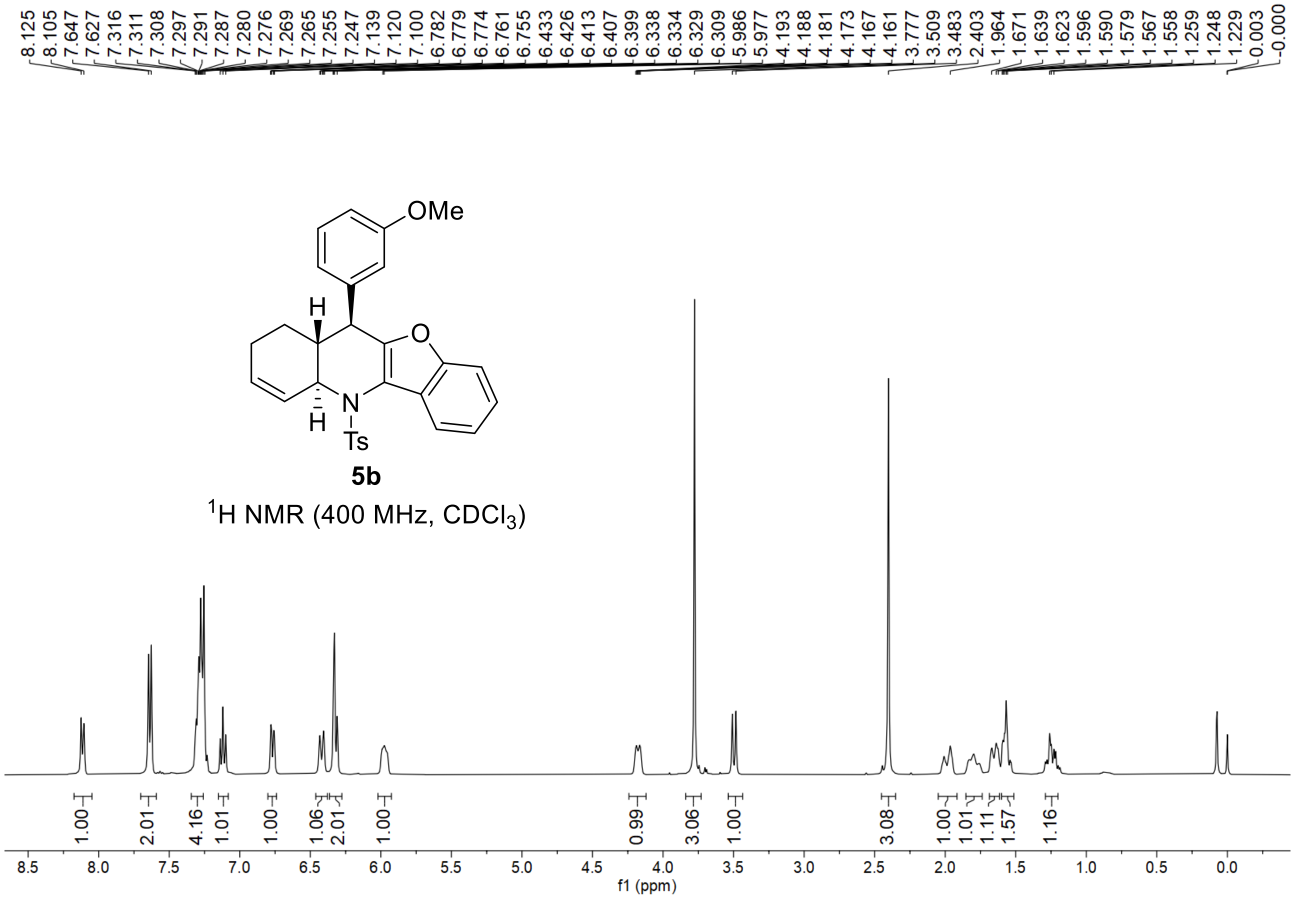
8.125 8.105 7.647 7.627 7.316 7.311 7.308 7.297 7.291 7.287 7.280 7.276 7.269 7.265 7.255 7.247 7.139 7.120 7.100 6.782 6.779 6.774 6.761 6.755 6.433 6.426 6.413 6.407 6.399 6.338 6.334 6.329 6.309 5.986 5.977 4.193 4.188 4.181 4.173 4.167 4.161 3.777 3.509 3.483 2.403 1.964 1.671 1.639 1.623 1.596 1.590 1.579 1.567 1.558 1.259 1.248 1.229 0.003 -0.000
OMe
H
O
N
H
Ts
5b
^{1}H NMR (400 MHz, CDCl$_3$)
1.00 2.01 4.16 1.01 1.00 1.06 2.01 1.00 0.99 3.06 1.00 3.08 1.00 1.01 1.11 1.57 1.16
f1 (ppm)

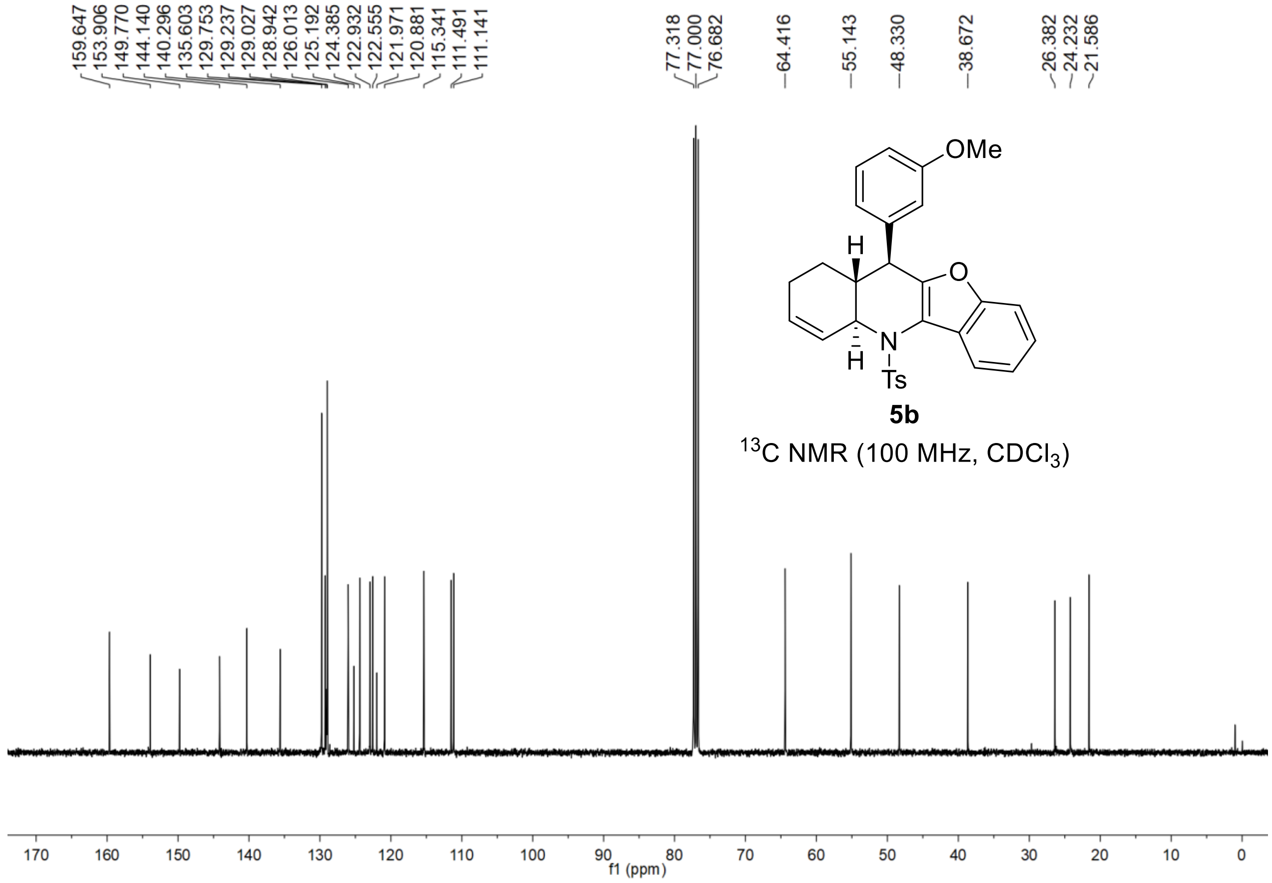
159.647 153.906 149.770 144.140 140.296 135.603 129.753 129.237 129.027 128.942 126.013 125.192 124.385 122.932 122.555 121.971 120.881 115.341 111.491 111.141
77.318 77.000 76.682
64.416
55.143
48.330
38.672
26.382 24.232 21.586
OMe
H
O
N
H
Ts
5b
^{13}C NMR (100 MHz, CDCl$_3$)
f1 (ppm)

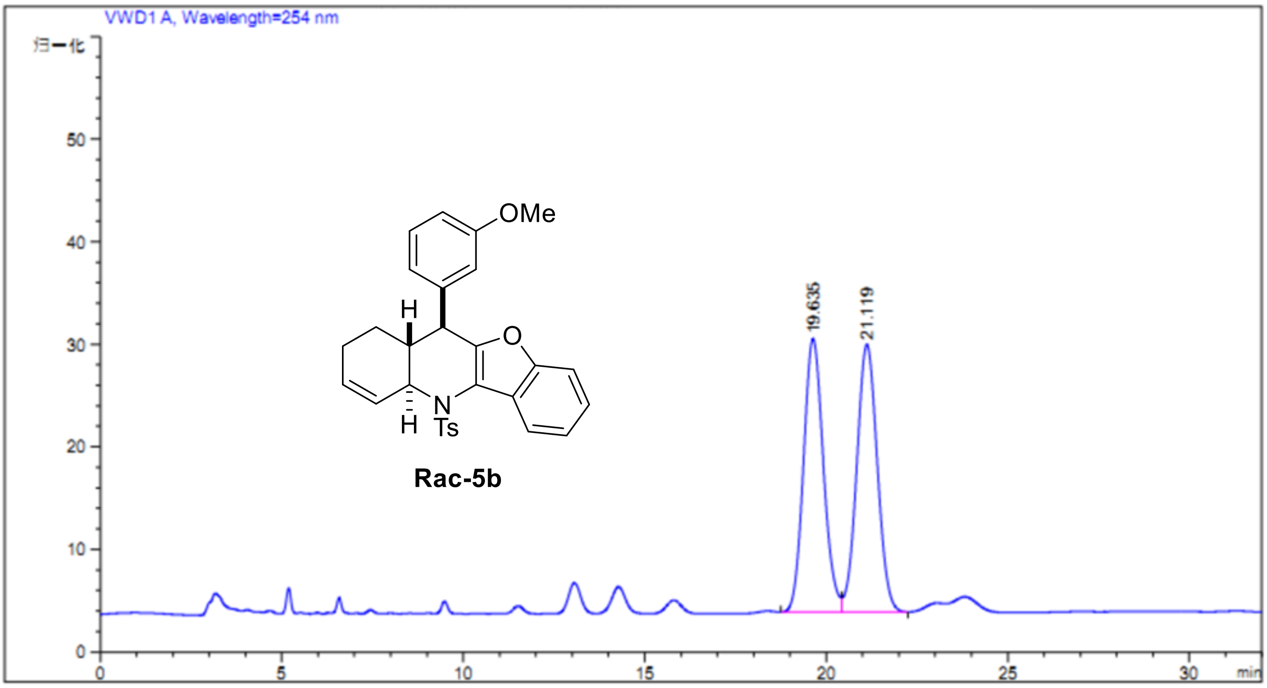

#	[min]		[min]	[mAU*s]	[mAU]	%
1	19.635	BV	0.5846	1015.80780	26.64064	49.7959
2	21.119	VB	0.6099	1024.13293	26.06264	50.2041

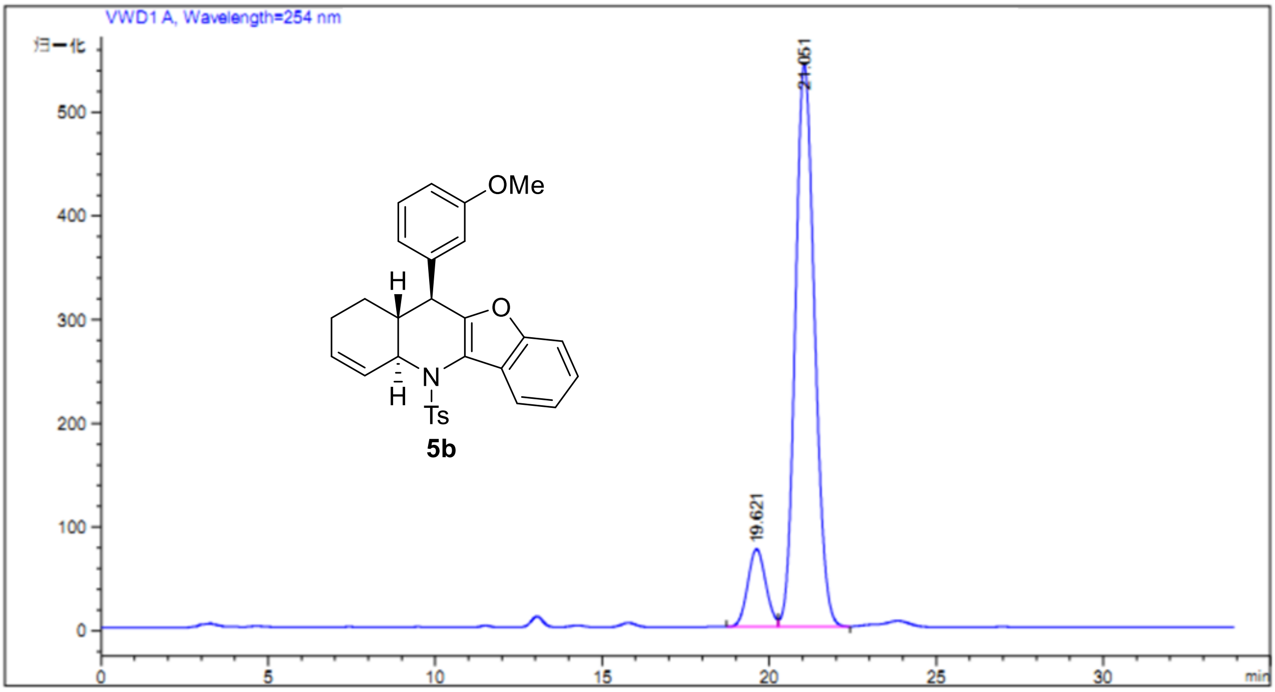

#	[min]		[min]	[mAU*s]	[mAU]	%
1	19.621	BV	0.5887	2819.46216	74.98166	11.4639
2	21.051	VB	0.6261	2.17748e4	541.86804	88.5361

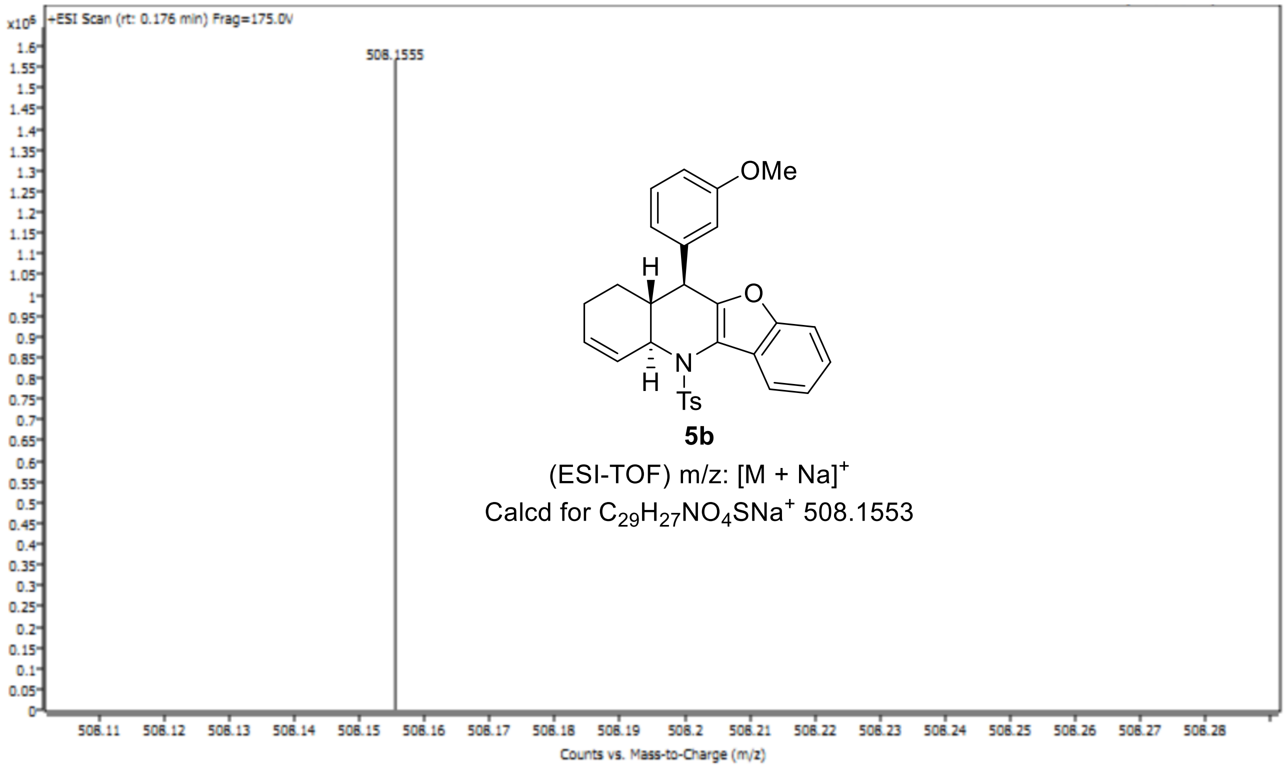

x10^6
+ESI Scan (rt: 0.176 min) Frag=175.0V
508.1555
OMe
H
O
H
N
Ts
5b
(ESI-TOF) m/z: [M + Na]+
Calcd for C29H27NO4SNa+ 508.1553
Counts vs. Mass-to-Charge (m/z)

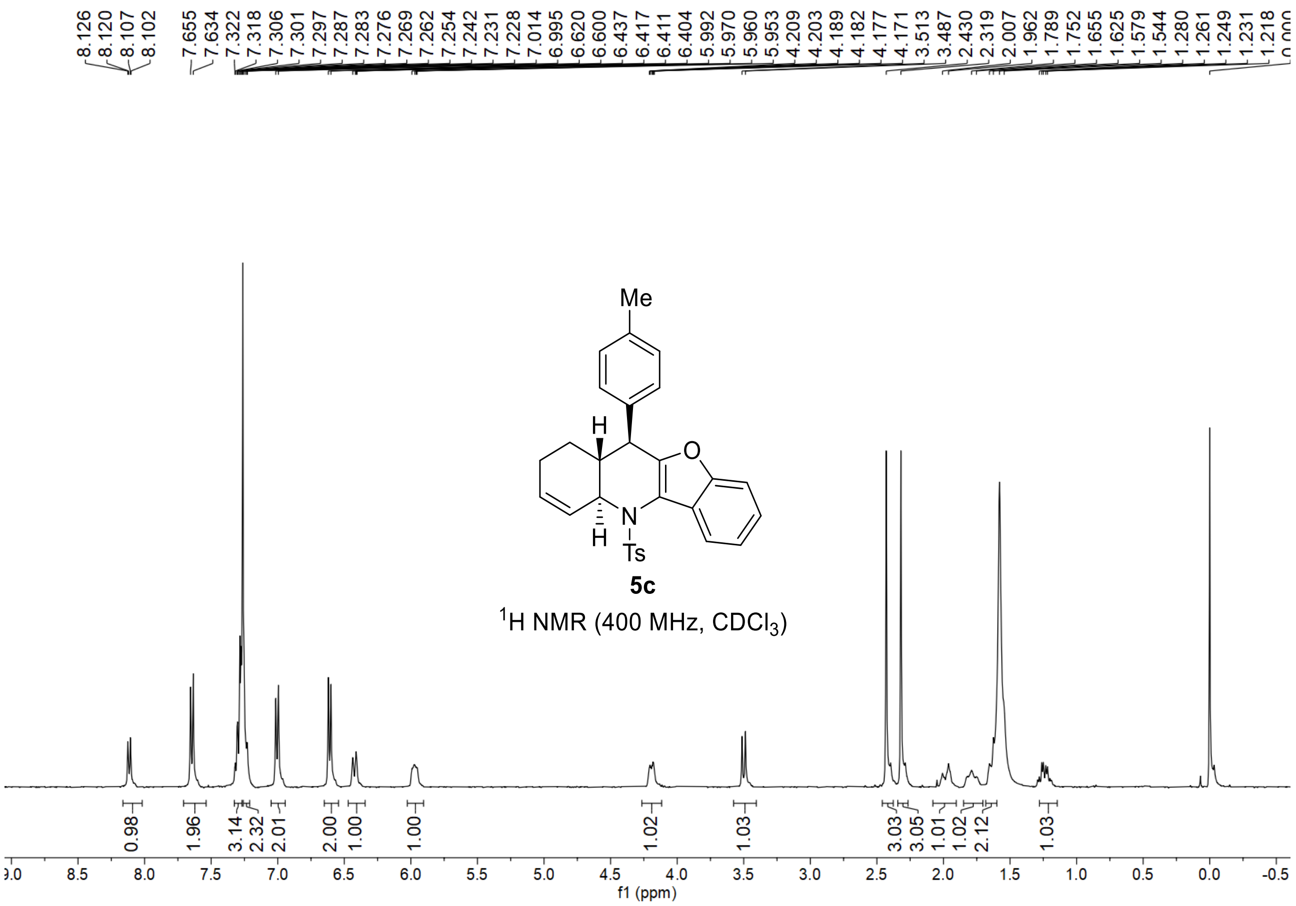
Me
H
O
N
H
Ts
5c
^{1}H NMR (400 MHz, CDCl$_3$)
f1 (ppm)

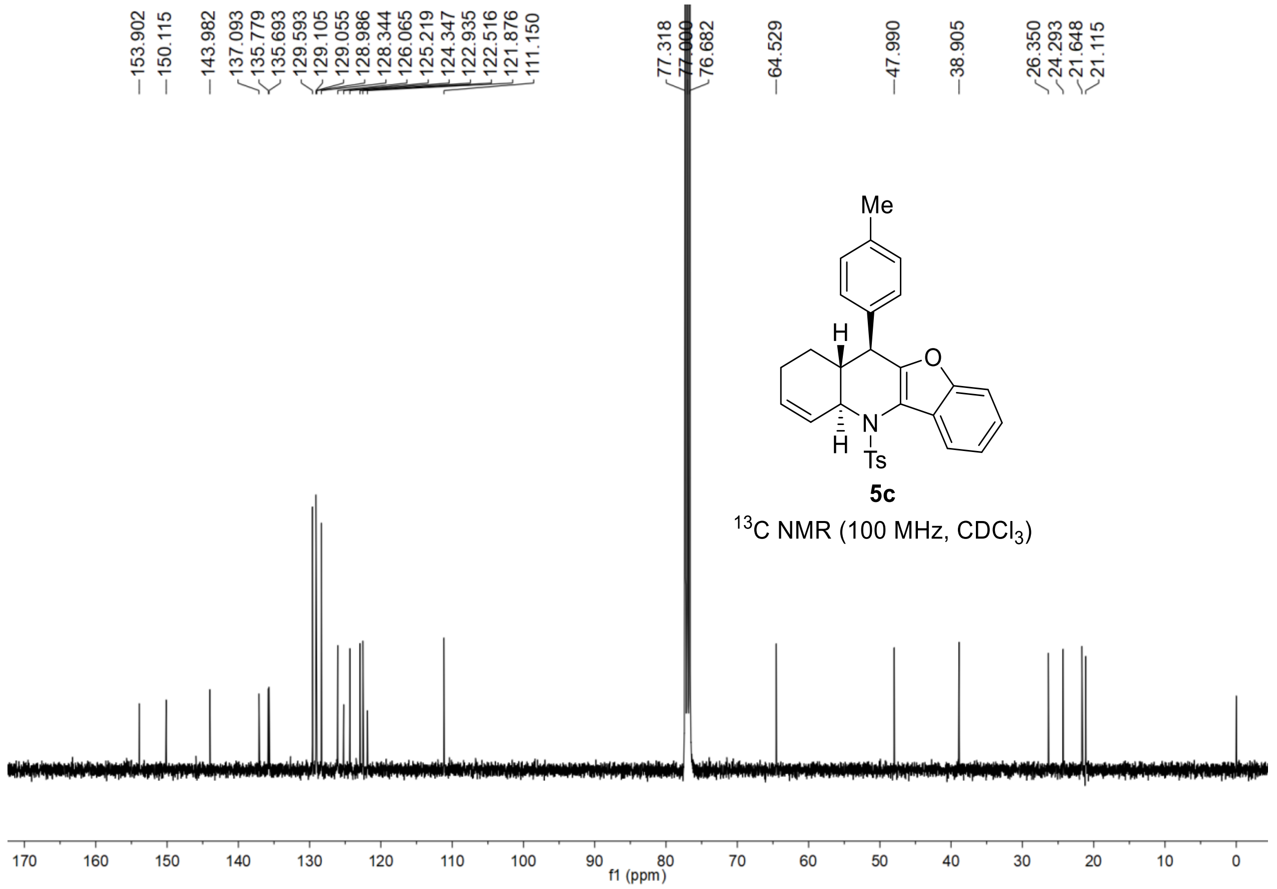
Me
H
O
N
H
Ts
5c
^{13}C NMR (100 MHz, CDCl$_3$)
f1 (ppm)

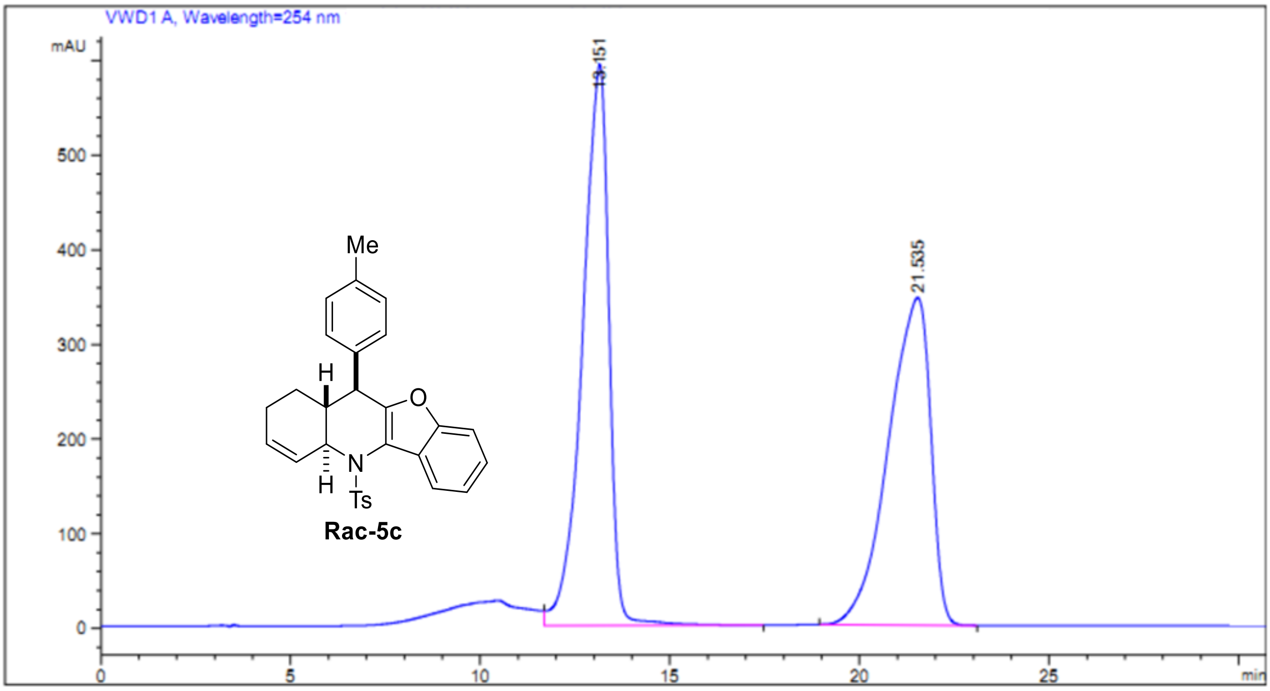

#	[min]		[min]	[mAU*s]	[mAU]	%
1	13.151	VB	0.7670	2.88403e4	592.89081	51.8688
2	21.535	BB	1.1689	2.67621e4	345.97769	48.1312

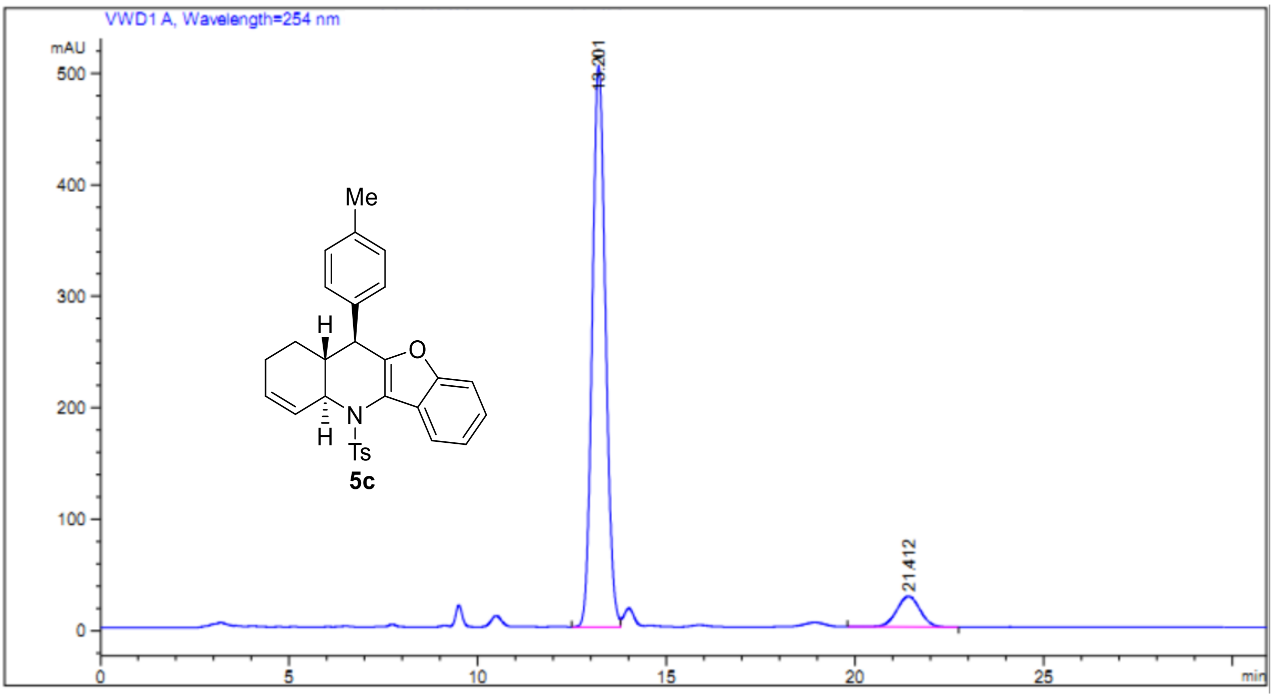

#	[min]		[min]	[mAU*s]	[mAU]	%
1	13.201	BV	0.3814	1.23008e4	502.99823	91.1729
2	21.412	BB	0.6764	1190.93762	27.53836	8.8271

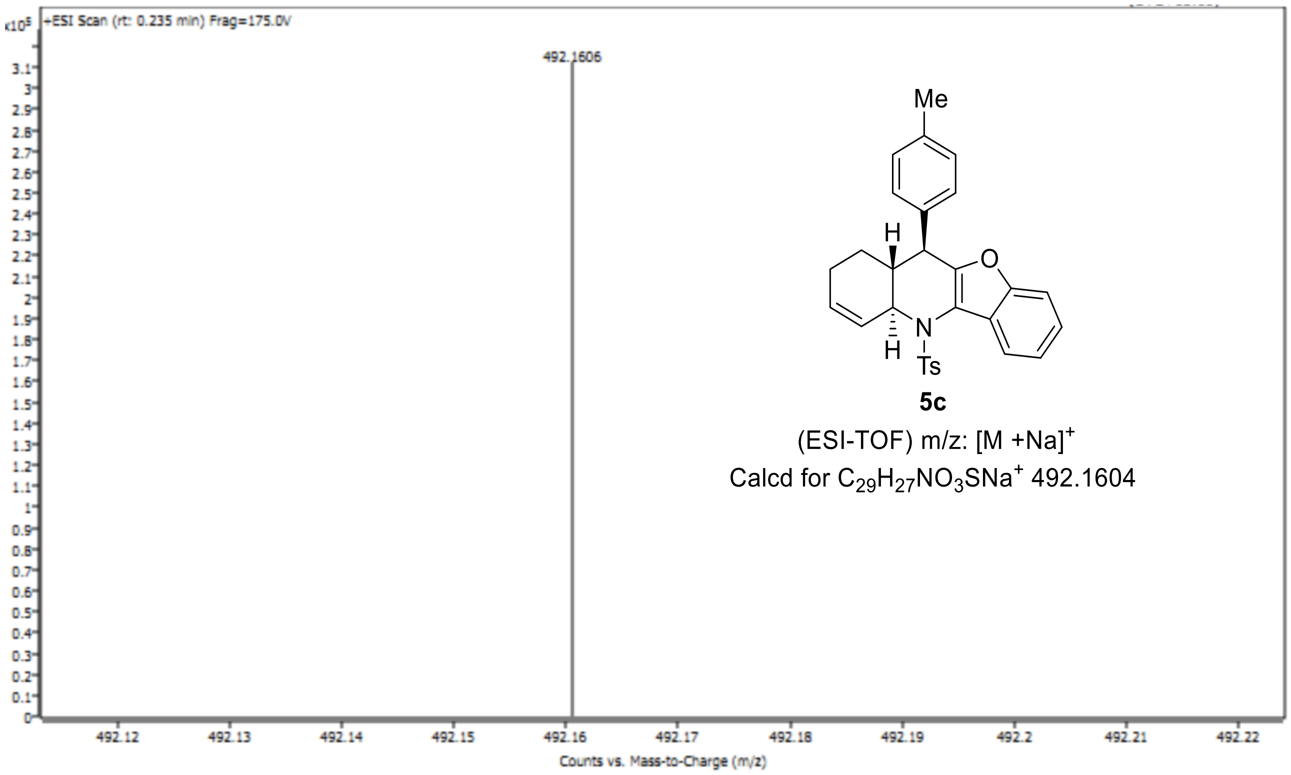
x10^5
+ESI Scan (rt: 0.235 min) Frag=175.0V
492.1606
Me
H
O
N
H
Ts
5c
(ESI-TOF) m/z: [M +Na]+
Calcd for C29H27NO3SNa+ 492.1604
Counts vs. Mass-to-Charge (m/z)

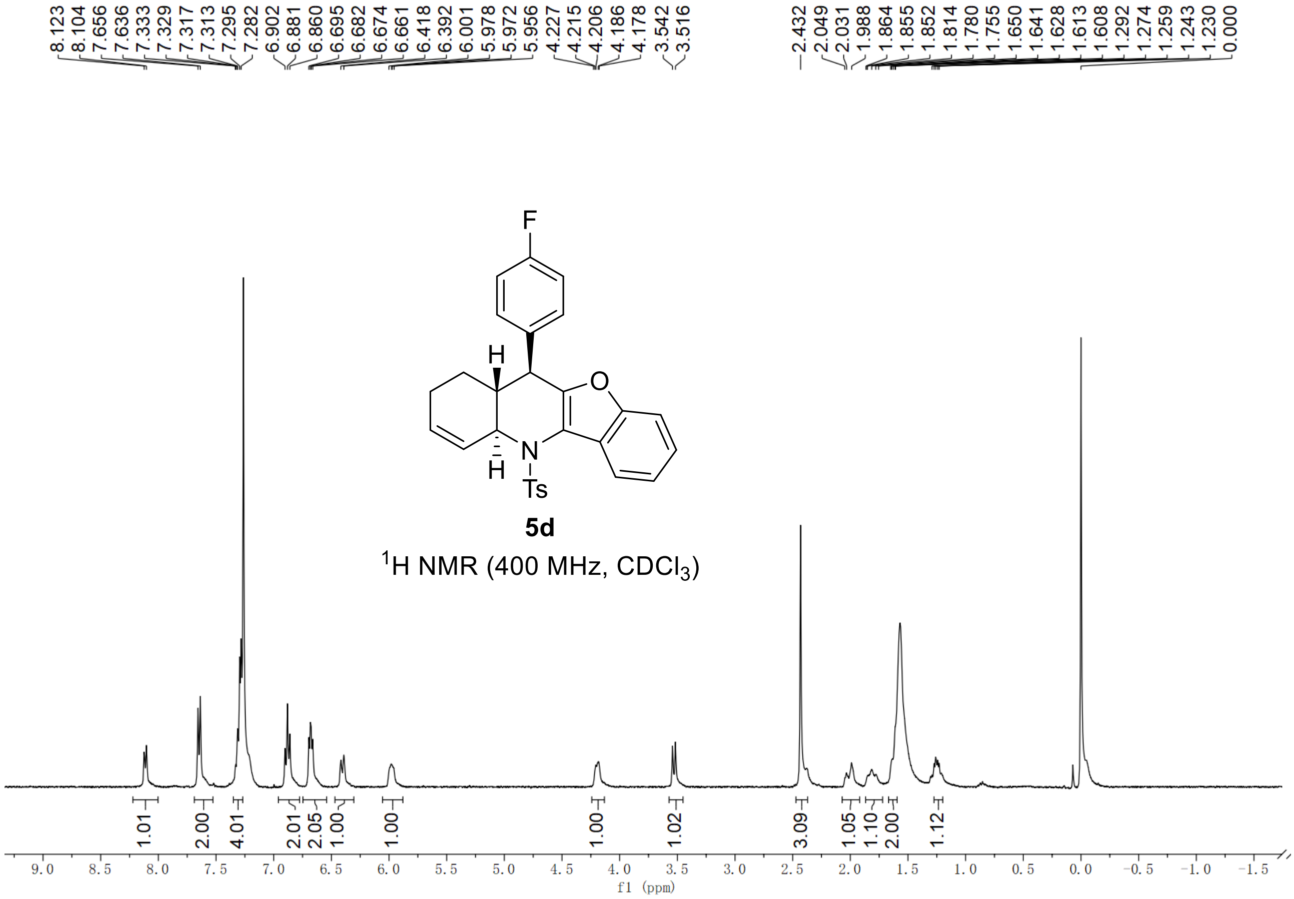
8.123 8.104 7.656 7.636 7.333 7.329 7.317 7.313 7.295 7.282 6.902 6.881 6.860 6.695 6.682 6.674 6.661 6.418 6.392 6.001 5.978 5.972 5.956 4.227 4.215 4.206 4.186 4.178 3.542 3.516
2.432 2.049 2.031 1.988 1.864 1.855 1.852 1.814 1.780 1.755 1.650 1.641 1.628 1.613 1.608 1.292 1.274 1.259 1.243 1.230 0.000
F
H
O
N
H
Ts
5d
1H NMR (400 MHz, CDCl3)
1.01 2.00 4.01 2.01 2.05 1.00 1.00 1.00 1.02 3.09 1.05 1.10 2.00 1.12
f1 (ppm)

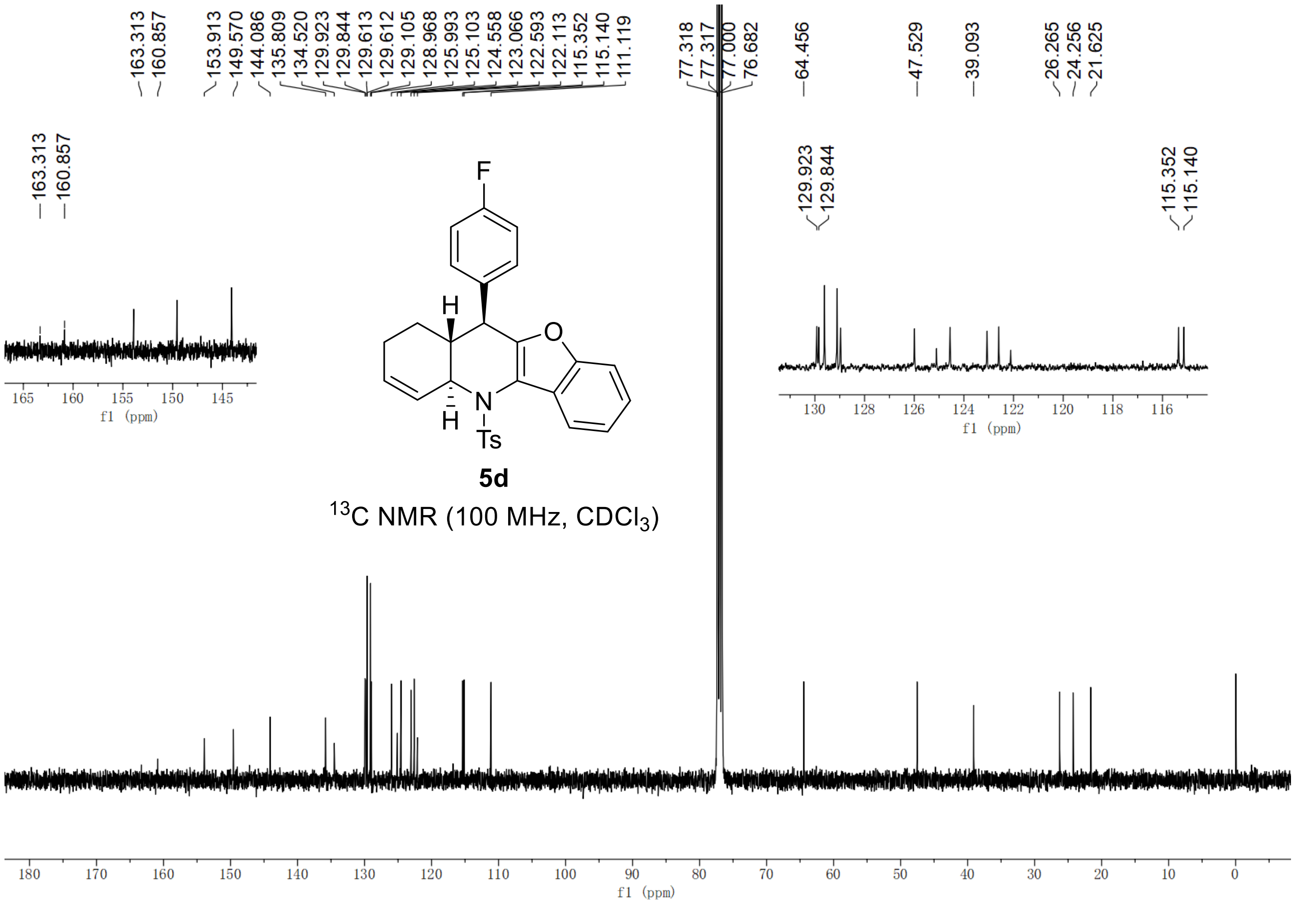
163.313 160.857 153.913 149.570 144.086 135.809 134.520 129.923 129.844 129.613 129.612 129.105 128.968 125.993 125.103 124.558 123.066 122.593 122.113 115.352 115.140 111.119
77.318 77.317 77.000 76.682 64.456 47.529 39.093 26.265 24.256 21.625
163.313 160.857
129.923 129.844
115.352 115.140
F
H
O
N
H
Ts
5d
13C NMR (100 MHz, CDCl3)
f1 (ppm)

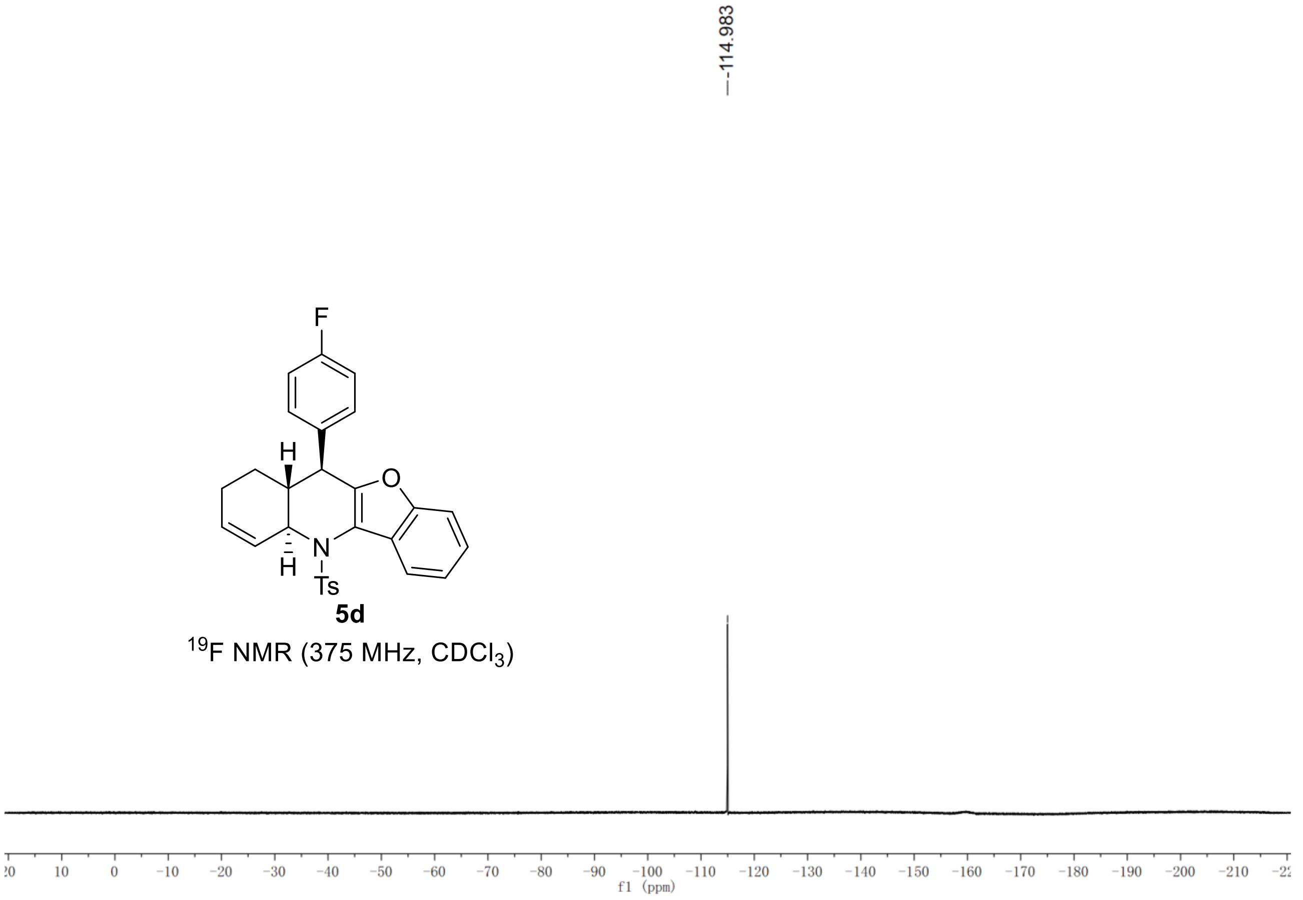

-114.983
F
H
O
H
N
Ts
5d
^{19}F NMR (375 MHz, $CDCl_3$)
f1 (ppm)

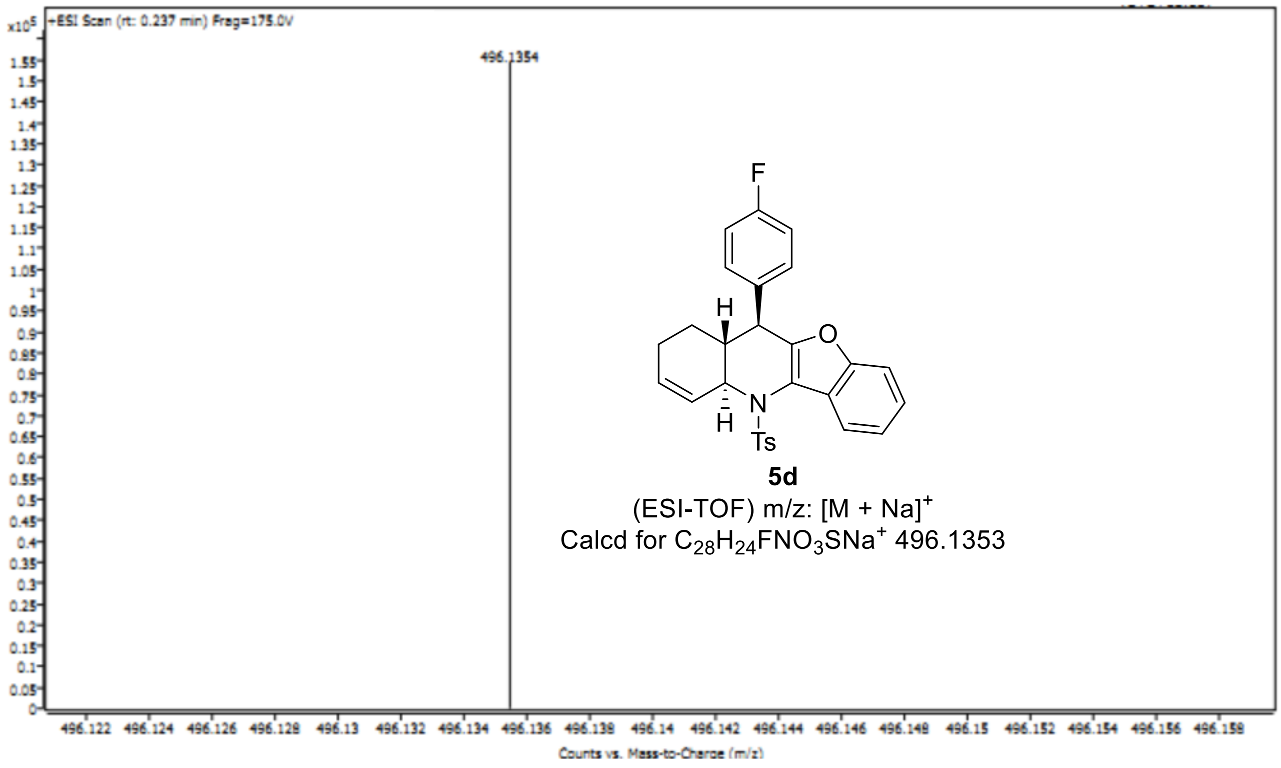

+ESI Scan (rt: 0.237 min) Frag=175.0V
496.1354
F
H
O
H
N
Ts
5d
(ESI-TOF) m/z: $[M + Na]^+$
Calcd for $C_{28}H_{24}FNO_3SNa^+$ 496.1353
Counts vs. Mass-to-Charge (m/z)

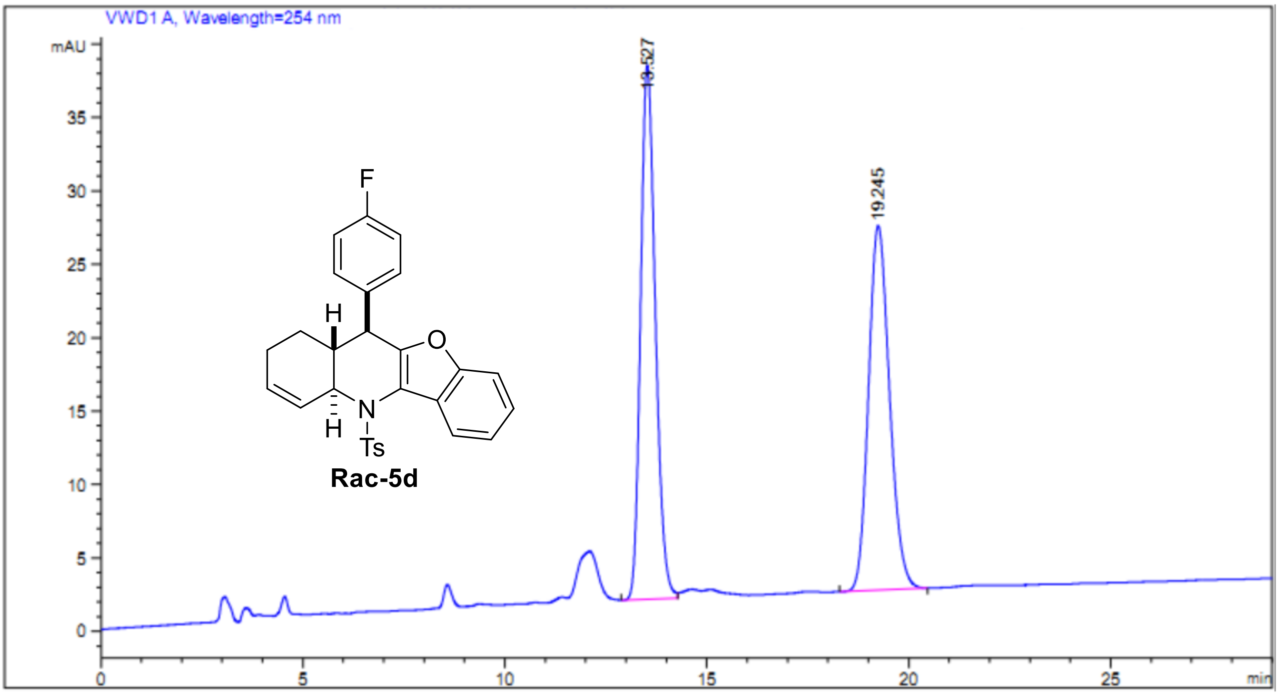

#	[min]		[min]	[mAU*s]	[mAU]	%
1	13.527	BV	0.3923	923.65717	36.37949	50.6615
2	19.245	BB	0.5595	899.53528	24.83106	49.3385

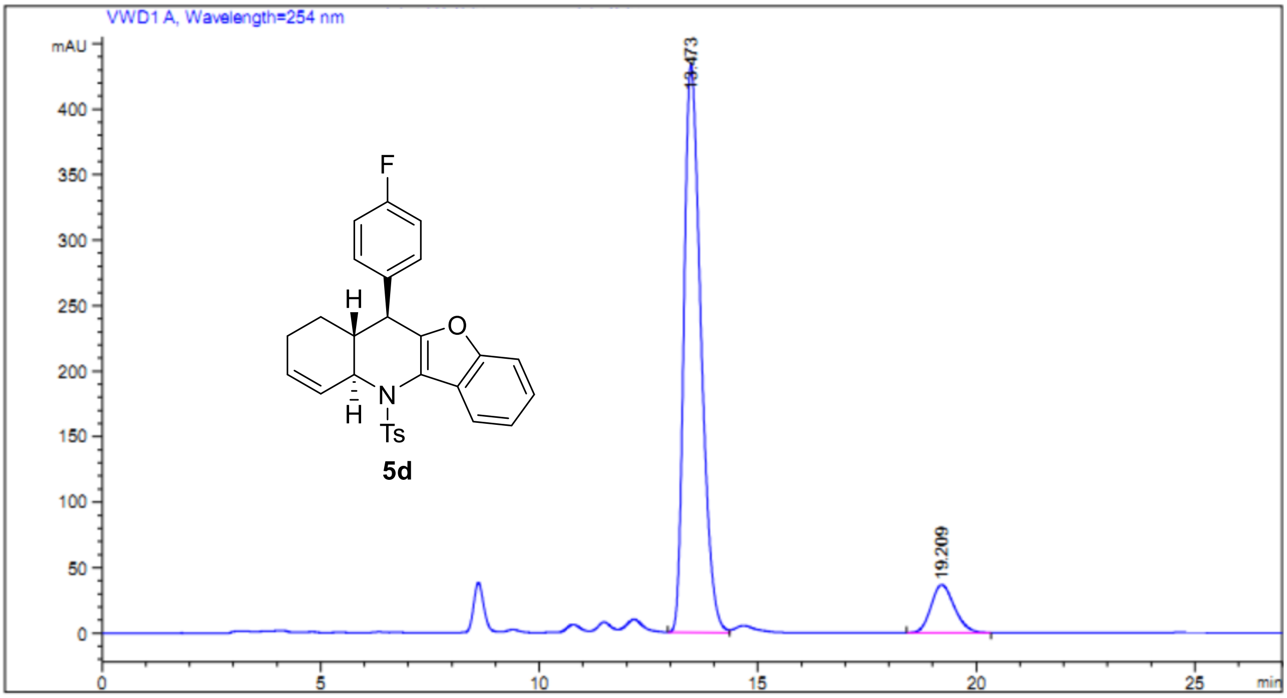

#	[min]		[min]	[mAU*s]	[mAU]	%
1	13.473	BV	0.4199	1.16948e4	433.10706	89.5814
2	19.209	BB	0.5723	1360.13989	36.68816	10.4186

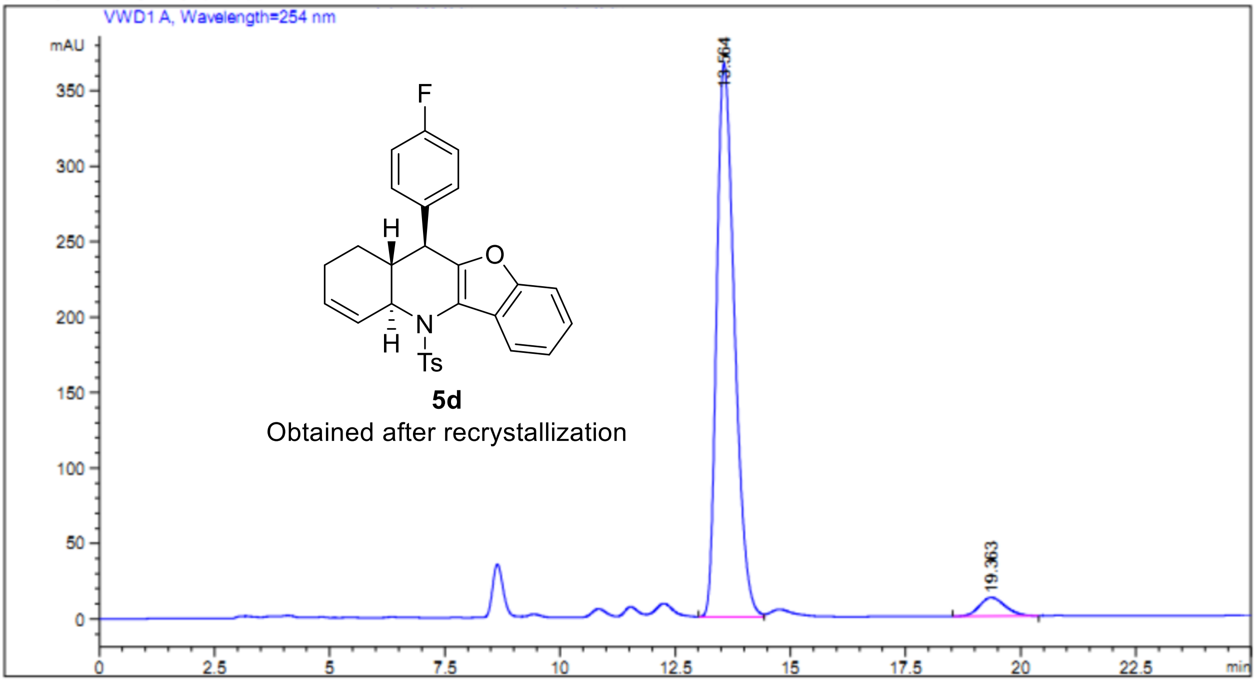

#	[min]		[min]	[mAU*s]	[mAU]	%
1	13.564	BV	0.4205	9921.04980	366.71774	95.3080
2	19.363	VV	0.6040	488.41245	12.43148	4.6920

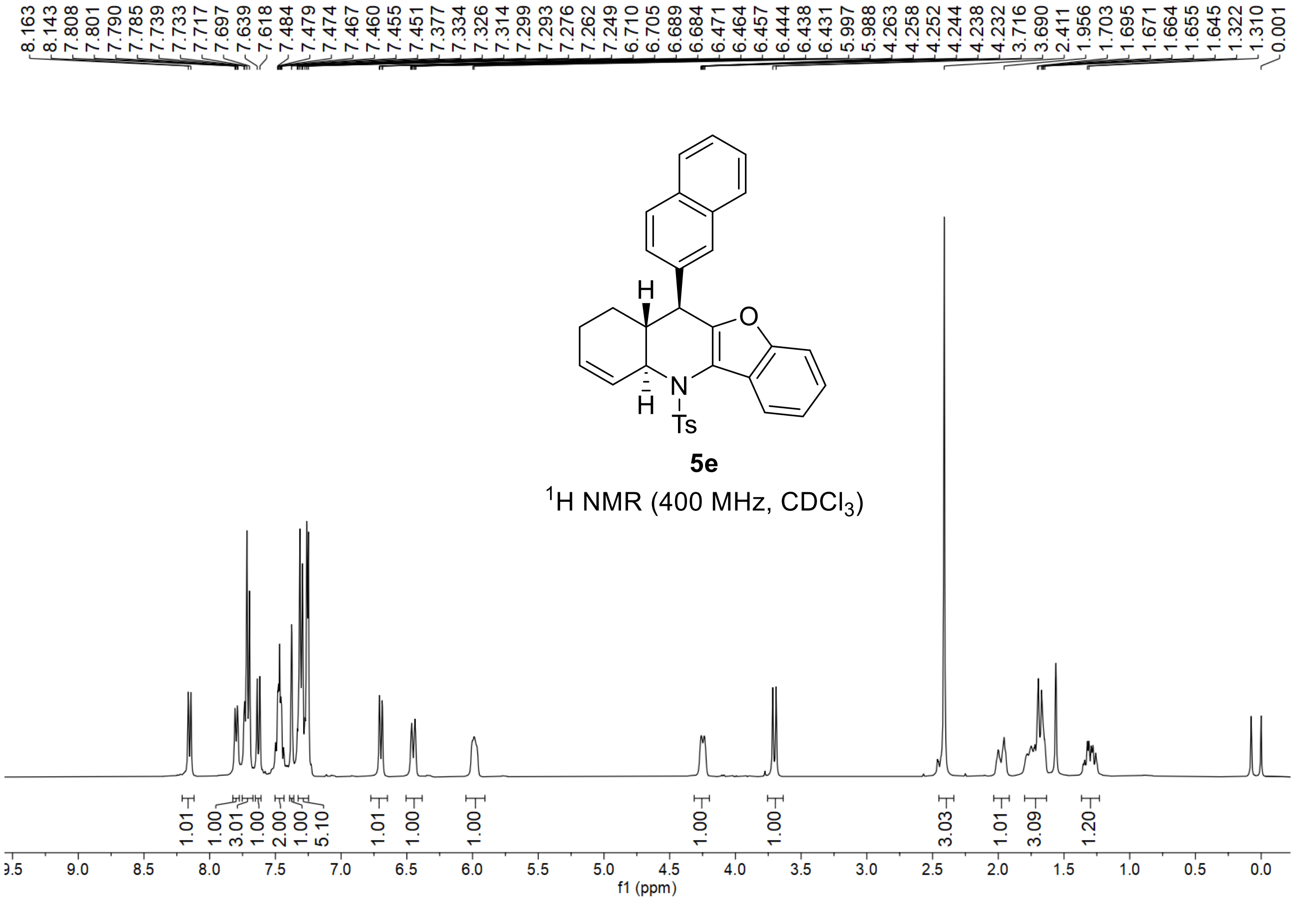
8.163 8.143 7.808 7.801 7.790 7.785 7.739 7.733 7.717 7.697 7.639 7.618 7.484 7.479 7.474 7.467 7.460 7.455 7.451 7.377 7.334 7.326 7.314 7.299 7.293 7.276 7.262 7.249 6.710 6.705 6.689 6.684 6.471 6.464 6.457 6.444 6.438 6.431 5.997 5.988 4.263 4.258 4.252 4.244 4.238 4.232 3.716 3.690 2.411 1.956 1.703 1.695 1.671 1.664 1.655 1.645 1.322 1.310 0.001
H
O
N
H
Ts
5e
1H NMR (400 MHz, CDCl3)
1.01 1.00 3.01 1.00 2.00 1.00 5.10 1.01 1.00 1.00 1.00 1.00 3.03 1.01 3.09 1.20
9.5 9.0 8.5 8.0 7.5 7.0 6.5 6.0 5.5 5.0 4.5 4.0 3.5 3.0 2.5 2.0 1.5 1.0 0.5 0.0
f1 (ppm)

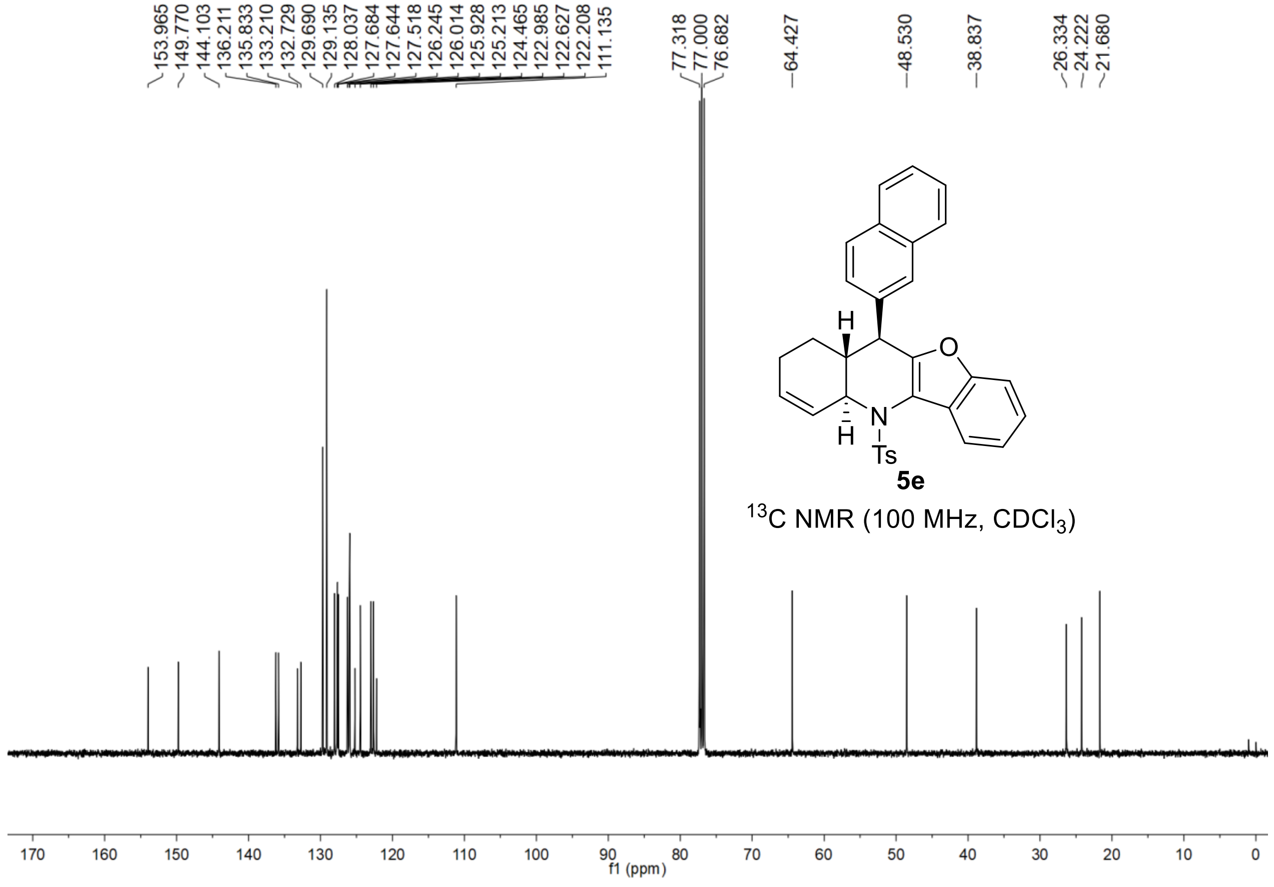
153.965 149.770 144.103 136.211 135.833 133.210 132.729 129.690 129.135 128.037 127.684 127.644 127.518 126.245 126.014 125.928 125.213 124.465 122.985 122.627 122.208 111.135 77.318 77.000 76.682 64.427 48.530 38.837 26.334 24.222 21.680
H
O
N
H
Ts
5e
13C NMR (100 MHz, CDCl3)
170 160 150 140 130 120 110 100 90 80 70 60 50 40 30 20 10 0
f1 (ppm)

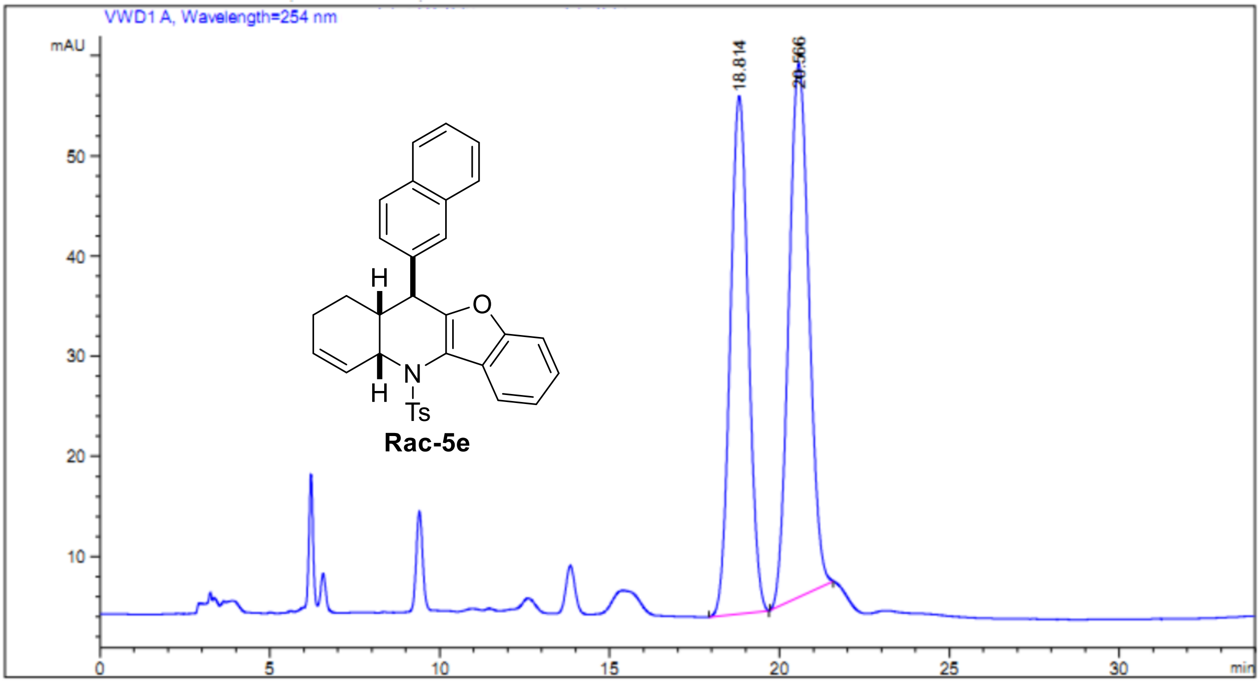

#	[min]		[min]	[mAU*s]	[mAU]	%
1	18.814	BB	0.5806	1914.38477	51.69145	46.5791
2	20.566	BBA	0.6454	2195.57861	53.27122	53.4209

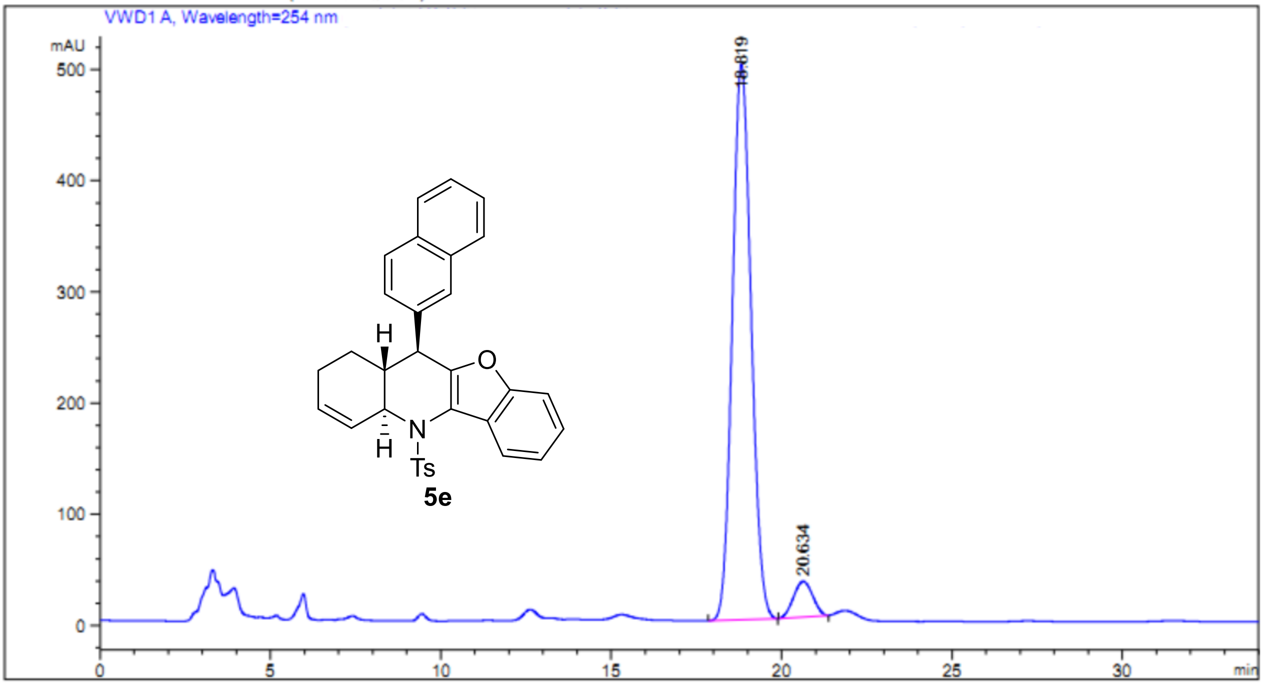

#	[min]		[min]	[mAU*s]	[mAU]	%
1	18.819	BB	0.5998	1.91467e4	499.76703	93.8734
2	20.634	BB	0.6256	1249.60059	32.02076	6.1266

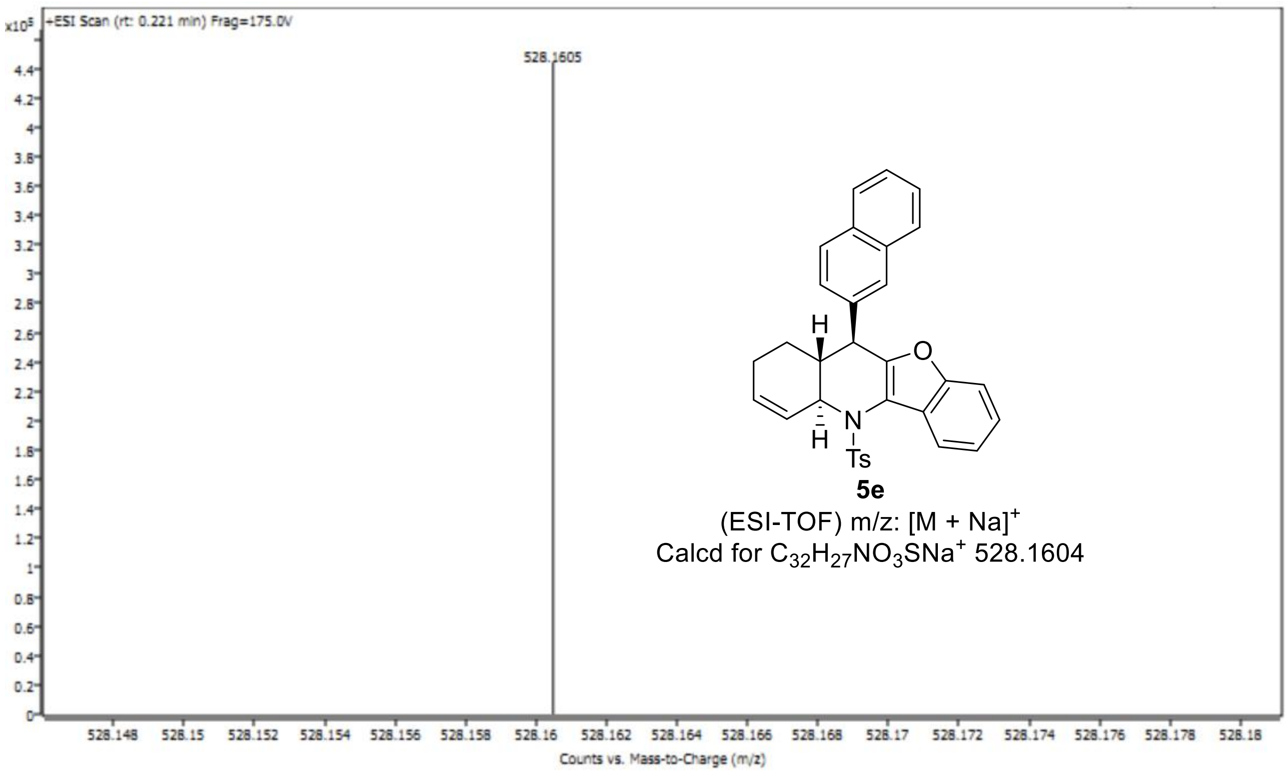

x10^5
+ESI Scan (rt: 0.221 min) Frag=175.0V
528.1605
Counts vs. Mass-to-Charge (m/z)
5e
(ESI-TOF) m/z: [M + Na]$^+$
Calcd for $C_{32}H_{27}NO_3SNa^+$ 528.1604

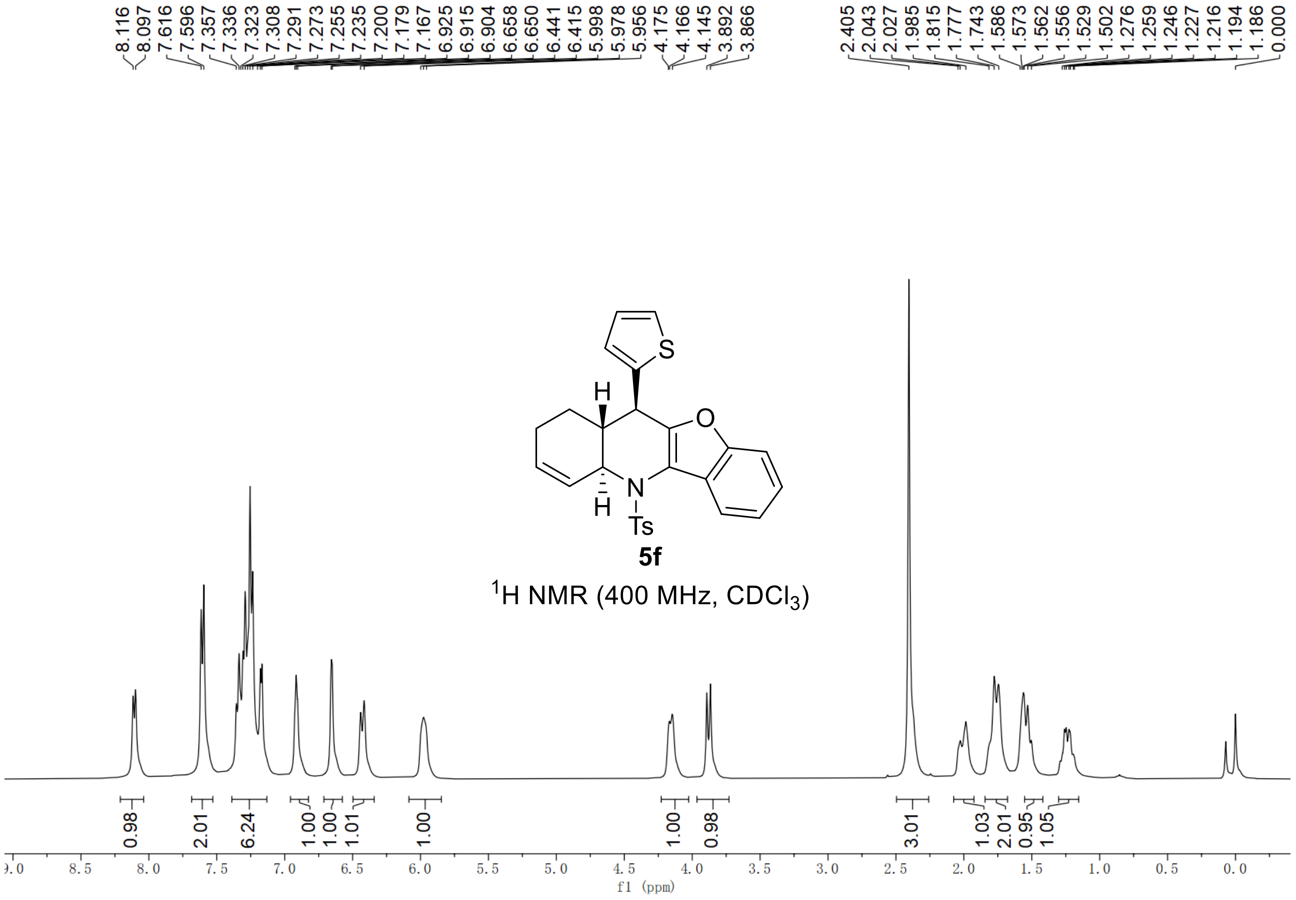
8.116 8.097 7.616 7.596 7.357 7.336 7.323 7.308 7.291 7.273 7.255 7.235 7.200 7.179 7.167 6.925 6.915 6.904 6.658 6.650 6.441 6.415 5.998 5.978 5.956 4.175 4.166 4.145 3.892 3.866
2.405 2.043 2.027 1.985 1.815 1.777 1.743 1.586 1.573 1.562 1.556 1.529 1.502 1.276 1.259 1.246 1.227 1.216 1.194 1.186 0.000
H
S
O
N
H
Ts
5f
1H NMR (400 MHz, CDCl3)
0.98 2.01 6.24 1.00 1.00 1.01 1.00 1.00 0.98 3.01 1.03 2.01 0.95 1.05
f1 (ppm)

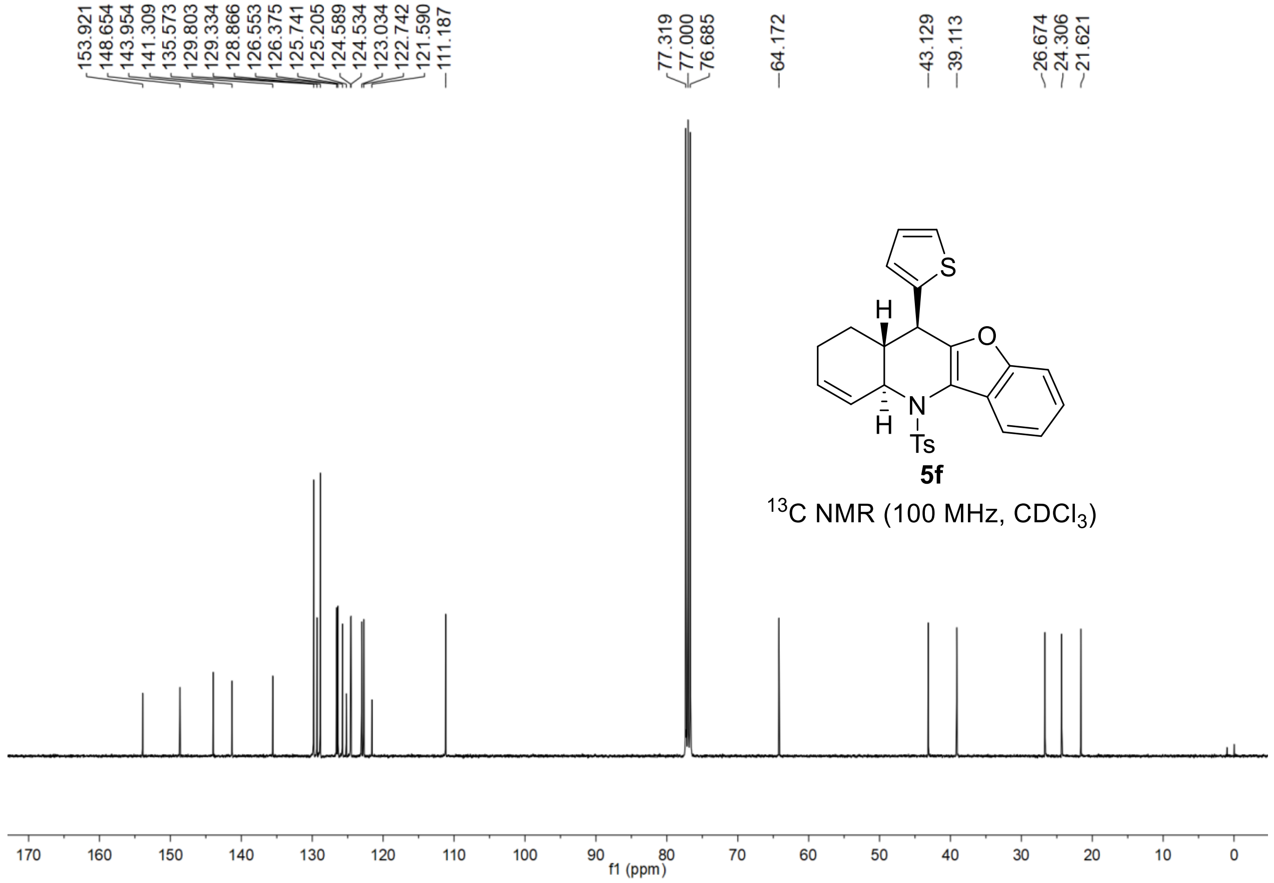
153.921 148.654 143.954 141.309 135.573 129.803 129.334 128.866 126.553 126.375 125.741 125.205 124.589 124.534 123.034 122.742 121.590 111.187
77.319 77.000 76.685 64.172 43.129 39.113 26.674 24.306 21.621
H
S
O
N
H
Ts
5f
13C NMR (100 MHz, CDCl3)
170 160 150 140 130 120 110 100 90 80 70 60 50 40 30 20 10 0
f1 (ppm)

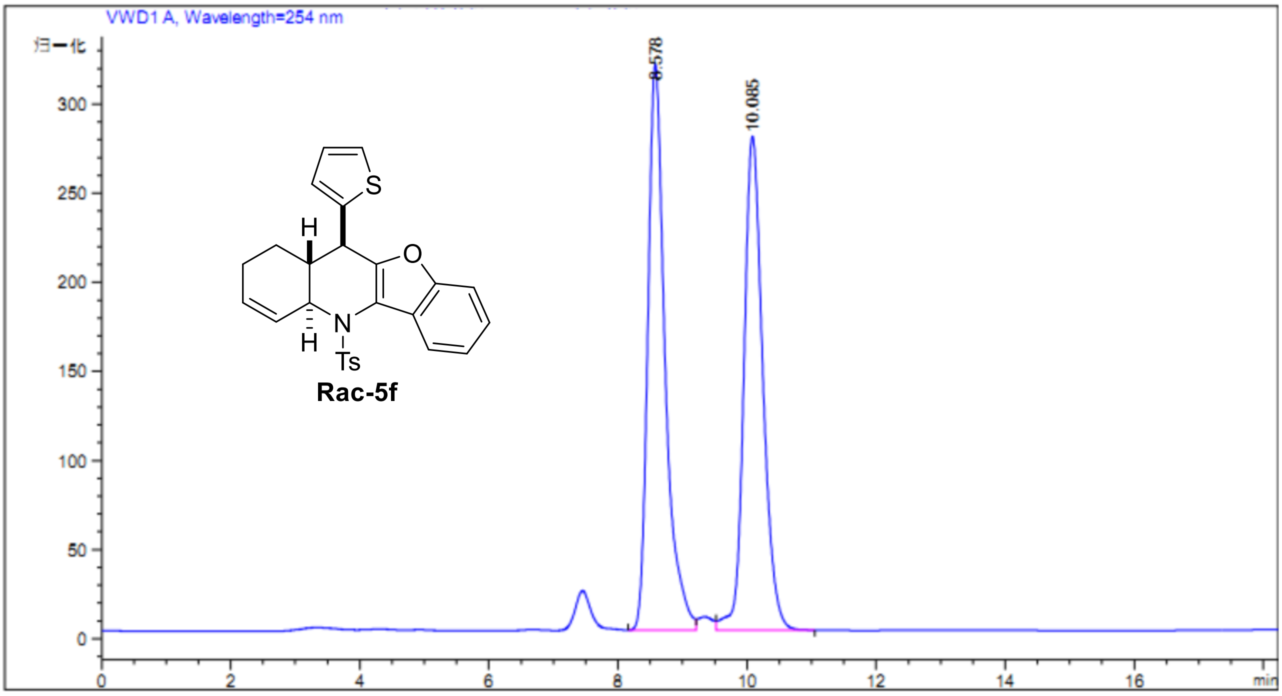

#	[min]		[min]	[mAU*s]	[mAU]	%
1	8.578	BV	0.2796	5904.43262	317.42151	51.1398
2	10.085	VB	0.3103	5641.23438	277.06821	48.8602

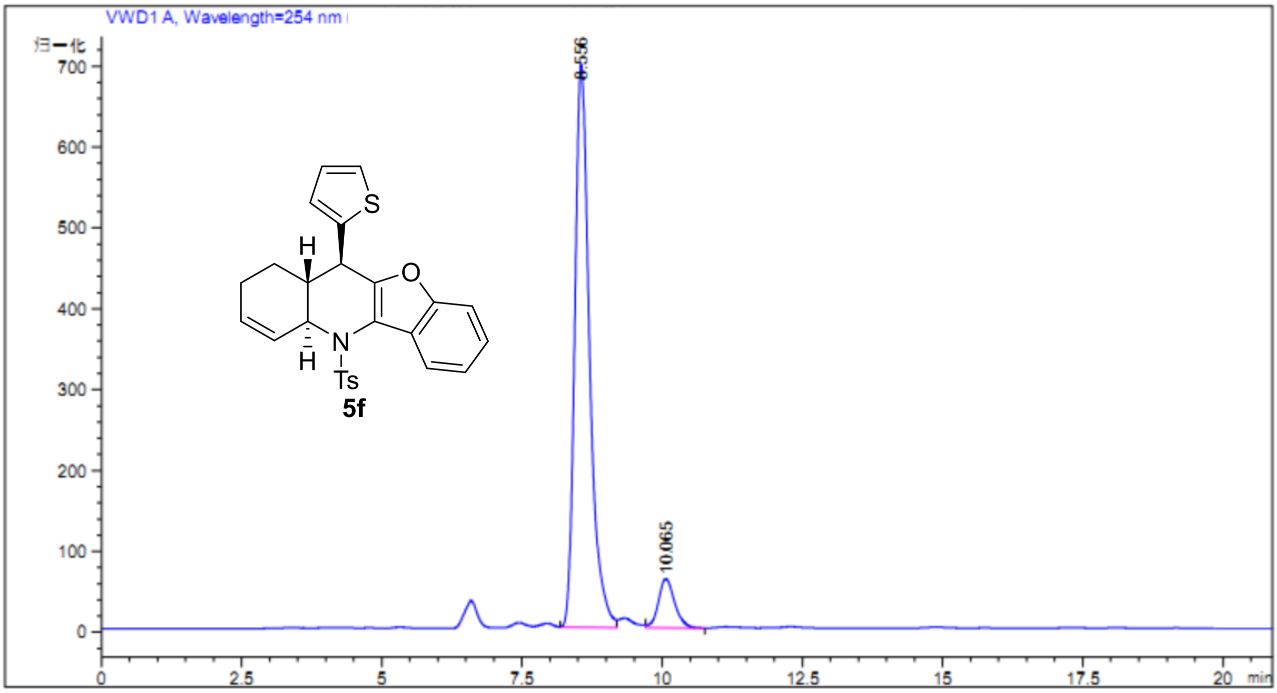

#	[min]		[min]	[mAU*s]	[mAU]	%
1	8.556	BV	0.2773	1.27241e4	696.05701	91.2607
2	10.065	VB	0.3049	1218.48169	60.47950	8.7393

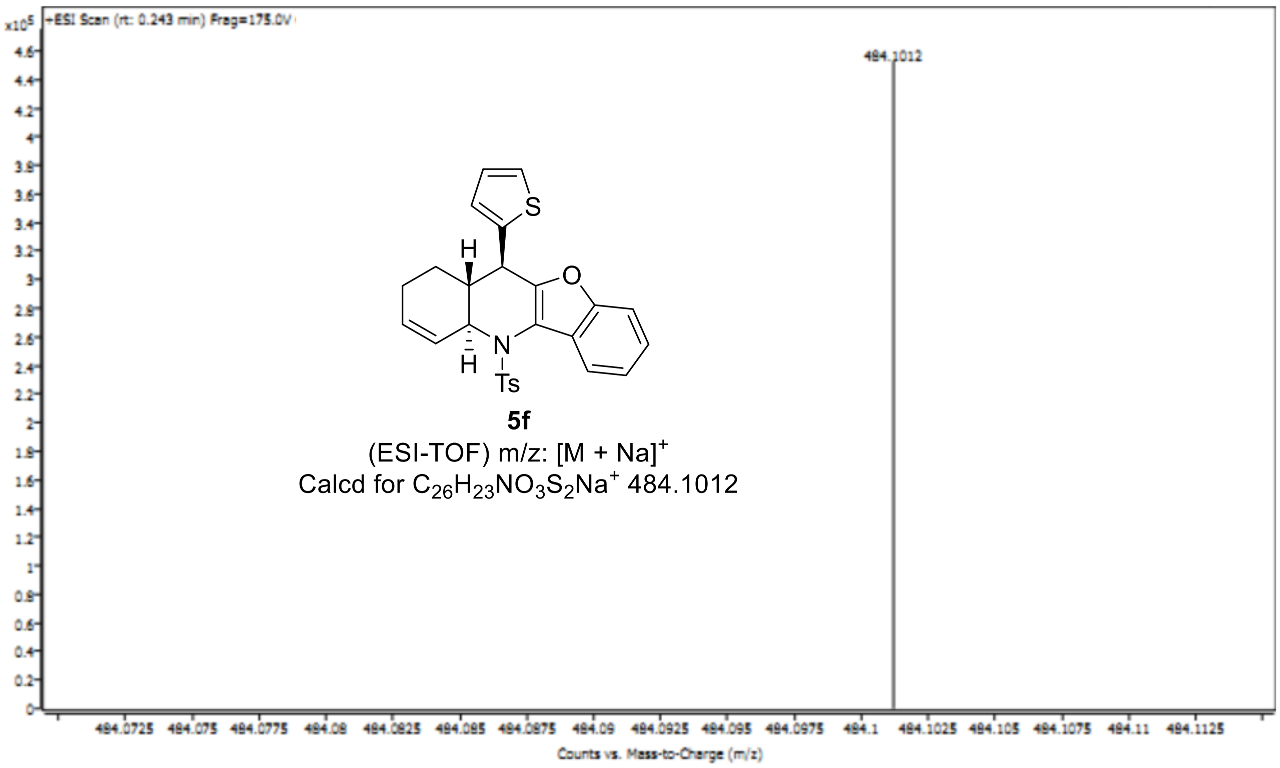

+ESI Scan (rt: 0.243 min) Frag=175.0V
x10^5
484.1012
5f
(ESI-TOF) m/z: [M + Na]+
Calcd for $C_{26}H_{23}NO_3S_2Na^+$ 484.1012
Counts vs. Mass-to-Charge (m/z)

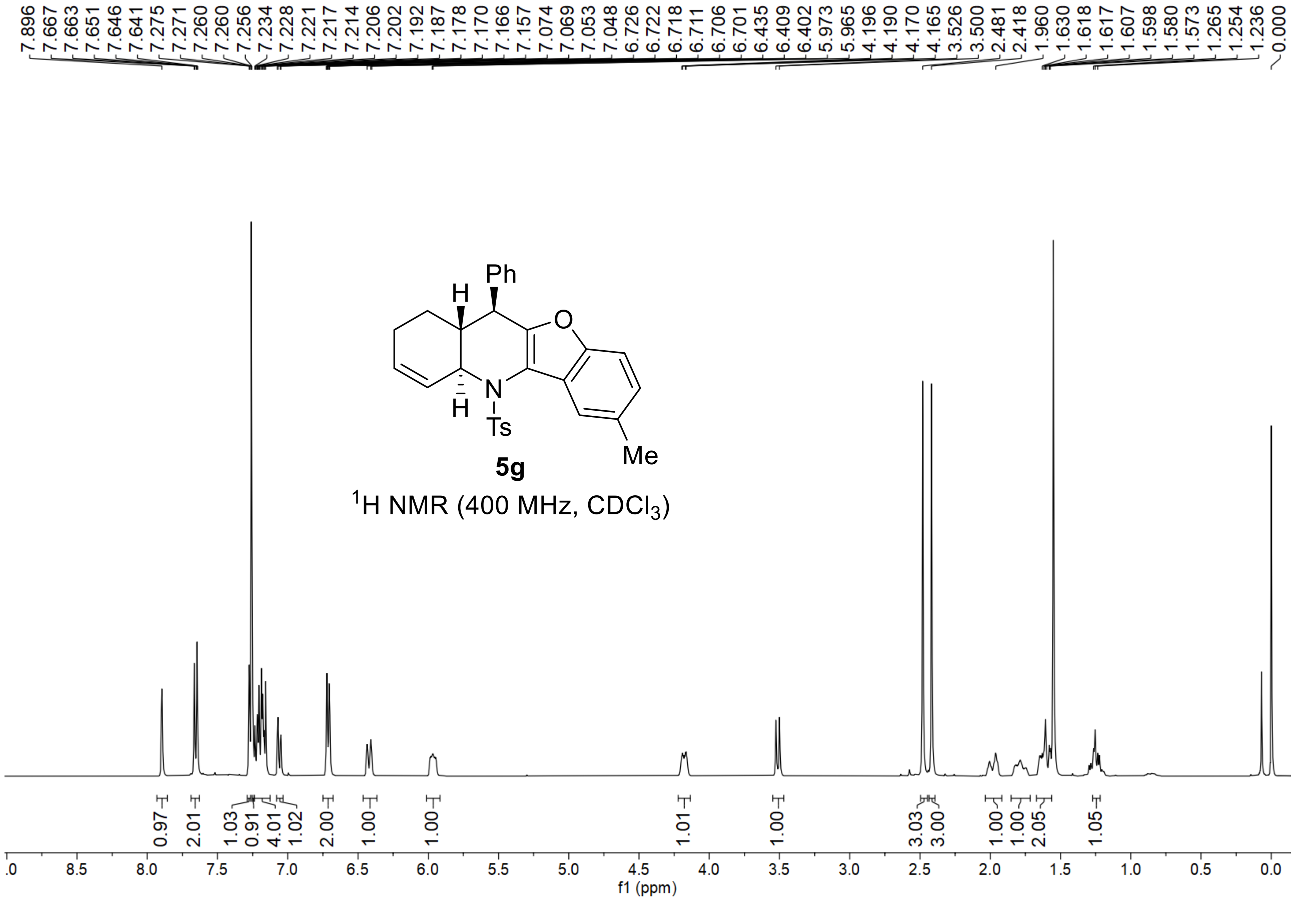

7.896
7.667
7.663
7.651
7.646
7.641
7.275
7.271
7.260
7.260
7.256
7.234
7.228
7.221
7.217
7.214
7.206
7.202
7.192
7.187
7.178
7.170
7.166
7.157
7.074
7.069
7.053
7.048
6.726
6.722
6.718
6.711
6.706
6.701
6.435
6.409
6.402
5.973
5.965
4.196
4.190
4.170
4.165
3.526
3.500
2.481
2.418
1.960
1.630
1.618
1.617
1.607
1.598
1.580
1.573
1.265
1.254
1.236
0.000
Ph
H
O
N
H
Ts
Me
5g
1H NMR (400 MHz, CDCl3)
0.97
2.01
1.03
0.91
4.01
1.02
2.00
1.00
1.00
1.01
1.00
3.03
3.00
1.00
1.00
2.05
1.05
f1 (ppm)

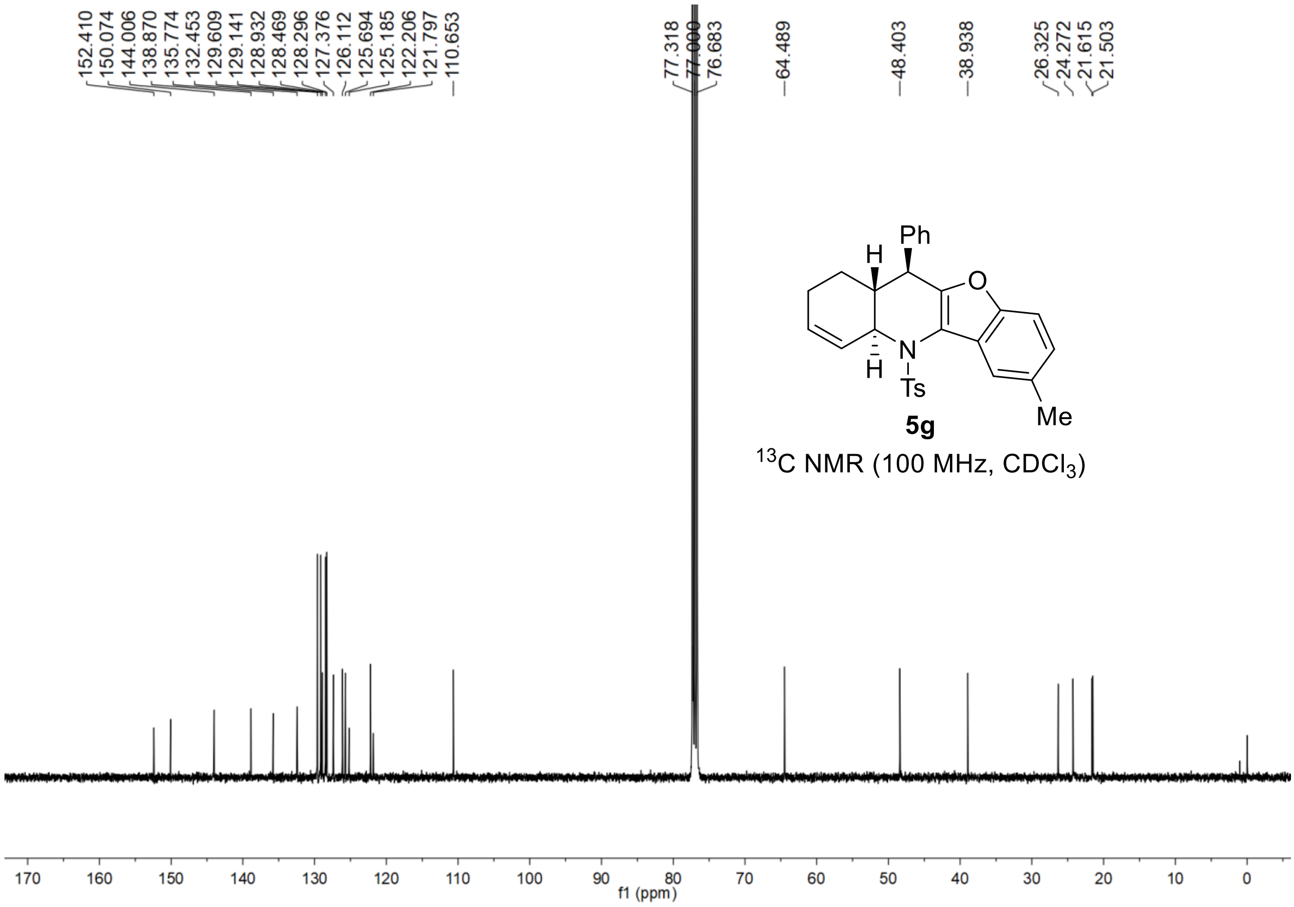

152.410
150.074
144.006
138.870
135.774
132.453
129.609
129.141
128.932
128.469
128.296
127.376
126.112
125.694
125.185
122.206
121.797
110.653
77.318
77.000
76.683
64.489
48.403
38.938
26.325
24.272
21.615
21.503
Ph
H
O
N
H
Ts
Me
5g
13C NMR (100 MHz, CDCl3)
f1 (ppm)

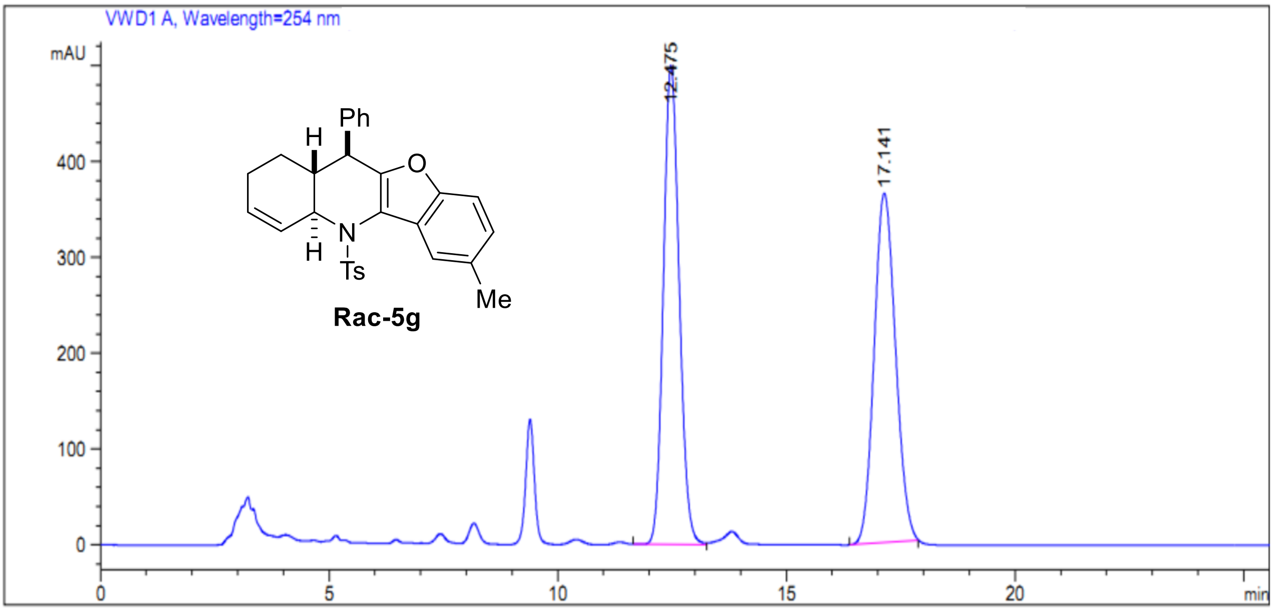

#	[min]		[min]	[mAU*s]	[mAU]	%
1	12.475	BV	0.3732	1.19901e4	499.58093	50.3257
2	17.141	BBA	0.5070	1.18349e4	364.45029	49.6743

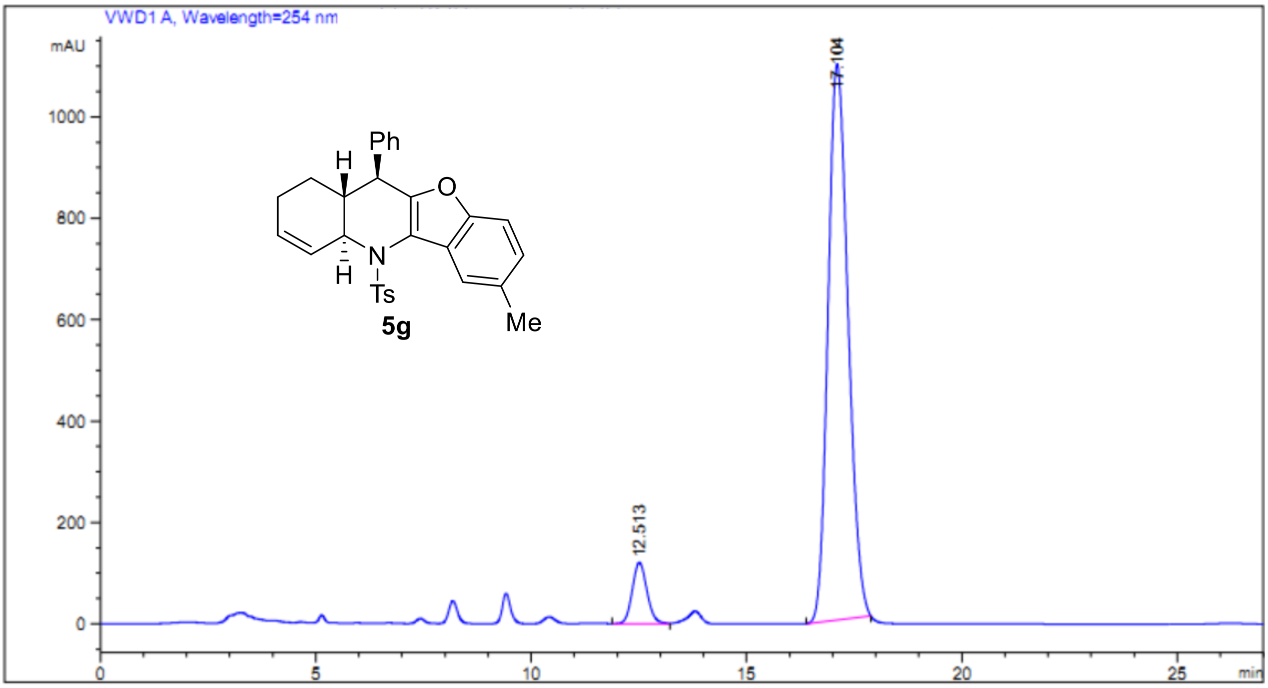

#	[min]		[min]	[mAU*s]	[mAU]	%
1	12.513	VV	0.3693	2898.59570	121.19361	7.5731
2	17.104	BBA	0.5046	3.53765e4	1096.43103	92.4269

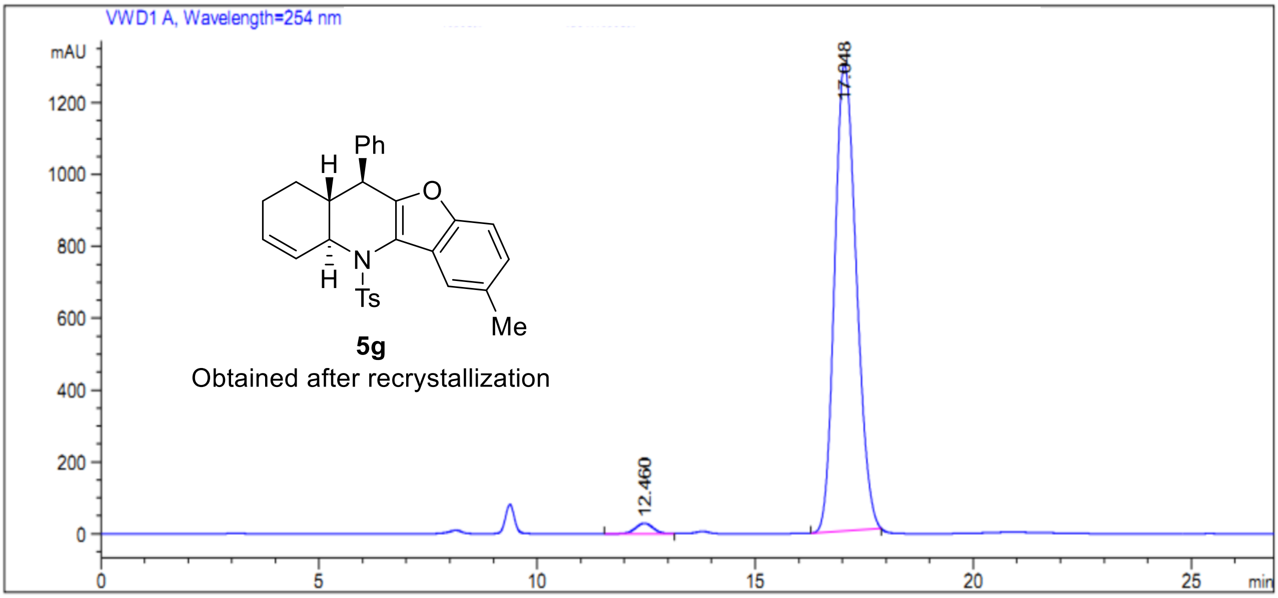

#	[min]		[min]	[mAU*s]	[mAU]	%
1	12.460	BV	0.4348	822.14380	29.32762	1.7954
2	17.048	BBA	0.5403	4.49707e4	1300.54907	98.2046

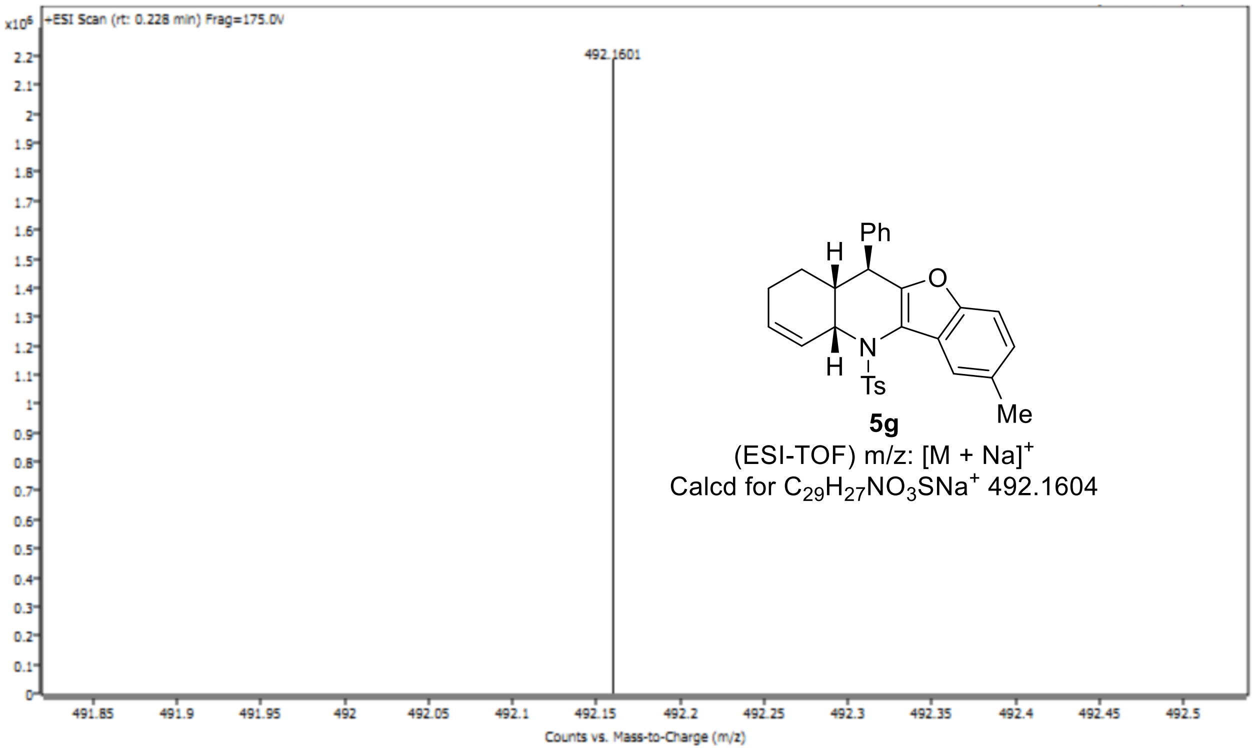

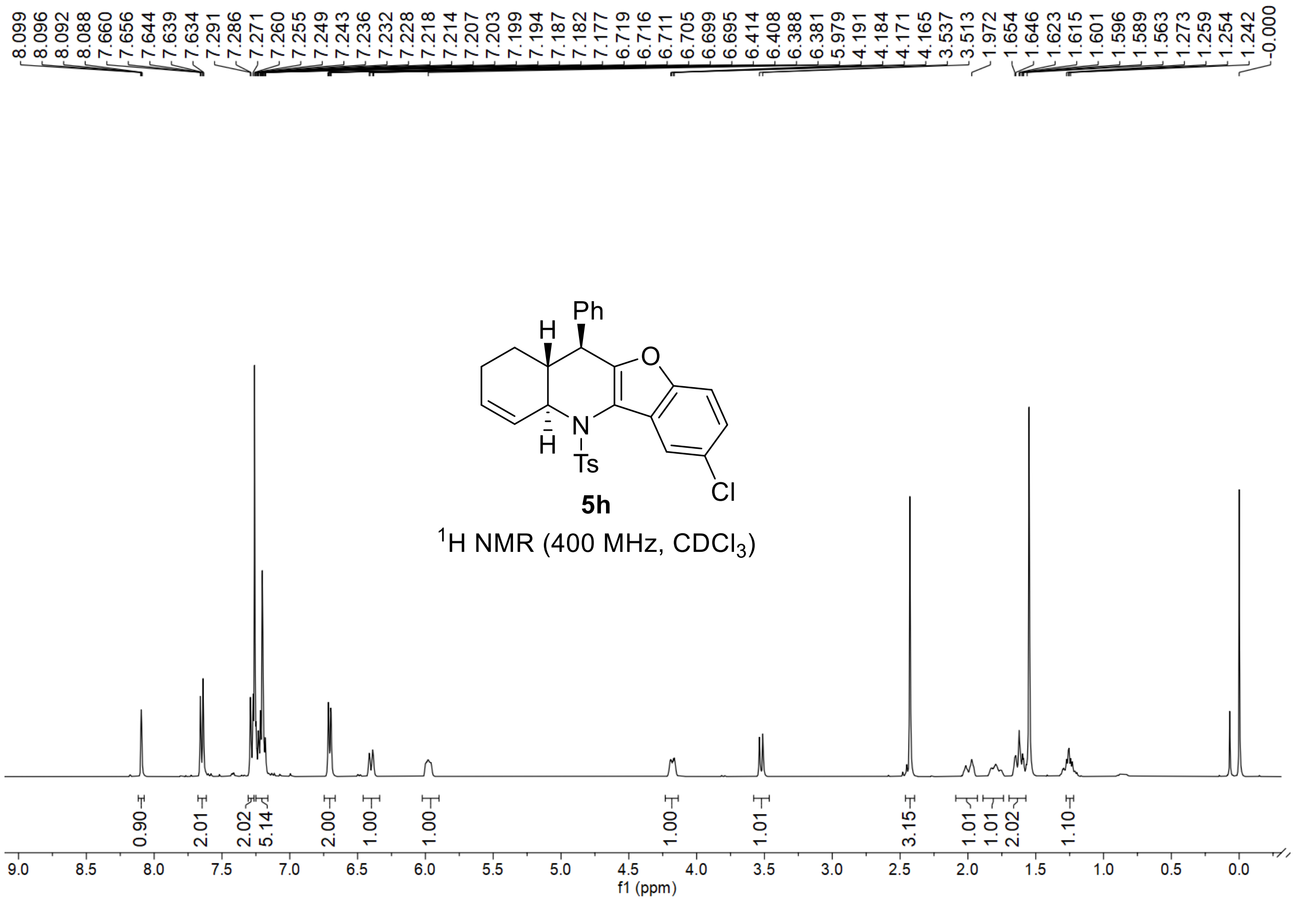
8.099 8.096 8.092 8.088 7.660 7.656 7.644 7.639 7.634 7.291 7.286 7.271 7.260 7.255 7.249 7.243 7.236 7.232 7.228 7.218 7.214 7.207 7.203 7.199 7.194 7.187 7.182 7.177 6.719 6.716 6.711 6.705 6.699 6.695 6.414 6.408 6.388 6.381 5.979 4.191 4.184 4.171 4.165 3.537 3.513 1.972 1.654 1.646 1.623 1.615 1.601 1.596 1.589 1.563 1.273 1.259 1.254 1.242 -0.000
Ph
H
O
N
H
Ts
Cl
5h
1H NMR (400 MHz, CDCl3)
0.90 2.01 2.02 5.14 2.00 1.00 1.00 1.00 1.01 3.15 1.01 1.01 2.02 1.10
9.0 8.5 8.0 7.5 7.0 6.5 6.0 5.5 5.0 4.5 4.0 3.5 3.0 2.5 2.0 1.5 1.0 0.5 0.0
f1 (ppm)

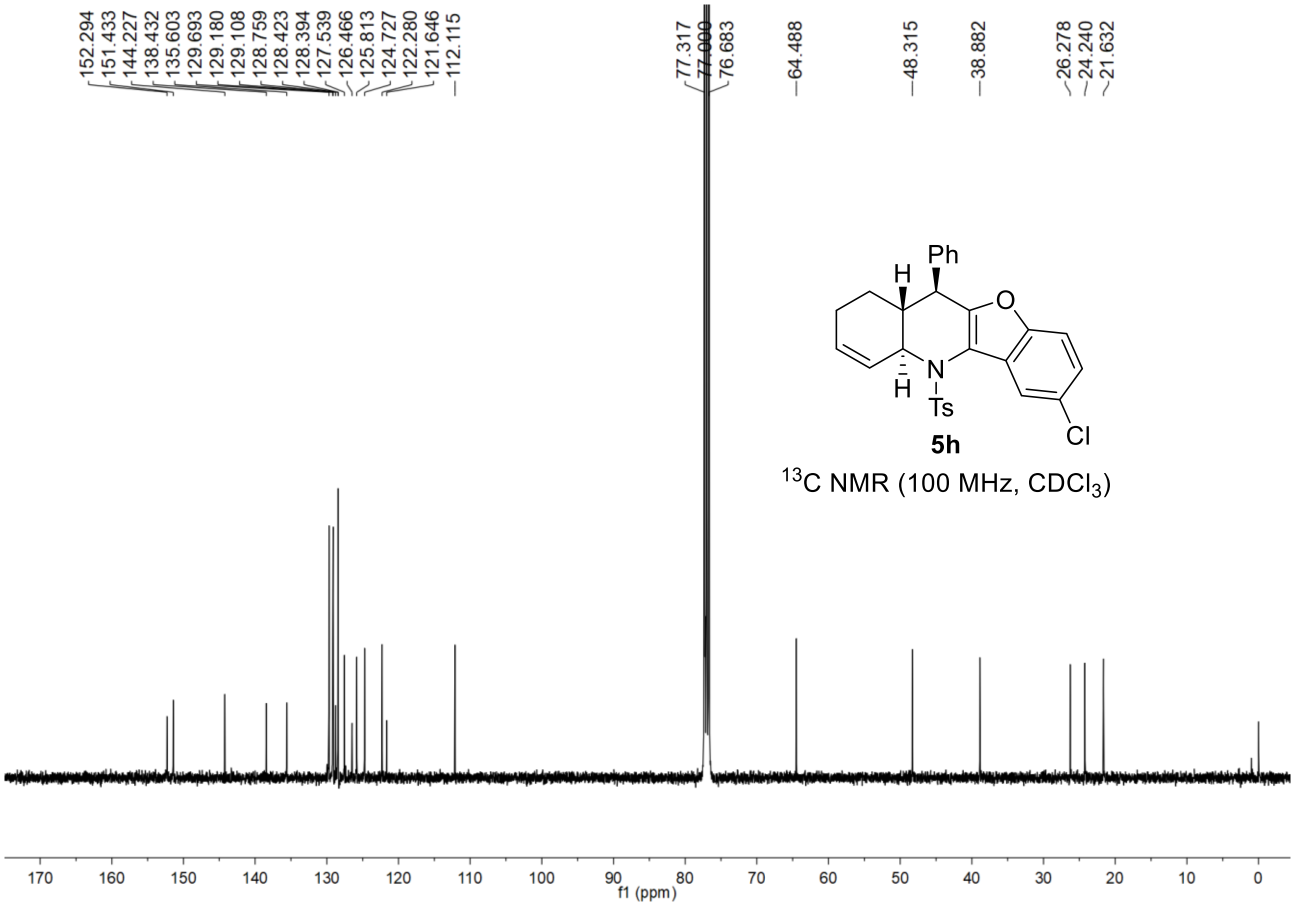
152.294 151.433 144.227 138.432 135.603 129.693 129.180 129.108 128.759 128.423 128.394 127.539 126.466 125.813 124.727 122.280 121.646 112.115
77.317 77.000 76.683
64.488
48.315
38.882
26.278 24.240 21.632
Ph
H
O
N
H
Ts
Cl
5h
13C NMR (100 MHz, CDCl3)
170 160 150 140 130 120 110 100 90 80 70 60 50 40 30 20 10 0
f1 (ppm)

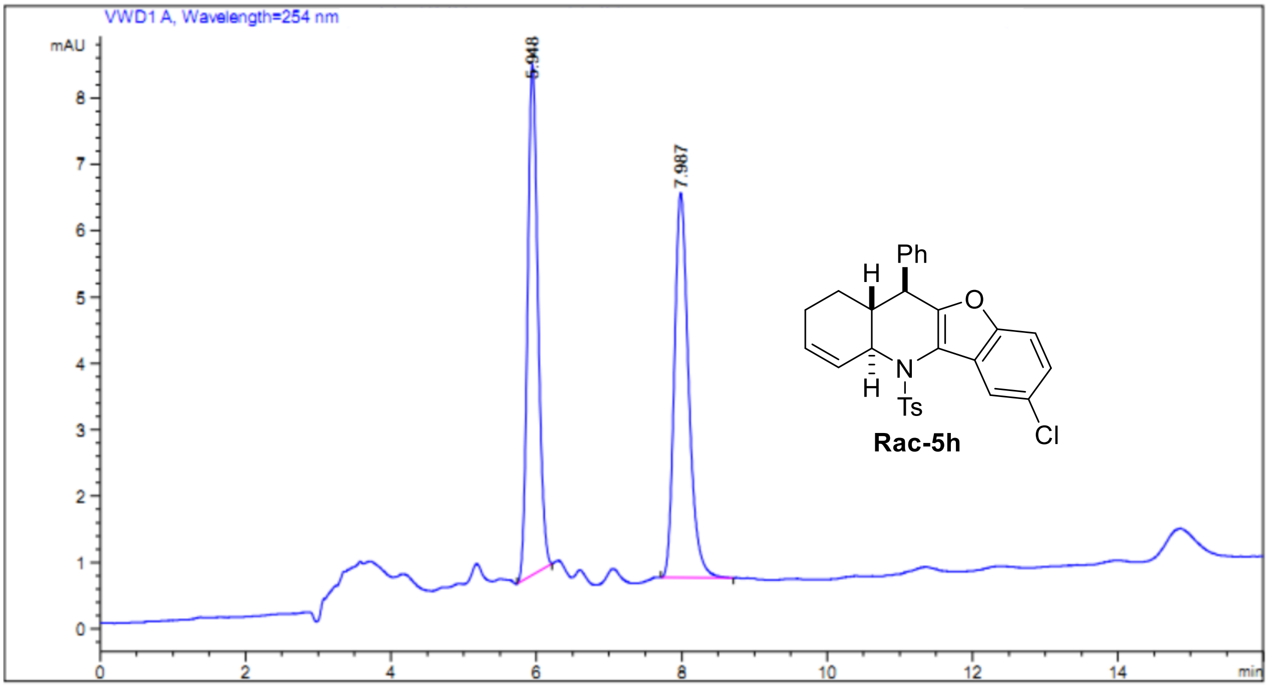

1	5.948	BB	0.1513	75.88798	7.70654	48.9486
2	7.987	BB	0.2071	79.14803	5.80458	51.0514

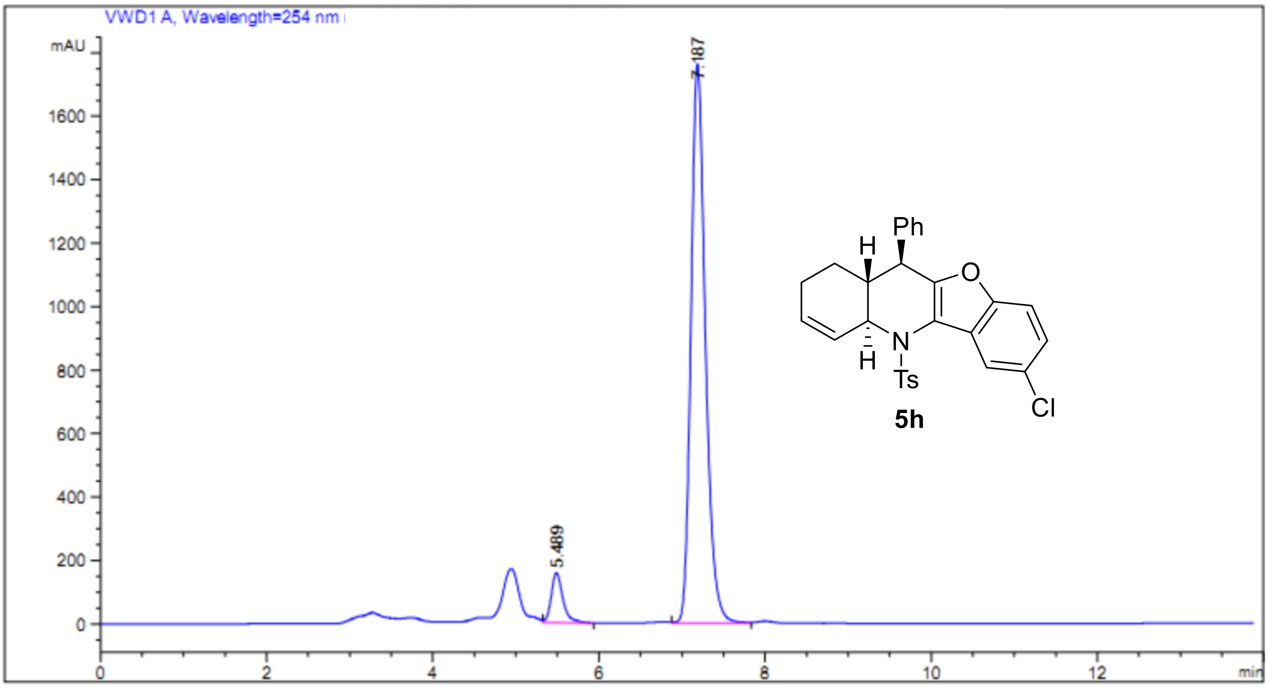

#	[min]		[min]	[mAU*s]	[mAU]	%
1	5.489	VB	0.1477	1536.48706	157.01198	6.7351
2	7.187	VV	0.1846	2.12767e4	1761.79443	93.2649

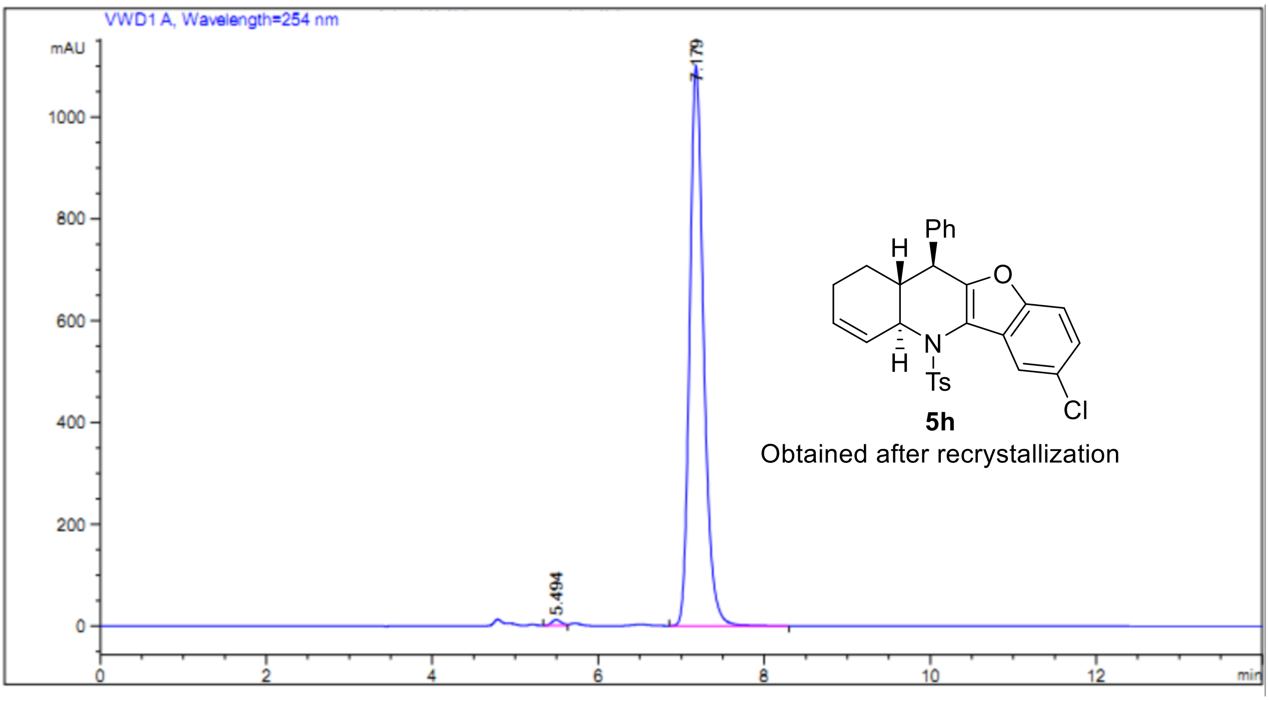

#	[min]		[min]	[mAU*s]	[mAU]	%
1	5.494	BV	0.1280	96.99508	11.61596	0.7467
2	7.179	VB	0.1790	1.28932e4	1100.01257	99.2533

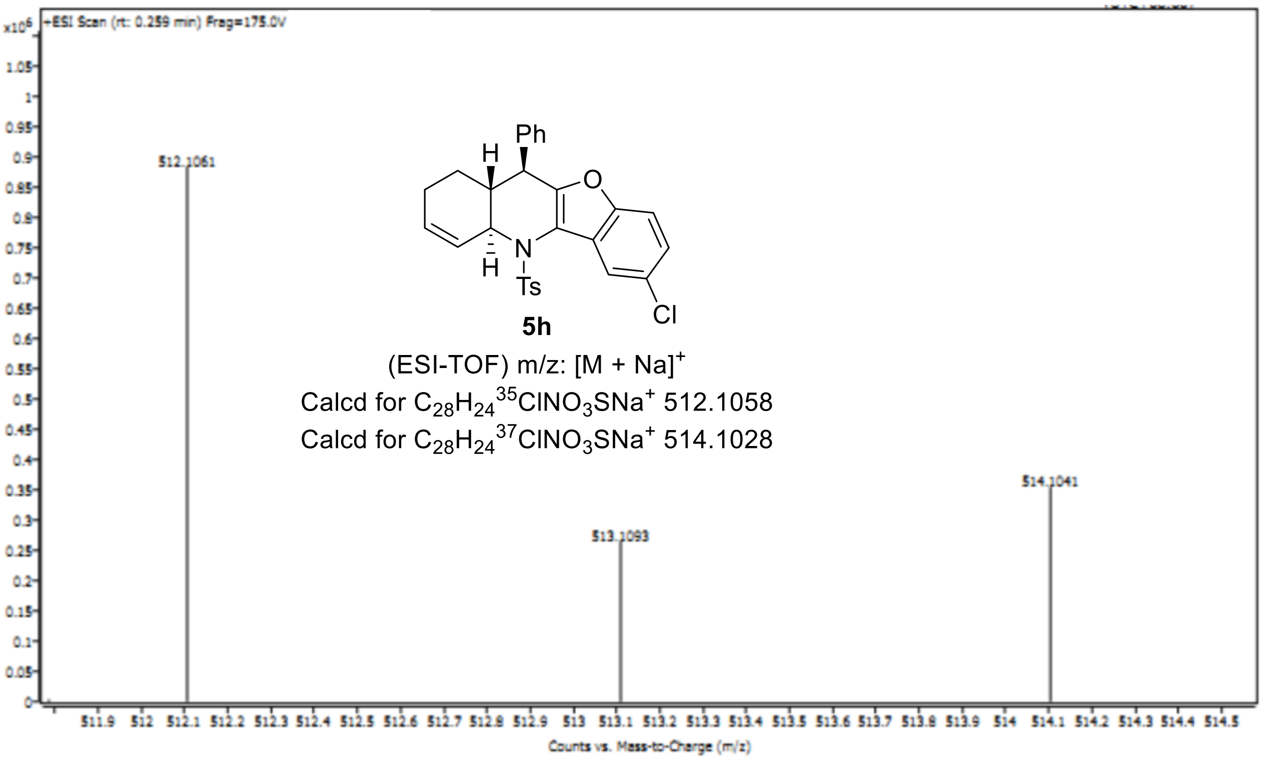

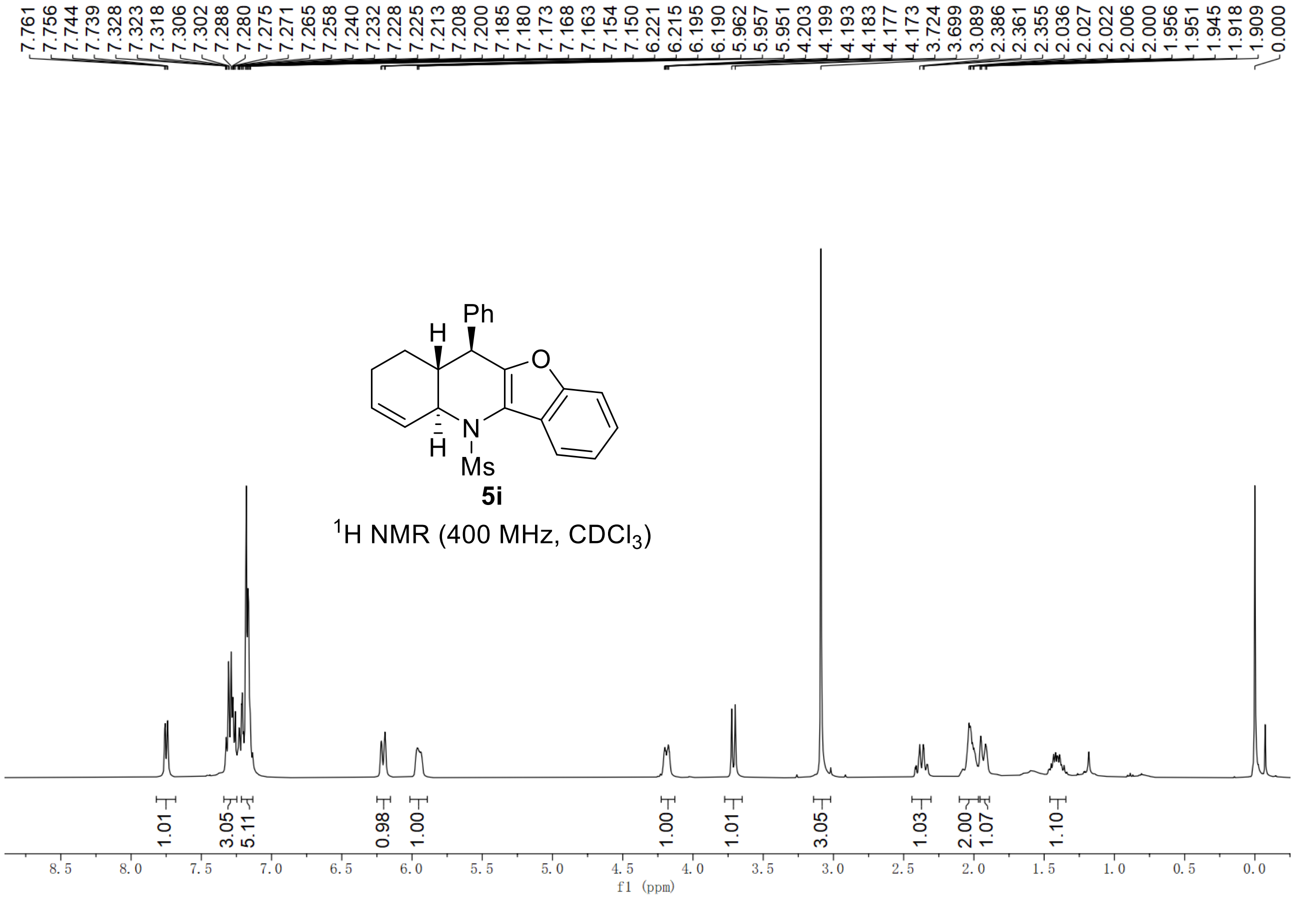
7.761
7.756
7.744
7.739
7.328
7.323
7.318
7.306
7.302
7.288
7.280
7.275
7.271
7.265
7.258
7.240
7.232
7.228
7.225
7.213
7.208
7.200
7.185
7.180
7.173
7.168
7.163
7.154
7.150
6.221
6.215
6.195
6.190
5.962
5.957
5.951
4.203
4.199
4.193
4.183
4.177
4.173
3.724
3.699
3.089
2.386
2.361
2.355
2.036
2.027
2.022
2.006
2.000
1.956
1.951
1.945
1.918
1.909
0.000
Ph
H
O
N
H
Ms
5i
^{1}H NMR (400 MHz, CDCl$_3$)
1.01
3.05
5.11
0.98
1.00
1.00
1.01
3.05
1.03
2.00
1.07
1.10
8.5
8.0
7.5
7.0
6.5
6.0
5.5
5.0
4.5
4.0
3.5
3.0
2.5
2.0
1.5
1.0
0.5
0.0
f1 (ppm)

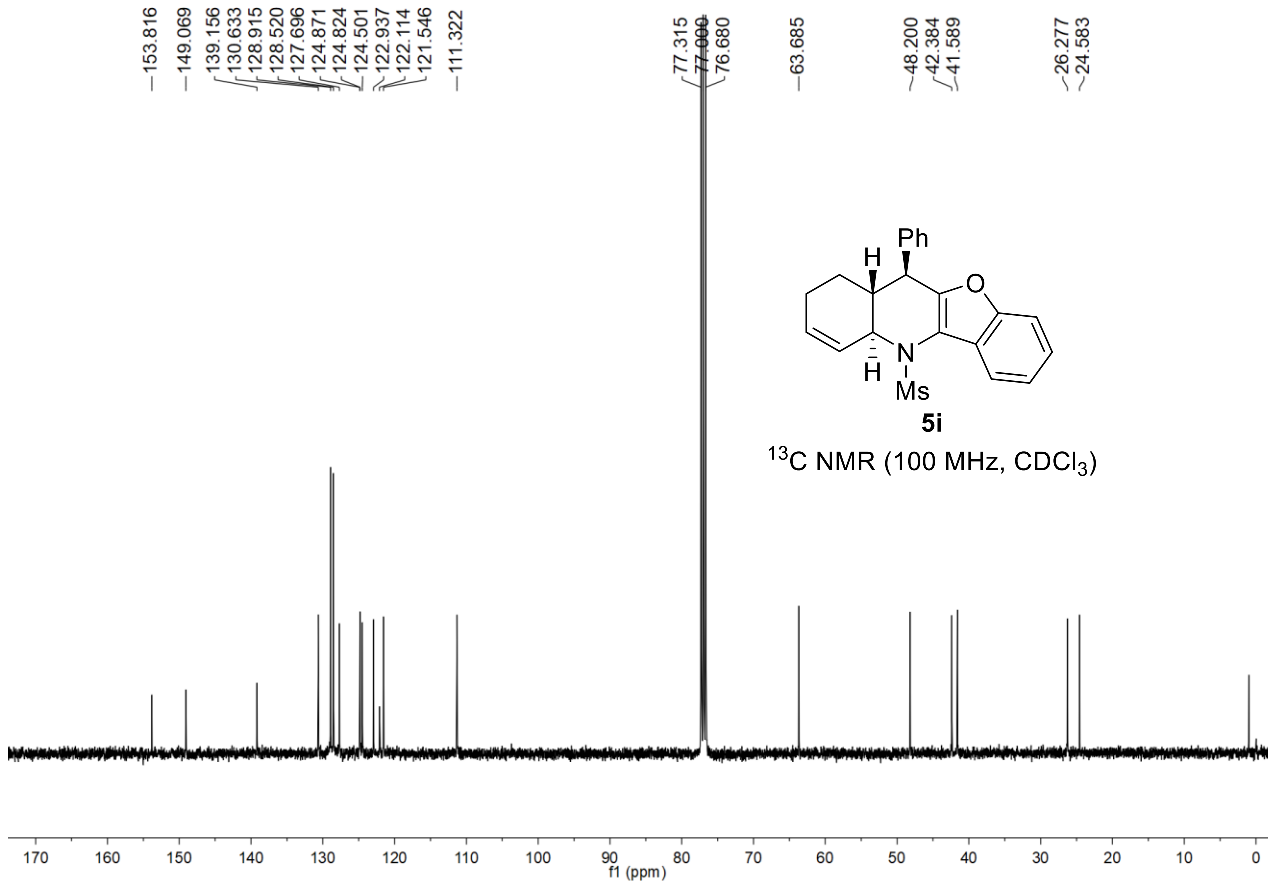
153.816
149.069
139.156
130.633
128.915
128.520
127.696
124.871
124.824
124.501
122.937
122.114
121.546
111.322
77.315
77.000
76.680
63.685
48.200
42.384
41.589
26.277
24.583
Ph
H
O
N
H
Ms
5i
^{13}C NMR (100 MHz, CDCl$_3$)
170
160
150
140
130
120
110
100
90
80
70
60
50
40
30
20
10
0
f1 (ppm)

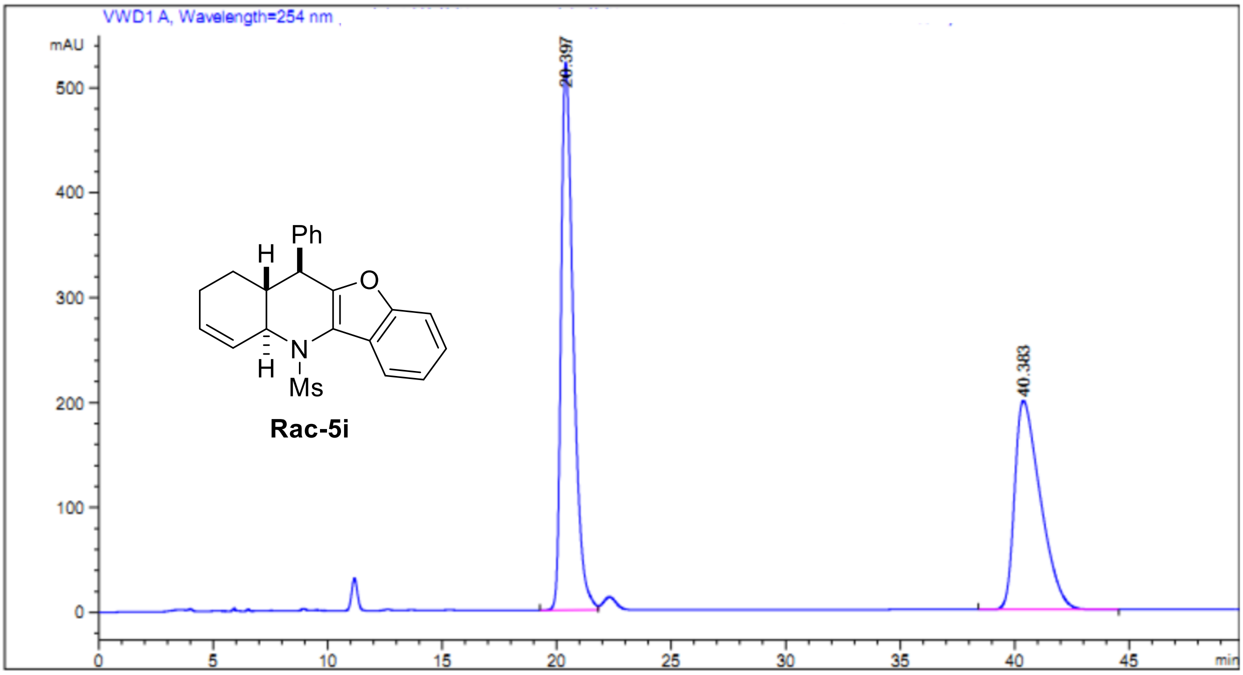

#	[min]		[min]	[mAU*s]	[mAU]	%
1	20.397	BV	0.5620	1.94044e4	521.82220	55.4913
2	40.383	BB	1.1802	1.55640e4	199.04520	44.5087

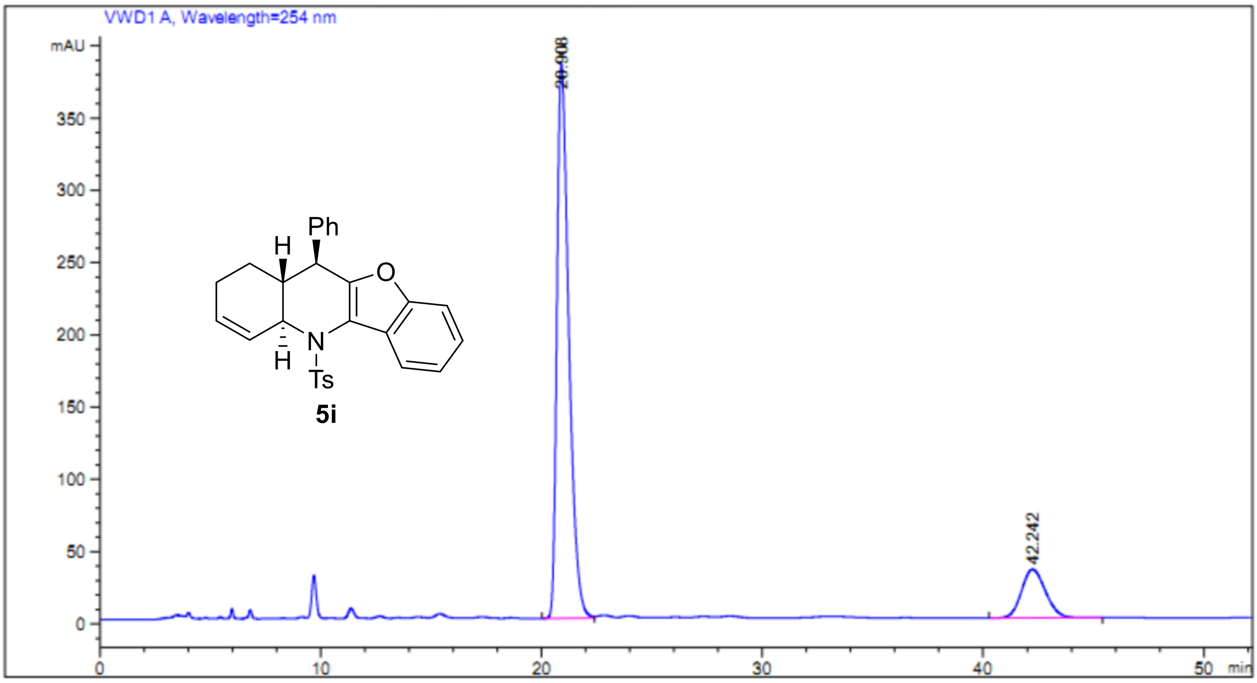

#	[min]		[min]	[mAU*s]	[mAU]	%
1	20.908	BV	0.5702	1.43412e4	383.52646	84.9784
2	42.242	BB	1.1768	2535.08228	33.61837	15.0216

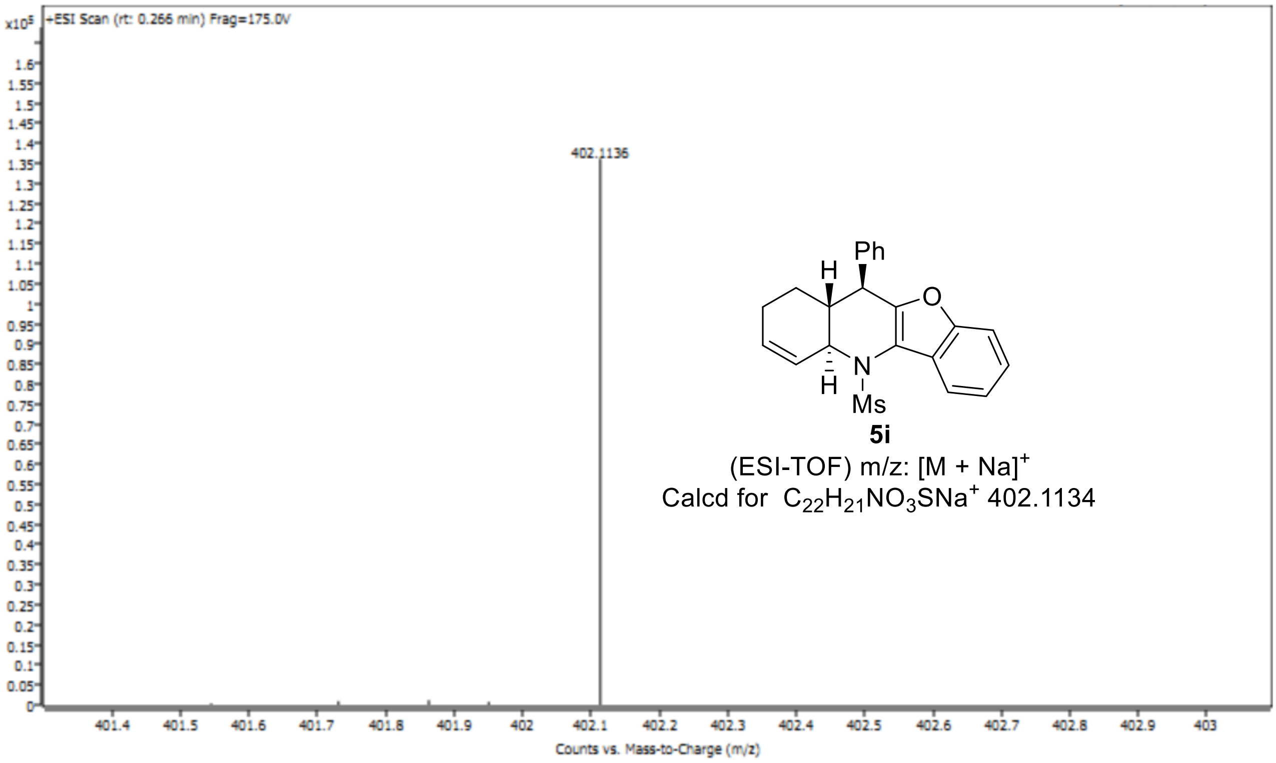
+ESI Scan (rt: 0.266 min) Frag=175.0V
x10^5
402.1136
Ph
H
O
N
H
Ms
5i
(ESI-TOF) m/z: [M + Na]+
Calcd for C22H21NO3SNa+ 402.1134
Counts vs. Mass-to-Charge (m/z)

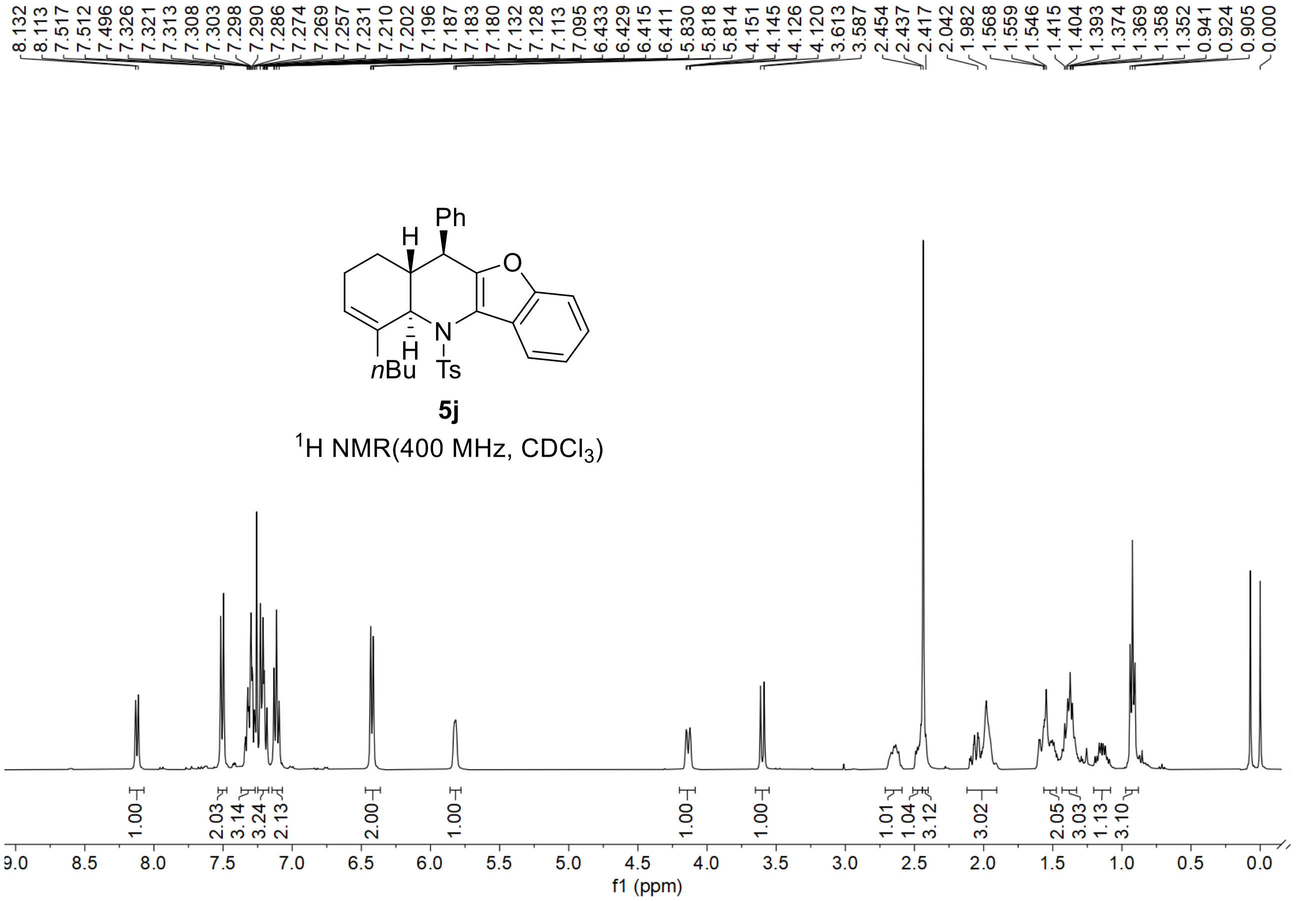
8.132 8.113 7.517 7.512 7.496 7.326 7.321 7.313 7.308 7.303 7.298 7.290 7.286 7.274 7.269 7.257 7.231 7.210 7.202 7.196 7.187 7.183 7.180 7.132 7.128 7.113 7.095 6.433 6.429 6.415 6.411 5.830 5.818 5.814 4.151 4.145 4.126 4.120 3.613 3.587 2.454 2.437 2.417 2.042 1.982 1.568 1.559 1.546 1.415 1.404 1.393 1.374 1.369 1.358 1.352 0.941 0.924 0.905 0.000
Ph
H
O
N
H
nBu
Ts
5j
1H NMR(400 MHz, CDCl3)
1.00 2.03 3.14 3.24 2.13 2.00 1.00 1.00 1.00 1.01 1.04 3.12 3.02 2.05 3.03 1.13 3.10
9.0 8.5 8.0 7.5 7.0 6.5 6.0 5.5 5.0 4.5 4.0 3.5 3.0 2.5 2.0 1.5 1.0 0.5 0.0
f1 (ppm)

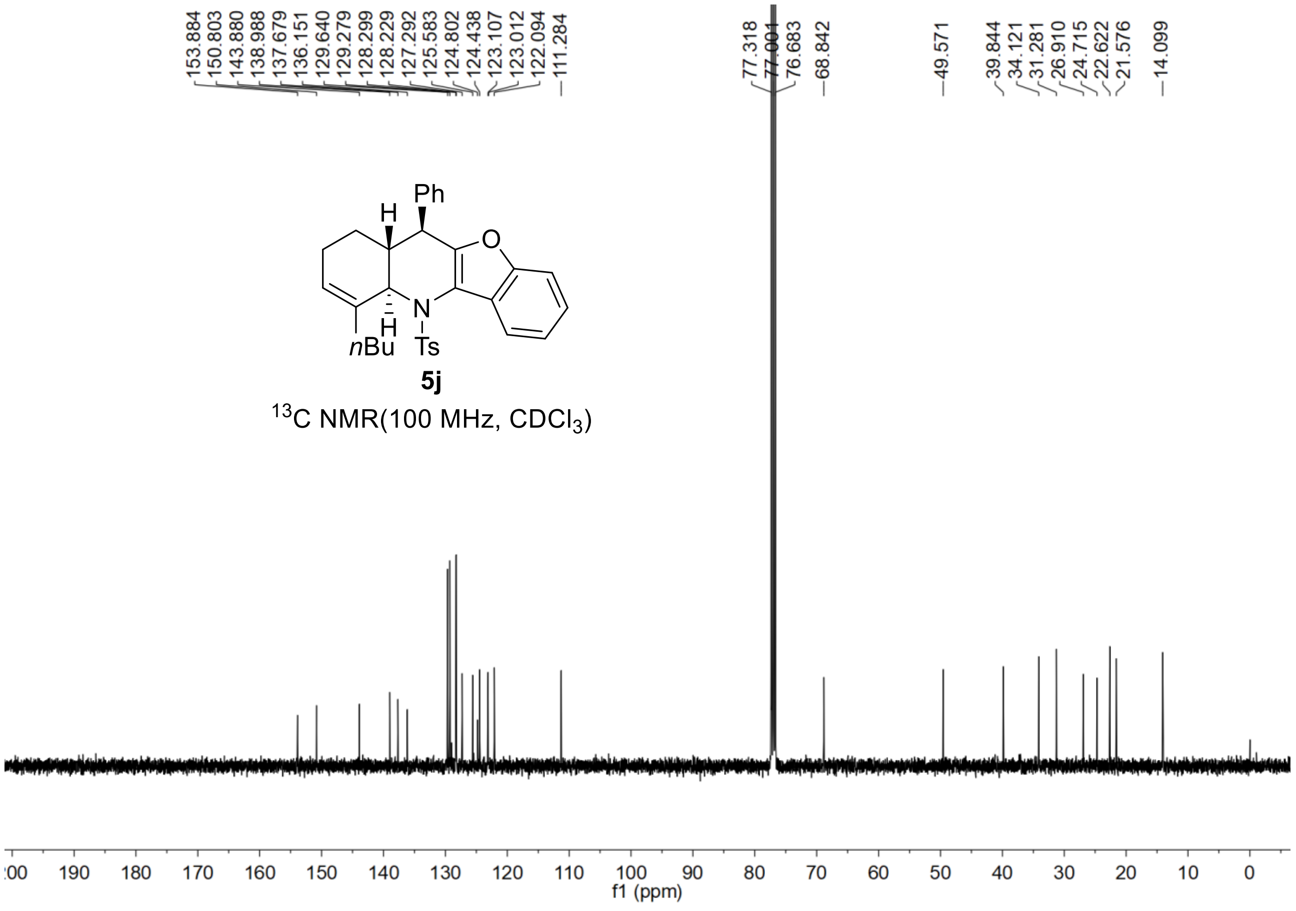
153.884 150.803 143.880 138.988 137.679 136.151 129.640 129.279 128.299 128.229 127.292 125.583 124.802 124.438 123.107 123.012 122.094 111.284
77.318 77.001 76.683 68.842
49.571
39.844 34.121 31.281 26.910 24.715 22.622 21.576
14.099
Ph
H
O
N
H
nBu
Ts
5j
13C NMR(100 MHz, CDCl3)
200 190 180 170 160 150 140 130 120 110 100 90 80 70 60 50 40 30 20 10 0
f1 (ppm)

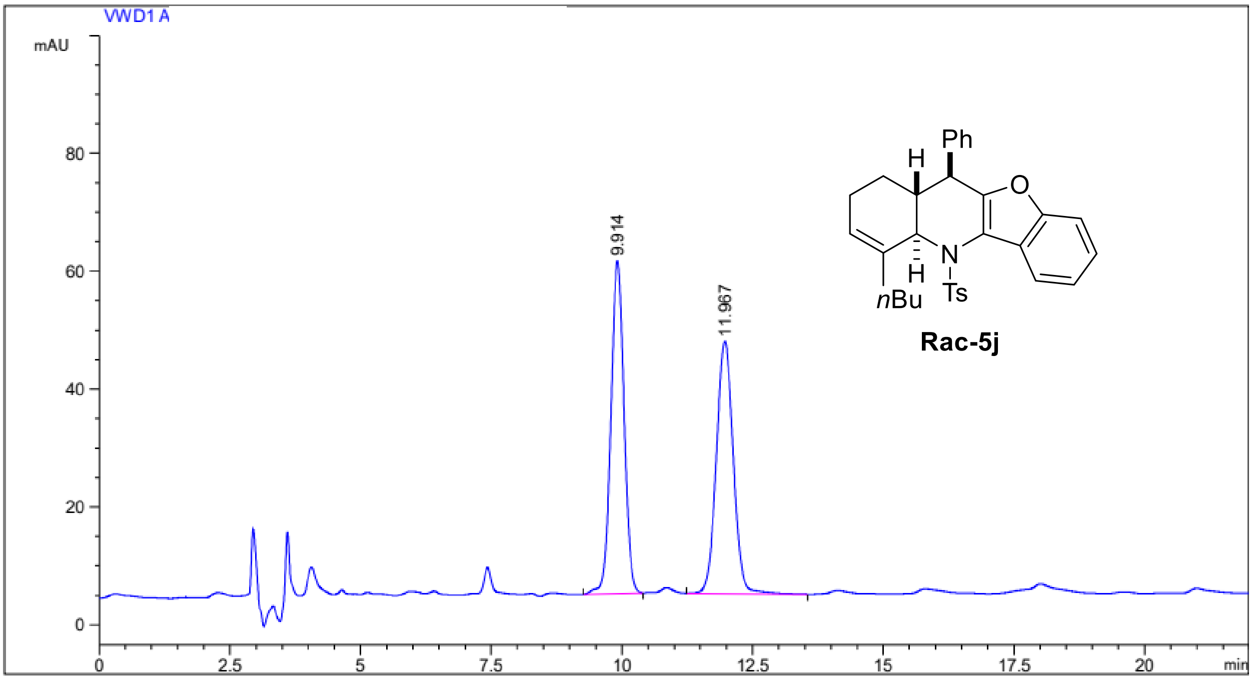

#	[min]		[min]	mAU *s	[mAU]	%
1	9.914	BB	0.2718	991.04480	56.45636	50.5513
2	11.967	BB	0.3516	969.42804	42.80004	49.4487

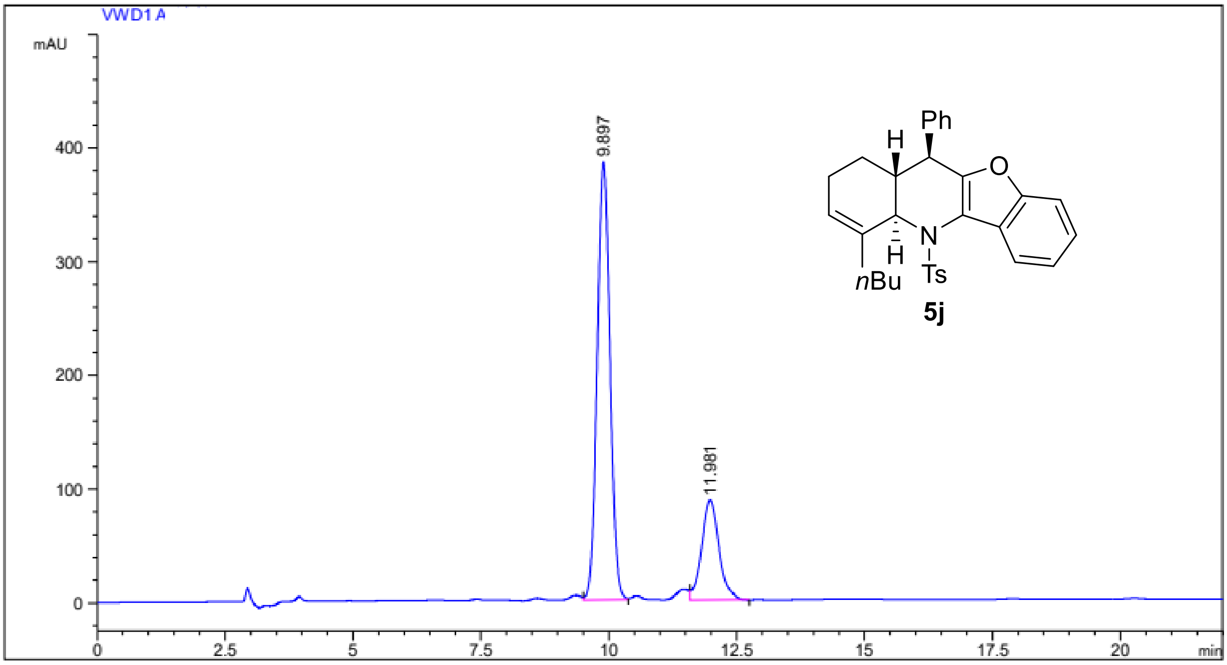

#	[min]		[min]	mAU *s	[mAU]	%
1	9.897	VV	0.2713	6697.98730	385.17892	76.3581
2	11.981	VB	0.3631	2073.82251	87.74405	23.6419

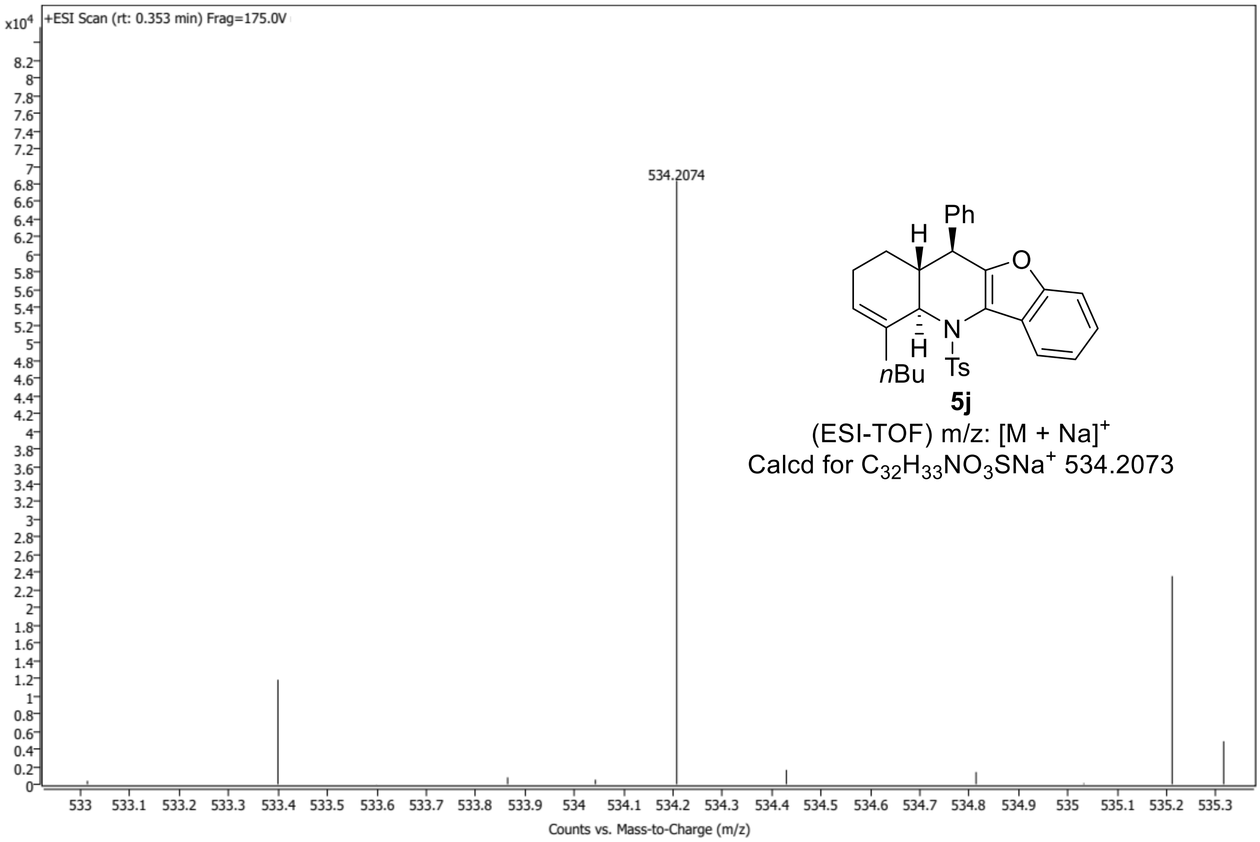
+ESI Scan (rt: 0.353 min) Frag=175.0V
x10^4
534.2074
Ph
H
O
H
N
nBu
Ts
5j
(ESI-TOF) m/z: [M + Na]+
Calcd for C32H33NO3SNa+ 534.2073
Counts vs. Mass-to-Charge (m/z)

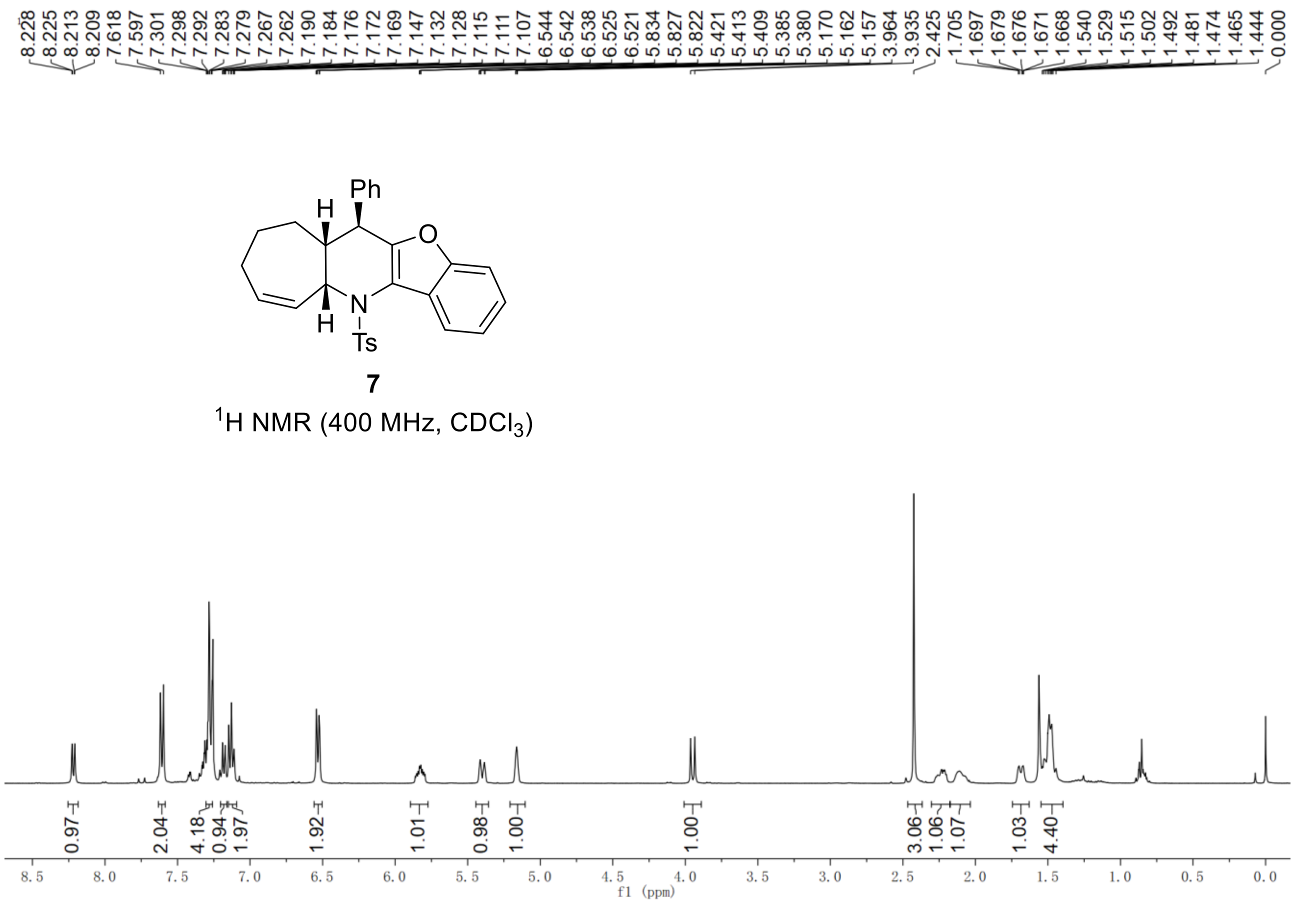
8.228
8.225
8.213
8.209
7.618
7.597
7.301
7.298
7.292
7.283
7.279
7.267
7.262
7.190
7.184
7.176
7.172
7.169
7.147
7.132
7.128
7.115
7.111
7.107
6.544
6.542
6.538
6.525
6.521
5.834
5.827
5.822
5.421
5.413
5.409
5.385
5.380
5.170
5.162
5.157
3.964
3.935
2.425
1.705
1.697
1.679
1.676
1.671
1.668
1.540
1.529
1.515
1.502
1.492
1.481
1.474
1.465
1.444
0.000
Ph
H
O
N
H
Ts
7
^{1}H NMR (400 MHz, CDCl$_3$)
0.97
2.04
4.18
0.94
1.97
1.92
1.01
0.98
1.00
1.00
3.06
1.06
1.07
1.03
4.40
f1 (ppm)

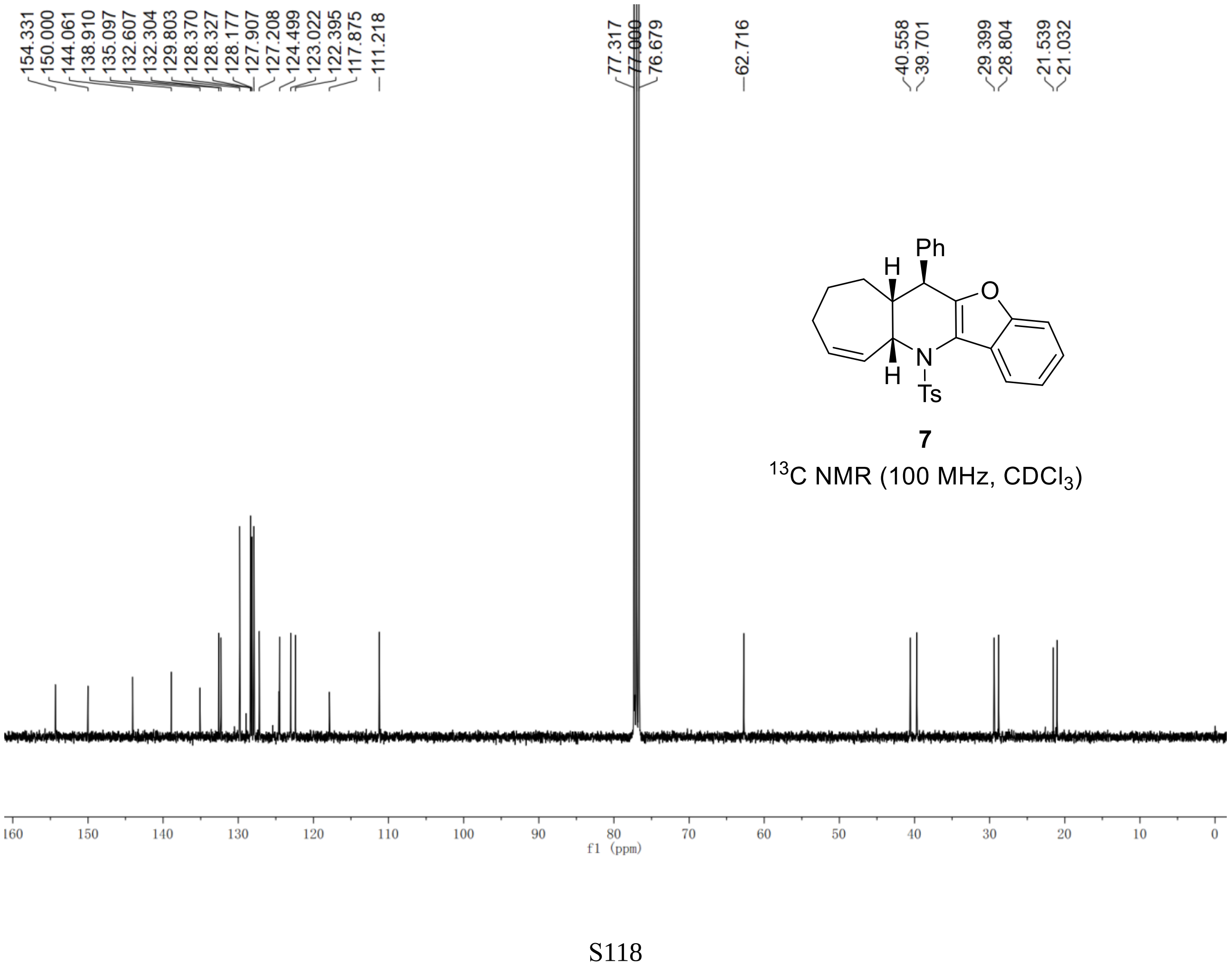
154.331
150.000
144.061
138.910
135.097
132.607
132.304
129.803
128.370
128.327
128.177
127.907
127.208
124.499
123.022
122.395
117.875
111.218
77.317
77.000
76.679
62.716
40.558
39.701
29.399
28.804
21.539
21.032
Ph
H
O
N
H
Ts
7
^{13}C NMR (100 MHz, CDCl$_3$)
f1 (ppm)

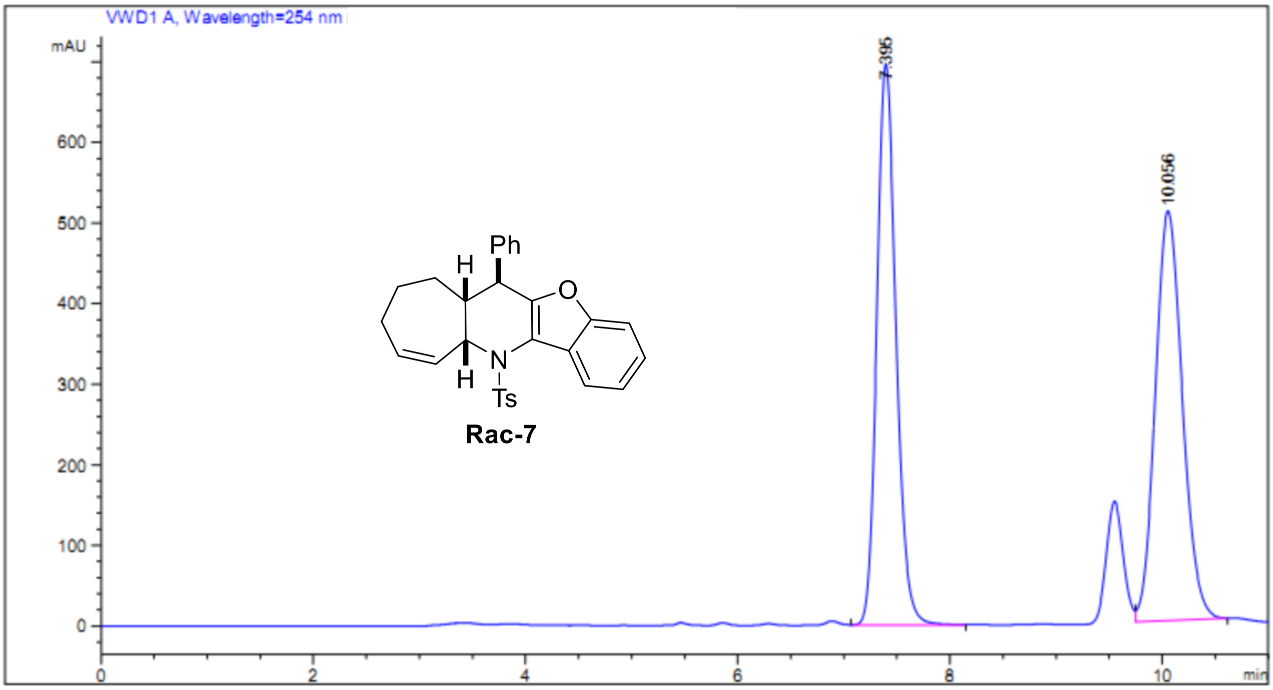

#	[min]		[min]	[mAU*s]	[mAU]	%
1	7.395	BBA	0.1926	8705.24414	695.48901	50.1577
2	10.056	BB	0.2706	8650.50391	499.21210	49.8423

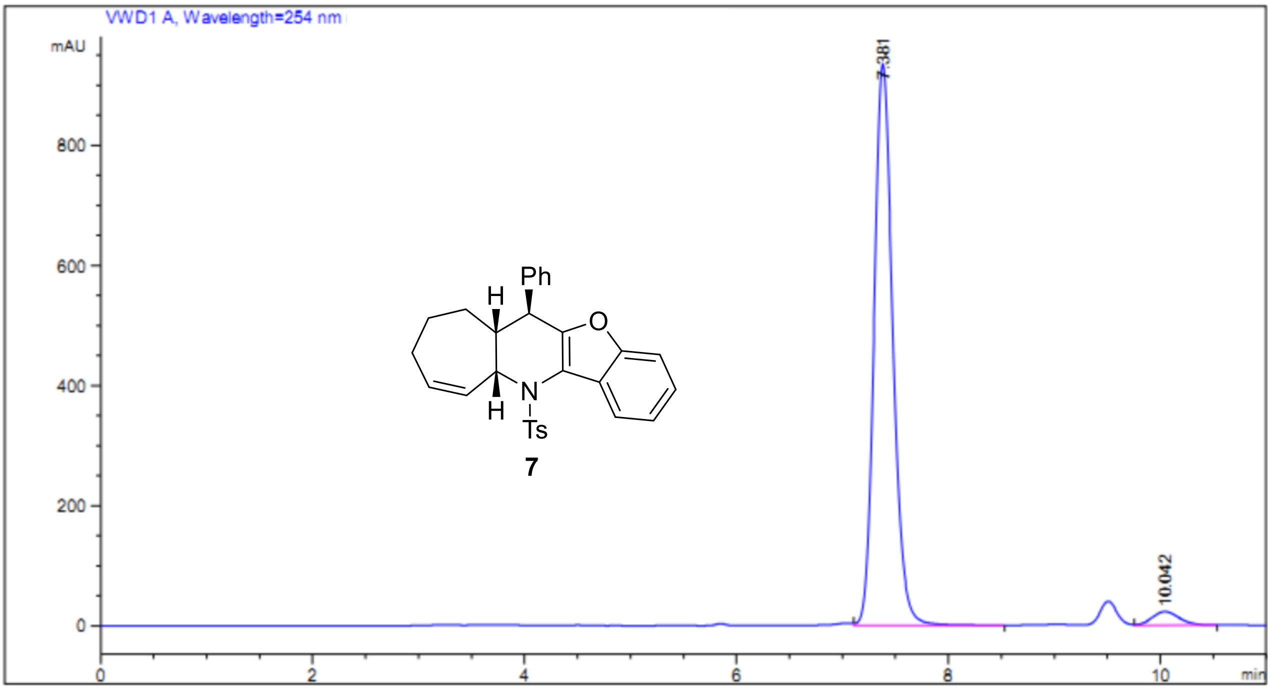

#	[min]		[min]	[mAU*s]	[mAU]	%
1	7.381	BBA	0.1884	1.13252e4	931.93732	96.7588
2	10.042	BB	0.2685	379.36380	22.12307	3.2412

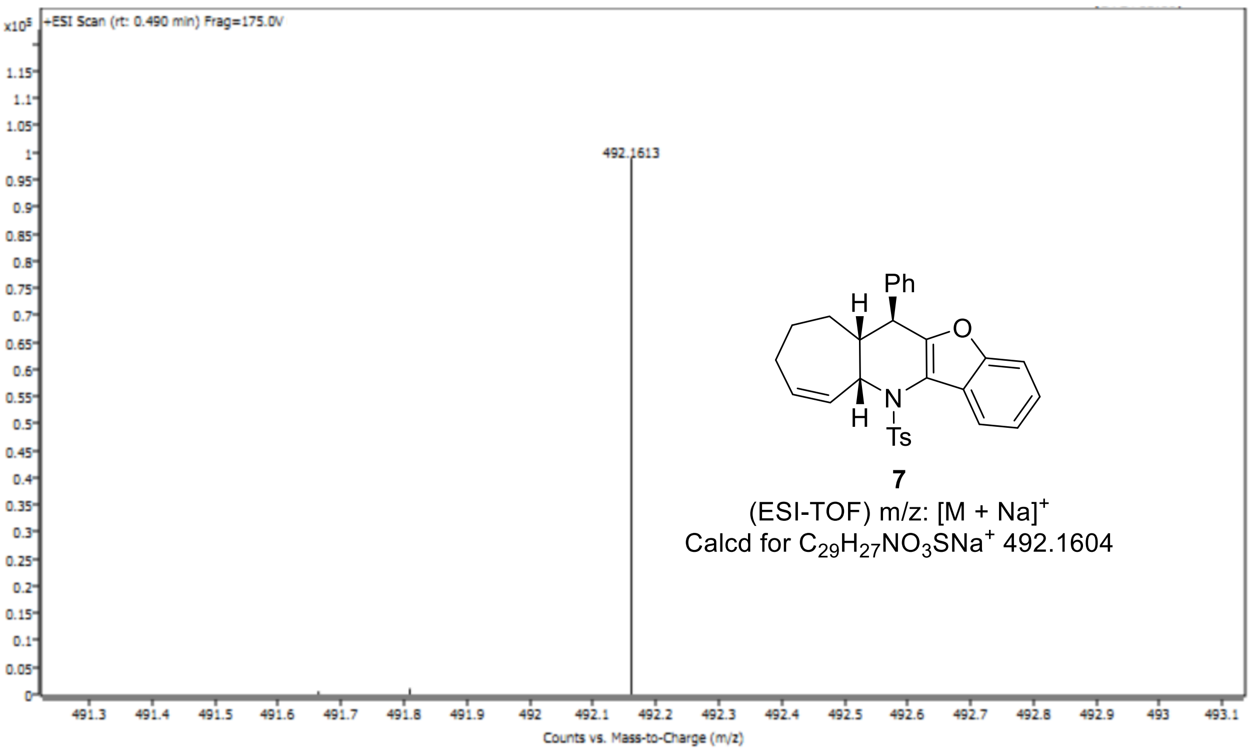
x10^5
+ESI Scan (rt: 0.490 min) Frag=175.0V
492.1613
Ph
H
O
N
H
Ts
7
(ESI-TOF) m/z: [M + Na]+
Calcd for C29H27NO3SNa+ 492.1604
Counts vs. Mass-to-Charge (m/z)

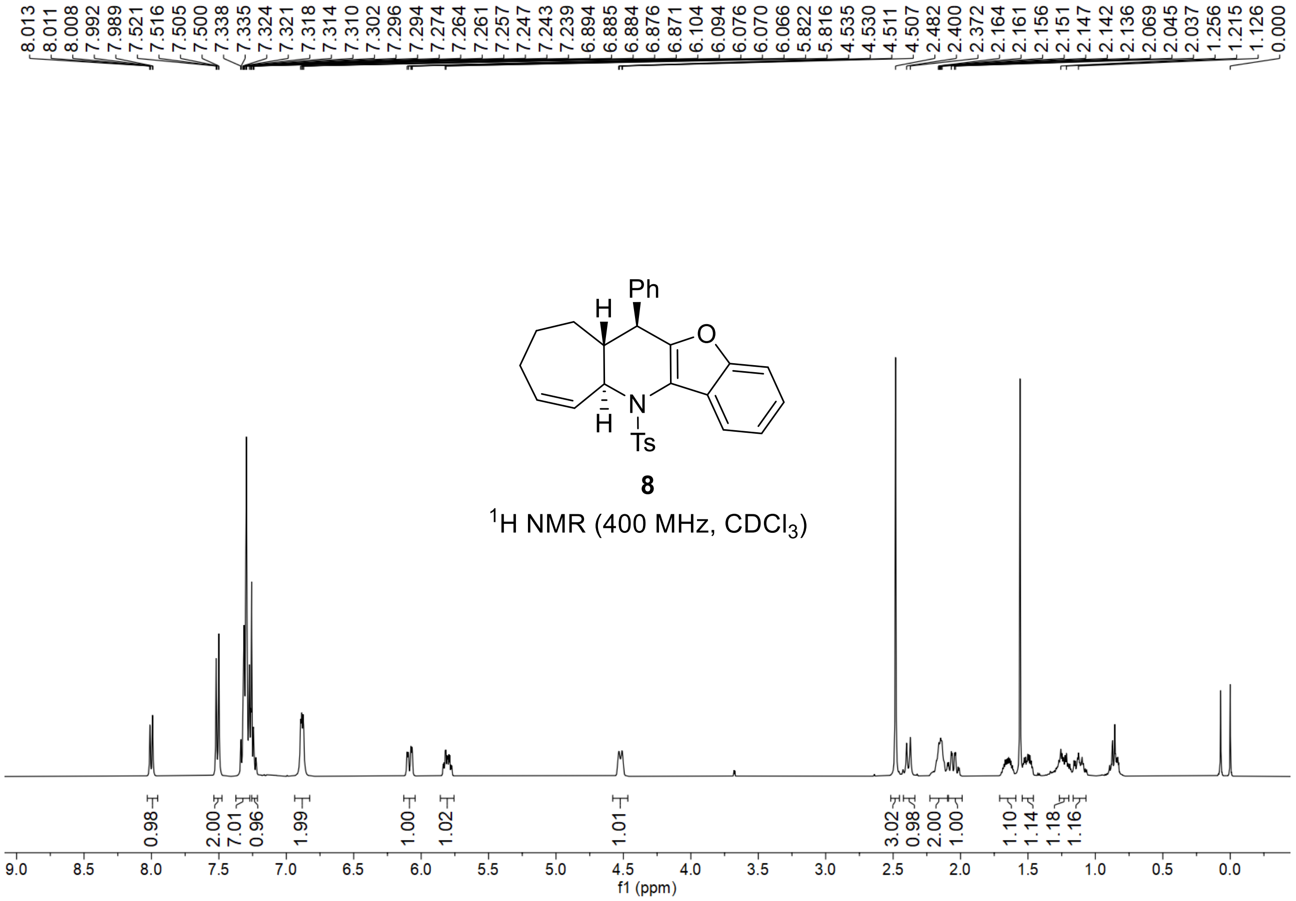

8.013 8.011 8.008 7.992 7.989 7.521 7.516 7.505 7.500 7.338 7.335 7.324 7.321 7.318 7.314 7.310 7.302 7.296 7.294 7.274 7.264 7.261 7.257 7.247 7.243 7.239 6.894 6.885 6.884 6.876 6.871 6.104 6.094 6.076 6.070 6.066 5.822 5.816 4.535 4.530 4.511 4.507 2.482 2.400 2.372 2.164 2.161 2.156 2.151 2.147 2.142 2.136 2.069 2.045 2.037 1.256 1.215 1.126 0.000
Ph
H
O
N
H
Ts
8
1H NMR (400 MHz, CDCl3)
0.98 2.00 7.01 0.96 1.99 1.00 1.02 1.01 3.02 0.98 2.00 1.00 1.10 1.14 1.18 1.16
9.0 8.5 8.0 7.5 7.0 6.5 6.0 5.5 5.0 4.5 4.0 3.5 3.0 2.5 2.0 1.5 1.0 0.5 0.0
f1 (ppm)

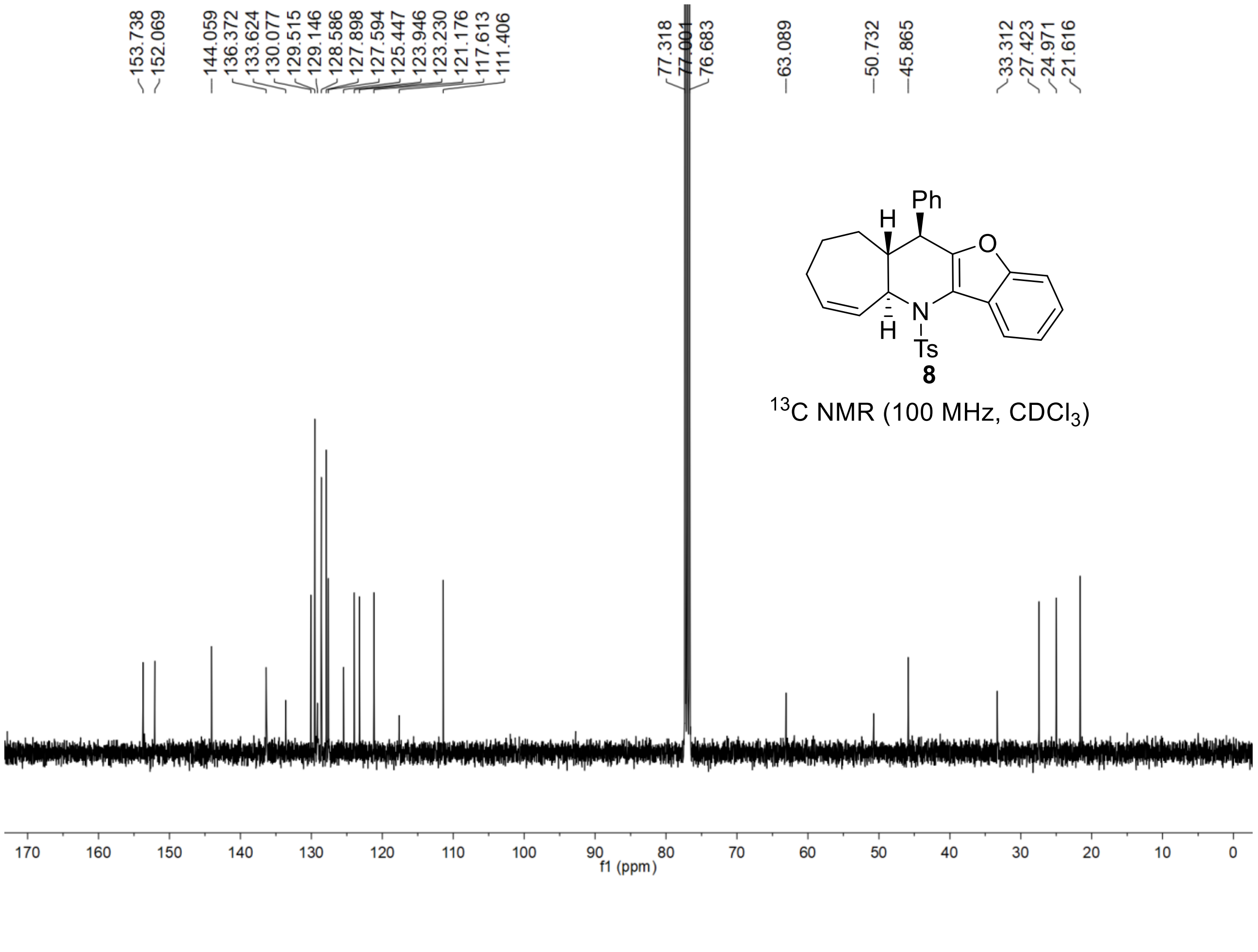

153.738 152.069 144.059 136.372 133.624 130.077 129.515 129.146 128.586 127.898 127.594 125.447 123.946 123.230 121.176 117.613 111.406 77.318 77.001 76.683 63.089 50.732 45.865 33.312 27.423 24.971 21.616
Ph
H
O
N
H
Ts
8
13C NMR (100 MHz, CDCl3)
170 160 150 140 130 120 110 100 90 80 70 60 50 40 30 20 10 0
f1 (ppm)

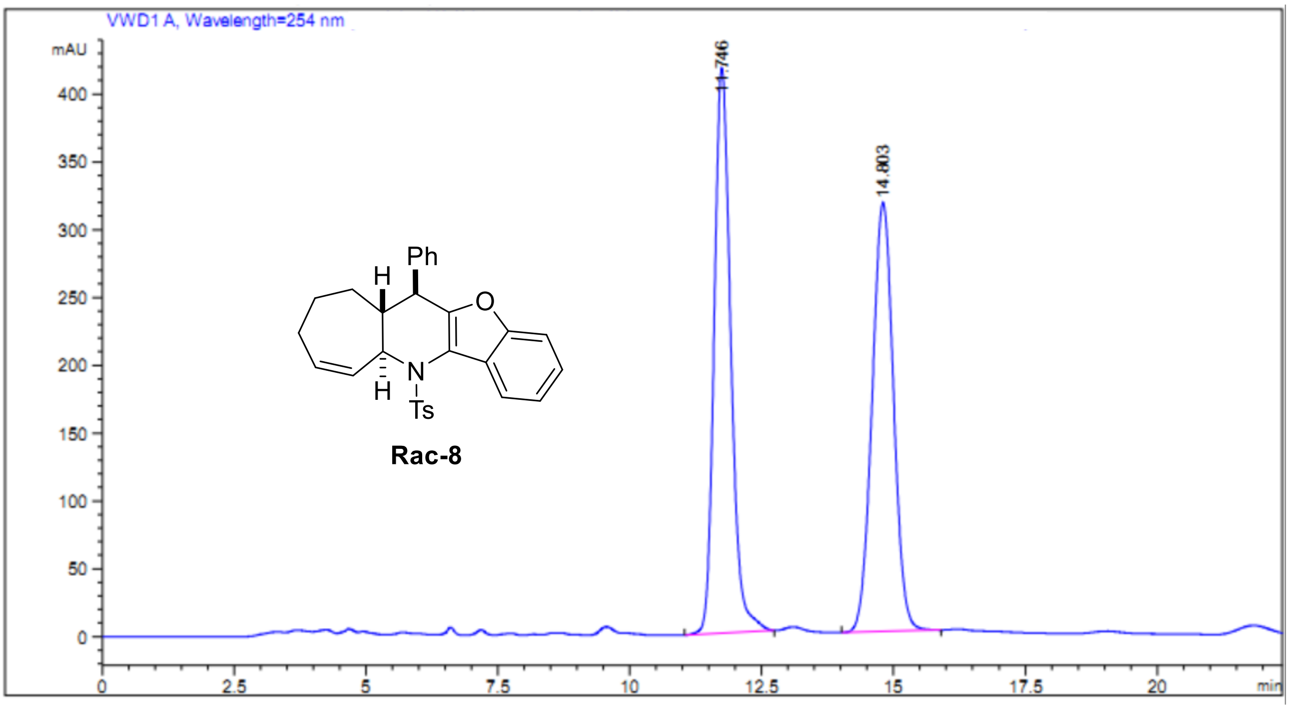

#	[min]		[min]	[mAU*s]	[mAU]	%
1	11.746	BBA	0.3386	9129.81934	416.64188	50.6234
2	14.803	BB	0.4393	8904.95898	316.14224	49.3766

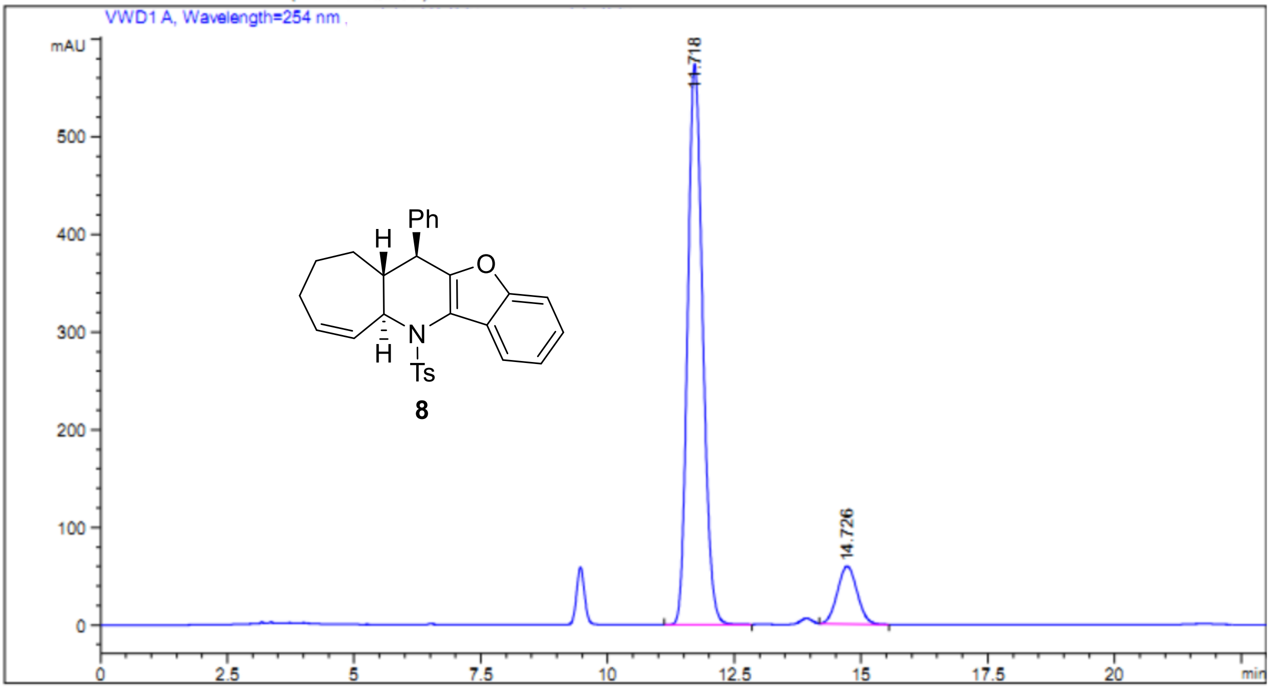

#	[min]		[min]	[mAU*s]	[mAU]	%
1	11.718	BBA	0.3223	1.18433e4	573.84747	88.1813
2	14.726	BBA	0.4212	1587.31763	59.08039	11.8187

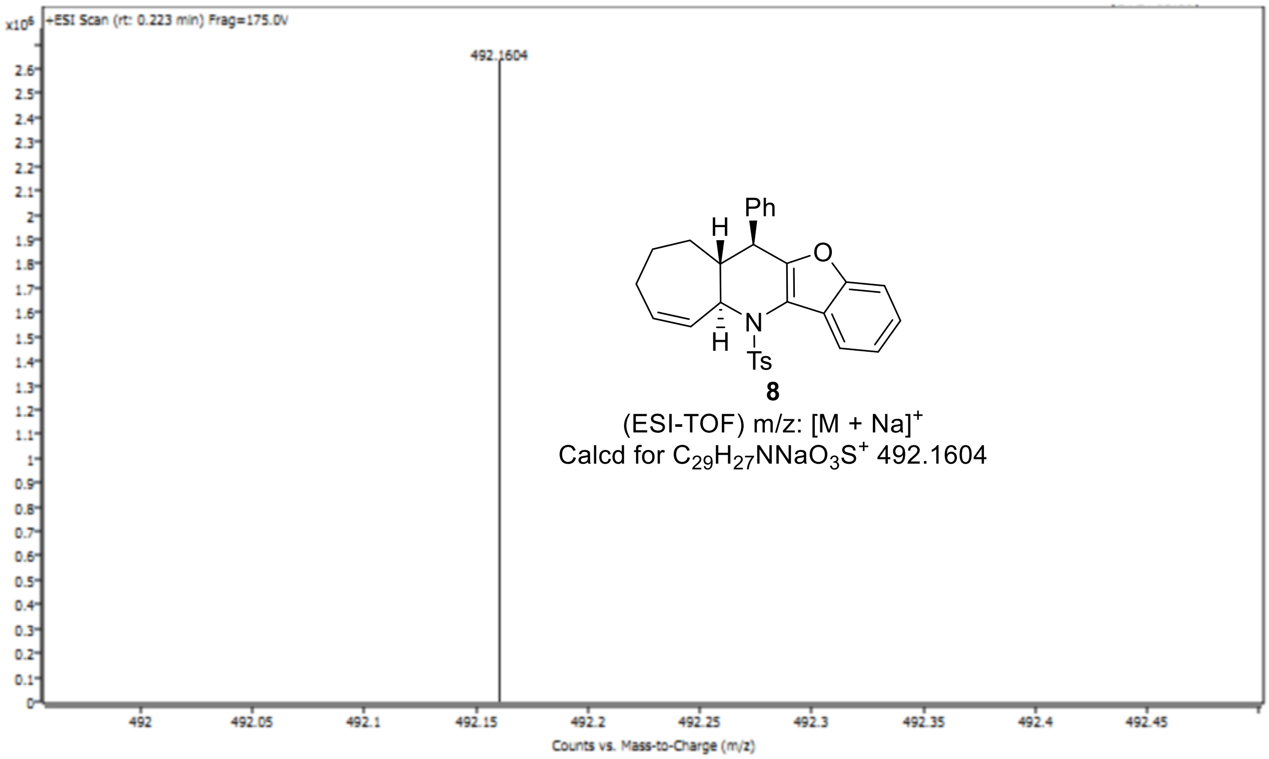

+ESI Scan (rt: 0.223 min) Frag=175.0V
x10^6
492.1604
Ph
H
O
N
H
Ts
8
(ESI-TOF) m/z: [M + Na]+
Calcd for C29H27NNaO3S+ 492.1604
Counts vs. Mass-to-Charge (m/z)

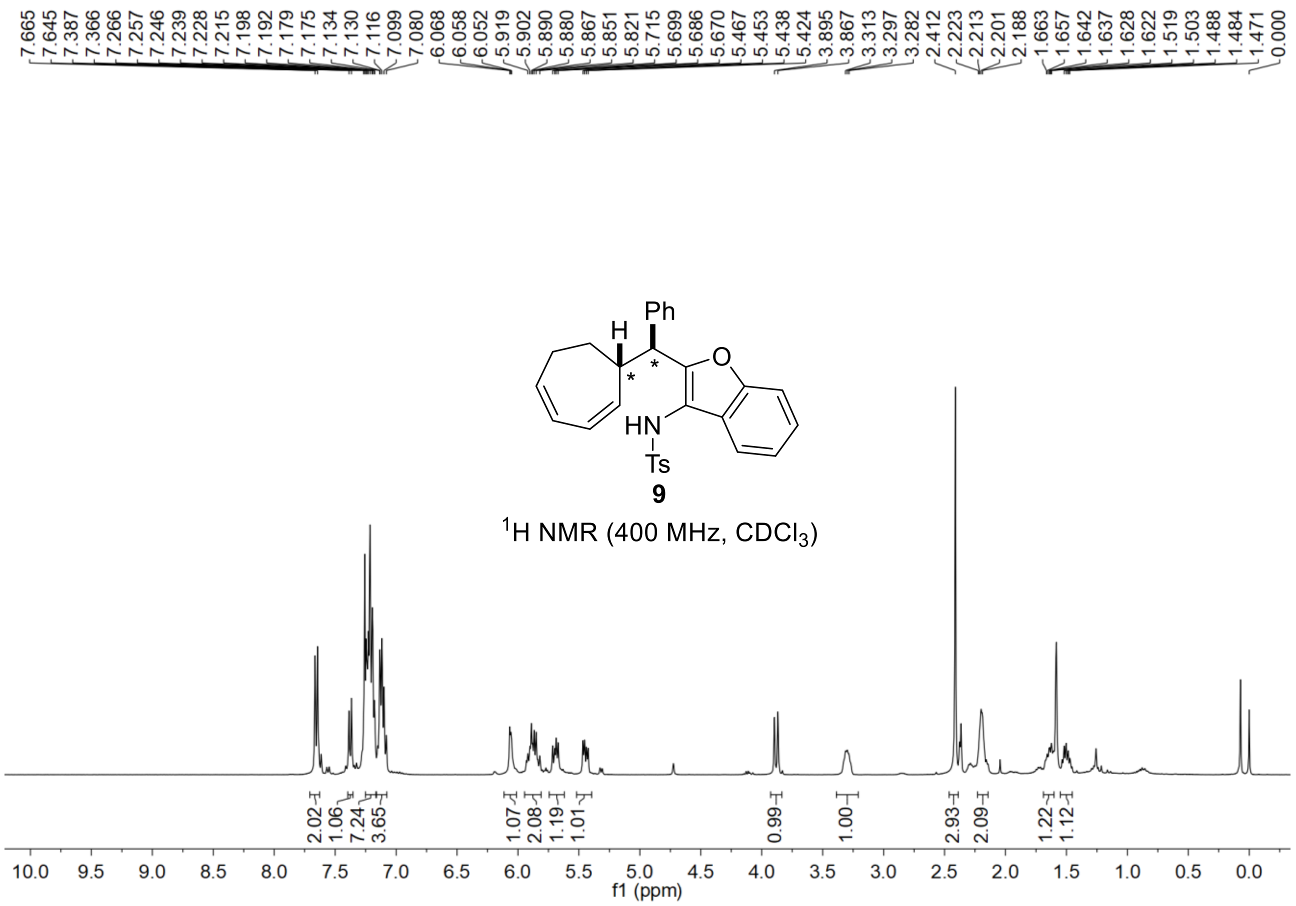
7.665 7.645 7.387 7.366 7.266 7.257 7.246 7.239 7.228 7.215 7.198 7.192 7.179 7.175 7.134 7.130 7.116 7.099 7.080 6.068 6.058 6.052 5.919 5.902 5.890 5.880 5.867 5.851 5.821 5.715 5.699 5.686 5.670 5.467 5.453 5.438 5.424 3.895 3.867 3.313 3.297 3.282 2.412 2.223 2.213 2.201 2.188 1.663 1.657 1.642 1.637 1.628 1.622 1.519 1.503 1.488 1.484 1.471 0.000
Ph
H
O
HN
Ts
9
1H NMR (400 MHz, CDCl3)
2.02 1.06 7.24 3.65 1.07 2.08 1.19 1.01 0.99 1.00 2.93 2.09 1.22 1.12
10.0 9.5 9.0 8.5 8.0 7.5 7.0 6.5 6.0 5.5 5.0 4.5 4.0 3.5 3.0 2.5 2.0 1.5 1.0 0.5 0.0
f1 (ppm)

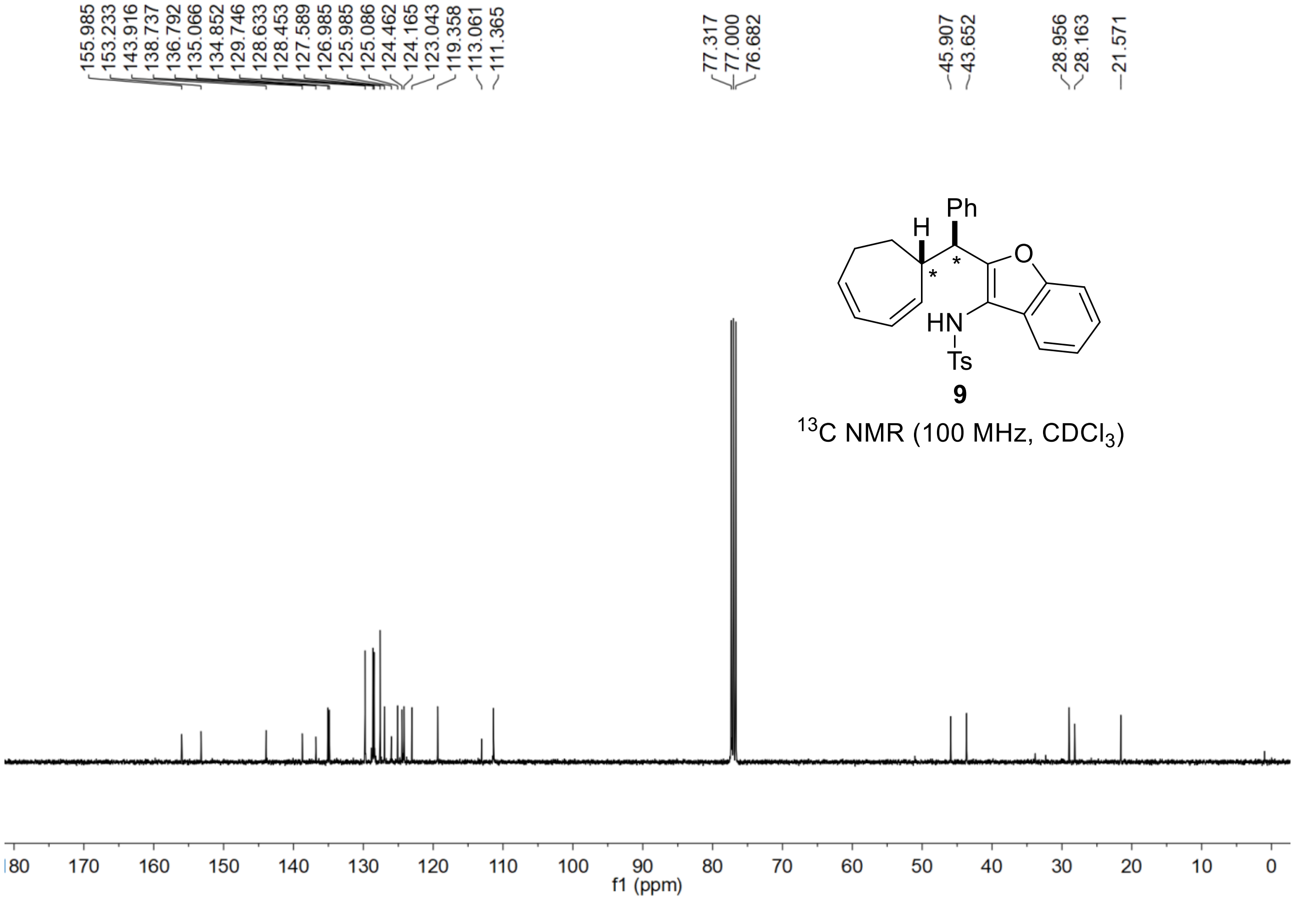
155.985 153.233 143.916 138.737 136.792 135.066 134.852 129.746 128.633 128.453 127.589 126.985 125.985 125.086 124.462 124.165 123.043 119.358 113.061 111.365
77.317 77.000 76.682
45.907 43.652
28.956 28.163
21.571
Ph
H
O
HN
Ts
9
13C NMR (100 MHz, CDCl3)
180 170 160 150 140 130 120 110 100 90 80 70 60 50 40 30 20 10 0
f1 (ppm)

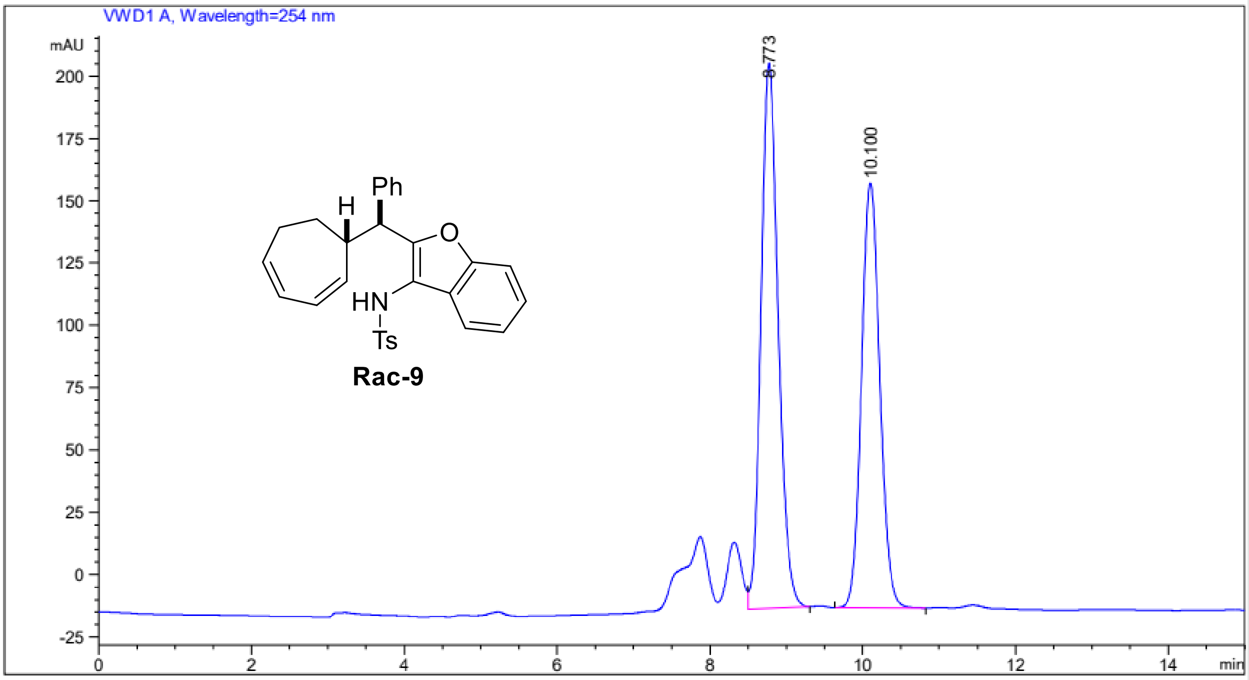

#	[min]		[min]	[mAU*s]	[mAU]	%
1	8.773	VB	0.2406	3418.23633	218.65475	54.6941
2	10.100	BB	0.2594	2831.50293	170.40413	45.3059

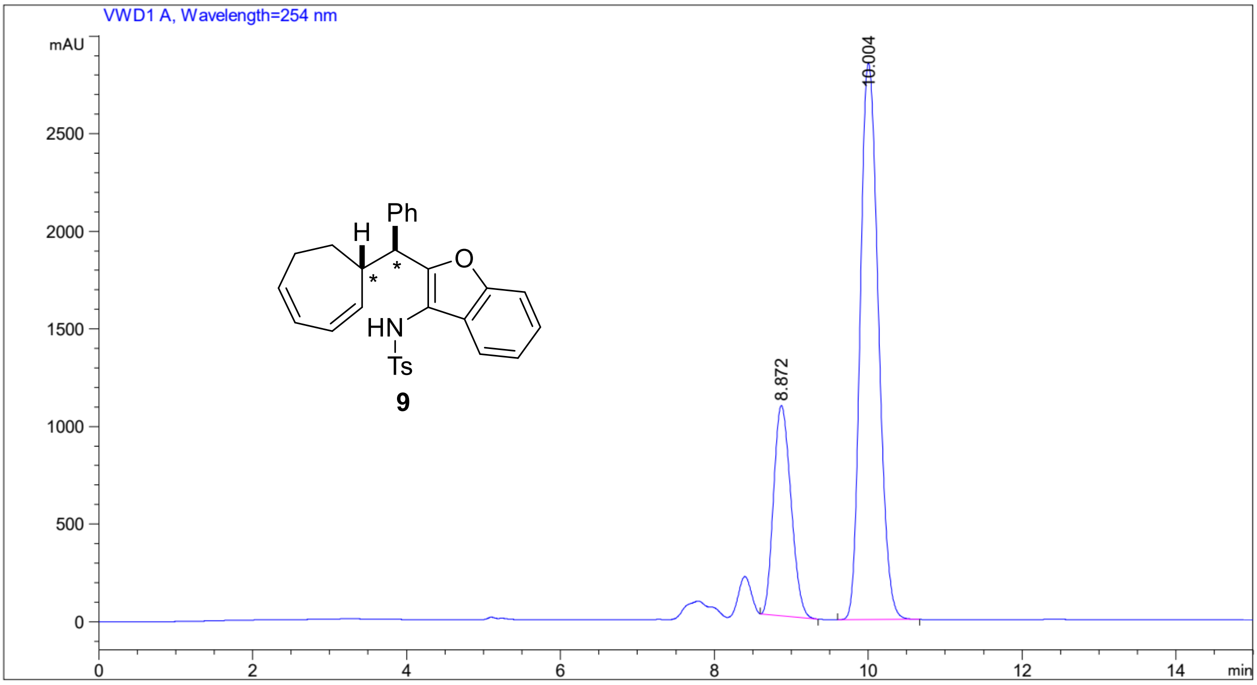

#	[min]		[min]	[mAU*s]	[mAU]	%
1	8.872	BBA	0.2502	1.69306e4	1078.31177	26.6872
2	10.004	BBA	0.2572	4.65102e4	2852.03198	73.3128

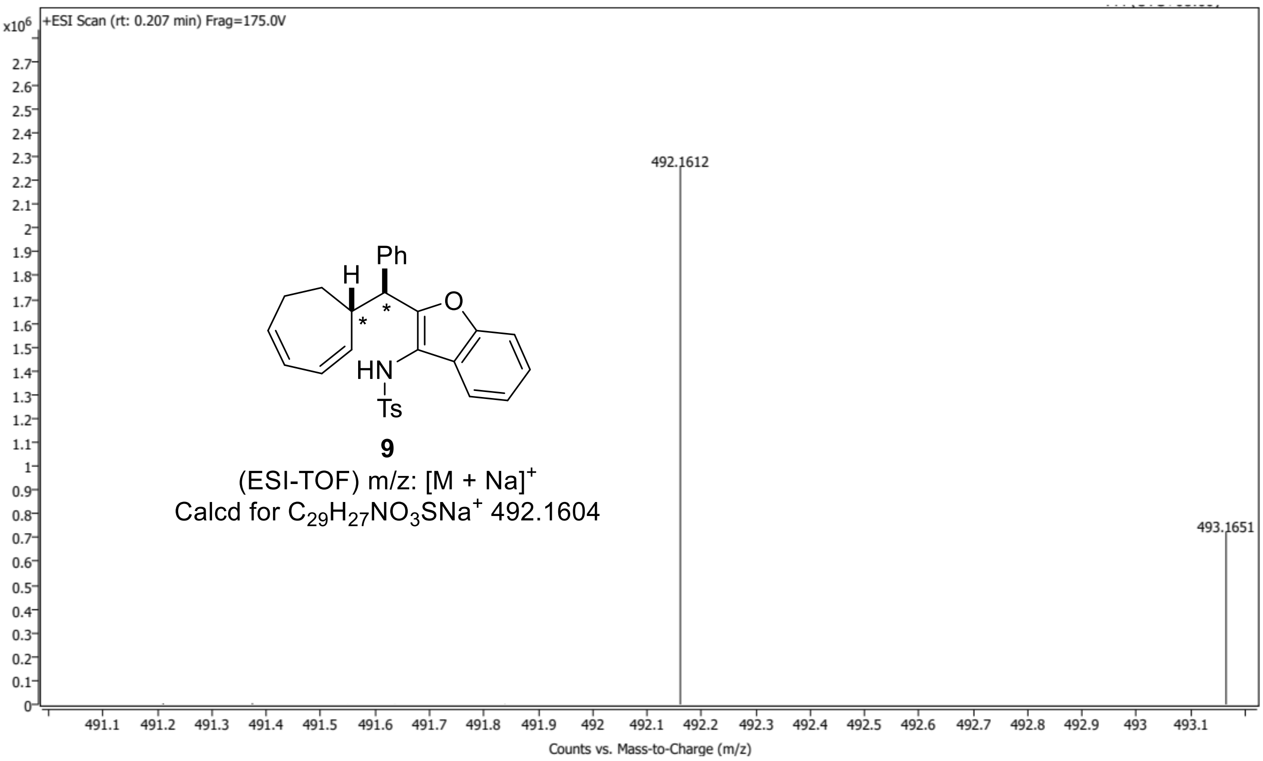
+ESI Scan (rt: 0.207 min) Frag=175.0V
x10^6
492.1612
493.1651
Ph
H
O
HN
Ts
9
(ESI-TOF) m/z: [M + Na]+
Calcd for C29H27NO3SNa+ 492.1604
Counts vs. Mass-to-Charge (m/z)

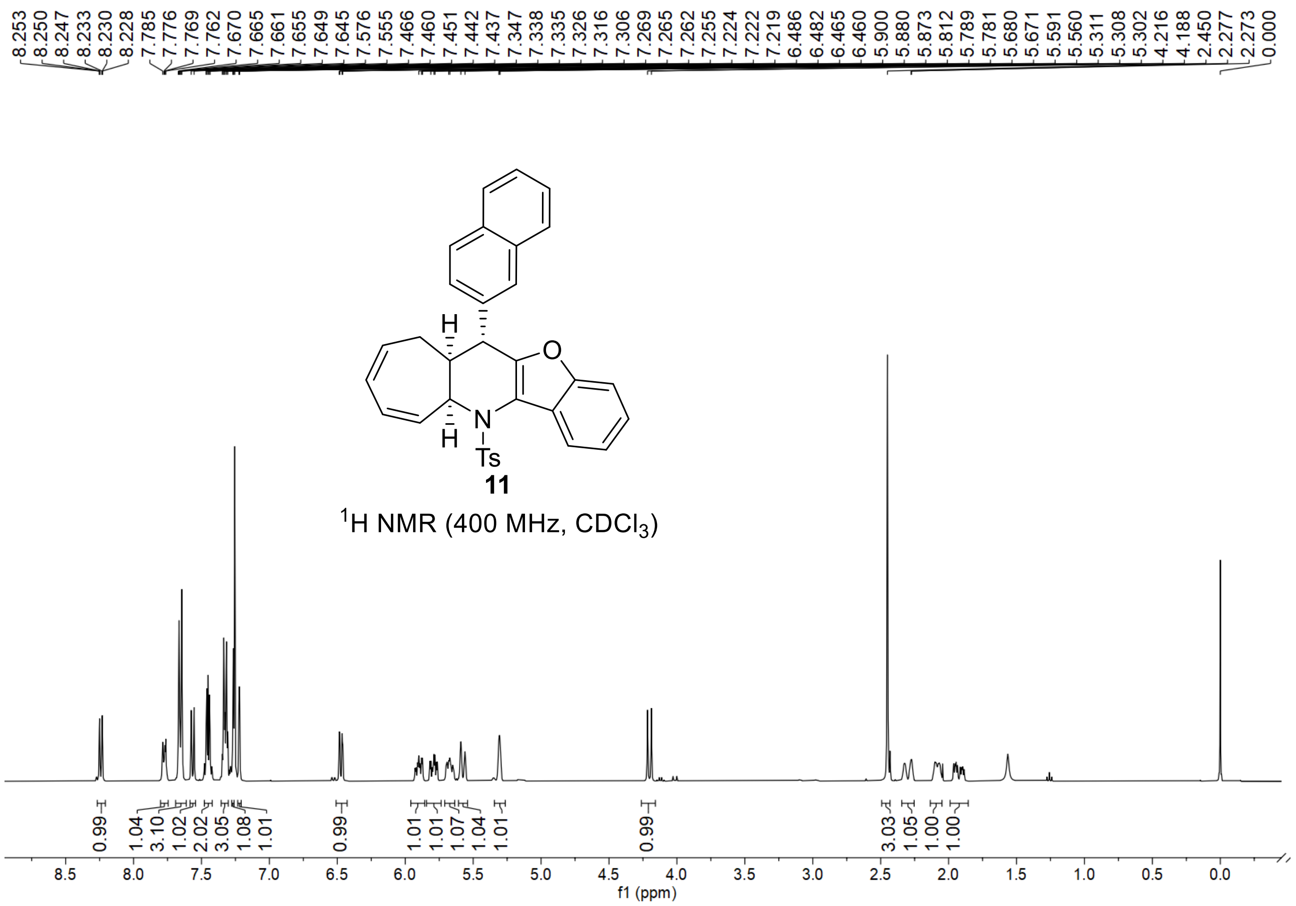
8.253
8.250
8.247
8.233
8.230
8.228
7.785
7.776
7.769
7.762
7.670
7.665
7.661
7.655
7.649
7.645
7.576
7.555
7.466
7.460
7.451
7.442
7.437
7.347
7.338
7.335
7.326
7.316
7.306
7.269
7.265
7.262
7.255
7.224
7.222
7.219
6.486
6.482
6.465
6.460
5.900
5.880
5.873
5.812
5.789
5.781
5.680
5.671
5.591
5.560
5.311
5.308
5.302
4.216
4.188
2.450
2.277
2.273
0.000
H
O
N
H
Ts
11
1H NMR (400 MHz, CDCl3)
0.99
1.04
3.10
1.02
2.02
3.05
1.08
1.01
0.99
1.01
1.01
1.07
1.04
1.01
0.99
3.03
1.05
1.00
1.00
f1 (ppm)

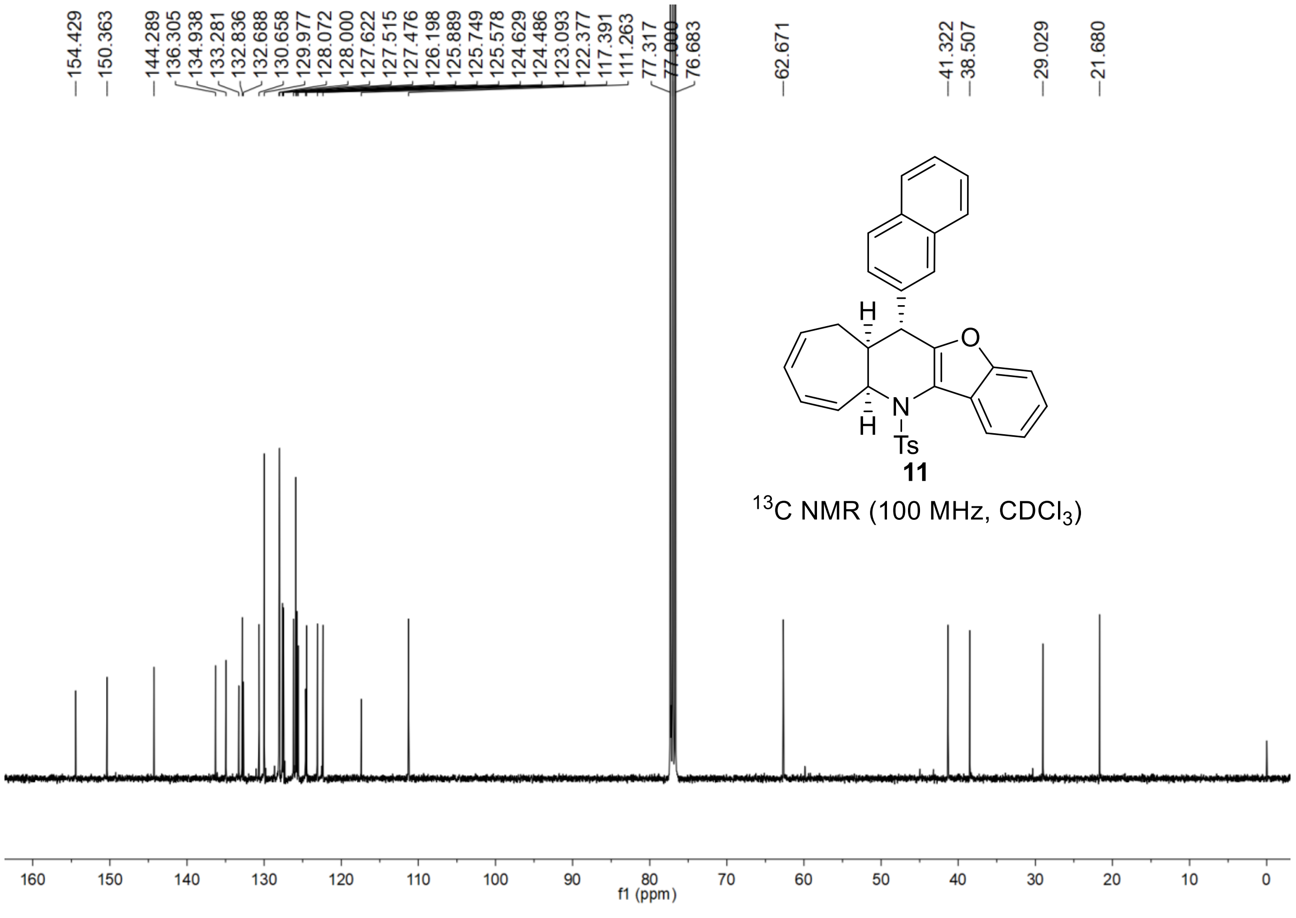
154.429
150.363
144.289
136.305
134.938
133.281
132.836
132.688
130.658
129.977
128.072
128.000
127.622
127.515
127.476
126.198
125.889
125.749
125.578
124.629
124.486
123.093
122.377
117.391
111.263
77.317
77.000
76.683
62.671
41.322
38.507
29.029
21.680
H
O
N
H
Ts
11
13C NMR (100 MHz, CDCl3)
f1 (ppm)

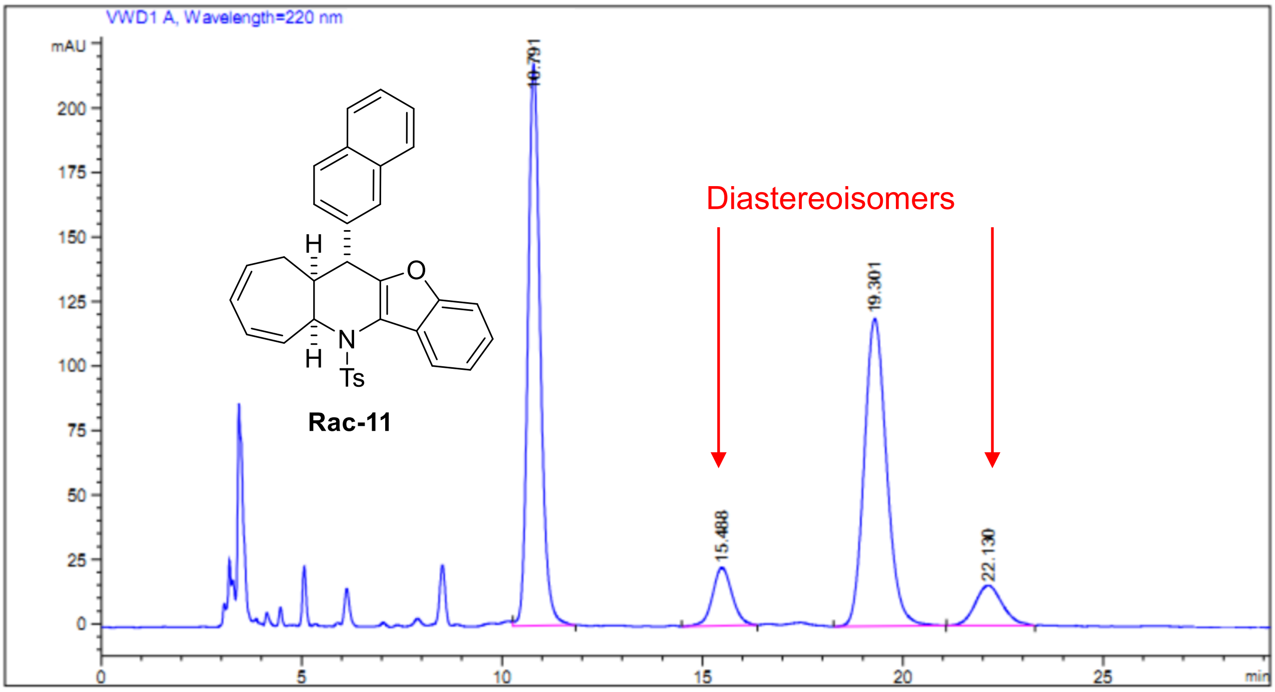

#	[min]		[min]	[mAU*s]	[mAU]	%
1	10.791	VB	0.3304	4667.36719	217.54463	43.1975
2	15.488	BB	0.5233	764.27209	22.48198	7.0735
3	19.301	BB	0.5989	4634.73438	119.27387	42.8955
4	22.130	BB	0.7169	738.33765	15.59334	6.8335

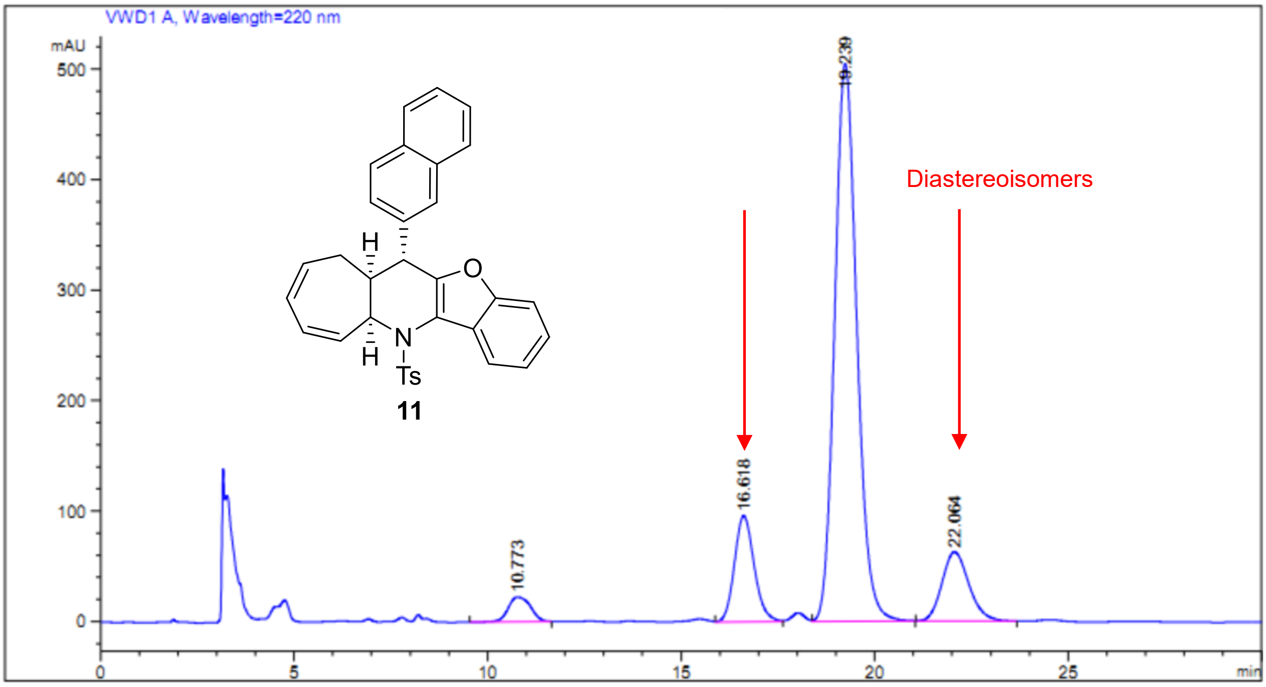

#	[min]		[min]	[mAU*s]	[mAU]	%
1	10.773	BB	0.6449	890.41455	22.37064	3.2569
2	16.618	VV	0.5473	3397.95801	95.90814	12.4288
3	19.239	VB	0.6132	2.00298e4	504.47507	73.2637
4	22.064	BB	0.7472	3021.17603	62.52697	11.0506

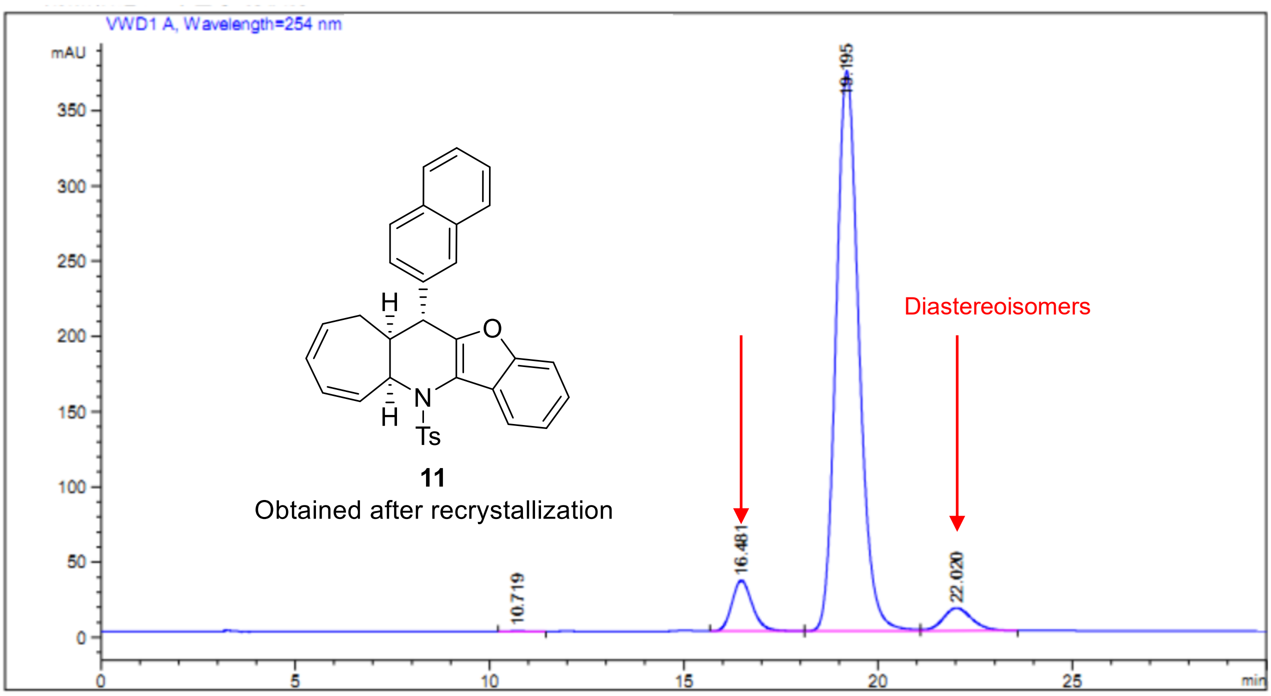

#	[min]		[min]	[mAU*s]	[mAU]	%
1	10.719	VB	0.3903	6.81185	2.07493e-1	0.0395
2	16.481	BB	0.5591	1227.66272	33.69169	7.1236
3	19.195	BV	0.6301	1.52169e4	372.03900	88.2974
4	22.020	VBA	0.7874	782.31226	15.14768	4.5394

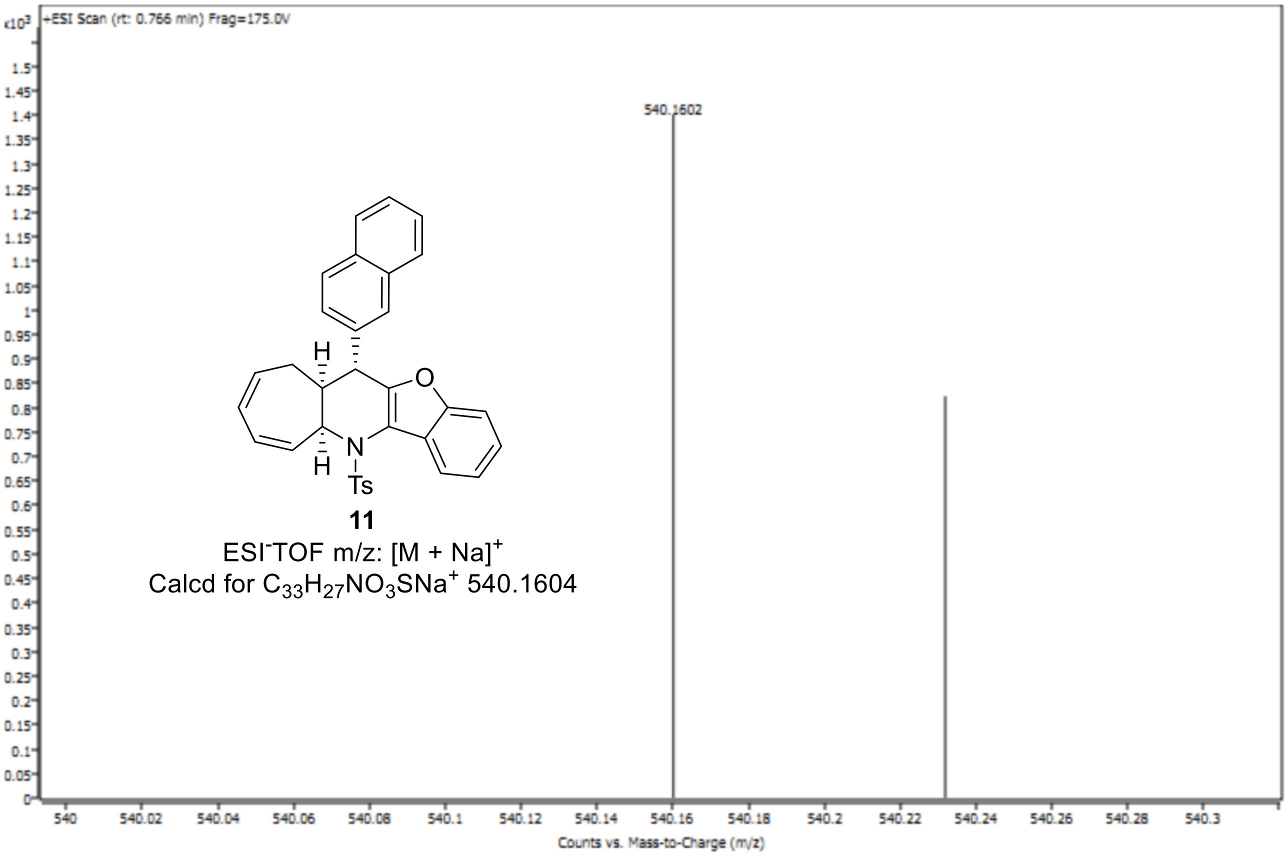

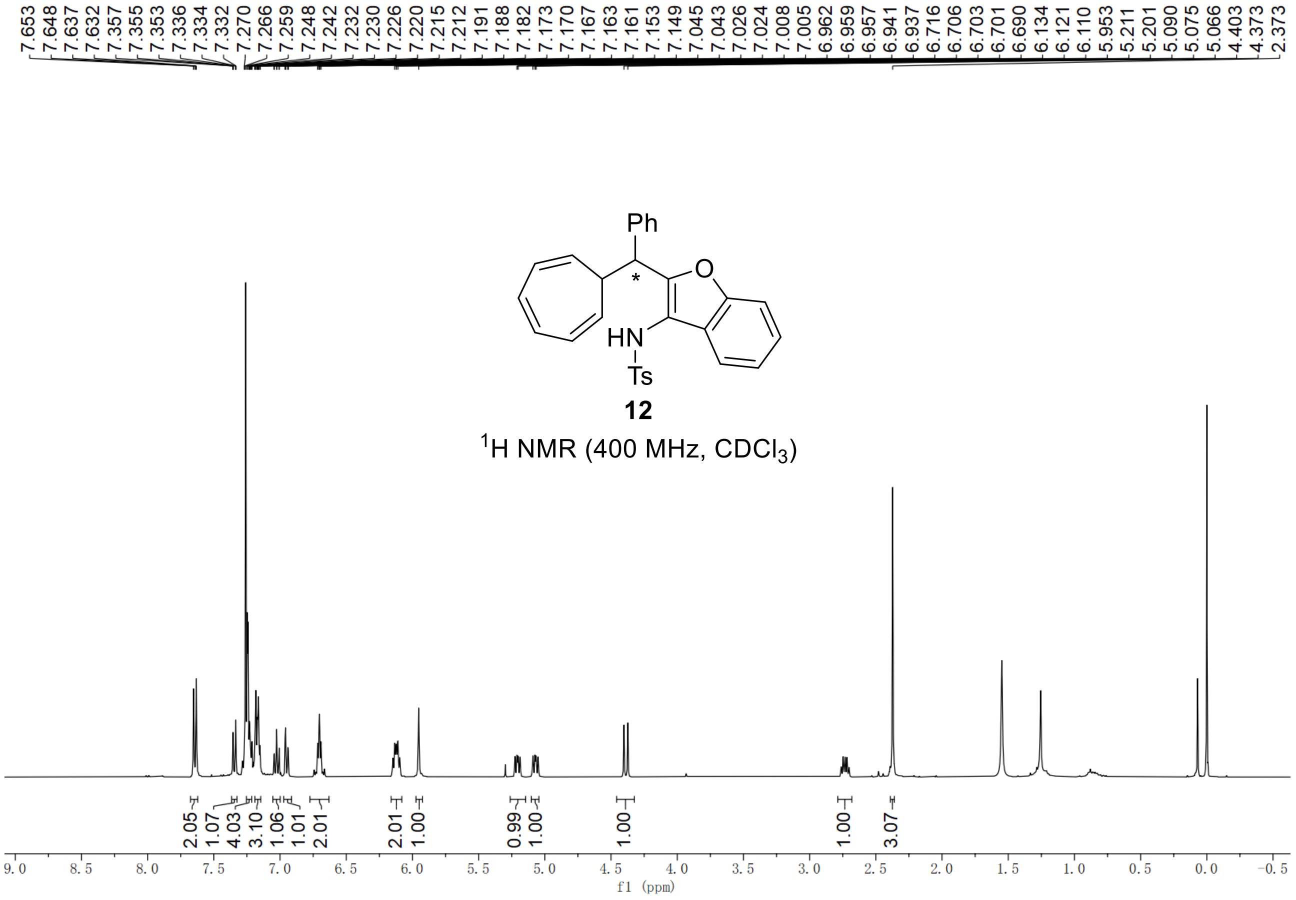

7.653 7.648 7.637 7.632 7.357 7.355 7.353 7.336 7.334 7.332 7.270 7.266 7.259 7.248 7.242 7.232 7.230 7.226 7.220 7.215 7.212 7.191 7.188 7.182 7.173 7.170 7.167 7.163 7.161 7.153 7.149 7.045 7.043 7.026 7.024 7.008 7.005 6.962 6.959 6.957 6.941 6.937 6.716 6.706 6.703 6.701 6.690 6.134 6.121 6.110 5.953 5.211 5.201 5.090 5.075 5.066 4.403 4.373 2.373
Ph
O
HN
Ts
12
1H NMR (400 MHz, CDCl3)
2.05 1.07 4.03 3.10 1.06 1.01 2.01 2.01 1.00 0.99 1.00 1.00 1.00 3.07
f1 (ppm)

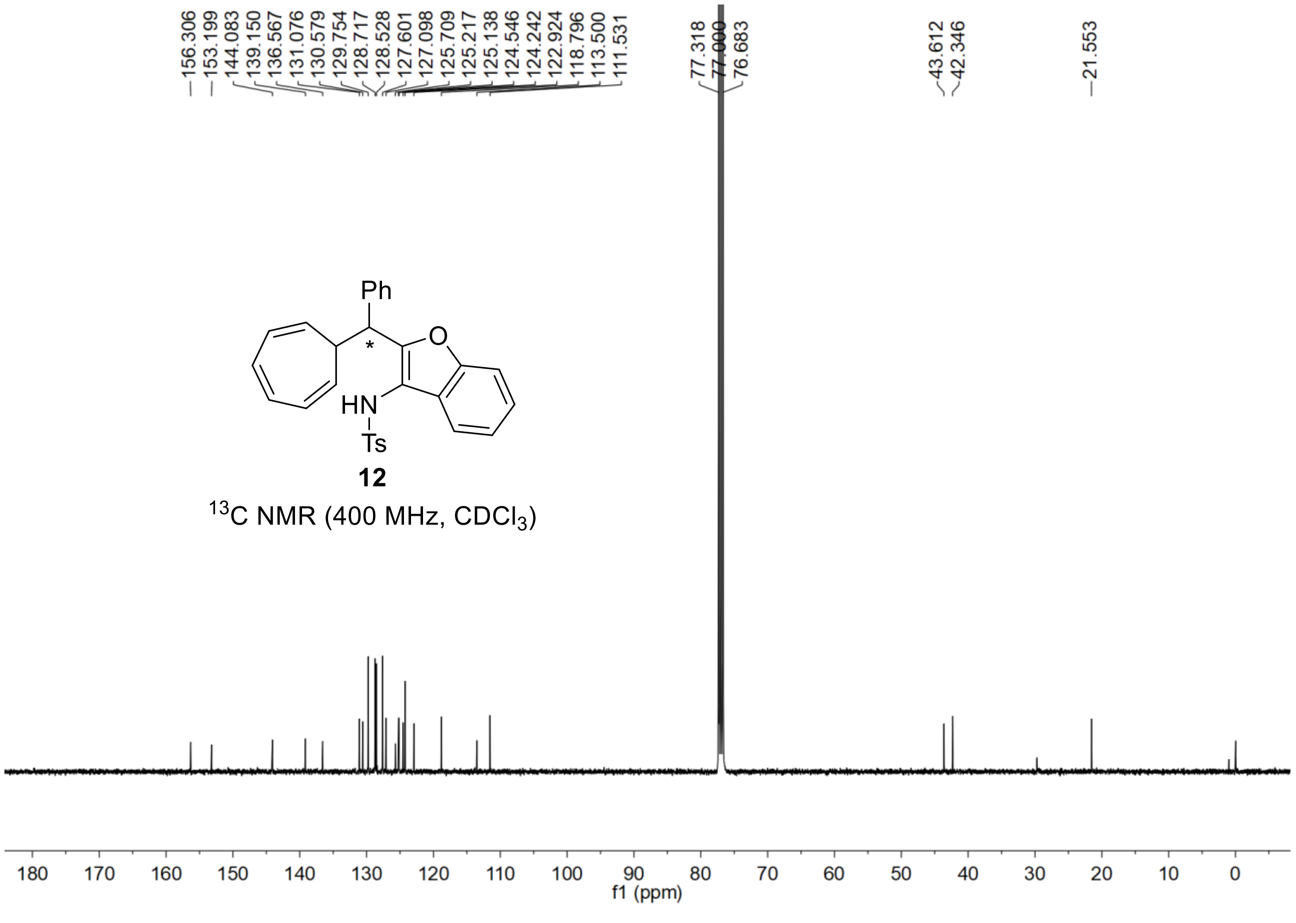

156.306 153.199 144.083 139.150 136.567 131.076 130.579 129.754 128.717 128.528 127.601 127.098 125.709 125.217 125.138 124.546 124.242 122.924 118.796 113.500 111.531
77.318 77.000 76.683
43.612 42.346
21.553
Ph
O
HN
Ts
12
13C NMR (400 MHz, CDCl3)
f1 (ppm)

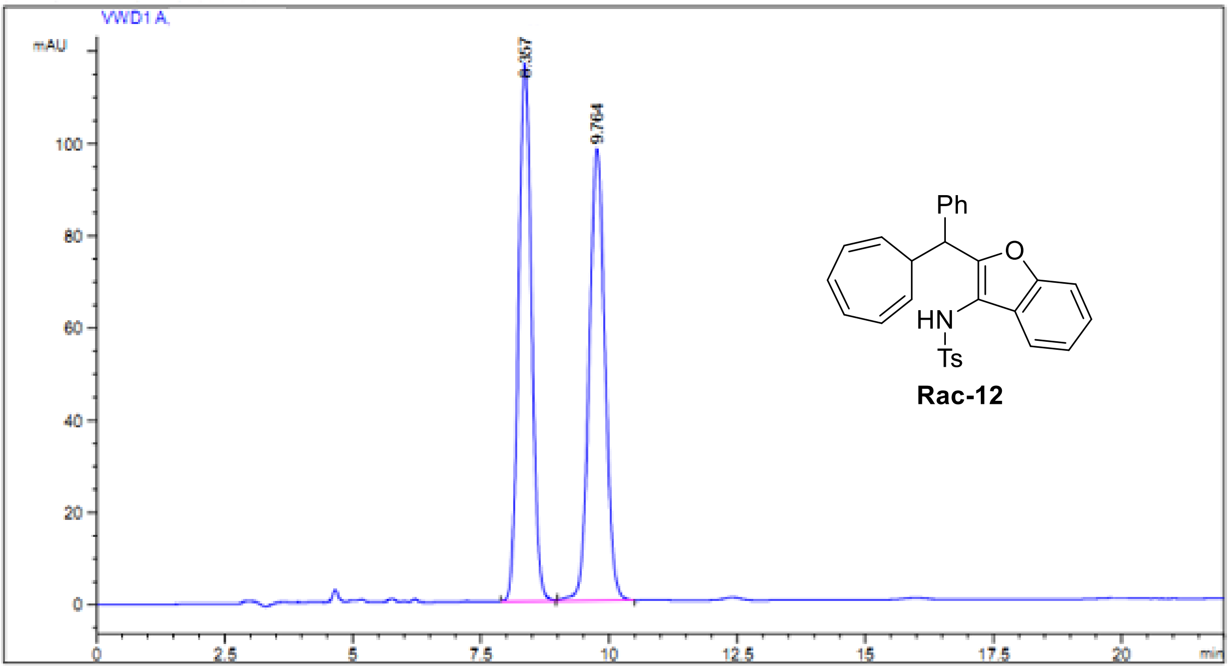

#	[min]		[min]	mAU *s	[mAU]	%
1	8.357	BB	0.2756	2071.52319	116.67725	49.6487
2	9.764	BB	0.3333	2100.84229	97.90084	50.3513

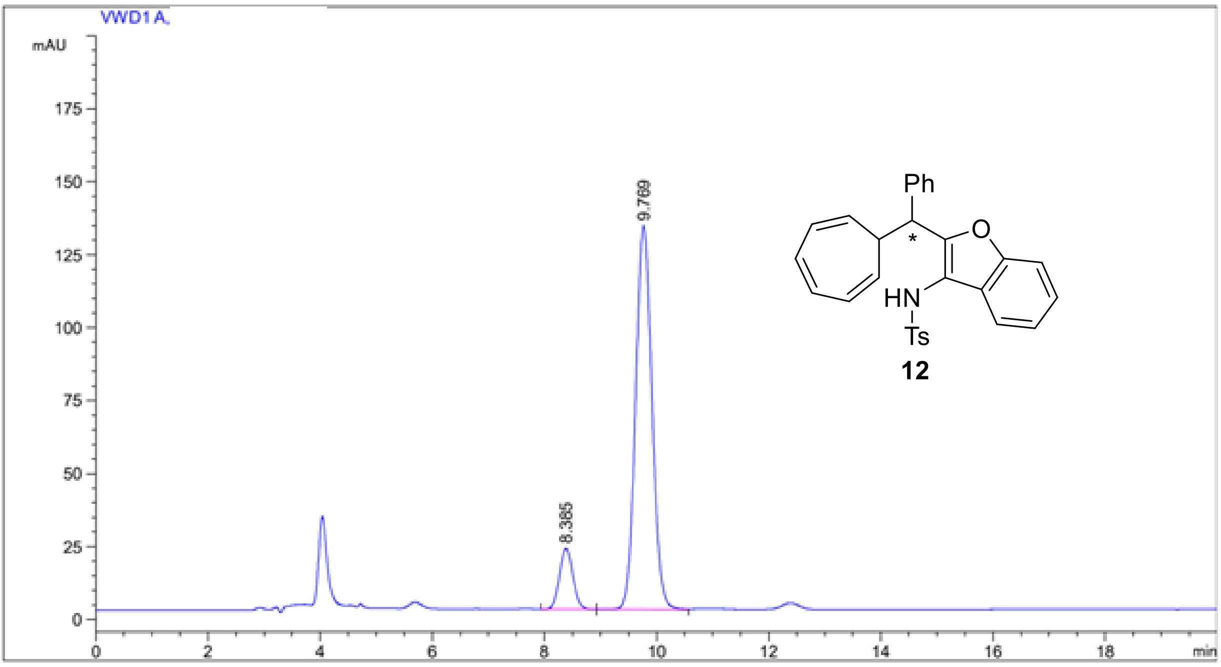

#	[min]		[min]	mAU *s	[mAU]	%
1	8.385	BB	0.2596	350.31448	20.90230	11.6298
2	9.769	BV	0.3150	2661.90674	131.40013	88.3702

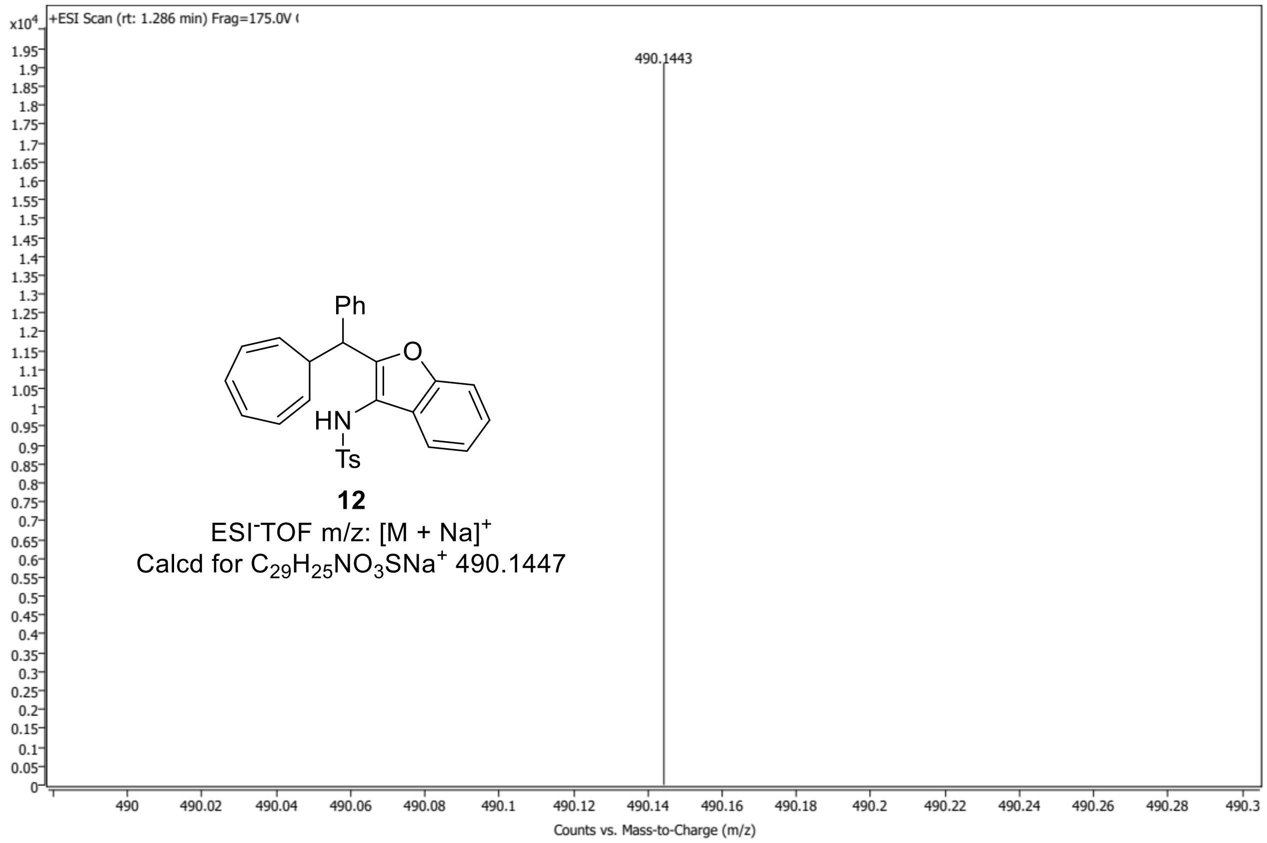
+ESI Scan (rt: 1.286 min) Frag=175.0V
x10^4
490.1443
Counts vs. Mass-to-Charge (m/z)
Ph
O
HN
Ts
12
ESI-TOF m/z: [M + Na]+
Calcd for C29H25NO3SNa+ 490.1447

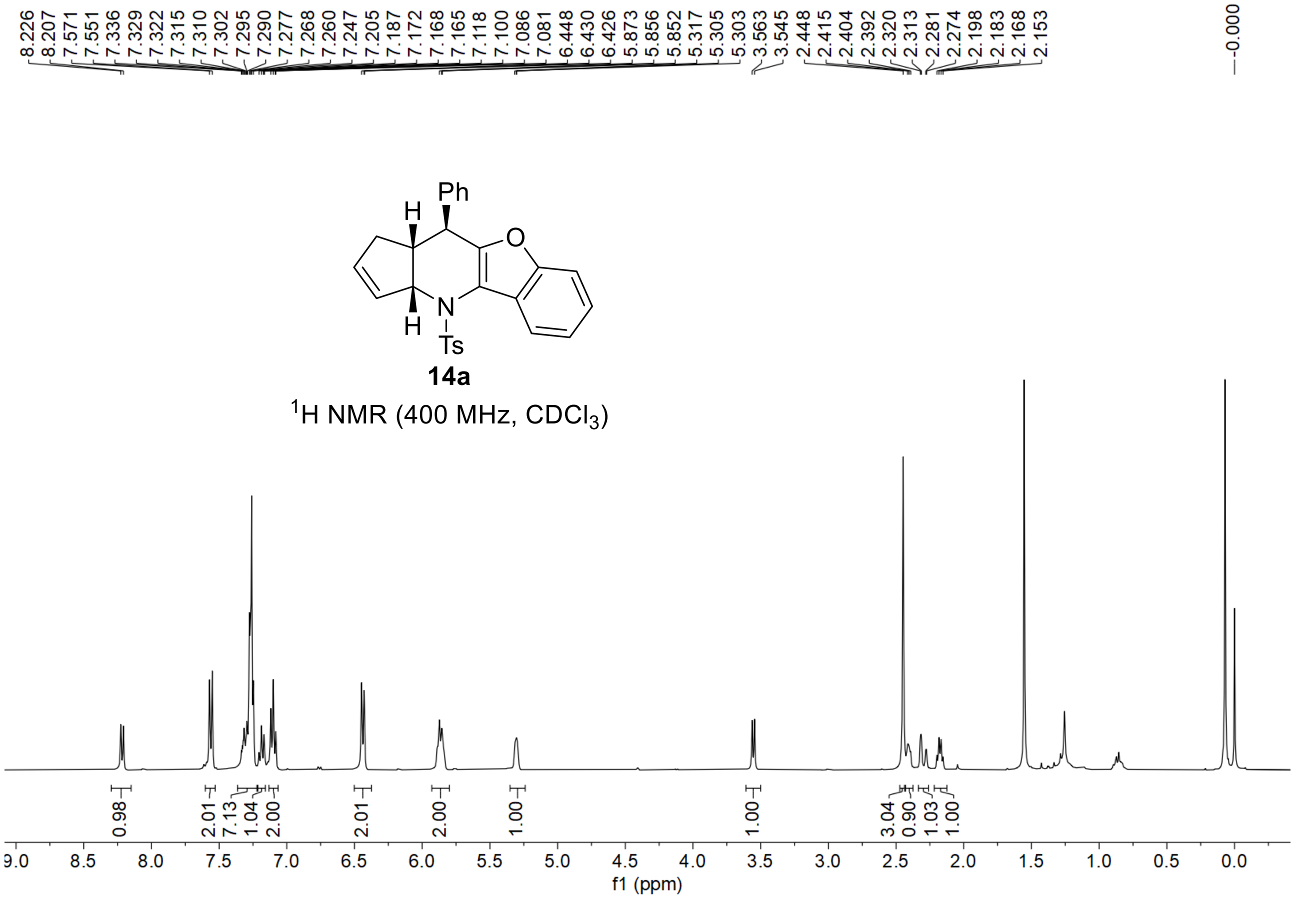
8.226 8.207 7.571 7.551 7.336 7.329 7.322 7.315 7.310 7.302 7.295 7.290 7.277 7.268 7.260 7.247 7.205 7.187 7.172 7.168 7.165 7.118 7.100 7.086 7.081 6.448 6.430 6.426 5.873 5.856 5.852 5.317 5.305 5.303 3.563 3.545 2.448 2.415 2.404 2.392 2.320 2.313 2.281 2.274 2.198 2.183 2.168 2.153
0.000
Ph
H
O
N
H
Ts
14a
1H NMR (400 MHz, CDCl3)
0.98 2.01 7.13 1.04 2.00 2.01 2.00 1.00 1.00 3.04 0.90 1.03 1.00
9.0 8.5 8.0 7.5 7.0 6.5 6.0 5.5 5.0 4.5 4.0 3.5 3.0 2.5 2.0 1.5 1.0 0.5 0.0
f1 (ppm)

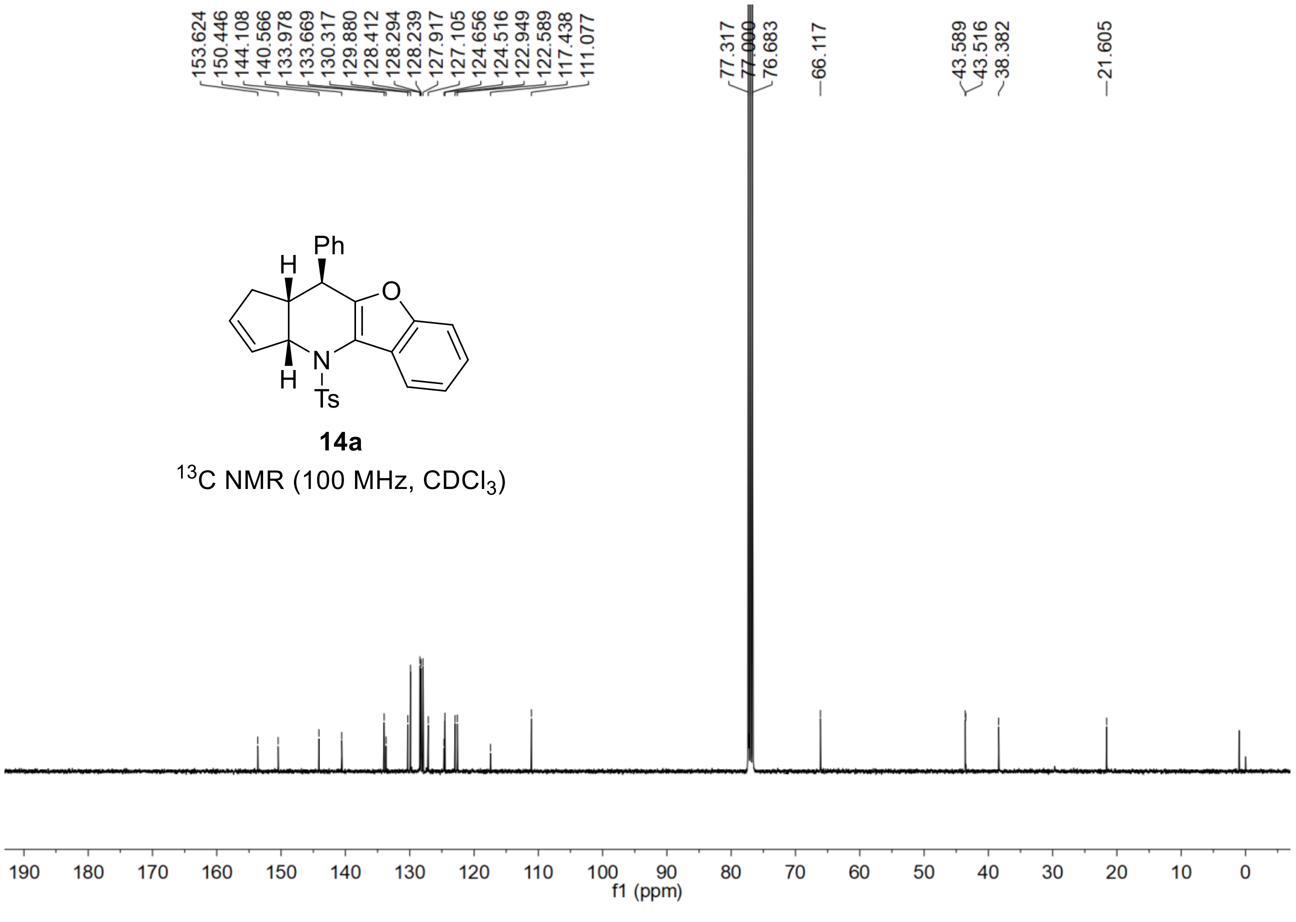
153.624 150.446 144.108 140.566 133.978 133.669 130.317 129.880 128.412 128.294 128.239 127.917 127.105 124.656 124.516 122.949 122.589 117.438 111.077
77.317 77.000 76.683
66.117
43.589 43.516 38.382
21.605
Ph
H
O
N
H
Ts
14a
13C NMR (100 MHz, CDCl3)
190 180 170 160 150 140 130 120 110 100 90 80 70 60 50 40 30 20 10 0
f1 (ppm)

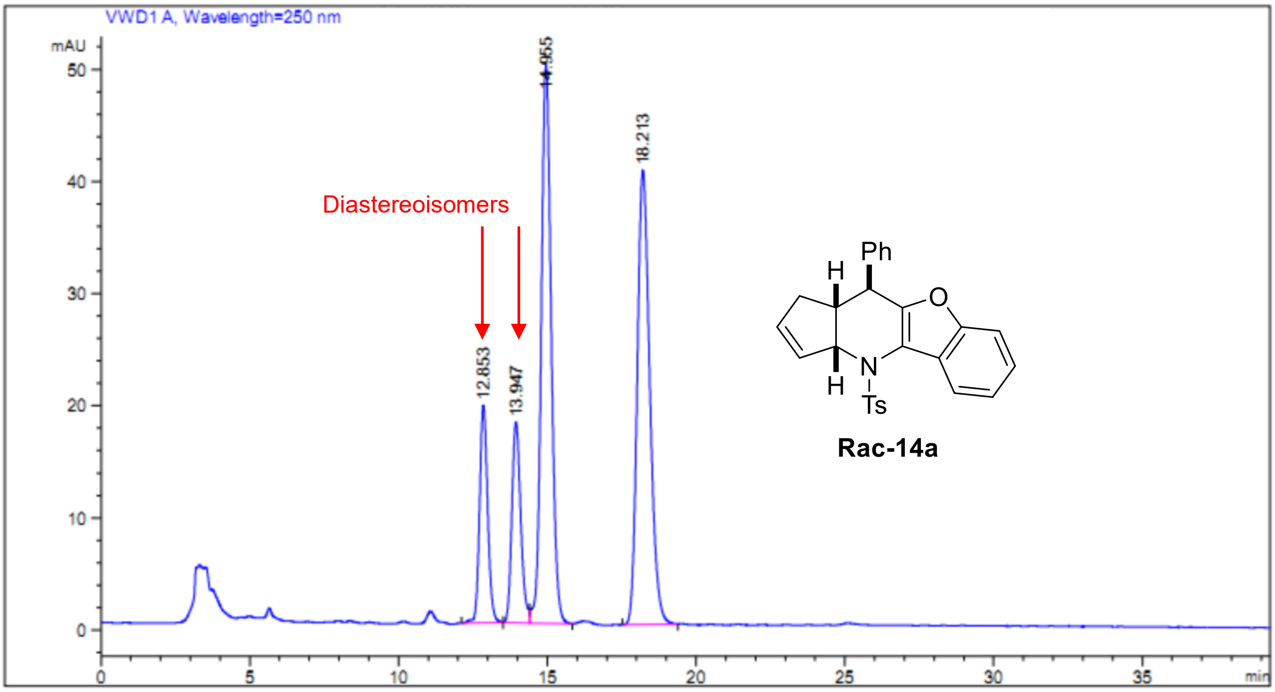

#	[min]		[min]	[mAU*s]	[mAU]	%
1	12.853	BB	0.2916	366.40060	19.40357	11.9039
2	13.947	BV	0.3182	369.78287	17.89857	12.0138
3	14.955	VB	0.3605	1160.38916	49.83633	37.6996
4	18.213	BB	0.4510	1181.41772	40.50112	38.3828

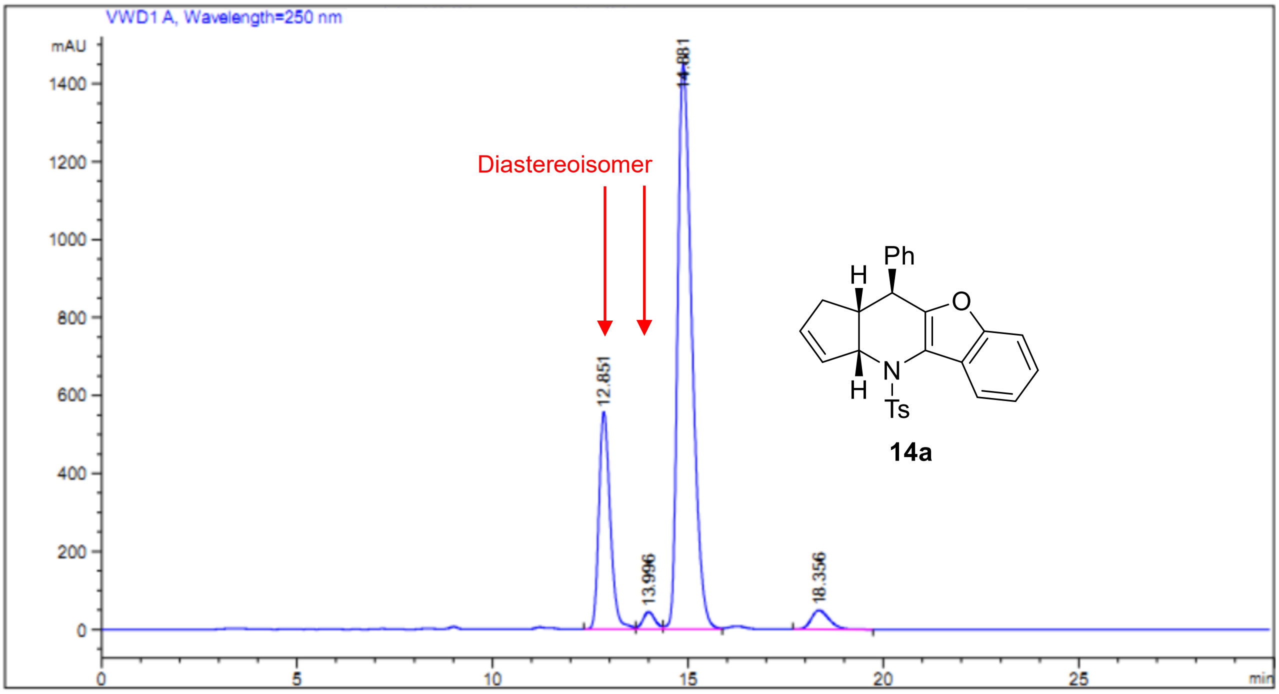

#	[min]		[min]	[mAU*s]	[mAU]	%
1	12.851	BV	0.3094	1.11714e4	557.65723	22.1517
2	13.996	VV	0.3319	960.78424	44.50592	1.9051
3	14.881	VV	0.3922	3.67631e4	1448.58972	72.8971
4	18.356	BB	0.4811	1536.18640	48.95470	3.0461

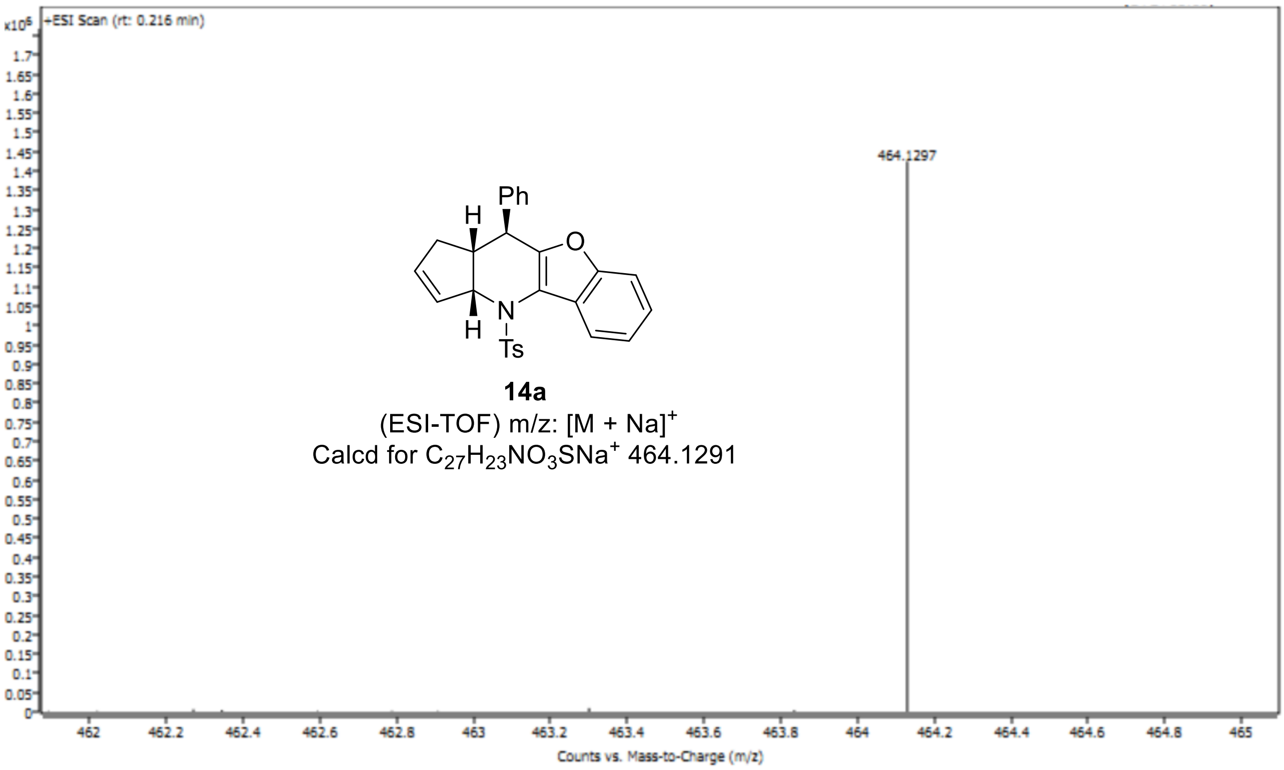
x10^6
+ESI Scan (rt: 0.216 min)
464.1297
Ph
H
O
N
H
Ts
14a
(ESI-TOF) m/z: [M + Na]+
Calcd for C27H23NO3SNa+ 464.1291
Counts vs. Mass-to-Charge (m/z)

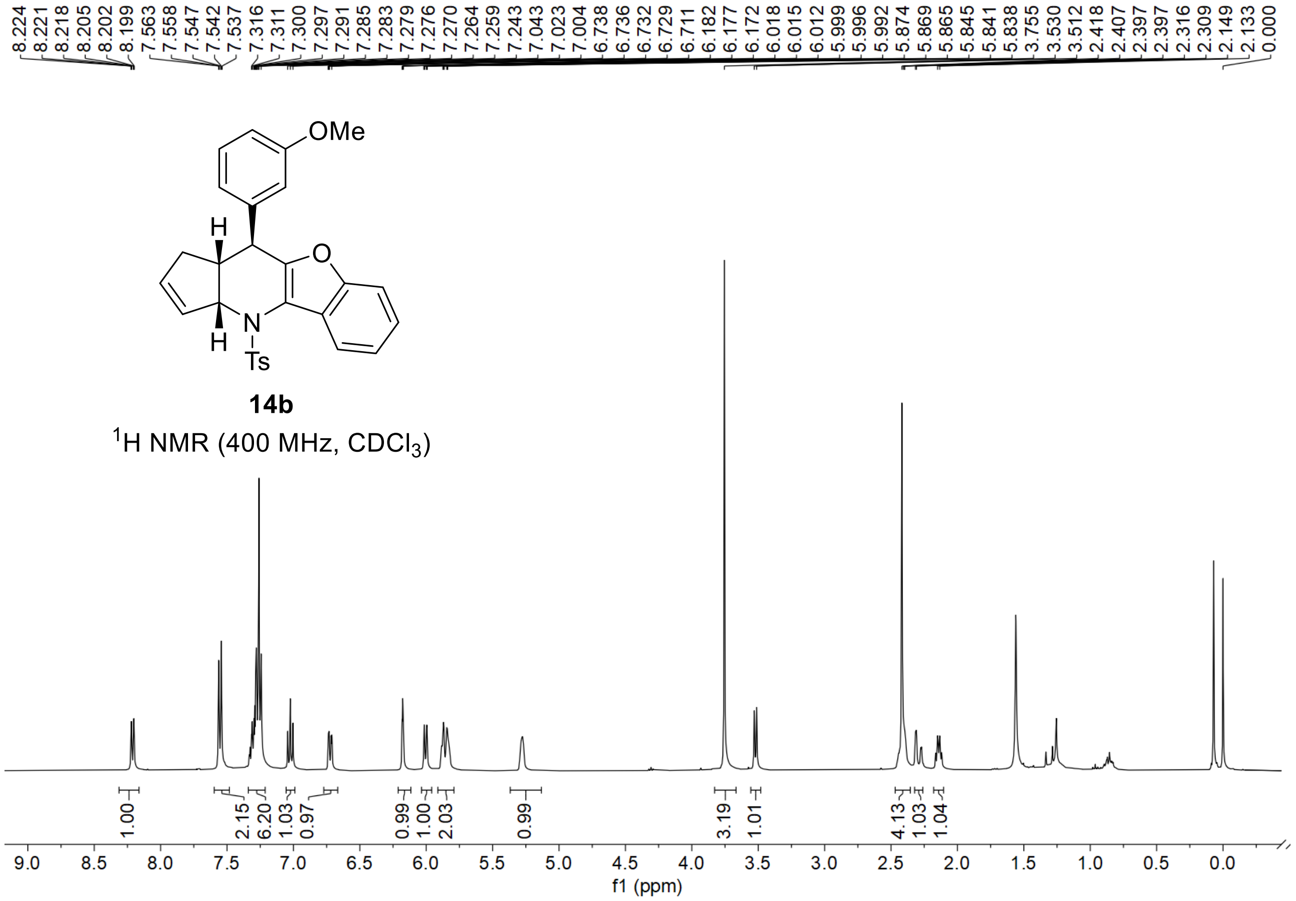
8.224 8.221 8.218 8.205 8.202 8.199 7.563 7.558 7.547 7.542 7.537 7.316 7.311 7.300 7.297 7.291 7.285 7.283 7.279 7.276 7.270 7.264 7.259 7.243 7.043 7.023 7.004 6.738 6.736 6.732 6.729 6.711 6.182 6.177 6.172 6.018 6.015 6.012 5.999 5.996 5.992 5.874 5.869 5.865 5.845 5.841 5.838 3.755 3.530 3.512 2.418 2.407 2.397 2.397 2.316 2.309 2.149 2.133 0.000
OMe
H
O
N
H
Ts
14b
1H NMR (400 MHz, CDCl3)
1.00 2.15 6.20 1.03 0.97 0.99 1.00 2.03 0.99 3.19 1.01 4.13 1.03 1.04
9.0 8.5 8.0 7.5 7.0 6.5 6.0 5.5 5.0 4.5 4.0 3.5 3.0 2.5 2.0 1.5 1.0 0.5 0.0
f1 (ppm)

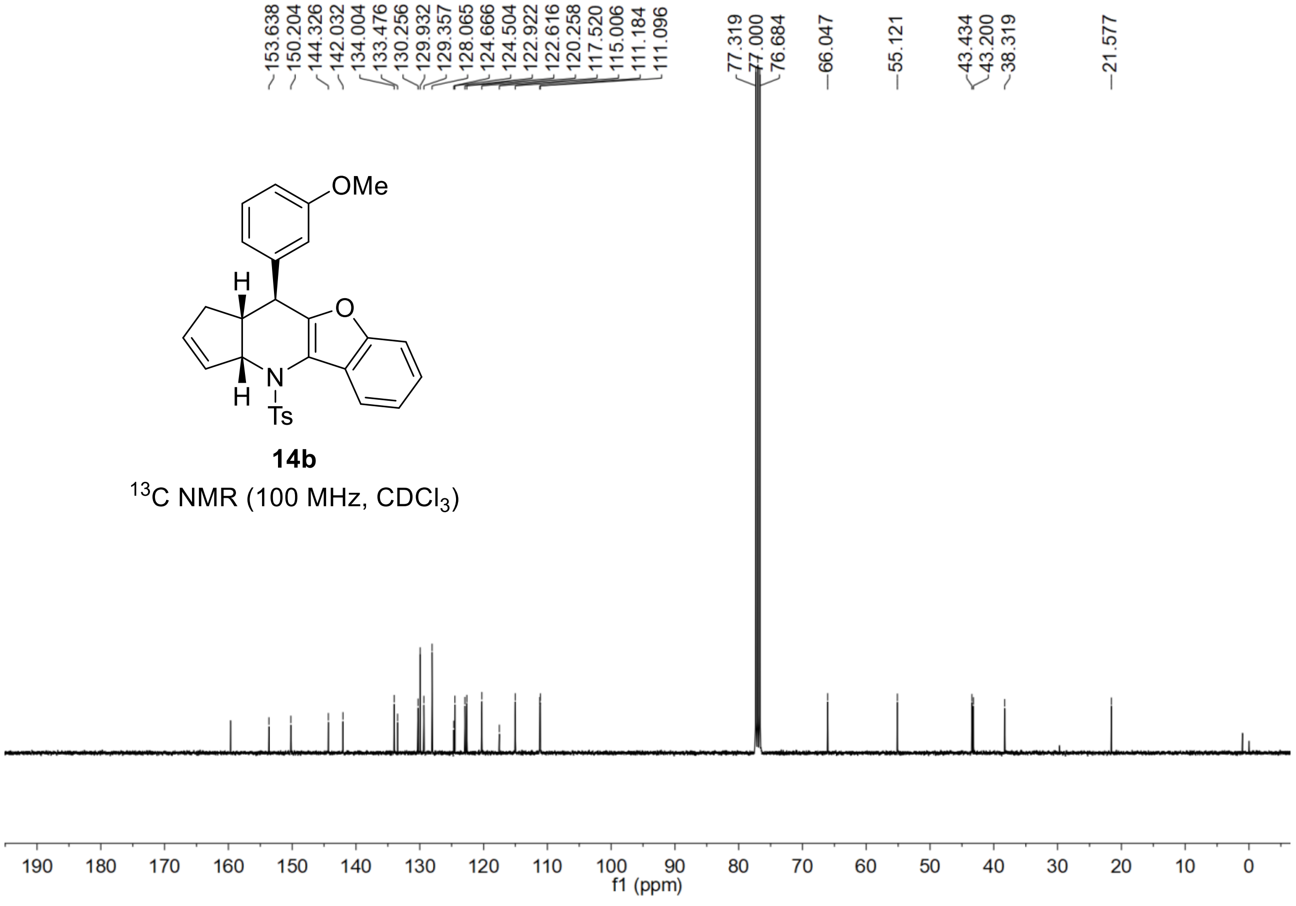
153.638 150.204 144.326 142.032 134.004 133.476 130.256 129.932 129.357 128.065 124.666 124.504 122.922 122.616 120.258 117.520 115.006 111.184 111.096
77.319 77.000 76.684
66.047
55.121
43.434 43.200 38.319
21.577
OMe
H
O
N
H
Ts
14b
13C NMR (100 MHz, CDCl3)
190 180 170 160 150 140 130 120 110 100 90 80 70 60 50 40 30 20 10 0
f1 (ppm)

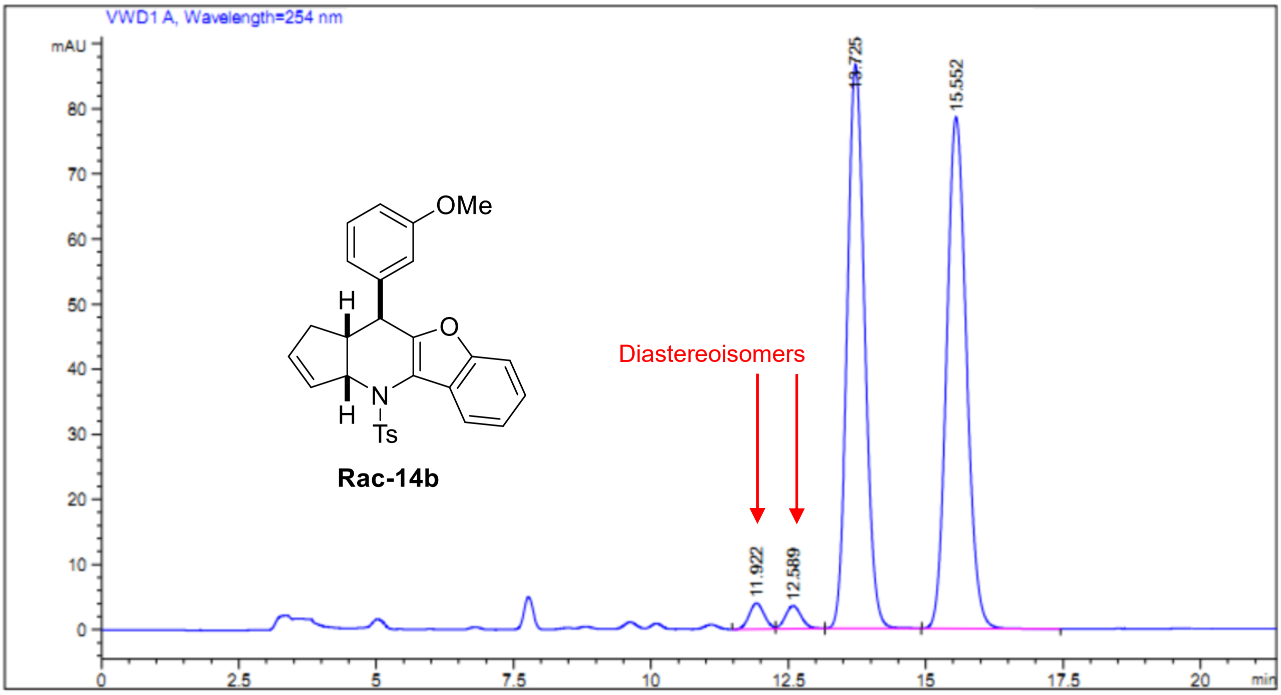

#	[min]		[min]	[mAU*s]	[mAU]	%
1	11.922	BV	0.2992	75.19041	3.97623	1.8714
2	12.589	VB	0.3089	69.20010	3.50649	1.7223
3	13.725	BB	0.3450	1911.88049	86.58559	47.5842
4	15.552	BB	0.3903	1961.61499	78.56947	48.8221

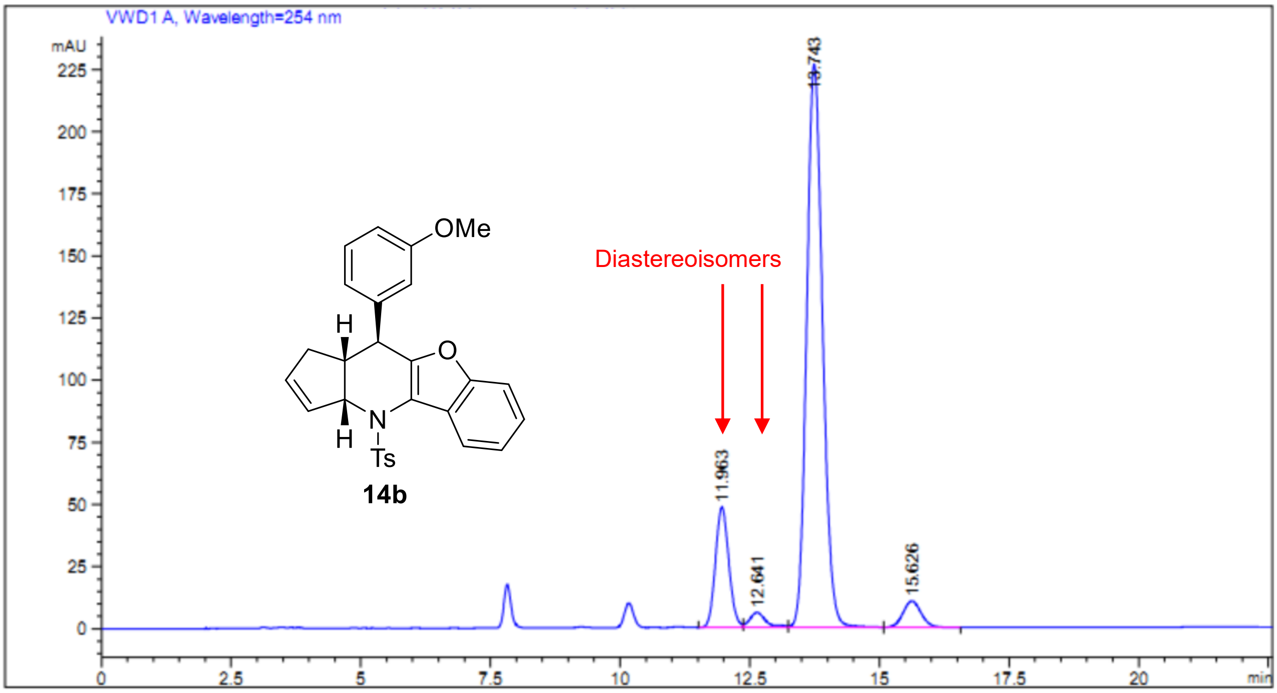

#	[min]		[min]	[mAU*s]	[mAU]	%
1	11.963	BV	0.2804	871.87396	48.31780	14.3100
2	12.641	VV	0.3276	131.92065	6.04084	2.1652
3	13.743	VB	0.3306	4833.42676	226.38196	79.3306
4	15.626	BB	0.3757	255.54683	10.55185	4.1943

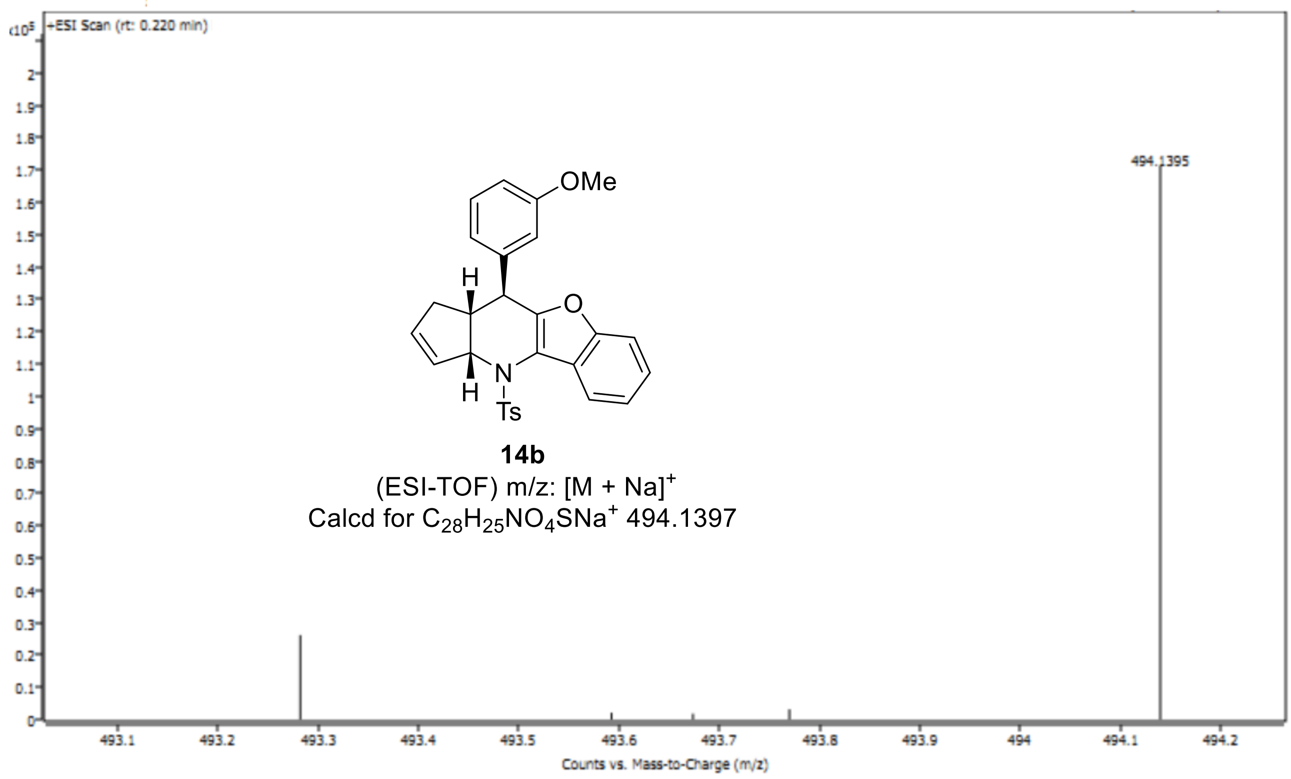
+ESI Scan (rt: 0.220 min)
x10^5
494.1395
14b
(ESI-TOF) m/z: [M + Na]+
Calcd for C28H25NO4SNa+ 494.1397
OMe
H
O
N
H
Ts
Counts vs. Mass-to-Charge (m/z)

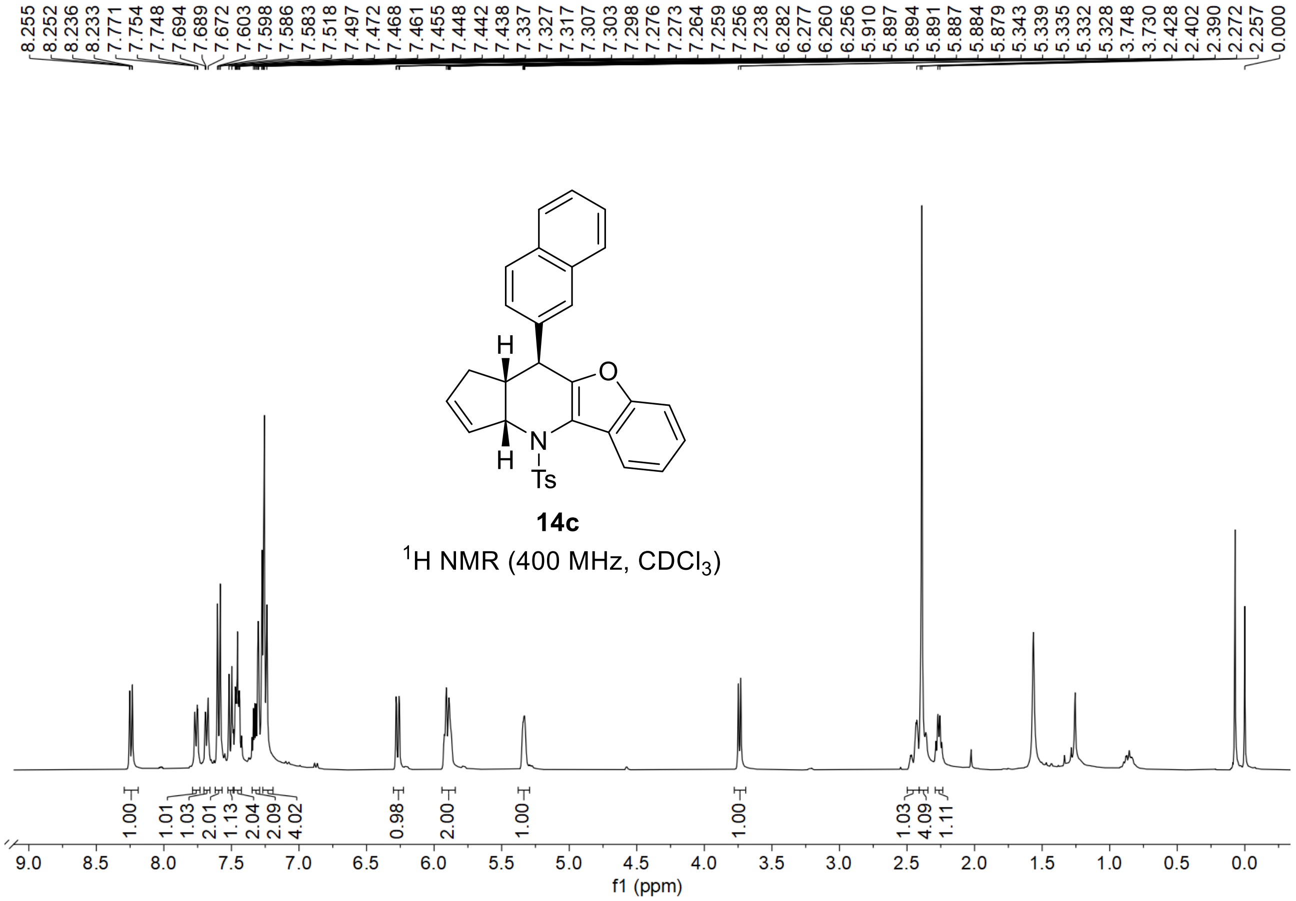
8.255
8.252
8.236
8.233
7.771
7.754
7.748
7.694
7.689
7.672
7.603
7.598
7.586
7.583
7.518
7.497
7.472
7.468
7.461
7.455
7.448
7.442
7.438
7.337
7.327
7.317
7.307
7.303
7.298
7.276
7.273
7.264
7.259
7.256
7.238
6.282
6.277
6.260
6.256
5.910
5.897
5.894
5.891
5.887
5.884
5.879
5.343
5.339
5.335
5.332
5.328
3.748
3.730
2.428
2.402
2.390
2.272
2.257
0.000
H
O
H
N
Ts
14c
^{1}H NMR (400 MHz, $CDCl_3$)
1.00
1.01
1.03
2.01
1.13
2.04
2.09
4.02
0.98
2.00
1.00
1.00
1.03
4.09
1.11
9.0 8.5 8.0 7.5 7.0 6.5 6.0 5.5 5.0 4.5 4.0 3.5 3.0 2.5 2.0 1.5 1.0 0.5 0.0
f1 (ppm)

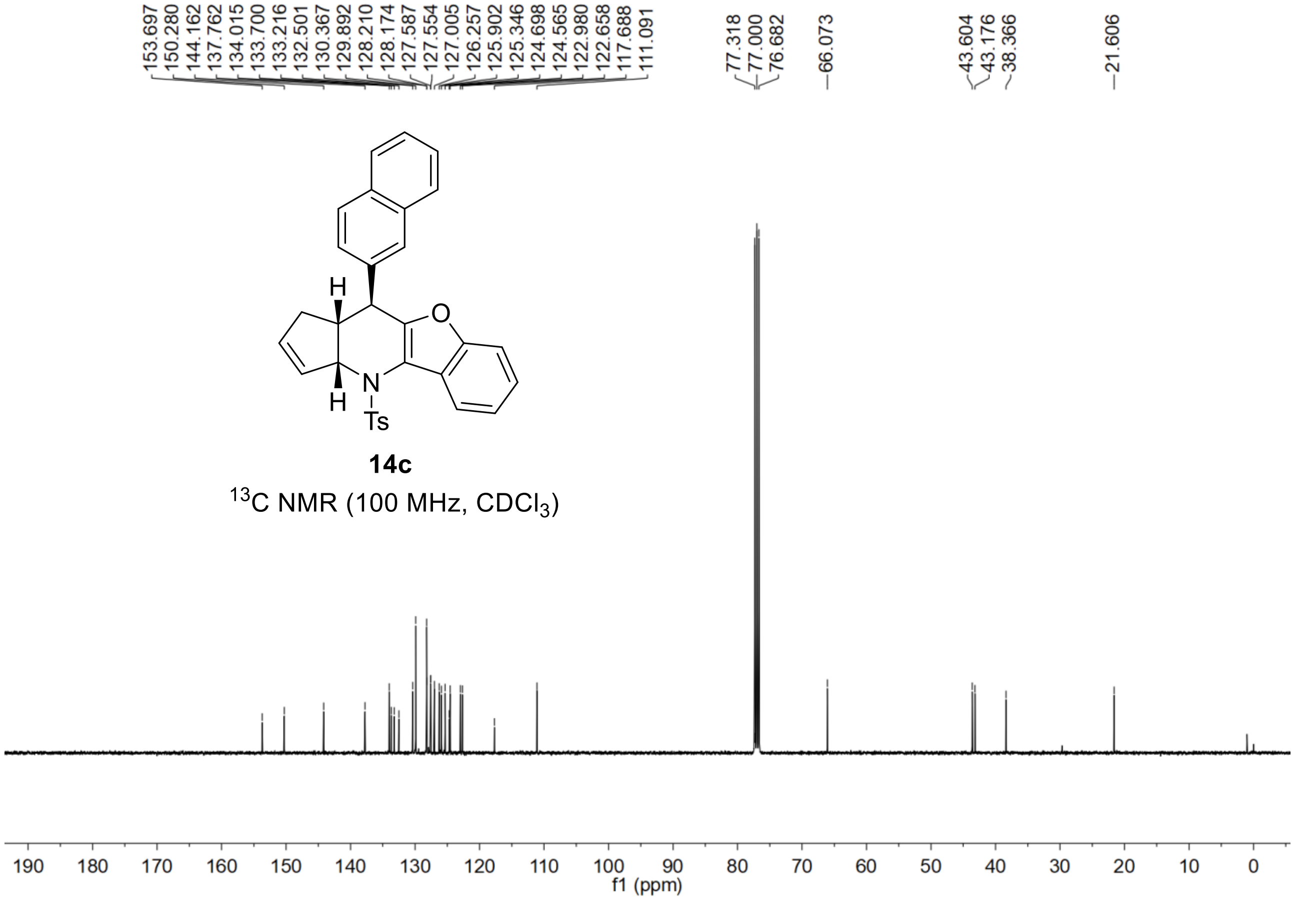
153.697
150.280
144.162
137.762
134.015
133.700
133.216
132.501
130.367
129.892
128.210
128.174
127.587
127.554
127.005
126.257
125.902
125.346
124.698
124.565
122.980
122.658
117.688
111.091
77.318
77.000
76.682
66.073
43.604
43.176
38.366
21.606
H
O
H
N
Ts
14c
^{13}C NMR (100 MHz, $CDCl_3$)
190 180 170 160 150 140 130 120 110 100 90 80 70 60 50 40 30 20 10 0
f1 (ppm)

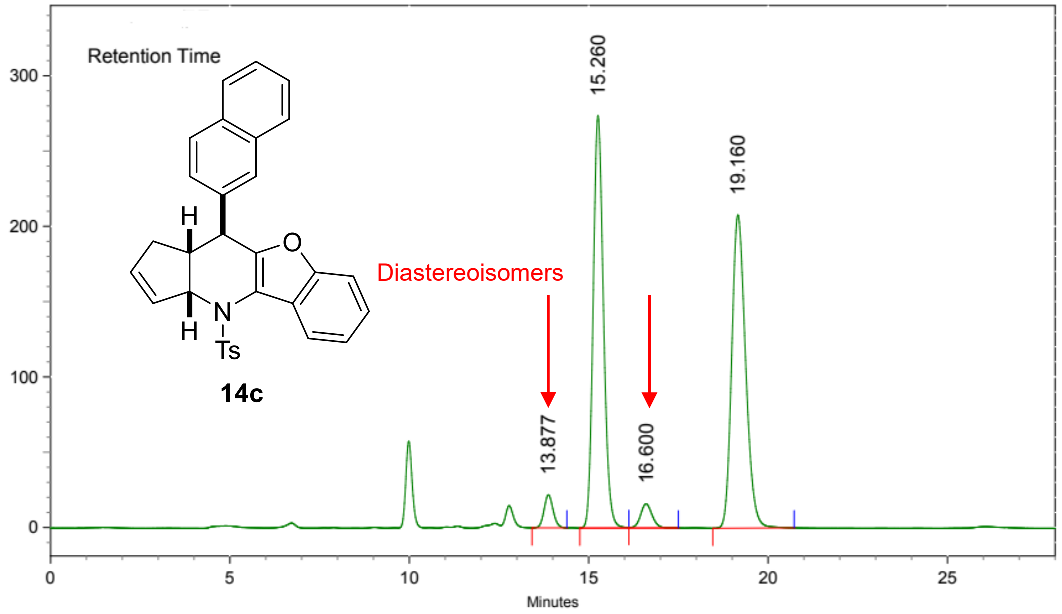

AREA PERCENT REPORT

Peak No.	Ret Time	Width	Height	Area	Area [%]
1	13.877	0.973	367474	6454178	3.2612
2	15.260	1.367	4594775	91634990	46.3023
3	16.600	1.380	268922	6026153	3.0450
4	19.160	2.267	3488554	93790732	47.3915

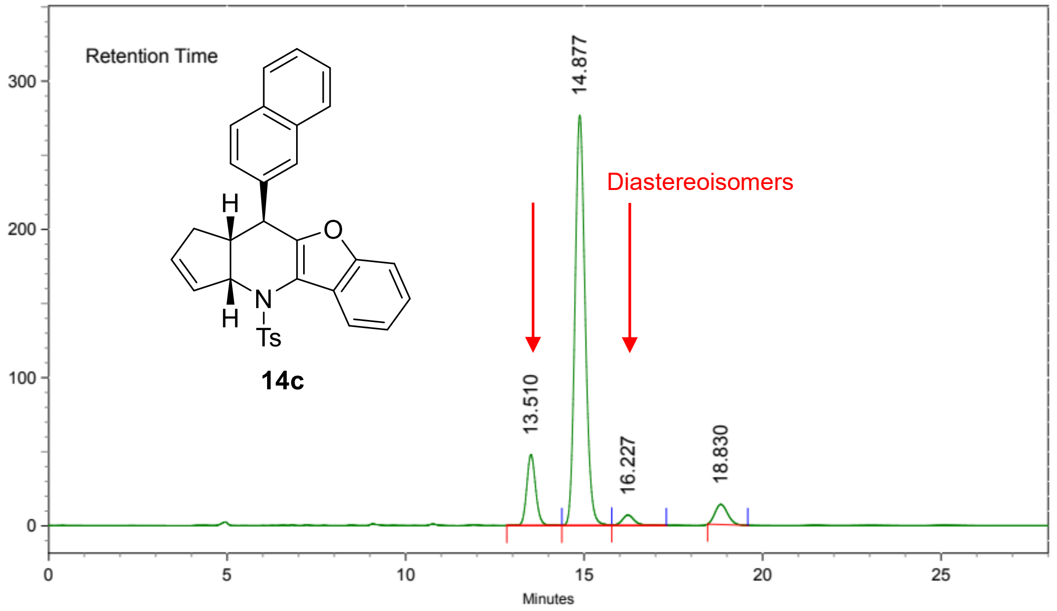

AREA PERCENT REPORT

Peak No.	Ret Time	Width	Height	Area	Area [%]
1	13.510	1.543	804048	14009941	12.2753
2	14.877	1.397	4643137	91635596	80.2897
3	16.227	1.530	118213	2872015	2.5164
4	18.830	1.127	227692	5613675	4.9186

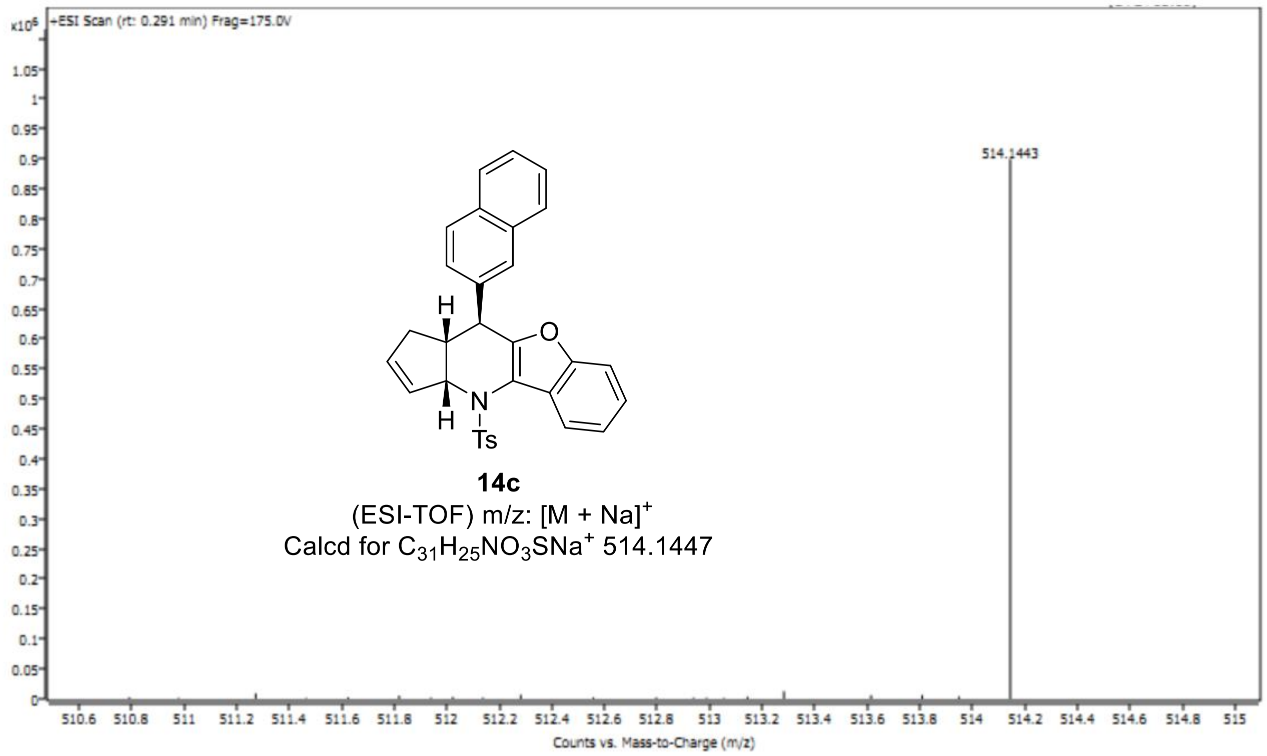
x10^6
+ESI Scan (rt: 0.291 min) Frag=175.0V
514.1443
H
O
N
H
Ts
14c
(ESI-TOF) m/z: [M + Na]$^+$
Calcd for $C_{31}H_{25}NO_3SNa^+$ 514.1447
Counts vs. Mass-to-Charge (m/z)

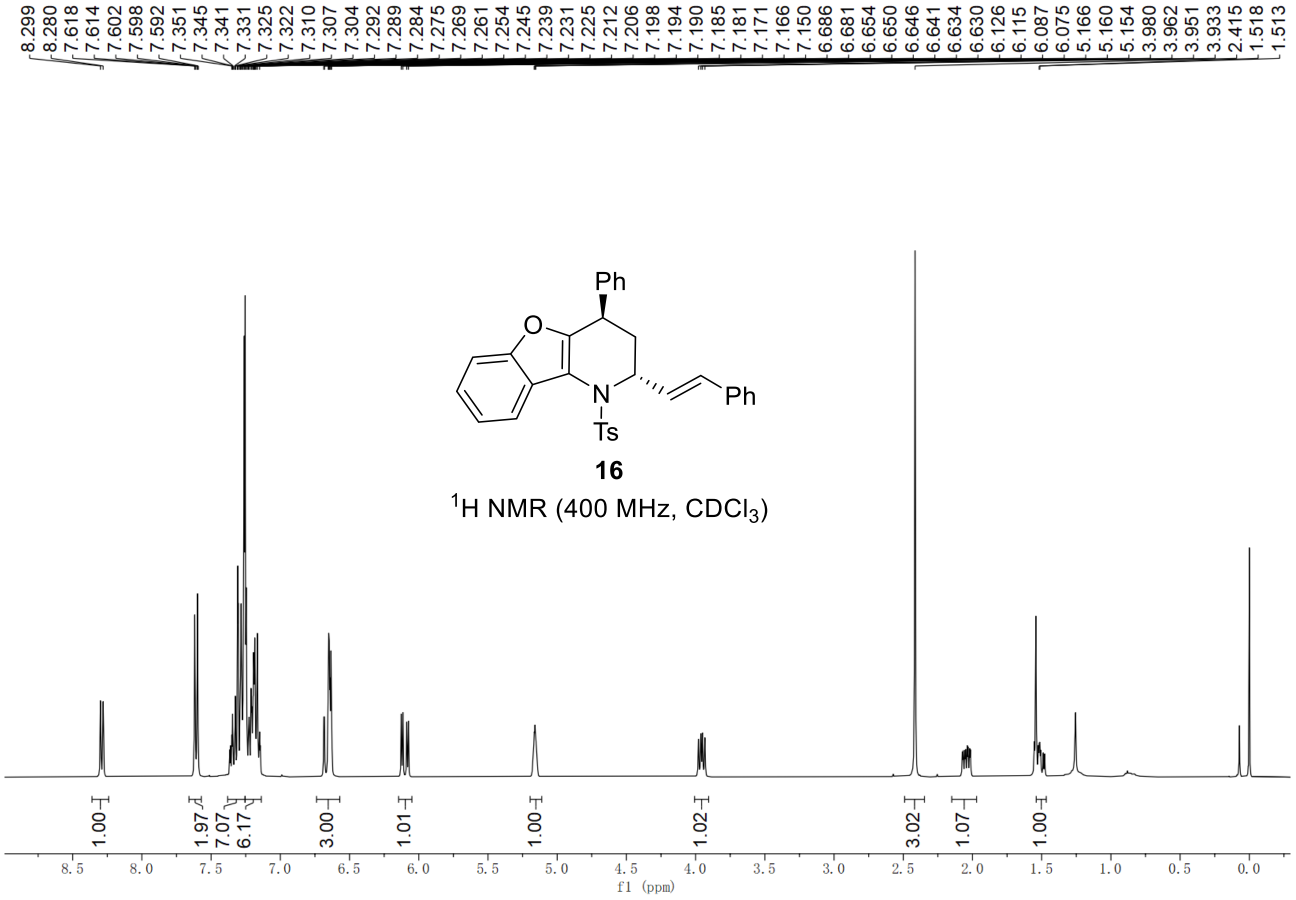
Ph
O
N
Ts
Ph
16
^{1}H NMR (400 MHz, $CDCl_3$)
f1 (ppm)

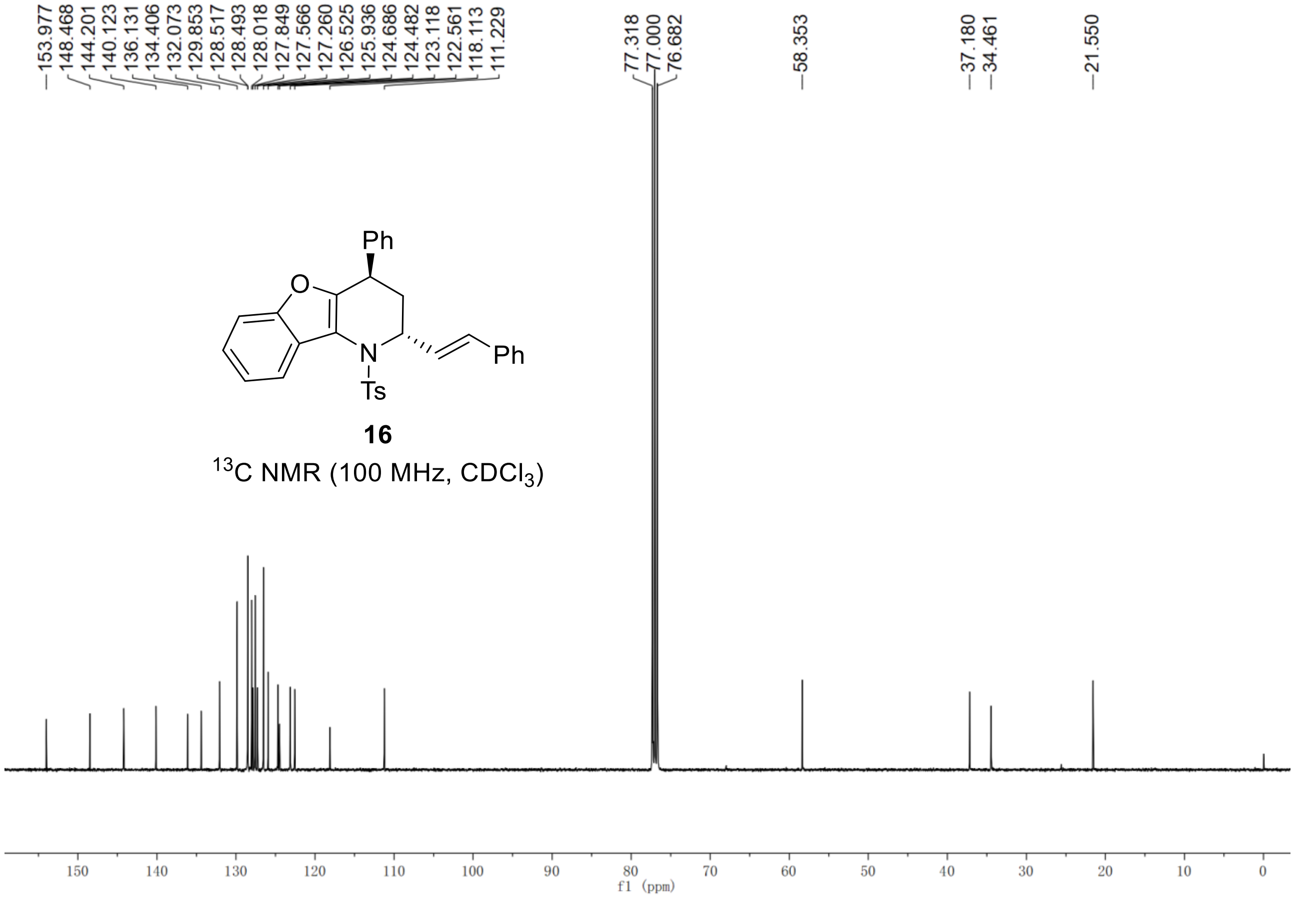
Ph
O
N
Ts
Ph
16
^{13}C NMR (100 MHz, $CDCl_3$)
f1 (ppm)

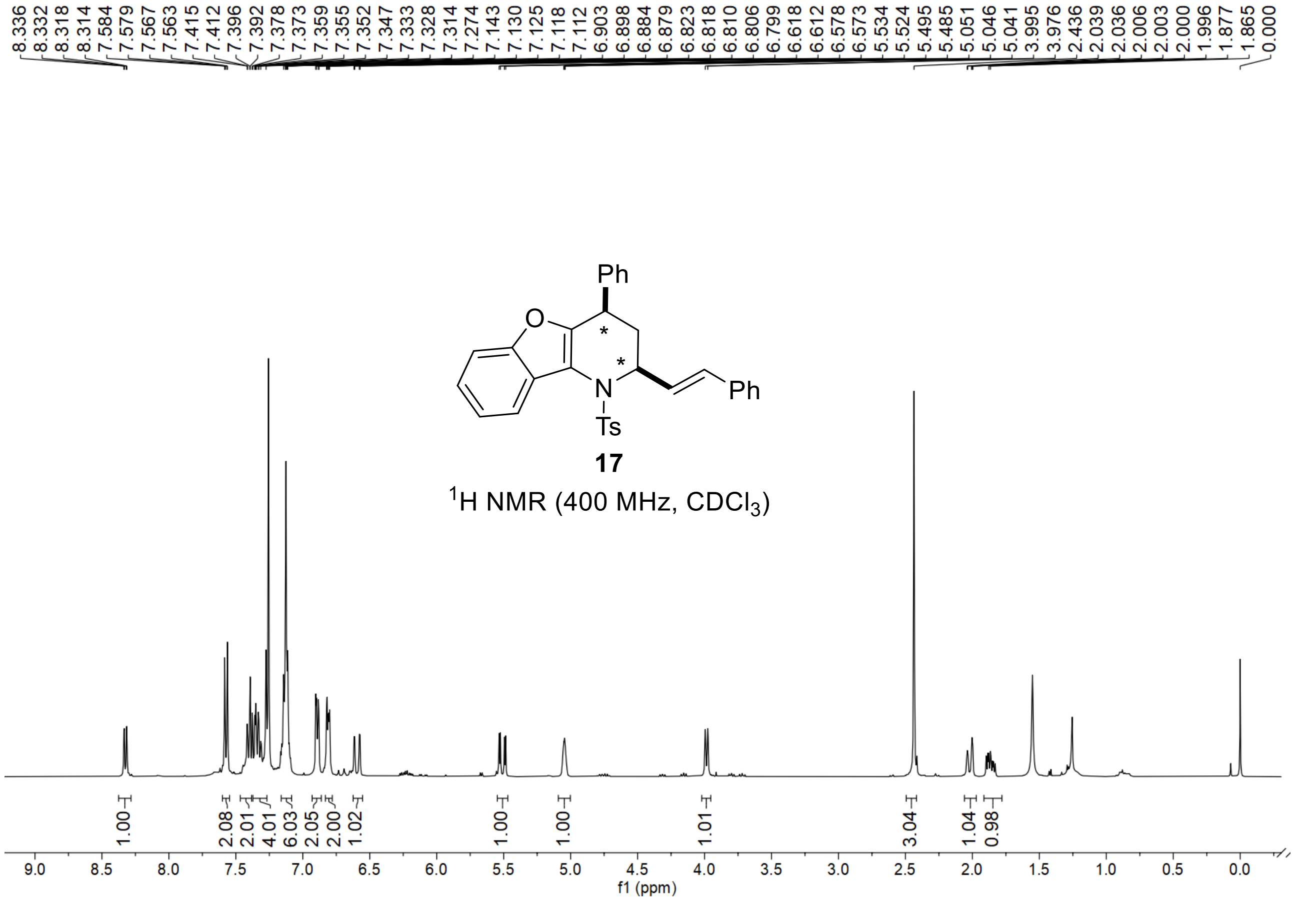
8.336 8.332 8.318 8.314 7.584 7.579 7.567 7.563 7.415 7.412 7.396 7.392 7.378 7.373 7.359 7.355 7.352 7.347 7.333 7.328 7.314 7.274 7.143 7.130 7.125 7.118 7.112 6.903 6.898 6.884 6.879 6.823 6.818 6.810 6.806 6.799 6.618 6.612 6.578 6.573 5.534 5.524 5.495 5.485 5.051 5.046 5.041 3.995 3.976 2.436 2.039 2.036 2.006 2.003 2.000 1.996 1.877 1.865 0.000
Ph
O
N
Ts
Ph
17
^{1}H NMR (400 MHz, CDCl$_3$)
1.00 2.08 2.01 4.01 6.03 2.05 2.00 1.02 1.00 1.00 1.01 3.04 1.04 0.98
f1 (ppm)

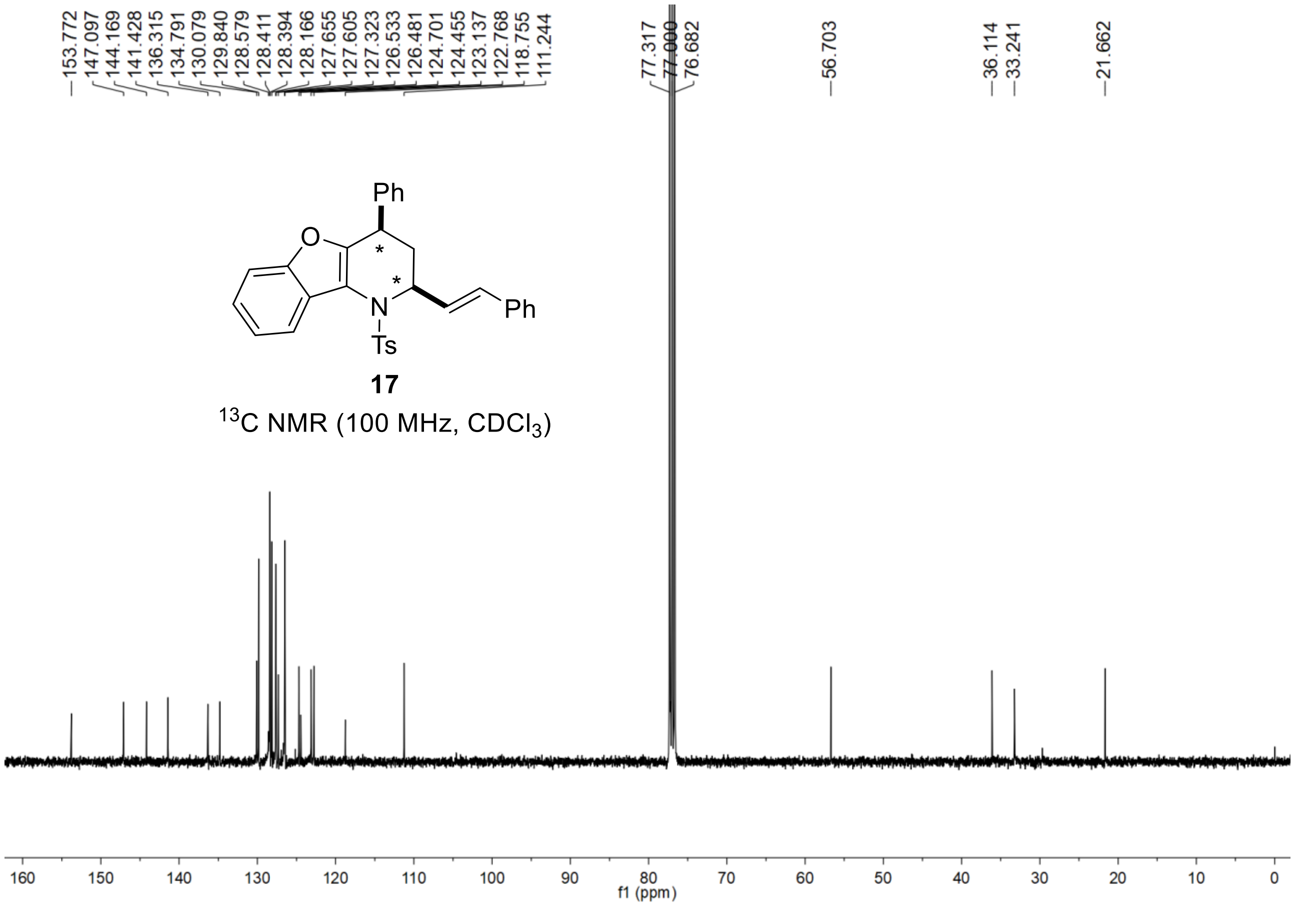
153.772 147.097 144.169 141.428 136.315 134.791 130.079 129.840 128.579 128.411 128.394 128.166 127.655 127.605 127.323 126.533 126.481 124.701 124.455 123.137 122.768 118.755 111.244
77.317 77.000 76.682
56.703
36.114 33.241
21.662
Ph
O
N
Ts
Ph
17
^{13}C NMR (100 MHz, CDCl$_3$)
f1 (ppm)

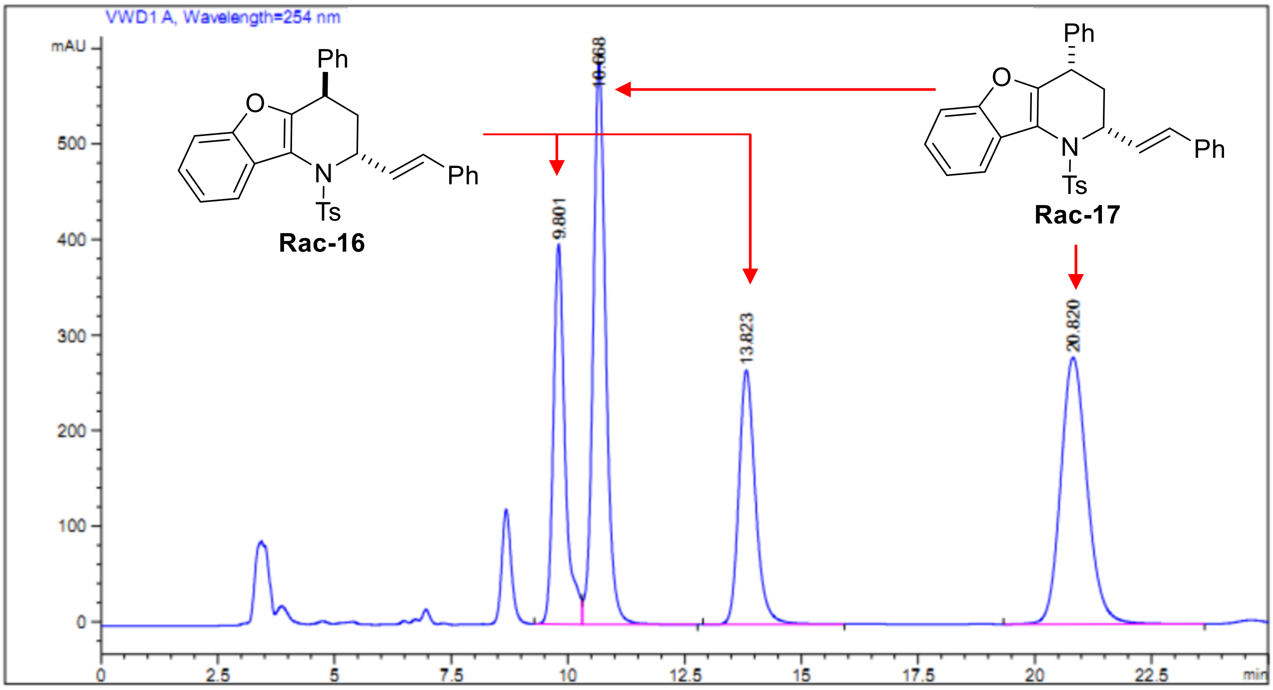

#	[min]		[min]	[mAU*s]	[mAU]	%
1	9.801	BV	0.2632	6980.00732	397.35217	19.5881
2	10.668	VB	0.2905	1.10772e4	585.84076	31.0861
3	13.823	BV	0.3827	6600.81689	266.02335	18.5239
4	20.820	BB	0.6042	1.09759e4	279.24176	30.8019

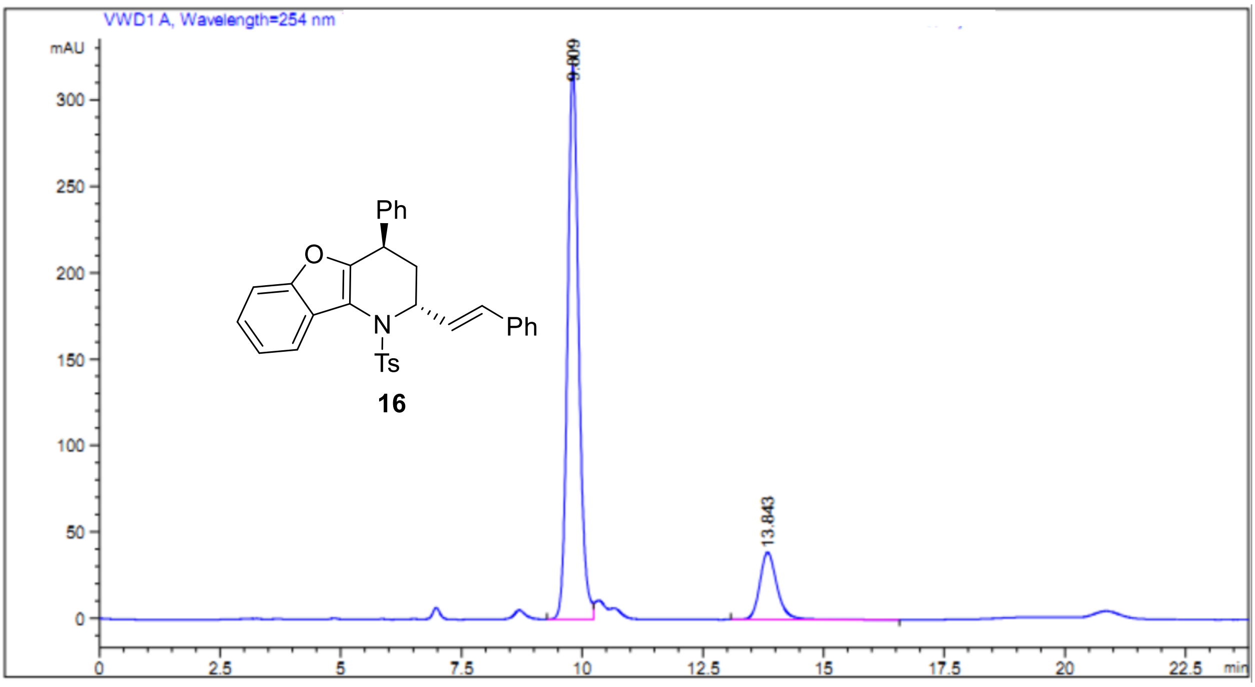

#	[min]		[min]	[mAU*s]	[mAU]	%
1	9.809	BV	0.2469	5171.30371	319.77383	84.2085
2	13.843	BB	0.3783	969.76636	38.89419	15.7915

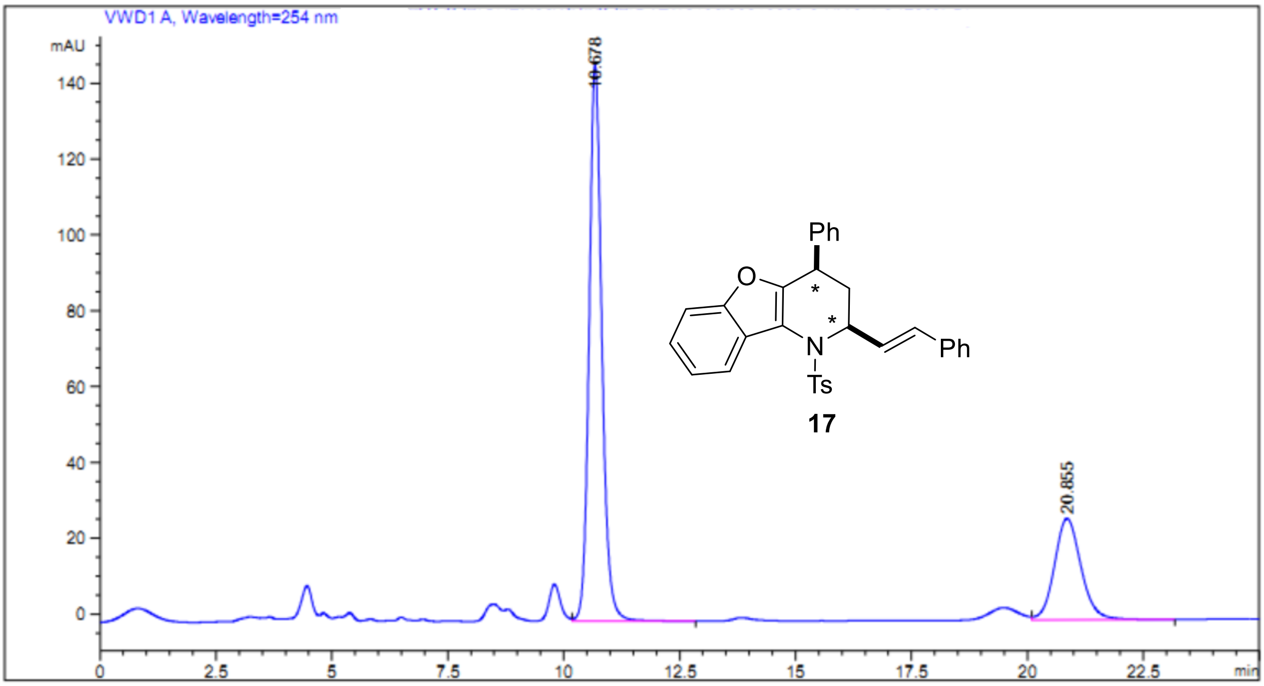

#	[min]		[min]	[mAU*s]	[mAU]	%
1	10.678	VB	0.2930	2803.35352	146.62024	72.3232
2	20.855	VB	0.6188	1072.79553	26.77868	27.6768

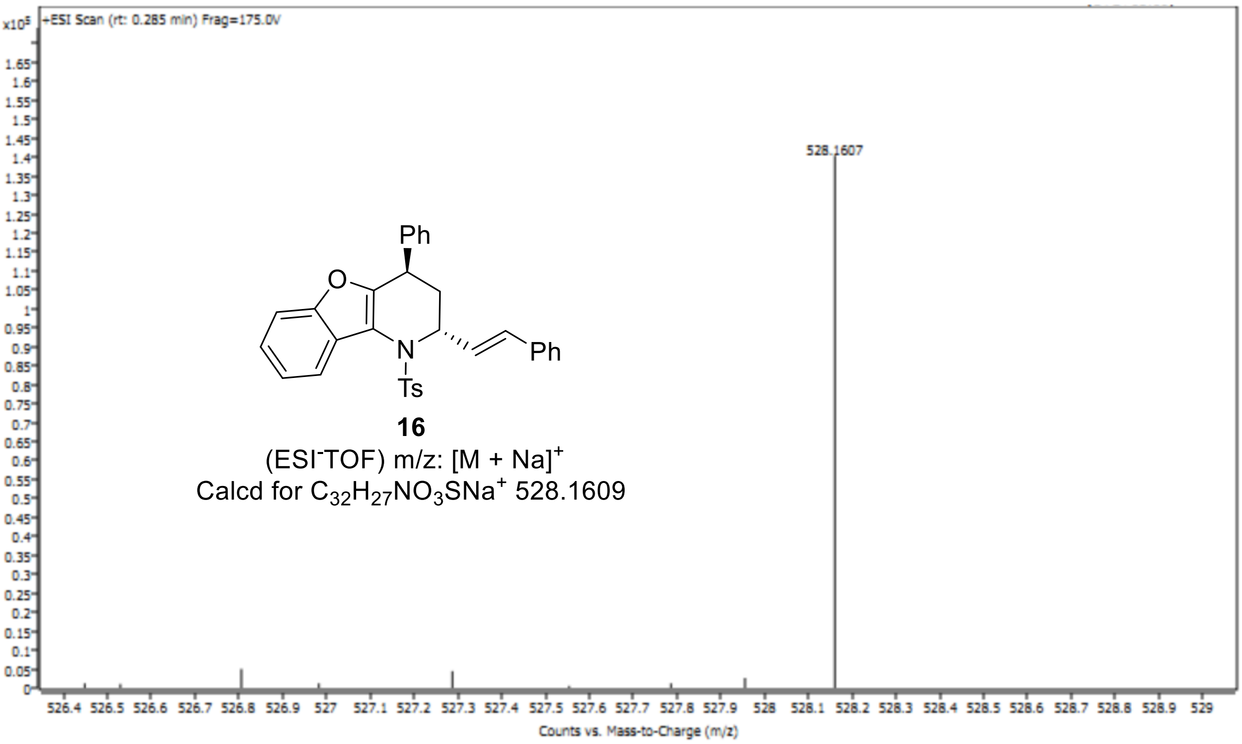

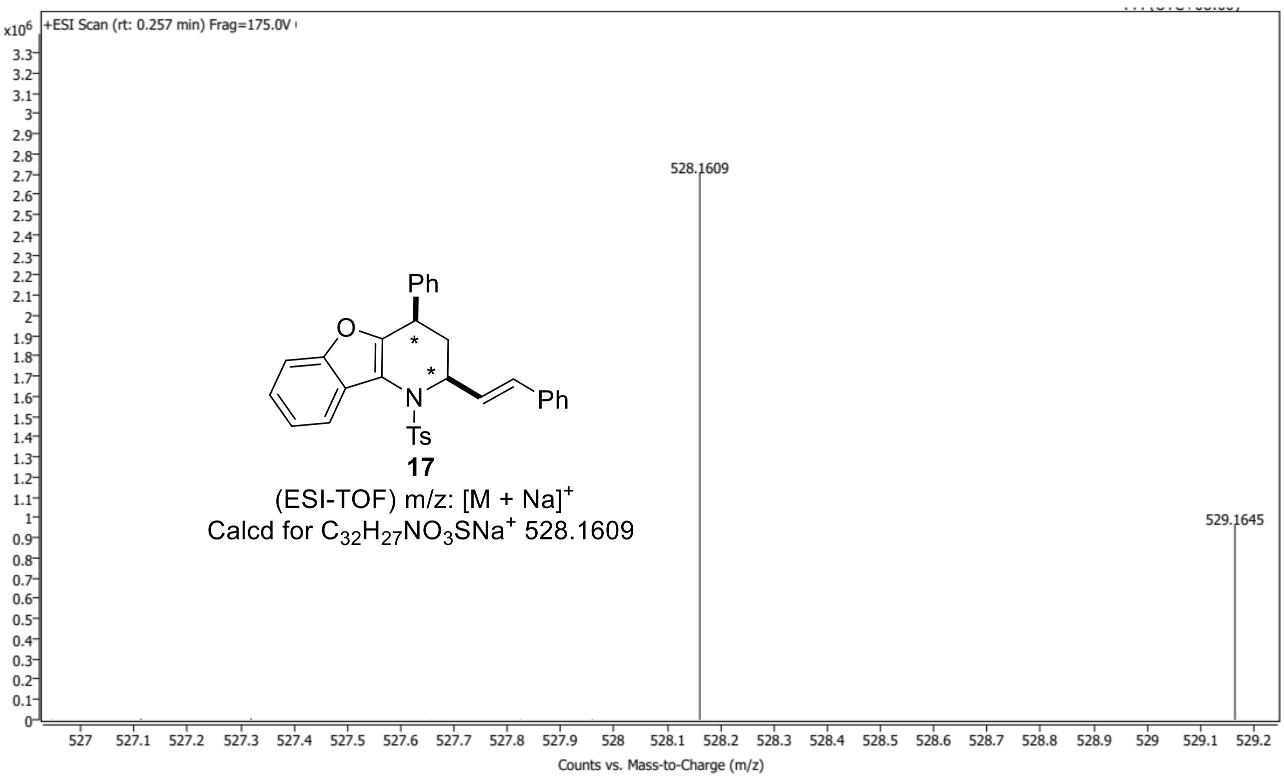
x10^6
+ESI Scan (rt: 0.257 min) Frag=175.0V
528.1609
529.1645
Ph
N
Ts
17
(ESI-TOF) m/z: [M + Na]+
Calcd for C32H27NO3SNa+ 528.1609
Counts vs. Mass-to-Charge (m/z)

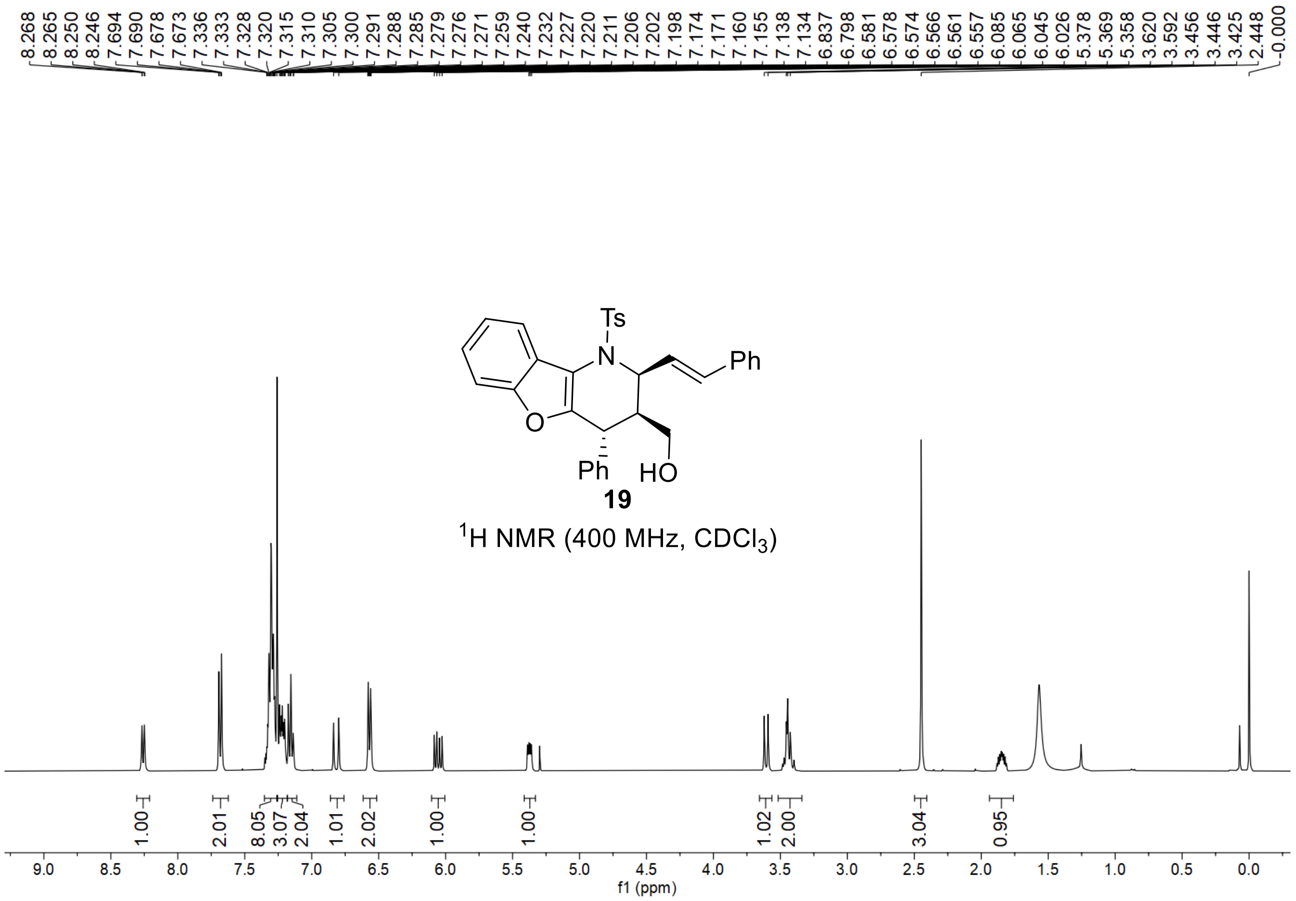
8.268
8.265
8.250
8.246
7.694
7.690
7.678
7.673
7.336
7.333
7.328
7.320
7.315
7.310
7.305
7.300
7.291
7.288
7.285
7.279
7.276
7.271
7.259
7.240
7.232
7.227
7.220
7.211
7.206
7.202
7.198
7.174
7.171
7.160
7.155
7.138
7.134
6.837
6.798
6.581
6.578
6.574
6.566
6.561
6.557
6.085
6.065
6.045
6.026
5.378
5.369
5.358
3.620
3.592
3.456
3.446
3.425
2.448
-0.000
Ts
N
Ph
O
Ph
HO
19
1H NMR (400 MHz, CDCl3)
1.00
2.01
8.05
3.07
2.04
1.01
2.02
1.00
1.00
1.02
2.00
3.04
0.95
f1 (ppm)

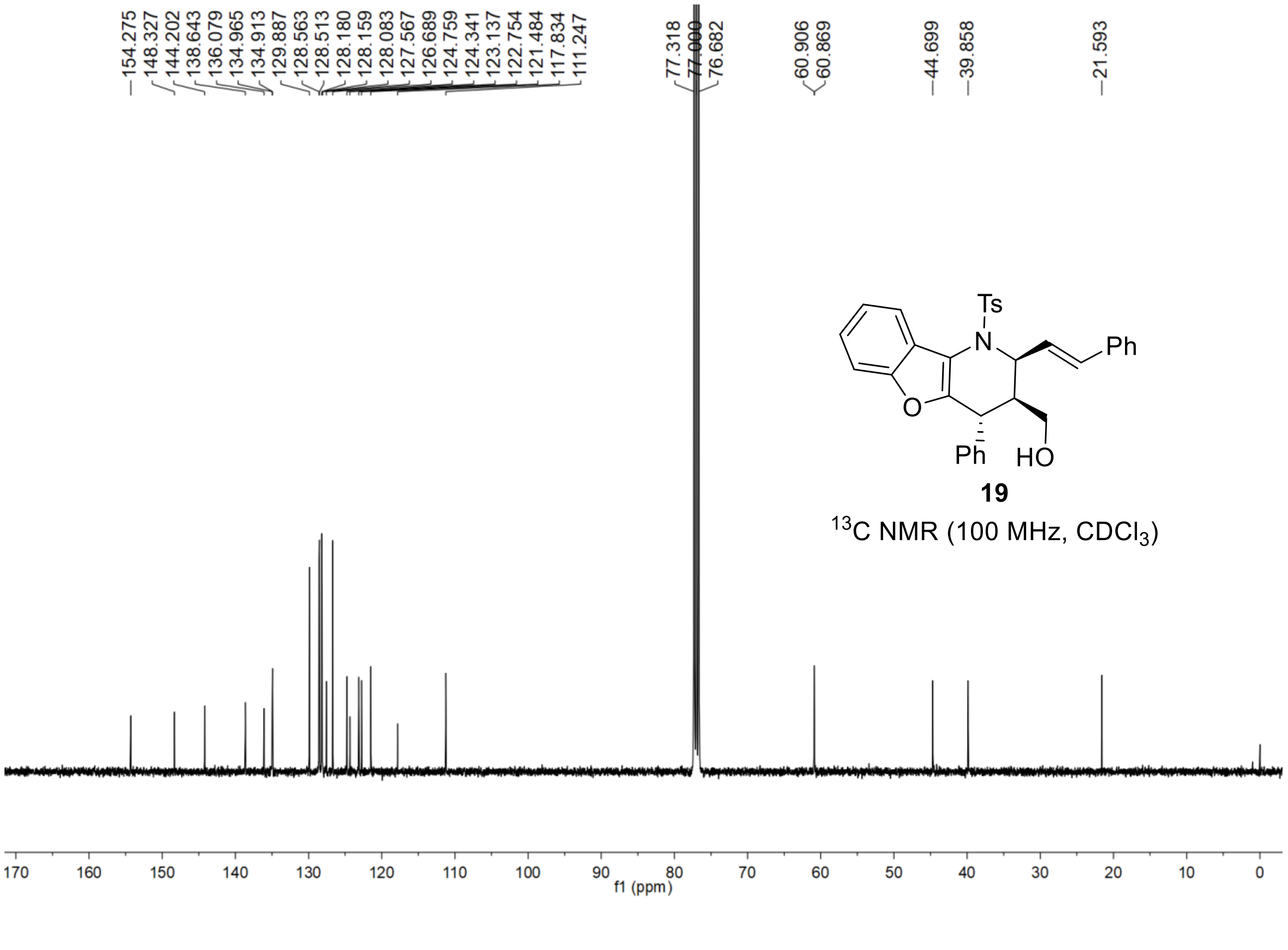
154.275
148.327
144.202
138.643
136.079
134.965
134.913
129.887
128.563
128.513
128.180
128.159
128.083
127.567
126.689
124.759
124.341
123.137
122.754
121.484
117.834
111.247
77.318
77.000
76.682
60.906
60.869
44.699
39.858
21.593
Ts
N
Ph
O
Ph
HO
19
13C NMR (100 MHz, CDCl3)
f1 (ppm)

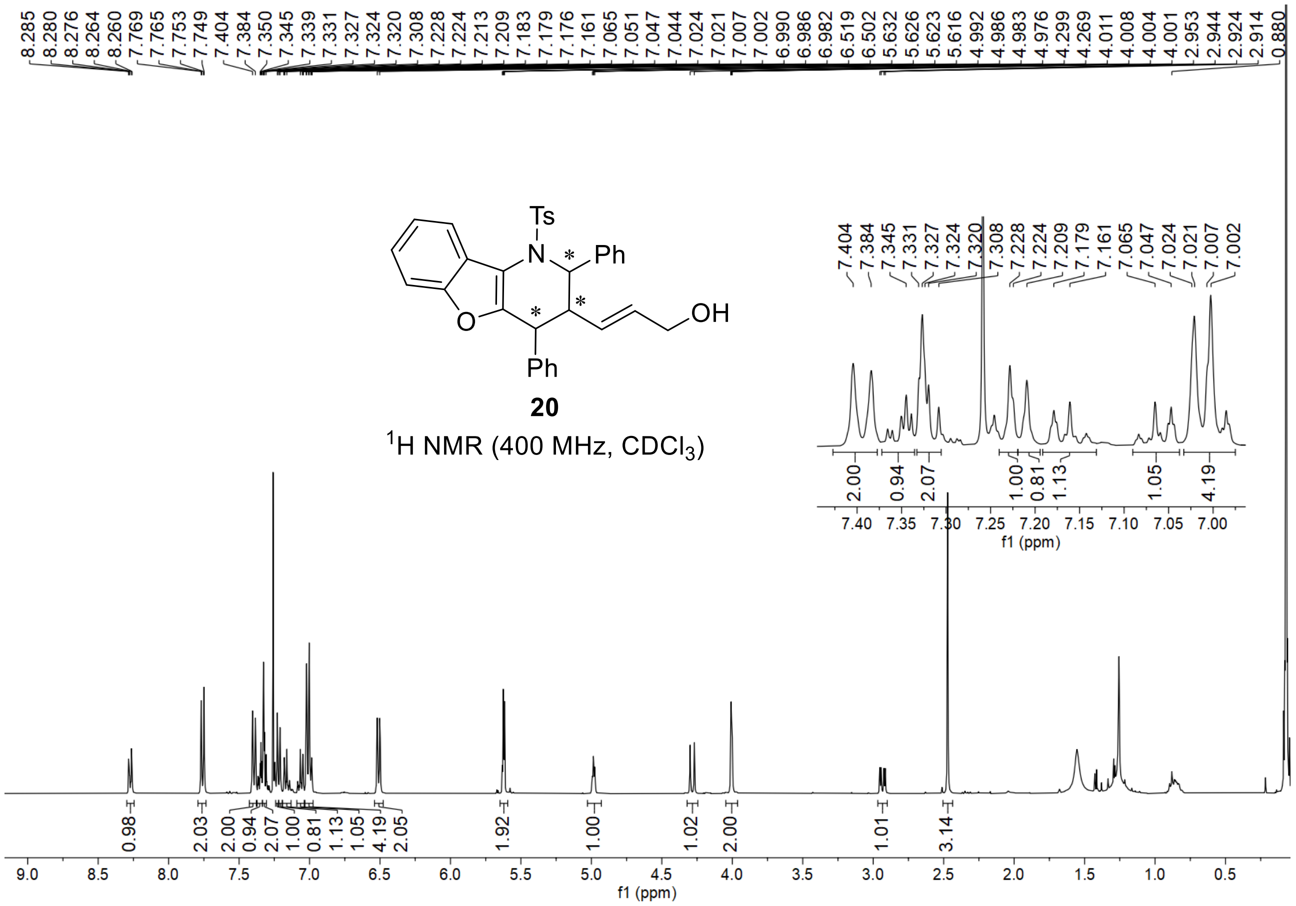
8.285 8.280 8.276 8.264 8.260 7.769 7.765 7.753 7.749 7.404 7.384 7.350 7.345 7.339 7.331 7.327 7.324 7.320 7.308 7.228 7.224 7.213 7.209 7.183 7.179 7.176 7.161 7.065 7.051 7.047 7.044 7.024 7.021 7.007 7.002 6.990 6.986 6.982 6.519 6.502 5.632 5.626 5.623 5.616 4.992 4.986 4.983 4.976 4.299 4.269 4.011 4.008 4.004 4.001 2.953 2.944 2.924 2.914 0.880
Ts
N
Ph
O
OH
Ph
20
1H NMR (400 MHz, CDCl3)
7.404 7.384 7.345 7.331 7.327 7.324 7.320 7.308 7.228 7.224 7.209 7.179 7.161 7.065 7.047 7.024 7.021 7.007 7.002
2.00 0.94 2.07 1.00 0.81 1.13 1.05 4.19
f1 (ppm)
0.98 2.03 2.00 0.94 2.07 1.00 0.81 1.13 1.05 4.19 2.05 1.92 1.00 1.02 2.00 1.01 3.14
f1 (ppm)

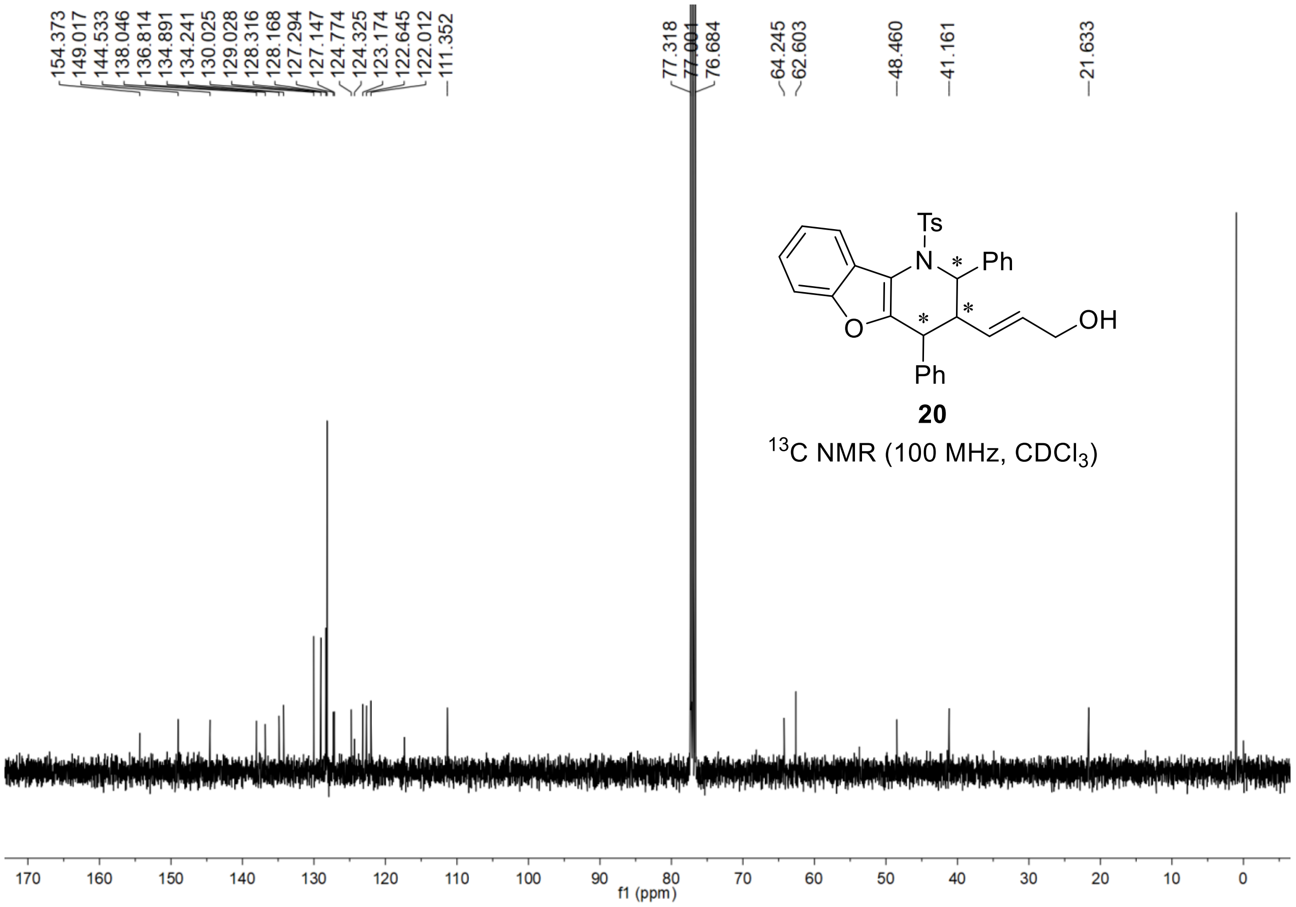
154.373 149.017 144.533 138.046 136.814 134.891 134.241 130.025 129.028 128.316 128.168 127.294 127.147 124.774 124.325 123.174 122.645 122.012 111.352
77.318 77.001 76.684
64.245 62.603
48.460
41.161
21.633
Ts
N
Ph
O
OH
Ph
20
13C NMR (100 MHz, CDCl3)
f1 (ppm)

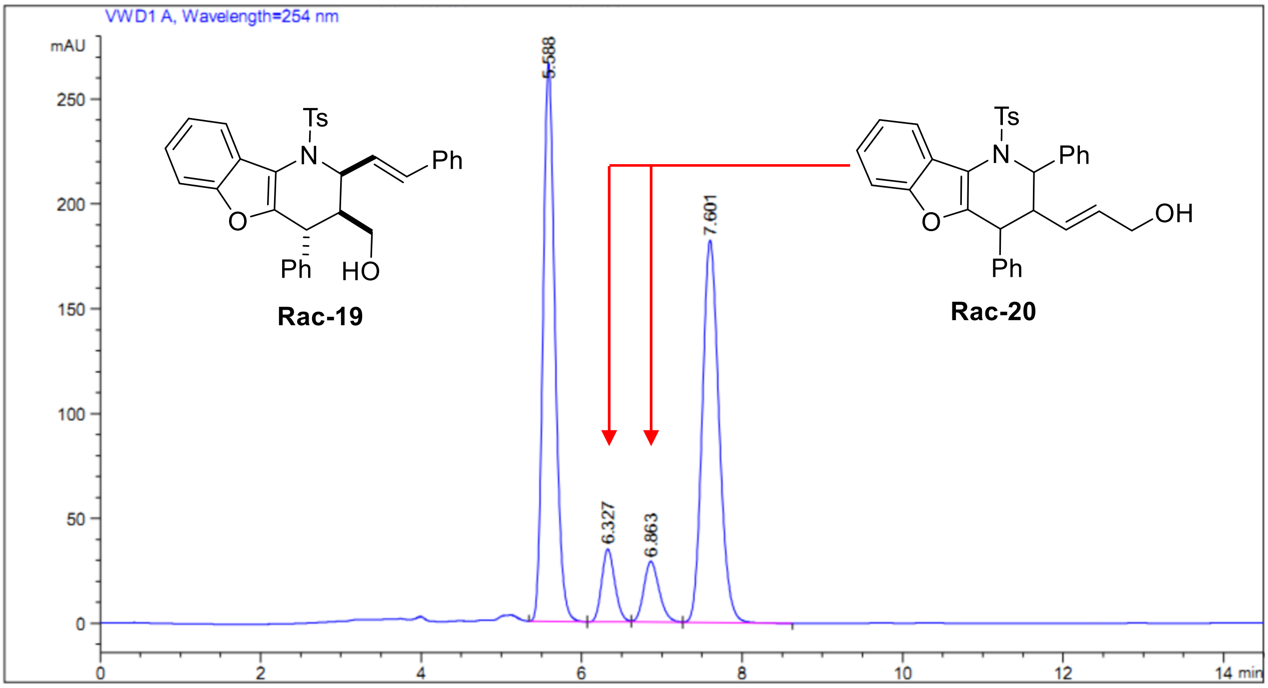

#	[min]		[min]	[mAU*s]	[mAU]	%
1	5.588	BB	0.1605	2764.00415	266.02731	44.6367
2	6.327	BV	0.1775	398.03018	34.69069	6.4279
3	6.863	VB	0.2047	384.44531	28.90234	6.2085
4	7.601	BB	0.2246	2645.74878	182.30461	42.7269

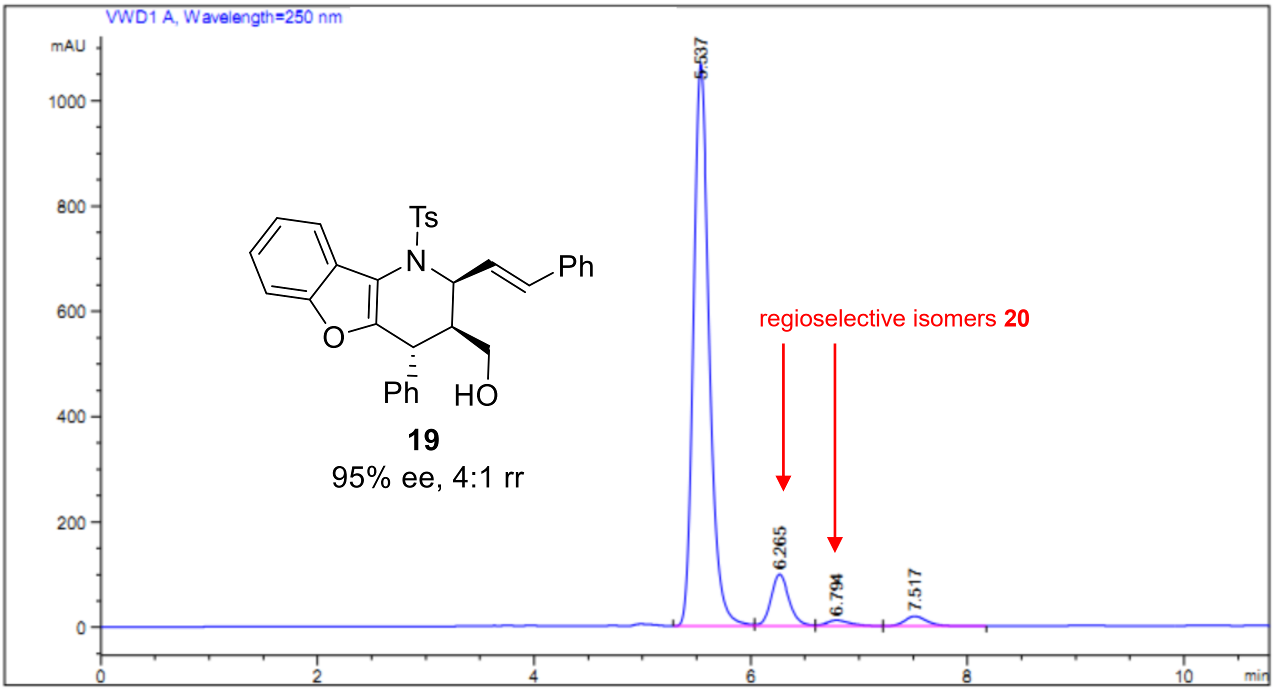

#	[min]		[min]	[mAU*s]	[mAU]	%
1	5.537	BV	0.1570	1.08929e4	1067.08411	87.3761
2	6.265	VV	0.1777	1143.10168	98.41490	9.1693
3	6.794	VB	0.2235	165.83662	11.02894	1.3302
4	7.517	BB	0.2234	264.83557	18.38567	2.1244

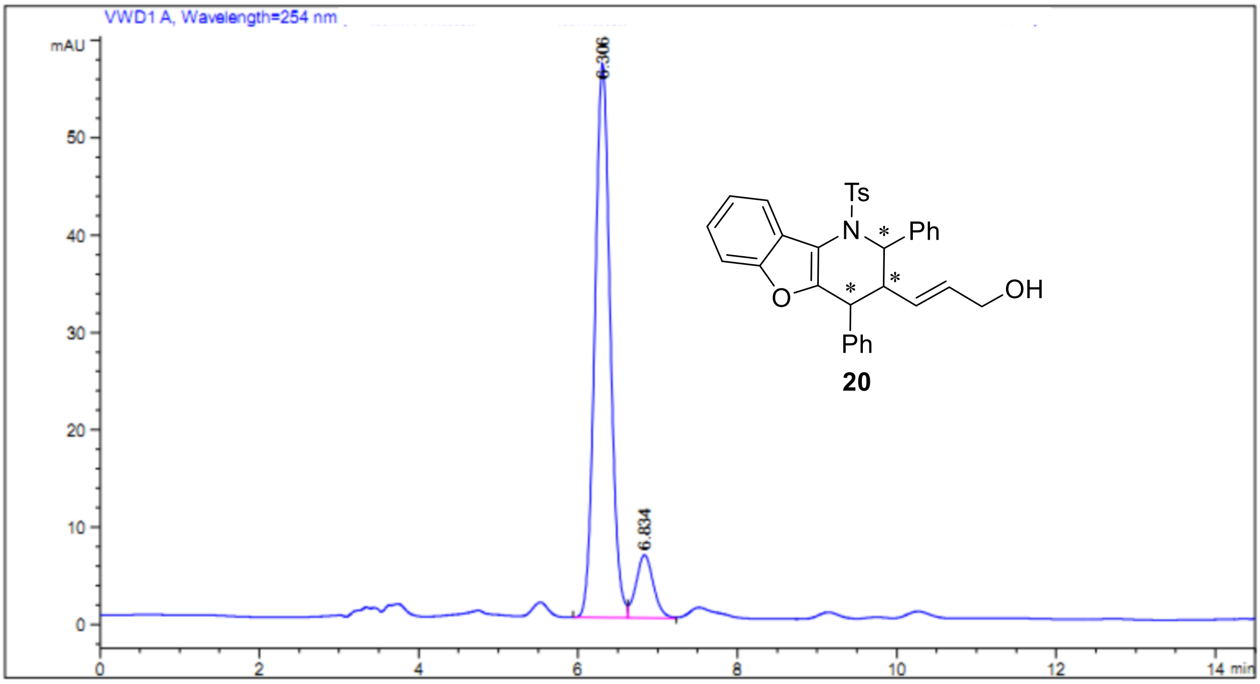

#	[min]		[min]	[mAU*s]	[mAU]	%
1	6.306	BV	0.2059	762.58545	56.88541	88.6225
2	6.834	VV	0.2318	97.90224	6.47265	11.3775

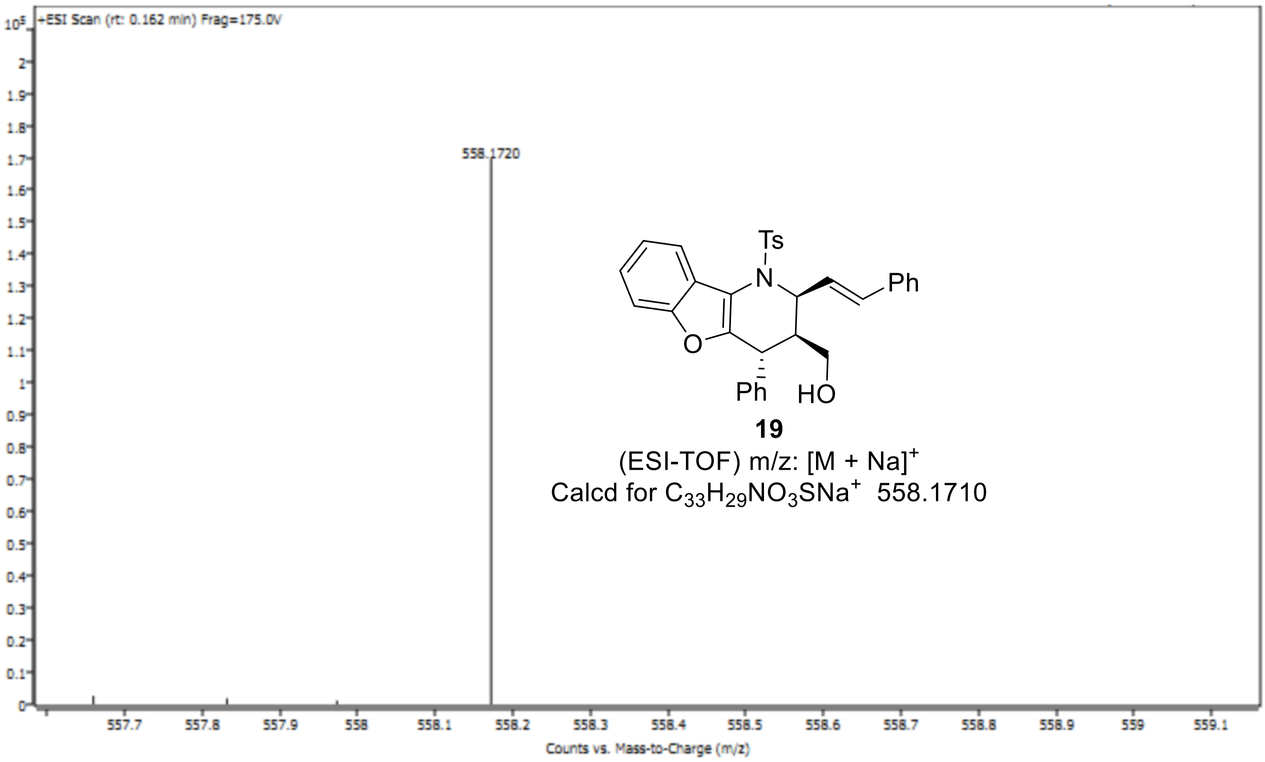

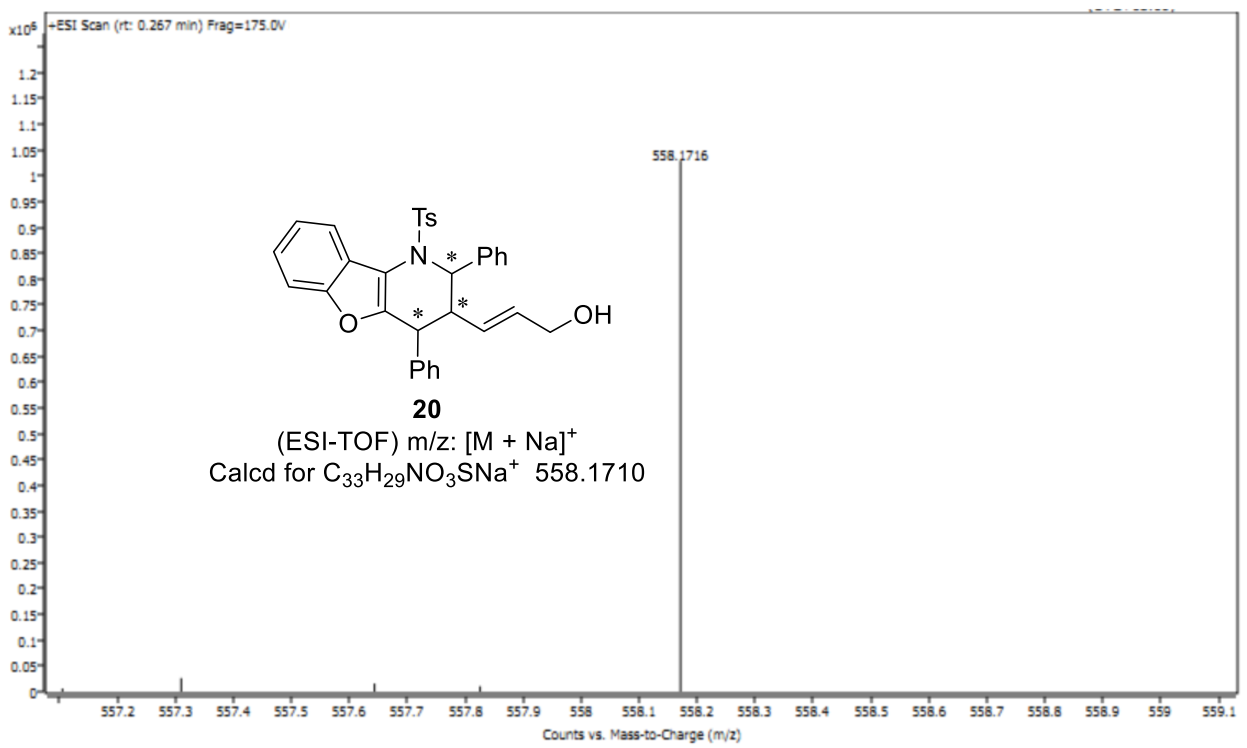
x10^6
+ESI Scan (rt: 0.267 min) Frag=175.0V
558.1716
Ts
N
Ph
O
OH
Ph
20
(ESI-TOF) m/z: [M + Na]+
Calcd for C33H29NO3SNa+ 558.1710
Counts vs. Mass-to-Charge (m/z)

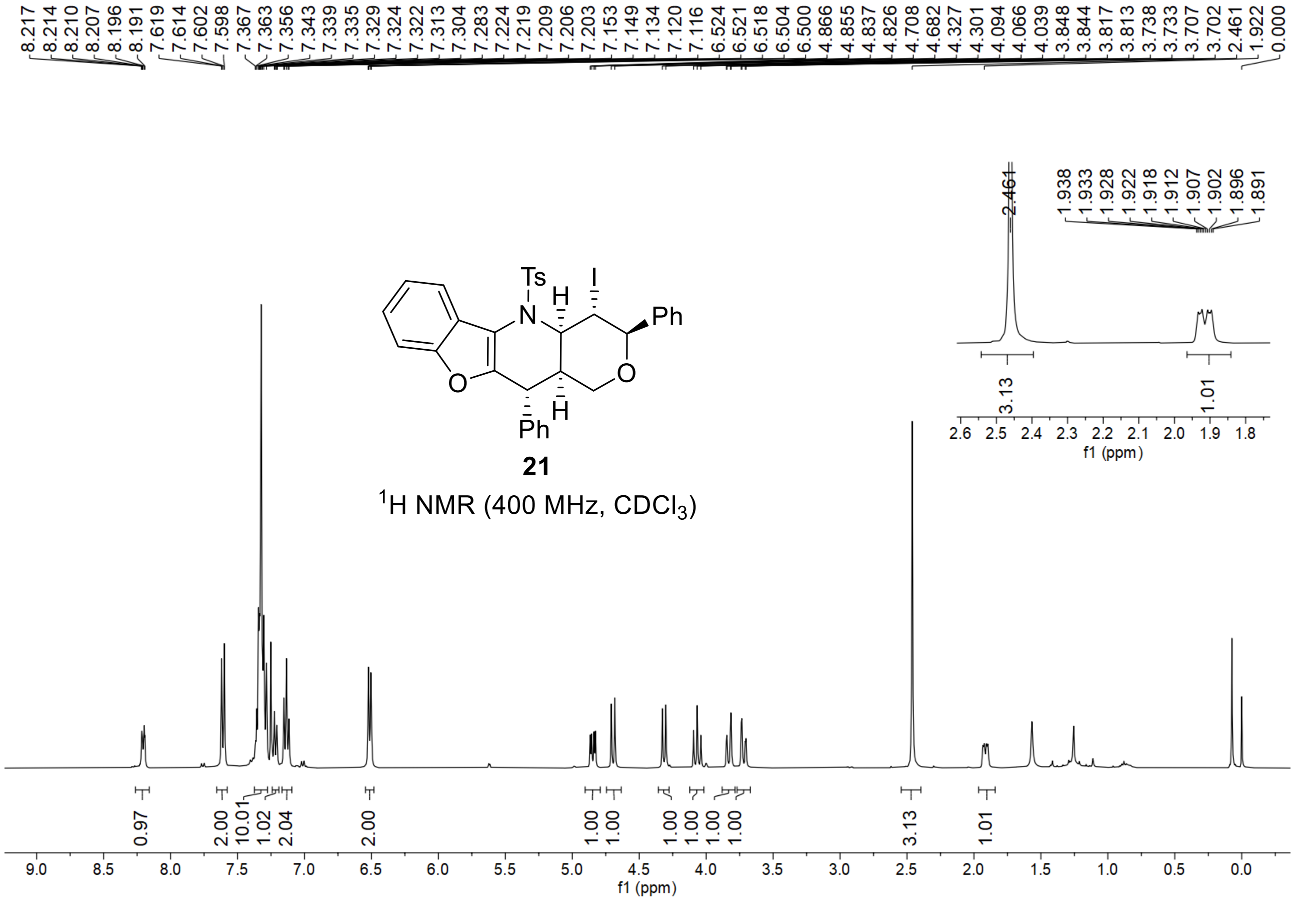

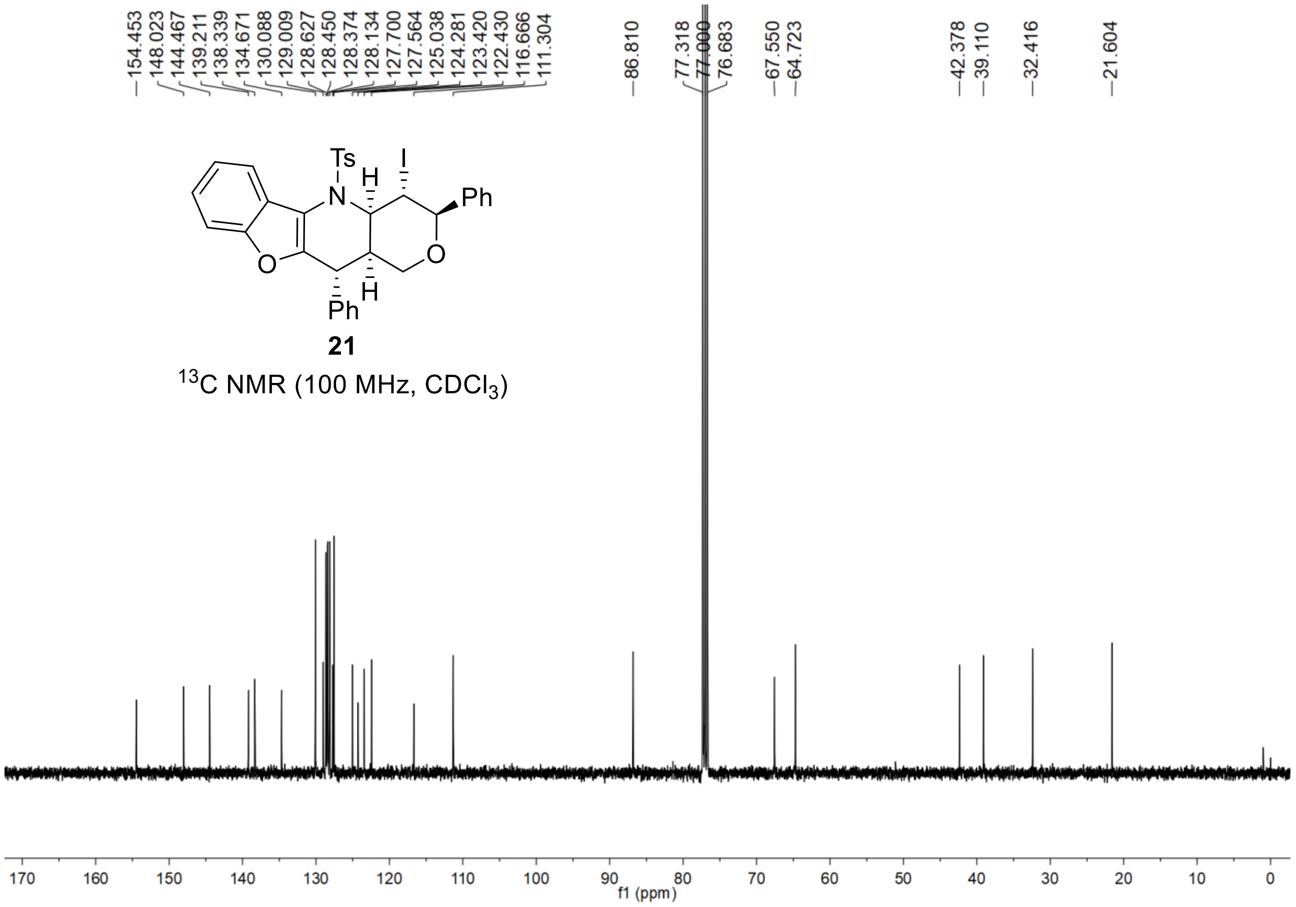

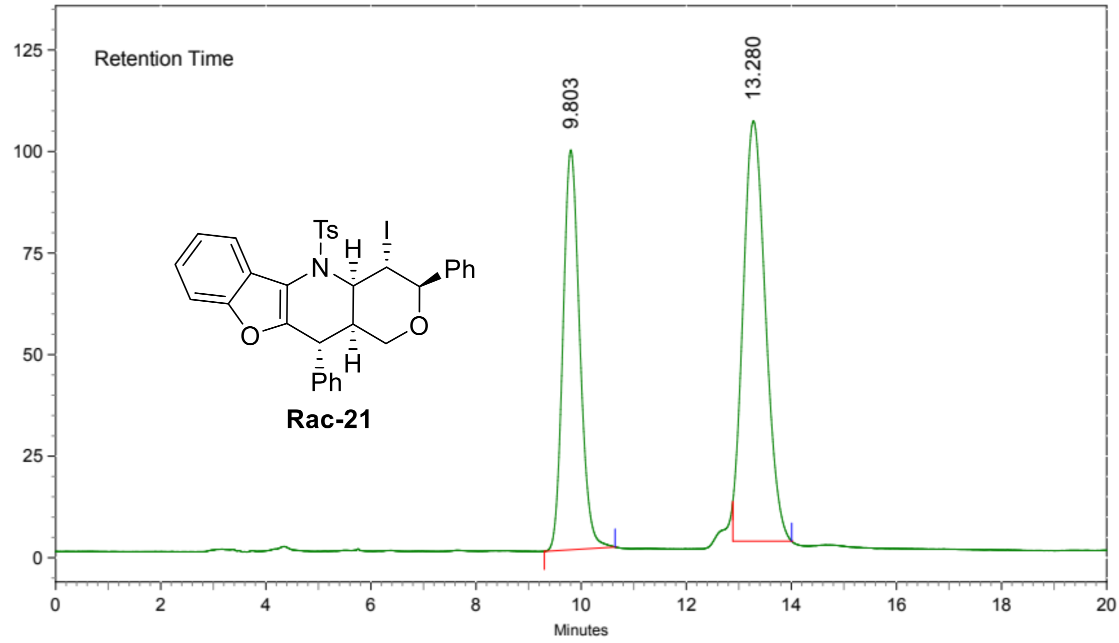

AREA PERCENT REPORT

Peak No.	Ret Time	Width	Height	Area	Area [%]
1	9.803	1.347	1650892	36488077	41.5486
2	13.280	1.120	1735418	51332240	58.4514

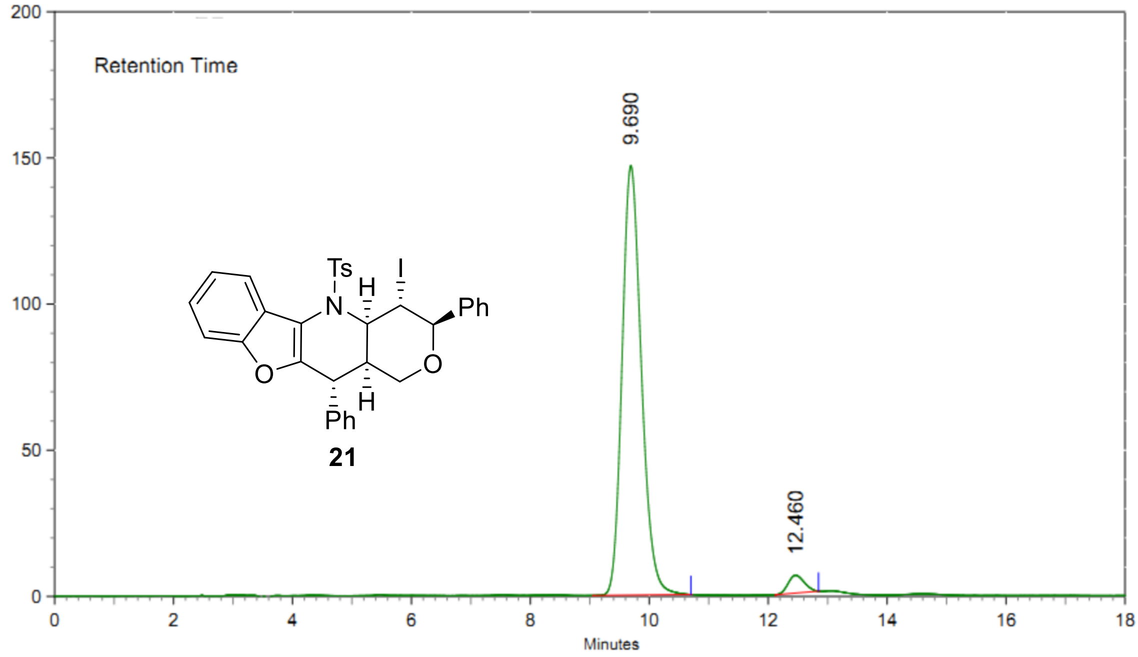

AREA PERCENT REPORT

Peak No.	Ret Time	Width	Height	Area	Area [%]
1	9.690	1.647	2464746	55543182	96.6446
2	12.460	0.727	102311	1928396	3.3554

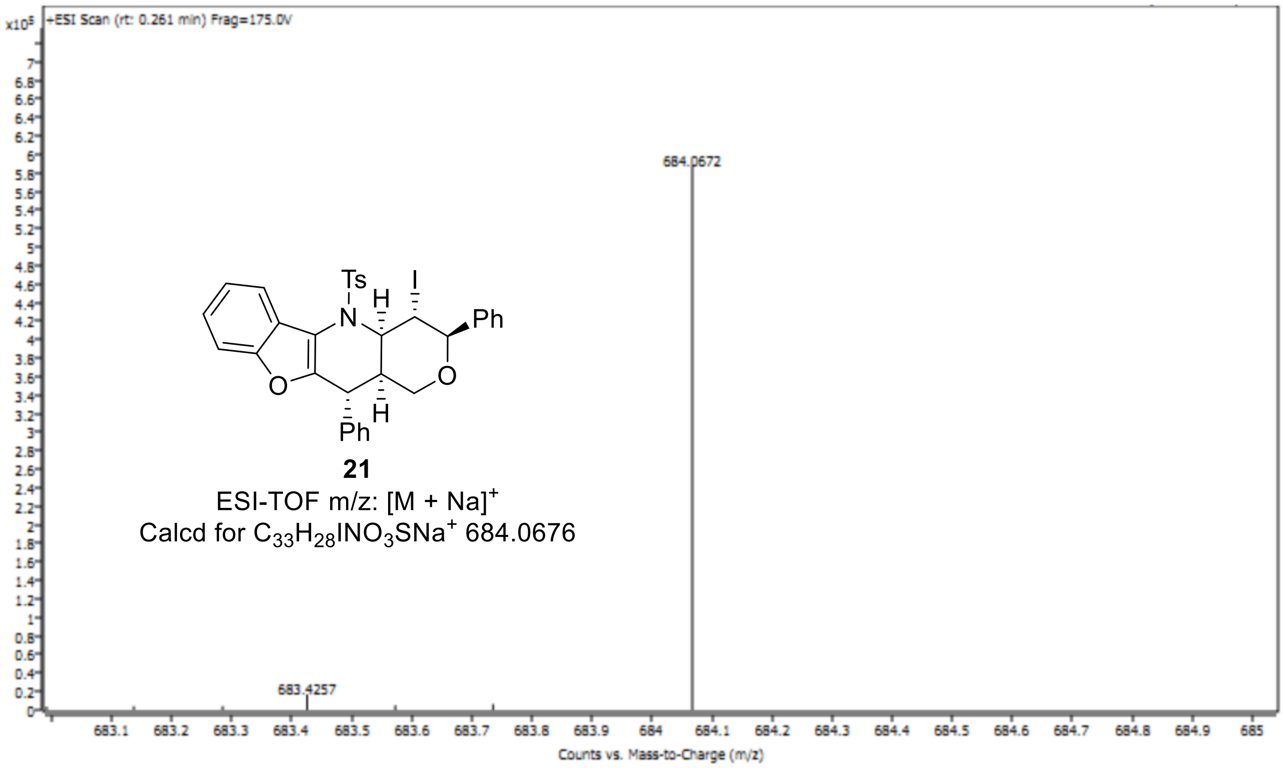
x10^5 +ESI Scan (rt: 0.261 min) Frag=175.0V
684.0672
683.4257
Ts
N
H
I
Ph
O
O
H
Ph
21
ESI-TOF m/z: [M + Na]+
Calcd for C33H28INO3SNa+ 684.0676
Counts vs. Mass-to-Charge (m/z)

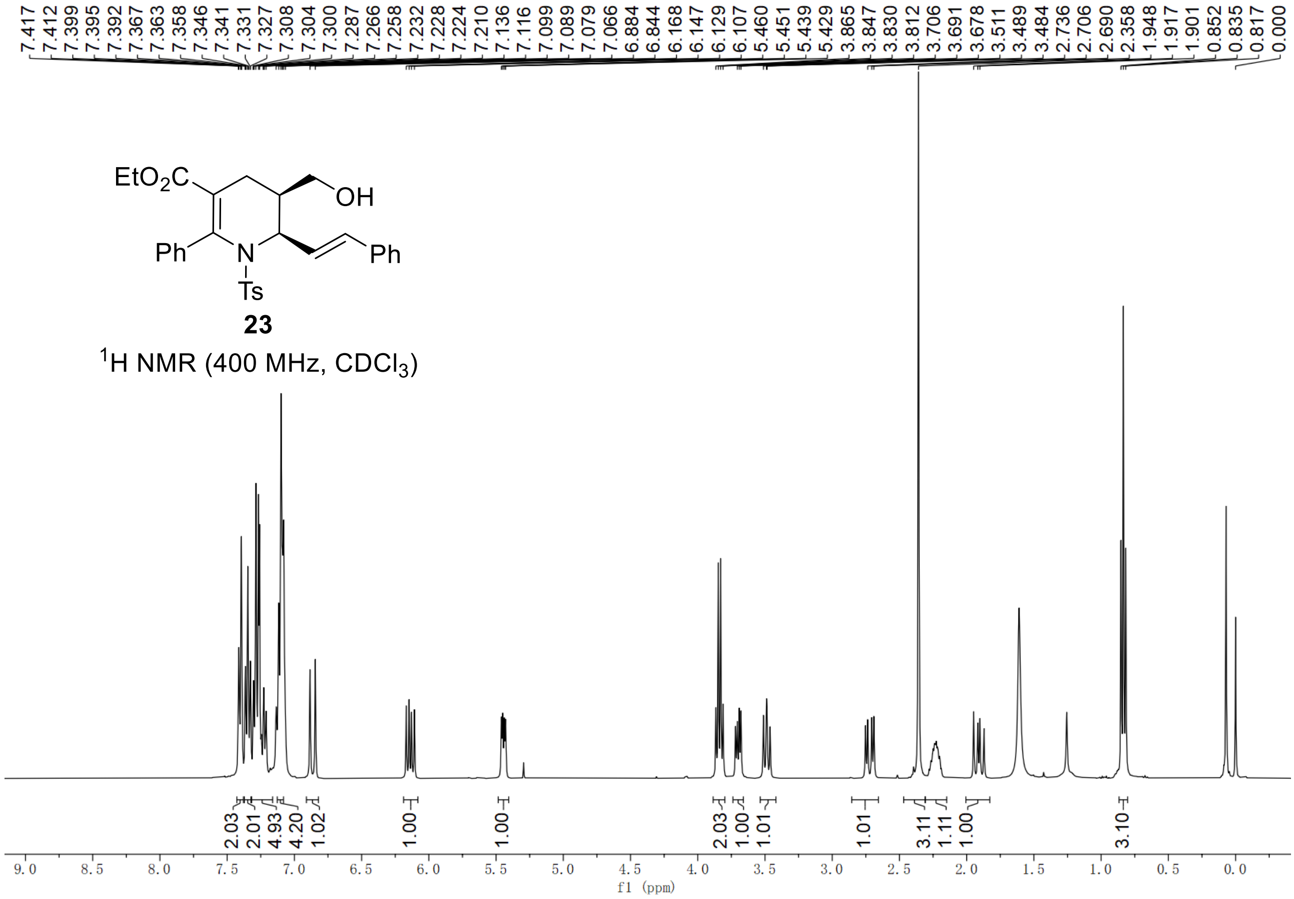

7.417 7.412 7.399 7.395 7.392 7.367 7.363 7.358 7.346 7.341 7.331 7.327 7.308 7.304 7.300 7.287 7.266 7.258 7.232 7.228 7.224 7.210 7.136 7.116 7.099 7.089 7.079 7.066 6.884 6.844 6.168 6.147 6.129 6.107 5.460 5.451 5.439 5.429 3.865 3.847 3.830 3.812 3.706 3.691 3.678 3.511 3.489 3.484 2.736 2.706 2.690 2.358 1.948 1.917 1.901 0.852 0.835 0.817 0.000
EtO2C
OH
Ph
N
Ph
Ts
23
1H NMR (400 MHz, CDCl3)
2.03 2.01 4.93 4.20 1.02 1.00 1.00 2.03 1.00 1.01 1.01 3.11 1.11 1.00 3.10
9.0 8.5 8.0 7.5 7.0 6.5 6.0 5.5 5.0 4.5 4.0 3.5 3.0 2.5 2.0 1.5 1.0 0.5 0.0
f1 (ppm)

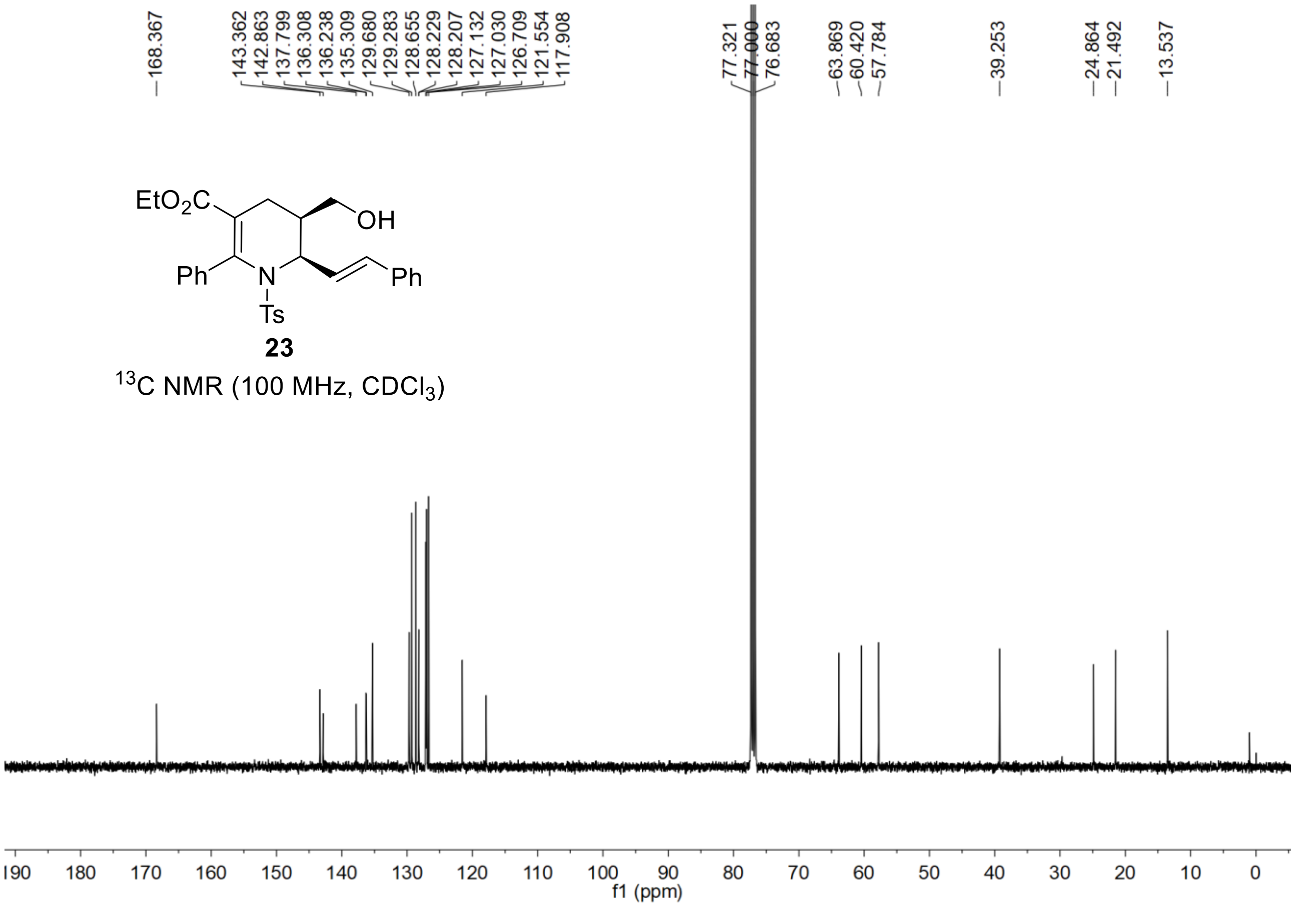

168.367
143.362 142.863 137.799 136.308 136.238 135.309 129.680 129.283 128.655 128.229 128.207 127.132 127.030 126.709 121.554 117.908
77.321 77.000 76.683
63.869 60.420 57.784
39.253
24.864 21.492
13.537
EtO2C
OH
Ph
N
Ph
Ts
23
13C NMR (100 MHz, CDCl3)
190 180 170 160 150 140 130 120 110 100 90 80 70 60 50 40 30 20 10 0
f1 (ppm)

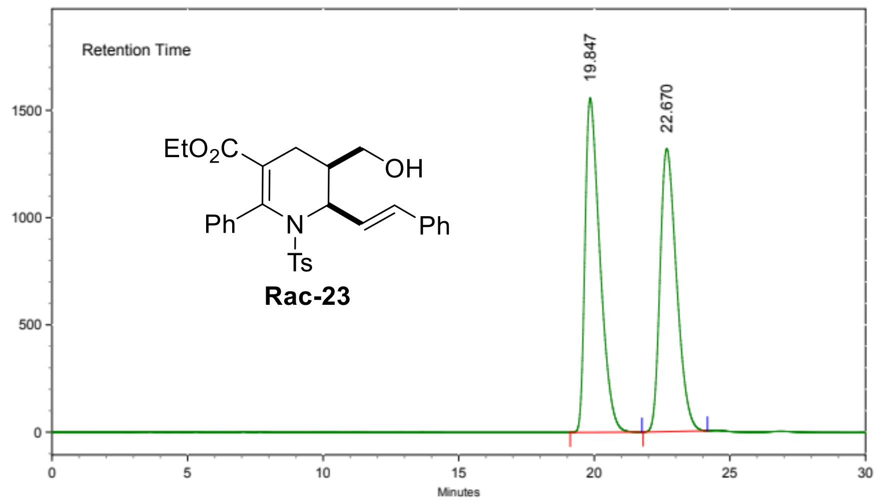

AREA PERCENT REPORT

Peak No.	Ret Time	Width	Height	Area	Area [%]
1	19.847	2.647	26140923	997199617	51.5302
2	22.670	2.373	22125655	937976107	48.4698

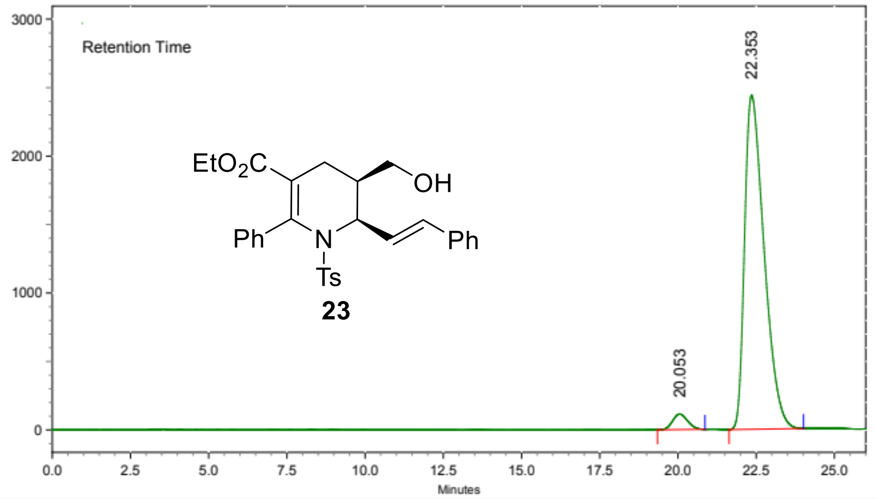

AREA PERCENT REPORT

Peak No.	Ret Time	Width	Height	Area	Area [%]
1	20.053	1.520	1904713	63455617	3.4477
2	22.353	2.380	40920672	1777076269	96.5523

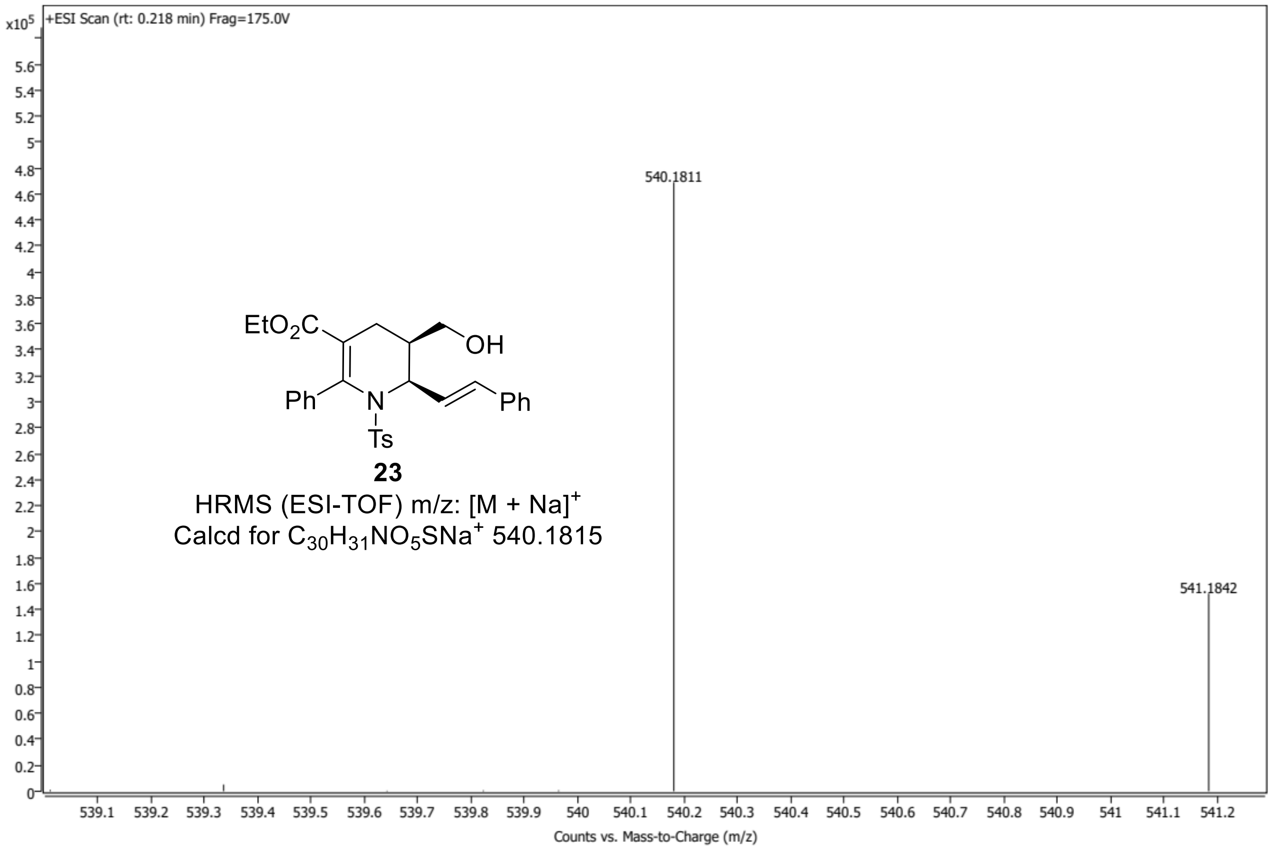
+ESI Scan (rt: 0.218 min) Frag=175.0V
x10^5
540.1811
541.1842
EtO2C
OH
Ph
N
Ph
Ts
23
HRMS (ESI-TOF) m/z: [M + Na]+
Calcd for C30H31NO5SNa+ 540.1815
Counts vs. Mass-to-Charge (m/z)

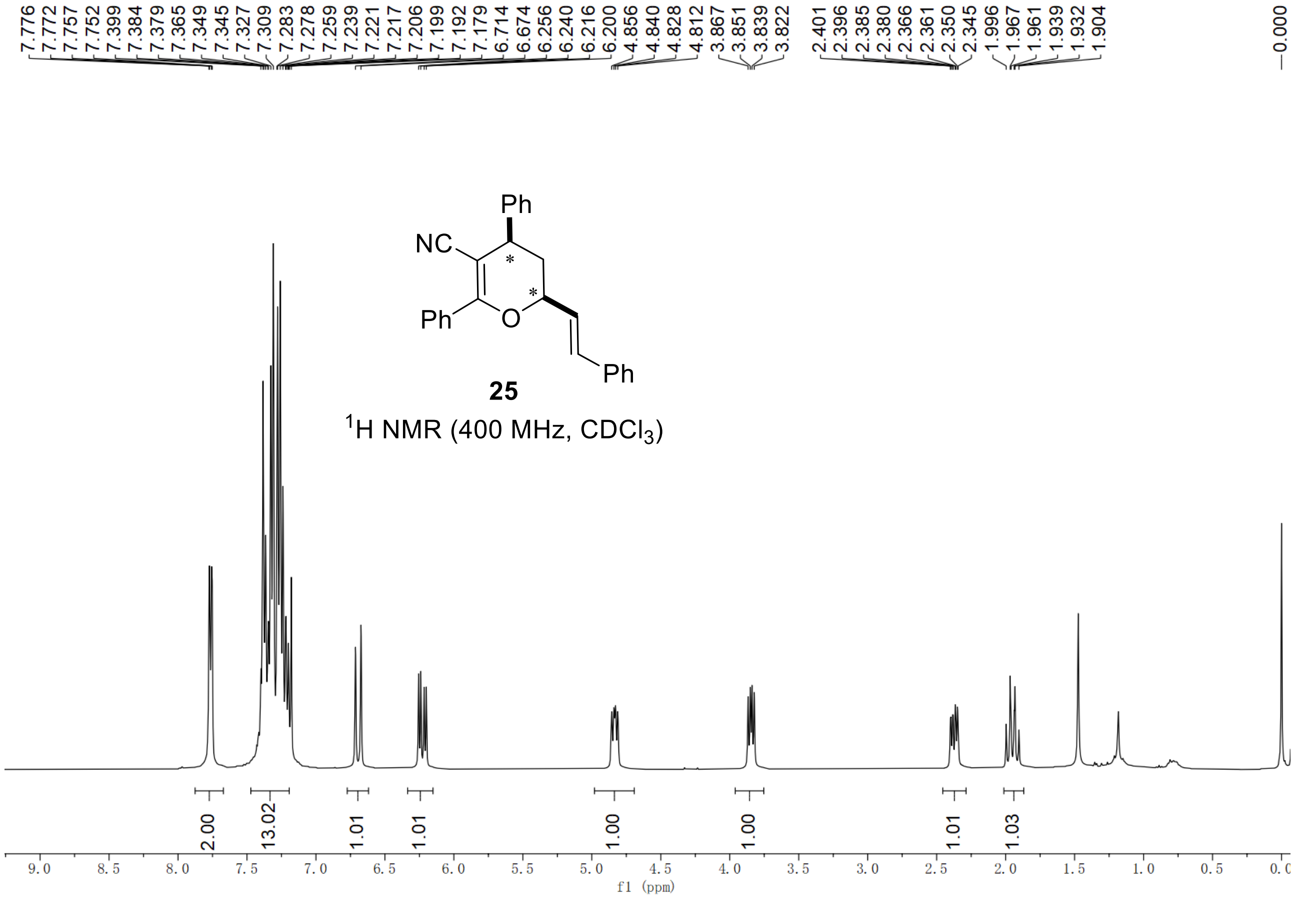
7.776 7.772 7.757 7.752 7.399 7.384 7.379 7.365 7.349 7.345 7.327 7.309 7.283 7.278 7.259 7.239 7.221 7.217 7.206 7.199 7.192 7.179 6.714 6.674 6.256 6.240 6.216 6.200 4.856 4.840 4.828 4.812 3.867 3.851 3.839 3.822 2.401 2.396 2.385 2.380 2.366 2.361 2.350 2.345 1.996 1.967 1.961 1.939 1.932 1.904
0.000
Ph
NC
Ph
O
Ph
25
^{1}H NMR (400 MHz, CDCl$_3$)
2.00 13.02 1.01 1.01 1.00 1.00 1.01 1.03
f1 (ppm)

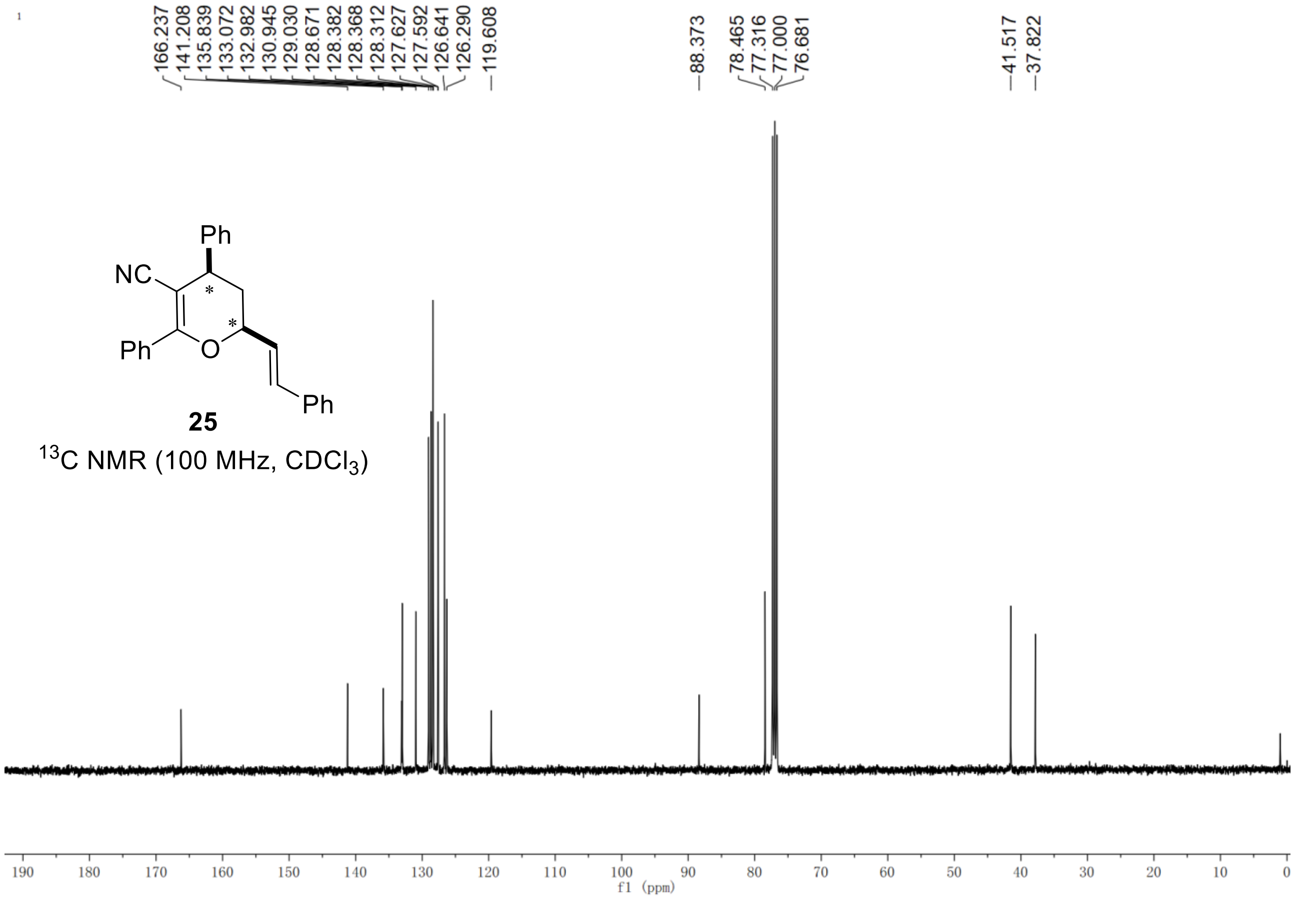
166.237 141.208 135.839 133.072 132.982 130.945 129.030 128.671 128.382 128.368 128.312 127.627 127.592 126.641 126.290 119.608
88.373
78.465 77.316 77.000 76.681
41.517 37.822
Ph
NC
Ph
O
Ph
25
^{13}C NMR (100 MHz, CDCl$_3$)
f1 (ppm)

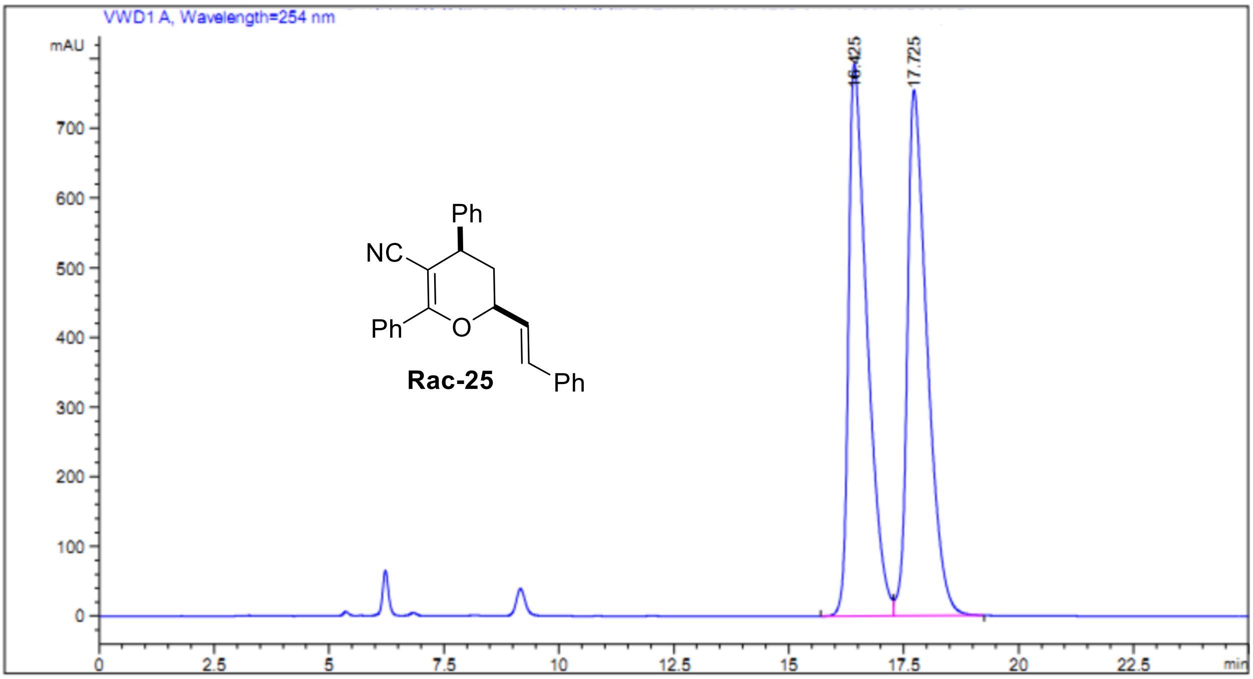

#	[min]		[min]	[mAU*s]	[mAU]	%
1	16.425	BV	0.4148	2.23577e4	793.06830	49.7986
2	17.725	VBA	0.4460	2.25386e4	754.67340	50.2014

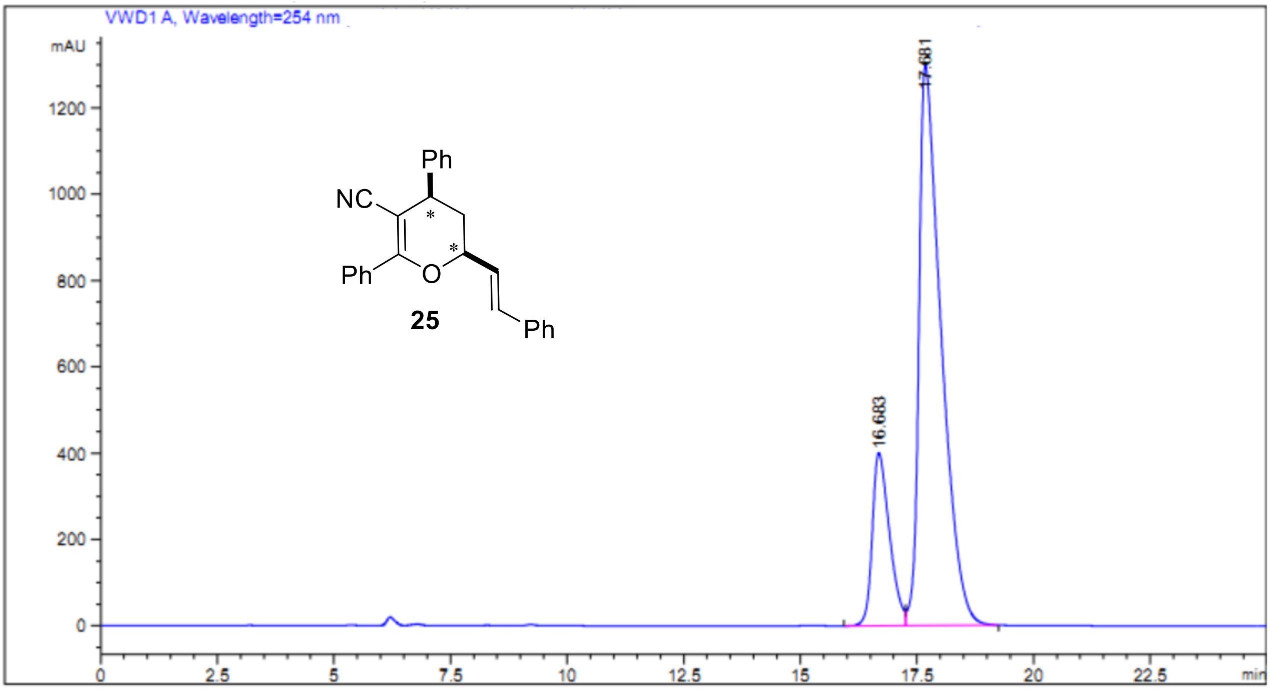

#	[min]		[min]	[mAU*s]	[mAU]	%
1	16.683	BV	0.3874	1.03131e4	400.96591	19.3568
2	17.681	VBA	0.4803	4.29659e4	1299.76550	80.6432

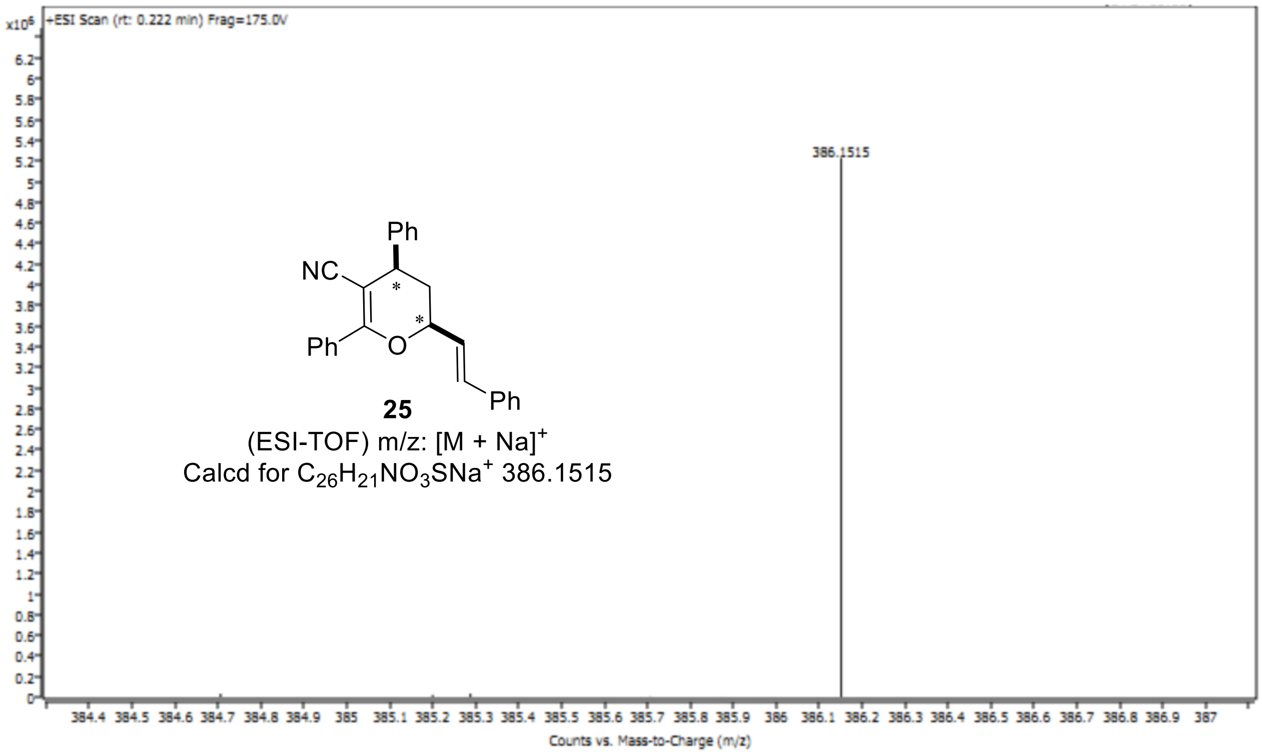
+ESI Scan (rt: 0.222 min) Frag=175.0V
x10^5
386.1515
Ph
NC
*
*
Ph
O
Ph
25
(ESI-TOF) m/z: [M + Na]+
Calcd for C26H21NO3SNa+ 386.1515
Counts vs. Mass-to-Charge (m/z)

9. Computational method

All calculations were carried out with the GAUSSIAN 09 packages[8]. The conformations of intermediates were generated by SYBYL-X 2.0 GA Conf. search module and initially optimized and screened by SYBYL-X 2.0[9]. The structures of diene and its Pd-complexes were calculated at M062X[10–14]/def2-TZVP[15] level and their molecular orbitals were calculated at M062X/def2-TZVPPD. The other geometries of all intermediates and transition states were optimized using B3LYP-D3[16,17] functional together with SDD[18] basis set for Pd atom and the standard 6-31G(d) basis set for the others[19], since these classical methods have been frequently used in the optimization of metal-complex[20–23]. All the optimized structures were calculated after considering various conformations and confirmed by frequency calculations to be either minima or transition states using the same level of theory. For transition states, intrinsic reaction coordinate analysis (IRC) was done to verify that they connect the right reactants[24]. To take solvent effects into account, solution-phase single-point calculations were performed on the gas-phase geometries. The solution-phase single point energy calculations were done using B3LYP-D3 at LANL2DZ for Pd atom and 6-31++G(d, p)[25–27] level for the others. Solvent effect was accounted for using self-consistent reaction field (SCRF) method, using SMD model and UAKS radii[28,29]. Toluene was used as the solvent. Solution-phase single-point energies corrected by the gas-phase Gibbs free energy corrections were used to describe all the reaction energetics. The molecular orbitals were calculated at B3LYP/def2-TZVPPD. All these energies correspond to the reference state of 1 mol/L, 298 K. All energetics reported throughout the text are in kcal/mol, and the bond lengths are in angstroms (Å). Structures were generated using GaussView 6.0[30] and CYLview[31].

8 M. J. Frisch, G. W. Trucks, H. B. Schlegel, G. E. Scuseria, M. A. Robb, J. R. Cheeseman, G. Scalmani, V. Barone, B. Mennucci, G. A. Petersson, H. Nakatsuji, M. Caricato, X. Li, H. P. Hratchian, A. F. Izmaylov, J. Bloino, G. Zheng, J. L. Sonnenberg, M. Hada, M. Ehara, K. T oyota, R. Fukuda, J. Hasegawa, M. Ishida, T. Nakajima, Y. Honda, O. Kitao, H. Nakai, T. Vreven, Montgomery Jr., J. A., J. E. Peralta, F. Ogliaro, M. J. Bearpark, J. Heyd, E. N. Brothers, K. N. Kudin, V. N. Staroverov, R. Kobayashi, J. Normand, K. Raghavachari, A. P. Rendell, J. C. Burant, S. S. Iyengar, J. Tomasi, M. Cossi, N. Rega, N. J. Millam, M. Klene, J. E. Knox, J. B. Cross, V. Bakken, C. Adamo, J. Jaramillo, R. Gomperts, R. E. Stratmann, O. Yazyev, A. J. Austin, R. Cammi, C. Pomelli, J. W. Ochterski, R. L. Martin, K. Morokuma, V. G. Zakrzewski, G. A. Voth, P. Salvador, J. J. Dannenberg, S. Dapprich, A. D. Daniels, Ö. Farkas, J. B. Foresman, J. V. Ortiz, J.

Cioslowski and D. J. Fox, Gaussian 09, Gaussian, Inc.: Wallingford, CT, USA, 2009.

9 Tripos International, Sybyl-X 2.0, Tripos International, St. Louis, MO, USA, 2012.

10 Y. Zhao and D.G. Truhlar, *Theor. Chem. Acc*., 2008, **120**, 215–224.

11 T. Giroday, *Comput. Theor. Chem*., 2014, **1046**, 81–92.

12 B. Yang, Z. Qi, Z. Jing, H. Shuang and H. Zhang, *Org. Electron.*, 2012. **13**, 2568–2574.

13 B. Feng, C. Sun, W. Zhao and S. Zhang, *Environ. Sci-Proc. Imp*., 2020, doi:10.1039/D0EM00112K.

14 M. A. Salvador, F.C.T. Antonio, G.D. da Silva, F. H. Bartoloni, E. Orestes, M. D. Coutinho-Neto and P. Homem-de-Mello, *Theor. Chem. Acc.*, 2020, **139**, 118–129.

15 F. Weigend and R. Ahlrichs, *Phys Chem Chem Phys*., 2005, **7**, 3297–3305.

16 A. D. Becke, *J. Chem. Phys*., 1993, **98**, 5648–5652.

17 C. Lee, W. Yang and R. G. Parr, *Phys. Rev. B.*, 1988, **37**, 785–789.

18 T. H. Jr and P. J. Hay in *Modern Theoretical Chemistry*, Ed. H. F. Schaefer III, Vol. 3 (Plenum, New York, 1977) 1–28.

19 V. A. Rassolov, M. A. Ratner, J. A. Pople, P. C. Redfern and L. A. Curtiss, *J. Comp. Chem.*, 2001, **22**, 976–984.

20 D. Shranami, *J. Org. Chem.,* 2018, **83**, 13911–13921.

21 E. Sultan and K. Duran, *J. Theor. Comput. Chem*., 2020*,* **19**, 2050006.

22 W. Huang, S. T. Chang, H. Huang, C. Wang, L. Chen, K. Chen and M. C. Lin, *J. Phys. Chem. C*., 2020, **124**, 4652–4659.

23 X. Hong, B. M. Trost and K. N. Houk, *J. Am. Chem. Soc*., 2013, **135**, 6588–6600.

24 K, Fukui, *Acc. Chem. Res*., 1981, **14**, 363–368.

25 R. C. Binning Jr and L. A. Curtiss, *J. Comp. Chem*., 1990, **11**, 1206–1216.

26 M. P. McGrath and L. Radom, *J. Chem. Phys*., 1991, **94**, 511–516.

27 L. A. Curtiss, M. P. McGrath, J.-P. Blaudeau, N. E. Davis, R. C., Binning Jr and L. Radom, *J. Chem. Phys*., 1995, **103**, 6104–6113.

28 A. V. Marenich, C. J. Cramer and D. G. Truhlar, *J. Phys. Chem. B*, 2009, **113**, 6378–6396.

29 R. F. Ribeiro, A. V. Marenich, C. J. Cramer and D. G. Truhlar, *J. Phys. Chem. B*, 2011, **115**, 14556–14562.

30 R. D. I. I. Dennington, T. A. Keith and J. M. Millam, GaussView, version 6.0, *Semichem Inc Shawnee Mission KS*, 2016.

31 C. Y. Legault, CYLview, version 1.0b; Universitéde Sherbrooke: Quebec, Canada, 2009; http://www.cylview.org.

diene-*exo*-TS

Zero-point correction=1.067183 (Hartree/Particle)

Thermal correction to Energy=1.134226

Thermal correction to Enthalpy=1.135170

Thermal correction to Gibbs Free Energy= 0.441481

E(Solv) = -1914.74329543

C	0.839968	1.454513	-1.614024
H	1.522270	0.840212	-2.195907
C	3.407484	-3.915645	1.404091
C	4.638859	-3.437017	0.925132
C	4.778586	-2.119921	0.490024
C	3.644756	-1.323059	0.545742
C	2.394102	-1.768360	1.019802
C	2.282689	-3.098111	1.461839
O	3.603684	-0.010070	0.152505
C	2.319965	0.433374	0.407863
C	1.501224	-0.608547	0.901563
C	1.856616	1.707441	-0.004770
N	0.194186	-0.338037	1.028605
C	-1.361998	-2.350722	0.006647
C	-2.712954	-2.576329	-0.258457
C	-3.083607	-3.290110	-1.396628
C	-2.122335	-3.784860	-2.285203
C	-0.770527	-3.554103	-1.994906
C	-0.384554	-2.846802	-0.859477
C	-2.526999	-4.524573	-3.535796
S	-0.937077	-1.437620	1.505515
O	-2.142550	-0.666180	1.845150
O	-0.421829	-2.399593	2.497679
C	2.799900	2.812761	-0.330650
C	2.500055	4.104474	0.126371
C	3.332661	5.180267	-0.174836
C	4.474768	4.982121	-0.951683
C	4.775898	3.702554	-1.422060
C	3.946686	2.625197	-1.117408
H	3.333086	-4.943494	1.746016
H	5.498384	-4.100310	0.896250
H	5.719988	-1.725792	0.122952
H	1.342467	-3.460344	1.855427
H	1.018788	2.020065	0.613791
H	-3.458523	-2.190647	0.427686
H	-4.137588	-3.464099	-1.598044
H	-0.006900	-3.936622	-2.667653
H	0.667353	-2.687355	-0.656929
H	-1.818374	-5.324747	-3.773850
H	-3.521693	-4.969141	-3.432944
H	-2.557087	-3.849019	-4.400739
H	1.609562	4.260460	0.730144
H	3.089238	6.171554	0.196784
H	5.125012	5.818839	-1.190377
H	5.662027	3.541705	-2.029655
H	4.190767	1.634740	-1.482290
C	-0.433174	0.916853	-1.312725
C	-1.512171	1.735963	-0.900533
C	-2.705590	1.227171	-0.479837
H	-2.810278	0.147997	-0.456009
C	-3.841641	1.982174	0.010731
C	-4.027644	3.351561	-0.265570
C	-4.798458	1.319672	0.804937
C	-5.129848	4.031334	0.238681

H	-3.316932	3.873363	-0.899376
C	-5.895913	2.004999	1.314571
H	-4.637977	0.274556	1.050678
C	-6.067403	3.362052	1.031679
H	-5.265102	5.084694	0.010021
H	-6.616586	1.483166	1.937471
H	-6.927217	3.896741	1.425037
H	-1.338960	2.811002	-0.866849
H	-0.604458	-0.149074	-1.402747
H	0.841822	2.498558	-1.920706

----

diene-*endo*-TS

Zero-point correction=0.509294 (Hartree/Particle)

Thermal correction to Energy=0.541050

Thermal correction to Enthalpy=0.541994

Thermal correction to Gibbs Free Energy= 0.441428

E(Solv) = -1914.75064850

C	-0.523863	2.014159	-1.749314
H	-1.455137	2.540589	-1.951680
C	3.317416	-1.889289	1.984249
C	4.290462	-0.892190	1.803332
C	3.942738	0.389902	1.375831
C	2.596255	0.617520	1.136380
C	1.596072	-0.355702	1.323909
C	1.968427	-1.637427	1.753450
O	2.064697	1.806659	0.682937
C	0.698322	1.618676	0.609267
C	0.345441	0.301216	0.960578
C	-0.206796	2.522164	-0.028679
N	-0.936490	-0.063818	0.790256
C	-3.105100	-1.470859	0.522193
C	-4.004773	-0.676320	1.235315
C	-5.317500	-0.573576	0.790333
C	-5.751614	-1.260341	-0.354792
C	-4.831393	-2.055683	-1.044579
C	-3.509291	-2.167880	-0.611442
C	-7.185599	-1.153779	-0.811517
S	-1.414796	-1.605389	1.088548
O	-1.417348	-1.901309	2.532062
O	-0.741730	-2.594768	0.209191
C	0.091943	3.985527	-0.109316
C	-0.941193	4.900204	0.133106
C	-0.718184	6.273095	0.039267
C	0.545014	6.752263	-0.308153
C	1.579378	5.849107	-0.560837
C	1.356289	4.476874	-0.466912
H	3.625120	-2.879474	2.304856
H	5.334507	-1.122563	1.994162
H	4.679643	1.172171	1.228376
H	1.224010	-2.409926	1.896434
H	-1.201028	2.322335	0.369323
H	-3.672151	-0.148326	2.122458
H	-6.022119	0.046556	1.339158
H	-5.152283	-2.597523	-1.930596
H	-2.794308	-2.791638	-1.136091
H	-7.520135	-0.110424	-0.836739
H	-7.319728	-1.577354	-1.811391
H	-7.857418	-1.691157	-0.130217
H	-1.926407	4.528661	0.404124

H	-1.530400	6.966643	0.237997
H	0.722675	7.821311	-0.382632
H	2.565899	6.214761	-0.832008
H	2.166515	3.782559	-0.657740
C	-0.544614	0.598209	-1.946084
C	0.678799	-0.090774	-1.976496
C	0.802615	-1.455445	-1.906633
H	-0.106384	-2.045432	-1.915865
C	2.026154	-2.182719	-1.674022
C	3.303977	-1.589161	-1.756760
C	1.936182	-3.533275	-1.274505
C	4.442264	-2.319764	-1.451493
H	3.400944	-0.552985	-2.062292
C	3.080374	-4.260989	-0.967948
H	0.953704	-3.979687	-1.165729
C	4.336320	-3.658170	-1.056677
H	5.418251	-1.847916	-1.514152
H	2.993051	-5.295895	-0.650519
H	5.231116	-4.225083	-0.815798
H	1.576366	0.522864	-1.921197
H	-1.479689	0.047680	-1.963654
H	0.325470	2.504116	-2.226967

----

com-INT1-2a

Zero-point correction=1.064644 (Hartree/Particle)

Thermal correction to Energy=1.133604

Thermal correction to Enthalpy=1.134548

Thermal correction to Gibbs Free Energy= 0.953350

E(Solv) = -4116.08708905

C	0.169423	2.582070	3.063625
H	0.188231	2.574899	4.149221
C	6.793082	-2.000134	-1.540711
C	6.976074	-1.012198	-2.523020
C	6.402294	0.253586	-2.398933
C	5.641512	0.482012	-1.262972
C	5.431500	-0.488114	-0.267130
C	6.024578	-1.751090	-0.406916
O	5.006544	1.663315	-0.992255
C	4.348959	1.510295	0.220641
C	4.570779	0.145420	0.732644
C	3.560395	2.452945	0.774970
N	3.976005	-0.222111	1.830521
C	2.371952	-1.711613	3.244641
C	2.309468	-1.049157	4.473529
C	1.071361	-0.880768	5.084078
C	-0.106124	-1.354118	4.479870
C	-0.006832	-2.042750	3.267627
C	1.228237	-2.231242	2.645784
C	-1.448082	-1.083191	5.112100
S	3.931125	-1.804193	2.377166
O	5.015770	-2.014060	3.345410
O	3.763775	-2.759609	1.264002
C	3.143979	3.737153	0.243339
C	2.442959	4.609643	1.100879
C	2.005885	5.852484	0.654434
C	2.249440	6.245942	-0.664526
C	2.917672	5.378601	-1.533897
C	3.364031	4.137351	-1.091419
H	7.256744	-2.973053	-1.668660

H	7.577197	-1.236139	-3.399638	H	0.388016	2.339627	0.356014
H	6.527540	1.024808	-3.150279	H	0.861041	0.613376	2.879465
H	5.866102	-2.519317	0.337378	H	-0.186598	3.492165	2.587193
H	3.158790	2.172216	1.743454	Pd	-1.058959	-0.040484	0.217841
H	3.216143	-0.665610	4.928638	P	-2.873107	1.402443	0.087415
H	1.010664	-0.356266	6.034467	P	-1.446197	-2.137176	-0.706974
H	-0.901081	-2.420210	2.782346	C	-2.332911	2.697117	-1.109863
H	1.296885	-2.751173	1.700273	C	-2.533798	2.547086	-2.489926
H	-1.660103	-0.006621	5.113806	C	-1.505832	3.741846	-0.662720
H	-2.254446	-1.581782	4.565688	C	-1.901519	3.398358	-3.397235
H	-1.480726	-1.422440	6.154106	H	-3.182837	1.762588	-2.861964
H	2.240348	4.291634	2.118817	C	-0.878183	4.593627	-1.569563
H	1.475893	6.514769	1.333277	H	-1.341351	3.880250	0.400657
H	1.914210	7.218456	-1.014783	C	-1.063725	4.417679	-2.942730
H	3.092552	5.673172	-2.564900	H	-2.067497	3.261948	-4.462629
H	3.870331	3.466221	-1.772109	H	-0.226063	5.378127	-1.201436
C	0.531241	1.501251	2.350502	H	-0.562482	5.072848	-3.649539
C	0.548673	1.412916	0.896832	C	-4.495946	0.827814	-0.565983
C	1.157176	0.329657	0.240575	C	-5.719418	1.146668	0.037547
H	1.602163	-0.443562	0.853784	C	-4.494049	-0.050743	-1.662044
C	1.618756	0.360860	-1.152066	C	-6.911162	0.607424	-0.450980
C	1.294432	1.403095	-2.043568	H	-5.744861	1.810052	0.894926
C	2.450636	-0.673267	-1.617937	C	-5.684399	-0.572868	-2.160136
C	1.782283	1.400583	-3.346036	H	-3.553484	-0.354109	-2.108408
H	0.652776	2.214479	-1.720527	C	-6.898539	-0.248516	-1.551968
C	2.934375	-0.675260	-2.924415	H	-7.851164	0.859372	0.032825
H	2.733863	-1.467008	-0.933578	H	-5.657857	-1.259637	-3.000455
C	2.603502	0.362214	-3.797848	H	-7.826628	-0.670892	-1.926758
H	1.514803	2.217046	-4.012140	C	-3.323100	2.350653	1.587913
H	3.588523	-1.478822	-3.251152	C	-3.941284	3.610060	1.551420
H	2.981665	0.364102	-4.816316	C	-3.029566	1.769554	2.828705

C	-4.264583	4.270243	2.735938
H	-4.164218	4.071649	0.594039
C	-3.367001	2.424225	4.013930
H	-2.514121	0.814379	2.853422
C	-3.981896	3.675460	3.969282
H	-4.740346	5.246385	2.698600
H	-3.135049	1.964534	4.970666
H	-4.235833	4.191014	4.891325
C	-1.151111	-2.112790	-2.521127
C	-0.969308	-3.273202	-3.289496
C	-1.070099	-0.862593	-3.150746
C	-0.736994	-3.181421	-4.661170
H	-0.983715	-4.246663	-2.809052
C	-0.835772	-0.771228	-4.522151
H	-1.155108	0.038935	-2.553315
C	-0.673473	-1.930325	-5.280734
H	-0.595632	-4.086422	-5.245780
H	-0.755213	0.207339	-4.985606
H	-0.481939	-1.862088	-6.348107
C	-3.174733	-2.748753	-0.508752
C	-3.894646	-2.286184	0.601616
C	-3.802032	-3.617904	-1.409797
C	-5.214676	-2.682395	0.810225
H	-3.419262	-1.578778	1.276120
C	-5.126283	-4.006753	-1.208078
H	-3.265285	-3.975220	-2.282039
C	-5.835107	-3.539558	-0.099300
H	-5.765706	-2.299362	1.664211
H	-5.607212	-4.671497	-1.920740
H	-6.870059	-3.834662	0.049125
C	-0.467695	-3.595476	-0.163925
C	-0.955767	-4.482691	0.805680
C	0.855276	-3.741735	-0.613831
C	-0.132173	-5.483283	1.324715
H	-1.979608	-4.391400	1.155127
C	1.673781	-4.743096	-0.096085
H	1.245213	-3.058820	-1.359986
C	1.184018	-5.613758	0.880239
H	-0.521556	-6.159934	2.080571
H	2.703046	-4.820213	-0.430000
H	1.827752	-6.383034	1.296206

2a-TS1

Zero-point correction=1.065047 (Hartree/Particle)

Thermal correction to Energy=1.132766

Thermal correction to Enthalpy=1.133710

Thermal correction to Gibbs Free Energy= 0.955246

E(Solv) = -4116.04728734

C	-2.473749	1.383475	-0.650999
H	-2.776404	2.001787	0.187922
C	-6.085864	-0.926700	4.305265
C	-6.718288	0.327737	4.242295
C	-6.647061	1.115450	3.093502
C	-5.915219	0.602210	2.031533
C	-5.273661	-0.652408	2.061435
C	-5.365510	-1.431700	3.226525
O	-5.728276	1.237863	0.834650
C	-4.970233	0.377770	0.045031

C	-4.641883	-0.798690	0.751861
C	-4.361247	0.801953	-1.156531
N	-3.822068	-1.694698	0.176051
C	-2.455033	-3.870330	-0.337212
C	-1.836257	-5.073359	0.008719
C	-1.274986	-5.867512	-0.987879
C	-1.322713	-5.481722	-2.333640
C	-1.964233	-4.280230	-2.656899
C	-2.524925	-3.471234	-1.671852
C	-0.667176	-6.312440	-3.407014
S	-3.090098	-2.878102	1.010977
O	-3.985700	-3.756278	1.795103
O	-1.918725	-2.341692	1.759761
C	-4.804888	1.968368	-1.956946
C	-4.613402	1.933033	-3.348512
C	-4.976978	3.009670	-4.153467
C	-5.536785	4.152959	-3.578945
C	-5.719537	4.206643	-2.195269
C	-5.354440	3.130157	-1.388777
H	-6.167004	-1.517275	5.213012
H	-7.274566	0.691487	5.101617
H	-7.130429	2.084068	3.023218
H	-4.895972	-2.406557	3.265981
H	-4.043686	-0.046790	-1.754468
H	-1.807355	-5.388041	1.046103
H	-0.788362	-6.800937	-0.716689
H	-2.010644	-3.963794	-3.695862
H	-3.014457	-2.538781	-1.917303
H	0.286321	-5.860556	-3.707863
H	-0.460985	-7.329459	-3.058384
H	-1.295664	-6.379503	-4.302024
H	-4.176117	1.045031	-3.798301
H	-4.828661	2.954503	-5.228407
H	-5.828504	4.992872	-4.203373
H	-6.151860	5.093172	-1.739144
H	-5.502351	3.180824	-0.317984
C	-1.683354	0.250162	-0.381575
C	-0.826671	-0.364361	-1.335679
C	-0.051420	-1.504989	-1.006480
H	-0.325343	-2.037790	-0.102600
C	0.775661	-2.258654	-1.955318
C	1.128562	-1.766141	-3.226987
C	1.288222	-3.509627	-1.564167
C	1.975405	-2.489381	-4.064461
H	0.756220	-0.799446	-3.552005
C	2.147077	-4.222405	-2.394877
H	1.009545	-3.913509	-0.598628
C	2.499149	-3.717806	-3.650154
H	2.236493	-2.087703	-5.040059
H	2.541868	-5.177076	-2.058586
H	3.170085	-4.274456	-4.298256
H	-0.828059	0.036862	-2.347514
H	-1.727263	-0.209646	0.600818
H	-2.237462	1.945450	-1.548296
Pd	1.125286	0.258267	-0.284435
P	2.826716	-0.799349	0.909939
P	1.417970	2.576017	-0.241040
C	2.433567	-2.507924	1.440959
C	1.155146	-2.750167	1.966246
C	3.313691	-3.582172	1.256297
C	0.762391	-4.043434	2.305721
H	0.440951	-1.941278	2.075942

C	2.914347	-4.878495	1.585385
H	4.300018	-3.409937	0.839206
C	1.640553	-5.111958	2.108695
H	-0.239243	-4.198960	2.690743
H	3.599140	-5.707413	1.427504
H	1.330706	-6.124066	2.354321
C	4.389561	-0.988073	-0.033540
C	4.282988	-1.296148	-1.399382
C	5.659663	-0.823177	0.534449
C	5.427214	-1.434857	-2.181884
H	3.304539	-1.417307	-1.849256
C	6.803970	-0.949296	-0.255612
H	5.754010	-0.586215	1.588822
C	6.690919	-1.253988	-1.613739
H	5.325419	-1.678389	-3.235402
H	7.784284	-0.811968	0.192395
H	7.583561	-1.351705	-2.225458
C	3.331530	0.056444	2.453384
C	3.046319	-0.464434	3.721432
C	3.928243	1.325009	2.357345
C	3.347821	0.271115	4.869784
H	2.587644	-1.442813	3.813979
C	4.242260	2.048194	3.503573
H	4.143640	1.751215	1.383082
C	3.945768	1.526177	4.764934
H	3.116474	-0.143746	5.846977
H	4.693038	3.031250	3.410390
H	4.175610	2.098590	5.659073
C	1.425587	3.248006	1.466062
C	2.045593	4.450441	1.827252
C	0.781954	2.484553	2.452186

C	2.020868	4.881668	3.153944
H	2.561250	5.042330	1.078205
C	0.752553	2.919701	3.775337
H	0.329130	1.535741	2.176314
C	1.374820	4.118456	4.128386
H	2.510297	5.812628	3.427085
H	0.263887	2.312596	4.531274
H	1.364489	4.452568	5.162054
C	0.082818	3.517408	-1.078180
C	-0.838206	4.312662	-0.387065
C	-0.090421	3.315250	-2.458300
C	-1.919701	4.886312	-1.061979
H	-0.719246	4.472768	0.679477
C	-1.163421	3.895997	-3.128925
H	0.612819	2.686572	-2.999450
C	-2.087957	4.678603	-2.429275
H	-2.641070	5.483932	-0.512028
H	-1.294050	3.724718	-4.193723
H	-2.945876	5.097057	-2.943537
C	2.925788	3.252932	-1.040836
C	3.022999	4.588936	-1.460322
C	4.014243	2.394083	-1.245648
C	4.199879	5.058847	-2.042528
H	2.172254	5.253743	-1.344005
C	5.192601	2.864985	-1.825870
H	3.931730	1.350946	-0.967202
C	5.288410	4.200010	-2.220497
H	4.266333	6.094869	-2.363143
H	6.023291	2.181118	-1.975216
H	6.202307	4.570099	-2.676786

2a-TS1'

Zero-point correction= 1.065449 (Hartree/Particle)

Thermal correction to Energy=1.133031

Thermal correction to Enthalpy= 1.133975

Thermal correction to Gibbs Free Energy= 0.955893

E(Solv) = -4116.04858361

C	-2.463182	-2.054054	0.072917
H	-2.412139	-2.892628	0.760543
C	-4.974707	4.311576	-0.980879
C	-5.442168	3.747988	-2.180585
C	-5.498190	2.365288	-2.351785
C	-5.059660	1.585222	-1.290053
C	-4.557074	2.119574	-0.085774
C	-4.532861	3.513946	0.070803
O	-5.068579	0.218263	-1.273382
C	-4.588357	-0.161812	-0.021247
C	-4.204283	0.962869	0.738566
C	-4.384536	-1.511683	0.355927
N	-3.607909	0.767014	1.929666
C	-1.539679	1.006575	3.466597
C	-1.738677	-0.181814	4.175486
C	-0.637484	-0.902246	4.627402
C	0.672649	-0.458510	4.382363
C	0.843715	0.744158	3.689418
C	-0.251026	1.480819	3.232653
C	1.861737	-1.268519	4.839935
S	-2.953619	1.930497	2.849449
O	-3.812015	2.261389	3.999782
O	-2.377496	3.073409	2.091440
C	-5.075125	-2.664156	-0.272054
C	-5.344544	-3.793792	0.518206
C	-5.954703	-4.922439	-0.023995
C	-6.302977	-4.946598	-1.375668
C	-6.033825	-3.832992	-2.174165
C	-5.423142	-2.703061	-1.633160
H	-4.954368	5.392201	-0.873385
H	-5.772474	4.397361	-2.986352
H	-5.870763	1.908734	-3.262707
H	-4.155764	3.952438	0.985021
H	-4.318181	-1.576221	1.439205
H	-2.748182	-0.531549	4.361678
H	-0.792936	-1.817891	5.193421
H	1.845447	1.118010	3.507175
H	-0.107850	2.414836	2.701341
H	2.050055	-2.115207	4.165115
H	2.770729	-0.659925	4.864636
H	1.702607	-1.685052	5.840629
H	-5.071799	-3.777853	1.570588
H	-6.160698	-5.781400	0.608905
H	-6.779870	-5.824417	-1.802648
H	-6.302375	-3.842870	-3.227173
H	-5.223199	-1.841795	-2.258303
C	-1.707852	-0.899865	0.365611
C	-1.531981	0.117349	-0.601215
C	-0.994590	1.394971	-0.324991
H	-1.052026	1.753661	0.695387
C	-0.956406	2.447483	-1.352218
C	-0.486461	2.202210	-2.653923
C	-1.391425	3.739912	-1.020077
C	-0.436799	3.226105	-3.595284

H	-0.112501	1.213989	-2.905765	C	6.020438	-3.202755	0.413916
C	-1.367846	4.756768	-1.973135	H	5.294391	-4.642852	1.843662
H	-1.747231	3.925781	-0.013661	H	6.434807	-1.692692	-1.071984
C	-0.881484	4.509231	-3.257992	H	7.067757	-3.397315	0.626504
H	-0.040712	3.028039	-4.587519	C	0.621443	-3.450396	0.530262
H	-1.725050	5.747852	-1.707082	C	0.156805	-4.669372	0.024303
H	-0.846916	5.308052	-3.993703	C	0.309350	-3.090402	1.850288
H	-1.849286	-0.101537	-1.620052	C	-0.608426	-5.517121	0.828255
H	-1.445650	-0.686700	1.395606	H	0.379053	-4.951164	-0.999596
H	-2.527988	-2.348869	-0.972846	C	-0.437957	-3.945565	2.656202
Pd	0.761730	-0.046534	-0.233065	H	0.618636	-2.122484	2.233001
P	1.570482	-2.244183	-0.471058	C	-0.902513	-5.160638	2.144853
P	2.527797	1.452898	-0.162129	H	-0.978319	-6.454200	0.422050
C	1.364028	-2.820611	-2.203776	H	-0.680266	-3.648428	3.671366
C	2.178835	-3.808401	-2.774669	H	-1.501230	-5.819965	2.766738
C	0.347927	-2.240853	-2.978752	C	3.138370	1.790283	-1.860919
C	1.977809	-4.208297	-4.096403	C	3.875544	2.936320	-2.192336
H	2.972709	-4.258014	-2.187035	C	2.842202	0.858931	-2.867859
C	0.145985	-2.644070	-4.298148	C	4.316355	3.137719	-3.500413
H	-0.275125	-1.468486	-2.539202	H	4.083850	3.682626	-1.432423
C	0.962811	-3.627735	-4.859868	C	3.287814	1.057837	-4.173925
H	2.615821	-4.973060	-4.530385	H	2.241952	-0.012852	-2.626792
H	-0.643952	-2.186748	-4.887260	C	4.026424	2.198734	-4.492675
H	0.811016	-3.938902	-5.889486	H	4.881798	4.032301	-3.745726
C	3.316920	-2.684648	-0.120135	H	3.045808	0.327293	-4.940762
C	3.678137	-3.643309	0.833357	H	4.367380	2.361009	-5.511256
C	4.325323	-1.987947	-0.805567	C	3.999937	0.845911	0.758453
C	5.025731	-3.897625	1.099945	C	3.799807	-0.094610	1.777494
H	2.909705	-4.192234	1.366948	C	5.297447	1.304426	0.498141
C	5.666260	-2.248956	-0.544227	C	4.874636	-0.563403	2.530818
H	4.059299	-1.231040	-1.536864	H	2.798069	-0.469054	1.960031

C	6.374291	0.828055	1.245015
H	5.469740	2.024274	-0.295063
C	6.164607	-0.104509	2.263123
H	4.706557	-1.304803	3.305436
H	7.378233	1.184239	1.031013
H	7.005882	-0.479740	2.838846
C	2.283637	3.118166	0.560790
C	2.633018	3.374743	1.895915
C	1.623106	4.113850	-0.174873
C	2.308797	4.593788	2.488903
H	3.168700	2.624994	2.468185
C	1.309295	5.334700	0.420605
H	1.352648	3.938145	-1.208885
C	1.641781	5.576067	1.753846
H	2.579482	4.776147	3.524983
H	0.787640	6.088438	-0.160978
H	1.383099	6.522957	2.218407

2a-INT2

Zero-point correction= 1.069180 (Hartree/Particle)

Thermal correction to Energy= 1.136198

Thermal correction to Enthalpy=1.137142

Thermal correction to Gibbs Free Energy=0.962215

E(Solv) = -4116.08309942

C	2.432504	0.478853	-2.023929
H	2.652920	-0.574565	-1.843757
C	4.388436	0.153024	4.393642
C	5.057696	-0.952351	3.837015
C	5.184737	-1.099343	2.453948
C	4.609552	-0.109468	1.666550
C	3.916864	0.995027	2.198037
C	3.812972	1.135449	3.588299
O	4.614034	-0.061815	0.300346
C	3.883136	1.074310	-0.054029
C	3.446857	1.762764	1.049328
C	3.640587	1.318883	-1.507588
N	2.721928	2.948360	0.997611
C	0.471286	4.298320	1.011870
C	-0.517918	4.973441	1.725461
C	-1.387850	5.833102	1.055169
C	-1.280104	6.037347	-0.325808
C	-0.249716	5.382799	-1.015109
C	0.619967	4.516426	-0.358137
C	-2.264562	6.908603	-1.064809
S	1.393208	3.009473	1.856825
O	1.487112	3.438611	3.276244
O	0.560430	1.752675	1.697069
C	4.907818	1.124748	-2.330073
C	5.948801	2.051356	-2.179279
C	7.140360	1.909698	-2.885557
C	7.314233	0.831722	-3.758373
C	6.286083	-0.096287	-3.912588
C	5.090833	0.049842	-3.203548
H	4.318706	0.240183	5.474642
H	5.490119	-1.703179	4.492431
H	5.713065	-1.938383	2.011753
H	3.279373	1.981924	4.008208
H	3.354748	2.376116	-1.570050
H	-0.584861	4.828111	2.797771

H	-2.163939	6.352401	1.612468	P	0.313835	-2.396641	-0.353392
H	-0.136387	5.546522	-2.084229	C	-3.360345	0.754100	1.259606
H	1.415585	4.004504	-0.884675	C	-2.434760	1.465351	2.037875
H	-3.016363	6.291201	-1.573903	C	-4.692149	1.184607	1.203978
H	-2.793224	7.584071	-0.384393	C	-2.847860	2.575384	2.773763
H	-1.768863	7.515684	-1.830412	H	-1.380953	1.204187	2.047019
H	5.818622	2.881067	-1.488808	C	-5.094553	2.306004	1.930131
H	7.934824	2.639573	-2.755204	H	-5.411782	0.663620	0.583433
H	8.243233	0.718657	-4.310501	C	-4.176676	3.000324	2.720322
H	6.409669	-0.940634	-4.586304	H	-2.116236	3.110679	3.369145
H	4.308753	-0.689005	-3.330851	H	-6.127218	2.638777	1.871887
C	1.184101	0.891222	-1.306902	H	-4.493661	3.873769	3.283339
C	0.066316	1.437569	-1.944968	C	-4.137363	-1.141102	-0.790641
C	-1.058225	1.849935	-1.186181	C	-4.019231	-0.808960	-2.145931
H	-0.860050	2.161336	-0.164433	C	-5.314346	-1.756008	-0.337322
C	-2.291879	2.381463	-1.779475	C	-5.054161	-1.088114	-3.037622
C	-2.661726	2.149360	-3.116595	H	-3.114873	-0.324594	-2.495709
C	-3.137305	3.168835	-0.980969	C	-6.345790	-2.042458	-1.230935
C	-3.841942	2.676021	-3.632651	H	-5.418450	-2.017486	0.710526
H	-2.027708	1.539948	-3.755095	C	-6.217351	-1.710593	-2.582084
C	-4.321598	3.691342	-1.496249	H	-4.950828	-0.816197	-4.084011
H	-2.853983	3.375858	0.044152	H	-7.250885	-2.523769	-0.871757
C	-4.681667	3.446209	-2.822451	H	-7.023109	-1.934450	-3.275351
H	-4.110611	2.483985	-4.667902	C	-2.759285	-2.043751	1.596283
H	-4.963536	4.287793	-0.854516	C	-2.353086	-1.762470	2.909288
H	-5.605674	3.852152	-3.224149	C	-3.076458	-3.366280	1.252978
H	0.010164	1.400773	-3.032070	C	-2.280174	-2.779565	3.857695
H	1.296381	1.105757	-0.244867	H	-2.091210	-0.751326	3.195662
H	2.316355	0.608618	-3.107431	C	-3.001499	-4.381660	2.206028
Pd	-0.694930	-0.311849	-0.747946	H	-3.388555	-3.610323	0.246124
P	-2.757955	-0.706824	0.331701	C	-2.605666	-4.091468	3.510983

H	-1.953451	-2.544933	4.865659
H	-3.252282	-5.399335	1.920413
H	-2.542860	-4.882961	4.252334
C	0.608917	-2.554624	1.442923
C	0.575967	-3.756710	2.159684
C	0.970638	-1.366654	2.101328
C	0.921509	-3.771908	3.510339
H	0.256469	-4.674829	1.683966
C	1.318341	-1.389691	3.450850
H	0.979271	-0.411719	1.585056
C	1.303969	-2.593700	4.154570
H	0.885915	-4.708454	4.060113
H	1.612782	-0.464258	3.931176
H	1.584943	-2.613276	5.203834
C	1.944708	-2.794889	-1.092221
C	3.131125	-2.693073	-0.357079
C	2.003233	-3.142455	-2.452774
C	4.358593	-2.942503	-0.973808
H	3.105474	-2.402072	0.686515
C	3.229646	-3.398653	-3.060055
H	1.087112	-3.220020	-3.031428
C	4.411902	-3.299602	-2.319391
H	5.273662	-2.831852	-0.402289
H	3.264467	-3.671686	-4.111088
H	5.370474	-3.482021	-2.795853
C	-0.677062	-3.802335	-0.990081
C	-0.317273	-5.143069	-0.783805
C	-1.790954	-3.517675	-1.788524
C	-1.107779	-6.169632	-1.297315
H	0.590942	-5.385667	-0.243103
C	-2.577157	-4.543206	-2.314814
H	-2.047673	-2.485185	-1.988049
C	-2.246238	-5.872712	-2.053110
H	-0.828503	-7.204081	-1.118793
H	-3.446669	-4.295749	-2.917122
H	-2.858318	-6.676895	-2.451387

2a-INT2'

Zero-point correction=1.068496 (Hartree/Particle)

Thermal correction to Energy=1.135904

Thermal correction to Enthalpy=1.136849

Thermal correction to Gibbs Free Energy= 0.959122

E(Solv) = -4116.07921471

C	2.367280	0.213690	-2.115138
H	2.216706	1.295094	-2.181399
C	2.259646	-5.615619	0.430621
C	2.371158	-5.831221	-0.955931
C	2.826935	-4.824878	-1.808534
C	3.147170	-3.608314	-1.218956
C	3.049046	-3.365743	0.163315
C	2.596472	-4.391912	1.005940
O	3.562142	-2.480976	-1.877043
C	3.711526	-1.485944	-0.904427
C	3.420120	-1.967278	0.350287
C	3.725532	-0.066161	-1.372328
N	3.474944	-1.204206	1.513346
C	2.708087	0.156617	3.598082
C	3.970265	0.742612	3.562989
C	4.266729	1.779911	4.450272

C	3.320962	2.239685	5.373525	C	0.576965	-1.583600	-1.715874
C	2.059313	1.625240	5.395218	C	-0.236088	-2.266047	-0.773467
C	1.750658	0.588901	4.519780	H	0.139326	-2.307463	0.245571
C	3.651807	3.348898	6.341956	C	-1.169047	-3.334574	-1.162614
S	2.211483	-1.127319	2.451806	C	-2.021479	-3.206840	-2.271676
O	1.844884	-2.321918	3.263039	C	-1.216712	-4.512158	-0.404464
O	0.981620	-0.556129	1.766553	C	-2.902140	-4.230072	-2.611345
C	4.898446	0.413750	-2.202775	H	-2.010233	-2.283759	-2.845763
C	5.185736	1.785590	-2.227561	C	-2.092949	-5.538864	-0.749042
C	6.227257	2.283227	-3.008954	H	-0.559700	-4.614930	0.451685
C	7.001346	1.412179	-3.778916	C	-2.940206	-5.401734	-1.849435
C	6.722541	0.045131	-3.759398	H	-3.565673	-4.111950	-3.463791
C	5.676773	-0.451962	-2.979057	H	-2.120060	-6.443467	-0.148391
H	1.901874	-6.422682	1.064349	H	-3.630568	-6.198911	-2.110993
H	2.091643	-6.794545	-1.372823	H	0.510823	-1.828221	-2.775260
H	2.914476	-4.971366	-2.880300	H	1.443745	-0.362113	-0.222783
H	2.492279	-4.210154	2.069406	H	2.410289	-0.188945	-3.132826
H	3.695723	0.518855	-0.445546	Pd	-0.856345	-0.140029	-0.759819
H	4.688596	0.387460	2.833607	P	-0.839603	2.158337	-1.143172
H	5.251122	2.241071	4.421270	P	-2.717047	-0.461123	0.599849
H	1.312593	1.962652	6.110713	C	-0.924757	2.532240	-2.939019
H	0.773743	0.117075	4.544687	C	-1.369297	3.772947	-3.421343
H	2.820752	4.056647	6.439951	C	-0.509928	1.554086	-3.854034
H	3.856994	2.951111	7.344423	C	-1.385784	4.030615	-4.791779
H	4.537391	3.907279	6.022588	H	-1.709334	4.532382	-2.724805
H	4.587388	2.463700	-1.623220	C	-0.525165	1.814481	-5.224351
H	6.439704	3.349340	-3.011874	H	-0.177668	0.589601	-3.485617
H	7.816741	1.796027	-4.386033	C	-0.962520	3.053276	-5.695389
H	7.322293	-0.640445	-4.352348	H	-1.731069	4.994781	-5.154067
H	5.462382	-1.515037	-2.968105	H	-0.199262	1.048412	-5.922085
C	1.311255	-0.470728	-1.297509	H	-0.977847	3.255804	-6.762426

C	-2.168787	3.220150	-0.464364
C	-1.927731	4.159677	0.543166
C	-3.471391	3.067147	-0.964930
C	-2.973122	4.944960	1.031488
H	-0.928079	4.276313	0.946596
C	-4.510675	3.854857	-0.477842
H	-3.671506	2.337990	-1.742207
C	-4.262095	4.800735	0.519771
H	-2.776206	5.667754	1.817695
H	-5.513286	3.727022	-0.876093
H	-5.071937	5.415995	0.901555
C	0.716270	2.940421	-0.570293
C	1.307772	4.015764	-1.248669
C	1.339747	2.415970	0.572688
C	2.506678	4.559477	-0.786999
H	0.844826	4.417047	-2.143947
C	2.533271	2.970541	1.036916
H	0.927942	1.551892	1.082387
C	3.118351	4.040540	0.357066
H	2.964745	5.384861	-1.324789
H	3.007282	2.549591	1.917139
H	4.054830	4.462315	0.711300
C	-4.311161	-0.414164	-0.288234
C	-5.552804	-0.553927	0.352411
C	-4.280067	-0.240993	-1.679128
C	-6.736016	-0.495626	-0.383200
H	-5.593203	-0.716362	1.424673
C	-5.463666	-0.185435	-2.415290
H	-3.317682	-0.146851	-2.174860
C	-6.693423	-0.307338	-1.766863
H	-7.691074	-0.601577	0.123185
H	-5.424231	-0.048549	-3.492196
H	-7.616723	-0.263496	-2.337442
C	-2.779292	0.844041	1.897345
C	-1.585176	1.109280	2.589893
C	-3.920465	1.598155	2.191947
C	-1.549918	2.090347	3.579256
H	-0.680120	0.557916	2.355739
C	-3.878521	2.576791	3.186000
H	-4.836537	1.450834	1.634277
C	-2.698599	2.821226	3.887091
H	-0.615283	2.284887	4.097468
H	-4.770445	3.159540	3.397400
H	-2.669916	3.588253	4.656207
C	-2.709342	-2.039836	1.540238
C	-1.595271	-2.302803	2.355040
C	-3.706037	-3.013847	1.407086
C	-1.499128	-3.505887	3.049860
H	-0.770762	-1.602120	2.415249
C	-3.608348	-4.214676	2.113149
H	-4.545935	-2.856635	0.740918
C	-2.513115	-4.460992	2.940648
H	-0.612048	-3.686237	3.649415
H	-4.386230	-4.964341	1.997863
H	-2.438850	-5.401376	3.479991

----

diene-Pd-*exo*-TS

Zero-point correction=1.068229 (Hartree/Particle)

Thermal correction to Energy=1.134899

Thermal correction to Enthalpy=1.135843

Thermal correction to Gibbs Free Energy= 0.961008

E(Solv) = -4116.05953140

C	2.475012	0.399318	-1.391204
H	2.325268	-0.648113	-1.121013
C	3.306996	2.802896	4.678022
C	4.127776	1.692778	4.946241
C	4.607119	0.888478	3.911664
C	4.222728	1.236329	2.623569
C	3.394078	2.340579	2.321521
C	2.936638	3.142769	3.378743
O	4.573909	0.555201	1.485492
C	3.968079	1.227391	0.441978
C	3.250282	2.313997	0.868744
C	3.951464	0.736753	-0.959001
N	2.488506	2.983129	-0.080538
C	0.637964	4.551670	-1.068914
C	-0.682020	4.991775	-0.966172
C	-1.414212	5.236253	-2.122855
C	-0.852450	5.030471	-3.389249
C	0.484570	4.620191	-3.466430
C	1.237563	4.387405	-2.315691
C	-1.693823	5.199275	-4.629678
S	1.498708	4.171319	0.450719
O	2.252548	5.351203	0.917000
O	0.458756	3.671153	1.393962
C	4.849461	-0.447294	-1.256087
C	5.718610	-0.414671	-2.349921
C	6.514502	-1.517574	-2.667674
C	6.451008	-2.672810	-1.887763
C	5.583132	-2.716281	-0.793095
C	4.786963	-1.615757	-0.483201
H	2.956706	3.414264	5.504825
H	4.398942	1.460002	5.972024
H	5.251719	0.033889	4.090311
H	2.312330	4.005371	3.186082
H	4.257051	1.582854	-1.587023
H	-1.137945	5.089562	0.012750
H	-2.453775	5.541654	-2.042567
H	0.939768	4.467899	-4.442131
H	2.261776	4.039220	-2.373030
H	-2.433889	4.391806	-4.700950
H	-2.246818	6.145469	-4.614366
H	-1.082404	5.177665	-5.537222
H	5.773557	0.484367	-2.958851
H	7.185943	-1.470524	-3.520741
H	7.074772	-3.529825	-2.127125
H	5.523026	-3.608955	-0.175991
H	4.117054	-1.663184	0.367062
C	1.341629	1.150963	-0.763858
C	0.144945	1.383472	-1.483647
C	-1.049960	1.788753	-0.820070
H	-0.933304	2.275949	0.140470
C	-2.319514	2.051136	-1.509153
C	-2.588035	1.584272	-2.810141
C	-3.337210	2.747853	-0.829840
C	-3.830060	1.800630	-3.404366
H	-1.822998	1.037905	-3.352857
C	-4.578771	2.956229	-1.421677
H	-3.142424	3.121326	0.169067
C	-4.834580	2.483703	-2.712552

H	-4.016761	1.429833	-4.408817	C	-1.805366	-1.521086	2.725583
H	-5.349630	3.490084	-0.872830	C	-1.337623	-0.947142	3.915227
H	-5.803469	2.647923	-3.175362	C	-1.603082	-2.894769	2.510987
H	0.178714	1.308059	-2.566600	C	-0.667081	-1.727013	4.859229
H	1.259857	1.100223	0.313714	H	-1.487335	0.109688	4.104367
H	2.405763	0.440468	-2.483190	C	-0.947362	-3.672439	3.460784
Pd	-0.990246	-0.378756	-0.430809	H	-1.946800	-3.358745	1.593513
P	-2.472370	-0.494042	1.357218	C	-0.465965	-3.088464	4.634167
P	-0.068935	-2.494291	-0.856219	H	-0.300712	-1.263742	5.770953
C	-2.933432	1.097148	2.138319	H	-0.787579	-4.729129	3.269299
C	-1.913609	2.017638	2.427718	H	0.065229	-3.690975	5.365213
C	-4.268485	1.469973	2.346635	C	1.044215	-2.985747	0.522057
C	-2.219718	3.288953	2.910784	C	1.470761	-4.298518	0.760248
H	-0.873867	1.769842	2.243531	C	1.489726	-1.961570	1.373201
C	-4.574031	2.743671	2.828073	C	2.341508	-4.573750	1.815963
H	-5.068483	0.774351	2.118389	H	1.115740	-5.108762	0.132010
C	-3.553482	3.655514	3.106903	C	2.362559	-2.233602	2.424833
H	-1.409754	3.987979	3.086996	H	1.129425	-0.949669	1.219845
H	-5.612826	3.024927	2.978531	C	2.792919	-3.543652	2.644382
H	-3.796879	4.650955	3.467786	H	2.665713	-5.595686	1.992788
C	-4.084138	-1.235224	0.902745	H	2.698739	-1.425343	3.067321
C	-4.630490	-0.855506	-0.335665	H	3.470528	-3.763259	3.464502
C	-4.759734	-2.170844	1.695250	C	1.025117	-2.483788	-2.334214
C	-5.832806	-1.408011	-0.770691	C	2.325034	-3.000417	-2.345505
H	-4.105666	-0.137078	-0.958653	C	0.532605	-1.843337	-3.483186
C	-5.956799	-2.734471	1.247670	C	3.129314	-2.861898	-3.479301
H	-4.343849	-2.468884	2.652087	H	2.731070	-3.473328	-1.459200
C	-6.494264	-2.355606	0.016078	C	1.327225	-1.724418	-4.620133
H	-6.246038	-1.105477	-1.728501	H	-0.468749	-1.419444	-3.468855
H	-6.470242	-3.466862	1.864457	C	2.633451	-2.225477	-4.615555
H	-7.425333	-2.796007	-0.329524	H	4.151170	-3.224863	-3.454313

H	0.936878	-1.226057	-5.503346
H	3.263996	-2.108570	-5.492310
C	-1.135070	-3.950513	-1.201358
C	-0.641514	-5.115980	-1.808677
C	-2.495774	-3.877315	-0.876135
C	-1.487153	-6.200004	-2.040426
H	0.397630	-5.165205	-2.119937
C	-3.342410	-4.962146	-1.108441
H	-2.897265	-2.963564	-0.457547
C	-2.836902	-6.128241	-1.683681
H	-1.095167	-7.098625	-2.508678
H	-4.393526	-4.882498	-0.846842
H	-3.492730	-6.974236	-1.869303

----

diene-Pd-*endo*-TS

Zero-point correction= 1.066491 (Hartree/Particle)

Thermal correction to Energy= 1.133862

Thermal correction to Enthalpy= 1.134806

Thermal correction to Gibbs Free Energy= 0.954264

E(Solv) = -4116.04662314

C	-1.553581	2.145838	-0.606189
H	-1.484535	2.703343	-1.539959
C	-3.557581	-2.000217	3.872430
C	-3.096030	-1.037788	4.789957
C	-2.898438	0.292081	4.409683
C	-3.171828	0.606408	3.084041
C	-3.651867	-0.332182	2.153645
C	-3.842797	-1.659312	2.552401
O	-2.982157	1.823730	2.482344
C	-3.339783	1.662079	1.139650
C	-3.756310	0.368699	0.883282
C	-2.898929	2.643659	0.125251
N	-3.914863	-0.281351	-0.313939
C	-5.945633	-0.897638	-1.901577
C	-5.619326	-2.103440	-2.524105
C	-6.605758	-3.068646	-2.704672
C	-7.923001	-2.847367	-2.276608
C	-8.225835	-1.628208	-1.658892
C	-7.247116	-0.653011	-1.468989
C	-8.992718	-3.887373	-2.507361
S	-4.666901	0.326059	-1.602373
O	-3.763500	0.257494	-2.777492
O	-5.370333	1.612790	-1.366828
C	-2.619783	4.063741	0.573008
C	-3.216106	5.127033	-0.112338
C	-2.878061	6.449835	0.182920
C	-1.929708	6.727635	1.168298
C	-1.332671	5.672435	1.864197
C	-1.676637	4.354960	1.569436
H	-3.681905	-3.028062	4.199930
H	-2.882527	-1.334386	5.813139
H	-2.540483	1.044380	5.105894
H	-4.189001	-2.395483	1.834643
H	-3.671847	2.666806	-0.642065
H	-4.604081	-2.267462	-2.868811
H	-6.354157	-4.009229	-3.190045
H	-9.242815	-1.438653	-1.323195
H	-7.478830	0.295542	-0.997197
H	-8.596105	-4.902489	-2.393732

H	-9.824709	-3.767681	-1.805631
H	-9.407267	-3.813641	-3.521825
H	-3.944070	4.911308	-0.889816
H	-3.353758	7.261645	-0.360952
H	-1.662140	7.755795	1.397098
H	-0.594746	5.873573	2.636783
H	-1.205842	3.544708	2.113177
C	-1.375851	0.696244	-0.842198
C	-1.040919	-0.162368	0.206819
C	-0.768099	-1.541302	0.024110
H	-1.077137	-1.977264	-0.924103
C	-0.713453	-2.463975	1.164146
C	-0.110123	-2.109855	2.383682
C	-1.305728	-3.731344	1.054228
C	-0.090420	-2.998322	3.452124
H	0.367483	-1.140837	2.481992
C	-1.302309	-4.616312	2.130948
H	-1.775717	-4.014440	0.117165
C	-0.686515	-4.258097	3.330819
H	0.392165	-2.709802	4.381270
H	-1.774785	-5.589357	2.028838
H	-0.673571	-4.950516	4.167688
H	-1.043727	0.235017	1.217450
H	-1.560369	0.287377	-1.830089
H	-0.750690	2.488415	0.045231
Pd	1.109298	-0.340016	-0.488482
P	2.251640	1.746369	-0.533408
P	2.703521	-2.036905	-0.394639
C	2.290953	2.453361	1.164576
C	3.252188	3.395457	1.558017
C	1.299312	2.071428	2.079480
C	3.213432	3.945575	2.839540
H	4.027644	3.697464	0.861602
C	1.258312	2.622836	3.359787
H	0.557975	1.339249	1.783677
C	2.217047	3.563246	3.741494
H	3.961834	4.675928	3.133654
H	0.475943	2.318794	4.049246
H	2.190049	3.995405	4.737623
C	4.004162	1.882556	-1.058888
C	4.401496	2.659424	-2.153149
C	4.968742	1.137390	-0.361893
C	5.740939	2.684445	-2.548333
H	3.668386	3.246771	-2.695113
C	6.303043	1.169848	-0.753950
H	4.674887	0.526355	0.485637
C	6.692423	1.940449	-1.852665
H	6.037518	3.290789	-3.399677
H	7.036001	0.579358	-0.212994
H	7.732845	1.956880	-2.164281
C	1.391098	3.031734	-1.515706
C	0.996516	4.260730	-0.972655
C	1.008305	2.706104	-2.826800
C	0.218086	5.144088	-1.722534
H	1.250939	4.514629	0.049538
C	0.259903	3.603308	-3.585916
H	1.272230	1.735647	-3.240251
C	-0.145310	4.820524	-3.029557
H	-0.128859	6.066288	-1.267481
H	-0.037070	3.338933	-4.596470
H	-0.761787	5.502400	-3.607635
C	3.424738	-2.161799	1.288999

C	4.112337	-3.298154	1.741555
C	3.274165	-1.071385	2.157588
C	4.646419	-3.332369	3.029471
H	4.208191	-4.165666	1.096846
C	3.811213	-1.103600	3.443866
H	2.719163	-0.200660	1.828510
C	4.499538	-2.235650	3.882224
H	5.172677	-4.219726	3.369581
H	3.679186	-0.248165	4.100279
H	4.913099	-2.268532	4.886131
C	4.133905	-1.779510	-1.518873
C	3.932454	-1.003917	-2.668225
C	5.397779	-2.334664	-1.281764
C	4.974898	-0.786461	-3.567815
H	2.958444	-0.553145	-2.838151
C	6.441677	-2.111678	-2.177984
H	5.573218	-2.927511	-0.390717
C	6.232489	-1.337266	-3.321116
H	4.810340	-0.170290	-4.446386
H	7.420906	-2.539514	-1.981762
H	7.050103	-1.157029	-4.013081
C	2.202834	-3.755989	-0.787414
C	2.308100	-4.231017	-2.104523
C	1.622608	-4.576523	0.191344
C	1.850211	-5.505535	-2.432363
H	2.758231	-3.607631	-2.870637
C	1.173179	-5.854424	-0.141733
H	1.522220	-4.224818	1.211206
C	1.283996	-6.322617	-1.450682
H	1.941972	-5.862848	-3.454128
H	0.724031	-6.474535	0.628057
H	0.930462	-7.317278	-1.706772

***exo*-Pd-16**

Zero-point correction=1.070504 (Hartree/Particle)

Thermal correction to Energy=1.137113

Thermal correction to Enthalpy=1.138057

Thermal correction to Gibbs Free Energy= 0.962391

E(Solv) = -4116.10682805

C	2.531249	-0.733242	-0.394626
H	1.694565	-1.424962	-0.352816
C	6.234651	3.387071	3.310184
C	6.931150	2.330587	3.926159
C	6.692266	1.002565	3.571491
C	5.735724	0.788553	2.588785
C	5.026035	1.826389	1.953846
C	5.283529	3.152567	2.320835
O	5.335166	-0.438840	2.107563
C	4.372721	-0.186721	1.158161
C	4.146843	1.151329	1.020693
C	3.702952	-1.312177	0.449515
N	3.116607	1.668573	0.194436
C	4.156050	1.326932	-2.352924
C	3.311581	1.036145	-3.422855
C	3.600470	-0.058408	-4.235729
C	4.713809	-0.870522	-3.984604
C	5.569711	-0.528851	-2.925924
C	5.304498	0.567419	-2.111022
C	4.984668	-2.096716	-4.819323

S	3.670482	2.564965	-1.162491	C	-0.810700	2.791953	-2.729923
O	4.877073	3.253971	-0.701616	C	-1.323126	4.198456	-0.840229
O	2.544868	3.311036	-1.718932	C	-1.561074	3.627862	-3.553547
C	3.281237	-2.430230	1.392598	H	-0.325353	1.919464	-3.155486
C	4.038887	-3.602631	1.457765	C	-2.080443	5.028809	-1.662364
C	3.649677	-4.664429	2.275296	H	-1.229667	4.424007	0.217194
C	2.489763	-4.561833	3.043993	C	-2.208627	4.748077	-3.024414
C	1.733185	-3.389229	2.996523	H	-1.641265	3.403732	-4.614265
C	2.128467	-2.331037	2.178983	H	-2.572370	5.898799	-1.235838
H	6.450900	4.408567	3.609371	H	-2.797225	5.396747	-3.666883
H	7.670777	2.551364	4.690207	H	1.001510	0.897659	-1.891767
H	7.217546	0.174528	4.035242	H	1.640441	0.572793	1.077961
H	4.767986	3.965943	1.824888	H	2.830492	-0.685902	-1.441131
H	4.425748	-1.750803	-0.251876	Pd	-1.127849	0.290907	-0.213552
H	2.441577	1.658917	-3.598507	P	-3.086991	1.064706	0.844190
H	2.947013	-0.291179	-5.072388	P	-1.354637	-1.992647	-0.637211
H	6.454942	-1.131590	-2.739365	C	-2.794841	2.591759	1.824390
H	5.967573	0.833935	-1.296392	C	-1.621078	2.661184	2.591262
H	4.714149	-3.006389	-4.267923	C	-3.641958	3.705019	1.777006
H	4.405396	-2.086810	-5.747630	C	-1.301653	3.817929	3.299458
H	6.045396	-2.179222	-5.079959	H	-0.945665	1.809431	2.606257
H	4.935647	-3.688028	0.849147	C	-3.314121	4.869779	2.473753
H	4.248514	-5.570776	2.306788	H	-4.547199	3.669486	1.180775
H	2.175946	-5.387983	3.675819	C	-2.145565	4.930383	3.234013
H	0.823134	-3.306234	3.580378	H	-0.387121	3.858943	3.884309
H	1.520953	-1.431549	2.136650	H	-3.971785	5.732838	2.417132
C	2.003125	0.642581	0.047309	H	-1.888842	5.840364	3.768728
C	0.871417	1.105745	-0.834615	C	-4.462771	1.547559	-0.277735
C	0.056890	2.173324	-0.436834	C	-4.126832	2.051586	-1.542746
H	0.223665	2.591025	0.553318	C	-5.815303	1.427745	0.073321
C	-0.686030	3.054655	-1.352747	C	-5.124489	2.426852	-2.441270

H	-3.085533	2.142609	-1.826571
C	-6.812131	1.796041	-0.830988
H	-6.088407	1.039028	1.048576
C	-6.469475	2.294560	-2.090221
H	-4.842350	2.821179	-3.412806
H	-7.857064	1.693797	-0.550946
H	-7.248053	2.579556	-2.792600
C	-3.936028	-0.045084	2.041710
C	-3.966267	0.204414	3.418424
C	-4.514847	-1.229037	1.553450
C	-4.560450	-0.712266	4.290012
H	-3.531212	1.116602	3.812693
C	-5.118507	-2.133184	2.420970
H	-4.489383	-1.445078	0.490276
C	-5.138837	-1.879909	3.795125
H	-4.575671	-0.505259	5.356767
H	-5.552233	-3.047125	2.026725
H	-5.598767	-2.592449	4.474041
C	-1.777523	-2.942401	0.882808
C	-2.356584	-4.218107	0.867276
C	-1.526746	-2.326291	2.116422
C	-2.671376	-4.864599	2.062468
H	-2.574485	-4.703333	-0.078354
C	-1.845048	-2.968909	3.311323
H	-1.108093	-1.323858	2.124510
C	-2.416513	-4.241241	3.286004
H	-3.122640	-5.852810	2.038354
H	-1.671793	-2.465454	4.257652
H	-2.675951	-4.740403	4.215409
C	0.060302	-2.935164	-1.341490
C	0.761091	-3.916091	-0.631094
C	0.531306	-2.540500	-2.606010
C	1.918933	-4.484507	-1.170235
H	0.427355	-4.219057	0.354561
C	1.676153	-3.116295	-3.146508
H	0.004468	-1.762525	-3.153056
C	2.378843	-4.087441	-2.424441
H	2.463924	-5.225295	-0.594195
H	2.030887	-2.799642	-4.122865
H	3.279961	-4.530368	-2.840232
C	-2.686972	-2.433758	-1.832575
C	-2.732718	-3.667038	-2.500970
C	-3.692199	-1.490698	-2.084420
C	-3.781385	-3.957425	-3.373599
H	-1.937963	-4.391669	-2.351936
C	-4.742679	-1.780297	-2.955662
H	-3.641311	-0.520307	-1.608154
C	-4.791825	-3.018145	-3.597956
H	-3.807061	-4.916261	-3.884321
H	-5.508822	-1.030871	-3.133940
H	-5.605314	-3.248019	-4.280516

***endo*-Pd-17**

Zero-point correction=1.069223 (Hartree/Particle)

Thermal correction to Energy=1.136377

Thermal correction to Enthalpy=1.137321

Thermal correction to Gibbs Free Energy= 0.957708

E(Solv) = -4116.08424089

C	-1.669585	2.391514	-1.377746	H	-5.623588	2.221104	3.788899
H	-1.904236	2.636011	-2.416387	H	-4.949601	-1.689516	0.702741
C	-5.838688	-0.973891	2.539889	H	-3.330058	3.754982	-1.126764
C	-6.036612	0.117204	3.405871	H	-3.857216	-2.515837	-2.358537
C	-5.491630	1.372041	3.126838	H	-5.506294	-4.217302	-1.593092
C	-4.757130	1.480162	1.955017	H	-8.425516	-1.119811	-1.007709
C	-4.558006	0.407202	1.060395	H	-6.770720	0.580066	-1.779440
C	-5.103138	-0.845864	1.364783	H	-7.594968	-4.639460	-0.259310
O	-4.103699	2.611672	1.509941	H	-8.837440	-3.379638	-0.133393
C	-3.497564	2.263516	0.327928	H	-8.612287	-4.257009	-1.650949
C	-3.749995	0.967408	0.003379	H	-2.681991	5.900326	-0.762576
C	-2.654470	3.211599	-0.454155	H	-1.378296	7.612775	0.463977
N	-3.110268	0.348193	-1.101206	H	0.156202	6.926730	2.301478
C	-5.236216	-0.881554	-2.111607	H	0.351359	4.519650	2.902386
C	-4.860724	-2.225715	-2.066633	H	-0.964427	2.825812	1.684693
C	-5.788399	-3.168436	-1.636219	C	-1.694529	0.858681	-1.228274
C	-7.082945	-2.785541	-1.249217	C	-0.958002	0.273404	-0.036725
C	-7.430033	-1.431063	-1.312124	C	-0.879010	-1.142744	0.019781
C	-6.514126	-0.472427	-1.742979	H	-1.199818	-1.664812	-0.882290
C	-8.083649	-3.818533	-0.794409	C	-1.076230	-1.940116	1.249150
S	-4.011073	0.336342	-2.552372	C	-0.404484	-1.677886	2.454865
O	-3.112735	-0.218751	-3.573155	C	-1.968493	-3.024387	1.217697
O	-4.697358	1.616065	-2.792835	C	-0.598073	-2.480438	3.576367
C	-1.916984	4.245744	0.375580	H	0.316217	-0.868450	2.490784
C	-2.021881	5.600086	0.047381	C	-2.173286	-3.823634	2.341278
C	-1.282599	6.564594	0.734892	H	-2.497542	-3.240491	0.293402
C	-0.424480	6.180953	1.765550	C	-1.482263	-3.562063	3.525863
C	-0.318857	4.830520	2.107884	H	-0.040938	-2.272582	4.486091
C	-1.060898	3.873348	1.418598	H	-2.868043	-4.658168	2.288728
H	-6.267305	-1.938470	2.796592	H	-1.628446	-4.192269	4.398536
H	-6.618323	-0.018612	4.312861	H	-1.108270	0.804249	0.901403

H	-1.303599	0.415784	-2.144863
H	-0.654488	2.734233	-1.192431
Pd	1.099658	-0.336090	-0.263789
P	2.452542	1.569889	-0.303305
P	2.463326	-2.239193	-0.161066
C	2.581764	2.308740	1.379106
C	3.576795	3.236869	1.718139
C	1.683655	1.885262	2.367965
C	3.666150	3.731445	3.019179
H	4.287405	3.561575	0.964245
C	1.781829	2.365811	3.674364
H	0.914701	1.168260	2.101201
C	2.774513	3.291689	4.001576
H	4.439468	4.452034	3.270235
H	1.082265	2.020414	4.430530
H	2.855565	3.668341	5.017387
C	4.233675	1.480633	-0.776403
C	4.779913	2.194957	-1.848935
C	5.061374	0.607712	-0.052083
C	6.123684	2.031609	-2.195821
H	4.159659	2.880235	-2.415994
C	6.400795	0.452164	-0.393186
H	4.653092	0.037291	0.775541
C	6.935907	1.160560	-1.472155
H	6.532680	2.590847	-3.033102
H	7.020994	-0.239384	0.168891
H	7.978468	1.028820	-1.747569
C	1.831983	2.925142	-1.370734
C	1.574069	4.220672	-0.909488
C	1.523254	2.597860	-2.702503
C	1.008454	5.170615	-1.763166
H	1.777577	4.484729	0.121097
C	0.981411	3.553129	-3.558397
H	1.685908	1.582561	-3.056092
C	0.715611	4.842149	-3.086228
H	0.782390	6.160184	-1.380077
H	0.743357	3.286626	-4.584217
H	0.269900	5.581173	-3.745884
C	3.115155	-2.534587	1.534720
C	3.545797	-3.791336	1.984982
C	3.164330	-1.448270	2.419690
C	4.026426	-3.950016	3.284978
H	3.482471	-4.651157	1.325727
C	3.647452	-1.604282	3.718319
H	2.803306	-0.480312	2.091940
C	4.080694	-2.857520	4.153822
H	4.353257	-4.929929	3.621902
H	3.672243	-0.748058	4.386871
H	4.450888	-2.986110	5.167136
C	3.959182	-2.228121	-1.232177
C	3.919297	-1.424308	-2.379740
C	5.118784	-2.962650	-0.954852
C	5.015223	-1.357486	-3.238318
H	3.029798	-0.831649	-2.575227
C	6.218975	-2.889032	-1.808490
H	5.170312	-3.579307	-0.063723
C	6.169174	-2.087185	-2.950698
H	4.977043	-0.717575	-4.114701
H	7.117836	-3.455345	-1.579768
H	7.031025	-2.023986	-3.609095
C	1.691528	-3.853940	-0.579568
C	1.840050	-4.415587	-1.855824

C	0.843357	-4.481516	0.346724
C	1.155454	-5.582040	-2.198306
H	2.495759	-3.943829	-2.580855
C	0.164496	-5.648720	0.001499
H	0.710806	-4.060160	1.336422
C	0.316358	-6.202643	-1.271081
H	1.282968	-6.007777	-3.189906
H	-0.491153	-6.115623	0.730771
H	-0.214781	-7.111958	-1.538698
